# Diaryldiazoketones as Effective Carbene Sources for Highly Selective Rh(II)-Catalyzed Intermolecular C–H Functionalization

**DOI:** 10.1021/jacs.3c14552

**Published:** 2024-03-13

**Authors:** Terrence-Thang H. Nguyen, Aaron T. Bosse, Duc Ly, Camila A. Suarez, Jiantao Fu, Kristin Shimabukuro, Djamaladdin G. Musaev, Huw M. L. Davies

**Affiliations:** Department of Chemistry, Emory University, Atlanta, Georgia 30322, United States

## Abstract

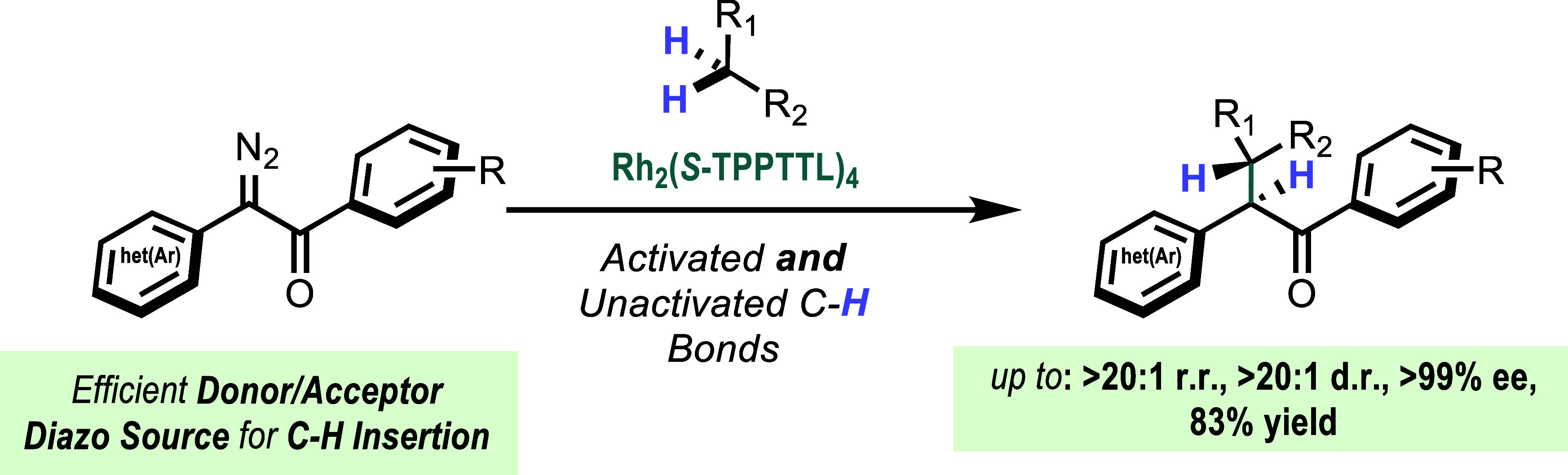

A novel donor/acceptor carbene intermediate has been developed using diaryldiazoketones as carbene precursors. In the presence of the chiral dirhodium catalyst, Rh_2_(*S*-TPPTTL)_4_, diaryldiazoketones undergo highly regio-, stereo-, and diastereoselective C–H functionalization of activated and unactivated secondary and tertiary C–H bonds. Computational studies revealed that the arylketo group behaves differently than the carboxylate acceptor group because the orientation of the arylketo group predetermines which face of the carbene will be attacked.

## Introduction

In recent years, C–H functionalization has evolved to become a powerful strategy for organic synthesis.^1^ Our group has reported the design and development of a variety of chiral dirhodium catalysts that are capable of highly site- and stereoselective C–H functionalization using rhodium-stabilized donor/acceptor carbenes as the reactive intermediates.^2^ Aryldiazoacetates have emerged as a privileged class of diazo compounds that yield superior results for these Rh(II)-catalyzed C–H functionalization.^2,3^ The exquisite selectivity comes from the subtle attenuation of the carbene by the aryl “donor group” and ester “acceptor group” and the absence of either of these groups results in a sharp drop in overall site selectivity and stereoselectivity. Even though intermolecular cyclopropanation of donor/acceptor carbenes with other acceptor functionality, such as ketones, phosphonates, trifluoromethyl, nitriles, and amides, have been reported,^4^ examples of C–H functionalization are limited to intramolecular reactions^5^ or intermolecular reactions with highly activated systems.^4a^ Thus, we were interested in expanding our reaction toolbox by developing new carbene precursors for C–H functionalization, with a particular emphasis on whether altering the acceptor group could alter the selectivity profile of the reaction.

**Scheme 1:**
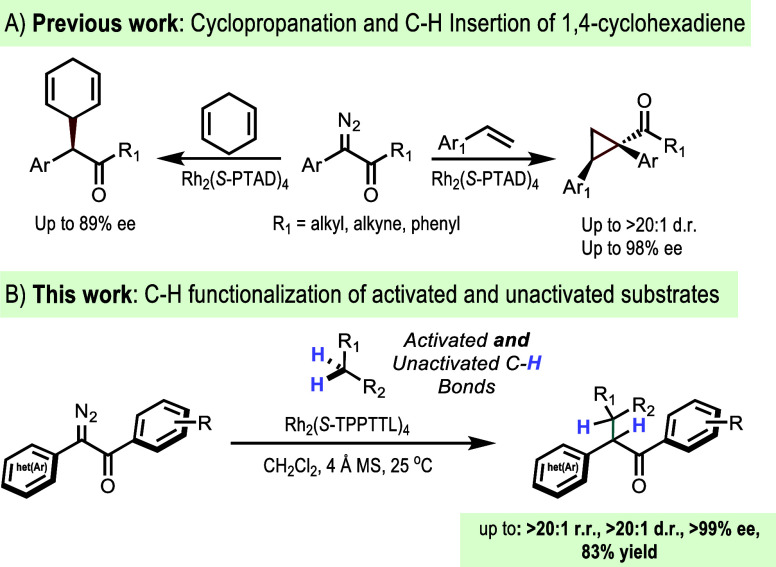
C–H Functionalization Using Aryldiazoketones

Recently, we showed that replacement of the ester with a phosphonate generated a more sterically demanding carbene system with a preference for primary benzylic C–H functionalization.^6^ Previously, we demonstrated that aryldiazoketones are competent carbene precursors for enantioselective cyclopropanation with simple olefins under the catalysis of Rh_2_(*S*-PTAD)_4_ (Scheme 1A).^4a^ The scope of olefins, however, was relatively narrow, and C–H functionalization was limited to only 1,4-cyclohexadiene as the substrate, which is highly electronically favored. This article describes the expansion of the once privileged ester acceptor group to an aryl ketone (Scheme 1B), enabled by one of the more recently developed catalysts, Rh_2_(*S*-TPPTTL)_4_, a dirhodium tetracarboxylate complex that catalyzes the selective functionalization of alkyl cyclohexanes using aryldiazoacetates.^7^ The highlight of this work is the remarkably high levels of site selectivity, diastereoselectivity, and enantioselectivity exhibited by the diaryldiazoketones, far exceeding what had been previously observed with the aryldiazoacetates.

## Results and Discussion

The first stage was to examine whether diazoketones could be broadly applied to a range of C–H functionalization reactions beyond very reactive substrates such as cyclohexadiene. The C–H functionalization of 4-ethyltoluene was selected as the model reaction because it is useful for determining the regio-, diastereo-, and enantioselectivity profile of a rhodium carbene system. Initial optimization was focused on the methyl ketone **1a** using Rh_2_(*S*-PTAD)_4_, previously disclosed by our group for cyclopropanation (entry 1, Table 1).^4a^ The standard reaction, however, proved to be unproductive for C–H functionalization. We hypothesized that the methyl group was not effective either because it had a different electronic profile to the ester or was too small. We changed to the diaryldiazoketone **1b** and found that it results in C–H functionalization, albeit in poor yield and as a mixture of the secondary and primary C–H functionalization products **2b** and **3b** (entry 2, Table 1). Similar trends were seen with electronically differentiated diaryldiazoketones **1c** and **1d** (entries 3 and 4, Table 1).

**Table 1:**
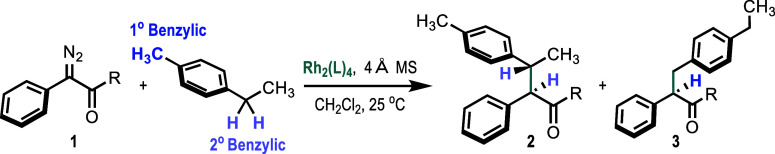

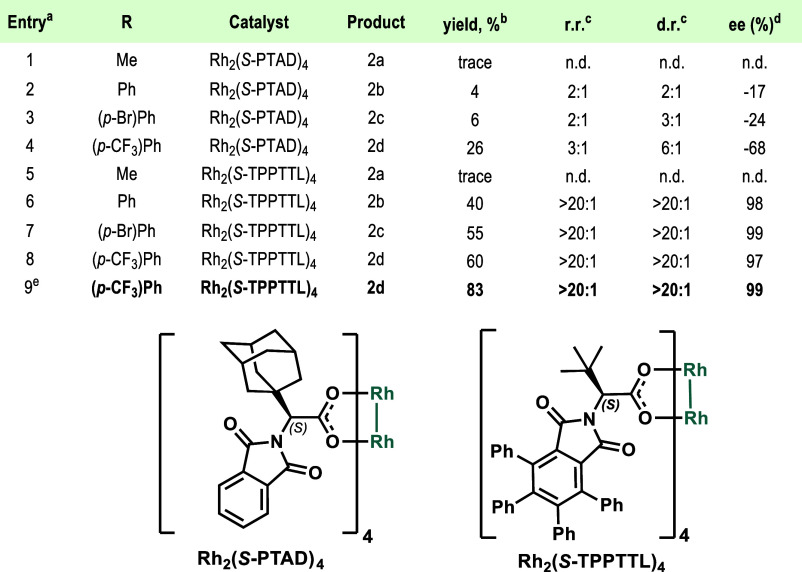
Optimization of the Reaction Conditions

a Reaction conditions: diaryldiazoketone (0.5 mmol) in 2.0 mL of DCM was added over 90 min to a solution of the substrate (5 equiv) and catalyst (0.5 mol %) in 2.0 mL of DCM at 25 °C. The reaction was allowed to stir for an additional 30 min after addition was completed.

b Yield was determined from isolated, spectroscopically homogeneous compounds.

c Regioselectivity and diastereoselectivity were determined from the ^1^H NMR spectrum of the unpurified reaction mixture.

d Enantiomeric excess (ee) data were measured using chiral HPLC analysis of the purified product.

e The reaction was conducted using 0.0625 M DCM, and the diaryldiazoketone and solution with substrate were both degassed before adding the diaryldiazoketone dropwise over 2–3 min.

At this stage, we wished to determine whether some of our newer catalysts could have an impact on improving the intermolecular C–H functionalization reactions with diazoketones. The majority of these catalysts were relatively ineffective, giving the products in a low yield (see Supporting Information Tables S2–S4), but Rh_2_(*S*-TPPTTL)_4_ was found to have exceptional properties in this reaction. The reaction with the methyl ketone **1a** was still not effective but it did generate the desired product **2a** in trace amounts (entry 5, Table 1). When the carbene precursor was changed to the diaryldiazoketone **1b**, the yield improved and **2b** was formed in 40% yield, essentially as a single diastereo- and regioisomer and with very high asymmetric induction (98% ee) (entry 6, Table 1). Similarly, high levels of site selectivity and stereoselectivity were seen with electronically differentiated diaryldiazoketones **1c** and **1d** (entries 7 and 8, Table 1). Finally, further studies were conducted to optimize the yield of the reaction. The main side reactions were carbene dimerization or reaction with oxygen, and these could be minimized by carefully sparging the reaction with nitrogen and lowering the reaction concentration (0.0625 M). Under these conditions, **2d** was formed in 83% yield (entry 9, Table 1).

**Scheme 2:**
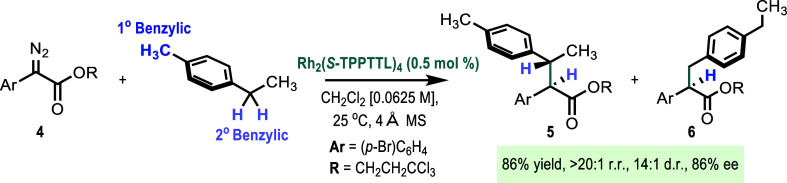
C–H Functionalization Using Aryldiazoacetate

The C–H functionalization with diaryldiazoketones is capable of displaying very high levels of site selectivity, diastereoselectivity, and enantioselectivity. The original goal of this project was to broaden the synthetic potential of the C–H functionalization with a ketone product that could be further manipulated. The optimization studies, however, revealed that diazoketone chemistry has the potential to take the selectivity associated with this chemistry to a new level. In order to illustrate this point, we conducted the parallel reference reactions with the aryldiazoacetate **4** (Scheme 2). As expected, the reaction is also selective. Rh_2_(*S*-TPPTTL)_4_ is not a particularly sterically demanding catalyst and therefore, prefers the secondary site for C–H functionalization to form **5** in preference to **6** (>20:1 r.r.), and the diastereoselectivity (14:1 d.r.) and enantioselectivity (86% ee) are remarkably high but nowhere near as selective as was seen with the diaryldiazoketone **1d** in Table 1, entry 9 (>20:1 r.r., >20:1 d.r., 99% ee).

**Scheme 3:**
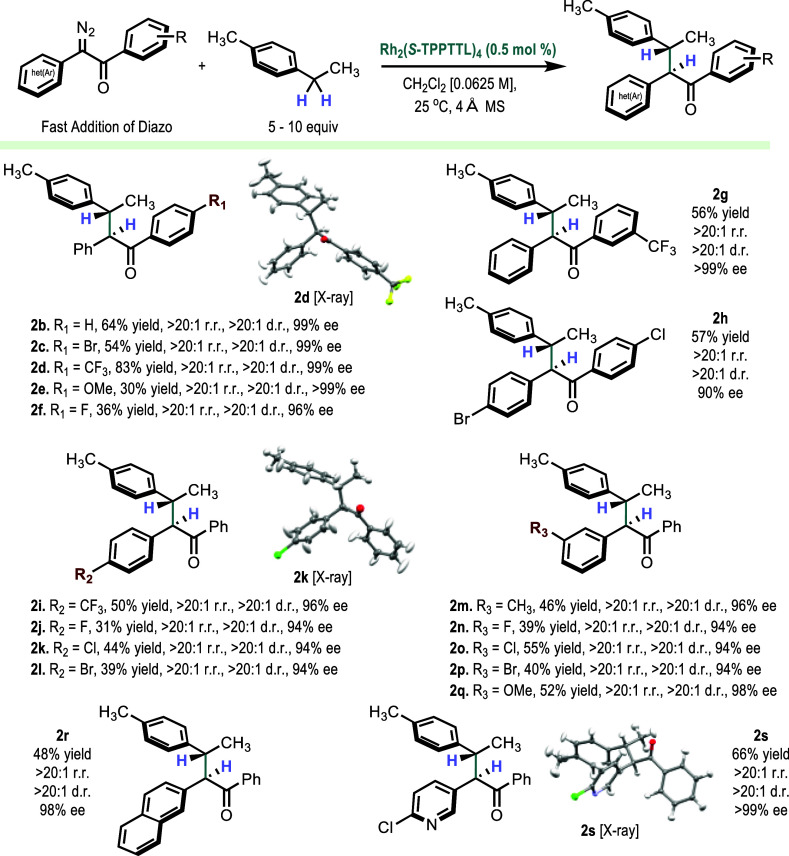
Scope of α-Aryl-α-Diazoketone Reactivity with 4-Ethyltoluene

With the optimized catalyst and reaction conditions in hand, we next examined the flexibility associated with the structure of the diaryldiazoketone (Scheme 3). Starting with variations of the acceptor-side on the diaryldiazoketone, the reaction tolerates substituents in the *para* and *meta* positions, as illustrated in the formation of **2b**–**2g**. In each case, the selectivity is exceptional (>20:1 r.r., >20:1 d.r., >96% ee). One limitation is having a substitution at the *ortho* position because this carbene precursor is unproductive at generating the C–H functionalization product. Placing groups in the *para* position on both sides of the aryl ring to form **2h** still allows the reaction to proceed with exceptionally high site selectivity and diastereoselectivity but with some drop in enantioselectivity to 90% ee. With regard to the donor α-aryl group, *para* and *meta* substitution were well tolerated, resulting in highly regioselective and diastereoselective reactions and the formation of **2i**–**2q** with routinely high enantioselectivity (94–98% ee). The donor aryl groups could also be a naphthyl or a pyridyl, forming **2r** and **2s**, respectively, both with exceptional selectivity and well tolerated in moderate yields. The relative configuration is readily assigned on the basis of the chemical shift of the homobenzylic methyl group because in the minor isomer, this signal is strongly shielded (see the Supporting Information for details).^8^ Additionally, we were able to determine the absolute stereochemical configurations of **2d**, **2k**, and **2s** by X-ray crystallography. The absolute configurations of the other C–H functionalization products are tentatively assigned by analogy. These studies showed that a variety of functionality can be accommodated into the diaryldiazoketones, and all the products are generated with exceptionally high site-, diastereo-, and enantioselectivity.

**Scheme 4:**
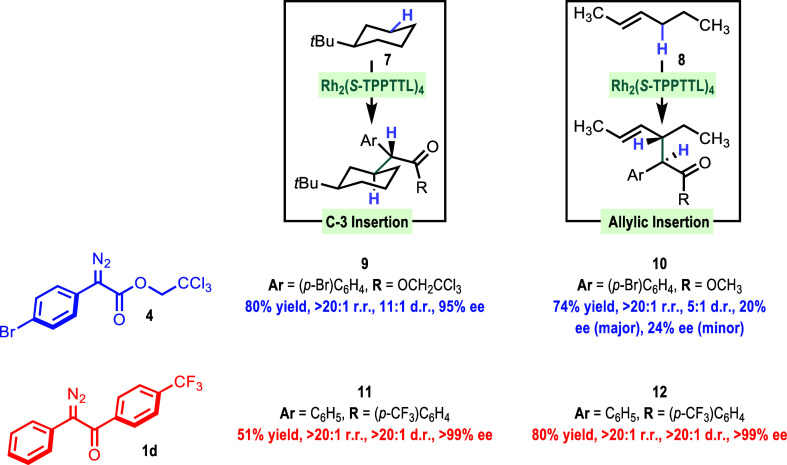
Comparison of α-Aryl-α-Diazoketone vs α-Aryl-α-Diazoacetates

When we began exploring the C–H functionalization with other substrates, it became clear that the diaryldiazoketones displayed selectivity much greater than that of the aryldiazoacetates. This is clearly seen in two head-to-head comparisons with standard substrates **7** and **8**, as shown in Scheme 4. The Rh_2_(*S*-TPPTTL)_4_-catalyzed reaction with *tert*-butylcyclohexane (**7**) is a classic example because it illustrates how interactions with the wall of the catalysts can result in unprecedented site selectivity.^7^ The equatorial C–H at C3, C4, and C5 are sterically and electronically in a similar environment but the Rh_2_(*S*-TPPTTL)_4_-catalyzed reaction with the aryldiazoacetates **4**, preferentially reacts at C3 to form **9** in >20:1 r.r., 11:1 d.r., and 95% ee. In the case of the same reaction conducted with the diaryldiazoketone **1d**, the selectivity is far superior, generating **11** in >20:1 r.r., >20:1 d.r., and 99% ee. The reaction with *trans*-2-hexene (**8**) is also highly significant because it is used to determine primary versus secondary site selectivity, and the reaction with a bulky catalyst favors that the primary C–H insertion products have been used as a key step in a total synthesis.^9^ Rh_2_(*S*-TPPTTL)_4_ is not a bulky catalyst.^7^ Hence, the preferred site selectivity is at the secondary site, although the reaction proceeds to form **10** with poor diastereoselectivity and enantioselectivity. In contrast, the reaction with the diazoketone **1d** forms **12** with exceptional stereocontrol (>20:1 d.r. and >99% ee).

**Scheme 5:**
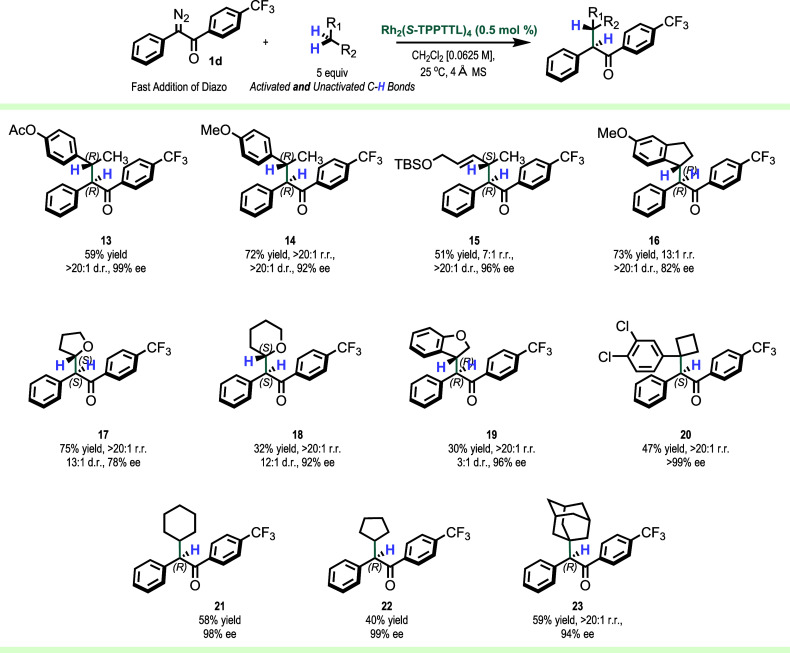
Scope of a Variety of Electronically Activated and Unactivated C–H Functionalization Substrates Using Diaryldiazoketones

The Rh_2_(*S*-TPPTTL)_4_-catalyzed C–H functionalization with the diaryldiazoketone **1d** was then applied to a range of representative substrates, and the results are shown in Scheme 5.^10^ These substrates underwent competent C–H functionalization at the secondary site as seen with the formation of **13**–**23** with >20:1 d.r. and enantioselectivity of >90% ee (apart from Indane **16** and THF **17**) for the major diastereomer. The stereoselectivity was generally far superior to the comparable reaction conducted with aryldiazoacetates (selected comparison reactions with the aryldiazoacetates are described in the Supporting Information, Figure S5). Acyclic benzylic and allylic C–H bonds were especially favorable as seen in the formation of **12–15**, generated with >20:1 d.r., and enantioselectivity of >92% ee. In the case of 3-methoxyindane, there was a strong preference for the formation of **16**, the product derived from C–H functionalization to the benzylic site *para* to the electron-donating methoxy group. Furthermore, the diastereoselectivity was very high (>20:1 d.r.), although the enantioselectivity was slightly lower (82% ee). Tetrahydrofuran and tetrahydropyran, even though electronically favorable, are challenging substrates for diastereoselective C–H insertion due to less steric differentiation between the adjacent oxygen atom and CH_2_ group, as can be seen in the formation of **17** and **18**.^11^ However, we were pleased to see improved diastereoselectivity (13:1 d.r.) in the formation of **17** with tetrahydrofuran over the analogous reaction with an aryldiazoacetate (1:1 d.r.),^11^ albeit with slightly lower enantioselectivity (78% ee). The reaction with benzodihydrofuran preferentially occurred at the benzylic site to form **19**, but in this case, the product was obtained with poor diastereocontrol (3:1 d.r.). The C–H functionalization can also occur at activated tertiary sites, as illustrated with an arylcyclobutane to form the C–H insertion product **20** with >99% ee.

The arylcyclobutane system is a good test for the steric encumbrance associated with the catalysts because sterically crowded catalyst preferentially reacts at the methylene site at C3.^12^ The formation of the tertiary C–H functionalization product is a further indication that the Rh_2_(*S*-TPPTTL)_4_/aryl ketone carbene system is not particularly sterically demanding. A few examples of unactivated substrates were also examined to form **21**–**23**. Cyclohexane and cyclopentane generated the C–H functionalization products **21** and **22** in >98% ee, whereas adamantane preferentially reacted at the tertiary site to form **23** in 94% ee. As Rh_2_(*S*-TPPTTL)_4_ is not particularly steric demanding, this carbene catalyst combination is not suitable for functionalizing *n*-alkanes because a mixture of regioisomers and diastereomers would be formed.

**Scheme 6:**
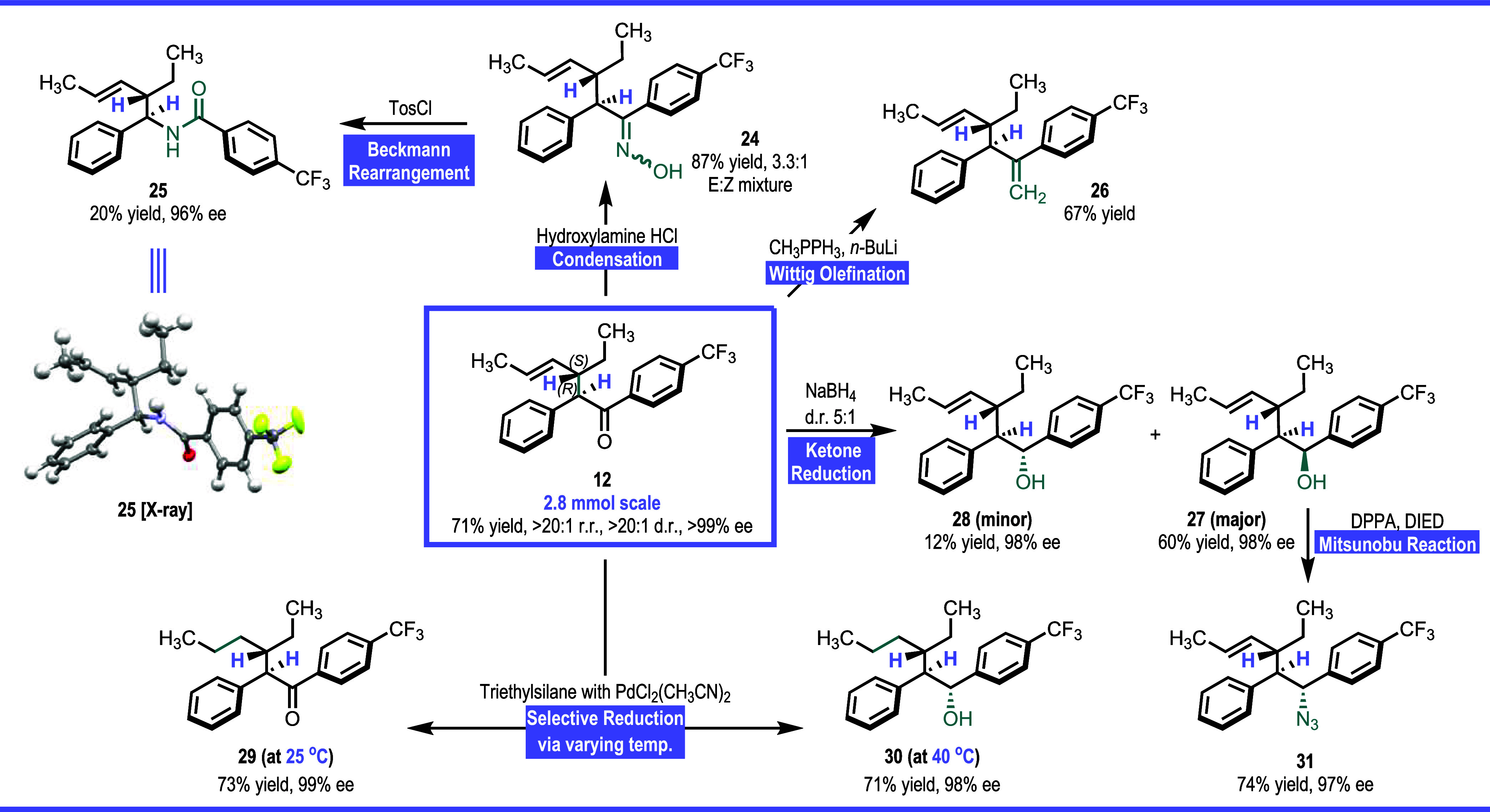
Applications of C–H Insertion Products

Having established a robust profile of substrates competent under our new diazoketone carbenes, we then sought to demonstrate its utility in postfunctionalization transformations. Notably, the transformation outlined in Scheme 6 are chemoselective for the ketone functional group, and thus these compounds would not be accessible using our standard aryldiazoacetate. Starting with the C–H insertion on *trans*-2-hexene, the reaction could be readily conducted on 2.8 mmol scale to form **12** in 71% yield with no change in site selectivity, diastereoselectivity, or enantioselectivity. Condensation of **12** with hydroxylamine generates ketoxime **24** in 87% yield as a mixture of *E*/*Z* isomers in a ratio of 3.3:1. Subjecting ketoxime **24** to Beckmann-rearrangement conditions^13^ with tosyl chloride furnished a 20% yield for desired benzylamide **25**. Since the desired ketoxime was approximately 20% of the *E*/*Z* mixture, it is hypothesized that the minor isomer rearranges cleanly to the benzylamide, while the major ketoxime isomer generates other types of products. Notably, the chiral benzylamide formed is generated in 96% ee, the relative and absolute configuration of which was confirmed by X-ray crystallography. A Wittig reaction on **12** readily formed the diene **26** in 67% yield as a single diastereomer, which indicates that no epimerization had occurred. Palladium-catalyzed reduction of **12** with triethylsilane resulted in the formation of ketone **29** in 73% yield. Under more forcing conditions, both the ketone and the alkene functionality were reduced to form predominately the alcohol **28** in 71% yield. In contrast, reduction of **12** with sodium-borohydride resulted in the preferential formation of alcohol **27** over the diastereomer **28** (5:1 d.r.) with the opposite configuration at the alcohol stereogenic center compared to the silane reduction. Further opportunities for diversification are possible from the alcohol **27** because it readily underwent a Mitsunobu reaction to generate the azide **31** with 97% ee. In all of these reaction, there appeared to be virtually no epimerization of the stereogenic center adjacent to the carbonyl group.

**Figure 1:**
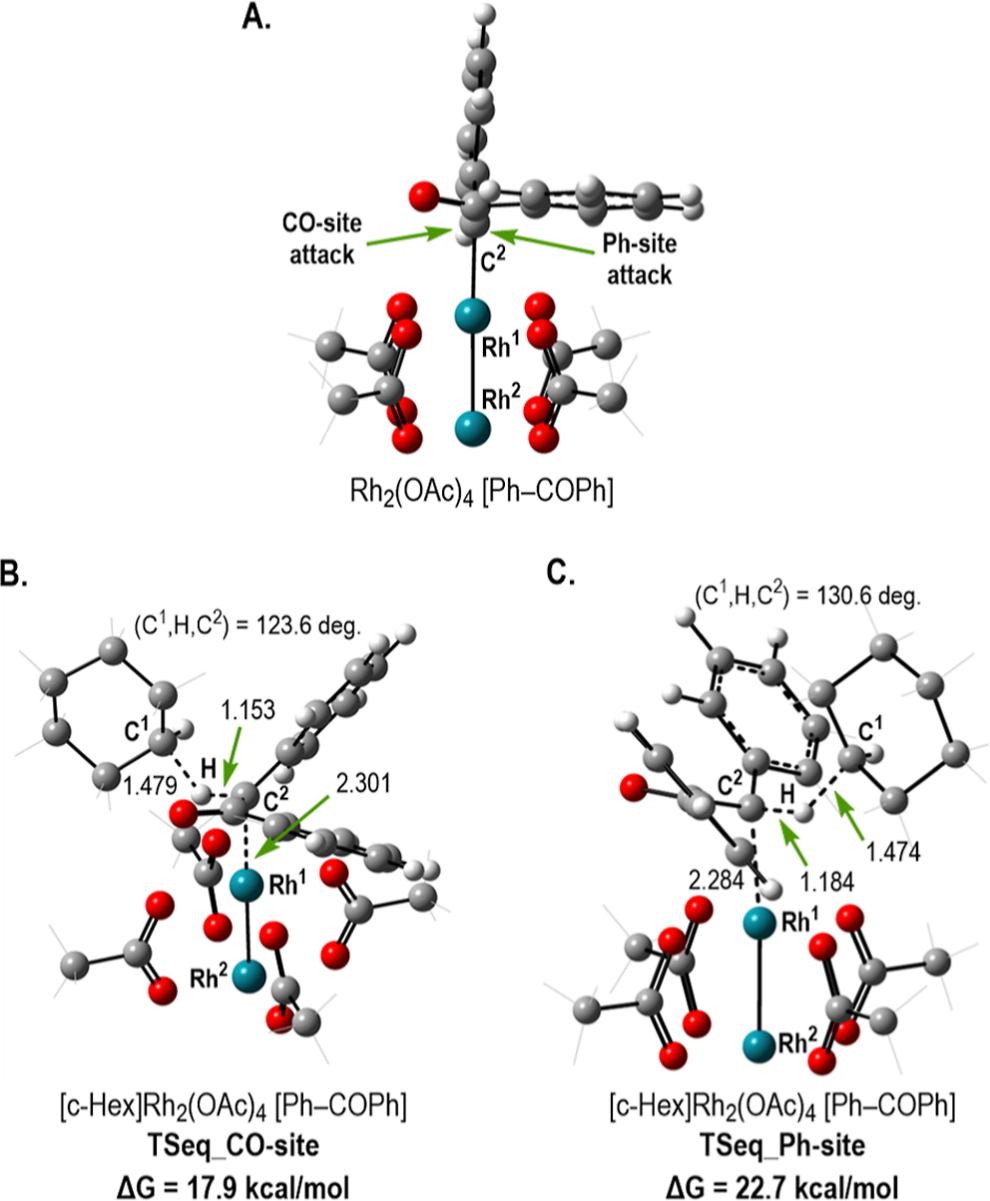
Calculated diarylketo carbene, Rh_2_(OAc)_4_[Ph–COPh] (A), and transition states for the carbene insertion into the equatorial C–H bond of the cyclohexane from the carbonyl (B) and aryl sites (C). Distances are given in Å. Analogous transition states for the carbene insertion into the axial C–H bond of the cyclohexane are given in the Supporting Information Section S9.

Even though the diastereoselectivity and enantioselectivity of aryldiazoacetates have generally been considered quite exceptional, it is clear that the diaryldiazoketones are far superior and, in particular, perform exceptionally well with the chiral Rh_2_(*S*-TPPTTL)_4_ catalyst. We have conducted computational studies to understand the differences between aryldiazoacetates and diaryldiazoketones. We first examined the binding of the two types of carbenes to dirhodium tetraacetate (which is selected as a model system). In the case of aryldiazoacetates, it is well established that (a) the aryl group of the donor group aligns in the plane of the rhodium carbene bond, whereas the acceptor group is orthogonal,^13^ and (b) the substrate can approach either from the side of the carbonyl or the alkoxy group, and the selectivity varies depending on which side it attacked.^13e^ The presented calculations [see Supporting Information for details of the used DFT approach, as well as (a) 3D structures of reactants, multiple transition states, and products of the reaction of (AcO)_4_Rh_2_-(diarylketo carbene) and (b) Cartesian coordinates of all calculated structures] show that diarylketo carbene coordination motif to the dirhodium core is the same as that in the case of aryldiazoacetates (see Figure 1). In the case of the diarylketo carbene, however, it is evident that the orthogonally positioned aryl group will strongly block attack of the substrate from its side. Hence, the approach of the substrate to the diarylketo carbene will be more stringent because the substrate can only approach on the side of the orthogonal keto functionality. This expectation is fully supported by the transition state calculations performed at the DFT level of theory with cyclohexane as a substrate. Indeed, as seen in Figure 1, carbene insertion into the equatorial C–H bond of the cyclohexane occurs with only 17.9 kcal/mol free energy barrier, while the same process needs 4.8 kcal/mol more energy if the attack occurs at the aryl side.

**Figure 2:**
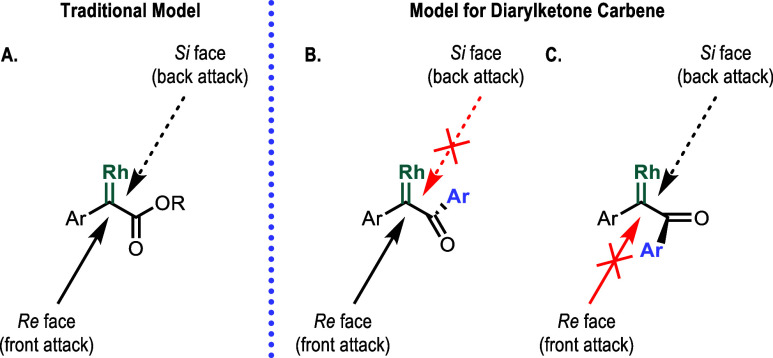
Comparison of the traditional model for asymmetric induction versus the new diarylketone model. In the case of the arylacetate (A), attack of the substrate can occur from either the carbonyl or alkoxy side, whereas in the case of the aryl ketone, (B, C), the attack is predetermined by the configuration of the carbene because attack at the aryl side is blocked.

The demanding constraints on the approach of the substrate to the carbene require a different way of analyzing the enantioselectivity of the reaction (Figure 2). Normally when considering the enantioselectivity of a carbene reaction, one analyzes how the chiral ligands of the catalyst influence which face of the carbene it attacked, as shown in structure (A).^13^ In the case of the aryl ester carbenes, we showed the situation was a bit more complicated because the two orientations possible for the orthogonal ester group gave different levels of enantioselectivity, but approach was possible from either side.^13e^ In the case of the diarylketo carbenes, the influence of the catalyst on the orientation of the orthogonal group will be crucial because the stereochemical outcome is predetermined by which aryl ketone orientation [structure (B) or structure (C)] is involved in generating the product (Figure 2B).

**Figure 3:**
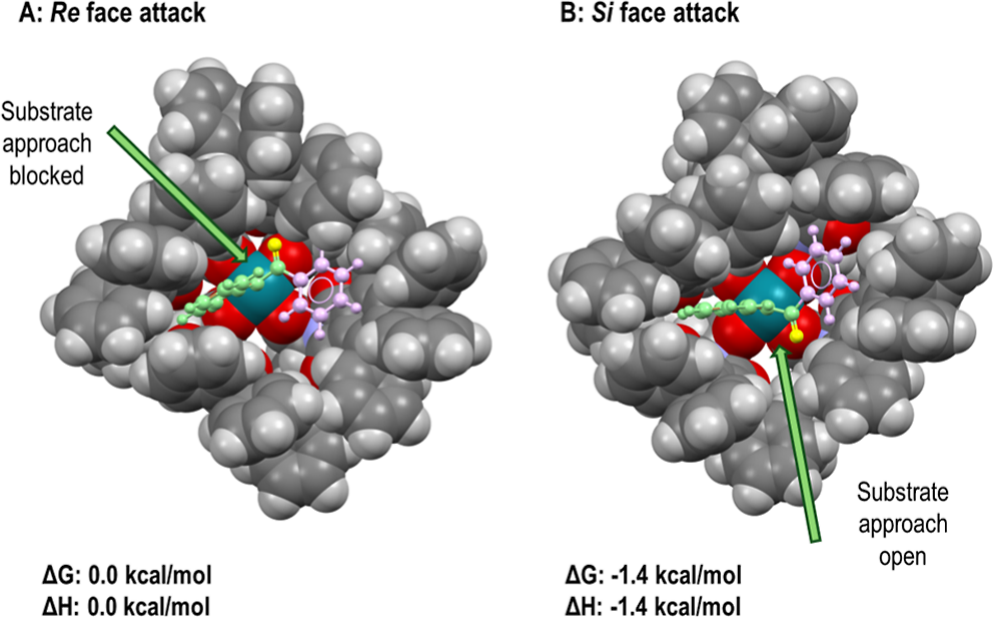
Calculated structures with relative energies of the Rh_2_(*S*-TPPTTL)_4_/diarylketo carbene complex to rationalize preferred Si face attack (B).

The second feature that needs to be determined is why the diarylketo carbenes perform so well when Rh_2_(*S*-TPPTTL)_4_ is used as the catalyst. Obtaining a definitive computational answer for this system is challenging because the dirhodium catalyst is so large. Previous studies, however, have shown that Rh_2_(*S*-TPPTTL)_4_ has special properties because the catalyst self-assembles into a C_4_-symmetric bowl-shaped structure with 16 phenyl groups on the periphery of the bowl.^7^ These phenyl groups are considered to preferentially tilt one way, leading to an induced helical chirality. Both of the aryl groups in the diarylketo carbenes are electron deficient and, therefore, would be expected to be involved in π-bonding to the aryl rings of the catalyst. Computational analysis of the diarylketo carbene bound to Rh_2_(*S*-TPPTTL)_4_ revealed that the keto aryl glides in-between two of the ligands, setting up an opportunity for π-stacking. Once it is locked in that position, it will force the substrate to approach from the side of the carbonyl, leading to a well-defined stereoinduction. The two most stable orientations are shown in Figure 3. Each one would result in the formation of a different enantiomer of the product, with the lowest energy structure, (B), resulting in the formation of the product with the observed absolute stereochemistry. Due to the size of the catalysts, a computational study to determine the energy of the transition state for reactions proceeding from (A) or (B) has not been determined. However, a visual examination of the structure (A) and (B) clearly indicates that attack of a substrate to the keto side of (B) is far more open than attack to the keto side of (A). However, not calculated at this stage, presumably the stringent demand of the approach of the substrates causes the site selectiveness and diastereoselectivity to be exceptional as well. More extensive computational studies will be conducted in the future to gain further insights into this remarkable level of stereocontrol.

## Conclusions

In conclusion, we have demonstrated donor/acceptor carbenes with a ketone as the acceptor group are competent in functionalizing a wide array of activated and unactivated C–H bonds. Optimization of the ketone/catalyst pairing led us to a highly selective system using a diaryldiazoketone and Rh_2_(*S*-TPPTTL)_4_. Following the C–H functionalization, we demonstrated that this new ketone handle can be used to access a novel chemical space. This space includes a plethora of functionalized chiral building blocks with orthogonal handles, useful for medicinal chemistry libraries and total synthesis applications. Ultimately, the development of the diaryldiazoketone not only expands our toolbox of carbenes compatible under dirhodium-catalyzed C–H functionalization but also demonstrates an advancement in site selectivity over the standard aryldiazoacetates.

## Supporting Information Available

Complete experimental procedures, materials, computational details, and compound characterizations; and crystal structure for compounds **2d**, **2k**, **2s**, and **25** (PDF)
