# Supplementary material for: Diaryldiazoketones as Effective Carbene Sources for Highly Selective Rh(II)-Catalyzed Intermolecular C–H Functionalization
Source: J Am Chem Soc. 2024 Mar 13;146(12):8447–55. doi: 10.1021/jacs.3c14552 (PMC10979447; doi:10.1021/jacs.3c14552)
Supplement: Supplementary file 1 — ja3c14552_si_001.pdf [file ja3c14552_si_001.pdf]

Diaryldiazoketones as Effective Carbene Sources for Highly Selective Rh(II)-catalyzed Intermolecular C-H Functionalization

Terrence-Thang H. Nguyen#, Aaron T. Bosse#, Duc Ly, Camila A. Suarez, Jiantao Fu, Kristin Shimabukuro, Djamaladdin G. Musaev* and Huw M. L. Davies*

Department of Chemistry, Emory University, 1515 Dickey Drive, Atlanta, Georgia 30322

Corresponding author email: hmdavie@emory.edu

Supporting Information

Complete experimental procedures, materials, computational details, and compound characterizations

Table of Contents

1. General Considerations 3

2. Catalyst Structures 4

3. Acquisition of Compounds 6

4. Experimental Procedure 8

A. Preparation of Weinreb Amides 8

General Procedure A for the Synthesis of Weinreb Amides 8

B. Preparation of Ketones 16

Procedure to Wash Magnesium Turnings: 16

Preparation of 1 M Grignard Solutions: 17

General Procedure B for the Synthesis of Ketones 17

C. Preparation of Aryldiazoketones 27

General Procedure C for the Synthesis of Aryldiazoketones 27

D. Asymmetric C–H Insertion and Product Characterization 42

General Procedure for the Aryldiazoketone C-H Insertion Reactions 42

Crude ^{1}H NMR of Initial Catalyst Screen for the Aryldiazoketone C-H Insertion 44

Comparison of C-H Insertion Reactions with Diazoketones Versus Diazoacetates 46

Challenging Substrates for C-H insertions with Diazoketones 47

Product Characterizations 47

E. Application of Aryldiazoketones and Characterization 82

i. General Procedure For Beckmann-Rearrangement and Characterization 82

ii. General Procedure for Wittig Olefination and Characterization 86

iii. General Procedure for Ketone Reduction and Characterization 87

iv. General Procedure for Selective Reduction and Characterization 89

v. General Procedure for Mitsunobu Reaction and Characterization 91

5. Crude C-H Insertion ^{1}H NMR Spectra for r.r. and d.r. Determination 93

6. NMR Spectra 108

7. HPLC/SFC Data 178

8. Determination of Absolute Configuration and X-ray data 207

9. Computational Procedure 210

10. References 238

1. General Considerations

Warning: This project involves the use of diazo compounds. Diazo compounds are known to have thermal stability issues and are explosive hazards; work with diazo compounds should be performed in a well-ventilated hood, require the use of PPE, and careful handling of the reagents.[1]

The absolute configuration is tentatively assigned by analogy based on X-ray crystallography. Reactions were carried out under nitrogen in flame-dried vessels unless otherwise specified. Dichloromethane, diethyl ether, tetrahydrofuran, and toluene were purified using a *Glass Contour Solvent System.* Dichloromethane used for C–H functionalization reactions was distilled under nitrogen from calcium hydride onto 4Å molecular sieves and stored under nitrogen for 24 h prior to use. Flash column chromatography was performed on Silicycle SiliaFlash P60 silica gel (60 Å pore size, 40–63 µm particle size, 230–400 mesh) and ACS reagent grade solvents. Reactions were monitored by thin layer chromatography (TLC) carried out on with aluminum-sheet or glass-backed silica gel plates, visualizing with UV light, and staining with aqueous KMnO4. Deuterated chloroform ($CDCl_3$) was neutralized with barium carbonate and filtered before use when acquiring NMR spectra of diazo compounds. All ^{1}H NMR spectra were recorded at either 400 MHz, 500 MHz, 600 MHz, or 800 MHz on Varian/Bruker-400, Varian-500, or Varian/Bruker-600/800 spectrometers. ^{13}C NMR spectra were recorded at either 101 MHz, 126 MHz, 151 MHz, 201 MHz on Varian/Bruker-400, Varian-500, Bruker 600/800 spectrometers. ^{19}F NMR spectra were recorded at 282, 376, 565, or 753 MHz on Varian-300, Varian/Bruker-400, Varian/Bruker-600/800 spectrometer. NMR spectra were obtained from solutions of deuterated chloroform ($CDCl_3$) with or without 0.03% TMS with residual solvent serving as internal standard (7.26 ppm for ^{1}H or 0.00 ppm and 77.16 ppm for ^{13}C) or deuterated benzene (C_6D_6) (7.16 ppm for ^{1}H and 128.06 ppm for ^{13}C). NMR shifts were reported in parts per million (d ppm). Abbreviations for signal multiplicity are as follow: s = singlet, d = doublet, t = triplet, q = quartet, m = multiplet, brs = broad singlet, dd = doublet of doublet, etc. Coupling constants (J values) were calculated directly from the spectra. All reagents were purchased commercially and used without further purification unless otherwise noted. IR spectra were collected on a Nicolet iS10 FT-IR spectrometer (cm^{-1}). Optical rotations were measured on Jasco P-2000 polarimeters and on Rudolph Research Analytical Automatic Polarimeter APIV-1W. Mass spectra were taken on a Thermo Finnigan LTQ-FTMS spectrometer with APCI, ESI or NSI. Racemic standards were generated by performing reactions with Rh_2(R/S-TPPTTL)$_4$, which was generated by dissolving an equimolar mixture of the R and S catalyst in a minimal amount of dichloromethane and concentrating under vacuum. The enantiomeric excess (ee) was determined by High Performance Liquid Chromatography analysis was performed on either Varian Prostar model 410 HPLC, Agilent 1100 Technologies HPLC, Agilent Technologies 1290 Infinity UHPLC instruments, or by Super Critical Fluid Chromatography using Water Acquity UPC^2 SFC system and the data outlined below varies in presentation based on the software used for each system. HPLC/SFC traces are reported based on the racemic retention times. The HPLC instruments used isopropanol/hexane gradient and commercial ChiralPak/ChiralCel columns from Daicel Chemical Industries, notably ChiralPak AD-H (5 µm particle size, 4.6 mm vs. 250 mm), ChiralCel OZ-H (5 µm particle size, 4.6 mm vs. 250 mm), and ChiralCel OD-H (5 µm particle size, 4.6 mm vs. 250 mm), ChiralCel AS-H (5 µm particle size, 4.6 mm vs. 250 mm), ChiralCel OJ-H (5 µm particle size, 4.6 mm vs. 250 mm), and Regis (S,S) Whelk-O1 5/100 Kromasil. The SFC system utilized supercritical fluid CO_2 with cosolvents of either HPLC-grade methanol, or acetonitrile, or ethanol, or isopropanol, or 1:1 MeOH:IPA with 0.2% formic acid, or 1:1 Ethanol:IPA with 0.2% formic acid, or 1:1 Ethanol:ACN with 0.2% formic acid, or 1:1:1 Ethanol:IPA:ACN with 20 mM ammonium formate with SFC columns: Trefoil AMY1 Column (2.5 µm, 3.0 mm X 150 mm), Trefoil CEL1 Column (2.5 µm, 3.0 mm X 150 mm), Trefoil CEL2 Column (2.5 µm, 3.0 mm X 150 mm), Regis (S,S) Whelk-O 1 Kromasil (3.5 µm, 3.0 mm X 150 mm), ChiralPak AD-3 (3.0 µm, 3.0 mm X 150 mm SFC),

ChiralCel OZ-3 (3.0 µm, 3.0 mm X 150 mm), ChiralCel OD-3 (3.0 µm, 3.0 mm X 150 mm SFC), ChiralCel OX-3 (3.0 µm, 3.0 mm X 150 mm SFC); ChiralCel OJ-3 (3.0 µm, 3.0 mm X 150 mm SFC); ChiralPak AS-3 (3.0 µm, 3.0 mm X 150 mm SFC).

2. Catalyst Structures

The following catalysts were used in this study and have been prepared previously.

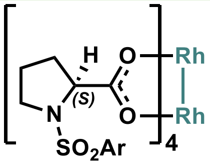<br>**Ar = *p*-($C_{12}H_{25}$)C_6H_4**<br>**Rh_2(*S*-DOSP)$_4$**	**Generation:** Prolinato<br>**Name:** Rh_2(*S*-DOSP)$_4$[2]
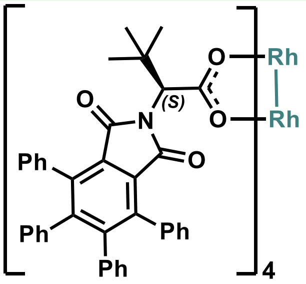<br>**Rh_2(*S*-TPPTTL)$_4$**	**Generation:** Phthalimido<br>**Name:** Rh_2(*S*-TPPTL)$_4$[3]
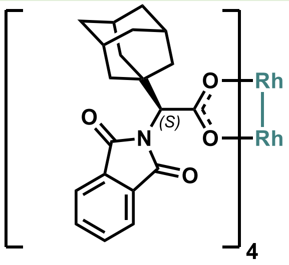<br>**Rh_2(*S*-PTAD)$_4$**	**Generation:** Phthalimido<br>**Name:** Rh_2(*S*-PTAD)$_4$[4]
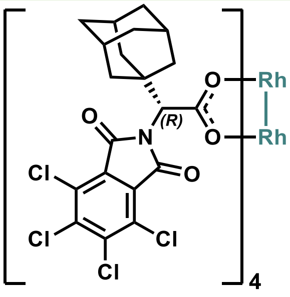<br>**Rh_2(*R*-TCPTAD)$_4$**	**Generation:** Phthalimido<br>**Name:** Rh_2(*R*-TCPTAD)$_4$[4]

<table>
<tr><td>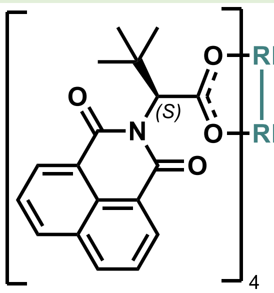

Rh$_2$(*S*-NTTL)$_4$</td><td>Generation: Napthalimido
Name: Rh$_2$(S-NTTL)$_4$[5]</td></tr>
<tr><td>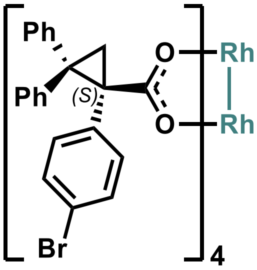

Rh$_2$(*S*-*p*-BrTPCP)$_4$</td><td>Generation: TPCP
Name: Rh$_2$(S-p-BrTPCP)$_4$[6]</td></tr>
<tr><td>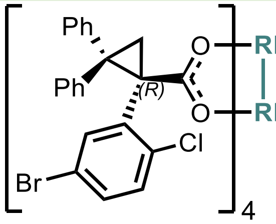

Rh$_2$(*R*-2Cl,5Br-TPCP)$_4$</td><td>Generation: TPCP
Name: Rh$_2$(R-2Cl,5Br-TPCP)$_4$[7]</td></tr>
</table>

Figure S1. Catalyst structures used in catalyst screen.

3. Acquisition of Compounds

Commercially available ketones

These ketones were purchased from commercial sources and used without purification:

1,2-diphenylethan-1-one

2-(4-Bromophenyl)-1-phenylethanone

2-(4-fluorophenyl)-1-phenylethanone

Substrates and reagents

These reagents were purchased from commercial sources and used without further purification. If a liquid, the material was dried with 4 Å MS over 24 hour and sparged with nitrogen for at least 10 minutes before use:

4-ethyl toluene

4-ethylphenyl acetate

1-ethyl-4-methoxybenzene

2,3-dihydrobenzofuran

5-methoxyindane

Cyclohexane

Cyclopentane

Trans-2-hexene

Tetrahydrofuran

Tetrahydropyran

Adamantane

The following compounds were prepared according to published procedures:

o-NBSA[1]

(E)-tert-butyldimethyl(pent-2-en-1-yloxy)silane[8]

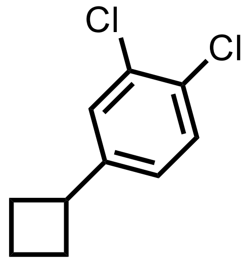

1,2-dichloro-4-cyclobutylbenzene (S1)

To a flame-dried round-bottom flask with findenser was charged with a stir-bar and oven-dried silver Mg turnings (646 mg, 1.5 equiv, 26.56 mmol). The round-bottom flask was sealed, flame-dried again, and backfilled with N_2 three times. To the reaction vessel was added dry THF (1 molar) and was allowed to stir for over 30 minutes under N_2 line. Afterwards, 4-bromo-1,2-dichlorobenzene (4.0 g, 1 equiv, 17.71 mmol) was added drop-wise over 10 min (can be very exothermic after complete addition). The Grignard was stirred for 1 h then cooled to 0 °C via ice bath. Then cyclobutanone (800 mg, 1 equiv, 11.41 mmol) was added and the reaction was warmed to room temperature overnight. The crude mixture was quenched with saturated ammonium chloride solution, extracted with ether two times, then dried with $MgSO_4$, filtered, and the filtrate concentrated *in vacuo*. The crude material was carried further without further purification.

^{1}H NMR (400 MHz, $CDCl_3$) δ 7.57 (d, *J* = 2.2 Hz, 1H), 7.42 (d, *J* = 8.3 Hz, 1H), 7.32 (dd, *J* = 8.3, 2.2 Hz, 1H), 2.52 – 2.45 (m, 2H), 2.39 – 2.29 (m, 2H), 2.09 – 1.98 (m, 1H), 1.71 (dtt, *J* = 11.5, 8.9, 7.5 Hz, 1H).

To a flame-dried 50 mL round-bottom charged with a stir-bar was backfilled with nitrogen three times and then was added 1-(3,4-dichlorophenyl)cyclobutan-1-ol (1.0 g, 1 equiv, 4.6 mmol) and TFA (7.1 ml, 20 equiv, 92 mmol) and then cooled to 0 °C in an ice bath for 10 min. At 0 °C, triethylsilane (3.7 ml, 5 equiv, 23 mmol) was added dropwise and then the mixture was warmed to room temperature and stirred overnight. The reaction was cooled to 0 °C and quenched with saturated sodium bicarbonate, extracted with CH_2Cl_2,

then dried with $MgSO_4$, filtered, and the filtrate concentrated *in vacuo*. The crude material was purified by flash chromatography (isocratic hexanes) to yield a pink oil. The residue was then subjected to Kugelrohr distillation (0.5 mmHg, 100 ºC) for 20 min to remove excess silane resulting in the clean product as a clear oil (442 mg, 48% yield).

Rf = 0.76 (hexanes)

^{1}H NMR (600 MHz, $CDCl_3$) δ 7.34 (d, *J* = 8.2 Hz, 1H), 7.28 (d, *J* = 2.1 Hz, 1H), 7.03 (dd, *J* = 8.2, 2.1 Hz, 1H), 3.49 (p, *J* = 8.6 Hz, 1H), 2.34 (qt, *J* = 7.6, 2.2 Hz, 2H), 2.15 – 1.95 (m, 3H), 1.90 – 1.81 (m, 1H).

^{13}C NMR (151 MHz, $CDCl_3$) δ 146.5, 132.0, 130.0, 129.3, 128.3, 125.8, 39.3, 29.5, 18.1.

IR (neat) 2963, 2940, 2891, 2861, 1590, 1557, 1472, 1443, 1396, 1378, 1331, 1244, 1131, 1096, 1028, 920, 874, 816, 787, 709, 672, 590 cm^{-1}.

HRMS (+p APCI) calc. mass for $C_{10}H_{10}Cl_2$ $[M+]^+$ - 200.0160; obs. mass for $C_{10}H_{10}Cl_2$ $[M+]^+$ - 200.0158.

4. Experimental Procedure

A. Preparation of Weinreb Amides

General Procedure A for the Synthesis of Weinreb Amides

To a vessel charged with a stir-bar was added the corresponding acetic acid (1 equiv), N,O-dimethylhydroxylamine hydrochloride (1.5 equiv), N-(3- Dimethylaminopropyl)-N'-ethylcarbodiimide hydrochloride (1.5 equiv), and N,N-dimethylpyridin-4-amine (1.5 equiv). To the solid mixture was added CH_2Cl_2 (0.250 molar). The reaction mixture was allowed to stir overnight at room temperature. Afterwards, the organic layer was washed with 1M HCl and then the organic layer was dried with $MgSO_4$, filtered, and the filtrate concentrated *in vacuo* to afford the corresponding Weinreb amide without further purification and generally clean by ^{1}H NMR. The weinreb amides were moved forwards to the next step without further characterization.

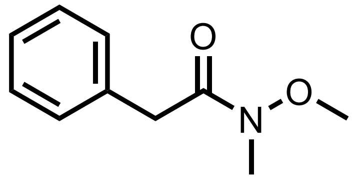

***N*-methoxy-*N*-methyl-2-phenylacetamide (S2)**

Prepared from general procedure A. To a 1 L round-bottom flask with stir-bar was charged with the 2-phenylacetic acid (1 equiv, 734 mmol), N,O-dimethylhydroxylamine hydrochloride (1.5 equiv, 1.10 mol), N-(3-Dimethylaminopropyl)-N'-ethylcarbodiimide hydrochloride (1.5 equiv, 1.10 mol), and N,N-dimethylpyridin-4-amine (1.5 equiv, 1.10 mol). To the solid mixture was added CH_2Cl_2 (0.250 molar). The corresponding compound is a transparent light-yellow oil (110 g, 614 mmol, 83% yield). The material was moved forwards to the next step without further characterization. The ^{1}H NMR spectrum is in good agreement with the corresponding literature precedent.[9]

^{1}H NMR (400 MHz, $CDCl_3$) δ 7.34 – 7.27 (m, 4H), 7.27 – 7.20 (m, 1H), 3.77 (s, 2H), 3.59 (s, 3H), 3.18 (s, 3H).

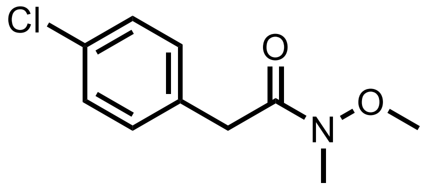

2-(4-chlorophenyl)-*N*-methoxy-*N*-methylacetamide (S3)

Prepared from general procedure A. To a 55 mL test-tube with stir-bar was charged with the 2-(4-chlorophenyl)acetic acid (1 equiv, 5.00 mmol), N,O-dimethylhydroxylamine hydrochloride (1.5 equiv, 7.50 mmol), N-(3-Dimethylaminopropyl)-N'-ethylcarbodiimide hydrochloride (1.5 equiv, 7.50 mmol), and N,N-dimethylpyridin-4-amine (1.5 equiv, 7.50 mmol). To the solid mixture was added CH_2Cl_2 (0.250 molar). The corresponding compound is a clear crystalline solid (0.982 g, 4.60 mmol, 92% yield). The material was moved

forwards to the next step without further characterization. The ^{1}H NMR spectrum is in good agreement with the corresponding literature precedent.[10]

^{1}H NMR (400 MHz, $CDCl_3$) δ 7.32 – 7.18 (m, 4H), 3.74 (s, 2H), 3.63 (s, 3H), 3.19 (s, 3H).

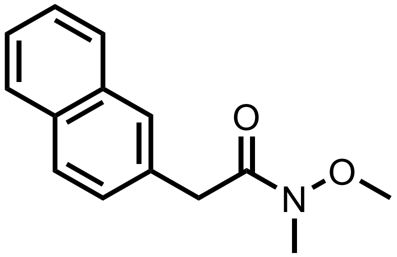

***N*-methoxy-*N*-methyl-2-(naphthalen-2-yl)acetamide (S4)**

Prepared from general procedure A. To a 55 mL test-tube with stir-bar was charged with 2-(naphthalen-2-yl)acetic acid (1 equiv., 5.00 mmol), N,O-dimethylhydroxylamine hydrochloride (1.5 equiv., 7.50 mmol), N-(3-Dimethylaminopropyl)-N'-ethylcarbodiimide hydrochloride (1.5 equiv., 7.50 mmol), and N,N-dimethylpyridin-4-amine (1.5 equiv., 7.50 mmol). To the solid mixture was added CH_2Cl_2 (0.250 molar). The corresponding compound is a clear light-yellow oil (1.08 g, 4.71 mmol, 94% yield). The material was moved forwards to the next step without further characterization. The ^{1}H NMR spectrum is in good agreement with the corresponding literature precedent.[9]

^{1}H NMR (400 MHz, $CDCl_3$) δ 7.80 (dt, *J* = 8.6, 3.3 Hz, 3H), 7.74 (s, 1H), 7.50 – 7.39 (m, 3H), 3.94 (s, 2H), 3.61 (s, 3H), 3.22 (s, 3H).

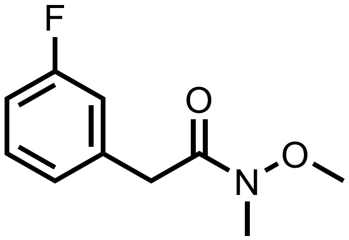

2-(3-fluorophenyl)-*N*-methoxy-*N*-methylacetamide (S5)

Prepared from general procedure A. To a 55 mL test-tube with stir-bar was charged with 2-(3-(fluorophenyl)acetic acid (1 equiv, 5.00 mmol), N,O-dimethylhydroxylamine hydrochloride (1.5 equiv, 7.50 mmol), N-(3-Dimethylaminopropyl)-N'-ethylcarbodiimide hydrochloride (1.5 equiv, 7.50 mmol), and N,N-dimethylpyridin-4-amine (1.5 equiv, 7.50 mmol). To the solid mixture was added CH_2Cl_2 (0.250 molar). The corresponding compound is a clear yellow oil (0.927 g, 4.70 mmol, 94% yield). The material was moved forwards to the next step without further characterization. The ^{1}H NMR spectrum is in good agreement with the corresponding literature precedent.[11]

^{1}H NMR (400 MHz, $CDCl_3$) δ 7.27 (td, *J* = 8.0, 5.9 Hz, 1H), 7.08 – 7.00 (m, 2H), 6.94 (tdd, *J* = 8.5, 2.7, 1.0 Hz, 1H), 3.76 (s, 2H), 3.63 (s, 3H), 3.20 (s, 3H).

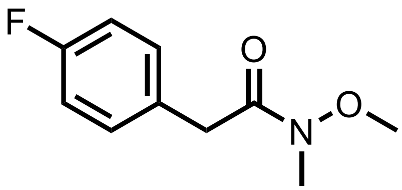

2-(4-fluorophenyl)-*N*-methoxy-*N*-methylacetamide (S6)

Prepared from general procedure A. To a 55 mL test-tube with stir-bar was charged with 2-(4-fluorophenyl)acetic acid (1 equiv, 5.00 mmol), N,O-dimethylhydroxylamine hydrochloride (1.5 equiv, 7.50 mmol), N-(3-Dimethylaminopropyl)-N'-ethylcarbodiimide hydrochloride (1.5 equiv, 7.50 mmol), and N,N-dimethylpyridin-4-amine (1.5 equiv, 7.50 mmol). To the solid mixture was added CH_2Cl_2 (0.250 molar). The corresponding compound is a clear oil (0.797 g, 4.04 mmol, 81% yield). The material was moved forwards to the next step without further characterization. The ^{1}H NMR spectrum is in good agreement with the corresponding literature precedent.[12]

^{1}H NMR (400 MHz, $CDCl_3$) δ 7.30 – 7.20 (m, 2H), 7.05 – 6.95 (m, 2H), 3.74 (s, 2H), 3.63 (s, 3H), 3.19 (s, 3H).

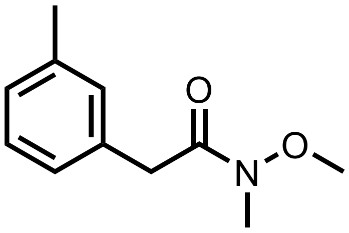

***N*-methoxy-*N*-methyl-2-(*m*-tolyl)acetamide (S7)**

Prepared from general procedure A. To a 55 mL test-tube with stir-bar was charged with 2-(m-tolyl)acetic acid (1 equiv, 5.00 mmol), N,O-dimethylhydroxylamine hydrochloride (1.5 equiv, 7.50 mmol), N-(3-Dimethylaminopropyl)-N'-ethylcarbodiimide hydrochloride (1.5 equiv, 7.50 mmol), and N,N-dimethylpyridin-4-amine (1.5 equiv, 7.50 mmol). To the solid mixture was added CH_2Cl_2 (0.250 molar). The corresponding compound is a clear oil (0.926 g, 4.79 mmol, 96% yield). The material was moved forwards to the next step without further characterization. The ^{1}H NMR spectrum is in good agreement with the corresponding literature precedent.[9]

^{1}H NMR (400 MHz, $CDCl_3$) δ 7.20 (t, *J* = 7.5 Hz, 1H), 7.13 – 7.04 (m, 3H), 3.74 (s, 2H), 3.61 (s, 3H), 3.19 (s, 3H), 2.33 (s, 3H).

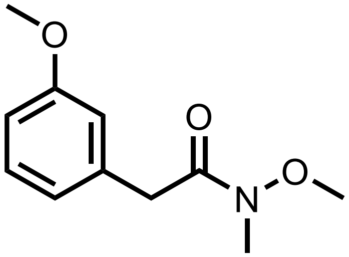

***N*-methoxy-2-(3-methoxyphenyl)-*N*-methylacetamide (S8)**

Prepared from general procedure A. To a 55 mL test-tube with stir-bar was charged with 2-(3-methoxyphenyl)acetic acid (1 equiv, 5.00 mmol), N,O-dimethylhydroxylamine hydrochloride (1.5 equiv, 7.50 mmol), N-(3-Dimethylaminopropyl)-N'-ethylcarbodiimide hydrochloride (1.5 equiv, 7.50 mmol), and N,N-

dimethylpyridin-4-amine (1.5 equiv, 7.50 mmol). To the solid mixture was added CH_2Cl_2 (0.250 molar). The corresponding compound is a clear light-yellow oil (0.899 g, 4.29 mmol, 86% yield). The material was moved forwards to the next step without further characterization. The ^{1}H NMR spectrum is in good agreement with the corresponding literature precedent.[13]

^{1}H NMR (400 MHz, $CDCl_3$) δ 7.22 (dd, *J* = 8.2, 7.5 Hz, 1H), 6.90 – 6.85 (m, 2H), 6.79 (ddd, *J* = 8.2, 2.6, 1.0 Hz, 1H), 3.80 (s, 3H), 3.75 (s, 2H), 3.61 (s, 3H), 3.19 (s, 3H).

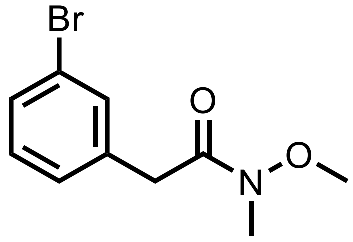

2-(3-bromophenyl)-*N*-methoxy-*N*-methylacetamide (S9)

Prepared from general procedure A. To a 55 mL test-tube with stir-bar was charged with 2-(3-bromophenyl)acetic acid (1 equiv, 5.00 mmol), N,O-dimethylhydroxylamine hydrochloride (1.5 equiv, 7.50 mmol), N-(3-Dimethylaminopropyl)-N'-ethylcarbodiimide hydrochloride (1.5 equiv, 7.50 mmol), and N,N-dimethylpyridin-4-amine (1.5 equiv, 7.50 mmol). To the solid mixture was added CH_2Cl_2 (0.250 molar). The corresponding compound is a transparent white amorphous material (1.18 g, 4.57 mmol, 91% yield). The material was moved forwards to the next step without further characterization. The ^{1}H NMR spectrum is in good agreement with the corresponding literature precedent.[14]

^{1}H NMR (400 MHz, $CDCl_3$) δ 7.45 (t, *J* = 1.9 Hz, 1H), 7.38 (dt, *J* = 7.6, 1.7 Hz, 1H), 7.25 – 7.15 (m, 2H), 3.74 (s, 2H), 3.64 (s, 3H), 3.20 (s, 3H).

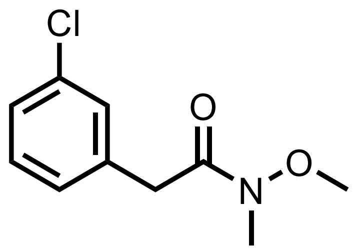

2-(3-chlorophenyl)-*N*-methoxy-*N*-methylacetamide (S10)

Prepared from general procedure A. To a 55 mL test-tube with stir-bar was charged with 2-(3-chlorophenyl)acetic acid (1 equiv, 5.00 mmol), N,O-dimethylhydroxylamine hydrochloride (1.5 equiv, 7.50 mmol), N-(3-Dimethylaminopropyl)-N'-ethylcarbodiimide hydrochloride (1.5 equiv, 7.50 mmol), and N,N-dimethylpyridin-4-amine (1.5 equiv, 7.50 mmol). To the solid mixture was added CH_2Cl_2 (0.250 molar). The corresponding compound is a clear yellow oil (0.933 g, 4.37 mmol, 87% yield). The material was moved forwards to the next step without further characterization. The ^{1}H NMR spectrum is in good agreement with the corresponding literature precedent.[15]

^{1}H NMR (400 MHz, CDCl$_3$) δ 7.29 (td, *J* = 1.7, 0.8 Hz, 1H), 7.25 – 7.22 (m, 2H), 7.18 (ddd, *J* = 6.6, 2.3, 1.6 Hz, 1H), 3.74 (s, 2H), 3.64 (s, 3H), 3.20 (s, 3H).

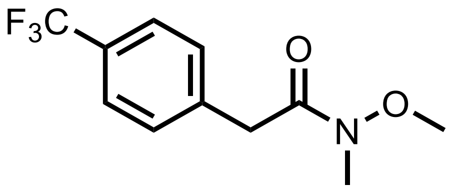

***N*-methoxy-*N*-methyl-2-(4-(trifluoromethyl)phenyl)acetamide (S11)**

Prepared from general procedure A. To a 55 mL test-tube with stir-bar was charged with 2-(4-(trifluoromethyl)phenyl)acetic acid (1 equiv, 5.00 mmol), N,O- dimethylhydroxylamine hydrochloride (1.5 equiv, 7.50 mmol), N-(3- Dimethylaminopropyl)-N'-ethylcarbodiimide hydrochloride (1.5 equiv, 7.50 mmol), and N,N-dimethylpyridin-4-amine (1.5 equiv, 7.50 mmol). To the solid mixture was added CH_2Cl_2 (0.250

molar). The corresponding compound is a clear crystalline solid (1.14 g, 4.61 mmol, 92% yield). The material was moved forwards to the next step without further characterization. The ^{1}H NMR spectrum is in good agreement with the corresponding literature precedent.[16]

^{1}H NMR (400 MHz, $CDCl_3$) δ 7.58 (d, *J* = 7.9 Hz, 2H), 7.41 (d, *J* = 7.8 Hz, 2H), 3.83 (s, 2H), 3.66 (s, 3H), 3.21 (s, 3H).

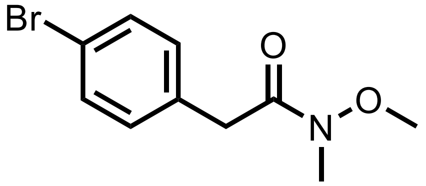

2-(4-bromophenyl)-*N*-methoxy-*N*-methylacetamide (S12)

Prepared from general procedure A. To a 55 mL test-tube with stir-bar was charged with 2-(4-bromophenyl)acetic acid (1 equiv, 5.00 mmol), N,O-dimethylhydroxylamine hydrochloride (1.5 equiv, 7.50 mmol), N-(3-Dimethylaminopropyl)-N'-ethylcarbodiimide hydrochloride (1.5 equiv, 7.50 mmol), and N,N-dimethylpyridin-4-amine (1.5 equiv, 7.50 mmol). To the solid mixture was added CH_2Cl_2 (0.250 molar). The corresponding compound is a white solid (1.13 g, 4.38 mmol, 88% yield). The material was moved forwards to the next step without further characterization. The ^{1}H NMR spectrum is in good agreement with the corresponding literature precedent.[9]

^{1}H NMR (400 MHz, $CDCl_3$) δ 7.44 (d, *J* = 8.4 Hz, 2H), 7.17 (d, *J* = 8.4 Hz, 2H), 3.72 (s, 2H), 3.63 (s, 3H), 3.19 (s, 3H).

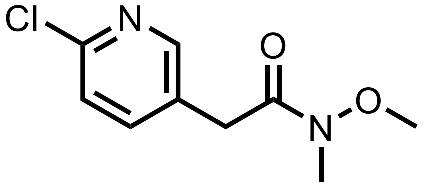

2-(6-chloropyridin-3-yl)-*N*-methoxy-*N*-methylacetamide (S13)

Prepared from general procedure A. To a 500 mL round-bottom flask with stir-bar was charged with 2-(6-chloropyridin-3-yl)acetic acid (1 equiv, 75.00 mmol), N,O- dimethylhydroxylamine hydrochloride (1.5 equiv, 112.5 mmol), N-(3- Dimethylaminopropyl)-N'-ethylcarbodiimide hydrochloride (1.5 equiv, 112.5 mmol), and N,N-dimethylpyridin-4-amine (1.5 equiv, 112.5 mmol). To the solid mixture was added CH_2Cl_2 (0.250 molar). The corresponding compound is a clear light-yellow oil (0.899 g, 4.29 mmol, 86% yield). The material was moved forwards to the next step without further characterization. The ^{1}H NMR spectrum is in good agreement with the corresponding literature precedent.[17]

^{1}H NMR (400 MHz, $CDCl_3$) δ 8.26 (d, *J* = 2.4 Hz, 1H), 7.62 (dd, *J* = 8.2, 2.3 Hz, 1H), 7.31 – 7.24 (m, 1H), 3.74 (s, 2H), 3.69 (d, *J* = 2.6 Hz, 3H), 3.19 (s, 3H).

B. Preparation of Ketones

Procedure to Wash Magnesium Turnings:

1. Add magnesium turnings to a large-fritted glass funnel.
2. Pour 1-3 M HCl onto magnesium turnings. This will effervesce the magnesium

exposing a fresh Magnesium surface. **WARNING**: **this is an exothermic reaction**. Pour slowly and triturate the turnings so that the solution saturates the turnings. This step usually requires excess HCl solution until every single turning has a fresh silvery Mg surface.

3. After washing the Mg, rinse the magnesium with excess ethanol to wash off any remaining aqueous solution while triturating.
4. Dry magnesium turnings by rinsing with excess diethyl ether while triturating.
5. After drying the silver Mg turnings with the ether, the silver Mg turnings were stored in an oven at 130 ^{o}C.

Preparation of 1 M Grignard Solutions:

To a flame-dried round-bottom flask with findenser charged with a stir-bar was added the oven-dried silver Mg turnings (1.1 equiv). The round-bottom flask was sealed and backfilled with N_2 three times. To the reaction vessel was added dry THF (1 molar) and was allowed to stir for over 30 minutes under N_2 line. Afterwards, the desired halide (1 equiv) was added drop-wise for over 30 minutes [can be very exothermic after complete addition]. The solution was then placed in a sonicator and sonicated under N_2 balloon for over 3 hours. The desired 1 M Grignard solution was then subjected to the Grignard addition.

General Procedure B for the Synthesis of Ketones

To a flame-dried round-bottom flask charged with a stir-bar was added the corresponding Weinreb amide (1 equiv), sealed and backfilled with N_2 three times, and subsequently, dry THF (0.15 molar) was added to the sealed-vessel under N_2 balloon. The reaction mixture was cooled to 0 ^{o}C with an ice bath and allowed to stir for over 10 minutes. Subsequently to the cooled solution was added the corresponding 1M Grignard reagent (1.5 equiv). The reaction mixture was allowed to stir overnight warming up to room temperature. After the elapsed time, to the reaction mixture was quenched with excess saturated NH_4Cl solution, diluted with ethyl acetate, extracted with ethyl acetate three times, the combined organic layers were dried with $MgSO_4$, filtered, and the filtrate concentrated *in vacuo*. The crude material was subjected to flash chromatography hexanes/ethyl acetate. The collected fractions were concentrated *in vacuo* to afford the corresponding ketone.

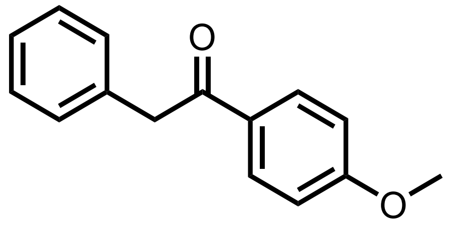

1-(4-methoxyphenyl)-2-phenylethan-1-one (S14)

1 M Grignard solution prepared from Preparation of 1 M Grignard Solutions. Magnesium (1.6 g, 2.5 equiv, 67 mmol) and 1-bromo-4-methoxybenzene (5.0 g, 3.3 mL, 1 equiv, 27 mmol) was used. The resultant Grignard solution is an opaque gray solution.

Ketone prepared from General Procedure B. N-methoxy-N-methyl-2-phenylacetamide (1.0 g, 1 equiv, 5.6 mmol) was used. The crude material was dry-loaded onto silica and subjected to flash chromatography hexanes/EtOAc. The collected fractions were concentrated *in vacuo* to afford 1-(4-methoxyphenyl)-2-phenylethan-1-one tinged with yellow amorphous material and white crystals. The material was dissolved in minimal hexanes and allowed to recrystallize in -20 °C freezer in a sealed vial, filtered, and the solids collected and dried to afford 1-(4-methoxyphenyl)-2-phenylethan-1-one (860 mg, 3.80 mmol, 68% yield) as white solids. The ^{1}H NMR spectrum is in good agreement with the corresponding literature precedent.[18]

^{1}H NMR (600 MHz, $CDCl_3$) δ 8.02 – 7.97 (m, 2H), 7.34 – 7.22 (m, 5H), 6.95 – 6.90 (m, 2H), 4.23 (s, 2H), 3.86 (s, 3H).

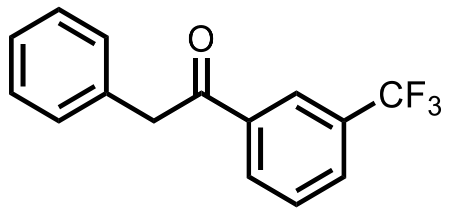

2-phenyl-1-(3-(trifluoromethyl)phenyl)ethan-1-one (S15)

1 M Grignard solution prepared from Preparation of 1 M Grignard Solutions. Magnesium (0.8 g, 1.5 equiv, 0.03 mol) and 1-bromo-3-(trifluoromethyl)benzene (5 g, 3 mL, 1 equiv, 0.02 mol) was used. The resultant Grignard solution is a dark brown solution.

Ketone prepared from General Procedure B. N-methoxy-N-methyl-2-phenylacetamide (2 g, 2 mL, 1 equiv, 0.01 mol) was used. The crude material was dry-loaded onto silica and subjected to flash chromatography hexanes/EtOAc. The collected fractions were concentrated *in vacuo* to afford 2-phenyl-1-(3-

(trifluoromethyl)phenyl)ethan-1-one (2.343 g, 8.867 mmol, 80% yield) as a yellow oil. The ^{1}H NMR spectrum is in good agreement with the corresponding literature precedent.[19]

^{1}H NMR (400 MHz, CDCl$_3$) δ 8.27 (tt, J = 1.8, 0.9 Hz, 1H), 8.18 (dt, J = 8.0, 1.5 Hz, 1H), 7.85 – 7.77 (m, 1H), 7.67 – 7.56 (m, 1H), 7.40 – 7.23 (m, 4H), 4.32 (s, 2H).

^{19}F NMR (376 MHz, CDCl$_3$) δ -62.8.

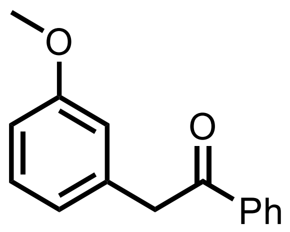

2-(3-methoxyphenyl)-1-phenylethan-1-one (S16)

Ketone prepared from General Procedure B. Phenylmagnesium bromide (4.080 g, 22.50 mL, 1 molar, 1.25 equiv, 22.50 mmol) and N-methoxy-2-(3-methoxyphenyl)-N- methylacetamide (3.767 g, 1 equiv, 18.00 mmol) was used. The crude material was dry-loaded onto silica and subjected to flash chromatography hexanes/EtOAc. The collected fractions were concentrated *in vacuo* to afford 2-(3-methoxyphenyl)-1-phenylethan-1- one (2.4995 g, 11.046 mmol, 61% yield) as a clear yellow oil. The ^{1}H NMR spectrum is in good agreement with the corresponding literature precedent.[20]

^{1}H NMR (400 MHz, CDCl$_3$) δ 8.05 – 7.98 (m, 2H), 7.60 – 7.51 (m, 1H), 7.50 – 7.41 (m, 2H), 7.27 – 7.19 (m, 1H), 6.90 – 6.76 (m, 3H), 4.26 (s, 2H), 3.79 (s, 3H).

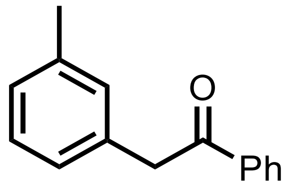

1-phenyl-2-(*m*-tolyl)ethan-1-one (S17)

Ketone prepared from General Procedure B. Phenylmagnesium bromide (4.080 g, 22.50 mL, 1 molar, 1.25 equiv, 22.50 mmol) and N-methoxy-N-methyl-2-(m-tolyl)acetamide (3.479 g, 1 equiv, 18.00 mmol) was used. The crude material was dry-loaded onto silica and subjected to flash chromatography hexanes/EtOAc. The collected fractions were concentrated *in vacuo* to afford 1-phenyl-2-(m-tolyl)ethan-1-one (2.17 g, 10.3 mmol, 57% yield) as a clear yellow oil. The ^{1}H NMR spectrum is in good agreement with the corresponding literature precedent.[21]

^{1}H NMR (400 MHz, $CDCl_3$) δ 8.06 – 7.99 (m, 2H), 7.60 – 7.51 (m, 1H), 7.51 – 7.42 (m, 2H), 7.22 (t, J = 7.5 Hz, 1H), 7.12 – 7.04 (m, 3H), 4.25 (s, 2H), 2.33 (d, J = 0.7 Hz, 3H).

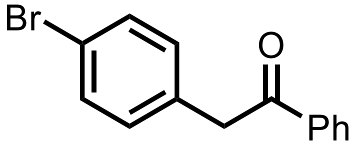

2-(4-bromophenyl)-1-phenylethan-1-one (S18)

Ketone prepared from General Procedure B. Phenylmagnesium bromide (4.080 g, 22.50 mL, 1 molar, 1.25 equiv, 22.50 mmol) and 2-(4-bromophenyl)-N-methoxy-N- methylacetamide (4.646 g, 1 equiv, 18.00 mmol) was used. The crude material was dry- loaded onto silica and subjected to flash chromatography hexanes/EtOAc. The collected fractions were concentrated *in vacuo* to afford 2-(4-bromophenyl)-1-phenylethan-1-one (1.656 g, 6.019 mmol, 33% yield) as a white powder. The ^{1}H NMR spectrum is in good agreement with the corresponding literature precedent.[22]

^{1}H NMR (400 MHz, $CDCl_3$) δ 8.04 – 7.96 (m, 2H), 7.62 – 7.53 (m, 1H), 7.52 – 7.43 (m, 4H), 7.40 – 7.32 (m, 1H), 7.16 – 7.10 (m, 2H), 4.25 (s, 2H).

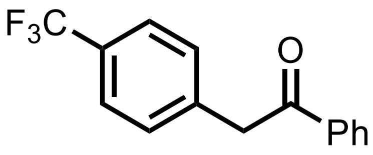

1-phenyl-2-(4-(trifluoromethyl)phenyl)ethan-1-one (S19)

Ketone prepared from General Procedure B. Phenylmagnesium bromide (4.080 g, 22.50 mL, 1 molar, 1.25 equiv, 22.50 mmol) and N-methoxy-N-methyl-2-(4- (trifluoromethyl) -phenyl)acetamide (4.450 g, 1 equiv, 18.00 mmol) was used. The crude material was dry-loaded onto silica and subjected to flash chromatography hexanes/EtOAc. The collected fractions were concentrated *in vacuo* to afford 1-phenyl-2- (4-(trifluoromethyl)phenyl)ethan-1-one (1.155 g, 4.371 mmol, 24% yield) as a white powder. The ^{1}H NMR spectrum is in good agreement with the corresponding literature precedent.[23]

^{1}H NMR (400 MHz, $CDCl_3$) δ 8.06 – 7.98 (m, 2H), 7.64 – 7.55 (m, 3H), 7.54 – 7.40 (m, 2H), 7.44 – 7.35 (m, 2H), 4.36 (s, 2H).

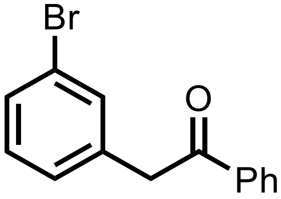

2-(3-bromophenyl)-1-phenylethan-1-one (S20)

Ketone prepared from General Procedure B. Phenylmagnesium bromide (4.080 g, 22.50 mL, 1 molar, 1.25 equiv, 22.50 mmol) and 2-(3-bromophenyl)-N-methoxy-N- methylacetamide (4.646 g, 1 equiv, 18.00 mmol) was used. The crude material was dry-loaded onto silica and subjected to flash chromatography hexanes/EtOAc. The collected fractions were concentrated *in vacuo* to afford 2-(3-bromophenyl)-1-phenylethan-1-one (2.16 g, 7.85 mmol, 44% yield) as a clear yellow oil. The ^{1}H NMR spectrum is in good agreement with the corresponding literature precedent.[24]

^{1}H NMR (400 MHz, CDCl$_3$) δ 8.04 – 7.97 (m, 2H), 7.63 – 7.56 (m, 1H), 7.51 – 7.45 (m, 2H), 7.44 – 7.37 (m, 2H), 7.28 – 7.15 (m, 2H), 4.26 (s, 2H).

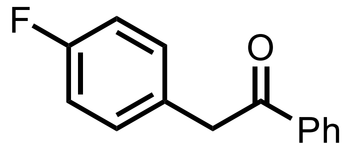

2-(4-fluorophenyl)-1-phenylethan-1-one (S21)

Ketone prepared from General Procedure B. Phenylmagnesium bromide (4.080 g, 22.50 mL, 1 molar, 1.25 equiv, 22.50 mmol) and 2-(4-fluorophenyl)-N-methoxy-N- methylacetamide (3.550 g, 1 equiv, 18.00 mmol) was used. The crude material was dry-loaded onto silica and subjected to flash chromatography hexanes/EtOAc. The collected fractions were concentrated *in vacuo* to afford 2-(4-fluorophenyl)-1-phenylethan-1-one (1.76 g, 8.22 mmol, 46% yield) as a white solid. The ^{1}H NMR spectrum is in good agreement with the corresponding literature precedent.[21]

^{1}H NMR (400 MHz, CDCl$_3$) δ 8.05 – 7.97 (m, 2H), 7.62 – 7.53 (m, 1H), 7.52 – 7.43 (m, 2H), 7.27 – 7.18 (m, 2H), 7.07 – 6.97 (m, 2H), 4.27 (d, J = 0.7 Hz, 2H).

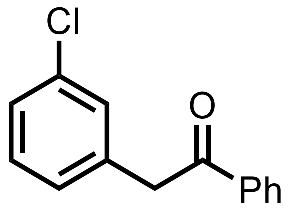

2-(3-chlorophenyl)-1-phenylethan-1-one (S22)

Ketone prepared from General Procedure B. Phenylmagnesium bromide (1.59 g, 8.75 mL, 1 molar, 1.25 equiv, 8.75 mmol) and 2-(3-chlorophenyl)-N-methoxy-N-methylacetamide (1.50 g, 1 equiv, 7.00 mmol) was used. The crude material was dry-loaded onto silica and subjected to flash chromatography hexanes/EtOAc. The collected fractions were concentrated *in vacuo* to afford 2-(3-chlorophenyl)-1-

phenylethan-1-one (422 mg, 1.83 mmol, 26% yield) as a clear yellow oil. The ^{1}H NMR spectrum is in good agreement with the corresponding literature precedent.[22]

^{1}H NMR (400 MHz, $CDCl_3$) δ 8.04 – 7.97 (m, 2H), 7.64 – 7.53 (m, 1H), 7.53 – 7.42 (m, 2H), 7.29 – 7.22 (m, 3H), 7.15 (ddd, J = 6.7, 3.1, 1.7 Hz, 1H), 4.27 (s, 2H).

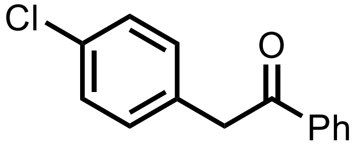

2-(4-chlorophenyl)-1-phenylethan-1-one (S23)

Ketone prepared from General Procedure B. Phenylmagnesium bromide (3.590 g, 19.80 mL, 1 molar, 1.1 equiv, 19.80 mmol) and 2-(4-chlorophenyl)-N-methoxy-N-methylacetamide (3.846 g, 1 equiv, 18.00 mmol) was used. The crude material was dry-loaded onto silica and subjected to flash chromatography hexanes/EtOAc. The collected fractions were concentrated *in vacuo* to afford 2-(4-chlorophenyl)-1-phenylethan-1-one (1.637 g, 7.096 mmol, 39% yield) as a white powder. The ^{1}H NMR spectrum is in good agreement with the corresponding literature precedent.[21]

^{1}H NMR (600 MHz, $CDCl_3$) δ 8.04 – 7.96 (m, 2H), 7.58 (ddt, J = 8.7, 7.0, 1.3 Hz, 1H), 7.53 – 7.45 (m, 2H), 7.32 – 7.28 (m, 2H), 7.23 – 7.17 (m, 2H).

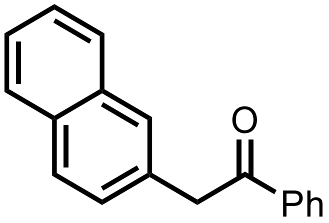

2-(naphthalen-2-yl)-1-phenylethan-1-one (S24)

Ketone prepared from General Procedure B. Phenylmagnesium bromide (4.080 g, 22.50 mL, 1 molar, 1.25 equiv, 22.50 mmol) and N-methoxy-N-methyl-2-(naphthalen-2-yl)acetamide (4.127 g, 1 equiv, 18.00 mmol) was used. The crude material was dry-loaded onto silica and subjected to flash chromatography hexanes/EtOAc. The collected fractions were concentrated *in vacuo* to afford 2-(naphthalen-2-yl)-1-phenylethan-1-one (3.2 g, 13 mmol, 72% yield) as a white powder. The ^{1}H NMR spectrum is in good agreement with the corresponding literature precedent.[22]

^{1}H NMR (600 MHz, $CDCl_3$) δ 8.08 – 8.03 (m, 1H), 7.84 – 7.76 (m, 3H), 7.73 (dd, J = 1.6, 0.8 Hz, 1H), 7.59 – 7.53 (m, 1H), 7.49 – 7.43 (m, 4H), 7.40 (dd, J = 8.4, 1.8 Hz, 1H), 7.26 (s, 1H), 4.46 (s, 2H).

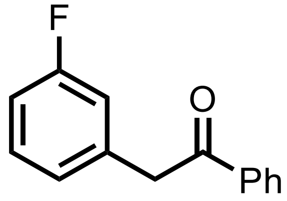

2-(3-fluorophenyl)-1-phenylethan-1-one (S25)

Ketone prepared from General Procedure B. Phenylmagnesium bromide (4.080 g, 22.50 mL, 1 molar, 1.25 equiv, 22.50 mmol) and 2-(3-fluorophenyl)-N-methoxy-N- methylacetamide (3.550 g, 1 equiv, 18.00 mmol) was used. The crude material was dry-loaded onto silica and subjected to flash chromatography hexanes/EtOAc. The collected fractions were concentrated *in vacuo* to afford 2-(3-fluorophenyl)-1-phenylethan-1-one (617.4 mg, 2.882 mmol, 16% yield) as a yellow oil. The ^{1}H NMR spectrum is in good agreement with the corresponding literature precedent.[24]

^{1}H NMR (600 MHz, $CDCl_3$) δ 8.01 (dd, J = 8.3, 1.3 Hz, 2H), 7.58 (ddt, J = 7.8, 7.0, 1.3 Hz, 1H), 7.48 (dd, J = 8.2, 7.3 Hz, 2H), 7.29 (td, J = 8.0, 6.0 Hz, 1H), 7.04 (ddd, J = 7.6, 1.6, 0.9 Hz, 1H), 7.01 – 6.94 (m, 2H), 4.29 (s, 2H).

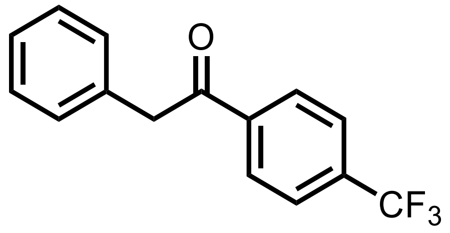

2-phenyl-1-(4-(trifluoromethyl)phenyl)ethan-1-one (S26)

1 M Grignard solution prepared from Preparation of 1 M Grignard Solutions. Magnesium (19.0 g, 1.1 equiv, 782 mmol) and 1-bromo-4-(trifluoromethyl)benzene (160 g, 98.6 mL, 1 equiv, 711 mmol) was used. The resultant Grignard solution is a dark brown solution.

Ketone prepared from General Procedure B. N-methoxy-N-methyl-2-phenylacetamide (10 g, 1 equiv, 44 mmol) was used. The crude material was dry-loaded onto silica and subjected to flash chromatography Hexanes/EtOAc. The collected fractions were concentrated *in vacuo*. The amorphous orange material was resuspended in minimal amount of ethanol and filtered. The white solids were dried to afford 2-phenyl-1-(4-(trifluoromethyl)phenyl)ethan-1-one (20 g, 76 mmol, 85% yield) as a white powder. The ^{1}H NMR spectrum is in good agreement with the corresponding literature precedent.[25]

^{1}H NMR (600 MHz, $CDCl_3$) δ 8.11 (ddt, J = 8.9, 1.9, 0.9 Hz, 2H), 7.72 (ddt, J = 8.4, 1.7, 0.8 Hz, 2H), 7.37 – 7.31 (m, 2H), 7.27 – 7.24 (m, 3H), 4.31 (s, 2H).

^{19}F NMR (376 MHz, $CDCl_3$) δ -63.2.

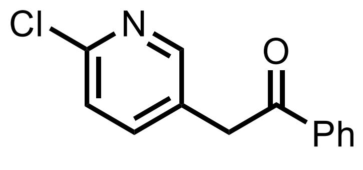

2-(6-chloropyridin-3-yl)-1-phenylethan-1-one (S27)

Ketone prepared from General Procedure B. Phenylmagnesium bromide (11.4 g, 62.9 mL, 1 molar, 1.5 equiv, 62.9 mmol) and 2-(6-chloropyridin-3-yl)-N-methoxy-N-methylacetamide (9.00 g, 1 equiv, 41.9

mmol) was used. The crude material was dry-loaded onto silica and subjected to flash chromatography hexanes/EtOAc. The collected fractions were concentrated *in vacuo* to afford 2-(6-chloropyridin-3-yl)-1-phenylethan-1-one (1.33 g, 5.74 mmol, 14% yield) as an off-white solid. The ^{1}H NMR spectrum is in good agreement with the corresponding literature precedent.[26]

^{1}H NMR (600 MHz, CDCl$_3$) δ 8.30 (dd, J = 2.5, 0.8 Hz, 1H), 8.03 – 8.00 (m, 2H), 7.64 – 7.58 (m, 2H), 7.53 – 7.49 (m, 2H), 7.32 (dd, J = 8.2, 0.7 Hz, 1H), 4.30 (s, 2H).

^{13}C NMR (151 MHz, CDCl$_3$) δ 195.88, 150.35, 150.20, 140.29, 136.07, 133.78, 130.19, 129.10, 128.91, 128.50, 128.39, 124.15, 41.42.

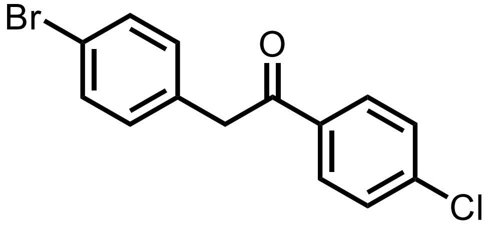

2-(4-bromophenyl)-1-(4-chlorophenyl)ethan-1-one (S28)

Ketone prepared from General Procedure B. (4-chlorophenyl) magnesium bromide (5.02 g, 23 mL, 1 molar, 1.5 equiv, 23.2 mmol) and 2-(4-bromophenyl)-N-methoxy-N- methylacetamide (4.0 g, 1 equiv, 15.5 mmol) was used. The crude material was dry-loaded onto silica and subjected to flash chromatography hexanes/EtOAc. The collected fractions were concentrated *in vacuo* to afford 2-(4-bromophenyl)-1-(4-chlorophenyl)ethan-1-one (3.97 g, 12.8 mmol, 83% yield) as a white powder. The ^{1}H NMR spectrum is in good agreement with the corresponding literature precedent.[10]

^{1}H NMR (400 MHz, CDCl$_3$) δ 7.95 – 7.90 (m, 2H), 7.48 – 7.42 (m, 4H), 7.15 – 7.10 (m, 2H), 4.21 (s, 2H).

C. Preparation of Aryldiazoketones

Warning: Diazo compounds are known to have thermal stability issues and are explosive hazards; work with diazo compounds should be performed in a well-ventilated hood, require the use of PPE, and careful handling of the reagents.[1]

General Procedure C for the Synthesis of Aryldiazoketones

Note: Hood lights were turned off to minimize product decomposition.

To a flame-dried vessel was added the corresponding ketone (1 equiv), *o*-NBSA (1.5 equiv), and acetonitrile (0.30 molar). The reaction mixture was back-filled with N_2 three times, and then the reaction mixture was cooled to 0 °C via ice-water bath. To the cooled reaction mixture was added 2,3,4,6,7,8,9,10-octahydropyrimido[1,2-a]azepine (4 equiv) drop-wise. The reaction mixture was stirred for 30 minutes in an ice-water bath. The wet crude material was diluted with a slurry containing SiO_2 with 1% TEA in 10% diethyl ether/90% hexanes and concentrated *in vacuo* to afford a dry-load. This dry-load was then subjected to a silica-plug doped with 1% TEA in 10% diethyl ether/90% hexanes. The colored material was collected, concentrated *in vacuo* to afford the corresponding diazoketone as generally a yellow or orange powder.

Storage: The vial was sealed under nitrogen and placed into a -20 °C freezer to minimize decomposition.

NMR acquisition: $CDCl_3$ was neutralized with barium carbonate and filtered before use. Due to long acquisition time of ^{13}C NMR, the diazo is not stable in deuterated solvents to obtain pure ^{13}C NMR spectra.

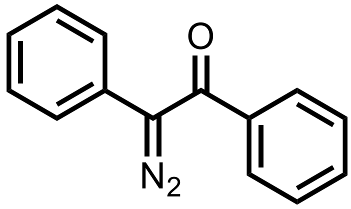

2-diazo-1,2-diphenylethan-1-one (1b)

Diazo was prepared from General Procedure C. 1,2-diphenylethan-1-one (5.0 g, 1 equiv, 25.48 mmol), *o*-NBSA (8.720 g, 1.5 equiv, 38.22 mmol), and 2,3,4,6,7,8,9,10-octahydropyrimido[1,2-a]azepine (15.51 g, 15.4 mL, 4 equiv, 101.9 mmol) was used. The crude material was subjected to a silica plug (15 inch

silica) doped with 1% TEA in 10% diethyl ether/90% hexanes. The dry-loaded, eluted through the silica plug (all yellow band collected). The material was concentrated *in vacuo* to afford 2-diazo-1,2- diphenylethan-1-one (3.65 g, 16.4 mmol, 65% yield) as an amorphous orange crystalline solid. The desired diazo was sensitive to deuterated solvents and was not stable in solution to obtain a pure ^{13}C NMR. The ^{1}H NMR spectrum is in good agreement with the corresponding literature precedent.[10]

^{1}H NMR (400 MHz, C_6D_6) δ 7.46 – 7.34 (m, 4H), 7.09 (t, 2H), 7.05 – 6.98 (m, 1H), 6.97 – 6.91 (m, 3H).

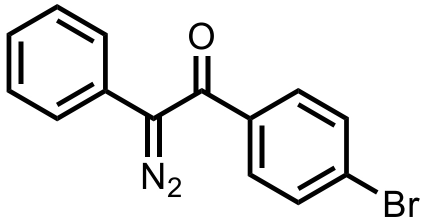

1-(4-bromophenyl)-2-diazo-2-phenylethan-1-one (1c)

Diazo was prepared from General Procedure C. 1-(4-bromophenyl)-2-phenylethan-1-one (5.73 g, 1 equiv, 20.83 mmol), *o*-NBSA (6.5 g, 1.3 equiv, 27.1 mmol), and 2,3,4,6,7,8,9,10-octahydropyrimido[1,2-a]azepine (6.98 g, 6.84 mL, 2.2 equiv, 45.82 mmol) was used. The crude material was subjected to a silica plug doped with 1% TEA in 10% diethyl ether/90% hexanes. The material was concentrated *in vacuo* to afford 1-(4-bromophenyl)-2-diazo-2-phenylethan-1-one (4.07 g, 13.5 mmol, 65% yield) as an amorphous orange powder. The desired diazo was sensitive to deuterated solvents and was not stable in solution to obtain a pure ^{13}C NMR.

^{1}H NMR (600 MHz, $CDCl_3$) δ 7.58 – 7.52 (m, 2H), 7.50 – 7.45 (m, 2H), 7.45 – 7.38 (m, 4H), 7.30 – 7.27 (m, 1H).

IR (neat): 3058, 2073, 1622, 1586, 1496, 1448, 1393, 1349, 1331, 1285, 1259, 1242, 1178, 1074, 1010, 910, 856, 829, 755, 739, 690, 651, 514, 490, 466, 450, 435, 417, 404 cm^{-1}.

HRMS (+p ESI): calc. mass for $C_{14}H_{10}O^{79}Br$ [M + H – N_2]$^-$ - 272.99095; obs. mass for $C_{14}H_{10}O^{79}Br$ [M + H – N_2]$^-$ - 272.99094.

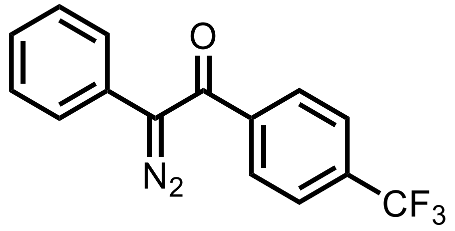

2-diazo-2-phenyl-1-(4-(trifluoromethyl)phenyl)ethan-1-one (1d)

Diazo was prepared from General Procedure C. 2-phenyl-1-(4-(trifluoromethyl)phenyl)ethan-1-one (500 mg, 1 equiv, 1.89 mmol), *o*-NBSA (518 mg, 1.2 equiv, 2.27 mmol), and 2,3,4,6,7,8,9,10-octahydropyrimido[1,2-a]azepine (576 mg, 0.57 mL, 2 equiv, 3.78 mmol) was used. The crude material was subjected to a silica plug doped with 1% TEA in 10% diethyl ether/90% hexanes. The material was concentrated *in vacuo* to afford 2-diazo-2-phenyl-1-(4-(trifluoromethyl)phenyl)ethan-1-one (490 mg, 1.69 mmol, 89% yield) as an amorphous orange powder. The desired diazo was sensitive to deuterated solvents and was not stable in solution to obtain a pure ^{13}C NMR.

^1H NMR (600 MHz, $CDCl_3$) δ 7.69 (q, J = 8.2 Hz, 4H), 7.42 (d, J = 6.9 Hz, 3H), 7.30 (t, J = 6.7 Hz, 2H).

^{19}F NMR (376 MHz, $CDCl_3$) δ -63.0.

IR (neat): 3015, 2970, 2098, 2078, 1738, 1614, 1590, 1573, 1495, 1448, 1407, 1363, 1323, 1284, 1241, 1217, 1176, 1124, 1111, 1075, 1062, 1024, 1015, 998, 964, 919, 866, 838, 773, 763, 753, 709, 689, 653, 653, 615, 591, 535, 512, 501, 493, 448, 407 cm^{-1}.

HRMS (+p ESI): calc. mass for $C_{15}H_{10}OF_3$ [M + H – N_2]$^-$ - 263.06783; obs. mass for $C_{15}H_{10}OF_3$ [M + H – N_2]$^-$ - 263.06783.

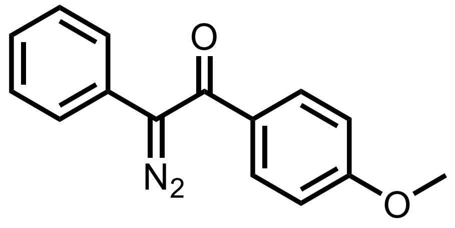

2-diazo-1-(4-methoxyphenyl)-2-phenylethan-1-one (1e)

Diazo was prepared from General Procedure C. 1-(4-methoxyphenyl)-2-phenylethan-1- one (226 mg, 1 equiv, 1.00 mmol), *o*-NBSA (342 mg, 1.5 equiv, 1.50 mmol), and 2,3,4,6,7,8,9,10-octahydropyrimido[1,2-a]azepine (609 mg, 603 µL, 4 equiv, 4.00 mmol) was used. The crude material was subjected to a silica plug doped with 1% TEA in 10% diethyl ether/90% hexanes. The material was concentrated *in vacuo* to afford 2-diazo- 1-(4-methoxyphenyl)-2-phenylethan-1-one (158.6 mg, 628.7 µmol, 63% yield) as an amorphous orange powder. The desired diazo was sensitive to deuterated solvents and was not stable in solution to obtain a pure ^{13}C NMR.

^{1}H NMR (600 MHz, CDCl$_3$) δ 7.64 – 7.58 (m, 2H), 7.47 – 7.37 (m, 4H), 7.28 – 7.23 (m, 1H), 6.93 – 6.87 (m, 2H), 3.85 (s, 3H).

IR (neat): 2934, 2838, 2066, 1737, 1600, 1573, 1509, 1496, 1461, 1417, 1347, 1328, 1307, 1283, 1248, 1171, 1111, 1069, 1029, 913, 859, 839, 786, 757, 691, 645, 621, 568, 519, 497, 406 cm^{-1}.

HRMS (+p ESI): calc. mass for $C_{15}H_{13}O_2$ [M + H – N$_2$]$^-$ - 225.09101; obs. mass for $C_{15}H_{13}O_2$ [M + H – N$_2$]$^-$ - 225.09094.

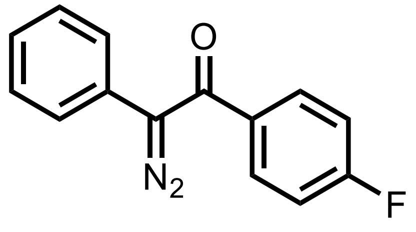

2-diazo-1-(4-fluorophenyl)-2-phenylethan-1-one (1f)

Diazo was prepared from General Procedure C. 1-(4-fluorophenyl)-2-phenylethan-1-one (1.71 g, 1 equiv, 7.98 mmol), *o*-NBSA (2.5 g, 1.3 equiv, 10.4 mmol), and 2,3,4,6,7,8,9,10- octahydropyrimido[1,2-a]azepine (1.45 g, 1.43 mL, 1.2 equiv, 9.57 mmol) was used. The crude material was subjected to a silica plug doped with 1% TEA in 10% diethyl ether/90% hexanes. The material was concentrated *in vacuo* to afford 2-diazo-1-(4-fluorophenyl)-2-phenylethan-1-one (1.22 g, 5.06 mmol, 63% yield) as an amorphous orange powder. The desired diazo was sensitive to deuterated solvents and was not stable in solution to obtain a pure ^{13}C NMR.

^{1}H NMR (600 MHz, $CDCl_3$) δ 7.65 – 7.60 (m, 2H), 7.45 – 7.38 (m, 3H), 7.30 – 7.24 (m, 2H), 7.12 – 7.06 (m, 2H).

^{19}F NMR (376 MHz, C_6D_6) δ -107.4, -107.4, -107.4, -107.4, -107.4, -107.4, -107.5, -107.5.

IR (neat): 3063, 2074, 1739, 1698, 1623, 1599, 1507, 1497, 1449, 1408, 1349, 1331, 1283, 1261, 1235, 1184, 1156, 1098, 1068, 1014, 980, 913, 863, 846, 757, 697, 638, 620, 609, 558, 517, 466, 453, 438, 427, 414 cm^{-1}.

HRMS (+p ESI): calc. mass for $C_{14}H_{10}OF$ [M + H – N_2]$^-$ - 213.07102; obs. mass for $C_{14}H_{10}OF$ [M + H – N_2]$^-$ - 213.07097.

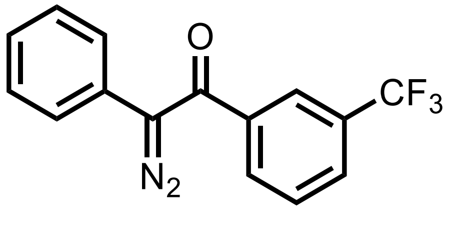

2-diazo-2-phenyl-1-(3-(trifluoromethyl)phenyl)ethan-1-one (1g)

Diazo was prepared from General Procedure C. 2-phenyl-1-(3- (trifluoromethyl)phenyl)ethan-1-one (793 mg, 1 equiv, 3.00 mmol), *o*-NBSA (1.03 g, 1.5 equiv, 4.50 mmol), and 2,3,4,6,7,8,9,10-octahydropyrimido[1,2-a]azepine (1.83 g, 1.81 mL, 4 equiv, 12.0 mmol) was used. The crude material was

subjected to a silica plug doped with 1% TEA in 10% diethyl ether/90% hexanes. The material was concentrated *in vacuo* to afford 2-diazo-2-phenyl-1-(3-(trifluoromethyl)phenyl)ethan-1-one (608.3 mg, 2.096 mmol, 70% yield) as an amorphous orange solid. The desired diazo was sensitive to deuterated solvents and was not stable in solution to obtain a pure ^{13}C NMR.

^{1}H NMR (400 MHz, C_6D_6) δ 7.69 (s, 1H), 7.31 (d, J = 7.8 Hz, 1H), 7.25 – 7.19 (m, 3H), 7.06 (dd, J = 8.5, 7.0 Hz, 2H), 6.97 – 6.92 (m, 1H), 6.70 (t, J = 7.9 Hz, 1H).

^{19}F NMR (376 MHz, C_6D_6) δ -62.6.

IR (neat): 3062, 2924, 2074, 1625, 1576, 1497, 1435, 1355, 1322, 1283, 1245, 1168, 1126, 1097, 1070, 1000, 911, 875, 814, 786, 775, 756, 731, 673, 663, 653, 614, 493, 464, 420, 410 cm^{-1}.

HRMS (+p ESI): calc. mass for $C_{15}H_{10}OF_3$ [M + H – N_2]$^-$ - 263.06783; obs. mass for $C_{15}H_{10}OF_3$ [M + H – N_2]$^-$ - 263.06774.

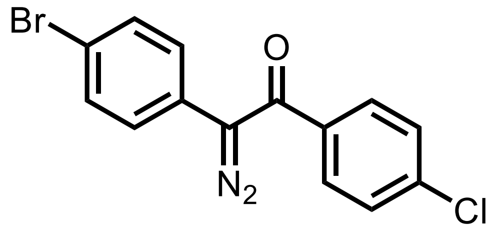

2-(4-bromophenyl)-1-(4-chlorophenyl)-2-diazoethan-1-one (1h)

Diazo was prepared from General Procedure C. 2-(4-bromophenyl)-1-(4-chlorophenyl)ethan-1-one (3.97 g, 1 equiv, 12.8 mmol), *o*-NBSA (4.4 g, 1.5 equiv, 19.2 mmol), and 2,3,4,6,7,8,9,10-octahydropyrimido[1,2-a]azepine (4.29 g, 4.25 mL, 2.2 equiv, 28.2 mmol) was used. The crude material was subjected to a silica plug doped with 1% TEA in 10% diethyl ether/90% hexanes. The material was concentrated *in vacuo* to afford 2-(4-bromophenyl)-1-(4-chlorophenyl)-2-diazoethan-1-one (3.79 g, 11.3 mmol, 88% yield) as an amorphous orange powder. The desired diazo was sensitive to deuterated solvents and was not stable in solution to obtain a pure ^{13}C NMR.

^{1}H NMR (600 MHz, $CDCl_3$) δ 7.57 – 7.51 (m, 4H), 7.44 – 7.39 (m, 2H), 7.36 – 7.30 (m, 2H).

IR (neat): 2074, 1624, 1588, 1488, 1398, 1337, 1272, 1242, 1179, 1091, 1013, 911, 857, 823, 744, 674, 610, 521, 489, 474, 455, 443, 434, 409 cm^{-1}.

HRMS (+p ESI): calc. mass for $C_{14}H_9O^{79}Br^{35}Cl$ [M + H – N_2]$^-$ - 306.95198; obs. mass for $C_{14}H_9O^{79}Br^{35}Cl$ [M + H – N_2]$^-$ - 306.95205.

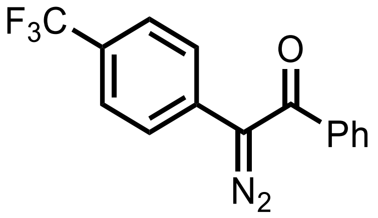

2-diazo-1-phenyl-2-(4-(trifluoromethyl)phenyl)ethan-1-one (1i)

Diazo was prepared from General Procedure C. 1-phenyl-2-(4- (trifluoromethyl)phenyl)ethan-1-one (793 mg, 1 equiv, 3.00 mmol), *o*-NBSA (1.03 g, 1.5 equiv, 4.50 mmol), and 2,3,4,6,7,8,9,10-octahydropyrimido[1,2-a]azepine (1.83 g, 1.81 mL, 4 equiv, 12.0 mmol) was used. The crude material was subjected to a silica plug doped with 1% TEA in 10% diethyl ether/90% hexanes. The material was concentrated *in vacuo* to afford 2-diazo-1-phenyl-2-(4-(trifluoromethyl)phenyl)ethan-1-one (636.3 mg, 2.192 mmol, 73% yield) as an amorphous yellow-orange powder. The desired diazo was sensitive to deuterated solvents and was not stable in solution to obtain a pure ^{13}C NMR.

^{1}H NMR (600 MHz, $CDCl_3$) δ 7.68 – 7.63 (m, 6H), 7.57 – 7.53 (m, 1H), 7.49 – 7.45 (m, 2H).

^{19}F NMR (376 MHz, C_6D_6) δ -62.9.

IR (neat): 3064, 2099, 2088, 1615, 1601, 1577, 1518, 1447, 1331, 1239, 1201, 1178, 1115, 1080, 1058, 1026, 1012, 854, 842, 829, 785, 711, 402 cm^{-1}.

HRMS (+p ESI): calc. mass for $C_{15}H_{10}OF_3$ [M + H – N_2]$^-$ - 263.06783; obs. mass for $C_{15}H_{10}OF_3$ [M + H – N_2]$^-$ - 263.06777.

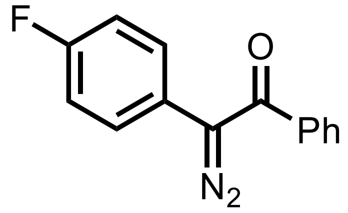

2-diazo-2-(4-fluorophenyl)-1-phenylethan-1-one (1j)

Diazo was prepared from General Procedure C. 2-(4-fluorophenyl)-1-phenylethan-1-one (643 mg, 1 equiv, 3.00 mmol), *o*-NBSA (1.03 g, 1.5 equiv, 4.50 mmol), and 2,3,4,6,7,8,9,10- octahydropyrimido[1,2-a]azepine (1.83 g, 1.81 mL, 4 equiv, 12.0 mmol) was used. The crude material was subjected to a silica plug doped with 1% TEA in 10% diethyl ether/90% hexanes. The material was concentrated *in vacuo* to afford 2-diazo-2-(4- fluorophenyl)-1-phenylethan-1-one (531.1 mg, 2.211 mmol, 74% yield) as an amorphous orange powder. The desired diazo was sensitive to deuterated solvents and was not stable in solution to obtain a pure ^{13}C NMR.

^{1}H NMR (600 MHz, $CDCl_3$) δ 7.63 – 7.59 (m, 2H), 7.53 – 7.50 (m, 1H), 7.49 – 7.40 (m, 4H), 7.15 – 7.09 (m, 2H).

^{19}F NMR (376 MHz, C_6D_6) δ -114.8 to -114.9 (tt, *J* = 8.4, 5.1 Hz).

IR (neat): 3111, 3060, 2077, 1892, 1738, 1621, 1575, 1508, 1489, 1444, 1415, 1342, 1309, 1286, 1243, 1228, 1180, 1171, 1103, 1064, 1025, 956, 856, 831, 815, 781, 717, 704, 671, 607, 584, 534, 508, 427 cm^{-1}.

HRMS (+p ESI): calc. mass for $C_{14}H_{10}OF$ $[M + H – N_2]^-$ - 213.07102; obs. mass for $C_{14}H_{10}OF$ $[M + H – N_2]^-$ - 213.07095.

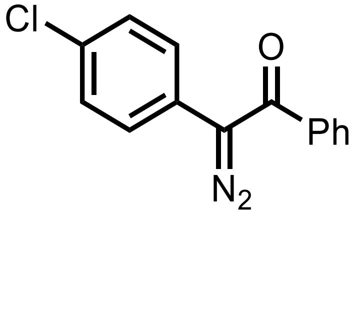

2-(4-chlorophenyl)-2-diazo-1-phenylethan-1-one (1k)

Diazo was prepared from General Procedure C. 2-(4-chlorophenyl)-1-phenylethan-1-one (231 mg, 1 equiv, 1.00 mmol), *o*-NBSA (342 mg, 1.5 equiv, 1.50 mmol), and 2,3,4,6,7,8,9,10- octahydropyrimido[1,2-a]azepine (609 mg, 603 μL, 4 equiv, 4.00 mmol) was used. The crude material was subjected to a silica plug doped with 1% TEA in 10% diethyl ether/90% hexanes. The material was concentrated *in vacuo* to afford 2-(4-chlorophenyl)-2- diazo-1-phenylethan-1-one (232.8 mg, 906.9 μmol, 91% yield) as an amorphous orange powder. The desired diazo was sensitive to deuterated solvents and was not stable in solution to obtain a pure ^{13}C NMR.

^{1}H NMR (600 MHz, $CDCl_3$) δ 7.64 – 7.59 (m, 2H), 7.54 – 7.51 (m, 1H), 7.47 – 7.41 (m, 4H), 7.40 – 7.37 (m, 2H).

IR (neat): 3110, 3055, 2089, 1969, 1911, 1673, 1621, 1587, 1578, 1492, 1446, 1402, 1337, 1274, 1243, 1209, 1187, 1160, 1094, 1063, 1024, 1013, 965, 934, 875, 850, 828, 796, 779, 759, 712, 701, 686, 661, 589, 547, 532, 501, 456, 447, 433, 424, 417, 406 cm^{-1}.

HRMS (+p ESI): calc. mass for $C_{14}H_{10}O^{35}Cl$ [M + H – N_2]$^-$ - 229.04147; obs. mass for $C_{14}H_{10}O^{35}Cl$ [M + H – N_2]$^-$ - 229.04145.

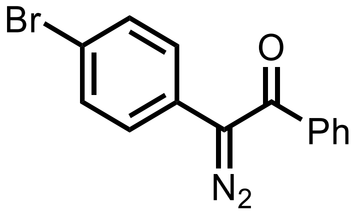

2-(4-bromophenyl)-2-diazo-1-phenylethan-1-one (1l)

Diazo was prepared from General Procedure C. 2-(4-bromophenyl)-1-phenylethan-1-one (825 mg, 1 equiv, 3.00 mmol), *o*-NBSA (1.03 g, 1.5 equiv, 4.50 mmol), and 2,3,4,6,7,8,9,10- octahydropyrimido[1,2-a]azepine (1.83 g, 1.81 mL, 4 equiv, 12.0 mmol) was used. The crude material was subjected to a silica plug

doped with 1% TEA in 10% diethyl ether/90% hexanes. The material was concentrated *in vacuo* to afford 2-(4-bromophenyl)-2- diazo-1-phenylethan-1-one (592.6 mg, 1.968 mmol, 66% yield) as an amorphous yellow powder. The desired diazo was sensitive to deuterated solvents and was not stable in solution to obtain a pure ^{13}C NMR.

^{1}H NMR (600 MHz, $CDCl_3$) δ 7.64 – 7.59 (m, 2H), 7.56 – 7.51 (m, 3H), 7.47 – 7.41 (m, 2H), 7.41 – 7.35 (m, 2H).

IR (neat): 3068, 2087, 1620, 1603, 1576, 1491, 1445, 1338, 1273, 1239, 1179, 1079, 1060, 1024, 1005, 848, 821, 782, 709, 699, 657, 588, 495, 436, 411 cm^{-1}.

HRMS (+p ESI): calc. mass for $C_{14}H_{10}O^{79}Br$ [M + H – N_2]$^-$ - 272.99095; obs. mass for $C_{14}H_{10}O^{79}Br$ [M + H – N_2]$^-$ - 272.99084.

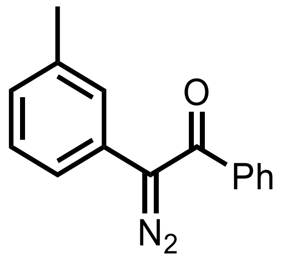

2-diazo-1-phenyl-2-(*m*-tolyl)ethan-1-one (1m)

Diazo was prepared from General Procedure C. 1-phenyl-2-(m-tolyl)ethan-1-one (631 mg, 1 equiv, 3.00 mmol), *o*-NBSA (1.03 g, 1.5 equiv, 4.50 mmol), and 2,3,4,6,7,8,9,10- octahydropyrimido[1,2-a]azepine (1.83 g, 1.81 mL, 4 equiv, 12.0 mmol) was used. The crude material was subjected to a silica plug doped with 1% TEA in 10% diethyl ether/90% hexanes. The material was concentrated *in vacuo* to afford 2-diazo-1-phenyl-2-(m- tolyl)ethan-1-one (448.2 mg, 1.897 mmol, 63% yield) as an amorphous orange powder. The desired diazo was sensitive to deuterated solvents and was not stable in solution to obtain a pure ^{13}C NMR.

^{1}**H NMR (600 MHz, $CDCl_3$) δ** 7.64 – 7.59 (m, 2H), 7.53 – 7.47 (m, 1H), 7.45 – 7.39 (m, 2H), 7.33 – 7.23 (m, 3H), 7.10 – 7.06 (m, 1H), 2.37 (s, 3H).

IR (neat): 3059, 2921, 2071, 1672, 1626, 1603, 1577, 1489, 1446, 1338, 1283, 1251, 1206, 1175, 1073, 1027, 1000, 908, 776, 709, 699, 662, 648, 496, 481, 471, 463, 446, 437, 420, 411, 406 cm^{-1}.

HRMS (+p ESI): calc. mass for $C_{15}H_{13}O$ [M + H – N_2]$^-$ - 209.09609; obs. mass for $C_{15}H_{13}O$ [M + H – N_2]$^-$ - 209.09617.

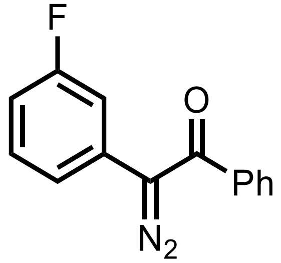

2-diazo-2-(3-fluorophenyl)-1-phenylethan-1-one (1n)

Diazo was prepared from General Procedure C. 2-(3-fluorophenyl)-1-phenylethan-1-one (643 mg, 1 equiv, 3.00 mmol), *o*-NBSA (1.03 g, 1.5 equiv, 4.50 mmol), and 2,3,4,6,7,8,9,10- octahydropyrimido[1,2-a]azepine (1.83 g, 1.81 mL, 4 equiv, 12.0 mmol) was used. The crude material was subjected to a silica plug doped with 1% TEA in 10% diethyl ether/90% hexanes. The material was concentrated *in vacuo* to afford 2-diazo-2-(3- fluorophenyl)-1-phenylethan-1-one (478.6 mg, 1.992 mmol, 66% yield) as an amorphous orange powder. The desired diazo was sensitive to deuterated solvents and was not stable in solution to obtain a pure ^{13}C NMR.

^{1}**H NMR (600 MHz, $CDCl_3$) δ** 7.65 – 7.61 (m, 2H), 7.53 (tt, J = 7.0, 1.3 Hz, 1H), 7.48 – 7.42 (m, 2H), 7.41 – 7.34 (m, 2H), 7.22 (ddd, J = 8.0, 1.9, 1.0 Hz, 1H), 6.95 (tdd, J = 8.3, 2.5, 0.9 Hz, 1H).

^{19}**F NMR (376 MHz, C_6D_6) δ** -111.4 to -111.5 (ddd, *J* = 10.4, 8.3, 6.2 Hz).

IR (neat): 3063, 2075, 1628, 1611, 1577, 1489, 1444, 1350, 1301, 1281, 1252, 1212, 1177, 1160, 1085, 1064, 1026, 1001, 911, 872, 853, 812, 801, 776, 710, 699, 679, 651, 643, 616, 555, 523, 506, 461, 449, 409 cm^{-1}.

HRMS (+p ESI): calc. mass for $C_{14}H_{10}OF$ $[M + H – N_2]^-$ - 213.07102; obs. mass for $C_{14}H_{10}OF$ $[M + H – N_2]^-$ - 213.07103.

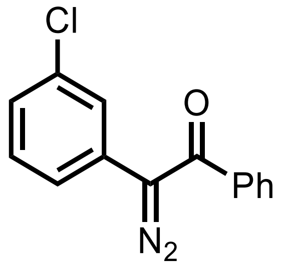

2-(3-chlorophenyl)-2-diazo-1-phenylethan-1-one (1o)

Diazo was prepared from General Procedure C. 2-(3-chlorophenyl)-1-phenylethan-1-one (461 mg, 1 equiv, 2.00 mmol), *o*-NBSA (685 mg, 1.5 equiv, 3.00 mmol), and 2,3,4,6,7,8,9,10- octahydropyrimido[1,2-a]azepine (1.22 g, 1.21 mL, 4 equiv, 8.00 mmol) was used. The crude material was subjected to a silica plug doped with 1% TEA in 10% diethyl ether/90% hexanes. The material was concentrated *in vacuo* to afford 2-(3-chlorophenyl)-2- diazo-1-phenylethan-1-one (376.5 mg, 1.467 mmol, 73% yield) as an amorphous orange powder. The desired diazo was sensitive to deuterated solvents and was not stable in solution to obtain a pure ^{13}C NMR.

^1H NMR (600 MHz, C_6D_6) δ 7.51 (s, 1H), 7.36 (d, J = 7.8 Hz, 2H), 7.05 – 6.99 (m, 1H), 6.98 – 6.90 (m, 4H), 6.77 (t, J = 8.0 Hz, 1H).

IR (neat): 3064, 2922, 2078, 1966, 1629, 1592, 1577, 1565, 1479, 1446, 1423, 1347, 1295, 1276, 1242, 1183, 1101, 1084, 1067, 1026, 995, 867, 777, 762, 749, 738, 709, 676, 664, 649, 638, 625, 611, 599, 586, 572, 559, 544, 519, 493, 482, 466, 453, 440, 427, 419 cm^{-1}.

HRMS (+p ESI): calc. mass for $C_{14}H_{10}O^{35}Cl$ [M + H – N_2]$^-$ - 229.04147; obs. mass for $C_{14}H_{10}O^{35}Cl$ [M + H – N_2]$^-$ - 229.04141.

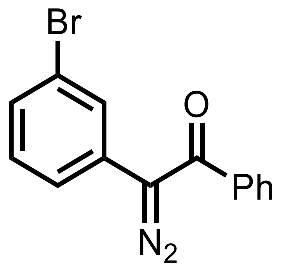

2-(3-bromophenyl)-2-diazo-1-phenylethan-1-one (1p)

Diazo was prepared from General Procedure C. 2-(3-bromophenyl)-1-phenylethan-1-one (825 mg, 1 equiv, 3.00 mmol), *o*-NBSA (1.03 g, 1.5 equiv, 4.50 mmol), and 2,3,4,6,7,8,9,10- octahydropyrimido[1,2-a]azepine (1.83 g, 1.81 mL, 4 equiv, 12.0 mmol) was used. The crude material was subjected to a silica plug doped with 1% TEA in 10% diethyl ether/90% hexanes. The material was concentrated *in vacuo* to afford 2-(3-bromophenyl)-2- diazo-1-phenylethan-1-one (645.8 mg, 2.145 mmol, 72% yield) as an amorphous yellow powder. The desired diazo was sensitive to deuterated solvents and was not stable in solution to obtain a pure ^{13}C NMR.

^1H NMR (600 MHz, $CDCl_3$) δ 7.71 (t, *J* = 1.9 Hz, 1H), 7.63 – 7.61 (m, 2H), 7.53 (ddt, *J* = 8.1, 6.9, 1.3 Hz, 1H), 7.47 – 7.41 (m, 4H), 7.39 (ddd, *J* = 8.0, 1.9, 1.0 Hz, 1H).

IR (neat): 3104, 3063, 2082, 1673, 1627, 1589, 1577, 1556, 1475, 1445, 1420, 1347, 1293, 1242, 1186, 1179, 1080, 1063, 1025, 993, 888, 860, 782, 775, 729, 707, 681, 667, 644, 616, 591, 552, 517, 473, 462, 454, 433, 410, 406 cm^{-1}.

HRMS (+p ESI): calc. mass for $C_{14}H_{10}O^{79}Br$ [M + H – N_2]$^-$ - 272.99095; obs. mass for $C_{14}H_{10}O^{79}Br$ [M + H – N_2]$^-$ - 272.99088.

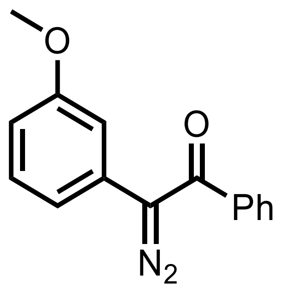

2-diazo-2-(3-methoxyphenyl)-1-phenylethan-1-one (1q)

Diazo was prepared from General Procedure C. 2-(3-methoxyphenyl)-1-phenylethan-1- one (679 mg, 1 equiv, 3.00 mmol), *o*-NBSA (1.03 g, 1.5 equiv, 4.50 mmol), and 2,3,4,6,7,8,9,10-octahydropyrimido[1,2-a]azepine (1.83 g, 1.81 mL, 4 equiv, 12.0 mmol) was used. The crude material was subjected to a silica plug doped with 1% TEA in 10% diethyl ether/90% hexanes. The material was concentrated *in vacuo* to afford 2-diazo-2-(3-methoxyphenyl)-1-phenylethan-1-one (547.7 mg, 2.171 mmol, 72% yield) as an amorphous orange powder. The desired diazo was sensitive to deuterated solvents and was not stable in solution to obtain a pure ^{13}C NMR.

^{1}H NMR (600 MHz, CDCl3) δ 7.62 (dd, J = 8.3, 1.3 Hz, 2H), 7.54 – 7.48 (m, 1H), 7.46 – 7.40 (m, 2H), 7.31 (t, J = 8.0 Hz, 1H), 7.12 (t, J = 2.2 Hz, 1H), 6.99 (ddd, J = 7.8, 1.8, 0.9 Hz, 1H), 6.81 (ddd, J = 8.3, 2.6, 0.9 Hz, 1H), 3.80 (s, 3H).

IR (neat): 3061, 3002, 2937, 2835, 2074, 1625, 1598, 1576, 1491, 1465, 1446, 1434, 1348, 1292, 1262, 1227, 1178, 1094, 1069, 1038, 1000, 921, 903, 854, 803, 774, 710, 699, 684, 652, 568, 523, 454, 440, 432, 417, 405 cm^{-1}.

HRMS (+p ESI): calc. mass for $C_{15}H_{13}O_2$ [M + H – N_2]$^-$ - 225.091; obs. mass for $C_{15}H_{13}O_2$ [M + H – N_2]$^-$ - 225.09107.

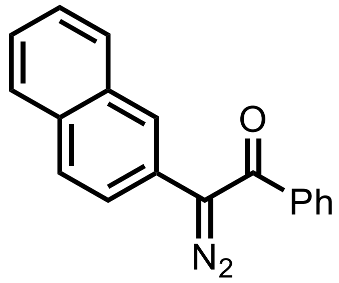

2-diazo-2-(naphthalen-2-yl)-1-phenylethan-1-one (1r)

Diazo was prepared from General Procedure C. 2-(naphthalen-2-yl)-1-phenylethan-1-one (739 mg, 1 equiv, 3.00 mmol), *o*-NBSA (1.03 g, 1.5 equiv, 4.50 mmol), and 2,3,4,6,7,8,9,10- octahydropyrimido[1,2-a]azepine (1.83 g, 1.81 mL, 4 equiv, 12.0 mmol) was used. The crude material was subjected to a silica plug doped with 1% TEA in 10% diethyl ether/90% hexanes. The material was concentrated *in vacuo* to afford 2-diazo-2-(naphthalen- 2-yl)-1-phenylethan-1-one (459.3 mg, 1.687 mmol, 56% yield) as an amorphous orange powder. The desired diazo was sensitive to deuterated solvents and was not stable in solution to obtain a pure ^{13}C NMR.

^{1}H NMR (600 MHz, $CDCl_3$) δ 8.02 (d, J = 1.9 Hz, 1H), 7.86 (d, J = 8.6 Hz, 1H), 7.85 – 7.79 (m, 2H), 7.68 – 7.64 (m, 1H), 7.55 – 7.46 (m, 4H), 7.46 – 7.40 (m, 2H).

IR (neat): 3056, 2073, 1625, 1597, 1576, 1505, 1445, 1356, 1326, 1268, 1232, 1177, 1061, 900, 855, 811, 780, 747, 709, 671, 597, 487, 474, 462, 456, 438, 420, 414, 408 cm^{-1}.

HRMS (+p ESI): calc. mass for $C_{18}H_{13}O$ [M + H – N$_2$]$^-$ - 245.09609; obs. mass for $C_{18}H_{13}O$ [M + H – N$_2$]$^-$ - 245.09593.

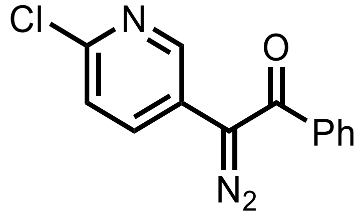

2-(6-chloropyridin-3-yl)-2-diazo-1-phenylethan-1-one (1s)

Diazo was prepared from General Procedure C. 2-(6-chloropyridin-3-yl)-1-phenylethan-1-one (695 mg, 1 Eq, 3.00 mmol), *o*-NBSA (1.03 g, 1.5 Eq, 4.50 mmol), and 2,3,4,6,7,8,9,10-octahydropyrimido[1,2-a]azepine (1.83 g, 1.81 mL, 4 Eq, 12.0 mmol) was used. The crude material was subjected to a silica plug doped with 1% TEA in 10% diethyl ether/90% hexanes. The material was concentrated *in vacuo* to afford 2-diazo-1-phenyl-2-(4-(trifluoromethyl)phenyl)ethan-1-one (636.3 mg, 2.192 mmol, 73.1% yield) as an amorphous yellow powder. The desired diazo was sensitive to deuterated solvents and was not stable in solution to obtain a pure ^{13}C NMR.

^1H NMR (600 MHz, $CDCl_3$) δ 8.48 (dd, *J* = 2.6, 0.7 Hz, 1H), 7.94 (dd, *J* = 8.5, 2.7 Hz, 1H), 7.66 – 7.61 (m, 2H), 7.58 – 7.54 (m, 1H), 7.50 – 7.46 (m, 2H), 7.38 (dd, *J* = 8.5, 0.8 Hz, 1H).

IR (neat): 3074, 2923, 2092, 1615, 1578, 1545, 1474, 1446, 1385, 1340, 1307, 1249, 1230, 1179, 1162, 1135, 1110, 1067, 1025, 1016, 978, 917, 854, 838, 782, 742, 729, 706, 693, 662, 631, 593, 544, 533, 501, 466, 416, 405 cm^{-1}.

HRMS (+p ESI): calc. mass for $C_{13}H_9ON_3{}^{35}Cl$ $[M + H]^+$ - 258.0429; obs. mass for $C_{13}H_9ON_3{}^{35}Cl$ $[M + H]^+$ - 258.04294.

D. Asymmetric C–H Insertion and Product Characterization

Warning: Diazo compounds are known to have thermal stability issues and are explosive hazards; work with diazo compounds should be performed in a well-ventilated hood, require the use of PPE, and careful handling of the reagents.[1]

General Procedure for the Aryldiazoketone C-H Insertion Reactions

Note: Hood lights were turned off to minimize diazo ketone decomposition.

To a flame-dried vessel charged with a stir-bar was added the corresponding substrate (5 - 10 equiv), Rh_2(*S*-TPPTTL)$_4$ (0.005 Eq, 0.5 mol %), and activated 4 Å MS (1 g/1 mmol diazoketone). The mixture was back-filled with N_2 three times. Dry distilled CH_2Cl_2 (0.125 M) was added to the mixture and the solution was allowed to stir at 25 °C for 10 minutes. Meanwhile to a separate flame-dried vessel was added dry distilled

CH_2Cl_2 (0.125 M) to the corresponding diazo ketone (1 equiv) under N_2. The diazo-solution was transferred to the reaction mixture containing the rhodium catalyst and trap in one-portion (fast addition). The total reaction concentration is 0.0625 M; half of 0.0625 M (0.125 M) was from the vessel containing trap & catalyst and the other half of 0.0625 M (0.125 M) was from the vessel containing the diazo. The reaction mixture was wrapped in tin foil and was allowed to stir vigorously overnight at 25 °C. The reaction mixture was then filtered through a celite plug, the filtrate concentrated *in vacuo* for crude ^{1}H NMR analysis to determine the regio- and diastereoselectivity. The crude was subjected to flash chromatography (generally 0 – 10% hexanes/ether) to afford the corresponding C-H insertion product.

Note: Solvent must be carefully dried (distilled over CaH_2 and stored on activated 4 Å MS). C-H substrates were dried in activated 4 Å MS and sparged with N_2 before used if the substrates are liquids.

Absolute configuration: the absolute configuration is tentatively assigned by analogy based on X-ray crystallography.

The below are unoptimized chiral catalyst screens for C-H insertion reactions that were used to examine the % yield, r.r., d.r., and ee.

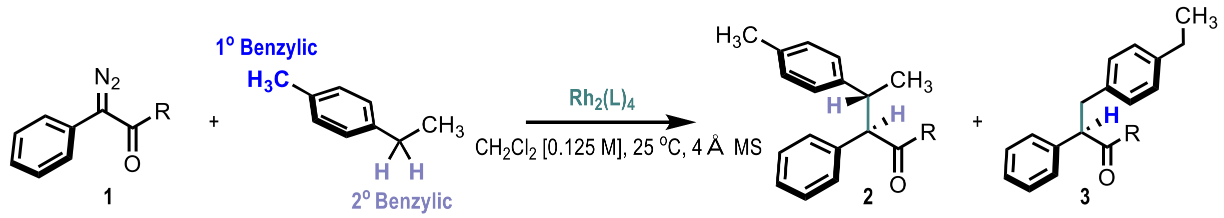

Entry	R	Catalyst	Product	yield, %[a]	r.r.[b]	d.r.[c]	ee (%)[d]
1	(*p*-Br)Ph	Rh_2(*S*-PTAD)$_4$	2c	15	2:1	>20:1	77
2	(*p*-CF_3)Ph	Rh_2(*S*-DOSP)$_4$	2d	24	>20:1	8:1	54
3	(*p*-CF_3)Ph	Rh_2(*S*-NTTL)$_4$	2d	1	>20:1	>20:1	32
4	(*p*-CF_3)Ph	Rh_2(*R*-TCPTAD)$_4$	2d	13	>20:1	>20:1	52
5	(*p*-CF_3)Ph	Rh_2(*R*-2-Cl-5-BrTPCP)$_4$	2d	3	5:1	>20:1	52
6	(*p*-CF_3)Ph	Rh_2(*S*-*p*-BrTPCP)$_4$	2d	4	7:1	>20:1	86

Figure S2. Initial unoptimized catalyst screen. aryldiazoketone in CH_2Cl_2 was added over 90 min to a solution of the substrate (5 equiv) and catalyst (0.5 mol%) in CH_2Cl_2 at 25 °C. The reaction was allowed to stir for an

additional 30 minutes after addition was completed. [a]Yield was determined by NMR with trichloroethylene as internal standard. [b,c]Regioselectivity and diastereoselectivity was determined by the reaction crude ^{1}H NMR spectra. [d]Enantiomeric excess (ee) data were measured using chiral HPLC analysis of purified product.

Crude ^{1}H NMR of Initial Catalyst Screen for the Aryldiazoketone C-H Insertion

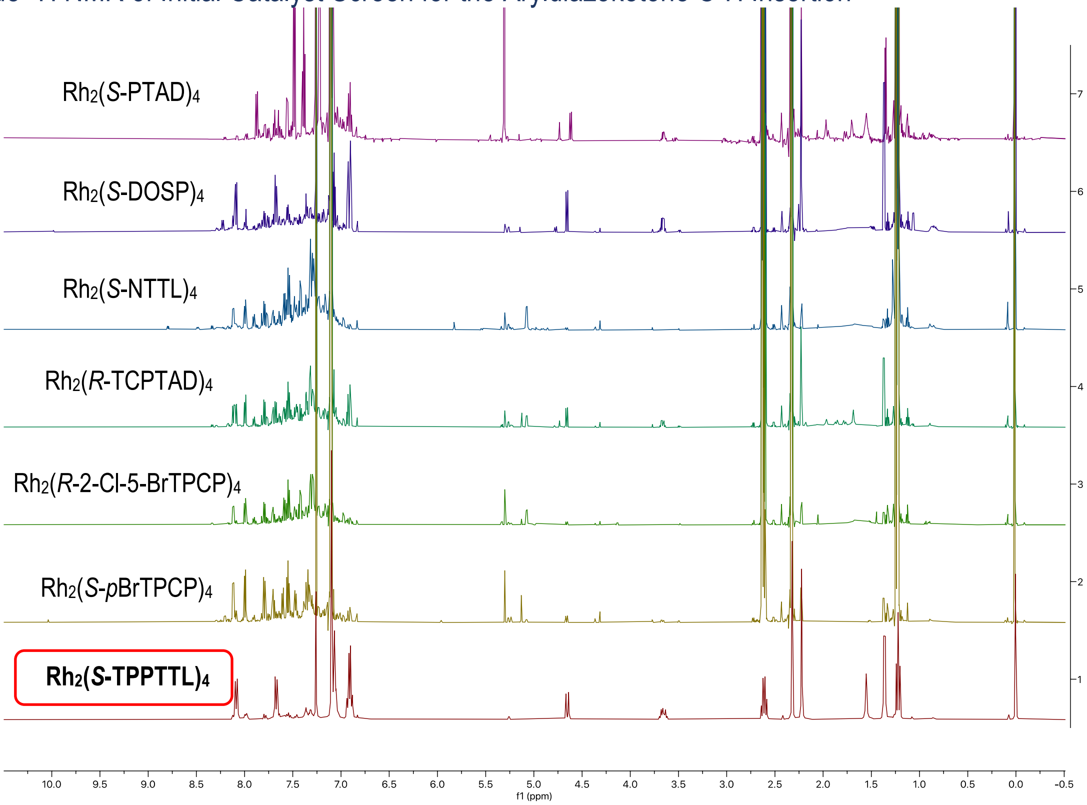

Figure S3. Crude ^{1}H NMR of catalyst screen. Reactivity with Rh_2(*S*-TPPTTL)$_4$ shows clean reactivity compared to other catalysts.

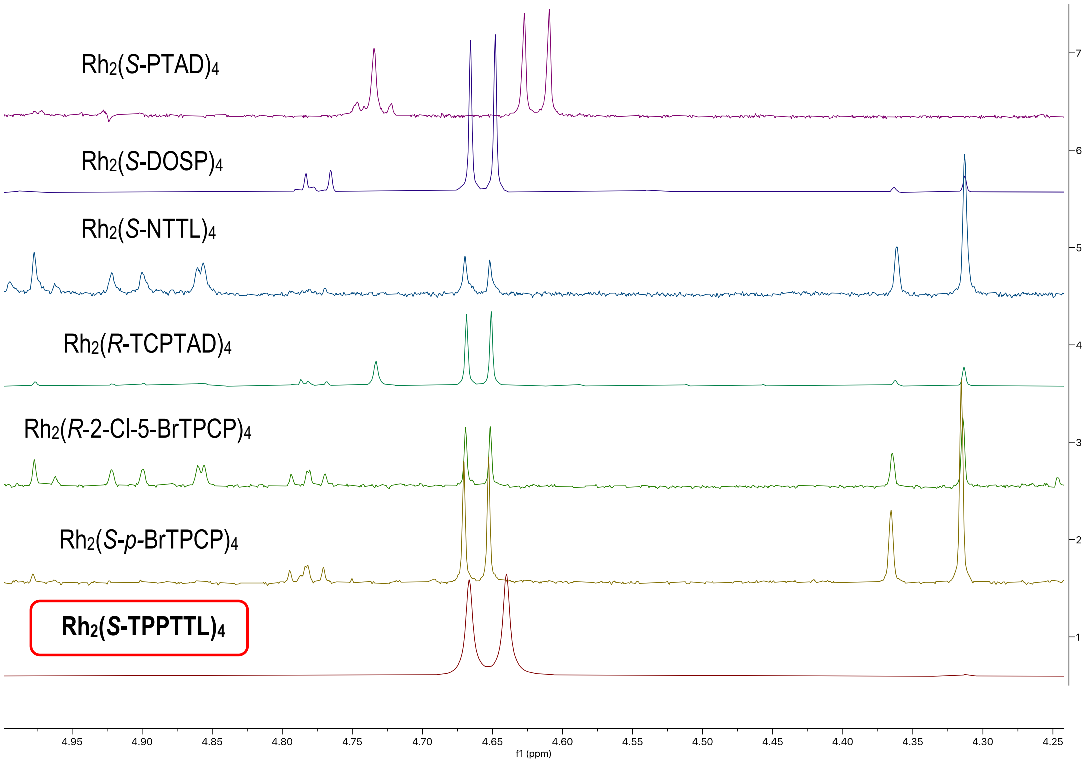

Figure S4. Crude ^{1}H NMR analysis of catalyst screen zoomed in to examine doublet benzylic C-H peak at 4.65 ppm. Reactivity with Rh_2(*S*-TPPTTL)$_4$ shows clean reactivity compared to other catalysts.

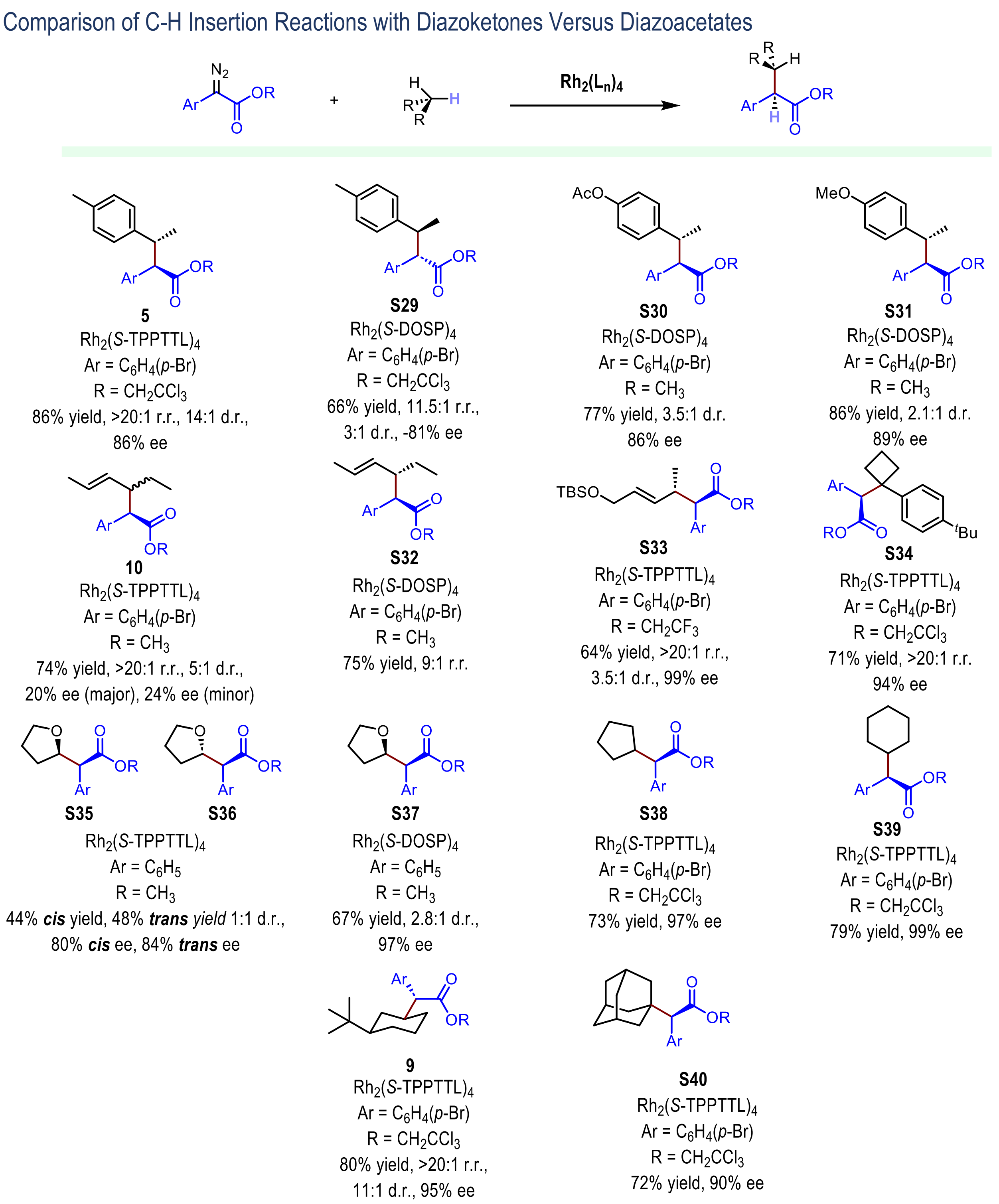

Figure S5. Comparison of diazoacetates and diazoketones. Conditions for **5**, **10**, **S35** & **S36**: aryldiazoacetate (0.3 mmol) in 2.4 mL of CH_2Cl_2 was added in one-portion to a solution of the substrate (5 equiv) and catalyst (0.5 mol%) in 2.4 mL of CH_2Cl_2 at 25 °C and allowed to react overnight. **S29**[27], **S30**[28], **S31**[28], **S32**[6], **S33**[8], **S34**[29], **S37**[30], **S38**[3], **S39**[3], **9**[3], and **S40**[3] are compounds from literature precedent.

Challenging Substrates for C-H insertions with Diazoketones

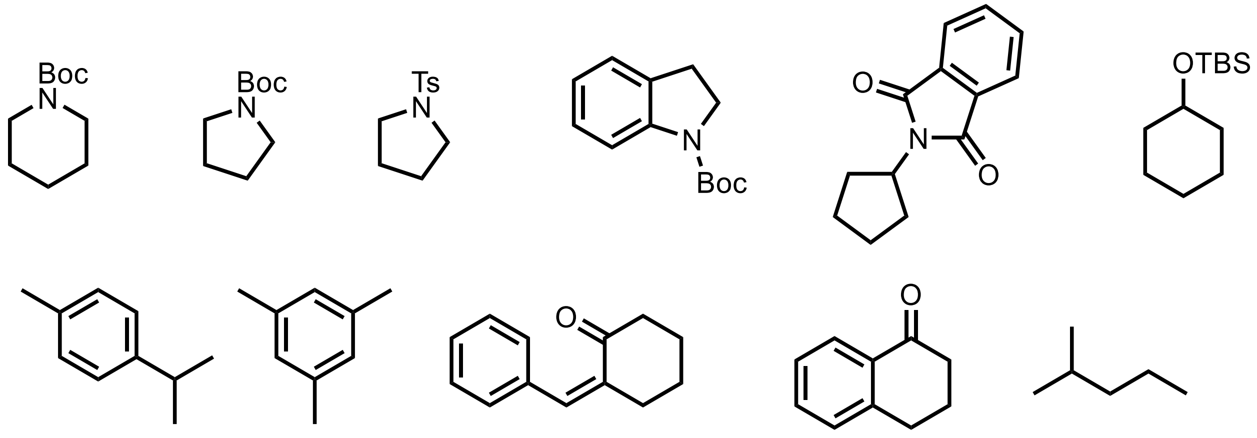

Figure S6. Challenging substrates that were difficult to achieve C-H insertions with diazoketones.

Product Characterizations

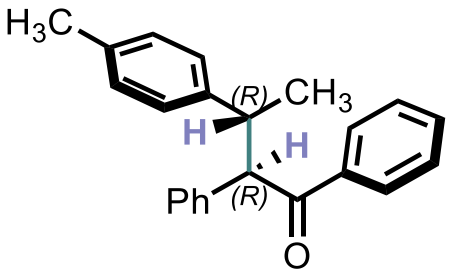

(2R,3R)-1,2-diphenyl-3-(p-tolyl)butan-1-one (2b)

This compound was prepared according to the general procedure for C-H insertion using 1-ethyl-4-methylbenzene (500 mg, 1.57 mL, 5 equiv, 11.25 mmol) as the substrate and 2-diazo-1,2-diphenylethan-1-one (500 mg, 1 equiv, 2.25 mmol) under the catalyst of Rh_2(*S*-TPPTTL)$_4$ (27.7 mg, 0.005 equiv, 0.5 mol%, 11.25 µmol). After flash chromatography (3% ether in hexanes), the product was obtained as an amorphous white solid (454 mg, 64% yield, >20:1 r.r., >20:1 d.r., 99% ee).

^{1}H NMR (600 MHz, $CDCl_3$) δ 8.04 – 7.99 (m, 2H), 7.54 – 7.48 (m, 1H), 7.42 (dd, J = 8.3, 7.2 Hz, 2H), 7.14 – 7.05 (m, 4H), 7.05 – 6.99 (m, 1H), 6.95 – 6.88 (m, 4H), 4.70 (d, J = 10.7 Hz, 1H), 3.67 (dq, J = 10.7, 6.7 Hz, 1H), 2.22 (s, 3H), 1.35 (d, J = 6.8 Hz, 3H).

^{13}C NMR (151 MHz, $CDCl_3$) δ 200.20, 141.20, 137.84, 137.64, 135.39, 132.94, 128.84, 128.68, 128.58, 128.57, 128.30, 127.60, 126.71, 61.00, 43.58, 21.08, 20.98.

IR (neat) 3024, 2963, 2923, 2873, 1671, 1596, 1579, 1514, 1492, 1447, 1373, 1346, 1288, 1267, 1233, 1202, 1175, 1102, 1074, 994, 932, 859, 813, 766, 754, 721, 698, 652, 642, 587, 555, 429, 418, 409 cm^{-1}.

HRMS (-p APCI) calc. mass for $C_{23}H_{21}O$ $[M–H]^-$ 313.1597; obs. mass for $C_{23}H_{21}O$ $[M–H]^-$ 313.1598.

$[\alpha]^{20}_D$: -152° (c 0.233 g/100 mL, CH_2Cl_2).

HPLC (OD column, 1.0 % *i*-propanol in hexane, 0.5 mL min^{-1}, 0.5 mg mL^{-1}, 30 min, UV 230 nm) retention times of 10.4 min (minor) and 12.2 min (major) 99% ee with Rh_2(*S*-TPPTTL)$_4$.

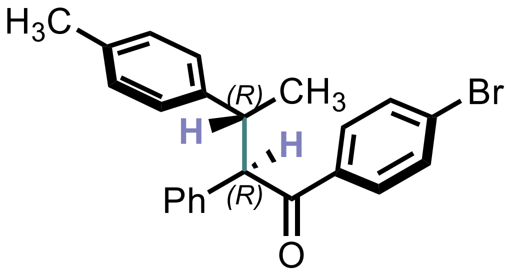

(2R,3R)-1-(4-bromophenyl)-2-phenyl-3-(p-tolyl)butan-1-one (2c)

This compound was prepared according to the general procedure for C-H insertion using 1-ethyl-4-methylbenzene (601 mg, 698 µL, 10 equiv, 5.00 mmol) as the substrate and 1-(4-bromophenyl)-2-diazo-2-phenylethan-1-one (151 mg, 1 equiv, 0.500 mmol) under the catalyst of Rh_2(*S*-TPPTTL)$_4$ (6.16 mg, 0.005 equiv, 0.5 mol%, 2.50 µmol). After flash chromatography (3% ether in hexanes), the product was obtained as an amorphous white solid (106 mg, 54% yield, >20:1 r.r., >20:1 d.r., 99% ee).

^{1}H NMR (600 MHz, $CDCl_3$) δ 7.88 – 7.84 (m, 2H), 7.57 – 7.52 (m, 2H), 7.10 – 7.01 (m, 5H), 6.94 – 6.87 (m, 4H), 4.61 (d, J = 10.6 Hz, 1H), 3.64 (dq, J = 10.6, 6.8 Hz, 1H), 2.22 (s, 3H), 1.34 (d, J = 6.8 Hz, 3H).

^{13}C NMR (151 MHz, $CDCl_3$) δ 199.06, 140.96, 137.48, 136.24, 135.49, 131.89, 130.10, 128.76, 128.71, 128.42, 128.15, 127.57, 126.88, 61.12, 43.44, 20.99.

IR (neat): 2991, 2942, 1674, 1581, 1514, 1483, 1452, 1394, 1331, 1307, 1292, 1266, 1199, 1174, 1107, 1069, 993, 907, 865, 852, 815, 807, 761, 734, 705, 669, 551, 520, 450, 419, 410 cm^{-1}.

HRMS (-p APCI): calc. mass for $C_{23}H_{20}O^{79}Br$ $[M – H]^-$ 391.0703; obs. mass for $C_{23}H_{20}O^{79}Br$ $[M – H]^-$ 391.0699.

$[\alpha]^{20}_D$: -62.8° (c 0.906 g/100 mL, EtOAc).

SFC (Trefoil CEL1, 1% of solvent blend (1:1 methanol:*i*-propanol with 0.2% formic acid), 2.5 mL min^{-1}, 10 min, UV 210 nm) retention times of 5.5 min (major) and 6.2 min (minor), 99% ee.

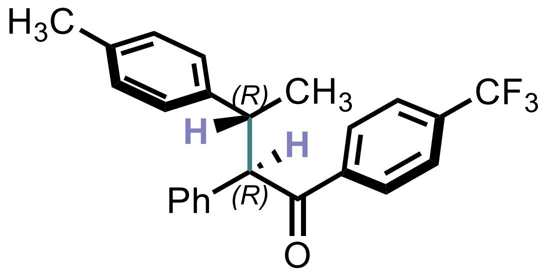

(2R,3R)-2-phenyl-3-(p-tolyl)-1-(4-(trifluoromethyl)phenyl)butan-1-one (2d)

This compound was prepared according to the general procedure for C-H insertion using 1-ethyl-4-methylbenzene (331 mg, 385 µL, 5 equiv, 2.76 mmol) as the substrate and 2-diazo-2-phenyl-1-(4-(trifluoromethyl)phenyl)ethan-1-one (160 mg, 1 equiv, 0.551 mmol) under the catalyst of Rh_2(*S*-TPPTTL)$_4$ (6.79 mg, 0.005 equiv, 0.5 mol%, 2.76 µmol). After flash chromatography (3% ether in hexanes), the product was obtained as a white solid (174 mg, 83% yield, >20:1 r.r., >20:1 d.r., 99% ee).

$[\alpha]^{20}_D$: -122.2° (c = 0.325 g/100 mL, EtOAc).

^{1}H NMR (800 MHz, $CDCl_3$) δ 8.09 (d, J = 8.1 Hz, 2H), 7.67 (d, J = 8.1 Hz, 2H), 7.15 – 7.02 (m, 5H), 6.96 – 6.86 (m, 4H), 4.66 (d, J = 10.6 Hz, 1H), 3.66 (dq, J = 10.7, 6.8 Hz, 1H), 2.22 (s, 3H), 1.37 (d, J = 6.8 Hz, 3H).

^{13}C NMR (201 MHz, $CDCl_3$) δ 199.1, 140.8, 140.2, 137.1, 135.6, 134.4, 134.2, 134.1, 133.9, 128.8, 128.8, 128.7, 128.5, 127.5, 127.0, 125.7, 125.6, 125.6, 125.6, 125.6, 124.2, 122.9, 121.5, 61.6, 43.4, 20.9, 20.9.

^{19}F NMR (753 MHz, $CDCl_3$) δ -63.2.

IR (neat): 2919, 1678, 1580, 1514, 1495, 1453, 1408, 1380, 1321, 1265, 1199, 1166, 1130, 1109, 1065, 996, 870, 857, 813, 774, 761, 746, 718, 706, 695, 667, 588, 550, 530, 518, 475, 413, 403 cm^{-1}.

HRMS (-p APCI): calc. mass for $C_{24}H_{20}F_3O$ $[M – H]^-$ 381.1461; obs. mass for $C_{24}H_{20}F_3O$ $[M – H]^-$ 381.1467.

HPLC (Chiralpak RRW column, 0.5% *i*-propanol in hexane, 0.5 mL min^{-1}, 30 min, UV 230 nm) retention times of 11.6 min (major) and 12.5 min (minor), 99% ee.

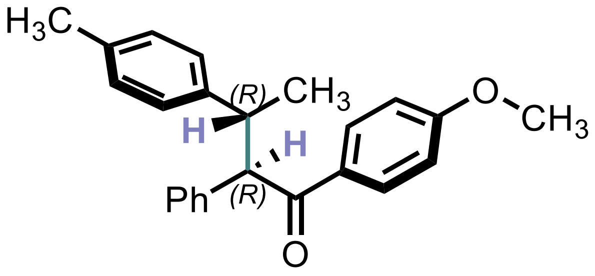

(2R,3R)-1-(4-methoxyphenyl)-2-phenyl-3-(p-tolyl)butan-1-one (2e)

This compound was prepared according to the general procedure for C-H insertion using 1-ethyl-4-methylbenzene (601 mg, 698 µL, 10 equiv, 5.00 mmol) as the substrate and 2-diazo-1-(4-methoxyphenyl)-2-phenylethan-1- one (126 mg, 1 equiv, 0.500 mmol) under the catalyst of Rh_2(*S*-TPPTTL)$_4$ (6.16 mg, 0.005 equiv, 0.5 mol%, 2.50 µmol). After flash chromatography (3% ether in hexanes), the product was obtained as an amorphous white solid (56 mg, 30% yield, >20:1 r.r., >20:1 d.r., >99% ee).

^{1}H NMR (600 MHz, $CDCl_3$) δ 8.03 – 8.00 (m, 2H), 7.12 – 7.04 (m, 4H), 7.03 – 6.96 (m, 1H), 6.93 – 6.88 (m, 6H), 4.65 (d, J = 10.7 Hz, 1H), 3.83 (s, 3H), 3.66 (dq, J = 10.9, 6.7 Hz, 1H), 2.21 (s, 3H), 1.33 (d, J = 6.7 Hz, 3H).

^{13}C NMR (151 MHz, $CDCl_3$) δ 198.6, 163.4, 141.4, 138.3, 135.3, 130.9, 130.6, 128.8, 128.6, 128.2, 127.6, 126.6, 113.8, 60.5, 55.5, 43.5, 21.1, 21.0.

IR (neat): 2962, 2931, 1668, 1598, 1574, 1510, 1453, 1418, 1306, 1288, 1260, 1205, 1168, 1115, 1073, 1031, 993, 922, 853, 814, 762, 739, 699, 635, 609, 553, 451, 433, 405 cm^{-1}.

HRMS (+p APCI): calc. mass for $C_{24}H_{25}O_2$ [M + H]$^+$ 345.1849; obs. mass for $C_{24}H_{25}O_2$ [M + H]$^+$ 345.1847.

$[\alpha]^{20}_D$: -60.3^o (c 0.410 g/100 mL, EtOAc).

HPLC (Chiralcel OD column, 0.5% *i*-propanol in hexane, 0.5 mL min^{-1}, 0.5 mg mL^{-1}, 60 min, UV 210 nm) retention times of 25.0 min (major) and 30.9 min (minor), >99% ee.

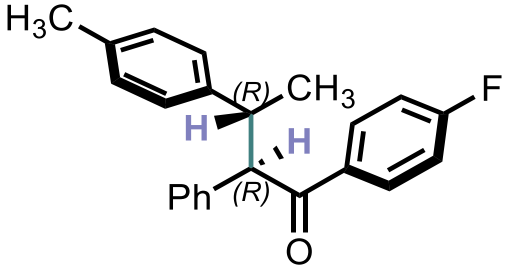

(2R,3R)-1-(4-fluorophenyl)-2-phenyl-3-(p-tolyl)butan-1-one (2f)

This compound was prepared according to the general procedure for C-H insertion using 1-ethyl-4-methylbenzene (601 mg, 698 µL, 10 equiv, 5.00 mmol) as the substrate and 2-diazo-1-(4-fluorophenyl)-2-phenylethan-1-one (120 mg, 1 equiv, 0.500 mmol) under the catalyst of Rh_2(*S*-TPPTTL)$_4$ (6.16 mg, 0.005 equiv, 0.5 mol%, 2.50 µmol). After flash chromatography (3% ether in hexanes), the product was obtained as an amorphous white solid (60 mg, 36% yield, >20:1 r.r., >20:1 d.r., 96% ee).

^{1}H NMR (600 MHz, CDCl$_3$) δ 8.04 (ddd, J = 8.9, 5.4, 1.8 Hz, 2H), 7.12 – 7.01 (m, 7H), 6.91 (qd, J = 8.1, 1.7 Hz, 4H), 4.63 (dd, J = 10.7, 1.6 Hz, 1H), 3.69 – 3.60 (m, 1H), 2.22 (s, 3H), 1.34 (dd, J = 6.9, 1.6 Hz, 3H).

^{13}C NMR (151 MHz, CDCl$_3$) δ 198.5, 166.5, 164.8, 141.0, 137.7, 135.4, 134.0 - 133.9 (d, J = 2.9 Hz), 131.2, 131.2, 128.8, 128.7, 128.4, 127.6, 126.8, 115.8, 115.6, 61.0, 43.5, 21.0, 20.9.

^{19}F NMR (565 MHz, CDCl$_3$) δ -105.4 (tt, J = 13.6, 10.2, 8.6, 5.3 Hz).

IR (neat): 3025, 2963, 2925, 1678, 1597, 1514, 1505, 1453, 1409, 1346, 1288, 1266, 1233, 1202, 1155, 995, 856, 826, 815, 761, 735, 698, 600, 551, 403 cm^{-1}.

HRMS (-p APCI): calc. mass for $C_{23}H_{20}OF$ [M - H]$^-$ 331.1503; obs. mass for $C_{23}H_{20}OF$ [M - H]$^-$ 331.1502.

[α]$^{20}_{D}$: -164.6° (c 0.327 g/100 mL, EtOAc).

SFC (ChiralCel OZ-3, 1% of solvent blend (1:1 methanol:*i*-propanol with 0.2% formic acid), 2.5 mL min^{-1}, 10 min, UV 210 nm) retention times of 4.6 min (major) and 9.1 min (minor), 96% ee.

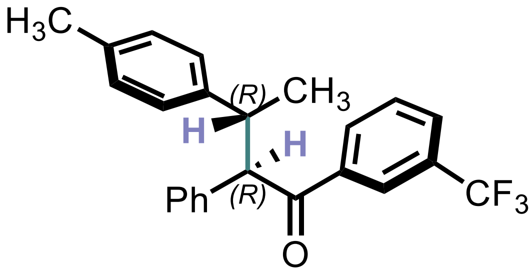

(2R,3R)-2-phenyl-3-(p-tolyl)-1-(3-(trifluoromethyl)phenyl)butan-1-one (2g)

This compound was prepared according to the general procedure for C-H insertion using 1-ethyl-4-methylbenzene (601 mg, 698 μL, 10 equiv, 5.00 mmol) as the substrate and 2-diazo-2-phenyl-1-(3-(trifluoromethyl)phenyl)ethan-1-one (145 mg, 1 equiv, 0.500 mmol) under the catalyst of Rh_2(*S*-TPPTTL)$_4$ (6.16 mg, 0.005 equiv, 0.5 mol%, 2.50 μmol). After flash chromatography (3% ether in hexanes), the product was obtained as an amorphous white solid (107 mg, 56% yield, >20:1 r.r., >20:1 d.r., >99% ee).

^{1}H NMR (600 MHz, $CDCl_3$) δ 8.26 (s, 1H), 8.16 (dt, J = 7.9, 1.5 Hz, 1H), 7.79 – 7.73 (m, 1H), 7.55 (t, J = 7.8 Hz, 1H), 7.12 – 7.05 (m, 4H), 7.08 – 7.00 (m, 1H), 6.92 (q, J = 8.1 Hz, 4H), 4.65 (d, J = 10.6 Hz, 1H), 3.67 (dq, J = 10.5, 6.7 Hz, 1H), 2.22 (s, 3H), 1.37 (d, J = 6.8 Hz, 3H).

^{13}C NMR (151 MHz, $CDCl_3$) δ 198.7, 140.8, 138.0, 137.2, 135.6, 131.7 - 130.9 (q, J = 33.0 Hz), 129.4 - 129.3 (q, J = 3.7 Hz), 128.8, 128.7, 128.5, 127.6, 127.0, 125.4 (q, J = 3.9 Hz), 126.4 & 124.6 - 121.0 (q, J = 272.6 Hz), 61.4, 43.5, 21.0, 20.9.

^{19}F NMR (565 MHz, $CDCl_3$) δ -62.8.

IR (neat): 2927, 1685, 1609, 1514, 1492, 1453, 1327, 1287, 1259, 1167, 1127, 1095, 1072, 1032, 999, 923, 813, 779, 760, 717, 697, 670, 648, 579, 552, 418 cm^{-1}.

HRMS (-p APCI): calc. mass for $C_{24}H_{20}OF_3$ [M – H]$^-$ 381.1471; obs. mass for $C_{24}H_{20}OF_3$ [M – H]$^-$ 381.1470.

[α]$^{20}_{D}$: -136.3° (c 0.635 g/100 mL, EtOAc).

HPLC (Chiralcel OD column, 0.5% *i*-propanol in hexane, 0.25 mL min^{-1}, 0.5 mg mL^{-1}, 30 min, UV 210 nm) retention times of 20.3 min (major) and 23.0 min (minor), >99% ee.

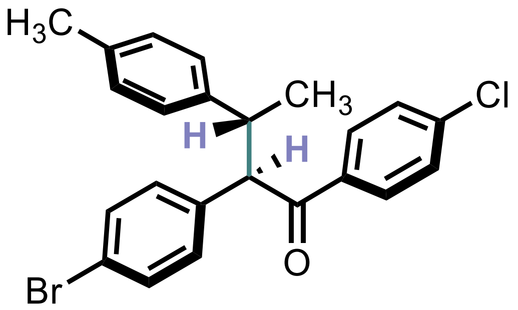

(2R,3R)-2-(4-bromophenyl)-1-(4-chlorophenyl)-3-(p-tolyl)butan-1-one (2h)

This compound was prepared according to the general procedure for C-H insertion using 1-ethyl-4-methylbenzene (601 mg, 698 µL, 10 equiv, 5.00 mmol) as the substrate and 2-(4-bromophenyl)-1-(4-chlorophenyl)-2- diazoethan-1-one (168 mg, 1 equiv, 0.500 mmol) under the catalyst of Rh_2(*S*-TPPTTL)$_4$ (6.16 mg, 0.005 equiv, 0.5 mol%, 2.50 µmol). After flash chromatography (3% ether in hexanes), the product was obtained as an amorphous off-white solid (122 mg, 57% yield, >20:1 r.r., >20:1 d.r., 90% ee).

^{1}H NMR (600 MHz, $CDCl_3$) δ 7.94 – 7.89 (m, 2H), 7.43 – 7.37 (m, 2H), 7.23 – 7.17 (m, 2H), 6.97 – 6.86 (m, 6H), 4.59 (d, *J* = 10.7 Hz, 1H), 3.60 (dq, *J* = 10.8, 6.8 Hz, 1H), 2.24 (s, 3H), 1.32 (d, *J* = 6.8 Hz, 3H).

^{13}C NMR (151 MHz, $CDCl_3$) δ 198.61, 140.56, 139.72, 136.62, 135.77, 135.59, 132.52, 131.56, 131.30, 130.38, 129.92, 129.54, 129.02, 128.92, 127.49, 121.00, 60.30, 43.48, 21.05, 21.00.

IR (neat): 2964, 2925, 1679, 1588, 1569, 1515, 1485, 1456, 1398, 1375, 1316, 1287, 1266, 1203, 1172, 1093, 1074, 1011, 997, 854, 814, 800, 741, 721, 681, 558, 512, 471, 423, 410 cm^{-1}.

HRMS (-p APCI): calc. mass for $C_{23}H_{19}OBrCl$ [M – H]$^-$ 425.0304; obs. mass for $C_{23}H_{19}OBrCl$ [M – H]$^-$ 425.0308.

[α]$^{20}_{D}$: -41.5° (c 0.334 g/100 mL, EtOAc).

HPLC (Chiralpak AD-H column, 0.3% *i*-propanol in hexane, 0.8 mL min^{-1}, 0.5 mg mL^{-1}, 60 min, UV 210 nm) retention times of 25.0 min (minor) and 30.9 min (major), 90% ee.

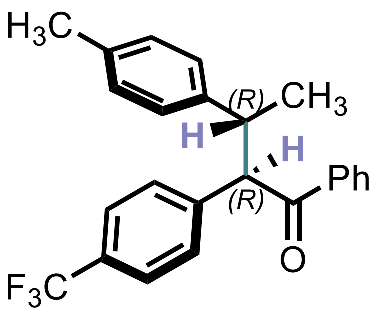

(2R,3R)-1-phenyl-3-(p-tolyl)-2-(4-(trifluoromethyl)phenyl)butan-1-one (2i)

This compound was prepared according to the general procedure for C-H insertion using 1-ethyl-4-methylbenzene (601 mg, 698 µL, 10 equiv, 5.00 mmol) as the substrate and 2-diazo-1-phenyl-2-(4-(trifluoromethyl)phenyl)ethan-1-one (145 mg, 1 Eq, 0.500 mmol) under the catalyst of Rh_2(*S*-TPPTTL)$_4$ (6.16 mg, 0.005 equiv, 0.5 mol%, 2.50 µmol). After flash chromatography (3% ether in hexanes), the product was obtained as an amorphous off-white solid (93 mg, 50% yield, >20:1 r.r., >20:1 d.r., 96% ee).

^{1}H NMR (600 MHz, $CDCl_3$) δ 8.03 – 7.98 (m, 2H), 7.58 – 7.52 (m, 1H), 7.48 – 7.42 (m, 2H), 7.33 (d, J = 8.1 Hz, 1H), 7.23 (d, J = 7.9 Hz, 2H), 6.96 – 6.87 (m, 4H), 4.79 (d, J = 10.8 Hz, 1H), 3.67 (dq, J = 10.8, 6.7 Hz, 1H), 2.23 (s, 3H), 1.35 (d, J = 6.8 Hz, 3H).

^{13}C NMR (151 MHz, $CDCl_3$) δ ^{13}C NMR (151 MHz, $CDCl_3$) **δ** 199.7, 141.9, 140.5, 137.3, 135.8, 133.4, 130.3 – 130.0 (dd, J = 14.2 Hz), 129.09, 128.9, 128.8, 128.5, 127.5, 125.3 - 125.2 (q, *J* = 3.8 Hz), 126.7 - 121.34 (q, *J* = 272.3 Hz), 60.5, 43.8, 21.1, 21.0.

^{19}F NMR (565 MHz, $CDCl_3$) δ -62.5.

IR (neat): 2966, 1680, 1615, 1596, 1580, 1515, 1448, 1419, 1324, 1290, 1267, 1203, 1165, 1123, 1068, 1019, 1001, 833, 814, 745, 721, 700, 656, 643, 599, 554, 437 cm^{-1}.

HRMS (-p APCI): calc. mass for $C_{24}H_{20}OF_3$ [M – H]$^-$ 381.1471; obs. mass for $C_{24}H_{20}OF_3$ [M – H]$^-$ 381.1468.

[α]$^{20}_{D}$: -89.8° (c 1.351 g/100 mL, EtOAc).

HPLC (Chiralcel OD column, 1.0% *i*-propanol in hexane, 0.5 mL min^{-1}, 0.5 mg mL^{-1}, 30 min, UV 230 nm) retention times of 9.9 min (minor) and 13.4 min (major), 96% ee.

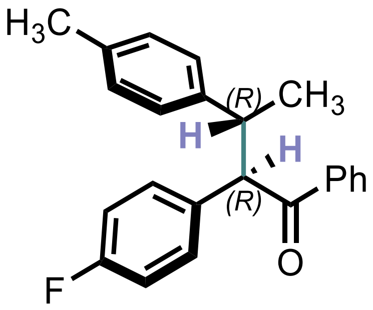

(2R,3R)-2-(4-fluorophenyl)-1-phenyl-3-(p-tolyl)butan-1-one (2j)

This compound was prepared according to the general procedure for C-H insertion using 1-ethyl-4-methylbenzene (601 mg, 698 µL, 10 equiv, 5.00 mmol) as the substrate and 2-diazo-2-(4-fluorophenyl)-1-phenylethan-1-one (120 mg, 1 equiv, 0.500 mmol) under the catalyst of Rh_2(*S*-TPPTTL)$_4$ (6.16 mg, 0.005 equiv, 0.5 mol%, 2.50 µmol). After flash chromatography (3% ether in hexanes), the product was obtained as an amorphous off-white solid (52 mg, 31% yield, >20:1 r.r., >20:1 d.r., 94% ee).

^{1}H NMR (600 MHz, CDCl$_3$) δ 8.02 – 7.97 (m, 2H), 7.53 (ddt, J = 7.7, 6.8, 1.2 Hz, 1H), 7.47 – 7.40 (m, 2H), 7.09 – 7.03 (m, 2H), 6.96 – 6.87 (m, 4H), 6.80 – 6.73 (m, 2H), 4.68 (d, J = 10.8 Hz, 1H), 3.62 (dq, J = 10.8, 6.8 Hz, 1H), 2.23 (s, 3H), 1.34 (d, J = 6.7 Hz, 3H).

^{13}C NMR (151 MHz, CDCl$_3$) δ 200.20, 162.45, 160.82, 140.96, 137.48, 135.57, 133.65, 133.63, 133.10, 130.28, 130.22, 128.80, 128.66, 128.52, 127.55, 115.28, 115.14, 60.05, 43.72, 21.00, 20.98.

^{19}F NMR (565 MHz, CDCl$_3$) δ -115.8 to -115.9 (td, *J* = 8.7, 4.7 Hz).

IR (neat): 2963, 2926, 1678, 1597, 1579, 1507, 1447, 1417, 1375, 1348, 1318, 1288, 1266, 1224, 1203, 1177, 1158, 1106, 1015, 1001, 937, 832, 813, 756, 732, 721, 690, 656, 642, 605, 565, 527, 513, 433, 422, 407 cm^{-1}.

HRMS (-p APCI): calc. mass for $C_{23}H_{20}OF$ $[M-H]^-$ 331.1503; obs. mass for $C_{23}H_{20}OF$ $[M-H]^-$ 331.1499.

$[\alpha]^{20}_D$: -153° (c 0.291 g/100 mL, EtOAc).

HPLC (Chiralcel OD column, 1.0% *i*-propanol in hexane, 0.5 mL min^{-1}, 0.5 mg mL^{-1}, 30 min, UV 230 nm) retention times of 10.7 min (minor) and 14.0 min (major), 94% ee.

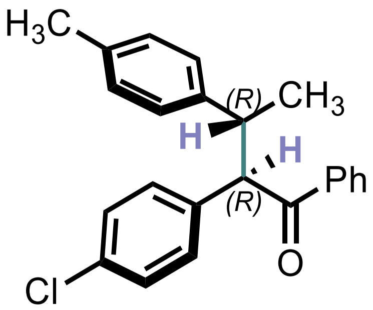

(2R,3R)-2-(4-chlorophenyl)-1-phenyl-3-(p-tolyl)butan-1-one (2k)

This compound was prepared according to the general procedure for C-H insertion using 1-ethyl-4-methylbenzene (601 mg, 698 µL, 10 equiv, 5.00 mmol) as the substrate and 2-(4-chlorophenyl)-2-diazo-1-phenylethan-1-one (128 mg, 1 equiv, 0.500 mmol) under the catalyst of Rh_2(*S*-TPPTTL)$_4$ (6.16 mg, 0.005 equiv, 0.5 mol%, 2.50 µmol). After flash chromatography (3% ether in hexanes), the product was obtained as an amorphous yellow solid (77 mg, 44% yield, >20:1 r.r., >20:1 d.r., 94% ee).

^{1}H NMR (600 MHz, $CDCl_3$) δ 8.01 – 7.96 (m, 2H), 7.56 – 7.50 (m, 1H), 7.46 – 7.40 (m, 2H), 7.04 (t, J = 0.9 Hz, 4H), 6.97 – 6.87 (m, 4H), 4.68 (d, J = 10.8 Hz, 1H), 3.62 (dq, J = 10.6, 6.7 Hz, 1H), 2.24 (s, 3H), 1.33 (d, J = 6.8 Hz, 3H).

^{13}C NMR (151 MHz, $CDCl_3$) δ 199.97, 140.82, 137.41, 136.42, 135.66, 133.18, 132.61, 130.09, 128.88, 128.69, 128.52, 128.52, 127.53, 60.15, 43.63, 21.11, 21.00.

IR (neat): 2963, 2925, 1678, 1595, 1580, 1515, 1489, 1447, 1409, 1375, 1348, 1318, 1287, 1266, 1203, 1174, 1091, 1014, 1001, 941, 866, 814, 801, 732, 716, 687, 657, 558, 518, 456, 417 cm^{-1}.

HRMS (-p APCI): calc. mass for $C_{23}H_{20}O^{35}Cl$ $[M-H]^-$ 347.1208; obs. mass for $C_{23}H_{20}O^{35}Cl$ $[M-H]^-$ 347.1202.

[α]$^{20}_{D}$: -100.7° (c 0.871 g/100 mL, EtOAc).

HPLC (Chiralcel OD column, 1.0% *i*-propanol in hexane, 0.5 mL min^{-1}, 0.5 mg mL^{-1}, 30 min, UV 230 nm) retention times of 10.2 min (minor) and 13.6 min (major), 94% ee.

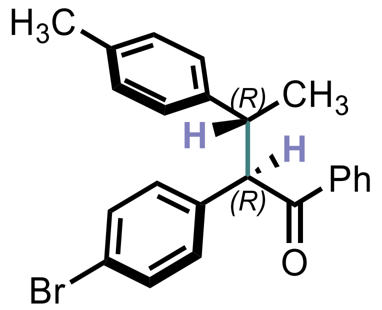

(2R,3R)-2-(4-bromophenyl)-1-phenyl-3-(p-tolyl)butan-1-one (2l)

This compound was prepared according to the general procedure for C-H insertion using 1-ethyl-4-methylbenzene (601 mg, 698 μL, 10 equiv, 5.00 mmol) as the substrate and 2-(4-bromophenyl)-2-diazo-1-phenylethan-1-one (151 mg, 1 equiv, 0.500 mmol) under the catalyst of Rh_2(*S*-TPPTTL)$_4$ (6.16 mg, 0.005 equiv, 0.5 mol%, 2.50 μmol). After flash chromatography (3% ether in hexanes), the product was obtained as an amorphous light-yellow solid (76 mg, 39% yield, >20:1 r.r., >20:1 d.r., 94% ee).

^{1}H NMR (600 MHz, $CDCl_3$) δ 8.01 – 7.95 (m, 2H), 7.56 – 7.50 (m, 1H), 7.48 – 7.40 (m, 2H), 7.22 – 7.16 (m, 2H), 7.01 – 6.96 (m, 2H), 6.95 (d, J = 8.0 Hz, 2H), 6.93 – 6.88 (m, 2H), 4.68 (d, J = 10.8 Hz, 1H), 3.62 (dq, J = 10.7, 6.8 Hz, 1H), 2.24 (s, 3H), 1.33 (d, J = 6.8 Hz, 3H).

^{13}C NMR (151 MHz, $CDCl_3$) δ 199.90, 140.79, 137.38, 136.94, 135.68, 133.20, 131.45, 130.47, 128.90, 128.69, 128.52, 127.52, 120.83, 60.19, 43.58, 21.13, 21.00.

IR (neat): 2963, 2924, 1678, 1595, 1579, 1515, 1485, 1447, 1405, 1374, 1348, 1318, 1288, 1266, 1202, 1175, 1108, 1073, 1010, 1001, 813, 800, 779, 731, 721, 704, 686, 655, 642, 630, 557, 515, 423, 411 cm^{-1}.

HRMS (-p APCI): calc. mass for $C_{23}H_{20}O^{79}Br$ [M – H]$^-$ 391.0703; obs. mass for $C_{23}H_{20}O^{79}Br$ [M – H]$^-$ 391.0700.

[α]$^{20}_{D}$: -69.5° (c 0.375 g/100 mL, EtOAc).

HPLC (Chiralcel OD column, 1.0% *i*-propanol in hexane, 0.5 mL min^{-1}, 0.5 mg mL^{-1}, 30 min, UV 230 nm) retention times of 10.9 min (minor) and 14.6 min (major), 94% ee.

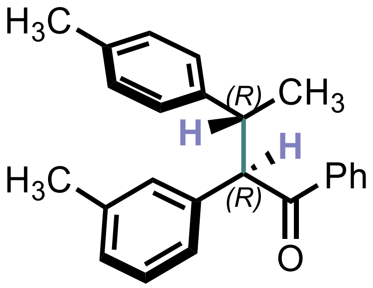

(2R,3R)-1-phenyl-2-(m-tolyl)-3-(p-tolyl)butan-1-one (2m)

This compound was prepared according to the general procedure for C-H insertion using 1-ethyl-4-methylbenzene (601 mg, 698 µL, 10 equiv, 5.00 mmol) as the substrate and 2-diazo-1-phenyl-2-(m-tolyl)ethan-1-one (118 mg, 1 equiv, 0.500 mmol) under the catalyst of Rh_2(*S*-TPPTTL)$_4$ (6.16 mg, 0.005 equiv, 0.5 mol%, 2.50 µmol). After flash chromatography (3% ether in hexanes), the product was obtained as an amorphous clear yellow oil (75 mg, 46% yield, >20:1 r.r., >20:1 d.r., 96% ee).

^{1}H NMR (600 MHz, $CDCl_3$) δ 8.02 (dd, J = 8.4, 1.3 Hz, 2H), 7.55 – 7.47 (m, 1H), 7.45 – 7.38 (m, 2H), 6.97 – 6.88 (m, 6H), 6.88 – 6.80 (m, 2H), 4.66 (d, J = 10.7 Hz, 1H), 3.64 (dq, J = 10.7, 6.7 Hz, 1H), 2.22 (s, 3H), 2.16 (s, 3H), 1.34 (d, J = 6.8 Hz, 3H).

^{13}C NMR (151 MHz, $CDCl_3$) δ 200.3, 141.3, 137.8, 137.7, 137.7, 135.3, 132.9, 129.3, 128.6, 128.6, 128.6, 128.1, 127.6, 127.5, 126.1, 60.9, 43.5, 21.3, 21.0, 21.0.

IR (neat): 3022, 2962, 2922, 2870, 1732, 1677, 1596, 1580, 1515, 1487, 1447, 1375, 1349, 1318, 1287, 1267, 1233, 1198, 1180, 1105, 1002, 927, 857, 814, 779, 749, 720, 703, 693, 644, 602, 539, 446, 422, 413 cm^{-1}.

HRMS (-p APCI): calc. mass for $C_{24}H_{23}O$ $[M – H]^-$ 327.1754; obs. mass for $C_{24}H_{23}O$ $[M – H]^-$ 327.1754.

$[\alpha]^{20}_D$: -121.2° (c 0.661 g/100 mL, EtOAc).

HPLC (Chiralcel OD column, 1.0% *i*-propanol in hexane, 0.5 mL min^{-1}, 0.5 mg mL^{-1}, 30 min, UV 230 nm) retention times of 9.0 min (minor) and 10.5 min (major), 96% ee.

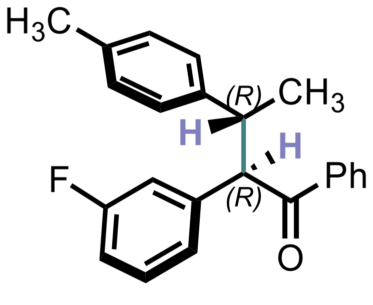

(2R,3R)-2-(3-fluorophenyl)-1-phenyl-3-(p-tolyl)butan-1-one (2n)

This compound was prepared according to the general procedure for C-H insertion using 1-ethyl-4-methylbenzene (601 mg, 698 µL, 10 equiv, 5.00 mmol) as the substrate and 2-diazo-2-(3-fluorophenyl)-1-phenylethan-1-one (120 mg, 1 equiv, 0.500 mmol) under the catalyst of Rh_2(*S*-TPPTTL)$_4$ (6.16 mg, 0.005 equiv, 0.5 mol%, 2.50 µmol). After flash chromatography (3% ether in hexanes), the product was obtained as an amorphous white solid (64 mg, 39% yield, >20:1 r.r., >20:1 d.r., 94% ee).

^{1}H NMR (600 MHz, $CDCl_3$) δ 8.03 – 7.99 (m, 2H), 7.53 (ddt, J = 7.8, 6.9, 1.3 Hz, 1H), 7.47 – 7.42 (m, 2H), 7.02 (td, J = 8.1, 6.2 Hz, 1H), 6.97 – 6.89 (m, 4H), 6.88 – 6.83 (m, 2H), 6.72 (tdd, J = 8.4, 2.4, 1.2 Hz, 1H), 4.70 (d, J = 10.8 Hz, 1H), 3.64 (dq, J = 10.7, 6.8 Hz, 1H), 2.23 (s, 3H), 1.34 (d, J = 6.8 Hz, 3H).

^{13}C NMR (151 MHz, $CDCl_3$) δ 199.78, 163.43, 161.80, 140.78, 140.40, 140.35, 137.44, 135.66, 133.19, 129.69, 129.63, 128.82, 128.69, 128.54, 127.50, 124.62, 124.60, 115.69, 115.54, 113.81, 113.67, 60.53, 43.70, 21.03, 20.98.

^{19}F NMR (565 MHz, $CDCl_3$) δ -113.1 to -113.2 (q, *J* = 9.2, 8.7 Hz).

IR (neat): 3023, 2963, 2925, 2872, 1679, 1610, 1588, 1515, 1485, 1446, 1375, 1348, 1300, 1285, 1266, 1254, 1199, 1180, 1160, 1136, 1117, 1074, 994, 957, 928, 853, 815, 781, 752, 729, 699, 690, 654, 569, 544, 521, 486, 466, 430, 423, 416 cm^{-1}.

HRMS (-p APCI): calc. mass for $C_{23}H_{20}OF$ [M – H]$^-$ 331.1503; obs. mass for $C_{23}H_{20}OF$ [M – H]$^-$ 331.1500.

[α]^{20}$_D$: -62.7° (c 0.683 g/100 mL, EtOAc).

HPLC (Chiralcel OD column, 1.0% *i*-propanol in hexane, 0.5 mL min^{-1}, 0.5 mg mL^{-1}, 30 min, UV 230 nm) retention times of 10.8 min (minor) and 13.8 min (major), 94% ee.

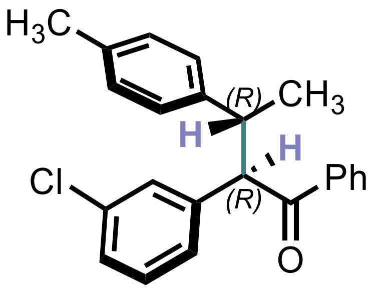

(2R,3R)-2-(3-chlorophenyl)-1-phenyl-3-(p-tolyl)butan-1-one (2o)

This compound was prepared according to the general procedure for C-H insertion using 1-ethyl-4-methylbenzene (601 mg, 698 µL, 10 equiv, 5.00 mmol) as the substrate and 2-(3-chlorophenyl)-2-diazo-1-phenylethan-1-one (128 mg, 1 equiv, 0.500 mmol) under the catalyst of Rh_2(*S*-TPPTTL)$_4$ (6.16 mg, 0.005 equiv, 0.5 mol%, 2.50 µmol). After flash chromatography (3% ether in hexanes), the product was obtained as an amorphous off-white solid (96 mg, 55% yield, >20:1 r.r., >20:1 d.r., 94% ee).

^{1}H NMR (600 MHz, $CDCl_3$) δ 8.02 – 7.98 (m, 2H), 7.54 (ddt, J = 8.6, 6.9, 1.3 Hz, 1H), 7.47 – 7.42 (m, 2H), 7.14 – 7.11 (m, 1H), 7.02 – 6.94 (m, 5H), 6.91 (d, J = 8.1 Hz, 2H), 4.68 (d, J = 10.7 Hz, 1H), 3.63 (dq, J = 10.7, 6.7 Hz, 1H), 2.24 (s, 3H), 1.34 (d, J = 6.8 Hz, 3H).

^{13}C NMR (151 MHz, $CDCl_3$) δ 199.7, 140.7, 139.9, 137.4, 135.7, 134.0, 133.2, 129.5, 128.8, 128.7, 128.7, 128.5, 127.5, 127.1, 127.0, 60.4, 43.7, 20.1, 20.1.

IR (neat): 3022, 2963, 2924, 1677, 1593, 1571, 1514, 1473, 1447, 1429, 1375, 1347, 1286, 1265, 1202, 1179, 1079, 1001, 993, 921, 856, 813, 780, 721, 688, 675, 654, 642, 602, 540, 450, 423, 412 cm^{-1}.

HRMS (-p APCI): calc. mass for $C_{23}H_{20}OCl$ [M – H]$^-$ 347.1208; obs. mass for $C_{23}H_{20}OCl$ [M – H]$^-$ 347.1199.

[α]$^{20}_{D}$: -105.9° (c 1.10 g/100 mL, EtOAc).

HPLC (Chiralcel OD column, 1.0% *i*-propanol in hexane, 0.5 mL min^{-1}, 0.5 mg mL^{-1}, 30 min, UV 280 nm) retention times of 10.0 min (minor) and 12.8 min (major), 94% ee.

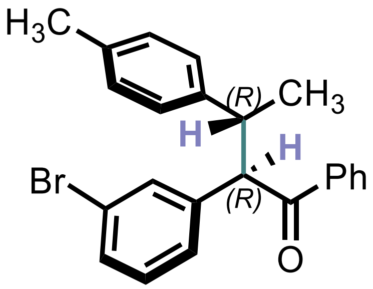

(2R,3R)-2-(3-bromophenyl)-1-phenyl-3-(p-tolyl)butan-1-one (2p)

This compound was prepared according to the general procedure for C-H insertion using 1-ethyl-4-methylbenzene (601 mg, 698 μL, 10 equiv, 5.00 mmol) as the substrate and 2-(3-bromophenyl)-2-diazo-1-phenylethan-1-one (151 mg, 1 equiv, 0.500 mmol) under the catalyst of Rh_2(*S*-TPPTTL)$_4$ (6.16 mg, 0.005 equiv, 0.5 mol%, 2.50 μmol). After flash chromatography (3% ether in hexanes), the product was obtained as an amorphous white solid (78 mg, 40% yield, >20:1 r.r., >20:1 d.r., 94% ee).

^{1}H NMR (600 MHz, $CDCl_3$) δ 8.03 – 7.97 (m, 2H), 7.54 (ddt, J = 7.8, 6.9, 1.3 Hz, 1H), 7.48 – 7.42 (m, 2H), 7.27 (t, J = 1.9 Hz, 1H), 7.16 (ddd, J = 7.9, 2.0, 1.1 Hz, 1H), 7.03 – 7.00 (m, 1H), 6.97 – 6.89 (m, 5H), 4.66 (d, J = 10.7 Hz, 1H), 3.62 (dq, J = 10.7, 6.7 Hz, 1H), 2.24 (s, 3H), 1.34 (d, J = 6.7 Hz, 3H).

^{13}C NMR (151 MHz, $CDCl_3$) δ 199.7, 140.7, 140.2, 137.4, 135.7, 133.2, 131.6, 129.9, 129.8, 128.8, 128.7, 128.6, 127.5, 122.3, 60.4, 43.8, 21.0, 20.9.

IR (neat): 2963, 2924, 2872, 1678, 1594, 1579, 1566, 1515, 1471, 1447, 1425, 1375, 1348, 1286, 1265, 1202, 1179, 1073, 1001, 919, 853, 813, 777, 721, 712, 692, 664, 654, 599, 538, 485, 442, 431, 412 cm^{-1}.

HRMS (-p APCI): calc. mass for $C_{23}H_{20}O^{79}Br$ [M–H]$^-$ 391.0703; obs. mass for $C_{23}H_{20}O^{79}Br$ [M–H]$^-$ 391.0701.

[α]$^{20}_{D}$: -125.4° (c 0.589 g/100 mL, EtOAc).

HPLC (Chiralcel OD column, 1.0% *i*-propanol in hexane, 0.5 mL min^{-1}, 0.5 mg mL^{-1}, 30 min, UV 230 nm) retention times of 10.6 min (minor) and 13.4 min (major), 94% ee.

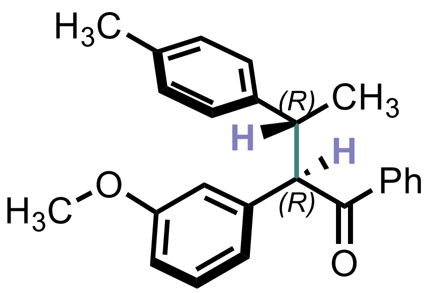

(2R,3R)-2-(3-methoxyphenyl)-1-phenyl-3-(p-tolyl)butan-1-one (2q)

This compound was prepared according to the general procedure for C-H insertion using 1-ethyl-4-methylbenzene (601 mg, 698 μL, 10 equiv, 5.00 mmol) as the substrate and 2-diazo-2-(3-methoxyphenyl)-1-phenylethan-1- one (126 mg, 1 equiv, 0.500 mmol) under the catalyst of Rh_2(*S*-TPPTTL)$_4$ (6.16 mg, 0.005 equiv, 0.5 mol%, 2.50 μmol). After flash chromatography (3% ether in hexanes), the product was obtained as an amorphous light-tan solid (89 mg, 52% yield, >20:1 r.r., >20:1 d.r., 98% ee).

^{1}H NMR (600 MHz, $CDCl_3$) δ 8.03 – 8.00 (m, 2H), 7.51 (ddt, *J* = 7.8, 6.9, 1.3 Hz, 1H), 7.44 – 7.40 (m, 2H), 6.98 (t, *J* = 7.9 Hz, 1H), 6.95 – 6.91 (m, 5H), 6.69 – 6.67 (m, 1H), 6.64 (dd, *J* = 2.7, 1.6 Hz, 1H), 4.67 (d, *J* = 10.7 Hz, 1H), 3.68 – 3.62 (m, 5H), 2.23 (s, 2H), 1.34 (d, *J* = 6.8 Hz, 3H).

^{13}C NMR (151 MHz, $CDCl_3$) δ 199.72, 140.69, 139.92, 137.40, 135.72, 134.04, 133.24, 129.49, 128.85, 128.74, 128.72, 128.55, 127.52, 127.09, 127.00, 60.43, 43.73, 21.05, 20.99, 20.98.

IR (neat): 2961, 2834, 1678, 1597, 1582, 1515, 1486, 1447, 1436, 1348, 1316, 1286, 1264, 1200, 1180, 1146, 1049, 1002, 992, 851, 815, 778, 749, 728, 692, 655, 545, 465, 446, 437, 429, 411 cm^{-1}.

MS (-p APCI): calc. mass for $C_{24}H_{23}O_2$ [M–H]$^-$ 343.1703; obs. mass for $C_{24}H_{23}O_2$ [M–H]$^-$ 343.1699.

[α]$^{20}_D$: -149.1° (c 0.801 g/100 mL, EtOAc).

HPLC (Chiralcel OD column, 1.0% *i*-propanol in hexane, 0.5 mL min^{-1}, 0.5 mg mL^{-1}, 30 min, UV 230 nm) retention times of 12.0 min (minor) and 14.1 min (major), 98% ee.

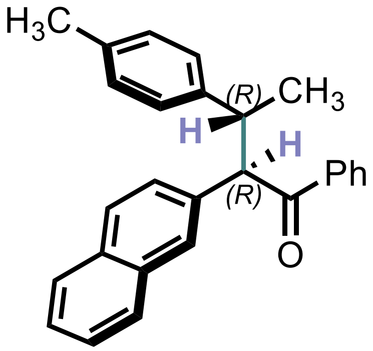

(2R,3R)-2-(naphthalen-2-yl)-1-phenyl-3-(p-tolyl)butan-1-one (2r)

This compound was prepared according to the general procedure for C-H insertion using 1-ethyl-4-methylbenzene (601 mg, 698 µL, 10 equiv, 5.00 mmol) as the substrate and 2-diazo-2-(naphthalen-2-yl)-1-phenylethan-1-one (136 mg, 1 equiv, 0.500 mmol) under the catalyst of Rh_2(*S*-TPPTTL)$_4$ (6.16 mg, 0.005 equiv, 0.5 mol%, 2.50 µmol). After flash chromatography (3% ether in hexanes), the product was obtained as an amorphous light-yellow solid (86 mg, 48% yield, >20:1 r.r., >20:1 d.r., 98% ee).

^{1}H NMR (600 MHz, $CDCl_3$) δ 8.05 – 8.03 (m, 2H), 7.67 – 7.65 (m, 2H), 7.59 – 7.55 (m, 2H), 7.51 – 7.47 (m, 1H), 7.41 (dd, J = 8.3, 7.2 Hz, 2H), 7.38 – 7.33 (m, 2H), 7.24 (dd, J = 8.5, 1.8 Hz, 1H), 6.98 – 6.85 (m, 4H), 4.89 (d, J = 10.7 Hz, 1H), 3.79 (dq, J = 10.7, 6.7 Hz, 1H), 2.16 (s, 3H), 1.40 (d, J = 6.7 Hz, 3H).

^{13}C NMR (151 MHz, $CDCl_3$) δ 200.1, 141.1, 137.6, 135.4, 135.4, 133.3, 133.0, 132.2, 128.8, 128.6, 128.0, 127.8, 127.6, 127.5, 126.9, 125.7, 125.6, 60.9, 43.6, 21.3, 20.9.

IR (neat): 3055, 2963, 2925, 1677, 1629, 1596, 1579, 1514, 1447, 1350, 1289, 1269, 1201, 1179, 1125, 1019, 1001, 960, 906, 850, 813, 745, 689, 562, 526, 477, 430, 415 cm^{-1}.

HRMS (-p APCI): calc. mass for $C_{27}H_{23}O$ $[M – H]^-$ 363.1746; obs. mass for $C_{27}H_{23}O$ $[M – H]^-$ 363.1754.

$[\alpha]^{20}_D$: -185.9° (c 0.457 g/100 mL, EtOAc).

HPLC: (Chiralcel OD column, 1.0% *i*-propanol in hexane, 0.5 mL min^{-1}, 0.5 mg mL^{-1}, 30 min, UV 230 nm) retention times of 11.6 min (minor) and 15.0 min (major), 98% ee.

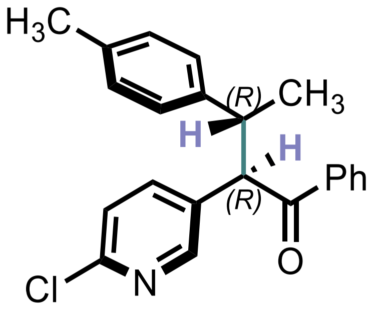

(2R,3R)-2-(6-chloropyridin-3-yl)-1-phenyl-3-(p-tolyl)butan-1-one (2s)

This compound was prepared according to the general procedure for C-H insertion using 1-ethyl-4-methylbenzene (601 mg, 698 µL, 10 equiv, 5.00 mmol) as the substrate and 2-(6-chloropyridin-3-yl)-2-diazo-1-phenylethan-1- one (129 mg, 1 equiv, 0.500 mmol) under the catalyst of Rh_2(*S*-TPPTTL)$_4$ (6.16 mg, 0.005 equiv, 0.5 mol%, 2.50 µmol). After flash chromatography (3% ether in hexanes), the product was obtained as an amorphous off-white solid (115 mg, 66% yield, >20:1 r.r., >20:1 d.r., >99% ee).

^{1}H NMR (600 MHz, $CDCl_3$) δ 8.01 – 7.98 (m, 3H), 7.60 – 7.54 (m, 2H), 7.49 – 7.45 (m, 2H), 7.09 (dd, J = 8.4, 0.7 Hz, 1H), 6.98 – 6.96 (m, 2H), 6.90 (d, J = 8.1 Hz, 2H), 4.75 (d, J = 10.7 Hz, 1H), 3.61 (dq, J = 10.7, 6.8 Hz, 1H), 2.24 (s, 3H), 1.35 (d, J = 6.8 Hz, 3H).

^{13}C NMR (151 MHz, $CDCl_3$) δ 199.50, 150.09, 149.91, 139.96, 138.39, 137.00, 136.26, 133.66, 132.76, 129.24, 128.91, 128.50, 127.41, 124.03, 56.93, 43.89, 21.09, 21.00.

IR (neat): 2965, 2924, 2872, 1717, 1678, 1595, 1580, 1563, 1515, 1457, 1386, 1346, 1291, 1259, 1205, 1179, 1139, 1105, 1023, 1000, 909, 815, 742, 729, 688, 659, 631, 562, 525, 485, 458, 415 cm^{-1}.

HRMS (+p APCI): calc. mass for $C_{22}H_{21}ON^{35}Cl$ [M + H]$^+$ 350.1306; obs. mass for $C_{22}H_{21}ON^{35}Cl$ [M + H]$^+$ 350.1303.

$[\alpha]^{20}_D$: -84.5° (c 0.832 g/100 mL, EtOAc).

HPLC (Chiralpak AS-H column, 1.0% *i*-propanol in hexane, 1.0 mL min^{-1}, 0.5 mg mL^{-1}, 30 min, UV 210 nm) retention times of 12.7 min (major) and 24.6 min (minor), >99% ee.

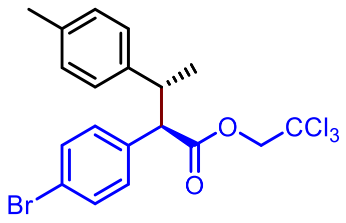

2,2,2-trichloroethyl (2R,3R)-2-(4-bromophenyl)-3-(p-tolyl)butanoate (5)

This compound was prepared according to the general procedure for C-H insertion 1-ethyl-4-methylbenzene (0.18 g, 0.21 mL, 5 equiv, 0.861 g/mL, 1.5 mmol) as the substrate and 2,2,2-trichloroethyl 2-(4-bromophenyl)-2-diazoacetate (0.11 g, 1 equiv, 0.30 mmol) under the catalyst of Rh_2(*S*-TPPTTL)$_4$ (3.7 mg, 0.005 equiv, 0.5 mol%, 1.5 μmol). From crude ^{1}H NMR analysis, diastereomeric mixture was determined by measuring doublet 4.72 – 4.69 ppm (major) and doublet 4.37 – 4.34 ppm (minor). After flash chromatography (hexanes/ether), the product was obtained as a mixture of diastereomer as a colorless oil (120.2 mg, 86% yield, >20:1 r.r., 14:1 d.r., 86% ee). The ^{1}H NMR spectrum is in good agreement with the corresponding literature precedent.[27]

^{1}H NMR (400 MHz, $CDCl_3$) δ 7.27 (d, J = 8.2 Hz, 2H), 7.07 (d, J = 8.5 Hz, 2H), 6.95 (d, J = 7.9 Hz, 2H), 6.89 (d, J = 8.2 Hz, 2H), 4.83 (d, J = 12.0 Hz, 1H), 4.68 (d, J = 12.0 Hz, 1H), 3.81 (d, J = 11.3 Hz, 1H), 3.46 (dq, J = 11.2, 6.8 Hz, 1H), 2.23 (s, 3H), 1.42 (d, J = 6.8 Hz, 3H).

SFC: (ChiralCel OJ-3, 1% of solvent blend (1:1 methanol:*i*-propanol with 0.2% formic acid), 2.5 mL min^{-1}, 10 min, UV 210 nm) retention times of 2.6 min (minor) and 3.2 min (major), 86% ee.

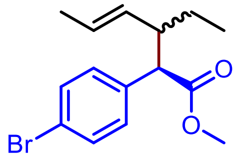

methyl (2R,E)-2-(4-bromophenyl)-3-ethylhex-4-enoate (10)

This compound was prepared according to the general procedure for C-H insertion using (E)-hex-2-ene (0.13 g, 0.19 mL, 5 equiv, 0.6733 g/mL, 1.5 mmol) as the substrate and methyl 2-(4-bromophenyl)-2-diazoacetate (77 mg, 1 equiv, 0.30 mmol) under the catalyst of Rh_2(*S*-TPPTTL)$_4$ (3.7 mg, 0.005 equiv, 0.5 mol%, 1.5 μmol). After flash chromatography (hexanes/ether), the product was obtained as a mixture of diastereomer as a colorless oil (68.8 mg, 74% yield, >20:1 r.r. 5:1 d.r., 20% ee (major), 24% ee (minor)). Diastereomeric mixture was determined by measuring singlet 3.7 ppm and singlet 3.6 ppm. The ^{1}H NMR spectrum is in good agreement with the corresponding literature precedent.[6]

^{1}H NMR (400 MHz, $CDCl_3$) δ (Major diastereomer) 7.39 (d, J = 8.5 Hz, 2H), 7.13 (d, J = 8.4 Hz, 2H), 5.18 (dd, J = 15.2, 6.4 Hz, 1H), 4.85 (ddd, J = 15.2, 9.3, 1.7 Hz, 1H), 3.65 (s, 3H), 3.38 (d, J = 10.4 Hz, 1H), 2.56 (qd, J = 9.9, 3.4 Hz, 1H), 1.67 (dd, J = 6.4, 1.7 Hz, 1H), 1.47 (dd, J = 6.4, 1.6 Hz, 3H), 1.34 – 1.11 (m, 1H), 0.87 (t, J = 7.3 Hz, 3H).

^{13}C NMR (101 MHz, $CDCl_3$) δ (Major diastereomer) 173.85, 136.87, 131.59, 131.27, 130.75, 130.65, 130.46, 128.24, 120.99, 56.48, 51.98, 47.90, 26.47, 17.89, 11.70.

SFC: (Regis (S,S) Whelk-O1, 1% of solvent blend (1:1 methanol:*i*-propanol with 0.2% formic acid), 2.5 mL min^{-1}, 10min, UV 210 nm) retention times of major diastereomer 1.8 min (minor) and 2.2 min (major), 20% ee; retention times of minor diastereomer 1.9 min (minor) and 2.5 min (major), 24% ee.

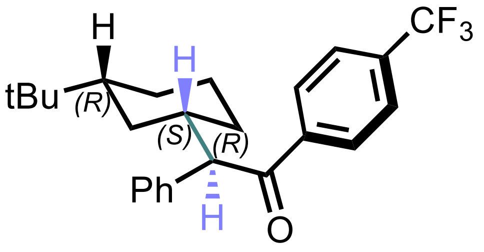

(R)-2-((1S,3R)-3-(tert-butyl)cyclohexyl)-2-phenyl-1-(4-(trifluoromethyl)phenyl)ethan-1-one (11)

This compound was prepared according to the general procedure for C-H insertion using *tert*-butylcyclohexane (210 mg, 5 equiv, 1.5 mmol) as the substrate and 2-diazo-2-phenyl-1-(4-(trifluoromethyl)phenyl)ethan-1-one (87 mg, 1 equiv, 0.3 mmol) under the catalyst of Rh_2(*S*-TPPTTL)$_4$ (4 mg, 0.005 equiv, 0.5 mol%, 1.7 μmol). After flash chromatography (3% ether in hexanes), the product was obtained as an amorphous white solid (62 mg, 51% yield, >20:1 r.r., >20:1 d.r., >99% ee).

Rf = 0.33 (10% diethyl ether/hexanes)

$[\alpha]^{20}_D$: -20.5° (c = 0.4, $CHCl_3$).

^{1}H NMR (400 MHz, $CDCl_3$) δ 8.05 (d, *J* = 8.1 Hz, 2H), 7.66 (d, *J* = 8.0 Hz, 2H), 7.30 – 7.28 (m, 4H), 7.24 – 7.18 (m, 1H), 4.27 (d, *J* = 9.9 Hz, 1H), 2.19 (tdt, *J* = 11.7, 9.8, 3.2 Hz, 1H), 1.86 – 1.69 (m, 3H), 1.44 – 1.23 (m, 2H), 0.96 (qt, *J* = 12.3, 3.3 Hz, 1H), 0.90 – 0.80 (m, 2H), 0.71 (s, 9H), 0.56 (q, *J* = 12.0 Hz, 1H).

^{13}C NMR (101 MHz, $CDCl_3$) δ 199.8, 140.5, 137.2, 134.0 (q, *J* = 32.6 Hz), 128.8 (d, *J* = 6.6 Hz), 127.3, 125.6 (q, *J* = 3.7 Hz), 123.6 (q, *J* = 272.7 Hz), 60.9, 47.7, 41.5, 32.5 (d, *J* = 5.1 Hz), 31.5, 27.4, 27.2, 26.4.

^{19}F NMR (565 MHz, $CDCl_3$) δ -63.2.

IR (neat) 2925, 2854, 2360, 2342, 1688, 1322, 1170, 1133, 1067, 748, 702, 418, cm^{-1}

HRMS (+p APCI) calc. mass for $C_{25}H_{30}F_3O$ $[M + H]^+$ 403.2249; obs. mass for $C_{25}H_{30}F_3O$ $[M + H]^+$ 403.2237.

HPLC (Chiralpak RRW column, 0.5% *i*-propanol in hexane, 0.25 mL min^{-1}, 30 min, UV 254 nm) retention time of 19.4 min (major) and 21.4 min (minor), >99% ee.

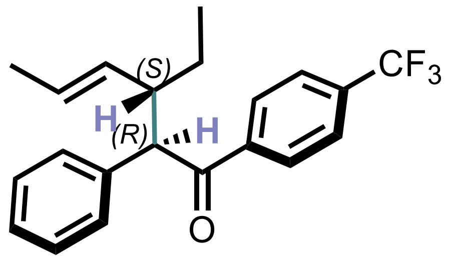

(2R,3S,E)-3-ethyl-2-phenyl-1-(4-(trifluoromethyl)phenyl)hex-4-en-1-one (12)

This compound was prepared according to the general procedure for C-H insertion using *trans*-2-hexene (0.31 ml, 5 equiv, 2.50 mmol) as the substrate and 2-diazo-2-phenyl-1-(4-(trifluoromethyl) -phenyl)ethan-1-one (160 mg, 1 equiv, 0.5 mmol) under the catalyst of Rh_2(*S*-TPPTTL)$_4$ (6.16 mg, 0.005 equiv, 0.5 mol%, 2.50 µmol). After flash chromatography (3% ether in hexanes), the product was obtained as a clear oil (139 mg, 80% yield, >20:1 r.r., >20:1 d.r., >99% ee).

Rf = 0.822 (5% diethyl ether/hexanes)

$[\alpha]^{20}_D$: -75.0° (c 1.996 g/100 mL, EtOAc).

^{1}H NMR (600 MHz, $CDCl_3$) δ 8.05 (d, *J* = 8.2 Hz, 2H), 7.66 (d, *J* = 8.3 Hz, 2H), 7.25 – 7.20 (m, 4H), 7.17 (td, *J* = 6.9, 1.6 Hz, 1H), 5.19 – 5.10 (m, 1H), 4.98 (ddd, *J* = 15.2, 9.4, 1.8 Hz, 1H), 4.43 (d, *J* = 9.9 Hz, 1H), 2.84 (qd, *J* = 9.6, 3.1 Hz, 1H), 1.53 (ddddd, *J* = 14.7, 11.5, 8.4, 5.3, 1.4 Hz, 1H), 1.44 (dd, *J* = 6.4, 1.6 Hz, 3H), 1.28 – 1.21 (m, 1H) 0.87 (t, *J* = 7.4 Hz, 3H).

^{13}C NMR (151 MHz, $CDCl_3$) δ 199.3, 140.3, 137.5, 134.0 (q, *J* = 32.6 Hz), 131.4, 129.1, 128.8, 128.56, 127.6, 127.0, 125.6 (q, *J* = 3.7 Hz), 123.6 (q, *J* = 272.8 Hz), 59.1, 48.1, 26.8, 17.8, 11.9.

^{19}F NMR (565 MHz, $CDCl_3$) δ -63.2.

IR (neat) 2964, 2933, 2360, 2342, 1686, 1322, 1170, 1132, 1067, 747, 410, cm^{-1}.

HRMS (+p APCI) calc. mass for $C_{21}H_{22}F_3O$ [M + H]$^+$ 347.1623; obs. mass for $C_{21}H_{22}F_3O$ [M + H]$^+$ 347.1609.

HPLC (Chiralpak RRW column, 1.0% *i*-propanol in hexane, 1.0 mL min^{-1}, 30 min, UV 230 nm) retention times of 5.8 min (major) and 6.7 min (minor), >99% ee.

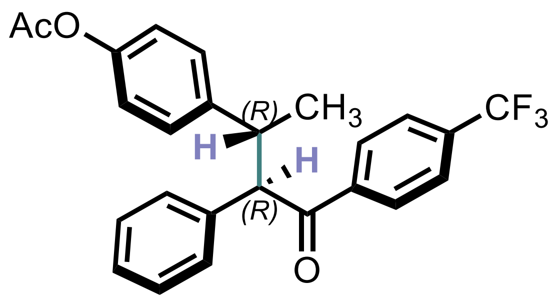

4-((2R,3R)-4-oxo-3-phenyl-4-(4-(trifluoromethyl)phenyl)butan-2-yl)phenyl acetate (13)

This compound was prepared according to the general procedure for C-H insertion using 4-ethylphenyl acetate (0.33 ml, 5 equiv, 2.07 mmol) as the substrate and 2-diazo-2-phenyl-1-(4-(trifluoromethyl)phenyl)ethan-1-one (120 mg, 1 equiv, 0.41 mmol) under the catalyst of Rh_2(*S*-TPPTTL)$_4$ (5 mg, 0.005 equiv, 0.5 mol%, 2.0 μmol). After flash chromatography (5% ether in hexanes), the product was obtained as a yellow oil (103 mg, 59% yield, >20:1 d.r., 99% ee).

Rf = 0.24 (20% diethyl ether/hexanes)

$[\alpha]^{20}_D$: -68.8^o (c = 1.24, $CHCl_3$).

^{1}H NMR (400 MHz, $CDCl_3$) δ 8.07 (d, J = 8.7 Hz, 2H), 7.67 (d, J = 8.1 Hz, 2H), 7.11 – 7.01 (m, J = 1.9, 2.0, 0.9, 1.6 Hz, 5H), 6.99 – 6.83 (ddd, J = 2.0, 2.1 Hz, 4H), 4.60 (d, J = 10.5 Hz, 1H), 3.69 (dq, J = 10.6, 6.7 Hz, 1H), 2.24 (s, 3H), 1.38 (d, J = 6.8 Hz, 3H).

^{13}C NMR (101 MHz, $CDCl_3$) δ 198.7, 169.4, 148.9, 141.3, 140.0 (d, *J* = 1.4 Hz), 136.8, 134.2 (q, *J* = 32.7 Hz), 128.8, 128.7, 128.6 (d, *J* = 2.2 Hz), 127.2, 125.6 (q, *J* = 3.7 Hz), 123.5 (q, *J* = 272.8 Hz), 120.9, 61.8, 43.3, 21.1, 20.6.

^{19}F NMR (376 MHz, $CDCl_3$) δ -63.2.

IR (neat) 2931, 1763, 1684, 1582, 1507, 1454, 1427, 1409, 1368, 1266, 1203, 1166, 1127 1110, 1065, 1014, 996, 940, 910, 870, 844, 821, 775, 737, 700, 633, 596, 553, 506, 448, 436, 426, 413, 403 cm^{-1}.

HRMS (-p APCI): calc. mass for $C_{25}H_{20}F_3O_3$ [M - H]$^-$ 425.1359; obs. mass for $C_{25}H_{20}F_3O_3$ [M - H]$^-$ 425.1365.

HPLC (Chiralpak RRW column, 2% *i*-propanol in hexane, 1 mL min^{-1}, 40 min, UV 230 nm) retention times of 26.8 min (major) and 32.1 min (minor), 99% ee.

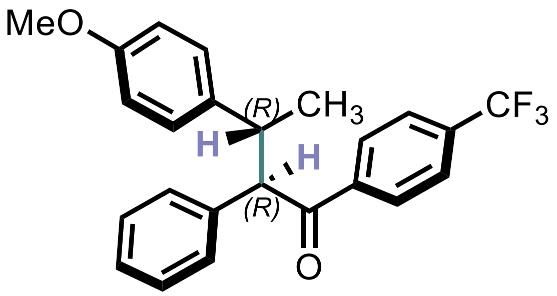

(2*R*,3*R*)-3-(4-methoxyphenyl)-2-phenyl-1-(4-(trifluoromethyl)phenyl)butan-1-one (14)

This compound was prepared according to the general procedure for C-H insertion using 1-ethyl-4-methoxybenzene (0.355 ml, 5 equiv, 2.50 mmol) as the substrate and 2-diazo-2-phenyl-1-(4-(trifluoromethyl)phenyl)ethan-1-one (145 mg, 1 equiv, 0.500 mmol) under the catalyst of Rh_2(*S*-TPPTTL)$_4$ (6.16 mg, 0.005 equiv, 0.5 mol%, 2.50 μmol). After flash chromatography (3% ether in hexanes), the product was obtained as an amorphous white powder (142 mg, 72% yield, >20:1 r.r., >20:1 d.r., 92% ee).

Rf = 0.324 (10% diethyl ether/hexanes)

$[\alpha]^{20}_D$: -142.2° (c = 1.04, $CHCl_3$).

^{1}H NMR (600 MHz, $CDCl_3$) δ 8.08 (d, J = 8.1 Hz, 2H), 7.67 (d, J = 8.1 Hz, 2H), 7.11 – 7.04 (m, 5H), 6.91 (d, J = 8.4 Hz, 2H), 6.68 – 6.64 (m, 2H), 4.61 (d, J = 10.5 Hz, 1H), 3.71 (s, 3H), 3.64 (dq, J = 10.8, 6.9 Hz, 1H), 1.36 (dd, J = 6.7, 0.9 Hz, 3H).

^{13}C NMR (151 MHz, $CDCl_3$) δ 199.1, 157.8, 140.2, 137.2, 135.9, 134.3, 134.0, 128.8, 128.8, 128.6, 128.5, 127.0, 125.6 (q, *J* = 3.8 Hz), 124.4, 122.6, 113.4, 61.9, 55.1, 43.0, 20.9.

^{19}F NMR (565 MHz, $CDCl_3$) δ -63.2.

IR (neat) 2990, 1677, 1607, 1581, 1512, 1454, 1410, 1325, 1267, 1246, 1165, 1134, 1109, 1067, 1034, 995, 869, 855, 824, 760, 747, 706, 695, 586, 558, 544, 527, 413, 403 cm^{-1}.

HRMS (–p APCI) calc. mass for $C_{24}H_{20}F_3O_2$ [M – H]$^-$ 397.1494; obs. mass for $C_{24}H_{20}F_3O_2$ [M – H]$^-$ 397.1421.

HPLC (Regis (S,S) Whelk-O1, 1% *i*-propanol in hexane, 2 mL min^{-1}, 30 min, UV 230 nm) retention times of 9.5 min (minor) and 12.3 min (major), 92% ee**.**

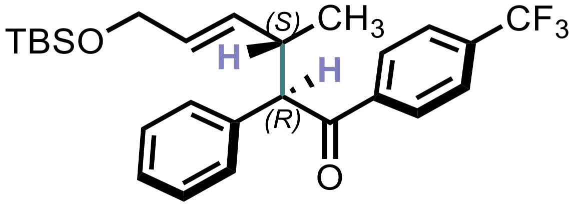

(2R,3S,E)-6-((tert-butyldimethylsilyl)oxy)-3-methyl-2-phenyl-1-(4-(trifluoromethyl)phenyl)hex-4-en-1-one (15)

This compound was prepared according to the general procedure for C-H insertion using (*E*)-*tert*-butyldimethyl(pent-2-en-1-yloxy)silane (301 mg, 5 equiv, 1.5 mmol) as the substrate and 2-diazo-2-phenyl-1-(4-(trifluoromethyl) -phenyl)ethan-1-one (87 mg, 1 equiv, 0.3 mmol) under the catalyst of Rh_2(*S*-TPPTTL)$_4$ (4 mg, 0.005 equiv, 0.5 mol%, 1.7 µmol). After flash chromatography (2% ether in hexanes), the product was obtained as a clear oil (71 mg, 51% yield, 7:1 r.r., >20:1 d.r., 96% ee).

Rf = 0.5 (10% diethyl ether/hexanes)

$[\alpha]^{20}_D$: -54.5^o (c = 1.0, $CHCl_3$).

^{1}H NMR (600 MHz, $CDCl_3$) δ 8.08 – 8.04 (m, 2H), 7.69 – 7.64 (m, 2H), 7.28 – 7.26 (m, 4H), 7.22 – 7.16 (m, 1H), 5.42 – 5.37 (m, 2H), 4.35 (d, *J* = 10.1 Hz, 1H), 3.97 – 3.95 (m, 2H), 3.19 (dddd, *J* = 12.4, 8.8, 6.6, 5.4 Hz, 1H), 1.13 (d, *J* = 6.5 Hz, 3H), 0.84 (s, 9H), -0.04 (d, *J* = 7.8 Hz, 6H).

^{13}C NMR (151 MHz, $CDCl_3$) δ 198.9, 140.1, 137.2, 134.1 (q, *J* = 32.7 Hz), 132.4, 129.8, 128.9, 128.8 (d, *J* = 2.7 Hz), 127.3, 125.6 (q, *J* = 3.7 Hz), 123.5 (q, *J* = 272.6 Hz), 63.5, 60.3, 39.5, 25.9, 19.1, 18.3, -5.3.

^{19}F NMR (565 MHz, $CDCl_3$) δ -63.2.

IR (neat) 3064, 3030, 2958, 2930, 2885, 2857, 1688, 1583, 1409, 1310, 1256, 1169, 1130, 835 cm^{-1}.

HRMS (+p APCI) calc. mass for $C_{26}H_{34}F_3O_2Si$ $[M + H]^+$ 463.2280; obs. mass for $C_{26}H_{34}F_3O_2Si$ $[M + H]^+$ 463.2274.

HPLC (Chiralpak RRW column, 0.5 % *i*-propanol in hexane, 0.5 mL min^{-1}, 0.5 mg mL^{-1}, 15 min, UV 230 nm) retention times of 9.2 min (major) and 10.5 min (minor), 96% ee.

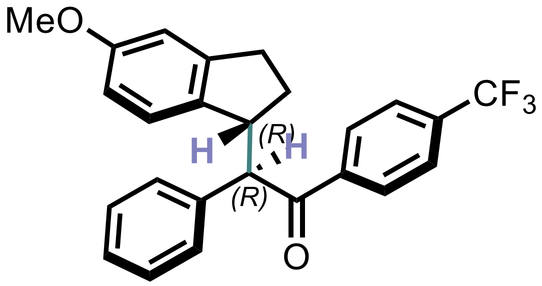

(*R*)-2-((*R*)-5-methoxy-2,3-dihydro-1*H*-inden-1-yl)-2-phenyl-1-(4-(trifluoromethyl) phenyl)ethan-1-one (16)

This compound was prepared according to the general procedure for C-H insertion using 5-methoxy-2,3-dihydro-1H-indene (0.362 ml, 5 equiv, 2.50 mmol) as the substrate and 2-diazo-2-phenyl-1-(4-(trifluoromethyl)phenyl)ethan-1-one (145 mg, 1 equiv, 0.500 mmol) under the catalyst of Rh_2(*S*-TPPTTL)$_4$ (6.16 mg, 0.005 equiv, 0.5 mol%, 2.50 µmol). After flash chromatography (3% ether in hexanes), the product was obtained as an amorphous off-white solid (149 mg, 73% yield, 13:1 r.r., >20:1 d.r., 82% ee).

Rf = 0.175 (10% diethyl ether/hexanes)

$[\alpha]^{20}_D$: -59.4^o (c = 1.05, $CHCl_3$)

^{1}H NMR (400 MHz, $CDCl_3$) δ 8.10 – 8.02 (m, 2H), 7.69 – 7.62 (m, 2H), 7.32 – 7.27 (m, 3H), 7.25 – 7.23 (m, 2H), 6.76 (d, *J* = 2.5 Hz, 1H), 6.42 – 6.34 (m, 1H), 5.90 (d, *J* = 8.4 Hz, 1H), 4.55 (d, *J* = 10.7 Hz, 1H), 4.05 (ddd, *J* = 11.2, 7.9, 3.9 Hz, 1H), 3.73 (s, 3H), 2.95 (dt, *J* = 16.1, 8.1 Hz, 1H), 2.82 (ddd, *J* = 16.1, 8.8, 4.3 Hz, 1H), 2.44 (ddt, *J* = 13.1, 8.9, 8.0 Hz, 1H), 1.85 – 1.72 (m, 1H).

^{13}C NMR (151 MHz, $CDCl_3$) δ 198.9, 159.0, 145.9, 139.8, 137.4, 136.2, 134.1, 129.4, 128.9, 128.9, 127.7, 126.1, 125.7 (q, J = 3.8 Hz), 124.4, 111.4, 109.7, 59.0, 55.3, 47.7, 32.2, 31.0.

^{19}F NMR (376 MHz, $CDCl_3$) δ -63.2.

IR (neat): 2938, 1677, 1605, 1583, 1492, 1454, 1409, 1327, 1246, 1164, 1127, 1108, 1067, 1029, 1015, 996, 922, 873, 858, 816, 773, 740, 705, 694, 589, 515, 470, 448, 435, 426, 413, 403 cm^{-1}.

HRMS (-p APCI) calc. mass for $C_{25}H_{20}F_3O_2$ $[M – H]^-$ 409.1494; obs. mass for $C_{25}H_{20}F_3O_2$ $[M – H]^-$ 409.1419.

HPLC (Chiralpak RRW column, 3.0% *i*-propanol in hexane, 0.1 mL min^{-1}, 0.1 mg mL^{-1}, 90 min, UV 230 nm) retention times of 75.0 min (major) and 80.9 min (minor), 82% ee.

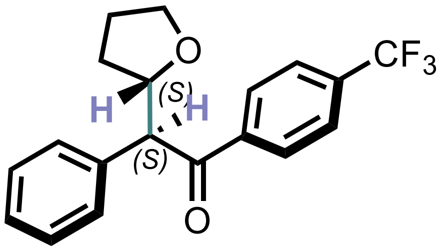

(*S*)-2-phenyl-2-((*S*)-tetrahydrofuran-2-yl)-1-(4-(trifluoromethyl)phenyl)ethan-1-one (17)

This compound was prepared according to the general procedure for C-H insertion using THF (0.9 ml, 5 equiv, 1.5 mmol) as the substrate and 2-diazo-2-phenyl-1-(4-(trifluoromethyl) -phenyl)ethan-1-one (87 mg, 1 equiv, 0.30 mmol) under the catalyst of Rh_2(*S*-TPPTTL)$_4$ (4 mg, 0.005 equiv, 0.5 mol%, 1.7 μmol). After flash chromatography (10% ether in hexanes), the product was obtained as an amorphous clear solid (75 mg, 75% yield, >20:1 r.r., 13:1 d.r., 78% ee).

Rf = 0.1 (10% diethyl ether/hexanes)

$[\alpha]^{20}_D$: -82.5° (c = 1.0, $CHCl_3$)

^{1}H NMR (600 MHz, $CDCl_3$) δ 8.03 (d, *J* = 8.2 Hz, 2H), 7.65 (d, *J* = 8.2 Hz, 2H), 7.40 – 7.35 (m, 2H), 7.35 – 7.30 (m, 2H), 7.29 – 7.18 (m, 1H), 4.59 – 4.51 (m, 2H), 3.85 (dt, *J* = 8.6, 6.8 Hz, 1H), 3.71 (dt, *J* = 8.3, 6.7 Hz, 1H), 2.30 – 2.21 (m, 1H), 1.90 (dtd, *J* = 11.8, 6.2, 4.1 Hz, 2H), 1.56 (tdd, *J* = 12.5, 8.3, 6.2 Hz, 1H).

^{13}C NMR (151 MHz, $CDCl_3$) δ 197.9, 139.3, 134.2 (q, *J* = 32.8 Hz), 130.4, 129.1, 129.0, 128.9, 128.7, 127.7, 125.6 (q, *J* = 3.8 Hz), 123.5 (q, *J* = 272.9 Hz), 81.0, 68.0, 59.6, 30.8, 25.7.

^{19}F NMR (565 MHz, $CDCl_3$) δ -63.2.

IR (neat) 2923, 2854, 2360, 2342, 1687, 1325, 1170, 1129, 1066, 701, 417, cm^{-1}.

HRMS (–p APCI) calc. mass for $C_{19}H_{16}F_3O_2$ $[M – H]^-$ 333.1097; obs. mass for $C_{19}H_{16}F_3O_2$ $[M – H]^-$ 333.1106.

HPLC (Chiralpak OD-H column, 1% *i*-propanol in hexane, 1 mL min^{-1}, 60 min, UV 210 nm) retention times of 8.3 min (minor) and 9.0 min (major), 78% ee.

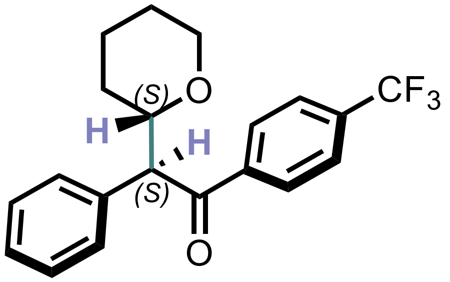

(S)-2-phenyl-2-((S)-tetrahydro-2H-pyran-2-yl)-1-(4-(trifluoromethyl)phenyl)ethan-1-one (18)

This compound was prepared according to the general procedure for C-H insertion using tetrahydropyran (0.245 ml, 5 equiv, 2.50 mmol) as the substrate and 2-diazo-2-phenyl-1-(4-(trifluoromethyl) -phenyl)ethan-1-one (145 mg, 1 equiv, 0.500 mmol) under the catalyst of Rh_2(*S*-TPPTTL)$_4$ (6.16 mg, 0.005 equiv, 0.5 mol%, 2.50 μmol). After flash chromatography (5% ether in hexanes), the product was obtained as a thick clear oil (56 mg, 32% yield, >20:1 r.r., 12:1 d.r, 92% ee).

Rf = 0.158 (10% diethyl ether/hexanes)

$[\alpha]^{20}_D$: -49.5° (c = 1.07, $CHCl_3$).

^{1}H NMR **(600 MHz, CDCl3) δ** 8.05 (d, *J* = 8.2 Hz, 2H), 7.67 (d, *J* = 8.2 Hz, 2H), 7.41 – 7.35 (m, 2H), 7.31 (dd, *J* = 8.4, 6.9 Hz, 2H), 7.26 – 7.22 (m, 1H), 4.65 (d, *J* = 9.1 Hz, 1H), 4.07 (ddd, *J* = 10.8, 9.1, 1.8 Hz, 1H), 3.93 – 3.87 (m, 1H), 3.34 (td, *J* = 11.6, 2.5 Hz, 1H), 1.86 – 1.76 (m, 2H), 1.63 – 1.52 (m, 2H), 1.49 (dtt, *J* = 9.4, 4.4, 2.3 Hz, 1H), 1.34 (tdd, *J* = 12.6, 10.6, 4.3 Hz, 1H).

^{13}C NMR (151 MHz, $CDCl_3$) δ 197.9, 139.8, 136.1, 134.3 (q, J = 32.6 Hz), 128.9 (d, J = 8.3 Hz), 128.8, 127.5, 125.7 (q, J = 3.7 Hz), 123.5 (q, *J* = 272.6 Hz), 79.4, 69.0, 59.7, 30.45, 29.7, 25.9, 23.4.

^{19}FNMR (565 MHz, CDCl3) δ -63.2.

IR (neat): 2937, 2850, 1681, 1581, 1510, 1495, 1453, 1408, 1321, 1295, 1266, 1200, 1166, 1126, 1089, 1065, 1048, 1011, 973, 905, 871, 849, 814, 773, 745, 695, 610, 594, 550, 528, 475, 435, 426, 410, 403 cm^{-1}.

HRMS (+p APCI) calc. mass for $C_{20}H_{20}F_3O_2$ $[M + H]^+$ 349.1337; obs. mass for $C_{20}H_{20}F_3O_2$ $[M + H]^+$ 349.1413.

HPLC: (Chiralpak ODH column, 1.0% *i*-propanol in hexane, 1.0 mL min^{-1}, 1.0 mg mL^{-1}, 30 min, UV 230 nm) retention times of 5.3 min (minor) and 6.4 (major), 92% ee.

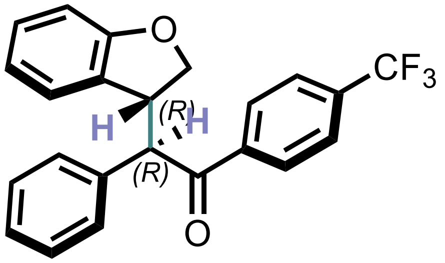

(*R*)-2-((*R*)-2,3-dihydrobenzofuran-3-yl)-2-phenyl-1-(4-(trifluoromethyl)phenyl)ethan-1-one (19)

This compound was prepared according to the general procedure for C-H insertion using 2,3-dihydrobenzofuran (0.22 ml, 5 equiv, 1.9 mmol) as the substrate and 2-diazo-2-phenyl-1-(4-(trifluoromethyl)phenyl)ethan-1-one (110 mg, 1 equiv, 0.38 mmol) under the catalyst of Rh_2(*S*-TPPTTL)$_4$ (5 mg, 0.005 equiv, 0.5 mol%, 2.0 µmol). After reaction completion and solvent removal *in vacuo*, the crude residue was subjected to Kugelrohr distillation (0.5 mmHg, 90 ºC) for one hour to remove excess starting material. After flash chromatography (5% ether in hexanes), the product was obtained as an amorphous yellow solid (43 mg, 30% yield, >20:1 r.r., 3:1 d.r., 96% ee).

Rf = 0.487 (15% diethyl ether/hexanes)

$[\alpha]^{20}_D$: -88.2^o (c = 0.95, $CHCl_3$).

^{1}H NMR (400 MHz, $CDCl_3$) δ 8.04 (dt, *J* = 8.1, 0.9 Hz, 2H), 7.64 (dd, *J* = 8.9, 0.8 Hz, 2H), 7.32 (qt, *J* = 4.9, 2.1 Hz, 3H), 7.20 (dd, *J* = 7.7, 1.9 Hz, 2H), 7.09 (tdd, *J* = 7.4, 1.4, 0.6 Hz, 1H), 6.80 (dd, *J* = 8.1, 1.0 Hz, 1H), 6.56 (td, *J* = 7.5, 1.0 Hz, 1H), 5.87 (dp, *J* = 7.5, 0.7 Hz, 1H), 4.79 (dd, *J* = 9.5, 8.3 Hz, 1H), 4.67 (d, *J* = 10.7 Hz, 1H), 4.28 (ddd, *J* = 10.4, 8.1, 4.0 Hz, 1H), 4.22 (dd, *J* = 9.5, 4.2 Hz, 1H).

^{13}C NMR (101 MHz, $CDCl_3$) δ 198.0, 160.3, 138.8, 136.1, 134.4 (q, *J* = 32.7 Hz), 129.3, 129.2, 129.11, 128.7, 128.1, 127.4, 126.0, 125.7 (q, *J* = 3.8 Hz), 123.4 (q, *J* = 272.8 Hz), 119.8, 109.5, 59.5, 45.2.

^{19}F NMR (376 MHz, $CDCl_3$) δ -63.3.

IR (neat): 2937, 1679, 1593, 1513, 1480, 1455, 1409, 1322, 1294, 1282, 1214, 1167, 1127, 1066, 1006, 997, 971, 922, 836, 774, 748, 734, 702, 605, 518, 504, 468, 436, 426, 413, 403 cm^{-1}.

HRMS (-p APCI) calc. mass for $C_{23}H_{16}F_3O_2$ [M – H]$^-$ 381.1181; obs. mass $C_{23}H_{16}F_3O_2$ [M – H]$^-$ 381.1105.

HPLC (Chiralpak RRW column, 10.0% *i*-propanol in hexane, 0.5 mL min^{-1}, 0.5 mg mL^{-1}, 15 min, UV 254 nm) retention times of 9.3 min (major) and 10.1 min (minor), 96% ee.

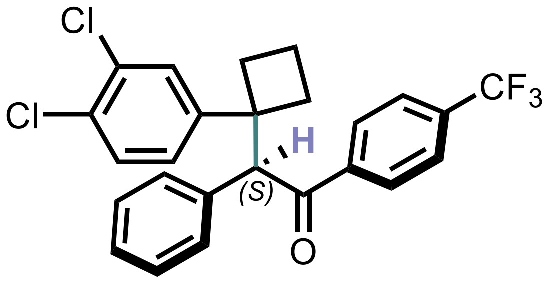

(*S*)-2-(1-(3,4-dichlorophenyl)cyclobutyl)-2-phenyl-1-(4-(trifluoromethyl)phenyl)ethan-1-one (20)

This compound was prepared according to the general procedure for C-H insertion using 1,2-dichloro-4-cyclobutylbenzene (302 mg, 5 equiv, 1.5 mmol) as the substrate and 2-diazo-2-phenyl-1-(4-(trifluoromethyl) -phenyl)ethan-1-one (87 mg, 1 equiv, 0.3 mmol) under the catalyst of Rh_2(*S*-TPPTTL)$_4$ (4 mg, 0.005 equiv, 0.5 mol%, 1.7 μmol). After flash chromatography (3% ether in hexanes), the product was obtained as a clear oil (65 mg, 47% yield, >20:1 r.r., >99% ee).

Rf = 0.54 (10% diethyl ether/hexanes)

$[\alpha]^{20}_D$: -68.5^o (c = 1.2, $CHCl_3$)

^{1}H NMR (400 MHz, $CDCl_3$) δ 7.86 – 7.78 (m, 2H), 7.60 – 7.52 (m, 2H), 7.32 – 7.27 (m, 3H), 7.24 (s, 1H), 7.19 (d, *J* = 2.2 Hz, 1H), 7.17 – 7.13 (m, 2H), 6.99 (dd, *J* = 8.4, 2.2 Hz, 1H), 4.90 (s, 1H), 2.70 (ddd, *J* = 11.5, 9.1, 6.4 Hz, 1H), 2.45 (ddd, *J* = 12.6, 8.5, 7.1 Hz, 3H), 1.81 – 1.54 (m, 2H).

^{13}C NMR (101 MHz, $CDCl_3$) δ 197.9, 147.9, 140.1, 135.0, 133.9 (q, *J* = 32.8 Hz), 131.2, 130.4, 130.0, 129.6, 129.1, 128.7, 128.6, 127.9, 127.8, 125.5 (q, *J* = 3.7 Hz), 123.5 (q, *J* = 272.7 Hz), 62.7, 49.4, 32.8, 31.8, 16.4.

^{19}F NMR (376 MHz, $CDCl_3$) δ -63.2.

IR (neat) 3063, 3027, 2981, 2944, 2867, 1745, 1689, 1470, 1310, 1209, 1168, 1128, 1067, 1014, 817, 712 cm^{-1}.

HRMS (–p APCI) calc. mass for $C_{25}H_{18}F_3OCl_2$ $[M – H]^-$ 461.0765; obs. mass for $C_{25}H_{18}F_3OCl_2$ $[M – H]^-$ 461.0694.

HPLC (Chiralpak RRW column, 1.0% *i*-propanol in hexane, 0.5 mL min^{-1}, 15 min, UV 230 nm) retention times of 10.3 min (major) and 11.9 min (minor), >99% ee.

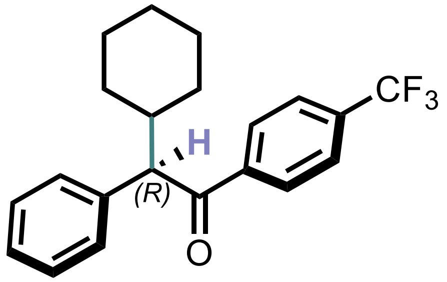

(*R*)-2-cyclohexyl-2-phenyl-1-(4-(trifluoromethyl)phenyl)ethan-1-one (21)

This compound was prepared according to the general procedure for C-H insertion using cyclohexane (126 mg, 5 equiv, 1.5 mmol) as the substrate and 2-diazo-2-phenyl-1-(4-(trifluoromethyl) -phenyl)ethan-1-one (87 mg, 1 equiv, 0.3 mmol) under the catalyst of Rh_2(*S*-TPPTTL)$_4$ (4 mg, 0.005 equiv,

0.5 mol%, 1.7 μmol). After flash chromatography (3% ether in hexanes), the product was obtained as a clear oil (60 mg, 58% yield, 98% ee).

Rf = 0.73 (10% diethyl ether/hexanes)

[α]$^{20}_{D}$: -5.0^{o} (c = 1.0, $CHCl_3$).

^{1}H NMR (600 MHz, $CDCl_3$) δ 8.05 (d, *J* = 8.1 Hz, 2H), 7.66 (d, *J* = 8.2 Hz, 2H), 7.33 – 7.27 (m, 4H), 7.24 – 7.19 (m, 1H), 4.28 (d, *J* = 10.1 Hz, 1H), 2.30 (qt, *J* = 10.9, 3.3 Hz, 1H), 1.83 (dt, *J* = 12.6, 3.1 Hz, 1H), 1.67 (ddt, *J* = 19.3, 12.5, 3.1 Hz, 3H), 1.39 – 1.28 (m, 2H), 1.17 (dddd, *J* = 25.2, 15.2, 12.2, 8.6 Hz, 2H), 0.98 (qd, *J* = 12.4, 3.4 Hz, 1H), 0.91 – 0.81 (m, 1H).

^{13}C NMR (151 MHz, $CDCl_3$) δ 199.7, 140.4, 137.3, 134.0 (q, *J* = 32.7 Hz), 128.9, 128.7, 127.3, 125.6 (q, *J* = 3.7 Hz), 123.6 (q, *J* = 272.6 Hz), 60.7, 41.0, 32.6, 30.6, 26.4, 26.1, 26.1.

^{19}F NMR (565 MHz, $CDCl_3$) δ -63.2.

IR (neat) 2926, 2854, 2360, 2342, 1686, 1323, 1260, 1169, 1132, 1066, 802, 429, cm^{-1}

HRMS (+p APCI) calc. mass for $C_{21}H_{22}F_3O$ [M + H]$^+$ 347.1623; obs. mass for $C_{21}H_{22}F_3O$ [M + H]$^+$ 347.1610.

HPLC (Regis (S,S) Whelk-O1, 0.5% *i*-propanol in hexane, 0.5 mL min^{-1}, 40 min, UV 230 nm) retention times of 13.2 min (minor) and 17.1 min (major), 98% ee.

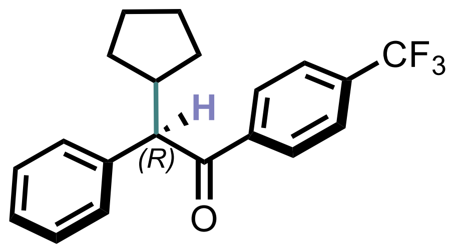

(*R*)-2-cyclopentyl-2-phenyl-1-(4-(trifluoromethyl)phenyl)ethan-1-one (22)

This compound was prepared according to the general procedure for C-H insertion using cyclopentane (0.75 ml, 10 equiv, 3.0 mmol) as the substrate and 2-diazo-2-phenyl-1-(4-(trifluoromethyl) -phenyl)ethan-1-one (87 mg, 1 equiv, 0.3 mmol) under the catalyst of Rh_2(*S*-TPPTTL)$_4$ (4 mg, 0.005 equiv,

0.5 mol%, 1.7 µmol). After flash chromatography (3% ether in hexanes), the product was obtained as a clear oil (40 mg, 40% yield, 99% ee).

Rf = 0.86 (10% diethyl ether/hexanes)

[α]$^{20}_{D}$: -86.9° (c = 1.0, $CHCl_3$).

^{1}H NMR (400 MHz, $CDCl_3$) δ 8.05 (dt, *J* = 8.1, 1.0 Hz, 2H), 7.68 – 7.61 (m, 2H), 7.33 – 7.27 (m, 4H), 7.24 – 7.18 (m, 1H), 4.28 (d, *J* = 10.5 Hz, 1H), 2.74 (dtt, *J* = 10.4, 9.2, 7.2 Hz, 1H), 2.03 – 1.91 (m, 1H), 1.66 – 1.58 (m, 3H), 1.53 – 1.36 (m, 2H), 1.21 – 1.02 (m, 2H).

^{13}C NMR (151 MHz, $CDCl_3$) δ 199.3, 139.9, 138.6, 133.9 (q, *J* = 32.6 Hz), 128.9, 128.8, 128.4, 127.2, 125.6 (q, *J* = 3.8 Hz), 123.6 (q, *J* = 272.6 Hz), 60.2, 43.7, 31.9, 30.9, 25.2, 24.7.

^{19}F NMR (565 MHz, $CDCl_3$) δ -63.2.

IR (neat) 3064, 3027, 2954, 2868, 1685, 1599, 1581, 1510, 1495, 1452, 1408, 1315, 1274, 1209, 1166, 1126, 1111, 1080, 1031, 1015, 1006, 988, 873, 822, 773, 744, 700 cm^{-1}.

HRMS (+p APCI) calc. mass for $C_{20}H_{20}F_3O$ [M + H]$^+$ 333.1388; obs. mass for $C_{20}H_{20}F_3O$ [M + H]$^+$ 333.1465.

HPLC (Chiralpak RRW column, 0.5 % *i*-propanol in hexane, 0.5 mL min^{-1}, 0.5 mg mL^{-1}, 15 min, UV 230 nm) retention times of 9.7 min (major) and 11.5 min (minor), 99% ee.

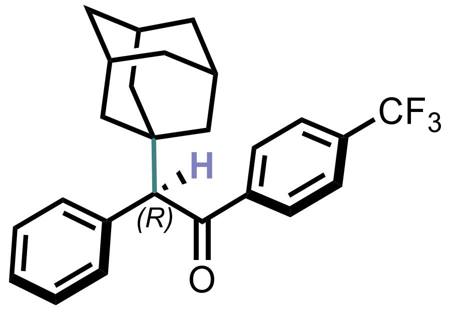

(*R*)-2-((3*S*,5*S*,7*S*)-adamantan-1-yl)-2-phenyl-1-(4-(trifluoromethyl)phenyl)ethan-1-one (23)

This compound was prepared according to the general procedure for C-H insertion using adamantane (341 mg, 5 equiv, 2.50 mmol) as the substrate and 2-diazo-2-phenyl-1-(4-(trifluoromethyl) -

phenyl)ethan-1-one (145 mg, 1 equiv, 0.500 mmol) under the catalyst of Rh_2(*S*-TPPTTL)$_4$ (6.16 mg, 0.005 equiv, 2.50 μmol). After flash chromatography (3% ether in hexanes) the product was obtained as an amorphous off-white solid (117 mg, 59% yield, >20:1 r.r., 94% ee).

Rf = 0.822 (5% diethyl ether/hexanes)

$[\alpha]^{20}_D$: -136.7° (c = 0.98, $CHCl_3$).

^{1}H NMR (400 MHz, $CDCl_3$) δ 7.99 (d, *J* = 8.2 Hz, 2H), 7.64 (d, *J* = 8.2 Hz, 2H), 7.40 – 7.35 (m, 2H), 7.34 – 7.27 (m, 3H), 4.33 (s, 1H), 1.96 (p, *J* = 3.1 Hz, 3H), 1.83 (dq, *J* = 12.0, 2.5 Hz, 3H), 1.70 – 1.56 (m, 9H).

^{13}C NMR (101 MHz, $CDCl_3$) δ 199.9, 141.8, 134.2, 133.7 (q, *J* = 32.7 Hz), 130.5, 128.5, 128.1, 127.3, 125.5 (q, *J* = 3.8 Hz), 123.6 (q, *J* = 272.6 Hz), 64.2, 40.2, 37.6, 36.8, 28.6.

^{19}F NMR (565 MHz, $CDCl_3$) δ -63.1.

IR (neat) 2906, 2849, 2360, 2342, 1738, 1323, 1132, 1067, 668, 418, cm^{-1}.

HRMS (–p APCI) calc. mass for $C_{25}H_{24}F_3O$ $[M – H]^-$ 397.1774; obs. mass for $C_{25}H_{24}F_3O$ $[M – H]^-$ 397.1778.

HPLC (Regis (S,S) Whelk-O1, 0.5% *i*-propanol in hexane, 0.5 mL min^{-1}, 40 min, UV 230 nm) retention times of 13.7 min (minor) and 16.0 min (major), 94% ee.

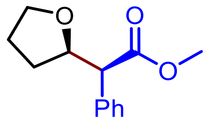

methyl (S)-2-phenyl-2-((R)-tetrahydrofuran-2-yl)acetate (S35)

This compound was prepared according to the general procedure for C-H insertion using tetrahydrofuran (0.11 g, 0.12 mL, 5 equiv, 0.899 g/mL, 1.5 mmol) as the substrate and methyl 2-diazo-2-phenylacetate (53 mg, 1 equiv, 0.30 mmol) under the catalyst of Rh_2(*S*-TPPTTL)$_4$ (3.7 mg, 0.005 equiv, 0.5 mol%, 1.5 μmol). Based on crude ^{1}H NMR, the d.r. is 1:1 cis:trans. After flash chromatography

(hexanes/ether), the single diastereomer was obtained as a colorless oil (28.9 mg, 44% yield, 80% ee). The ^{1}H NMR spectrum is in good agreement with the corresponding literature precedent.[31]

^{1}H NMR (400 MHz, $CDCl_3$) δ 7.45 – 7.27 (m, 5H), 4.46 (dt, J = 8.4, 6.9 Hz, 1H), 3.81 (dt, J = 8.4, 6.8 Hz, 1H), 3.71 (dt, J = 8.4, 6.8 Hz, 1H), 3.67 (s, 3H), 3.63 (d, J = 8.6 Hz, 1H), 2.20 – 2.05 (m, 1H), 1.88 (dqd, J = 7.8, 6.7, 1.7 Hz, 2H), 1.67 (dq, J = 12.2, 7.9 Hz, 1H).

SFC: (ChiralCel OZ-3, 2% of solvent blend (1:1 methanol:*i*-propanol with 0.2% formic acid), 2.5 mL min^{-1}, 15 min, UV 210 nm) retention times of 2.7 min (major) and 3.6 min (minor), 80% ee.

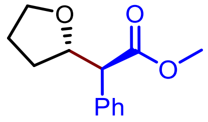

methyl (S)-2-phenyl-2-((S)-tetrahydrofuran-2-yl)acetate (S36)

This compound was prepared according to the general procedure for C-H insertion using tetrahydrofuran (0.11 g, 0.12 mL, 5 equiv, 0.899 g/mL, 1.5 mmol) as the substrate and methyl 2-diazo-2-phenylacetate (53 mg, 1 equiv, 0.30 mmol) under the catalyst of Rh_2(*S*-TPPTTL)$_4$ (3.7 mg, 0.005 equiv, 0.5 mol%, 1.5 μmol). Based on crude ^{1}H NMR, the d.r. is 1:1 cis:trans. After flash chromatography (hexanes/ether), the single diastereomer was obtained as a colorless oil (31.9 mg, 48% yield, 84% ee). The ^{1}H NMR spectrum is in good agreement with the corresponding literature precedent.[31]

^{1}H NMR (400 MHz, $CDCl_3$) δ 7.38 – 7.27 (m, 5H), 4.52 (dt, J = 10.0, 7.0 Hz, 1H), 3.92 (dt, J = 8.3, 6.9 Hz, 1H), 3.84 (td, J = 7.9, 6.1 Hz, 1H), 3.70 (s, 3H), 3.53 (d, J = 9.9 Hz, 1H), 1.99 – 1.77 (m, 2H), 1.75 – 1.63 (m, 1H), 1.52 – 1.37 (m, 1H).

SFC: (ChiralCel OZ-3, 2% of solvent blend (1:1 methanol:*i*-propanol with 0.2% formic acid), 2.5 mL min^{-1}, 15 min, UV 210 nm) retention times of 3.3 min (minor) and 5.3 min (major), 84% ee.

E. Application of Aryldiazoketones and Characterization

Absolute configuration: the absolute configuration is tentatively assigned by analogy based on X-ray crystallography.

i. General Procedure For Beckmann-Rearrangement and Characterization

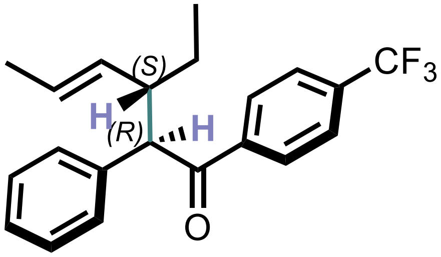

(2R,3S,E)-3-ethyl-2-phenyl-1-(4-(trifluoromethyl)phenyl)hex-4-en-1-one (12)

This compound was prepared according to the general procedure for C-H insertion using *trans*-2-hexene (1.73 mL, 1.16 g, 5 equiv, 13.8 mmol) as the substrate and 2-diazo-2-phenyl-1-(4-(trifluoromethyl) -phenyl)ethan-1-one (0.800 g, 1 equiv, 2.8 mmol) under the catalyst of Rh_2(*S*-TPPTTL)$_4$ (34 mg, 0.005 equiv, 0.5 mol%, 13.8 μmol). After flash chromatography (3% ether in hexanes) the product was obtained as a white solid (initially as a clear oil but solidifies after leaving product in a -20 ^{o}C freezer) (0.673 g, 71% yield, >20:1 r.r., >20:1 d.r., >99% ee).

Rf = 0.822 (5% diethyl ether/hexanes)

$[\alpha]^{20}_{D}$: -75.0^{o} (c 1.996 g/100 mL, EtOAc).

^{1}H NMR (600 MHz, $CDCl_3$) δ 8.05 (d, *J* = 8.2 Hz, 2H), 7.66 (d, *J* = 8.3 Hz, 2H), 7.25 – 7.20 (m, 4H), 7.17 (td, *J* = 6.9, 1.6 Hz, 1H), 5.19 – 5.10 (m, 1H), 4.98 (ddd, *J* = 15.2, 9.4, 1.8 Hz, 1H), 4.43 (d, *J* = 9.9 Hz, 1H), 2.84 (qd, *J* = 9.6, 3.1 Hz, 1H), 1.53 (ddddd, *J* = 14.7, 11.5, 8.4, 5.3, 1.4 Hz, 1H), 1.44 (dd, *J* = 6.4, 1.6 Hz, 3H), 1.28 – 1.21 (m, 1H) 0.87 (t, *J* = 7.4 Hz, 3H).

^{13}C NMR (151 MHz, $CDCl_3$) δ 199.3, 140.3, 137.5, 134.0 (q, *J* = 32.6 Hz), 131.4, 129.1, 128.8, 128.56, 127.6, 127.0, 125.6 (q, *J* = 3.7 Hz), 123.6 (q, *J* = 272.8 Hz), 59.1, 48.1, 26.8, 17.8, 11.9.

^{19}F NMR (565 MHz, $CDCl_3$) δ -63.2.

IR (neat) 2964, 2933, 2360, 2342, 1686, 1322, 1170, 1132, 1067, 747, 410, cm^{-1}

HRMS (+p APCI) calc. mass for $C_{21}H_{22}F_3O$ $[M + H]^+$ 347.1623; obs. mass for $C_{21}H_{22}F_3O$ $[M + H]^+$ 347.1609.

HPLC (Chiralpak RRW column, 1.0% *i*-propanol in hexane, 1.0 mL min^{-1}, 30 min, UV 230 nm) retention times of 5.8 min (major) and 6.7 min (minor), >99% ee.

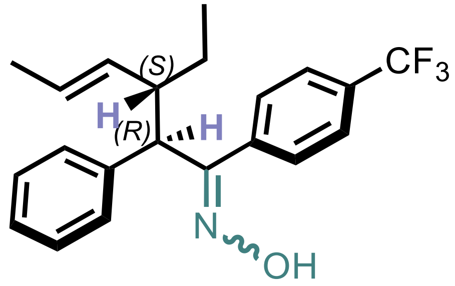

(2R,3S,4E)-3-ethyl-2-phenyl-1-(4-(trifluoromethyl)phenyl)hex-4-en-1-one oxime (24)

To a flame-dried round-bottom flask was added (2R,3S,E)-3-ethyl-2-phenyl-1-(4-(trifluoromethyl)phenyl)hex-4-en-1-one **(12)** (607 mg, 1 equiv, 1.75 mmol) and hydroxylamine hydrochloride (731 mg, 6 equiv, 10.5 mmol) and sealed. The reaction vessel was back-filled nitrogen three times. Subsequently, ethanol (80.7 mg, 1.75 mL, 1 molar, 1 equiv, 1.75 mmol) was added to the reaction vessel. Pyridine (832 mg, 10.5 mL, 1 molar, 6 equiv, 10.5 mmol) was added to the stirring vessel slowly. After complete addition of pyridine, the reaction mixture was allowed to react overnight at 120 °C. The reaction was cooled to room temperature and then concentrated *in vacuo*, neutralized with saturated $NaHCO_3$ solution, extracted with EtOAc, dried with $MgSO_4$, filtered, the filtrate concentrated *in vacuo* and then purified via flash chromatography hexane/EtOAc (0-100%; product elutes at 50%). The collected fractions were concentrated *in vacuo* to afford (2R,3S,4E)-3-ethyl-2-phenyl-1-(4-(trifluoromethyl)phenyl)hex-4-en-1-one oxime (550 mg, 1.53 mmol, 87.3% yield) as a clear salmon-color oil.

Note: Oxime product seems to decompose over several days into a solid. ^{1}H NMR analysis shows disappearance of alkene peaks of decomposed material.

E:Z ratio: 3.3:1 based on ^{19}F-NMR analysis.

^{1}H-NMR: For mixture of isomers - ^{1}H NMR (400 MHz, $CDCl_3$) **δ** 8.41 (s, 0.2H), 7.65 (s, 1H), 7.57 – 7.54 (m, 2H), 7.51 – 7.49 (d, *J* = 8.0 Hz, 0.5H), 7.31 – 7.15 (m, 6H), 7.11 – 7.04 (m, 4H), 5.40 – 5.29 (m, 0.4H), 5.20 – 5.10 (m, 1H), 5.03 (ddd, *J* = 15.2, 8.9, 1.6 Hz, 0.3H), 4.91 (ddq, *J* = 15.1, 9.2, 1.5 Hz, 1H), 4.75 (d, *J* = 11.4 Hz, 0.3H), 4.13 (q, *J* = 7.1 Hz, 1H), 3.57 (d, *J* = 10.7 Hz, 1H), 2.81 – 2.71 (m, 0.3H), 2.58 (qd, *J* = 9.6, 2.8 Hz, 1H), 2.00 – 1.86 (m, 1H), 1.54 (dd, *J* = 6.4, 1.6 Hz, 1H), 1.43 (dd, *J* = 6.4, 1.6 Hz, 3H), 1.40 – 1.29 (m, 1H), 0.90 (dt, *J* = 14.3, 7.3 Hz, 4H).

^{13}C-NMR: (101 MHz, $CDCl_3$) **δ** 171.23, 160.32, 159.40, 138.96, 138.75, 138.09, 132.50, 131.99, 130.49, 130.17, 129.90, 129.49, 128.56, 128.07, 128.04, 127.97, 127.91, 127.41, 126.78, 126.65, 124.99, 124.95, 124.91, 122.57, 77.35, 77.23, 77.03, 76.71, 60.44, 57.23, 48.99, 46.72, 44.33, 26.50, 26.23, 21.08, 18.00, 17.83, 14.21, 11.76, 11.53, 0.01.

^{19}F NMR (376 MHz, $CDCl_3$) δ -62.8, -62.8.

IR (neat): 2963, 2932, 1452, 1405, 1324, 1167, 1127, 1111, 1063, 1018, 965, 843, 731, 701 cm^{-1}.

HRMS (+p APCI): calc. mass for $C_{21}H_{23}ONF_3$ [M + H]$^+$ 362.1726; obs. mass for $C_{21}H_{23}ONF_3$ [M + H]$^+$ 362.17222.

HPLC: (Chiralpak AD-H column, 1.0% *i*-propanol in hexane, 1.0 mL min^{-1}, 0.5 mg mL^{-1}, 60 min, UV 210 nm) retention times of 24.5 min, 29.2 min, 34.0 min, and 48.8 min.

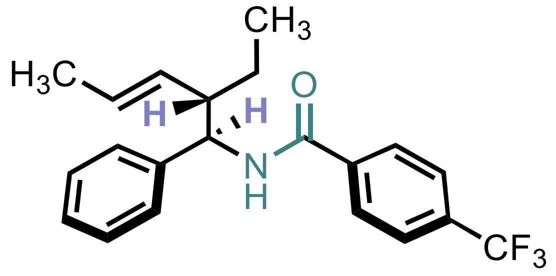

N-((1R,2S,E)-2-ethyl-1-phenylpent-3-en-1-yl)-4-(trifluoromethyl)benzamide (25)

The procedure is adapted from a literature precedent.[32] To a flame-dried round-bottom flask charged with a stir-bar was added (2R,3S,4E)-3-ethyl-2-phenyl-1-(4-(trifluoromethyl)phenyl)hex-4-en-1-one oxime **(24)** (127 mg, 1 equiv, 351 μmol), 4-methylbenzenesulfonyl chloride (67.0 mg, 1 equiv, 351 μmol) and DMAP (4.29 mg, 0.1 equiv, 35.1 μmol) and sealed. Subsequently, triethylamine (35.6 mg, 49.0 μL, 1 equiv, 351 μmol) was added to the stirring vessel. The reaction mixture was allowed to stir at room temperature overnight. The reaction was then concentrated *in vacuo*, purified via flash chromatography hexane/pet ether (0-100%; product elutes at 50%). The collected fractions were concentrated *in vacuo*, resuspended in neat ACN, the suspension filtered thru a 0.2 μm filter, and the filtrate subjected to Prep HPLC (95-5 reverse phase H_2O/ACN + 0.1% TFA). The collected fractions were concentrated *in vacuo*, free-based with a mixture of saturated bicarbonate solution and CH_2Cl_2, the organic layer extracted, dried with $MgSO_4$, filtered, and the filtrate concentrated *in vacuo* to afford N-((1R,2S,E)-2-ethyl-1-phenylpent-3-en-1-yl)-4-(trifluoromethyl)benzamide (25 mg, 69 μmol, 20% yield, 96% ee) as an amorphous white solid. Minute crystals were grown from slow-evaporation in ACN/H_2O at room temperature.

^{1}H NMR (600 MHz, $CDCl_3$) δ 7.87 – 7.83 (m, 2H), 7.72 – 7.69 (m, 2H), 7.34 – 7.30 (m, 2H), 7.29 – 7.21 (m, 3H), 6.73 (d, J = 8.4 Hz, 1H), 5.61 (dqd, J = 15.2, 6.4, 0.7 Hz, 1H), 5.21 – 5.11 (m, 2H), 2.38 (tt, J = 9.8, 4.7 Hz, 1H), 1.74 (dd, J = 6.4, 1.7 Hz, 3H), 1.54 (dqd, J = 13.4, 7.4, 4.5 Hz, 1H), 1.09 (ddq, J = 14.5, 10.0, 7.4 Hz, 1H), 0.88 (t, J = 7.4 Hz, 3H).

^{13}C NMR (152 MHz, $CDCl_3$) δ 164.8, 139.4, 138.1, 133.5 – 132.9 (q, *J* = 32.6 Hz), 130.5, 129.3, 128.2, 127.6, 127.4, 127.3, 125.7 - 125.7 (q, *J* = 3.7 Hz), 126.4 - 121.0 (d, *J* = 272.6 Hz), 56.5, 49.9, 24.9, 18.2, 12.0.

^{19}F NMR (565 MHz, $CDCl_3$) δ -63.0.

IR (neat): 3285, 3031, 2961, 2929, 2856, 1635, 1579, 1543, 1497, 1454, 1407, 1325, 1168, 1130, 1068, 1017, 964, 856, 773, 701, 573, 470, 419, 406 cm^{-1}.

HRMS (-p APCI): calc. mass for $C_{21}H_{23}ONF_3$ $[M + H]^+$ 362.1726; obs. mass for $C_{21}H_{23}ONF_3$ $[M + H]^+$ 362.17242.

[α]$^{20}_{D}$: -19° (c 0.051 g/100 mL, EtOAc).

HPLC: (Chiralcel OD-H column, 1.0% *i*-propanol in hexane, 1.0 mL min^{-1}, 0.5 mg mL^{-1}, 60 min, UV 210 nm) retention times of 17.1 min (major) and 28.8 min (minor), 96% ee.

ii. General Procedure for Wittig Olefination and Characterization

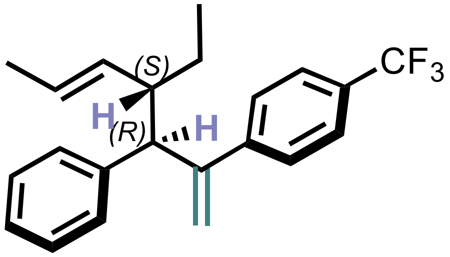

1-((3R,4S,E)-4-ethyl-3-phenylhepta-1,5-dien-2-yl)-4-(trifluoromethyl)benzene (26)

A 2.46 M solution of *n*-butyllithium in hexanes (14.4 mg, 93.8 μL, 2.40 molar, 1.5 equiv, 225 μmol) was added dropwise to a solution of methyltriphenylphosphonium bromide (85.7 mg, 1.6 equiv, 240 μmol) in THF (12.0 mL) at 0 °C and the reaction mixture was stirred at 0 °C for 30 min. A solution of (2R,3S,E)-3-ethyl-2-phenyl-1-(4-(trifluoromethyl)phenyl)hex-4-en-1-one **(12)** (52.0 mg, 1 equiv, 0.150 mmol) in THF (3.00 mL) was added dropwise at 0 °C. The reaction mixture gradually warmed up to room temperature overnight. A saturated aqueous solution of ammonium chloride (30 mL) was added and the reaction mixture was extracted with ether (3 x 30 mL). The ether layers were combined, washed with a saturated aqueous solution of sodium chloride (20 mL), dried over sodium sulfate, and concentrated *in vacuo* to yield a clear colorless liquid. The crude liquid was purified by SiO_2 column chromatography with 100% hexane to afford product as a clear oil (34.5 mg, 67% yield).

R_f (1% Et_2O/Hexane) = 0.5

^{1}H NMR (400 MHz, $CDCl_3$) δ 7.49 (d, J = 8.1 Hz, 2H), 7.36 – 7.29 (m, 2H), 7.26 – 7.19 (m, 2H), 7.17 – 7.11 (m, 3H), 5.46 – 5.37 (m, 2H), 5.16 (dq, J = 15.3, 6.3 Hz, 1H), 5.06 – 4.92 (m, 1H), 3.64 (d, J = 9.3 Hz, 1H), 2.44 (qd, J = 9.3, 3.2 Hz, 1H), 1.81 (dqd, J = 13.3, 7.4, 3.2 Hz, 1H), 1.49 (dd, J = 6.3, 1.6 Hz, 3H), 1.29 – 1.19 (m, 1H), 0.89 (t, J = 7.4 Hz, 3H).

^{13}**C NMR (101 MHz, CDCl$_3$) δ** 150.3, 147.1 (q, J = 1.4 Hz), 141.7, 132.7, 129.14, 129.08 (q, J = 32.3 Hz), 127.9, 127.2, 126.7, 126.2, 125.0 (q, J = 3.8 Hz), 123.8 (q, J = 271.9 Hz), 115.2, 55.9, 48.2, 26.5, 17.9, 11.9.

^{19}**F NMR (376 MHz, CDCl$_3$) δ** -62.5.

IR (neat): 607, 699, 759, 849, 909, 966, 1016, 1066, 1094, 1123, 1165, 1323, 1378, 1404, 1453, 1495, 1616, 2932, 2963 cm^{-1}.

HRMS (+p APCI): calc. mass for $C_{22}H_{24}F_3$ [M + H]$^+$ 345.1825; obs. mass for $C_{22}H_{24}F_3$ [M + H]$^+$ 345.1828

[α]^{20}$_D$: -60.3^o (c = 0.410 mg/mL, CHCl$_3$).

HPLC: Product cannot be resolved with chiral HPLC, UPLC, and SFC conditions.

iii. General Procedure for Ketone Reduction and Characterization

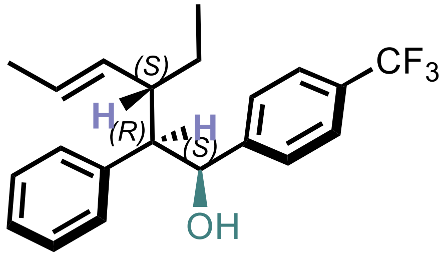

(1S,2R,3S,E)-3-ethyl-2-phenyl-1-(4-(trifluoromethyl)phenyl)hex-4-en-1-ol (27)

NaBH$_4$ (68.8 mg, 3 equiv, 1.8 mmol) was added to (2R,3S,E)-3-ethyl-2-phenyl-1-(4-(trifluoromethyl)phenyl)hex-4-en-1-one **(12)** (210 mg, 1 equiv, 0.60 mmol) in a co-solvent of THF (3.0 mL) and MeOH (3.0 mL) at -30 ^{o}C. The reaction mixture was stirred for 5 hours at -30 ^{o}C and the solvent was concentrated. The residue was diluted with water (10 mL) and the mixture was extracted with CH_2Cl_2 (3 x 5 mL). The combined organic layers were washed with brine, dried, filtered and the filtrate evaporated to afford crude product. Purification on silica gel with gradient 0-10% Et_2O in hexane afford major (1S,2R,3S,E)-3-ethyl-2-phenyl-1-(4-(trifluoromethyl)phenyl)hex-4-en-1-ol **(27)** (127.5 mg, 60% yield, 98% ee), and minor (1R,2R,3S,E)-3-ethyl-2-phenyl-1-(4-(trifluoromethyl)phenyl)hex-4-en-1-ol **(28)** (25.5 mg, 12% yield, 98% ee). The combined yield is 72% yield and the d.r. of the reduction is 5:1.

R$_f$ (6:1 Hexanes:Et_2O) = 0.25

^{1}H NMR (400 MHz, CDCl$_3$) δ 7.62 (d, J = 7.6 Hz, 2H), 7.49 (d, J = 8.0 Hz, 2H), 7.39 – 7.32 (m, 2H), 7.31 – 7.21 (m, 3H), 5.29 (dq, J = 15.3, 6.3 Hz, 1H), 5.16 – 5.05 (m, 2H), 3.00 (dd, J = 9.2, 4.1 Hz, 1H), 1.96 – 1.78 (m, 2H), 1.70 (dd, J = 6.3, 1.6 Hz, 3H), 1.32 – 1.25 (m, 1H), 0.96 (ddt, J = 13.4, 9.2, 7.3 Hz, 1H), 0.70 (t, J = 7.4 Hz, 3H).

^{13}C NMR (101 MHz, CDCl$_3$) δ 146.7 (q, J = 1.4 Hz), 137.8, 131.3, 130.3, 130.0 (q, J = 32.3 Hz), 128.2, 127.8, 127.5, 127.1, 125.3 (q, J = 3.8 Hz), 124.2 (q, J = 272.0 Hz), 75.2, 57.9, 45.4, 26.8, 18.1, 11.7.

^{19}F NMR (376 MHz, CDCl$_3$) δ -62.4.

IR (neat): 611, 651, 703, 743, 754, 843, 896, 979, 1017, 1039, 1067, 1122, 1163, 1323, 1379, 1421, 1453, 1497, 1620, 2365, 2932, 2963, 3416 cm^{-1}.

HRMS (-p ESI): calc. mass for $C_{21}H_{23}O^{35}ClF_3$ [M + Cl]$^-$ 383.1395; obs. mass for $C_{21}H_{23}O^{35}ClF_3$ [M + Cl]$^-$ 383.1397.

[α]$^{20}_{D}$: -60.3° (c = 0.410 mg/mL, CHCl$_3$).

HPLC: (Regis (S,S) Whelk-O1, 1.0% *i*-propanol in hexane, 1.0 mL min^{-1}, 1.0 mg mL^{-1}, 30 min, UV 230 nm) retention times of 16.9 min (minor) and 18.7 min (major), 98% ee.

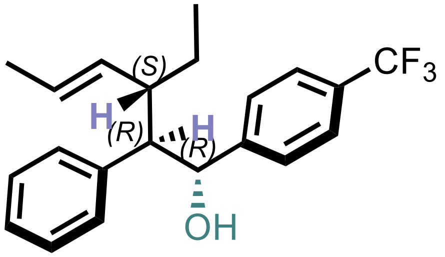

(1R,2R,3S,E)-3-ethyl-2-phenyl-1-(4-(trifluoromethyl)phenyl)hex-4-en-1-ol (28)

R$_f$ (6:1 Hexanes:Et$_2$O) = 0.30

^{1}H NMR (400 MHz, CDCl$_3$) δ 7.41 (d, J = 8.1 Hz, 2H), 7.27 (d, J = 8.8 Hz, 2H), 7.19 – 7.09 (m, 3H), 7.03 – 6.93 (m, 2H), 5.71 (dq, J = 15.3, 6.4 Hz, 1H), 5.40 – 5.23 (m, 1H), 5.05 (d, J = 10.0 Hz, 1H), 3.01 (dd, J = 10.0, 4.0 Hz, 1H), 2.88 – 2.70 (m, 1H), 2.23 (s, 1H), 1.82 (dd, J = 6.4, 1.6 Hz, 3H), 1.38 – 1.30 (m, 1H), 1.04 – 0.94 (m, 1H), 0.86 (t, J = 7.3 Hz, 3H).

^{13}C NMR (101 MHz, $CDCl_3$) δ 147.5, 138.4, 131.9, 130.0, 129.3 (q, J = 32.3 Hz), 127.68, 127.66, 127.3, 126.4, 124.9 (q, J = 3.8 Hz), 124.1, (q, J = 272.1 Hz), 75.3, 57.6, 44.7, 26.6, 18.2, 12.2.

^{19}F NMR (376 MHz, $CDCl_3$) δ -62.5.

IR (neat): 608, 636, 681, 704, 758, 814, 842, 980, 1017, 1037, 1069, 1126, 1165, 1326, 1416, 1454, 1495, 1620, 1746, 2362, 2929, 2961, 3390 cm^{-1}.

HRMS (-p ESI): calc. mass for $C_{21}H_{23}O^{35}ClF_3$ $[M + Cl]^-$ 383.1395; obs. mass for $C_{21}H_{23}O^{35}ClF_3$ $[M + Cl]^-$ 383.1397.

$[\alpha]^{20}_D$: -60.3° (c = 0.410 mg/mL, $CHCl_3$).

SFC: (Regis (S,S) Whelk-O1, 1% of solvent blend (1:1 methanol:*i*-propanol with 0.2% formic acid), 2.5 mL min^{-1}, 1.0 mg mL^{-1}, 5 min, UV 230 nm) retention times of 1.6 (major) and 2.2 min (minor), 98% ee.

iv. General Procedure for Selective Reduction and Characterization

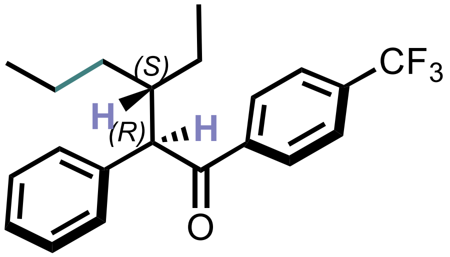

(2R,3S)-3-ethyl-2-phenyl-1-(4-(trifluoromethyl)phenyl)hexan-1-one (29)

To a solution of (2R,3S,E)-3-ethyl-2-phenyl-1-(4-(trifluoromethyl)phenyl)hex-4-en-1-one **(12)** (34.6 mg, 1 equiv, 0.100 mmol) and triethylsilane (140 mg, 192 μL, 12 equiv, 1.20 mmol) in ethanol (1.0 mL) was added $PdCl_2(CH_3CN)_2$ (259 μg, 0.10 equiv, 10.0 μmol) under argon atmosphere. The reaction mixture was stirred for 8 hours at 25 °C and checking conversion by NMR. After completion, the organic solvents were evaporated and the residual oil was subjected to silica gel column chromatography (gradient 0-2% Et_2O in hexanes) to give a colorless oil (25.5 mg, 73% yield, 99% ee) which slowly solidifies under high vacuum.

R_f (9:1 Hexanes:Et_2O) = 0.65

^{1}H NMR (400 MHz, CDCl$_3$) δ 8.06 (d, J = 8.1 Hz, 2H), 7.66 (d, J = 8.2 Hz, 2H), 7.37 – 7.26 (m, 4H), 7.24 – 7.18 (m, 1H), 4.49 (d, J = 10.3 Hz, 1H), 2.52 – 2.34 (m, 1H), 1.52 – 1.36 (m, 2H), 1.31 – 1.25 (m, 1H), 1.20 – 0.92 (m, 3H), 0.86 (t, J = 7.5 Hz, 3H), 0.74 (t, J = 6.9 Hz, 3H).

^{13}C NMR (101 MHz, CDCl$_3$) δ 199.7, 140.3, 137.5, 134.0 (q, J = 32.6 Hz), 129.1, 128.9, 128.7, 127.3, 125.6 (q, J = 3.7 Hz), 123.6 (q, J = 272.7 Hz), 57.4, 41.5, 31.3, 23.8, 18.9, 14.3, 10.5.

^{19}F NMR (376 MHz, CDCl$_3$) δ -63.2.

IR (neat): 515, 547, 599, 702, 744, 827, 872, 1014, 1067, 1112, 1131, 1169, 1202, 1268, 1322, 1408, 1455, 1494, 1510, 1582, 1686, 2184, 2365, 2874, 2933, 2960 cm^{-1}.

HRMS (+p APCI): calc. mass for $C_{21}H_{24}OF_3$ [M + H]$^+$ 349.1774; obs. mass for $C_{21}H_{24}OF_3$ 349.1768 [M + H]$^+$.

[α]$^{20}_{D}$: -56.3^o (c = 0.85 g/100 mL, CHCl$_3$).

HPLC: (Regis (S,S) Whelk-O1, 0% *i*-propanol in hexane, 1.0 mL min^{-1}, 1.0 mg mL^{-1}, 30 min, UV 230 nm) retention times of 10.4 min (minor) and 14.1 min (major), 99% ee.

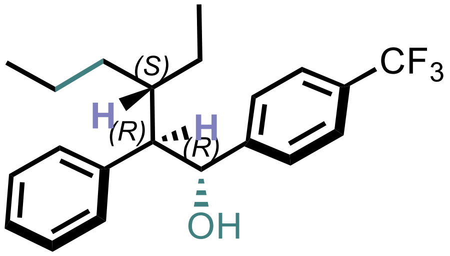

(1R,2R,3S)-3-ethyl-2-phenyl-1-(4-(trifluoromethyl)phenyl)hexan-1-ol (30)

To a solution of (2R,3S,E)-3-ethyl-2-phenyl-1-(4-(trifluoromethyl)phenyl)hex-4-en-1-one **(12)** (34.6 mg, 1 equiv, 0.100 mmol) and triethylsilane (140 mg, 192 μL, 12 equiv, 1.20 mmol) in ethanol (1.0 mL) was added $PdCl_2(CH_3CN)_2$ (259 μg, 0.10 equiv, 10.0 μmol) under an argon atmosphere. The reaction mixture was stirred for 36 hours at 40 ^{o}C and checking conversion by NMR. After completion, the organic solvents

were evaporated and the residual oil was subjected to silica gel column chromatography (gradient 0-12% Et_2O in hexanes) to afford the product as a colorless oil (24.9 mg, 71% yield, 98% ee).

R_f (9:1 Hexanes:Et_2O) = 0.10

^{1}H NMR (400 MHz, $CDCl_3$) δ 7.40 (d, J = 8.1 Hz, 2H), 7.20 (d, J = 8.1 Hz, 2H), 7.15 – 7.08 (m, 3H), 6.92 (dd, J = 7.4, 2.2 Hz, 2H), 5.18 (dd, J = 8.9, 4.0 Hz, 1H), 3.10 (dd, J = 8.9, 5.4 Hz, 1H), 2.06 – 1.95 (m, 2H), 1.70 – 1.59 (m, 1H), 1.54 – 1.37 (m, 2H), 1.34 – 1.23 (m, 1H), 1.21 – 1.11 (m, 1H), 1.10 – 0.99 (m, 1H), 0.96 (t, J = 7.4 Hz, 3H), 0.90 (t, J = 7.3 Hz, 3H).

^{13}C NMR (101 MHz, $CDCl_3$) δ 147.7, 138.7, 130.0, 129.3 (q, J = 32.1 Hz), 127.7, 127.2, 126.5, 124.8 (q, J = 3.8 Hz), 124.1 (q, J = 272.0 Hz), 74.6, 55.0, 39.7, 32.3, 23.9, 20.9, 14.6, 11.6.

^{19}F NMR (376 MHz, $CDCl_3$) δ -62.5.

IR (neat): 602, 609, 618, 625, 634, 641, 649, 658, 667, 673, 683, 703, 750, 796, 844, 912, 936, 944, 951, 1017, 1041, 1068, 1125, 1164, 1324, 1380, 1417, 1454, 1496, 1620, 2874, 2931, 2959, 3029, 3442 cm^{-1}.

HRMS (+p ESI): calc. mass for $C_{21}H_{25}O^{35}ClF_3$ [M + ^{35}Cl]$^+$ - 385.1552; obs. mass for $C_{21}H_{25}O^{35}ClF_3$ [M + ^{35}Cl]$^+$ - 385.1552.

$[\alpha]^{20}_D$: +14.5° (c = 1.27 g/100 mL, $CHCl_3$).

SFC: (Regis (S,S) Whelk-O1, 1% of solvent blend (1:1 methanol:*i*-propanol with 0.2% formic acid), 2.5 mL min^{-1}, 1.0 mg mL^{-1}, 5 min, UV 230 nm) retention times of 1.8 min (major) and 2.0 min (minor), 98% ee.

v. General Procedure for Mitsunobu Reaction and Characterization

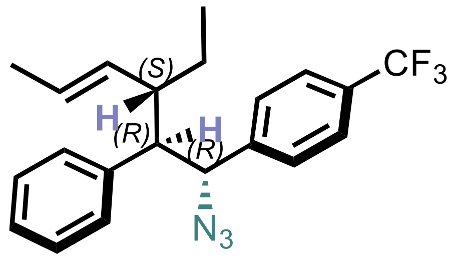

1-((1R,2R,3S,E)-1-azido-3-ethyl-2-phenylhex-4-en-1-yl)-4-(trifluoromethyl)benzene (31)

To an 8 mL vial, (1S,2R,3S,E)-3-ethyl-2-phenyl-1-(4-(trifluoromethyl)phenyl)hex-4-en-1-ol **(27)** (58.3 mg, 1 equiv, 0.17 mmol) and triphenylphosphine (87.9 mg, 2 equiv, 0.34 mmol) in toluene (1.5 mL) at 0 ^{o}C was added diphenyl phosphorazidate (92.10 mg, 72.1 µL, 2 equiv, 0.34 mmol) and diisopropyl (E)-diazene-1,2-dicarboxylate (67.67 mg, 65.9 µL, 2 equiv, 0.33 mmol) under an argon atmosphere. The reaction mixture was stirred for 18 hours and allowed to warmed up to room temperature by not replenishing the ice-bath. The reaction mixture was directly loaded by silica gel and purified by flash chromatography (100% hexane) to afford product as a clear oil (46.5 mg, 74% yield, 97% ee).

R_f (Hexane) = 0.21

^{1}H NMR (400 MHz, $CDCl_3$) δ 7.42 (d, J = 8.1 Hz, 2H), 7.29 – 7.21 (m, 2H), 7.15 – 7.03 (m, 3H), 6.96 – 6.88 (m, 2H), 5.74 (dq, J = 15.4, 6.4 Hz, 1H), 5.21 – 5.12 (m, 1H), 4.81 (d, J = 11.5 Hz, 1H), 3.01 (dd, J = 11.5, 3.6 Hz, 1H), 2.75 (tt, J = 9.4, 4.2 Hz, 1H), 1.83 (dd, J = 6.4, 1.7 Hz, 3H), 1.38 – 1.28 (m, 1H), 1.05 – 0.92 (m, 1H), 0.87 (t, J = 7.2 Hz, 3H).

^{13}C NMR (101 MHz, $CDCl_3$) δ 142.8 (q, J = 1.4 Hz), 137.1, 130.7, 129.8 (q, J = 32.4 Hz), 129.7, 128.6, 128.3, 127.7, 126.7, 125.3 (q, J = 3.8 Hz), 123.9 (q, J = 272.2 Hz), 67.9, 54.8, 45.4, 26.7, 18.3, 12.2.

^{19}F NMR (376 MHz, $CDCl_3$) δ -62.6.

IR (neat): 572, 603, 634, 666, 703, 757, 814, 840, 927, 979, 1018, 1068, 1109, 1123, 1165, 1246, 1323, 1419, 1454, 1496, 1620, 2097, 2963 cm^{-1}.

HRMS (+p ESI): calc. mass for $C_{21}H_{23}NF_3$ [M + H - N_2]$^+$ 346.1777; obs. mass for $C_{21}H_{23}NF_3$ [M + H - N_2]$^+$ 346.1786.

$[\alpha]^{20}_D$: -11.2^o (c = 1.34 g/100 mL, $CHCl_3$).

HPLC: (Chiralpak AD-H column, 0% *i*-propanol in hexane, 0.25 mL min^{-1}, 1.0 mg mL^{-1}, 30 min, UV 230 nm) retention times of 14.1 min (major) and 16.4 min (minor), 97% ee.

5. Crude C-H Insertion ^1H NMR Spectra for r.r. and d.r. Determination

Absolute configuration: the absolute configuration is tentatively assigned by analogy based on X-ray crystallography. The minor diastereomer/regioisomer stereoconfiguration is a relative stereoconfiguration; the absolute stereoconfiguration is not known of the minor diastereomers/regioisomers.

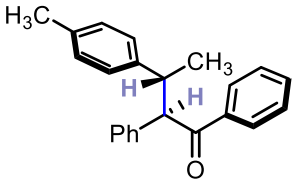

2b

^1H NMR (600 MHz, $CDCl_3$)

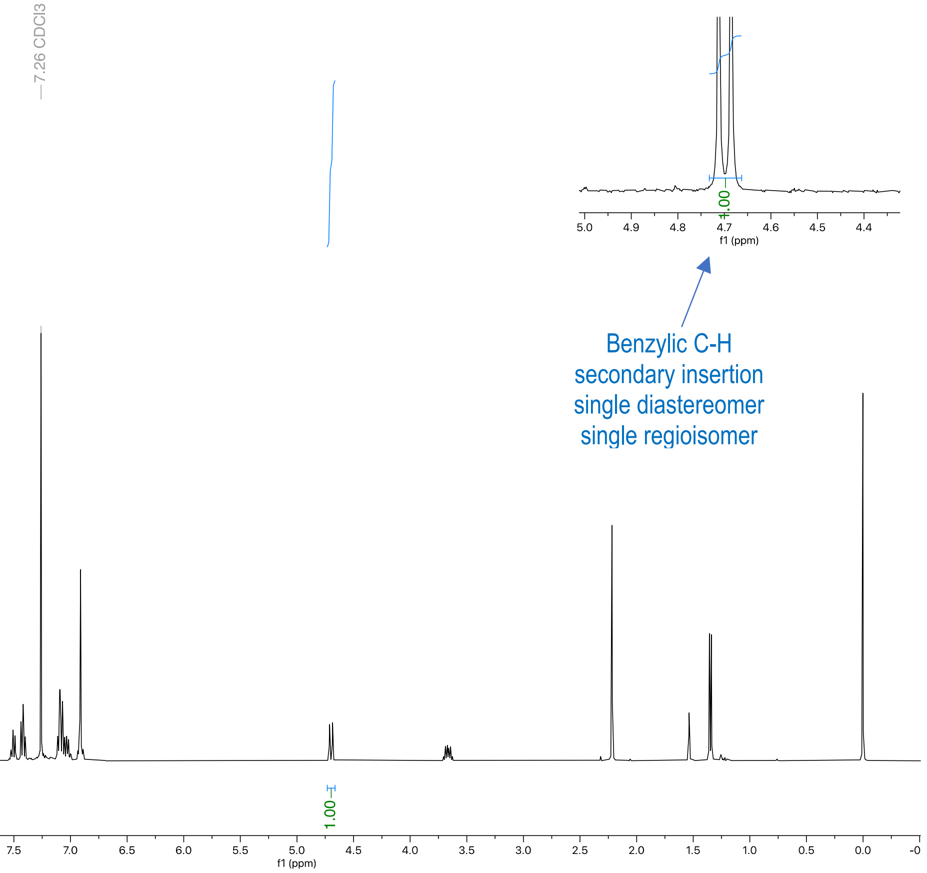

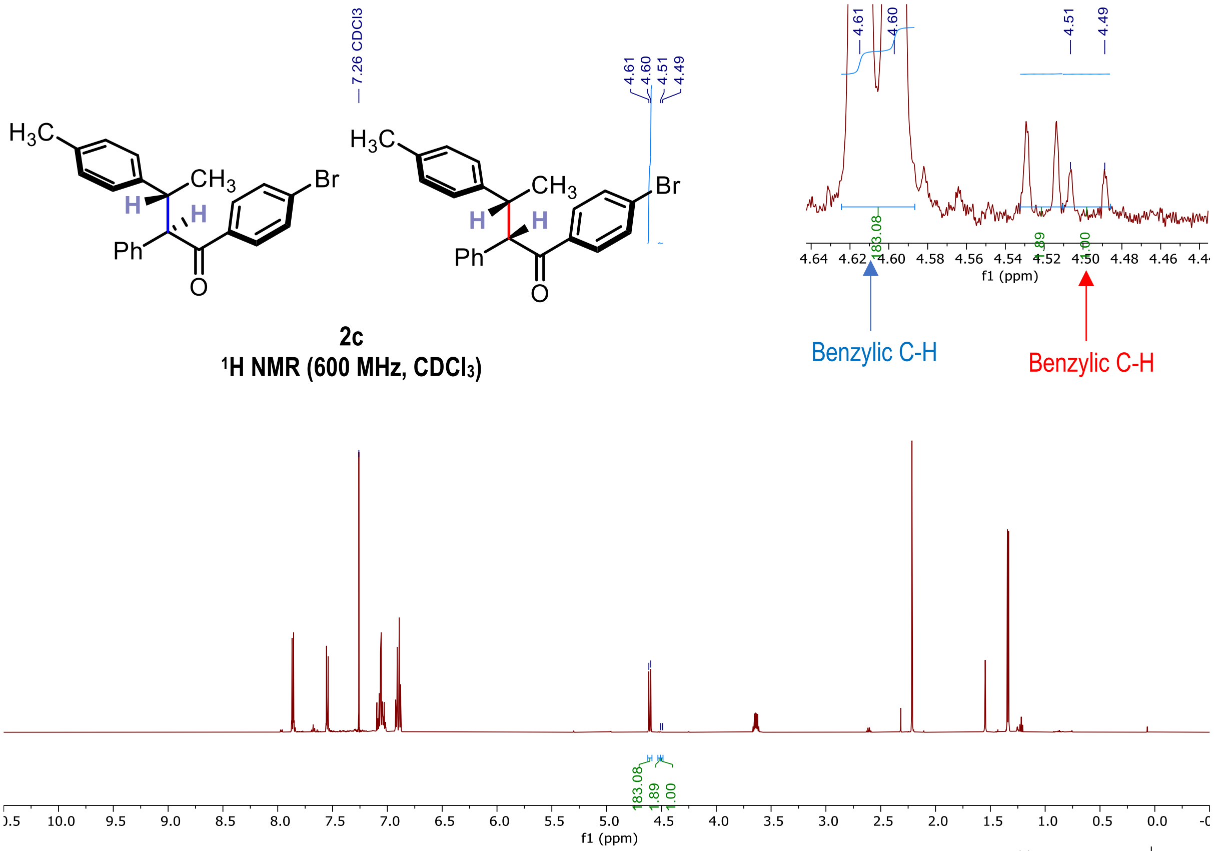

2c
^1H NMR (600 MHz, $CDCl_3$)

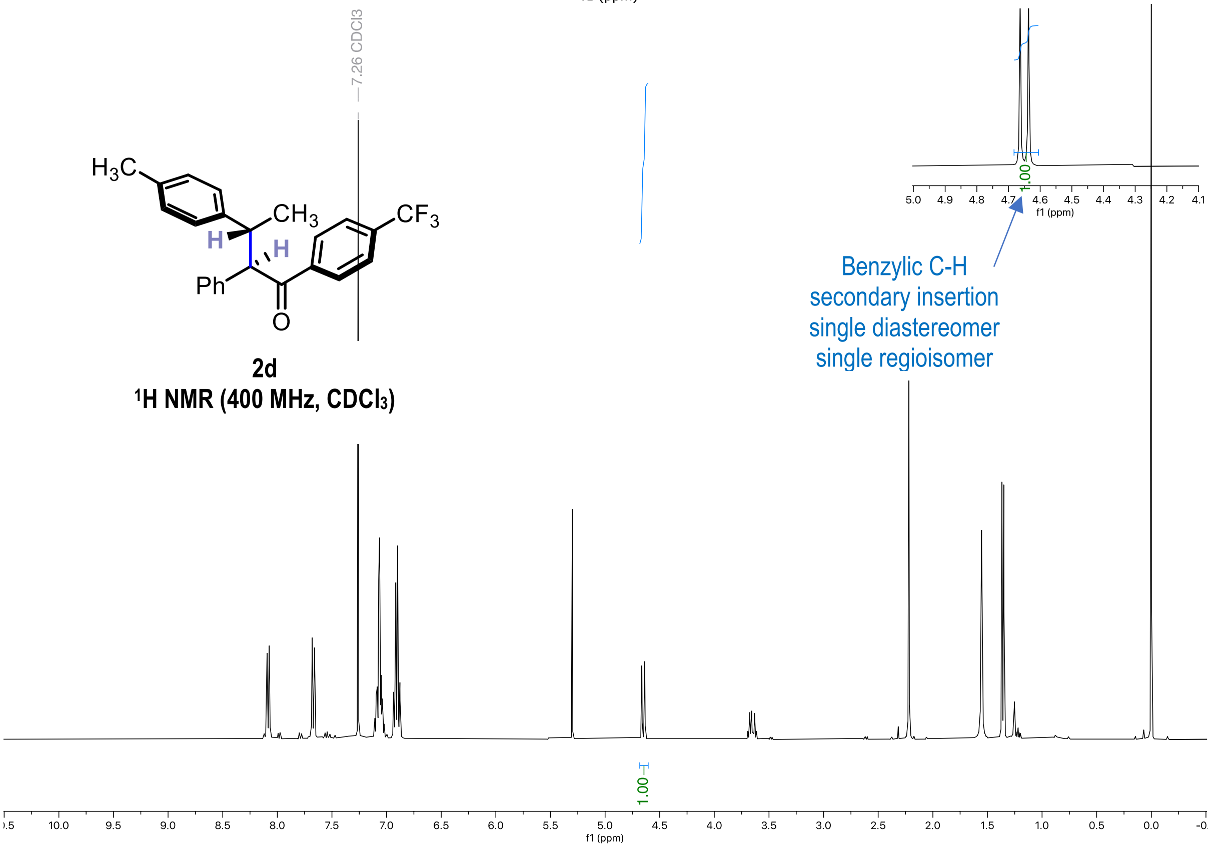

2d
^1H NMR (400 MHz, $CDCl_3$)

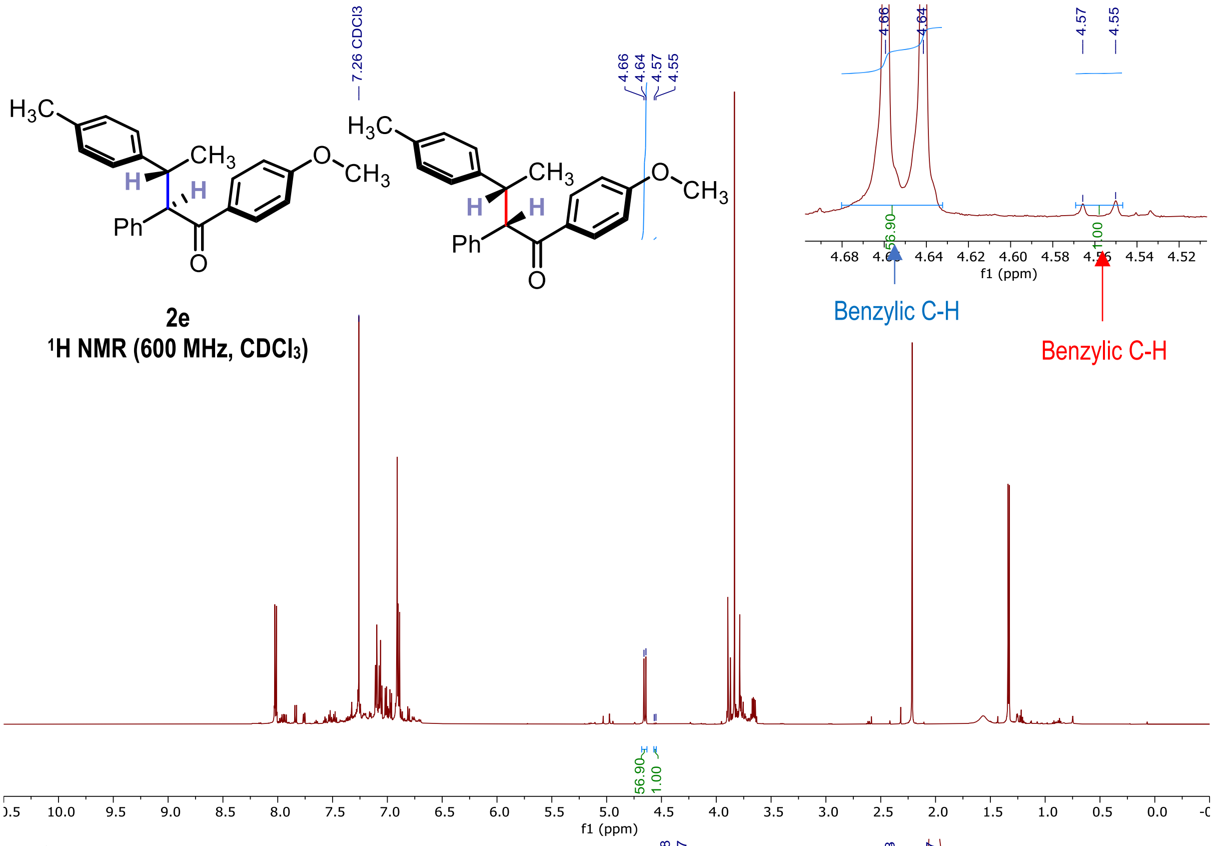
2e
^{1}H NMR (600 MHz, CDCl$_3$)
7.26 CDCl3
4.66
4.64
4.57
4.55
56.90
1.00
Benzylic C-H
Benzylic C-H
f1 (ppm)

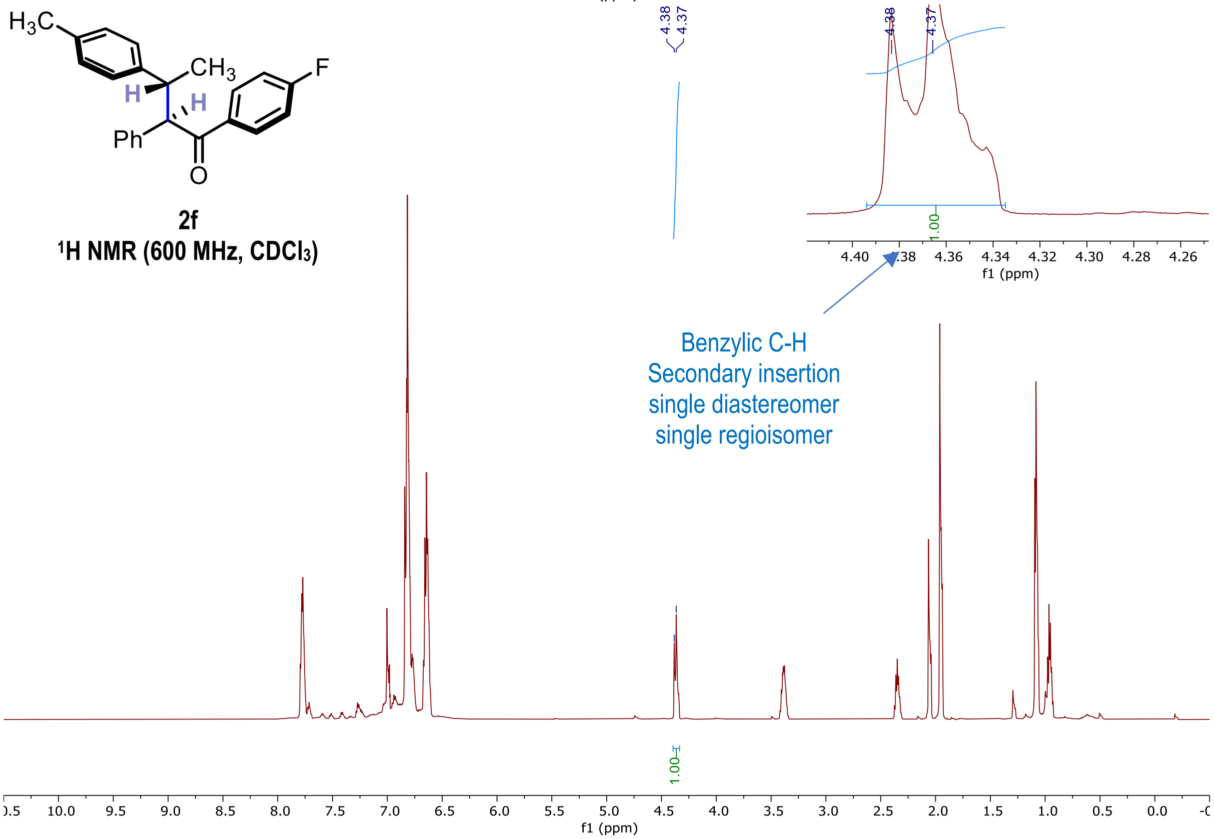
2f
^{1}H NMR (600 MHz, CDCl$_3$)
4.38
4.37
1.00
Benzylic C-H
Secondary insertion
single diastereomer
single regioisomer
f1 (ppm)

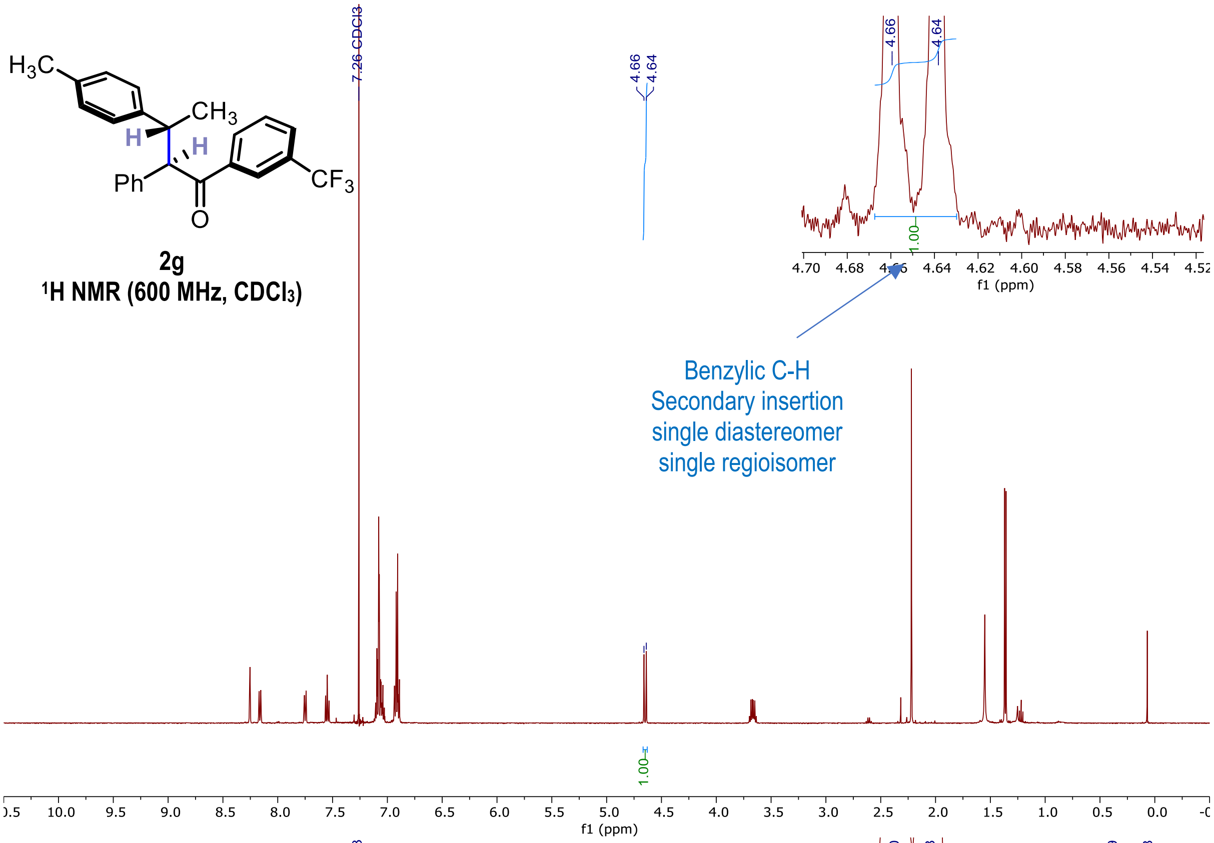

2g
^{1}H NMR (600 MHz, $CDCl_3$)

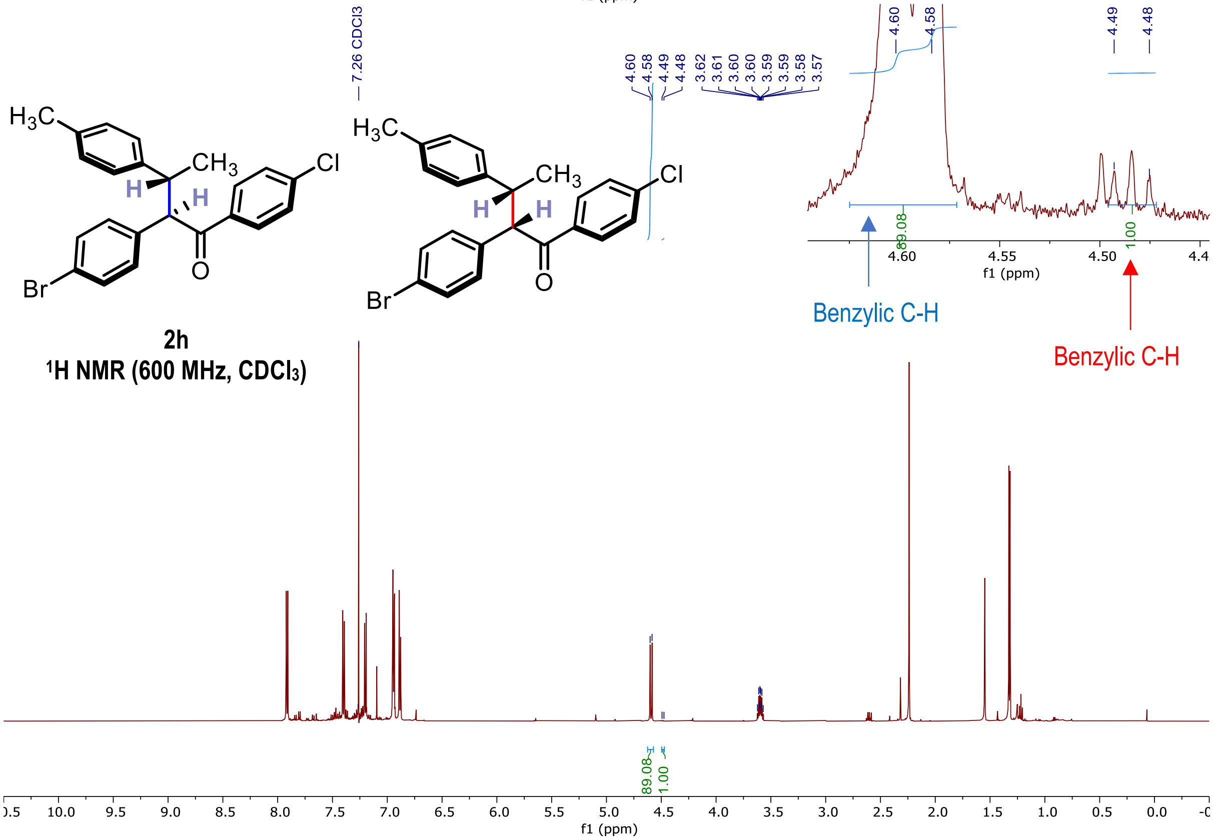

2h
^{1}H NMR (600 MHz, $CDCl_3$)

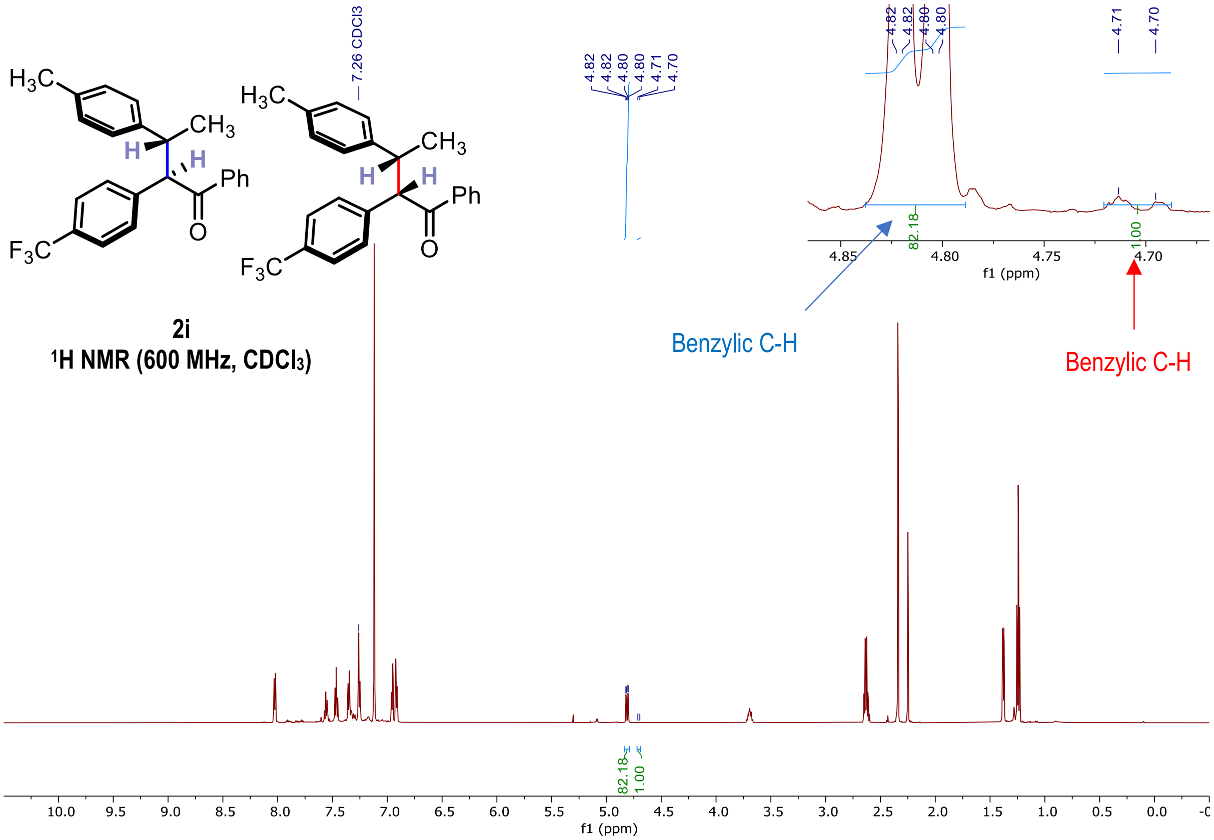

2i
^{1}H NMR (600 MHz, CDCl$_3$)
7.26 CDCl3
4.82
4.82
4.80
4.80
4.71
4.70
82.18
1.00
Benzylic C-H
Benzylic C-H
f1 (ppm)

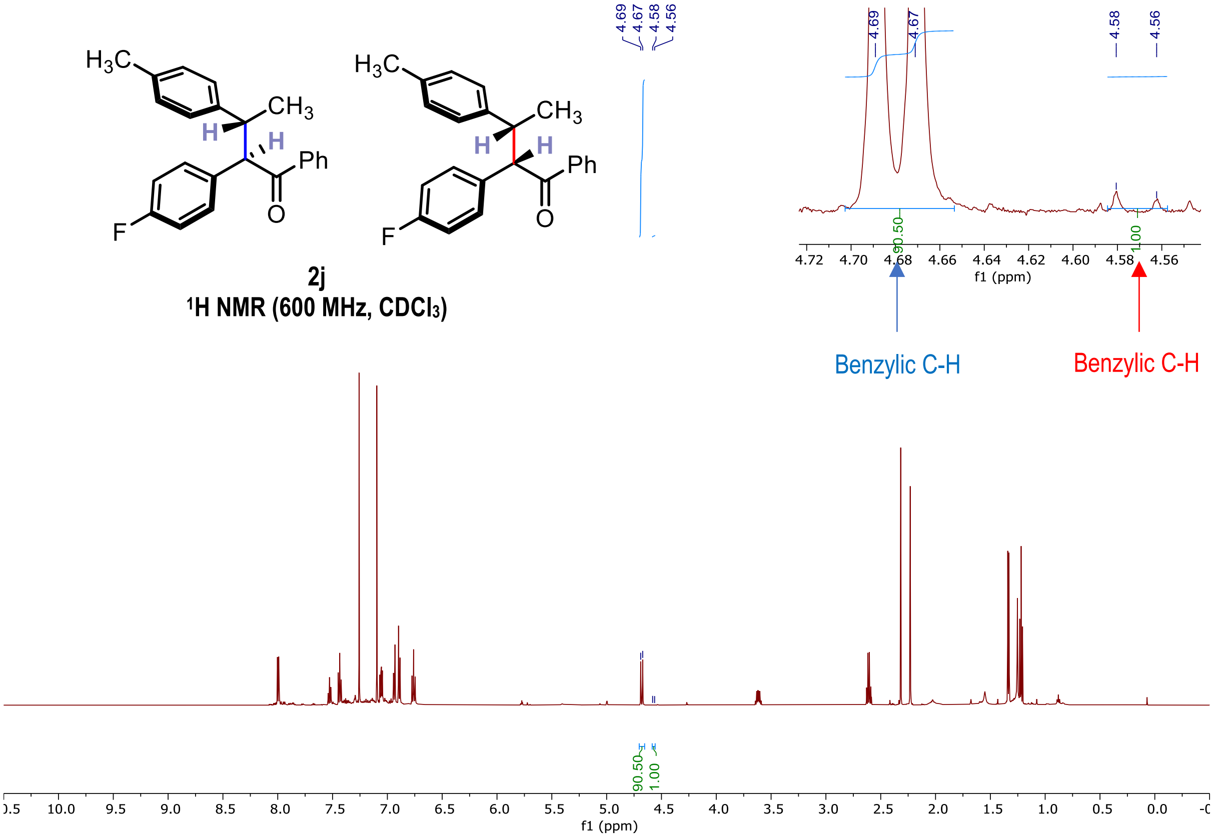

2j
^{1}H NMR (600 MHz, CDCl$_3$)
4.69
4.67
4.58
4.56
90.50
1.00
Benzylic C-H
Benzylic C-H
f1 (ppm)

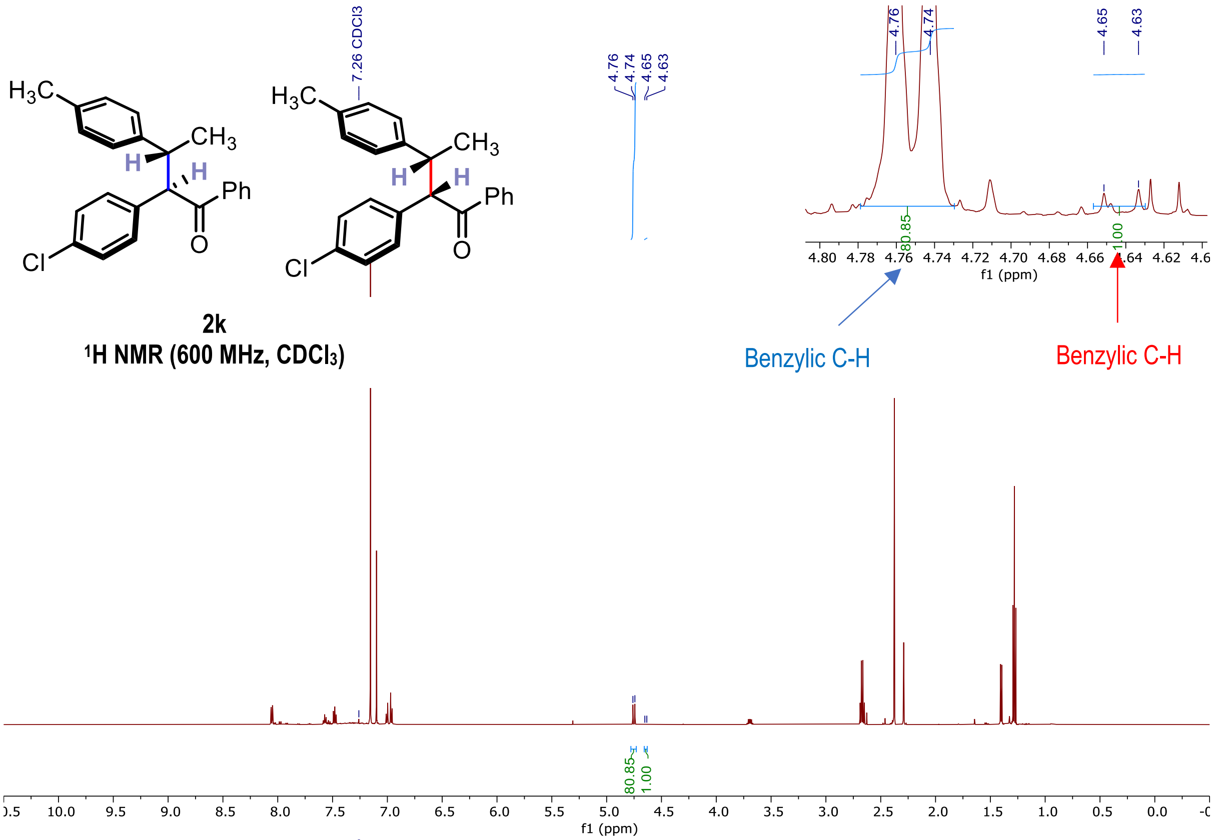

2k

^{1}H NMR (600 MHz, $CDCl_3$)

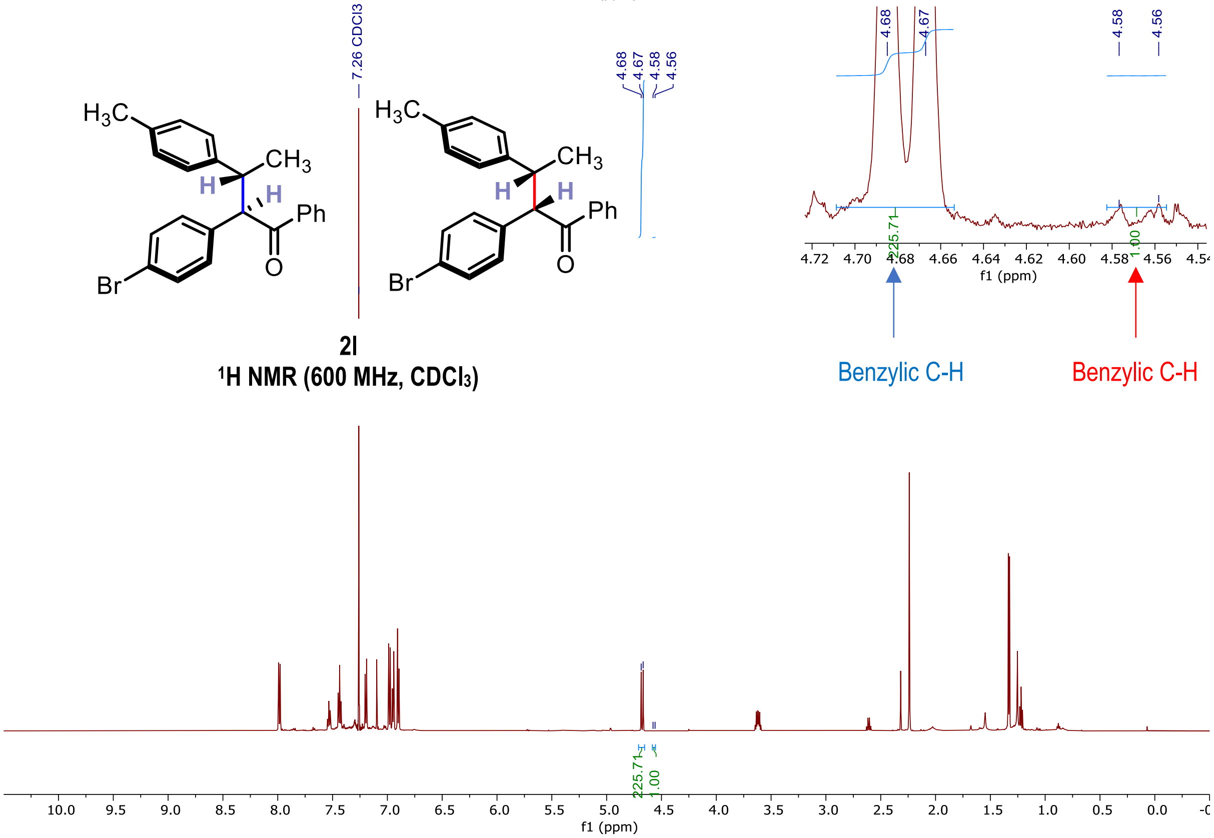

2l

^{1}H NMR (600 MHz, $CDCl_3$)

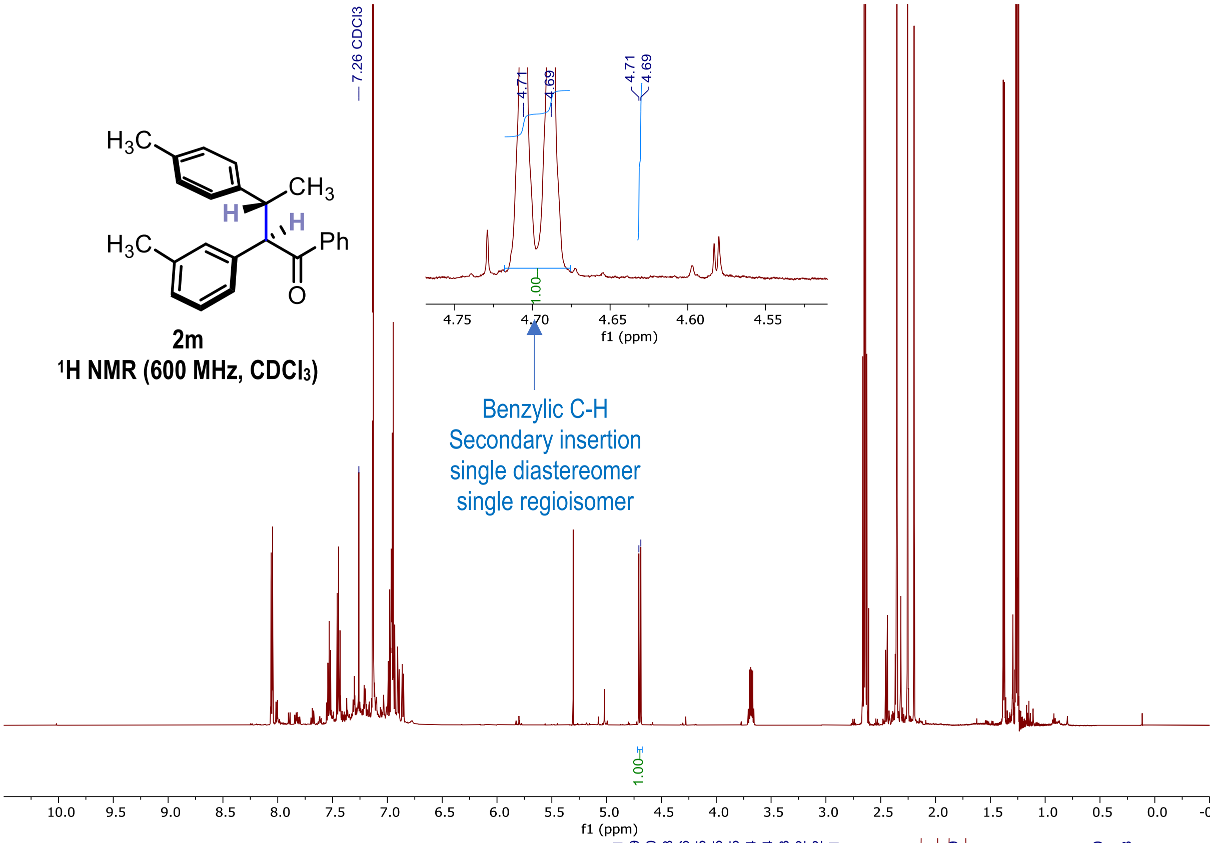

2m

^{1}H NMR (600 MHz, CDCl$_3$)

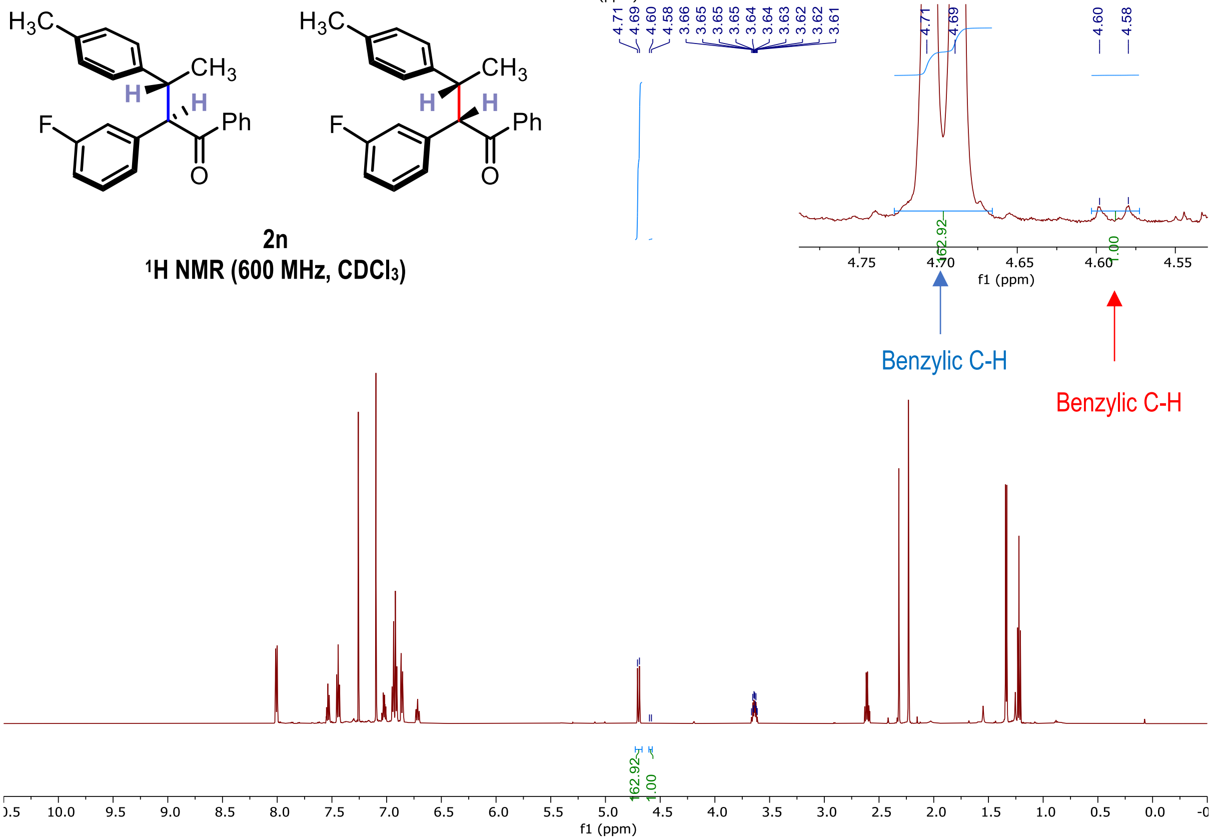

2n

^{1}H NMR (600 MHz, CDCl$_3$)

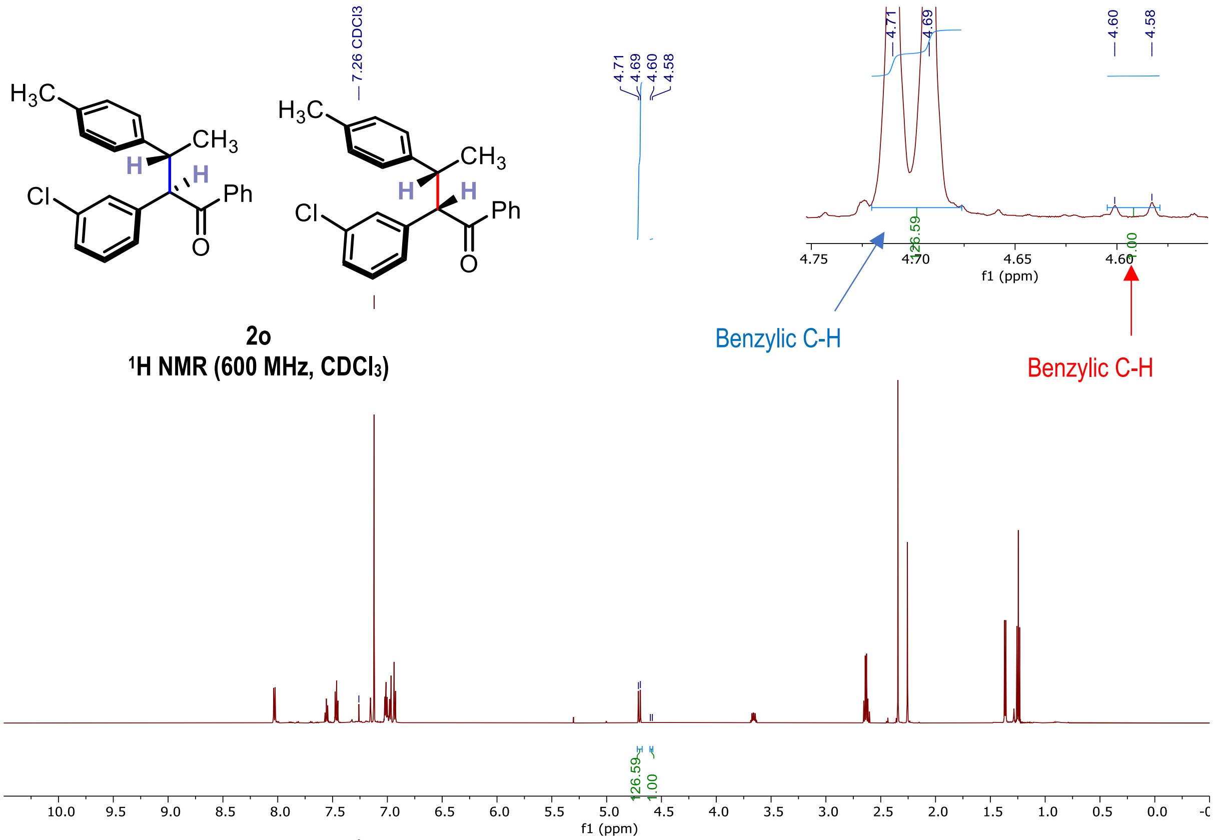

2o
^{1}H NMR (600 MHz, CDCl$_3$)

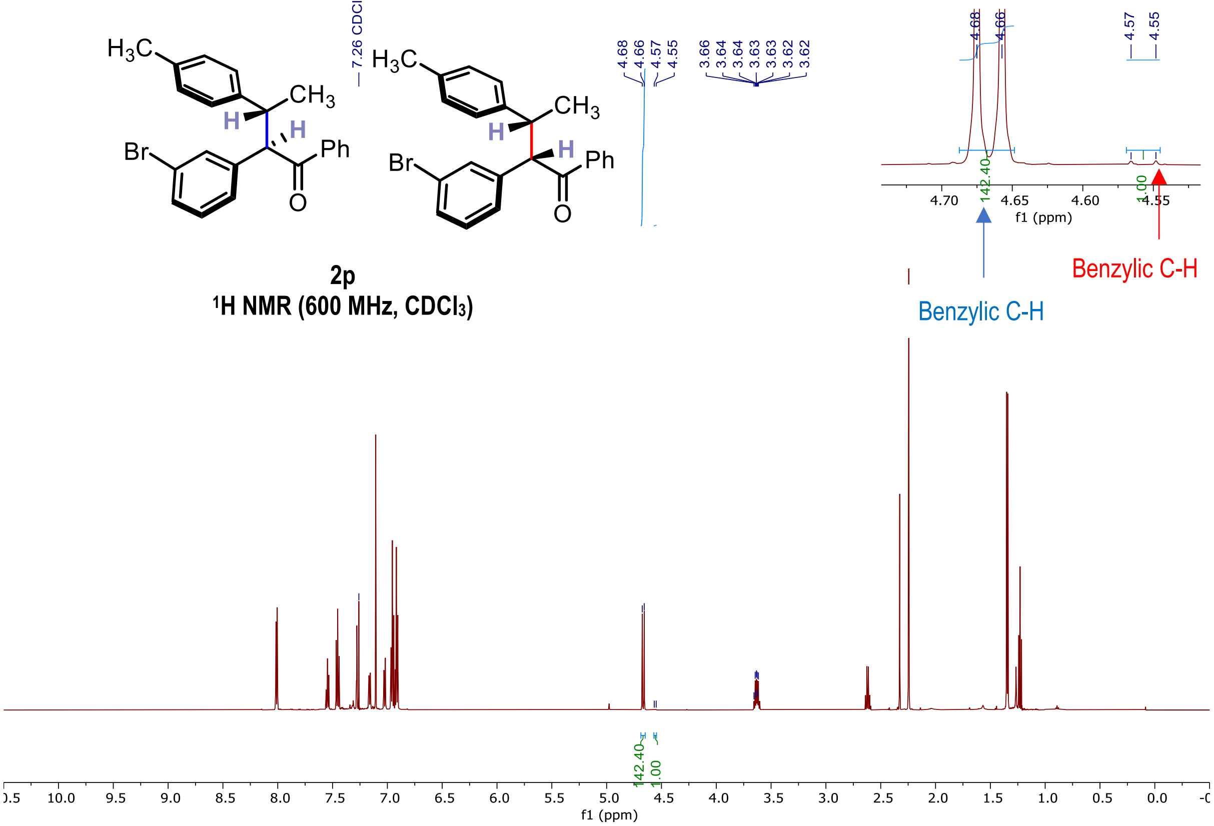

2p
^{1}H NMR (600 MHz, CDCl$_3$)

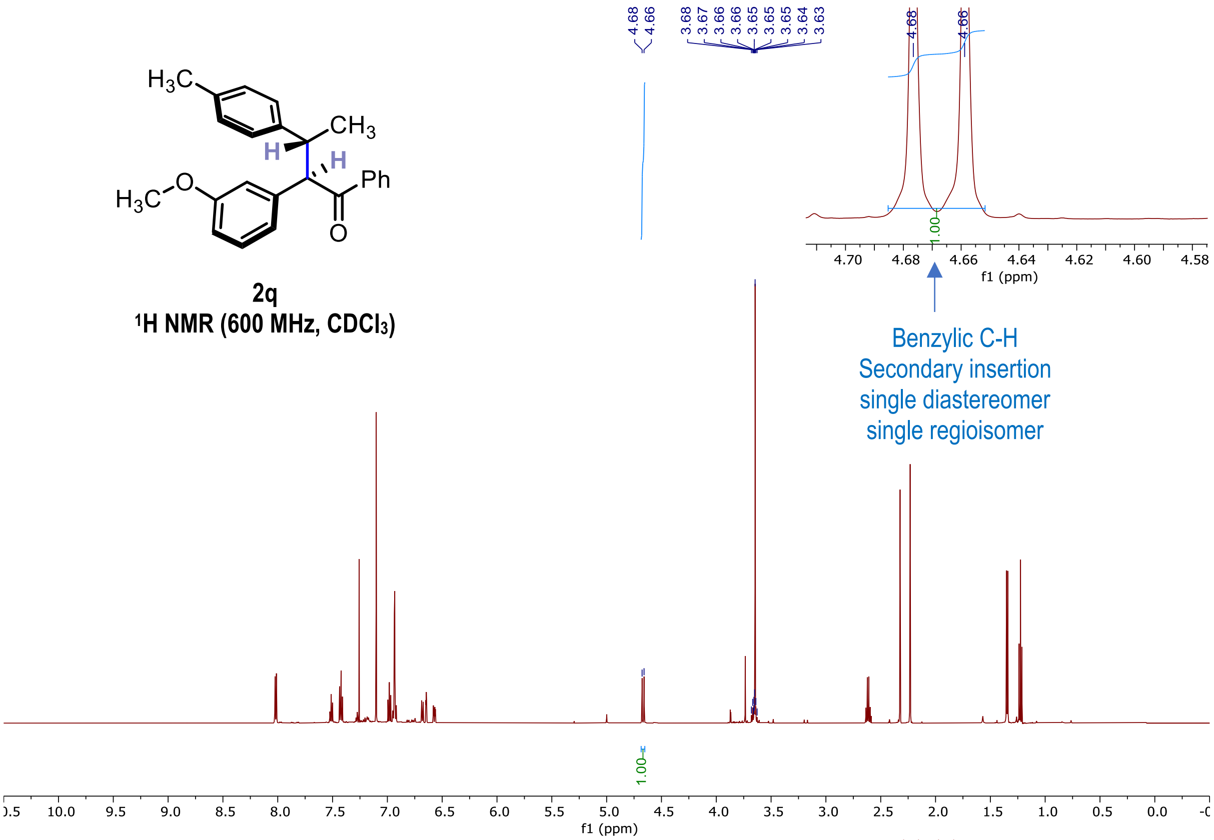

2q
1H NMR (600 MHz, CDCl3)
Benzylic C-H
Secondary insertion
single diastereomer
single regioisomer
f1 (ppm)

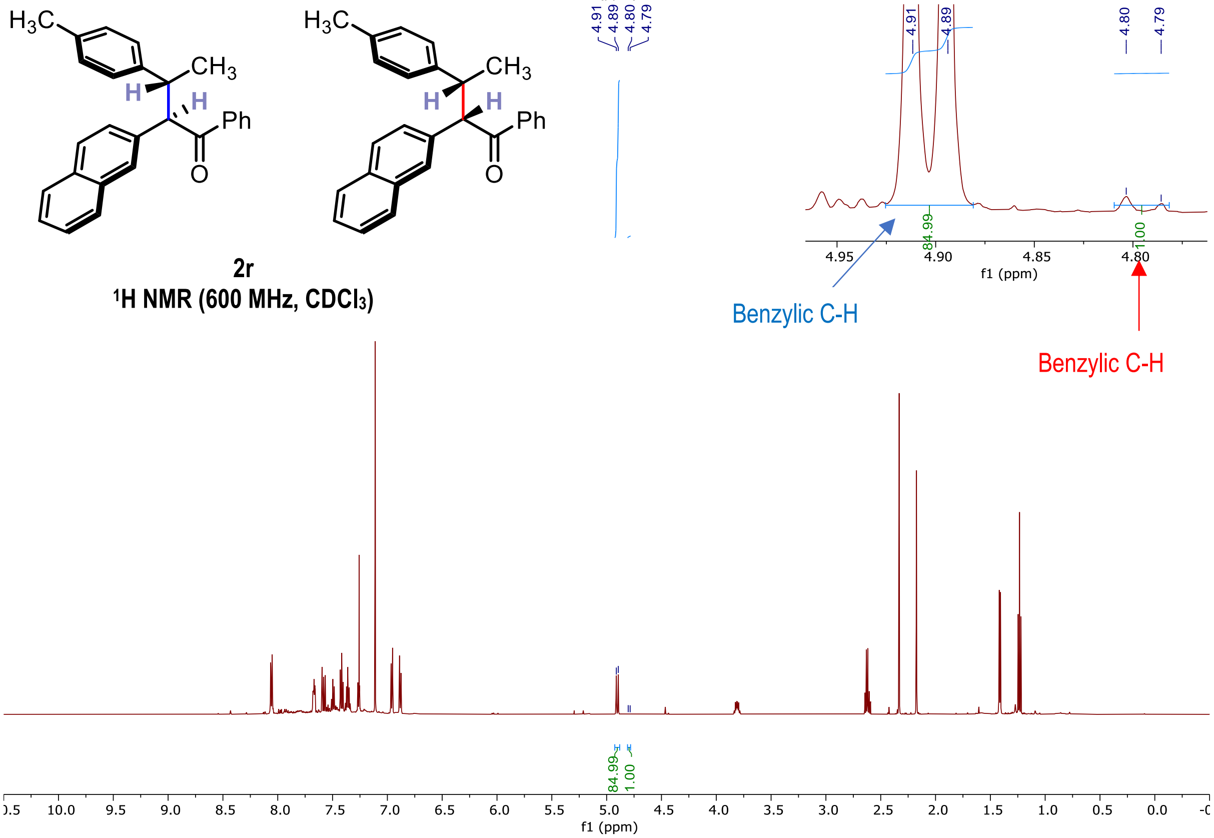

2r
1H NMR (600 MHz, CDCl3)
Benzylic C-H
Benzylic C-H
f1 (ppm)

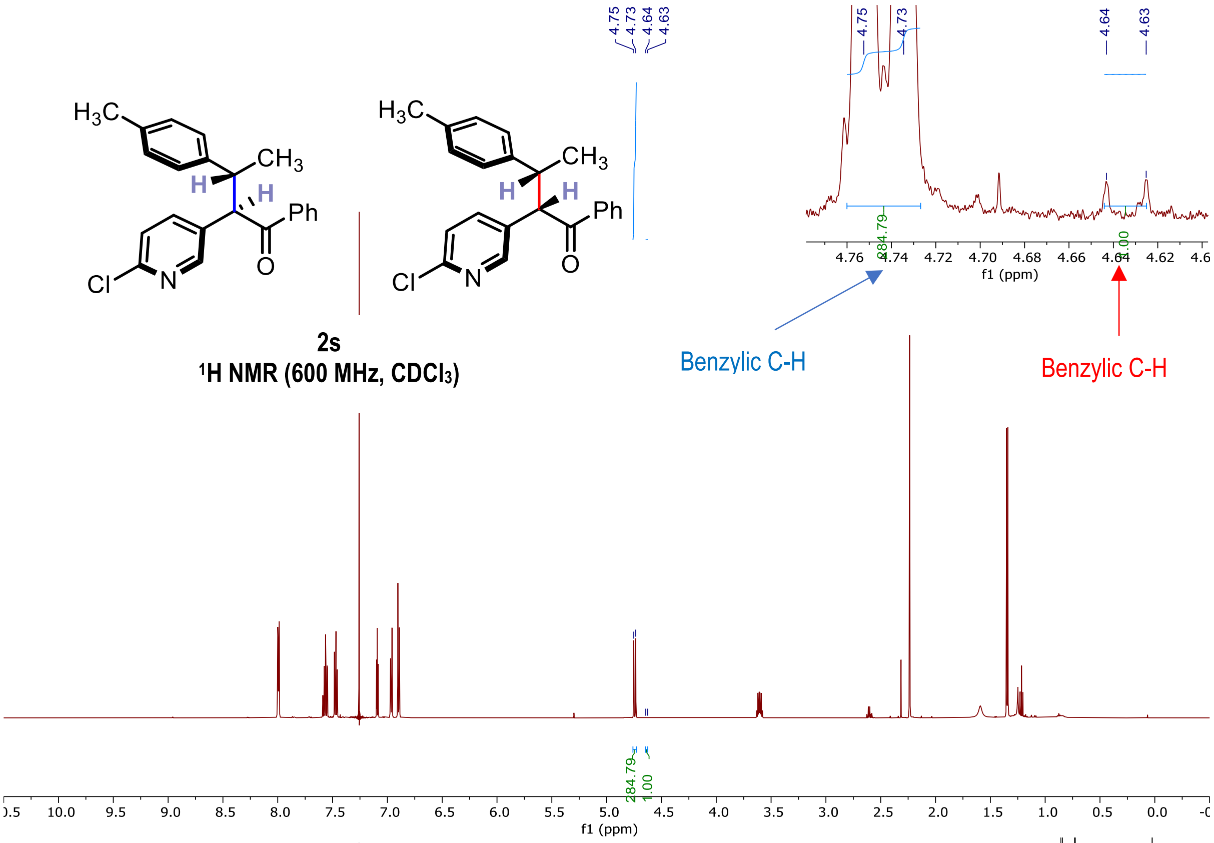

2s
^{1}H NMR (600 MHz, $CDCl_3$)

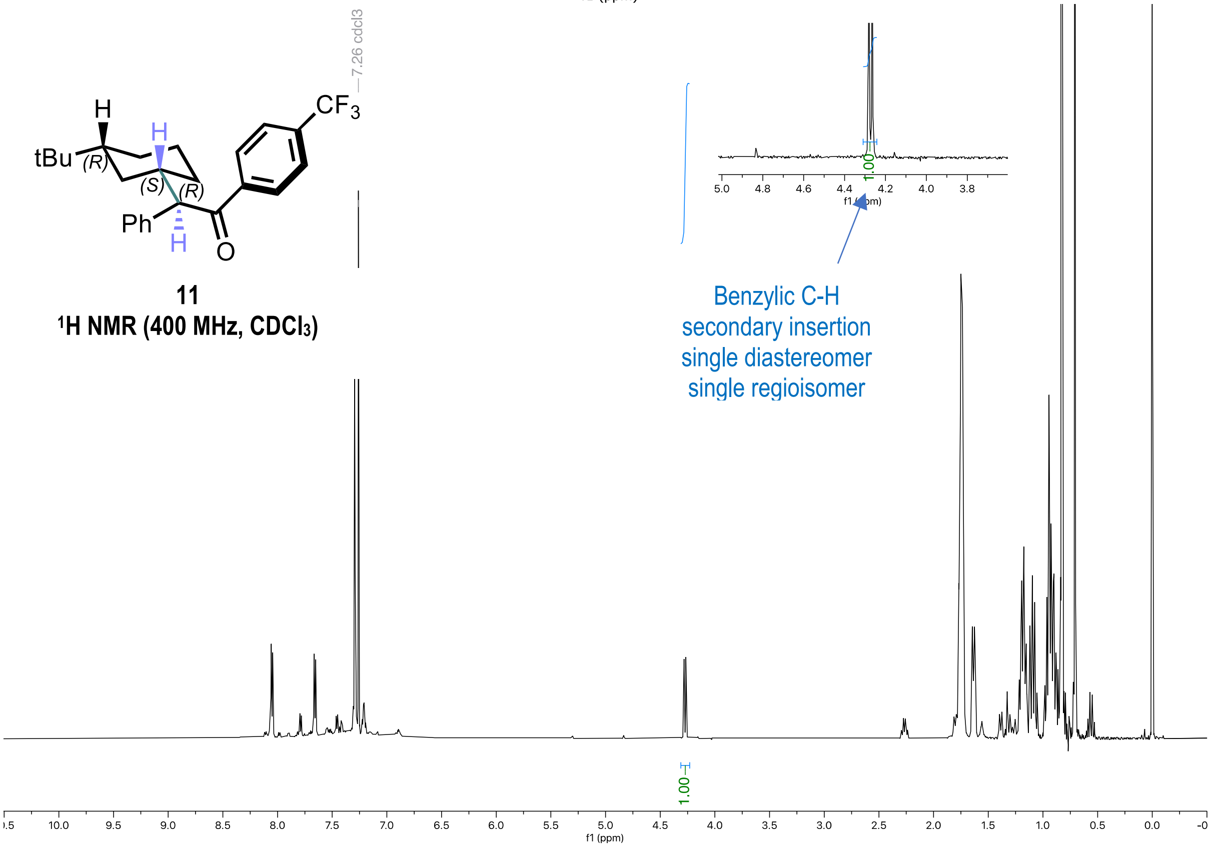

11
^{1}H NMR (400 MHz, $CDCl_3$)

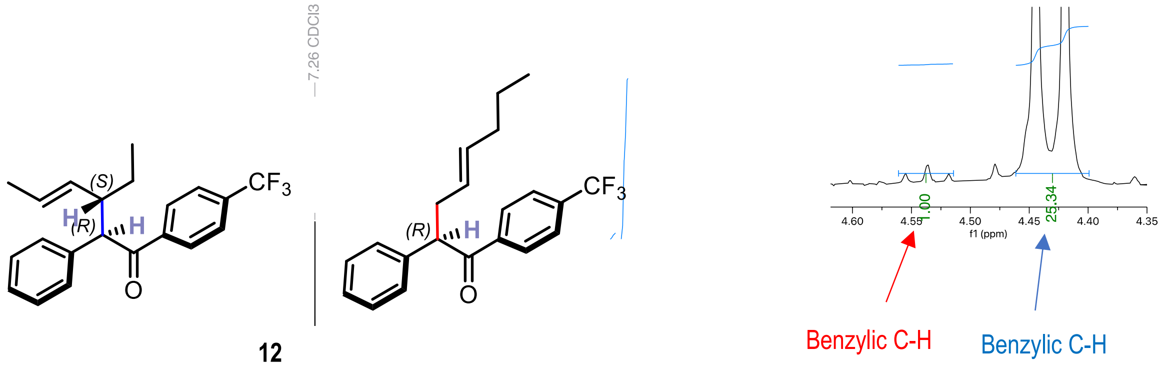

12
^{1}H NMR (600 MHz, $CDCl_3$)

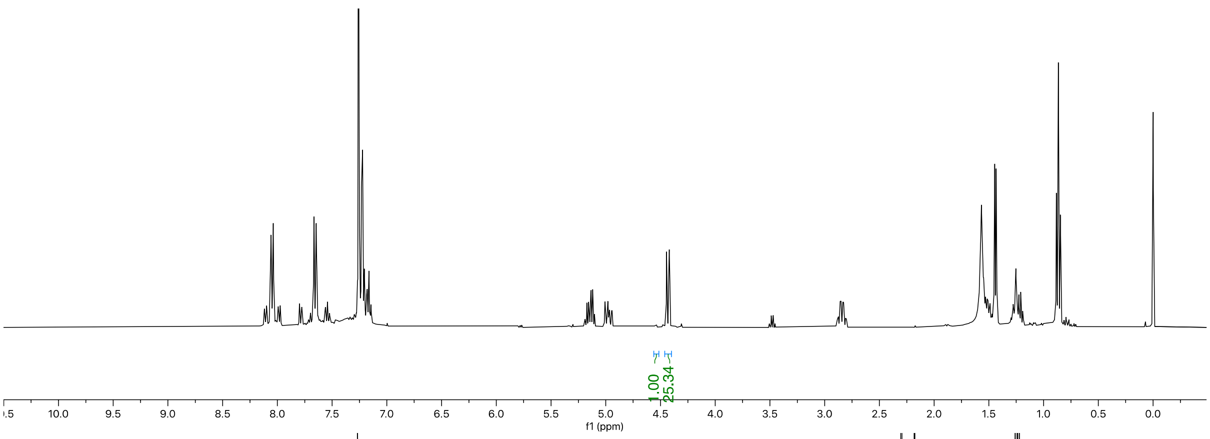

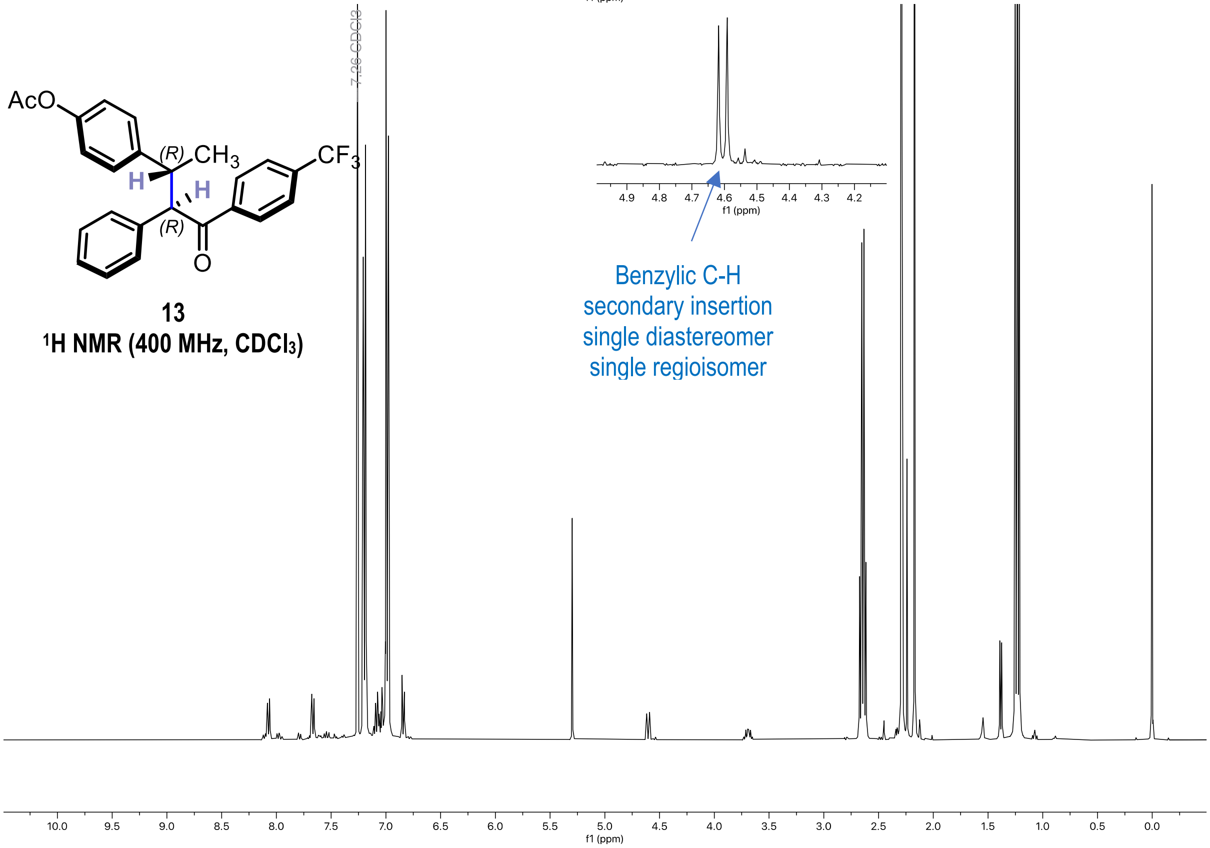

13
^{1}H NMR (400 MHz, $CDCl_3$)

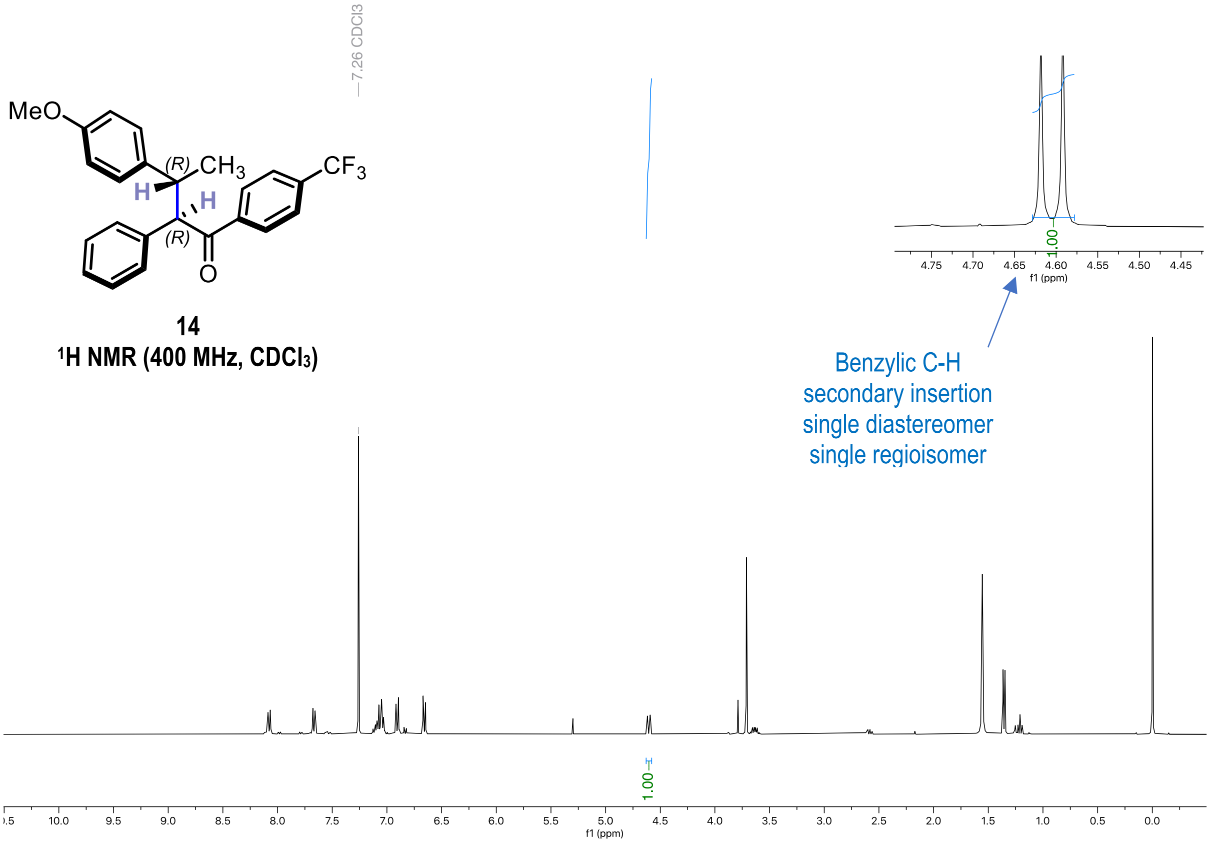

14

^{1}H NMR (400 MHz, $CDCl_3$)

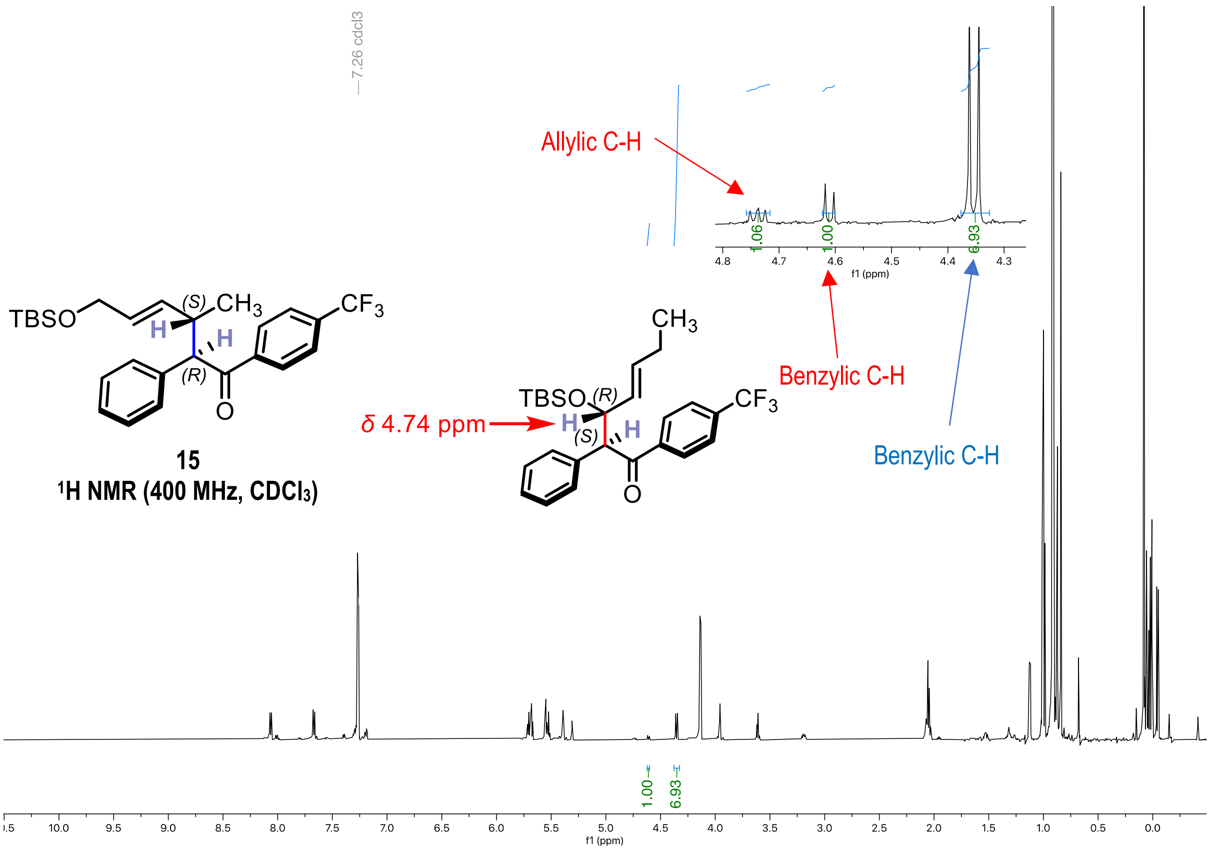

15

^{1}H NMR (400 MHz, $CDCl_3$)

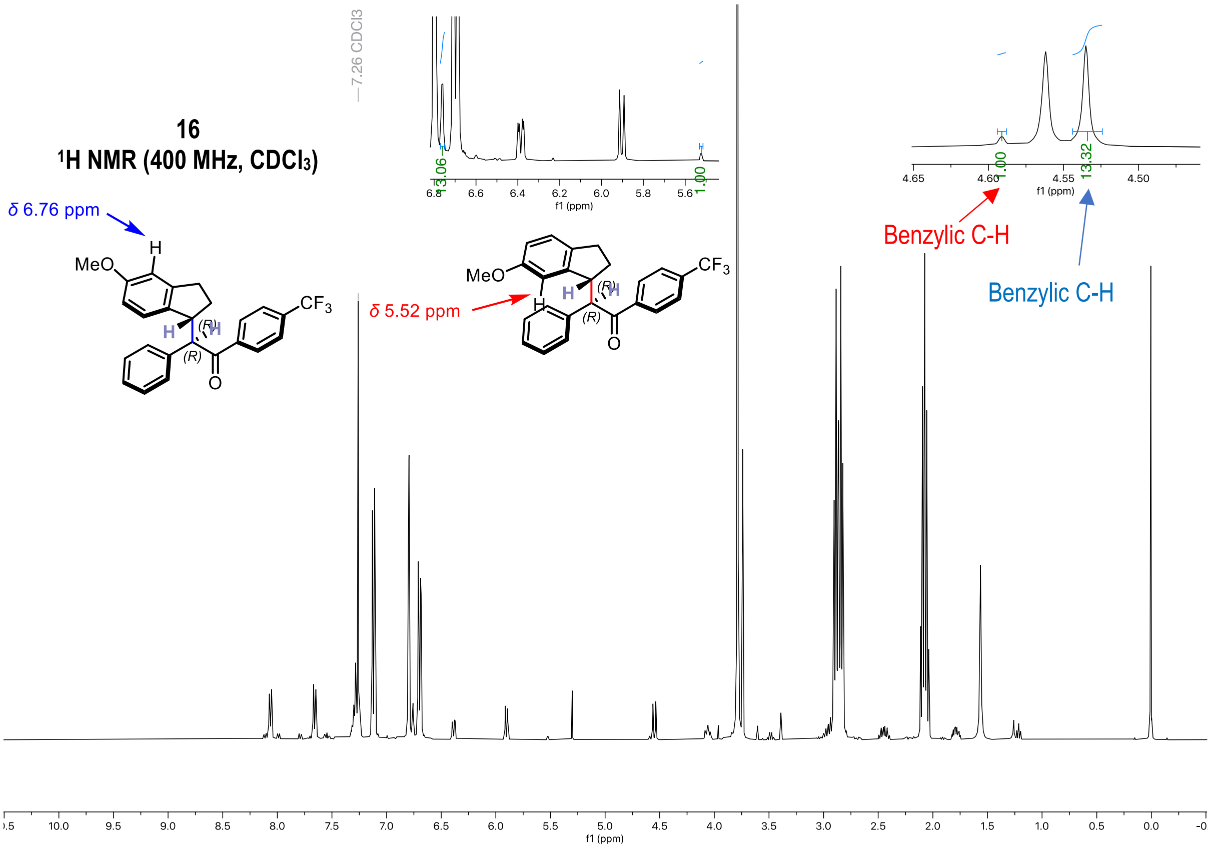
16
¹H NMR (400 MHz, CDCl₃)
δ 6.76 ppm
δ 5.52 ppm
7.26 CDCl3
Benzylic C-H
Benzylic C-H
f1 (ppm)

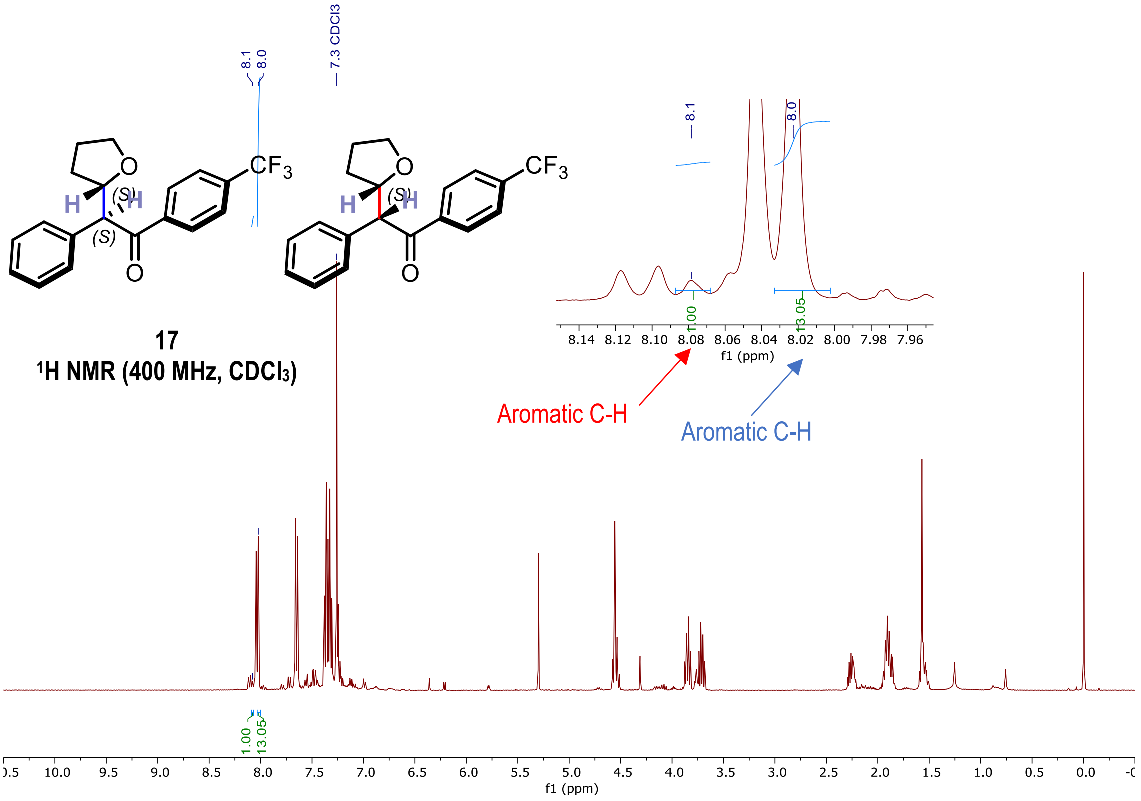
17
¹H NMR (400 MHz, CDCl₃)
7.3 CDCl3
Aromatic C-H
Aromatic C-H
f1 (ppm)

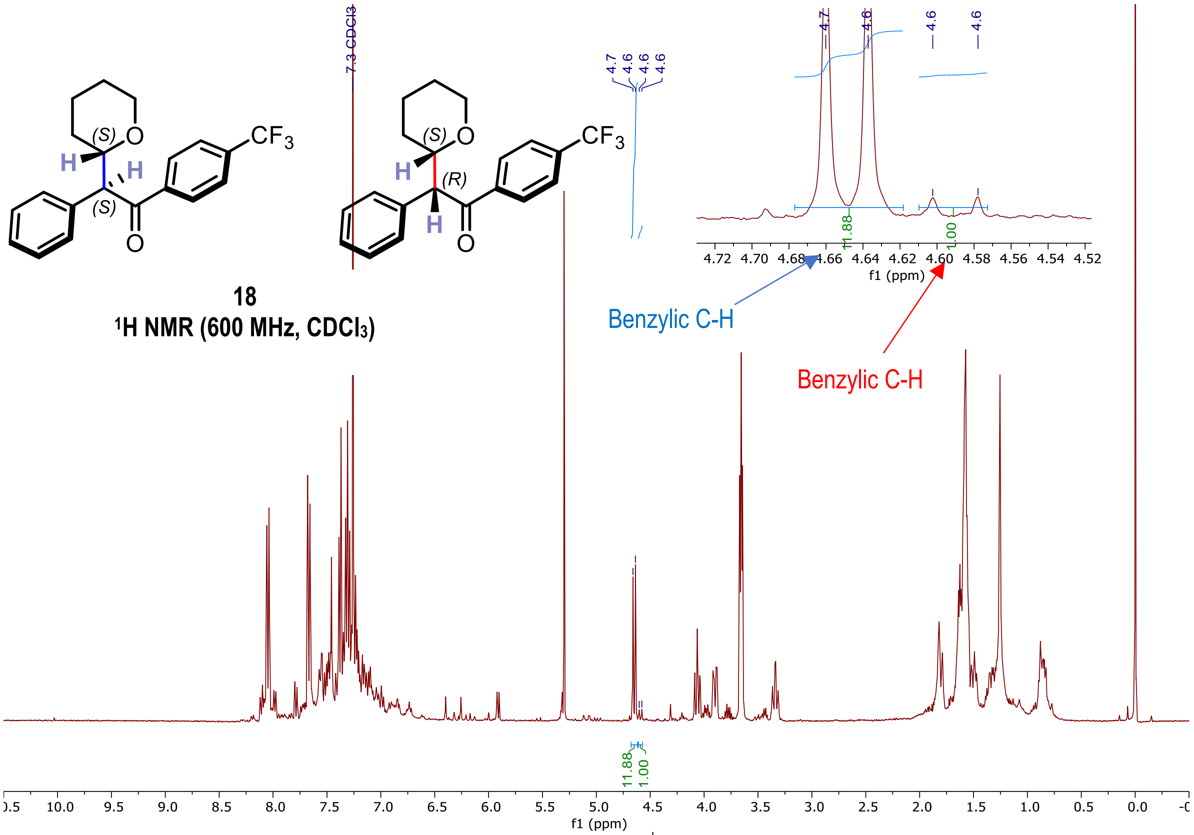
18
¹H NMR (600 MHz, CDCl₃)
7.3 CDCl3
Benzylic C-H
Benzylic C-H
11.88
1.00
f1 (ppm)

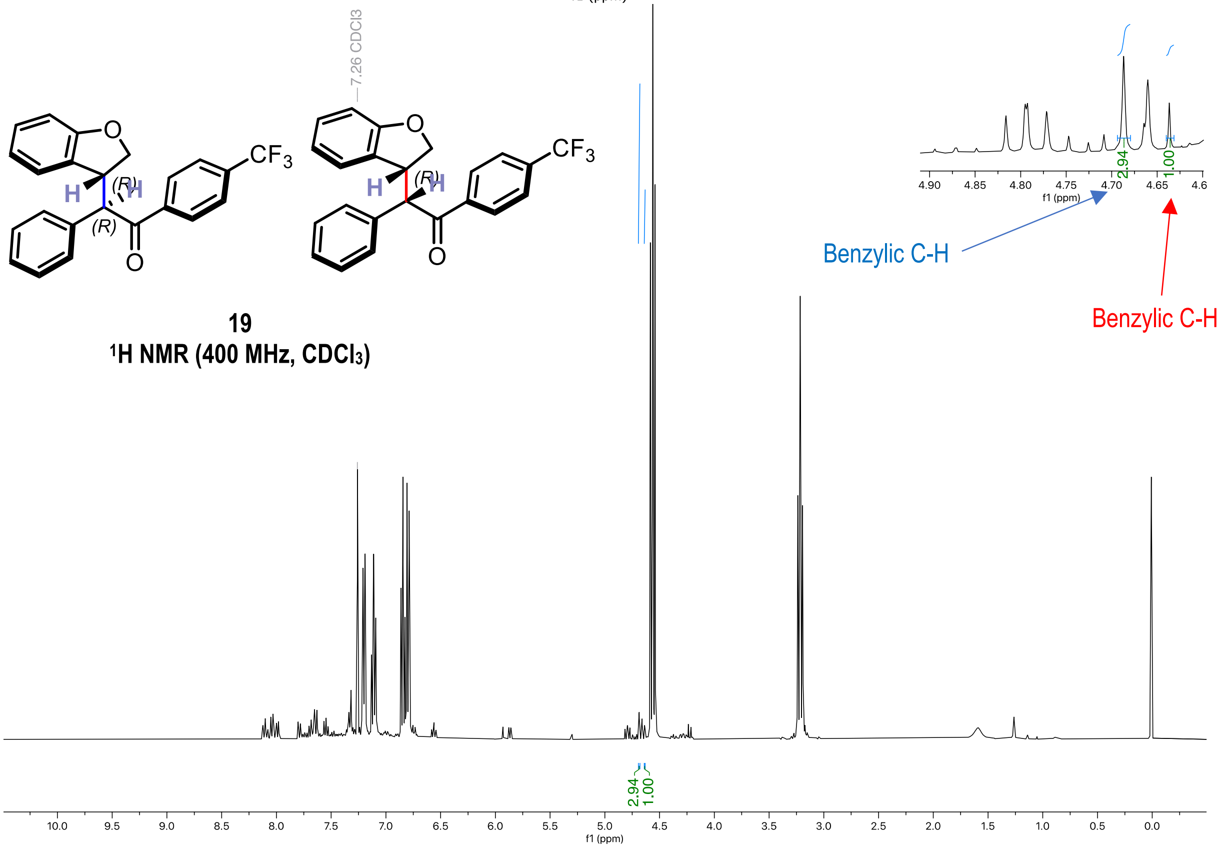
19
¹H NMR (400 MHz, CDCl₃)
7.26 CDCl3
Benzylic C-H
Benzylic C-H
2.94
1.00
f1 (ppm)

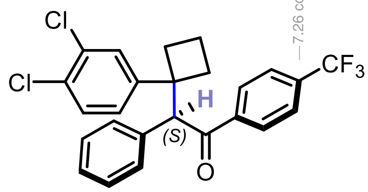

20

^{1}H NMR (400 MHz, $CDCl_3$)

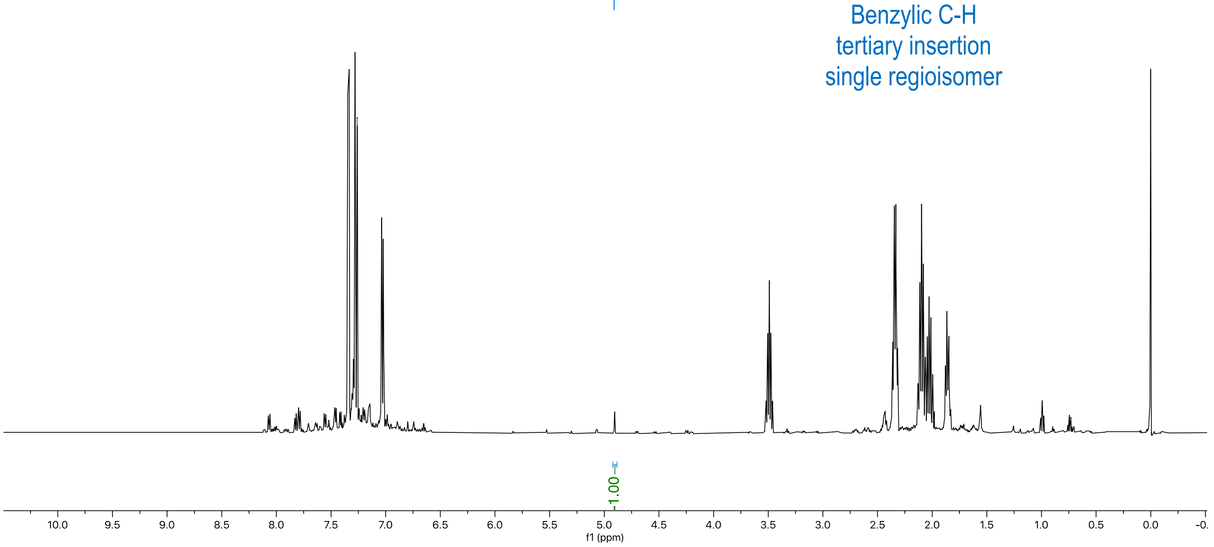

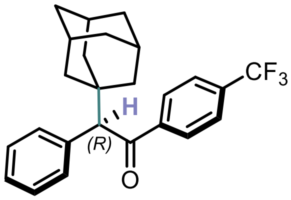

23

^{1}H NMR (400 MHz, $CDCl_3$)

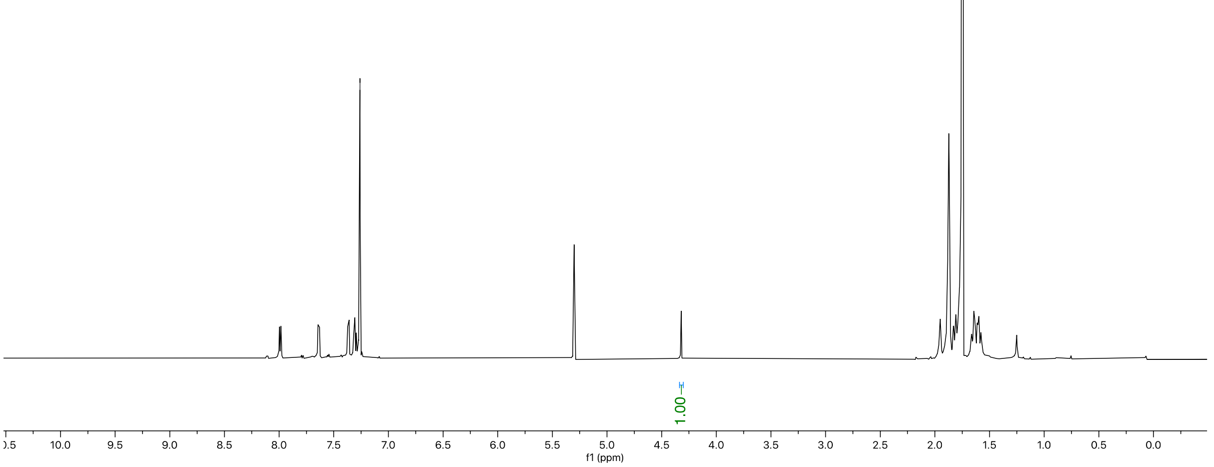

6. NMR Spectra

Absolute configuration: the absolute configuration is tentatively assigned by analogy based on X-ray crystallography. The minor diastereomer/regioisomer stereoconfiguration is a relative stereoconfiguration; the absolute stereoconfiguration is not known of the minor diastereomers/regioisomers.

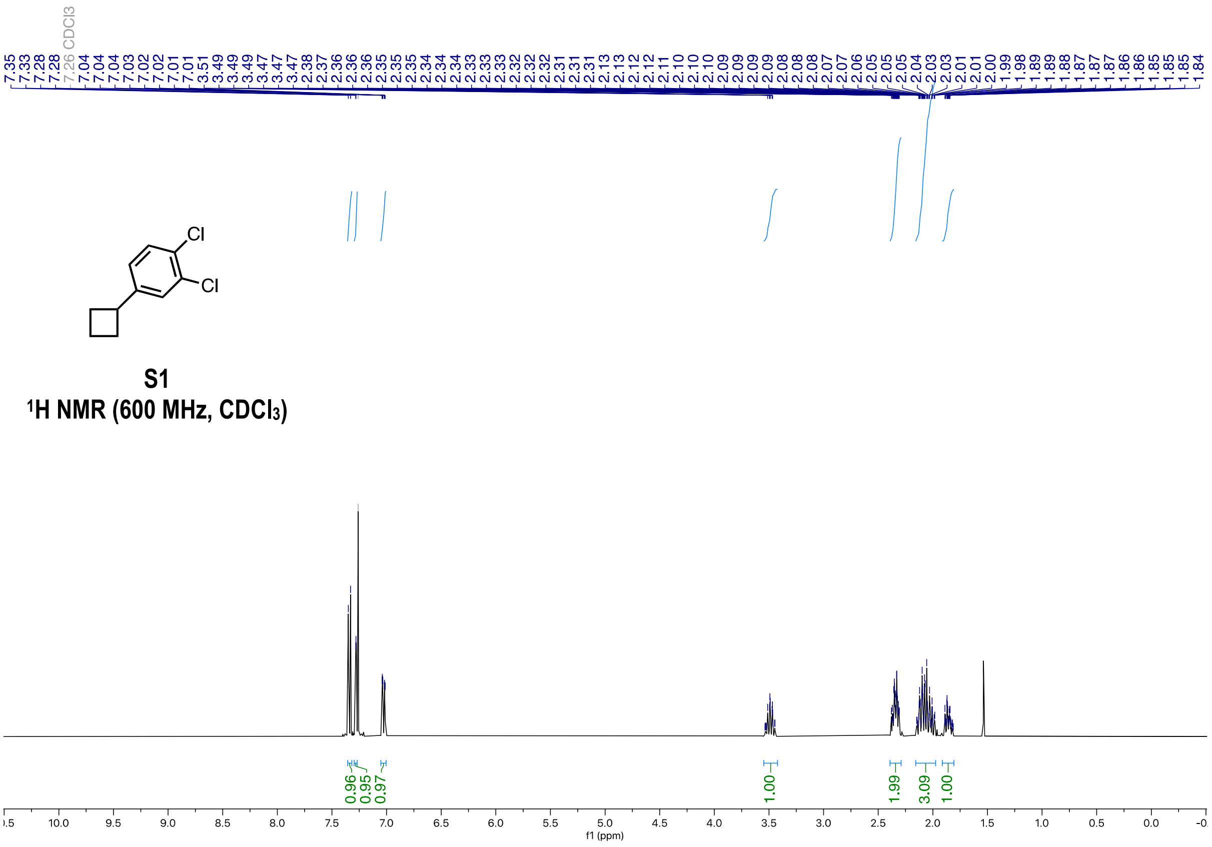

S1

^{1}H NMR (600 MHz, $CDCl_3$)

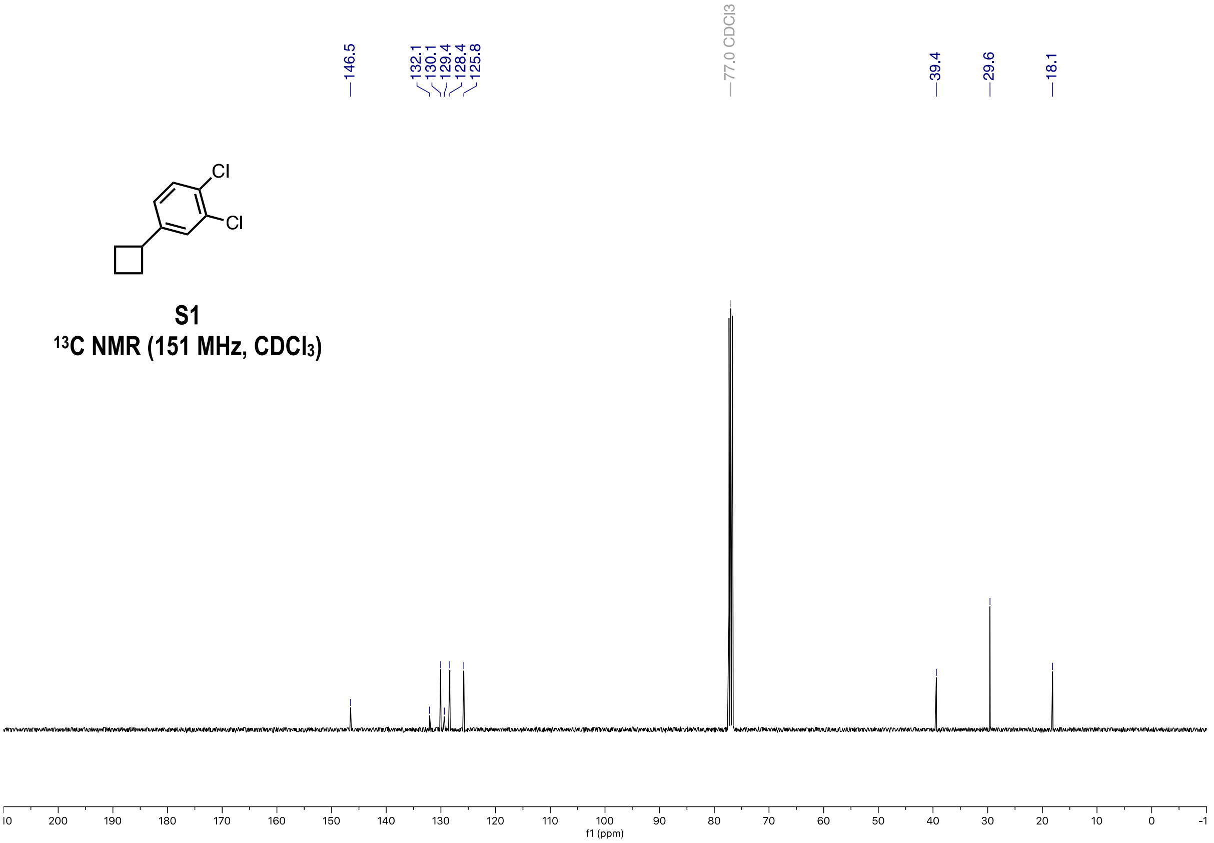

S1
^{13}C NMR (151 MHz, $CDCl_3$)

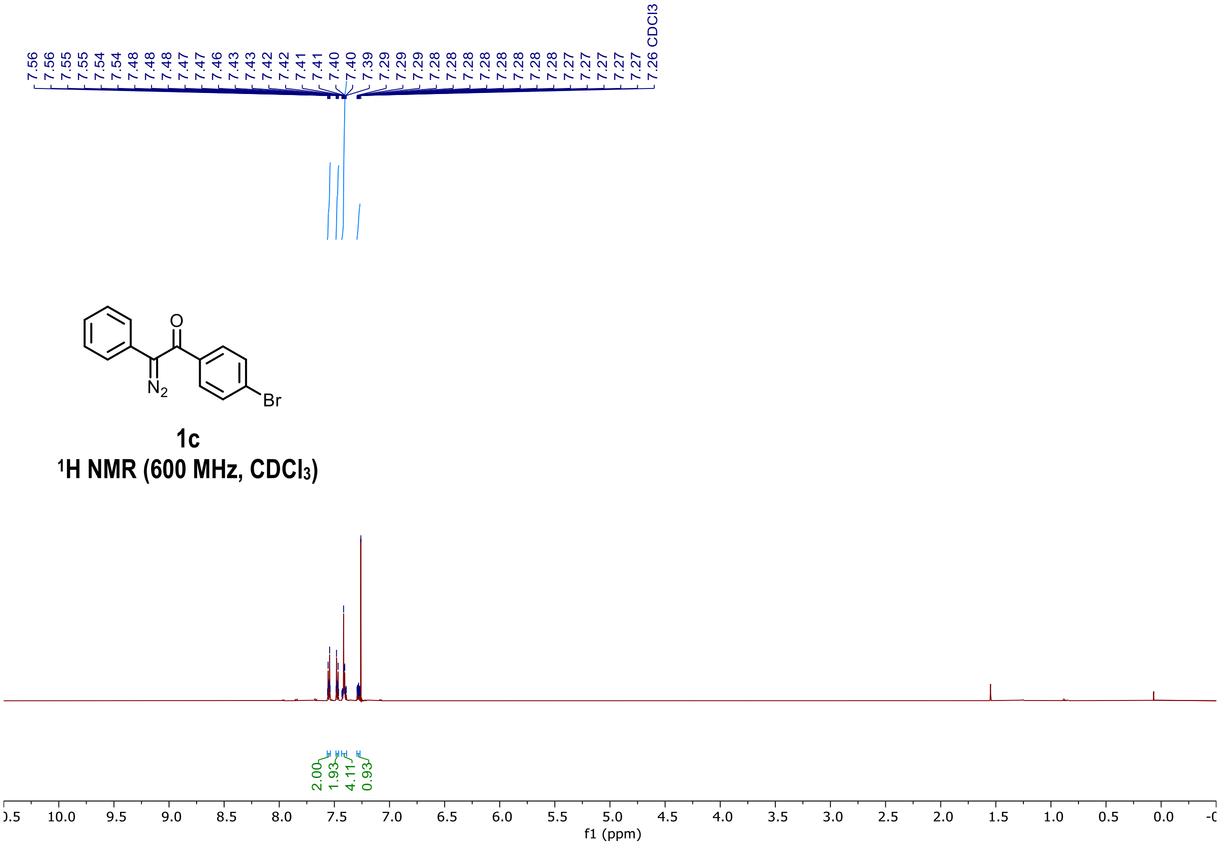

1c
^{1}H NMR (600 MHz, $CDCl_3$)

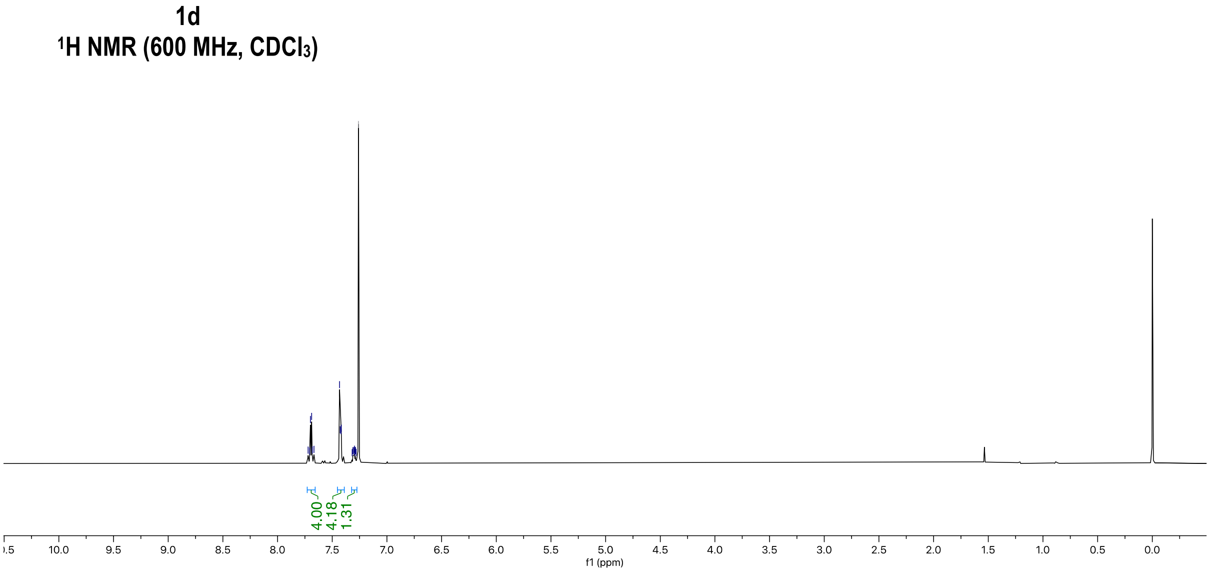

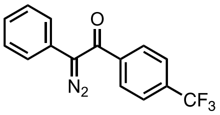

1d

^{1}H NMR (600 MHz, $CDCl_3$)

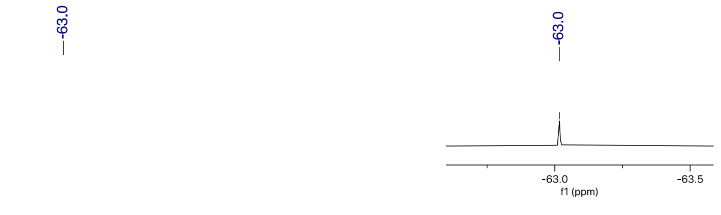

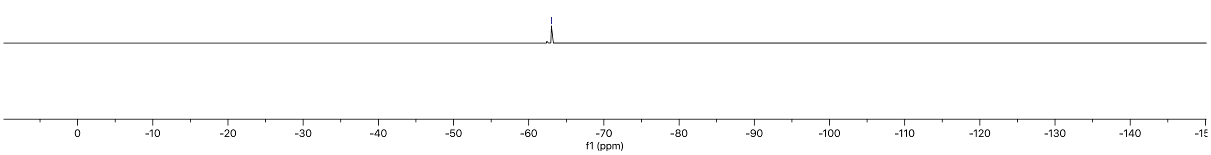

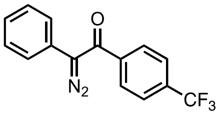

1d

^{19}F NMR (376 MHz, $CDCl_3$)

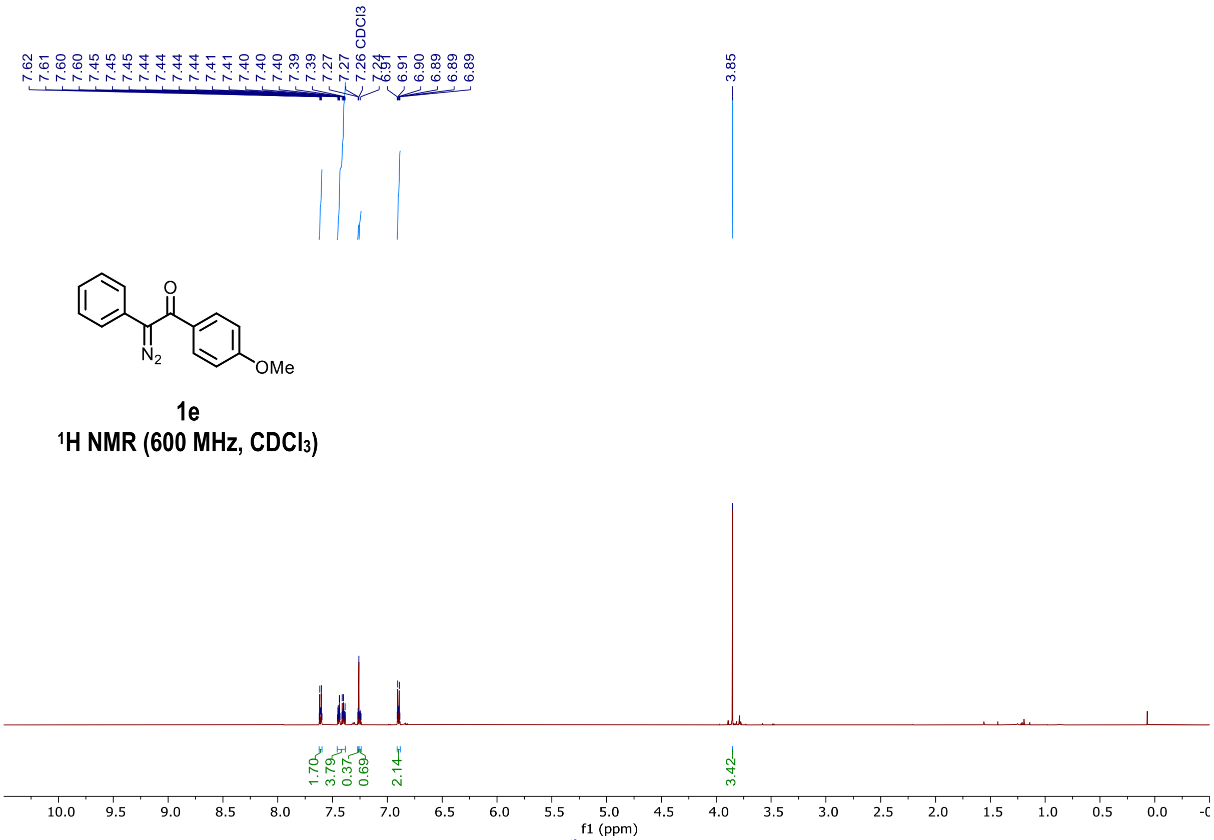

1e

^{1}H NMR (600 MHz, $CDCl_3$)

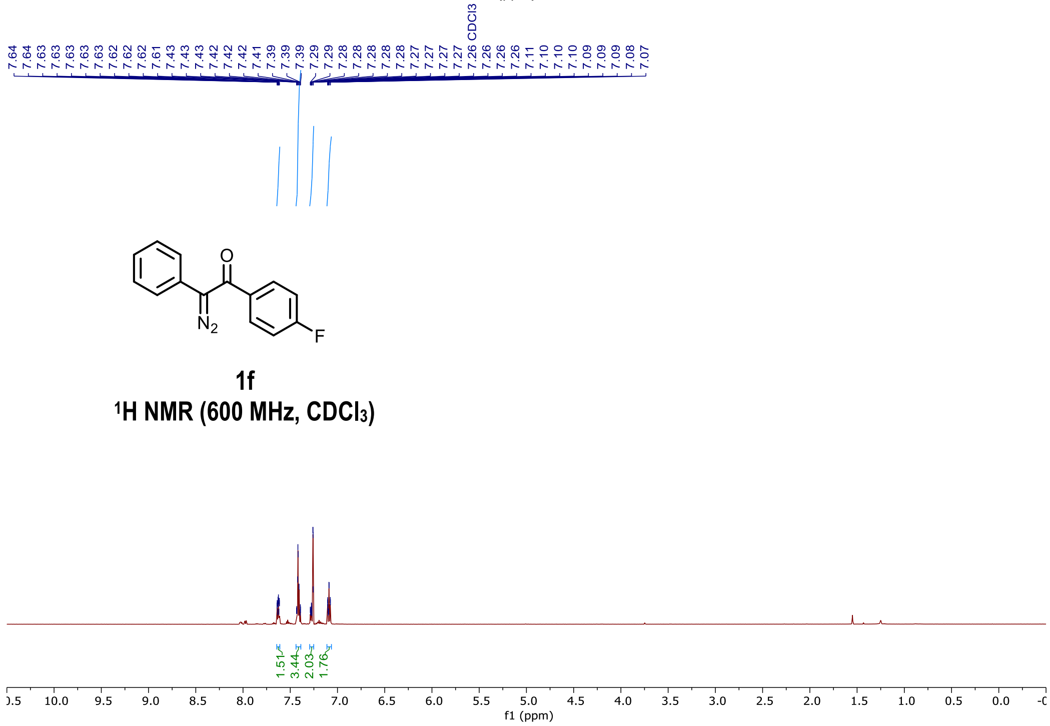

1f

^{1}H NMR (600 MHz, $CDCl_3$)

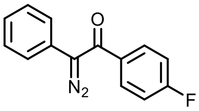

1f
^{19}F NMR (376 MHz, C_6D_6)

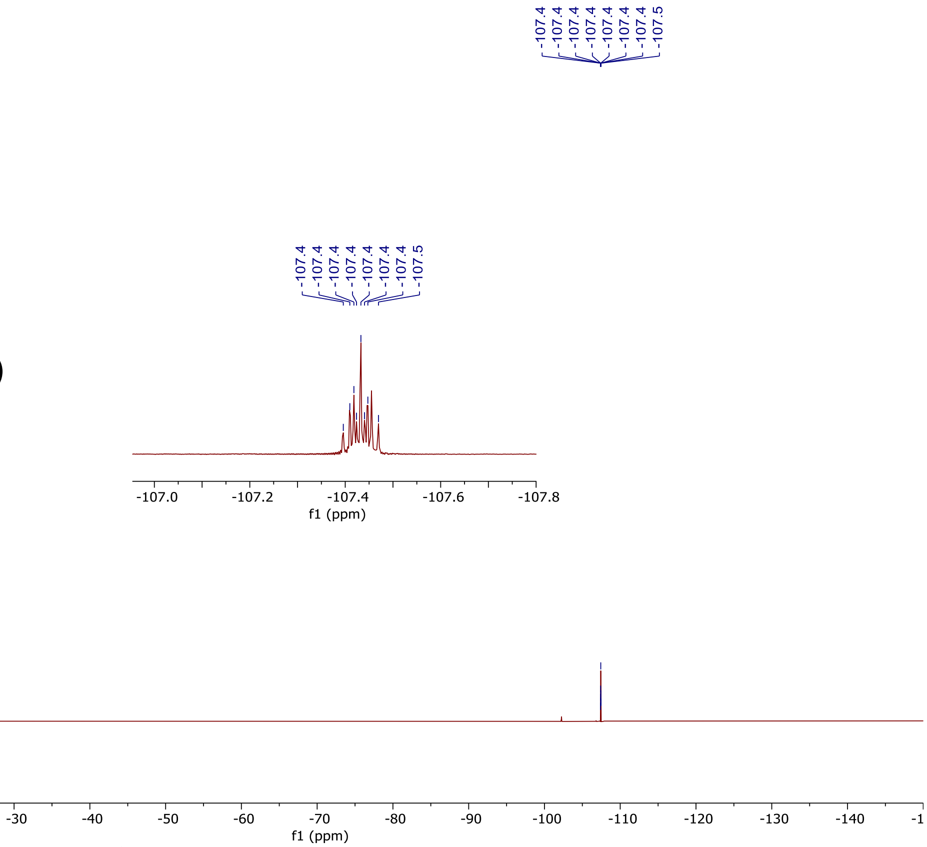

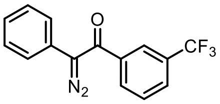

1g
^{1}H NMR (400 MHz, C_6D_6)

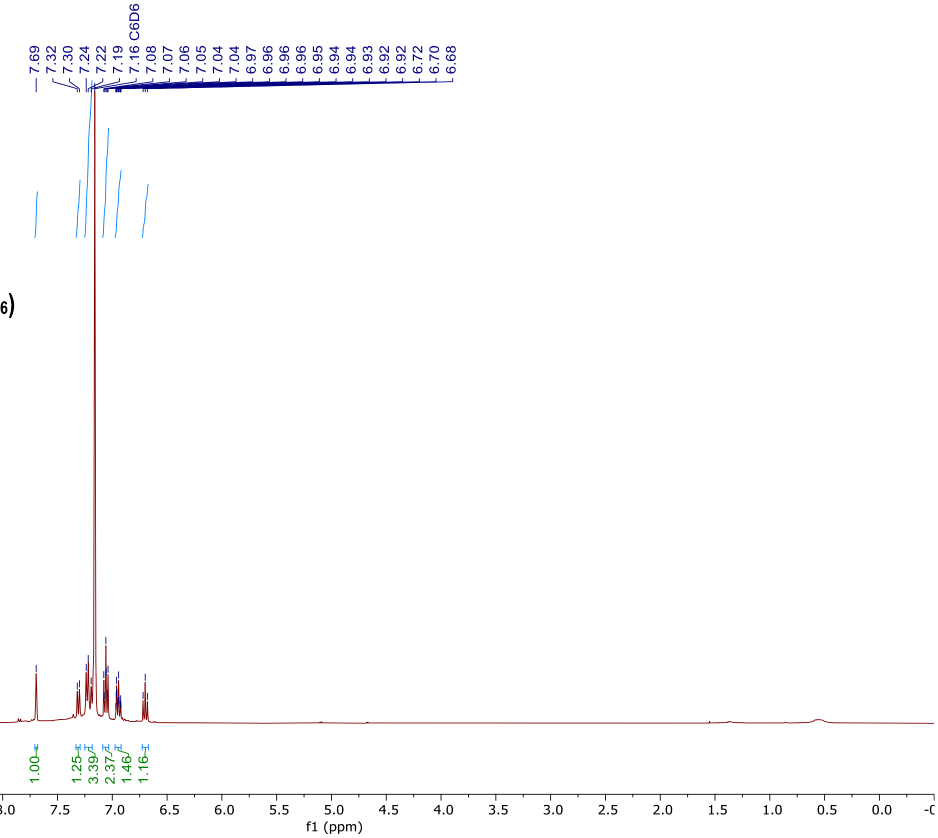

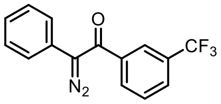

1g

^{19}F NMR (376 MHz, C_6D_6)

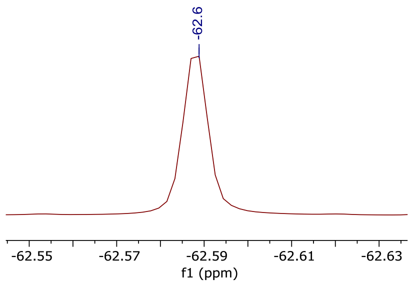

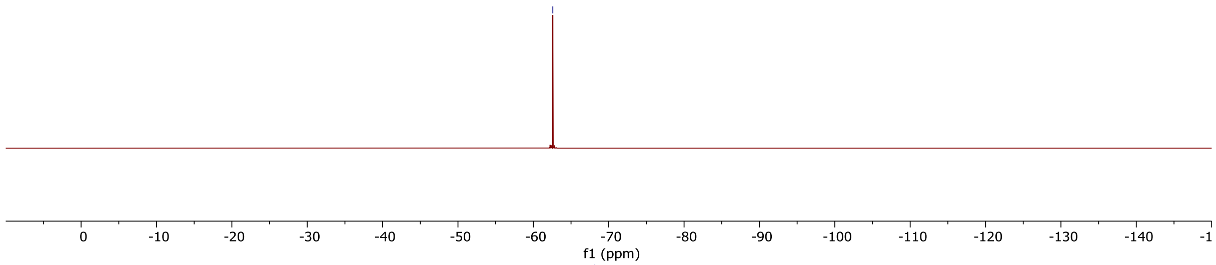

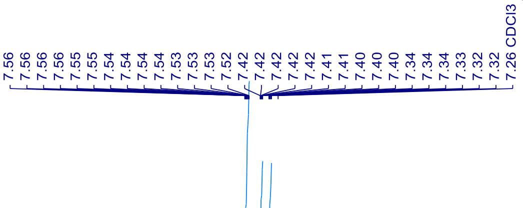

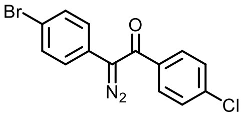

1h

^{1}H NMR (600 MHz, $CDCl_3$)

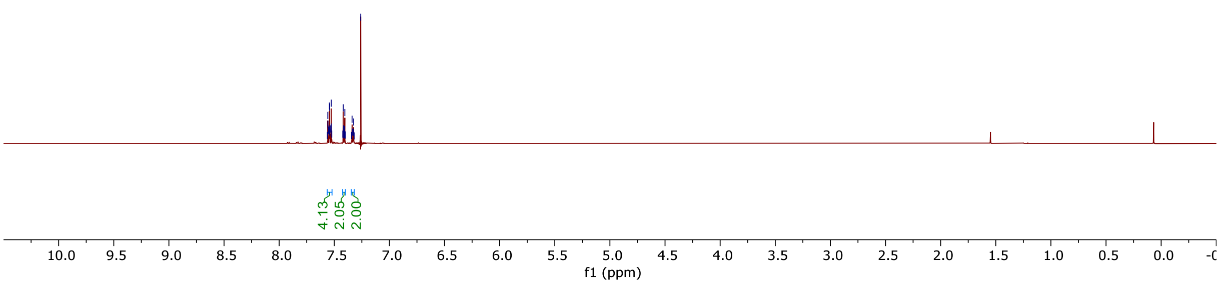

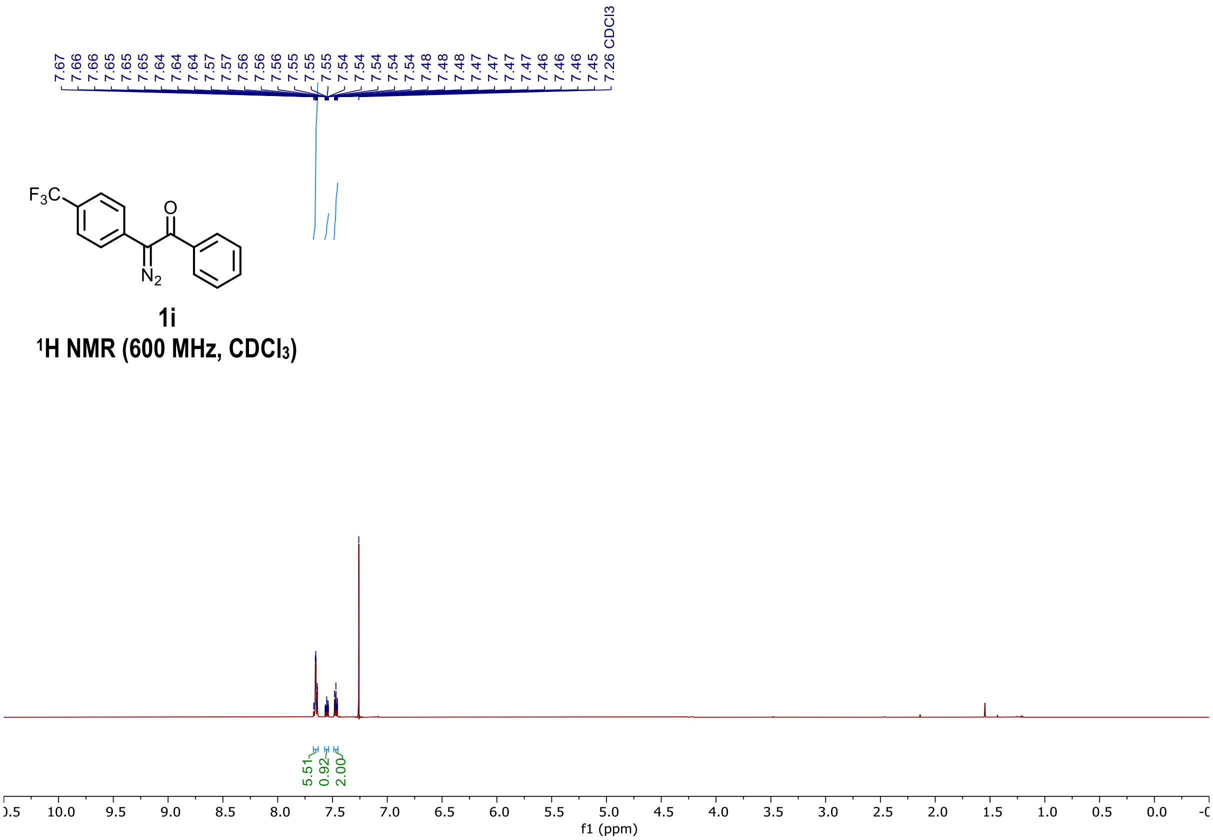

1i
^{1}H NMR (600 MHz, $CDCl_3$)

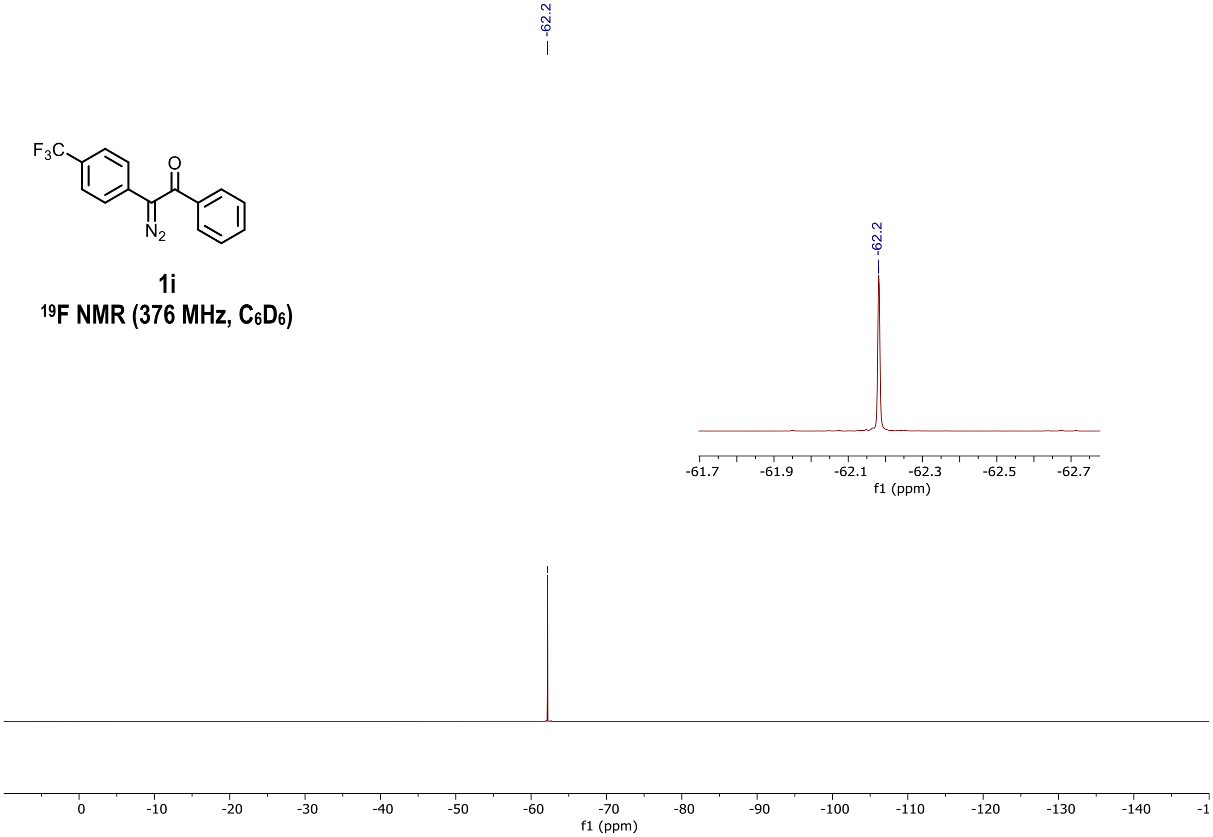

1i
^{19}F NMR (376 MHz, C_6D_6)

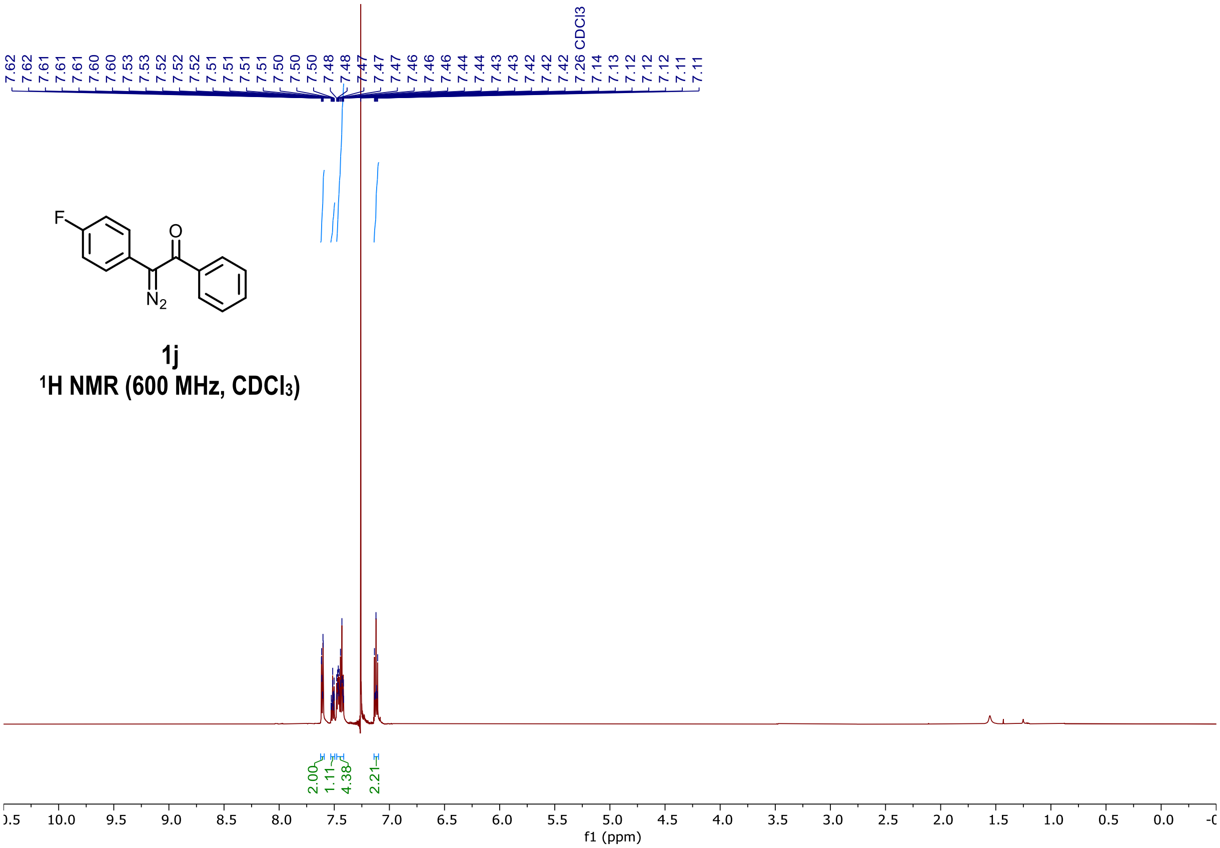

1j

^{1}H NMR (600 MHz, $CDCl_3$)

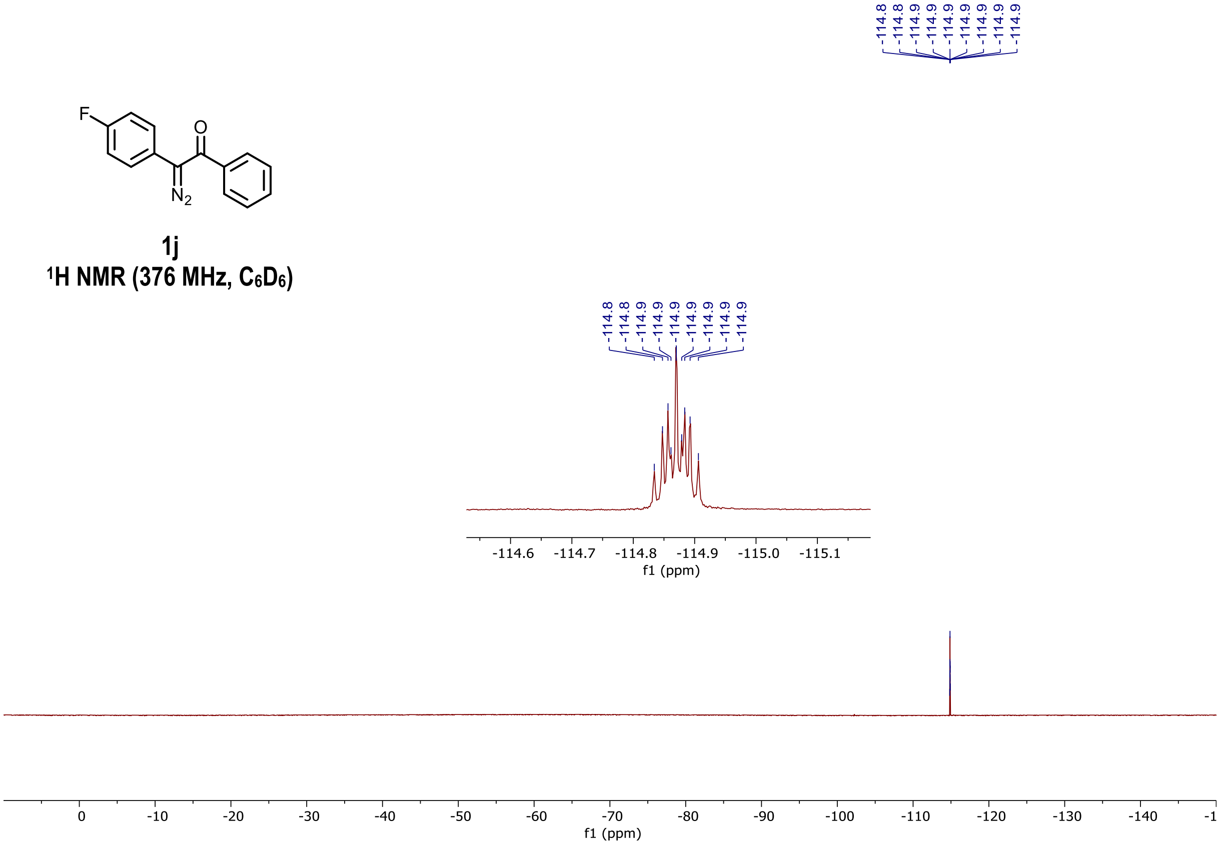

1j

^{1}H NMR (376 MHz, C_6D_6)

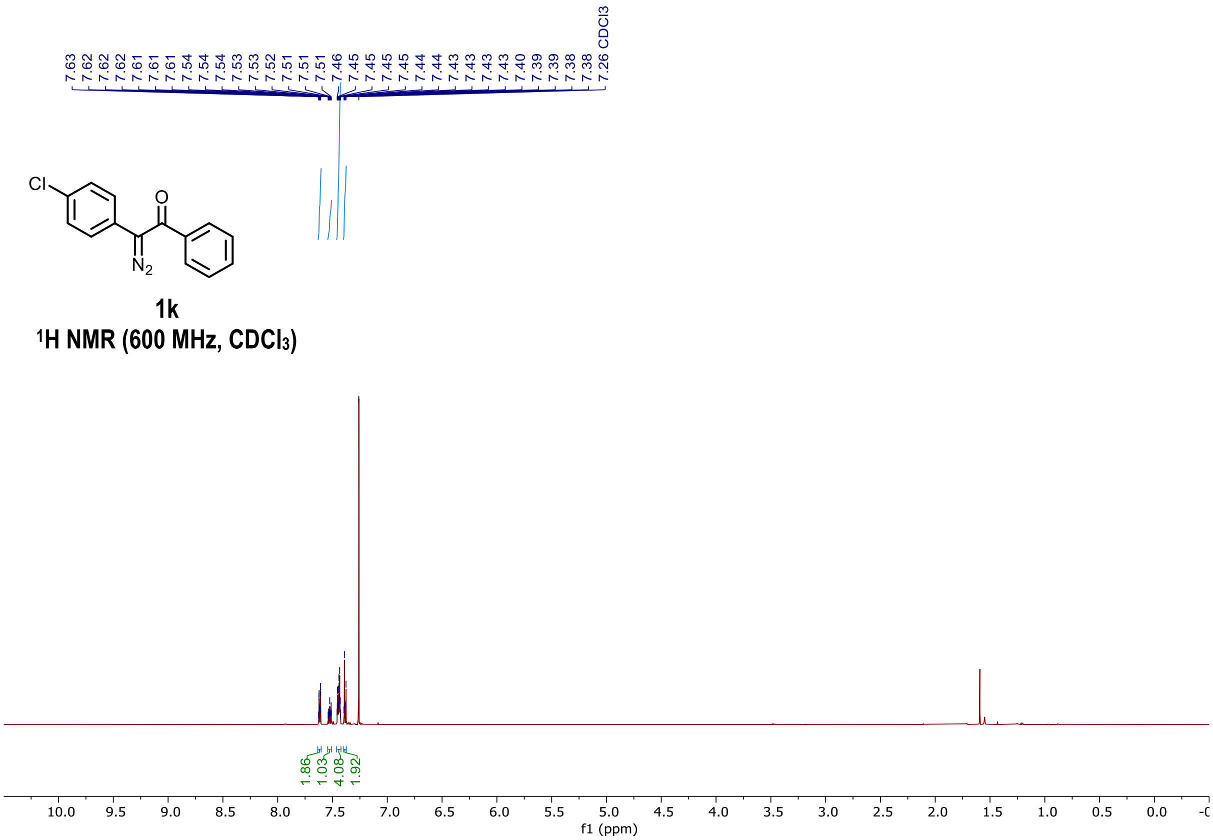

1k

^{1}H NMR (600 MHz, $CDCl_3$)

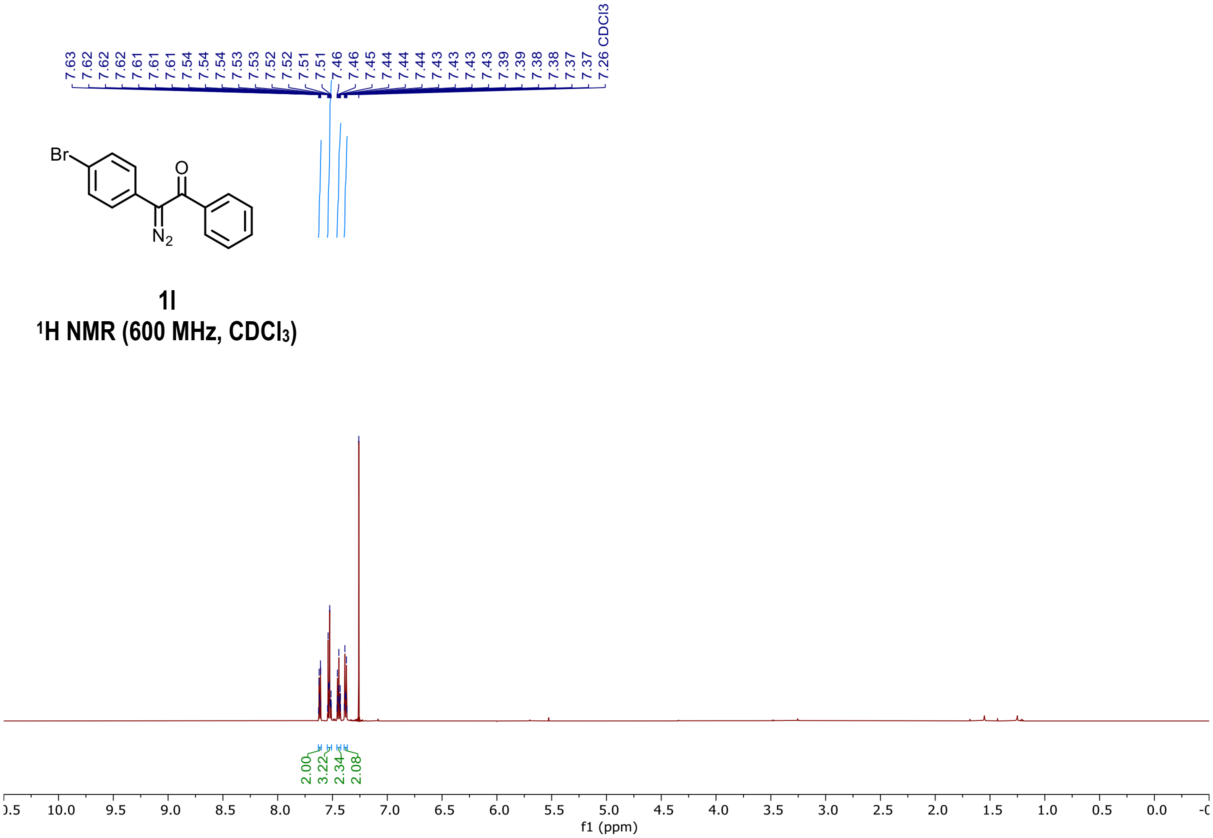

1l

^{1}H NMR (600 MHz, $CDCl_3$)

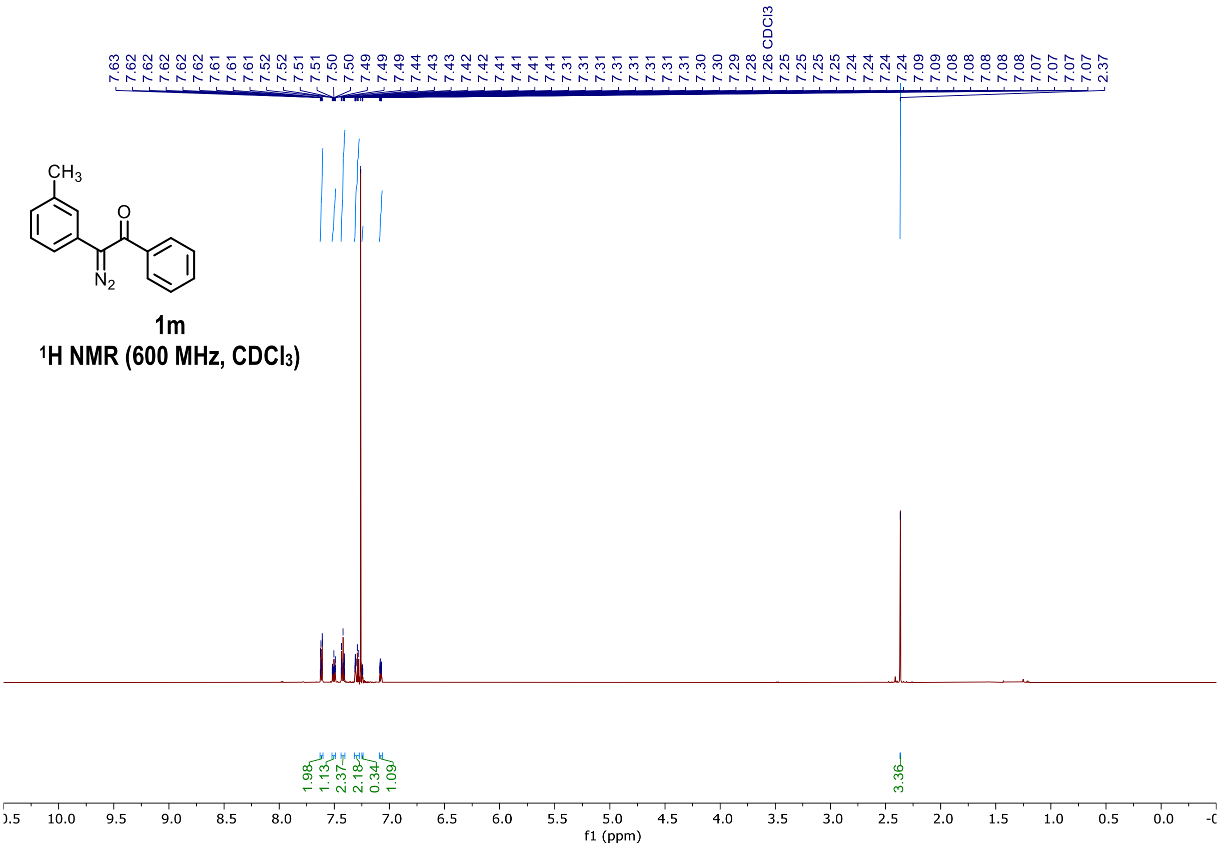
7.63 7.62 7.62 7.62 7.62 7.61 7.61 7.61 7.52 7.52 7.51 7.51 7.50 7.50 7.49 7.49 7.44 7.43 7.43 7.42 7.42 7.41 7.41 7.41 7.31 7.31 7.31 7.31 7.31 7.31 7.31 7.30 7.30 7.29 7.28 7.26 CDCl3 7.25 7.25 7.25 7.25 7.24 7.24 7.24 7.24 7.09 7.09 7.08 7.08 7.08 7.08 7.08 7.07 7.07 7.07 7.07 2.37
CH3
O
N2
1m
1H NMR (600 MHz, CDCl3)
1.98
1.13
2.37
2.18
0.34
1.09
3.36
f1 (ppm)

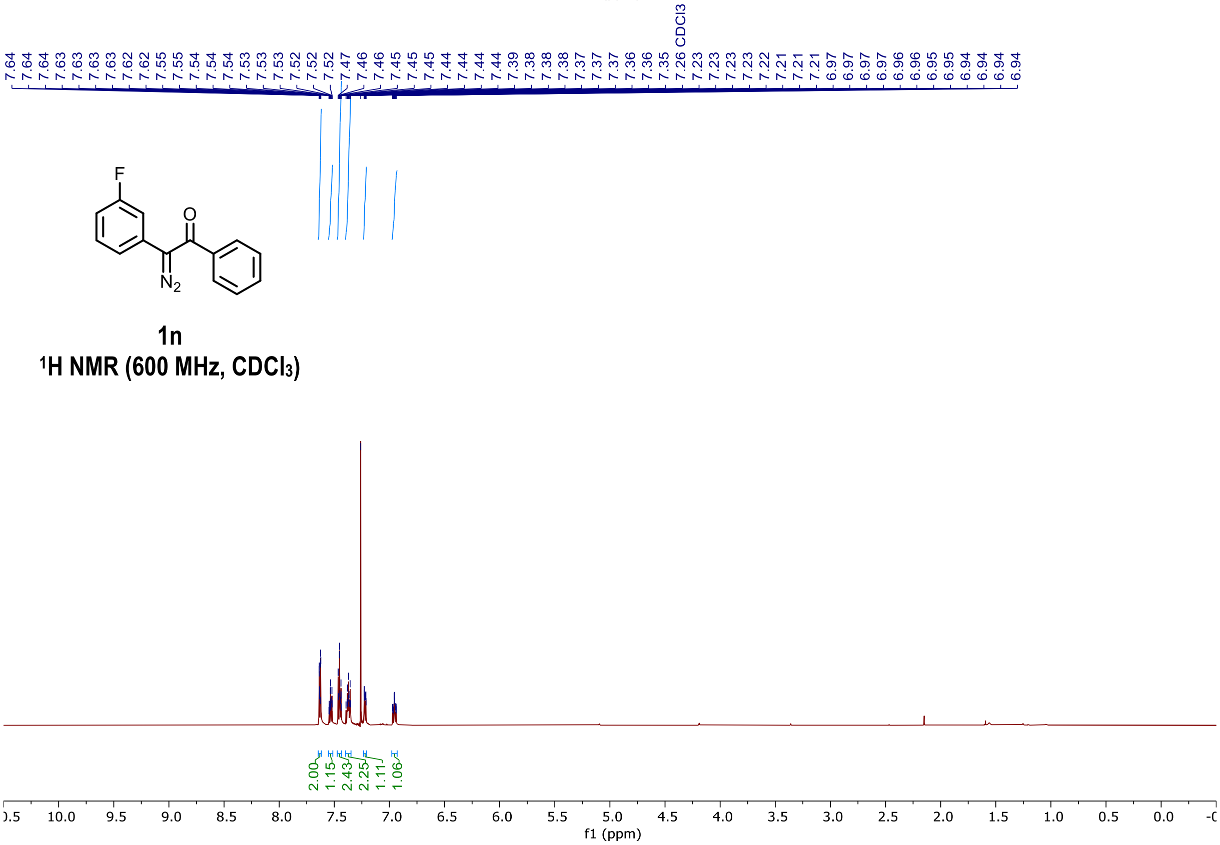
7.64 7.64 7.64 7.63 7.63 7.63 7.62 7.62 7.55 7.55 7.54 7.54 7.54 7.53 7.53 7.52 7.52 7.52 7.47 7.46 7.46 7.45 7.45 7.44 7.44 7.44 7.39 7.38 7.38 7.37 7.37 7.36 7.36 7.35 7.26 CDCl3 7.23 7.23 7.23 7.23 7.22 7.21 7.21 7.21 6.97 6.97 6.97 6.97 6.96 6.95 6.95 6.94 6.94 6.94 6.94
F
O
N2
1n
1H NMR (600 MHz, CDCl3)
2.00
1.15
2.43
2.25
1.11
1.06
f1 (ppm)

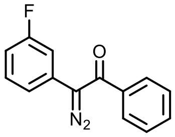

1n
^{19}F NMR (376 MHz, C_6D_6)

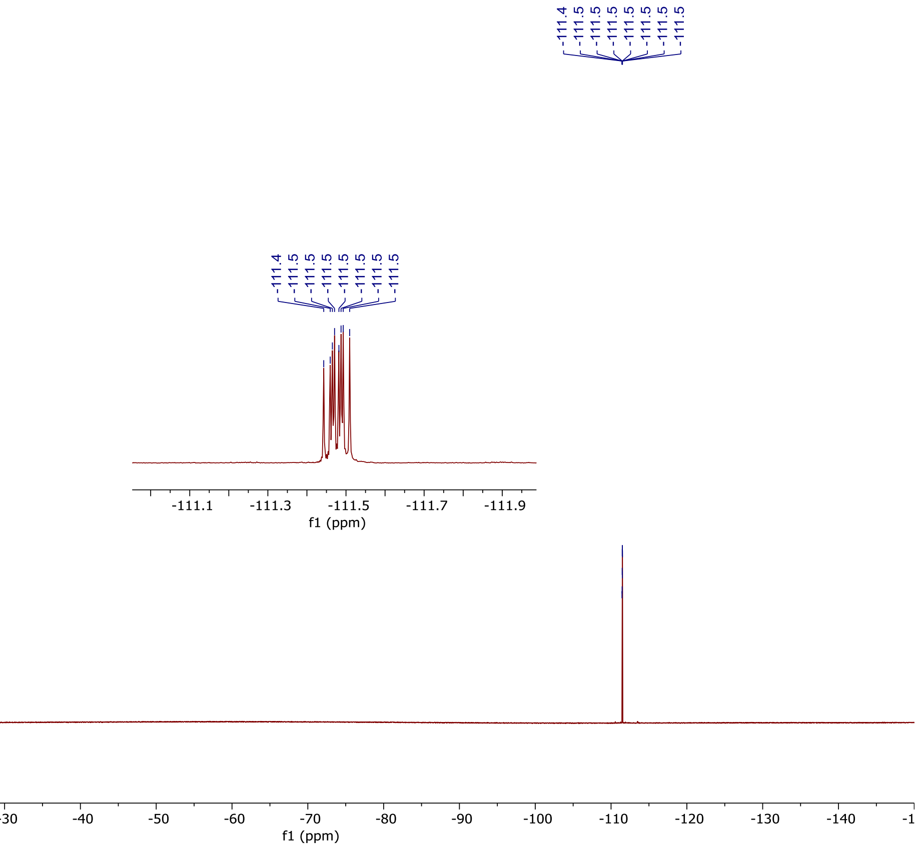

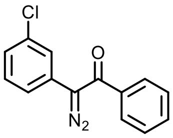

1o
^{1}H NMR (600 MHz, C_6D_6)

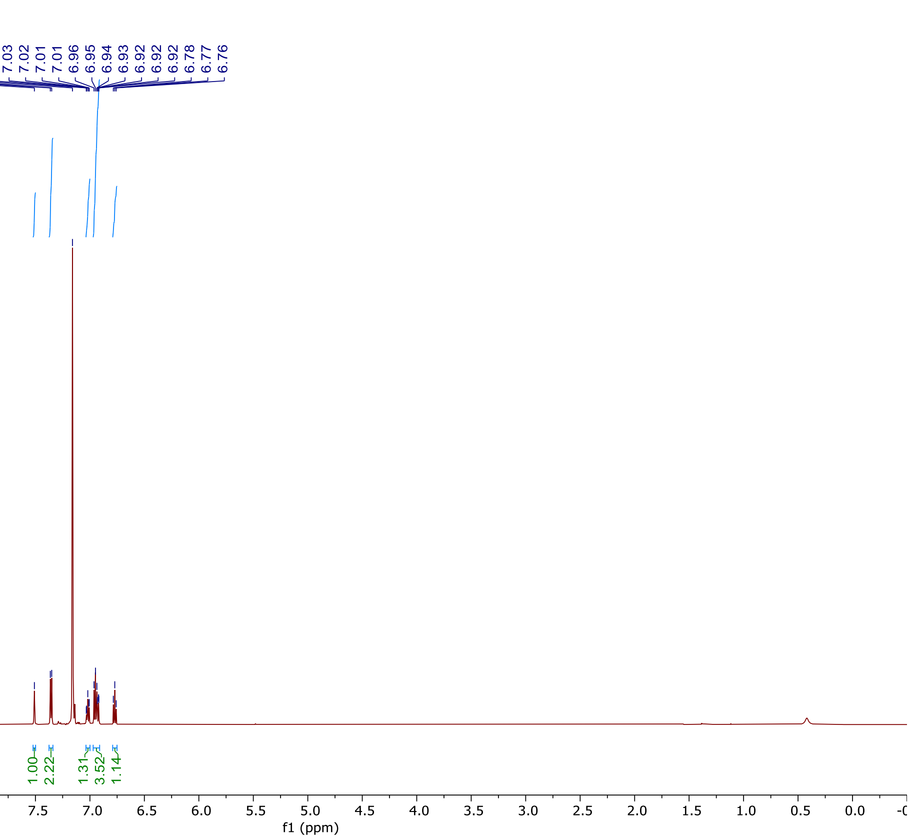

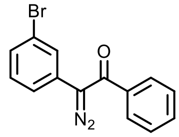

1p
^{1}H NMR (600 MHz, CDCl$_3$)

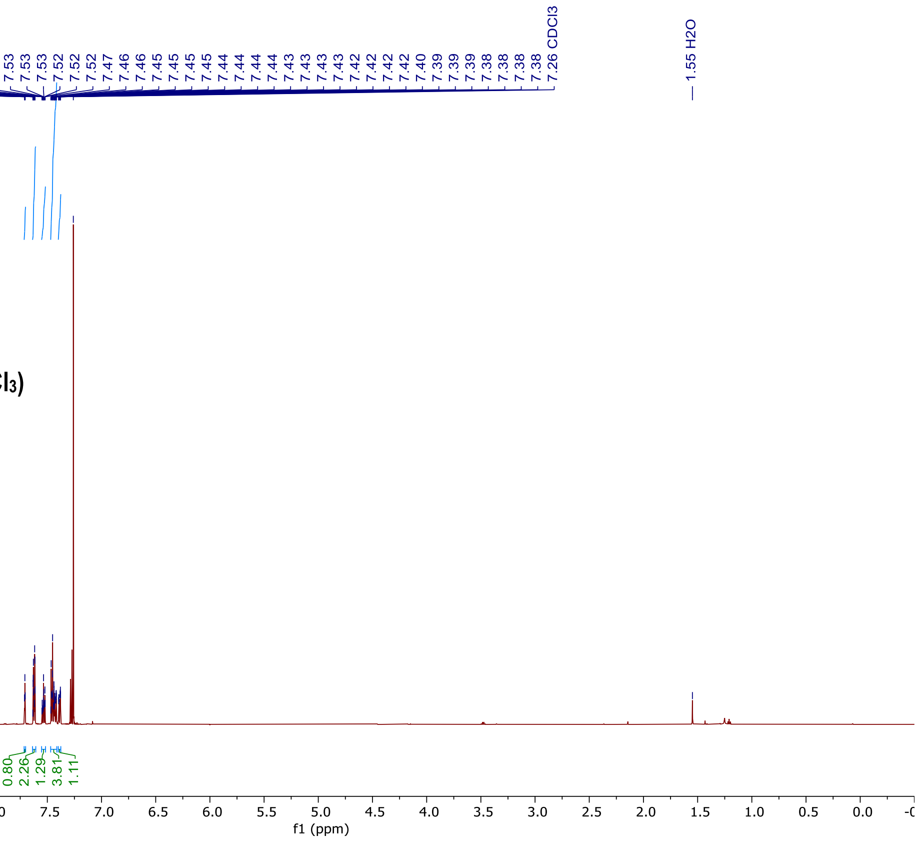

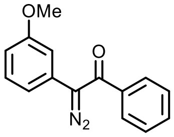

1q
^{1}H NMR (600 MHz, CDCl$_3$)

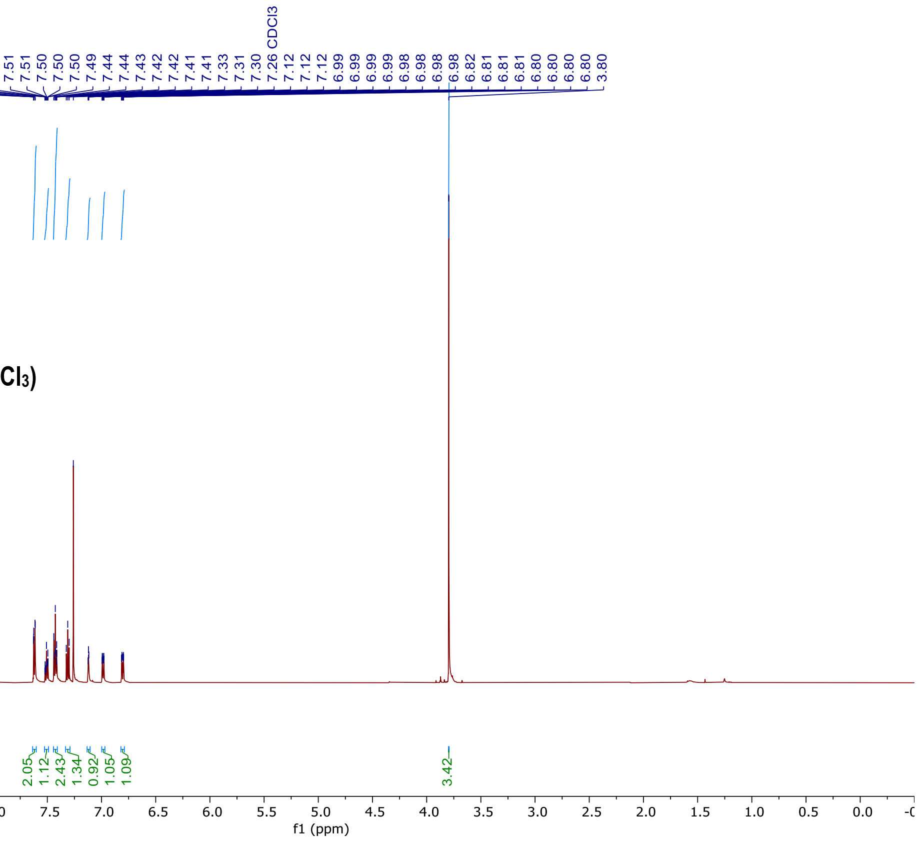

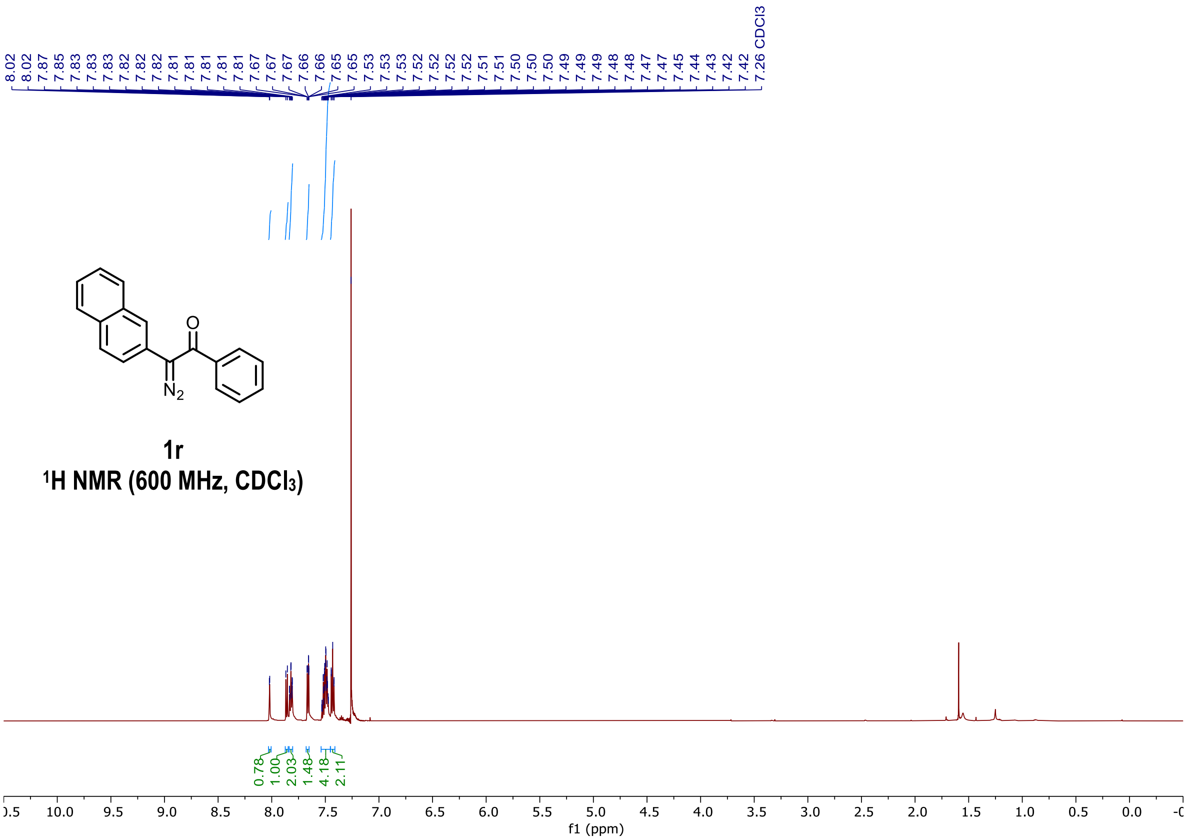
8.02
8.02
7.87
7.85
7.83
7.83
7.83
7.82
7.82
7.81
7.81
7.81
7.81
7.67
7.67
7.67
7.66
7.66
7.65
7.65
7.53
7.53
7.52
7.52
7.52
7.52
7.51
7.50
7.50
7.50
7.49
7.49
7.49
7.48
7.48
7.47
7.47
7.45
7.44
7.43
7.42
7.26 CDCl3
O
N2
1r
1H NMR (600 MHz, CDCl3)
0.78
1.00
2.03
1.48
4.18
2.11
f1 (ppm)

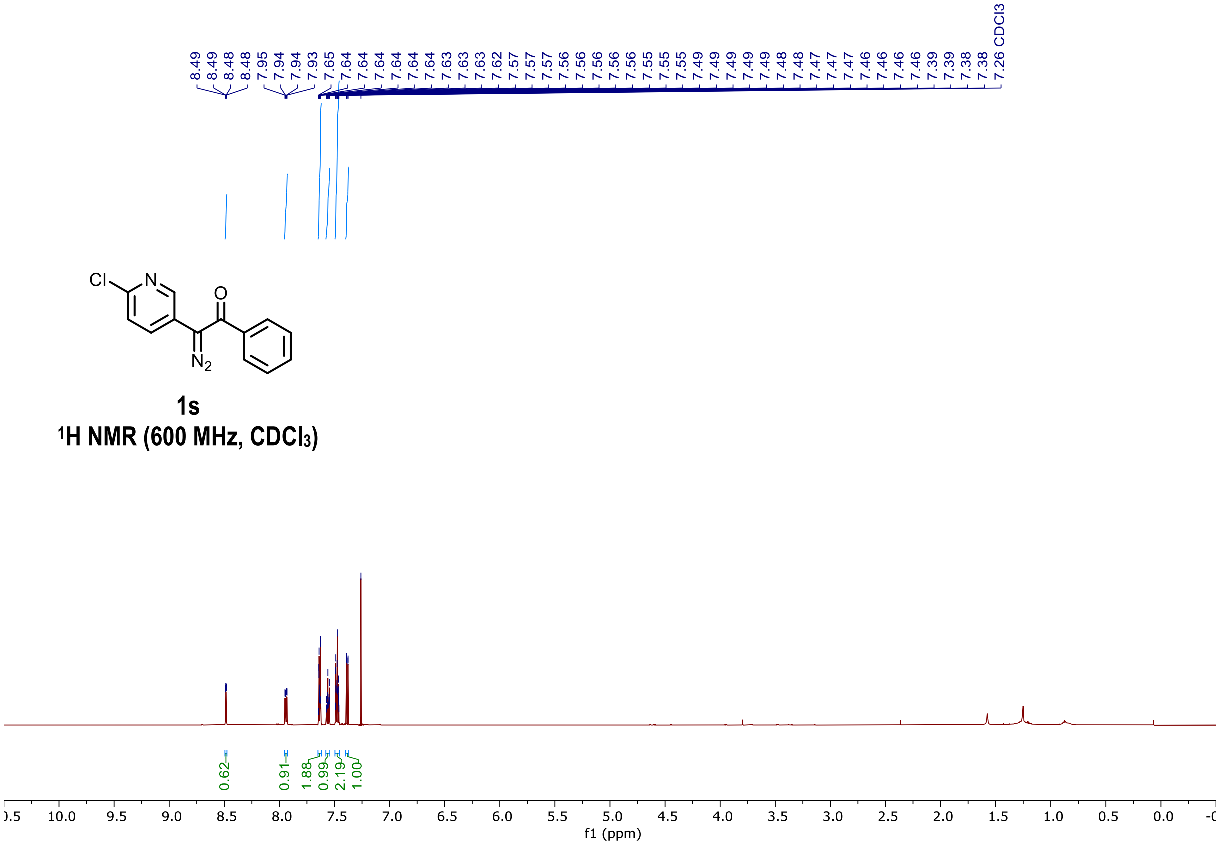
8.49
8.49
8.48
8.48
7.95
7.94
7.94
7.93
7.65
7.64
7.64
7.64
7.64
7.63
7.63
7.63
7.62
7.57
7.57
7.56
7.56
7.56
7.56
7.55
7.55
7.49
7.49
7.49
7.49
7.48
7.48
7.47
7.47
7.46
7.46
7.46
7.39
7.39
7.38
7.38
7.26 CDCl3
Cl
N
O
N2
1s
1H NMR (600 MHz, CDCl3)
0.62
0.91
1.88
0.99
2.19
1.00
f1 (ppm)

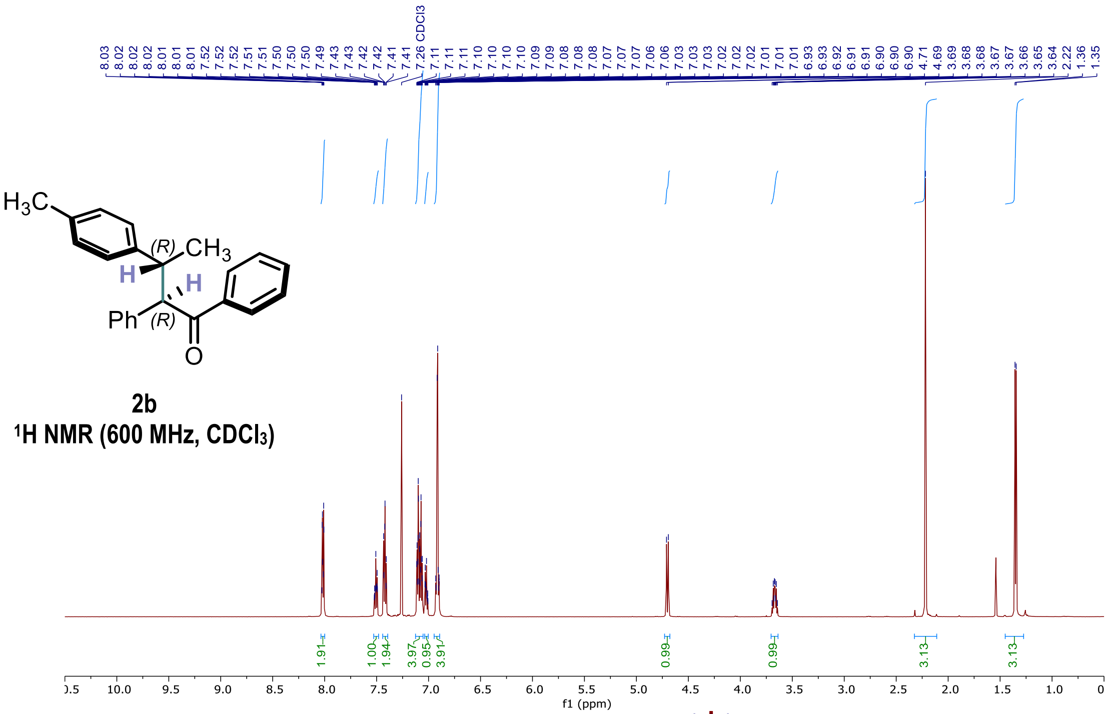

2b

¹H NMR (600 MHz, CDCl₃)

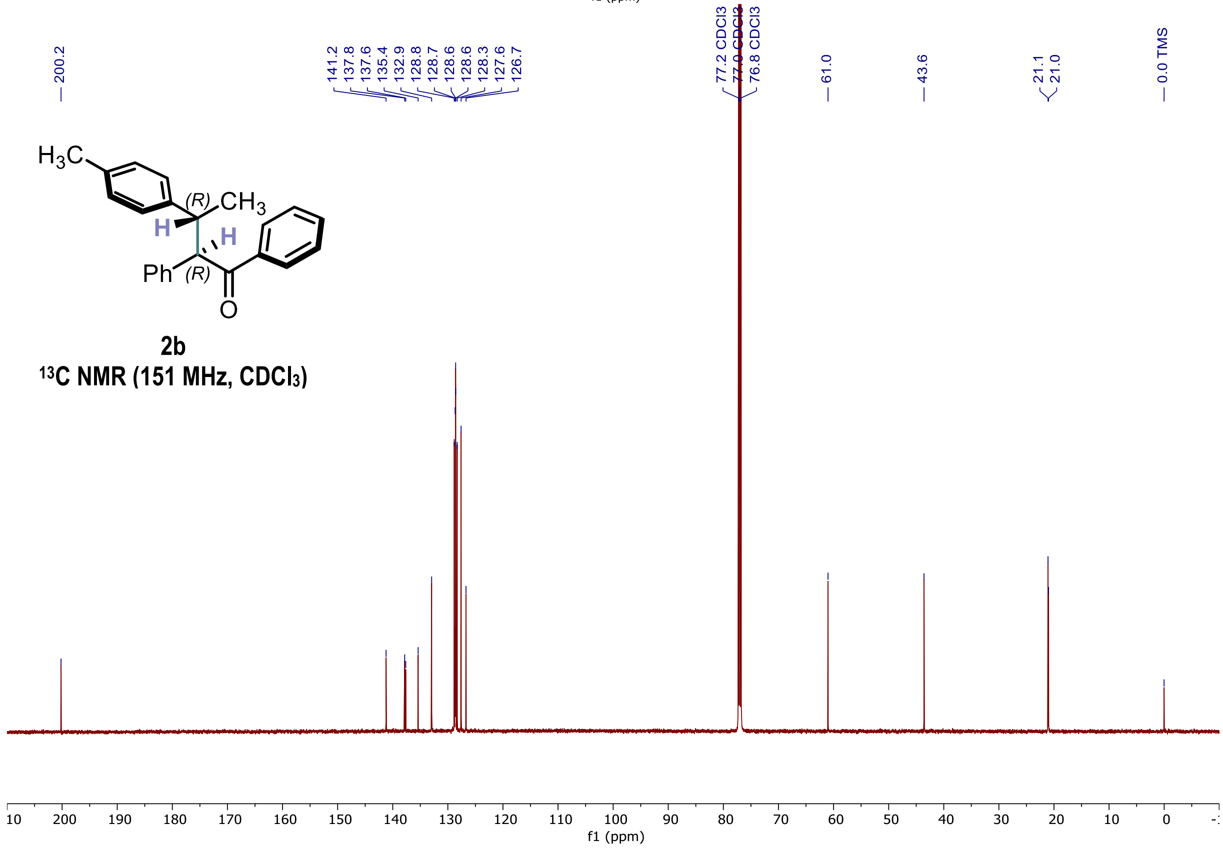

2b

¹³C NMR (151 MHz, CDCl₃)

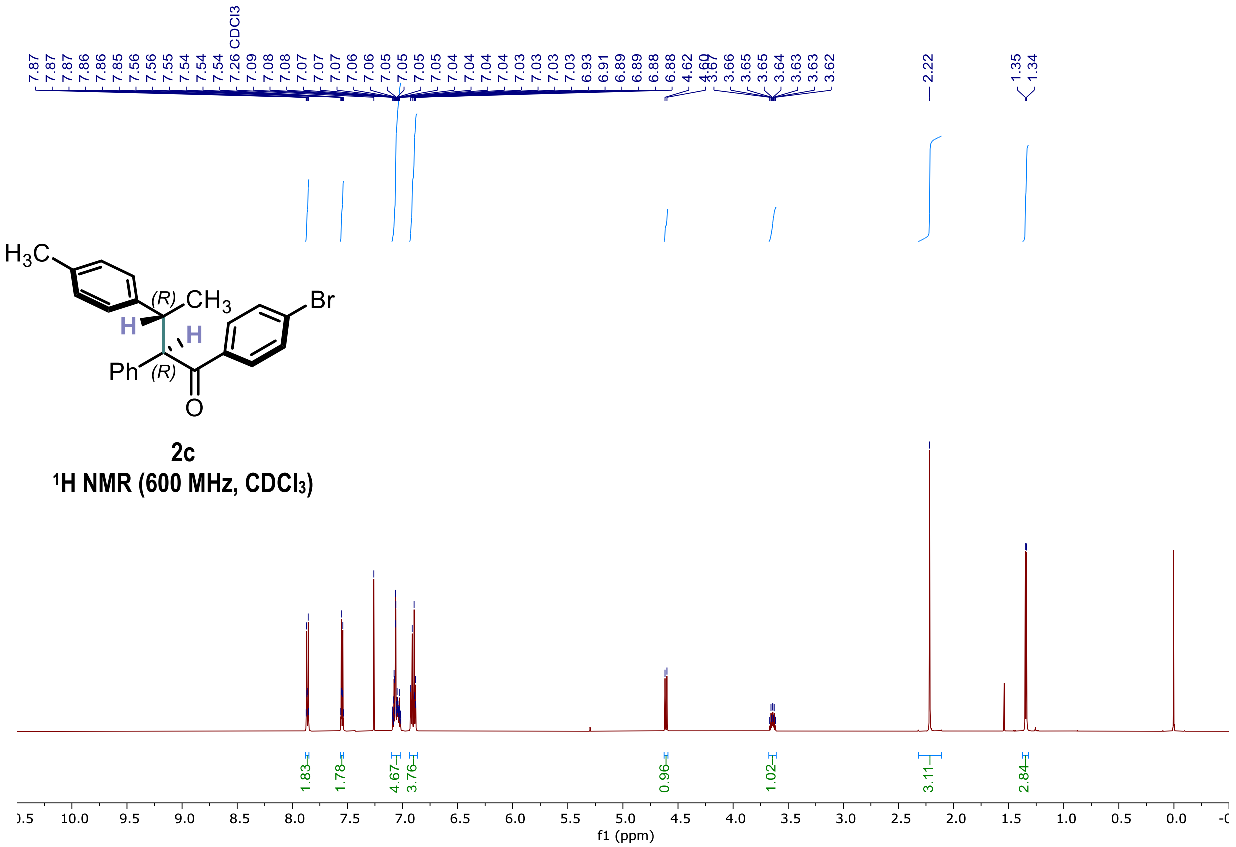

2c

^{1}H NMR (600 MHz, $CDCl_3$)

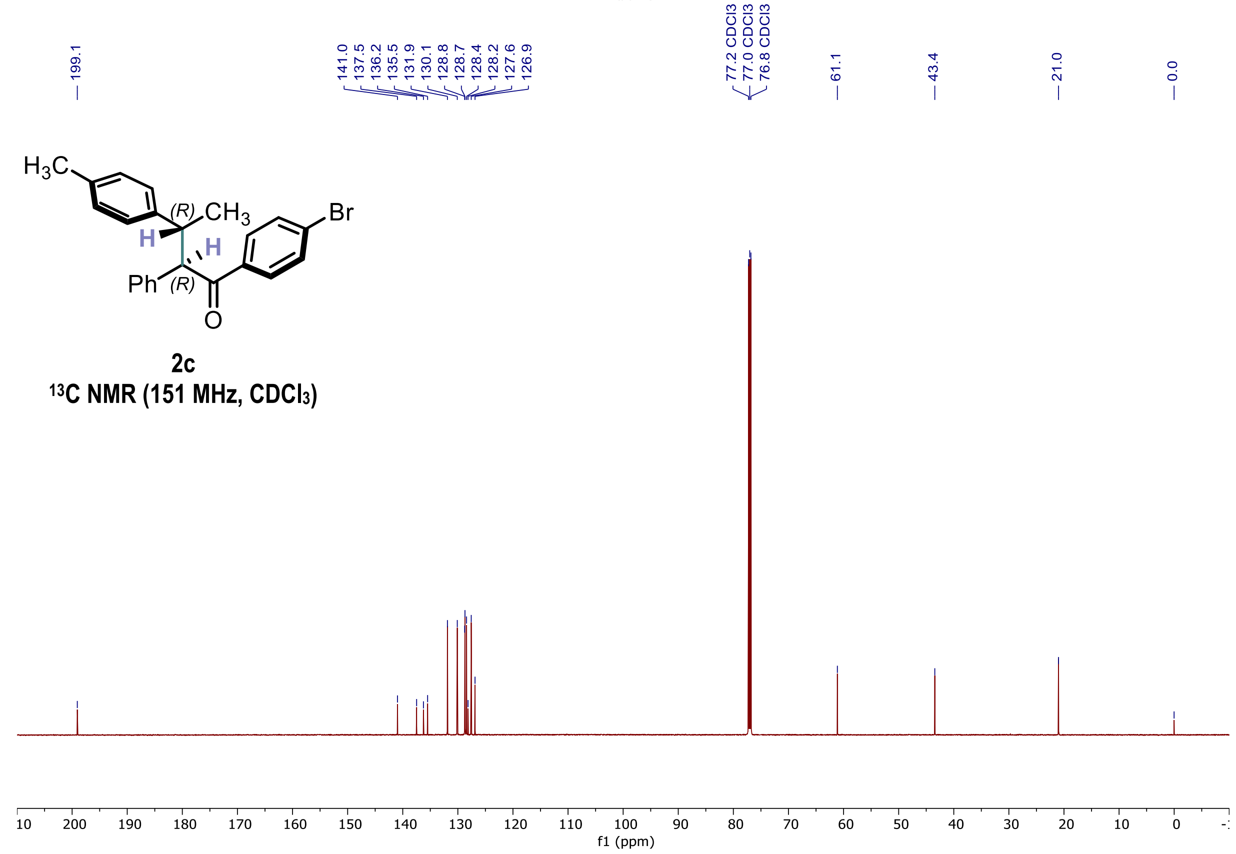

2c

^{13}C NMR (151 MHz, $CDCl_3$)

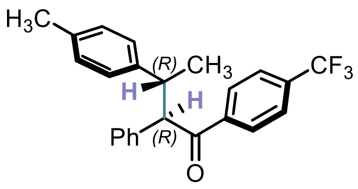

2d

^{1}H NMR (800 MHz, $CDCl_3$)

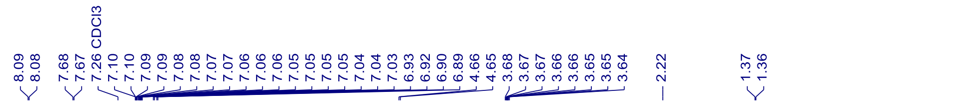

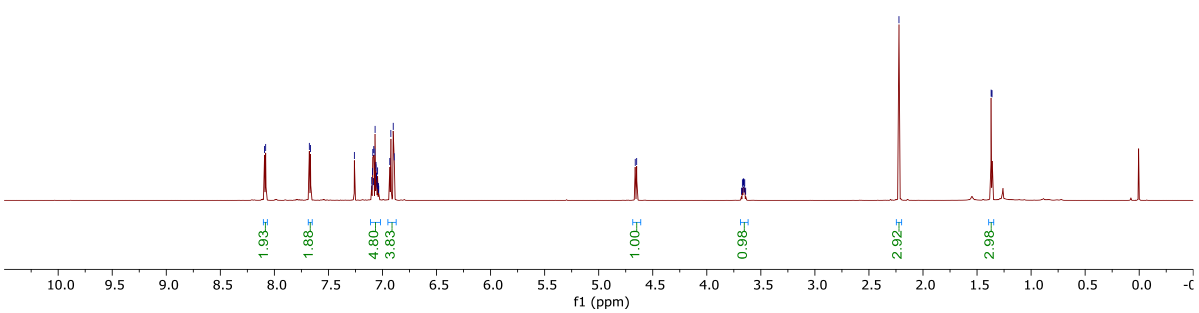

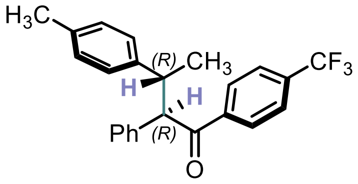

2d
^{13}C NMR (201 MHz, $CDCl_3$)

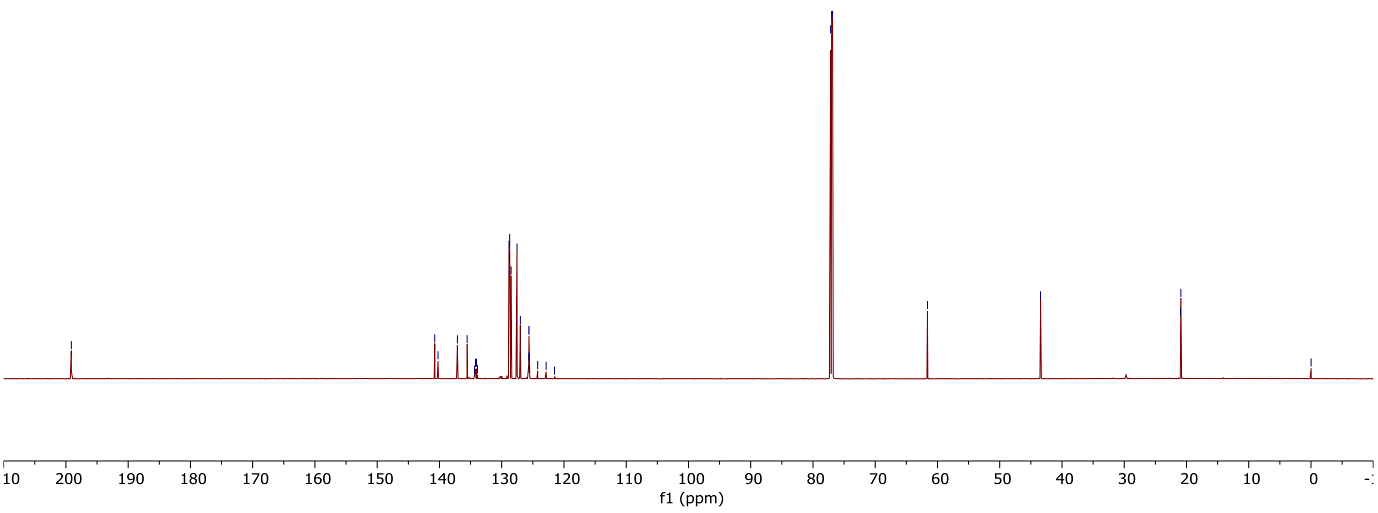

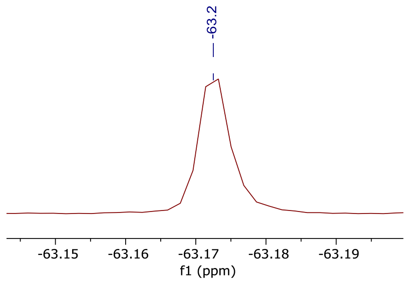

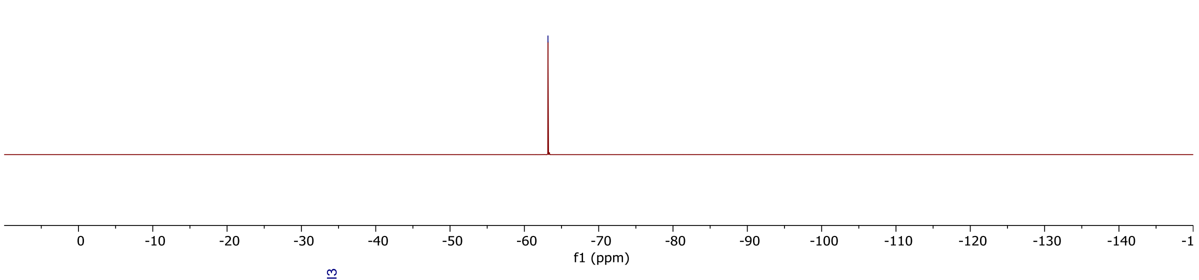

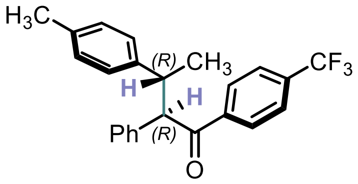

2d

^{19}F NMR (753 MHz, CDCl$_3$)

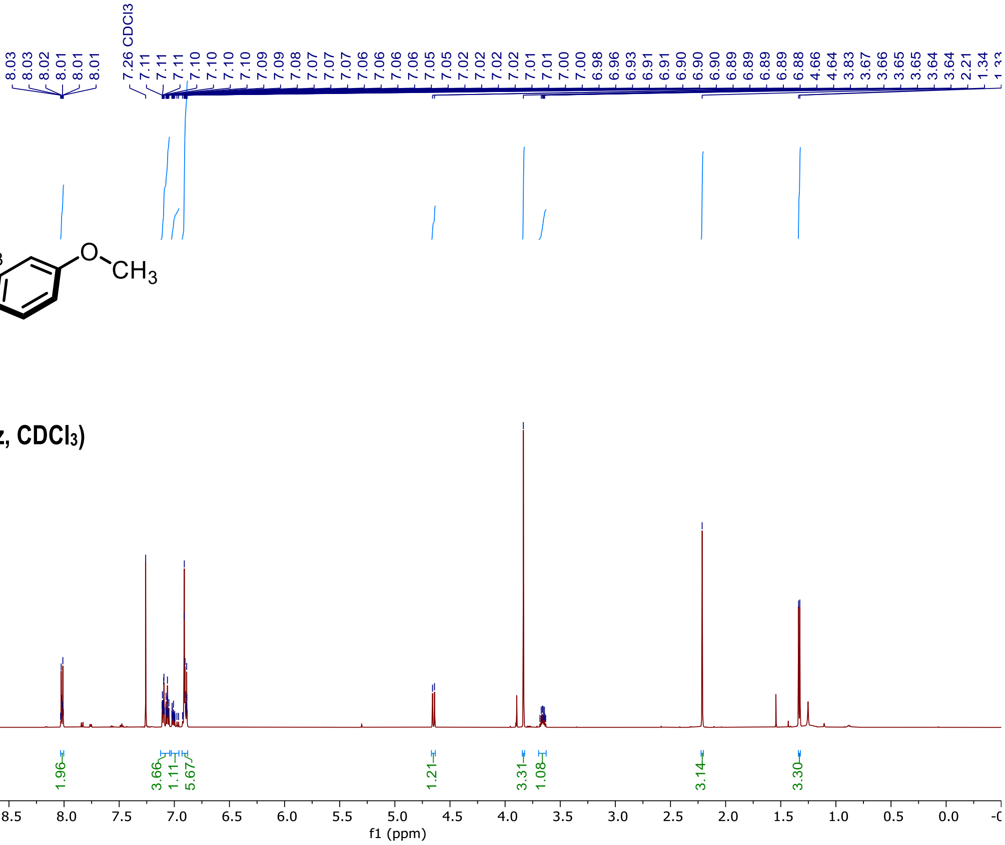

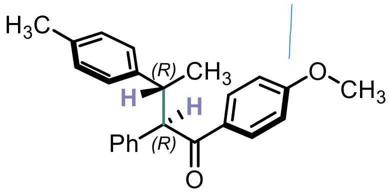

2e

^{1}H NMR (600 MHz, CDCl$_3$)

2e
^{13}C NMR (151 MHz, CDCl$_3$)

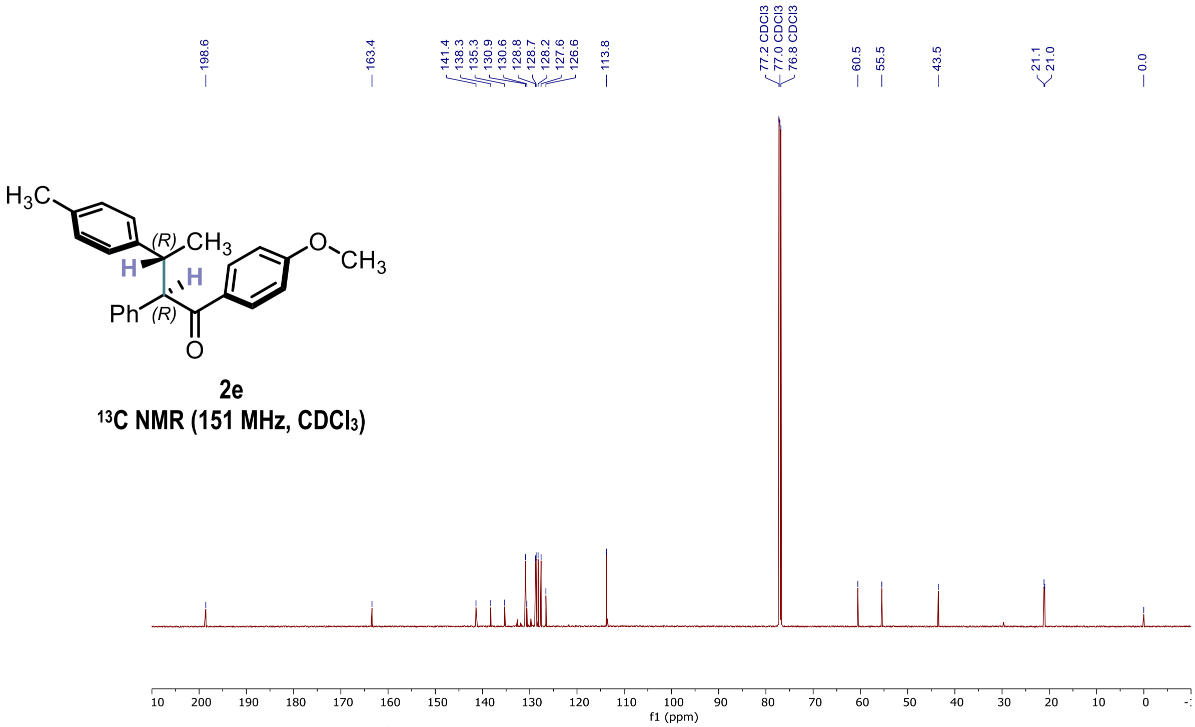

2f
^{1}H NMR (600 MHz, CDCl$_3$)

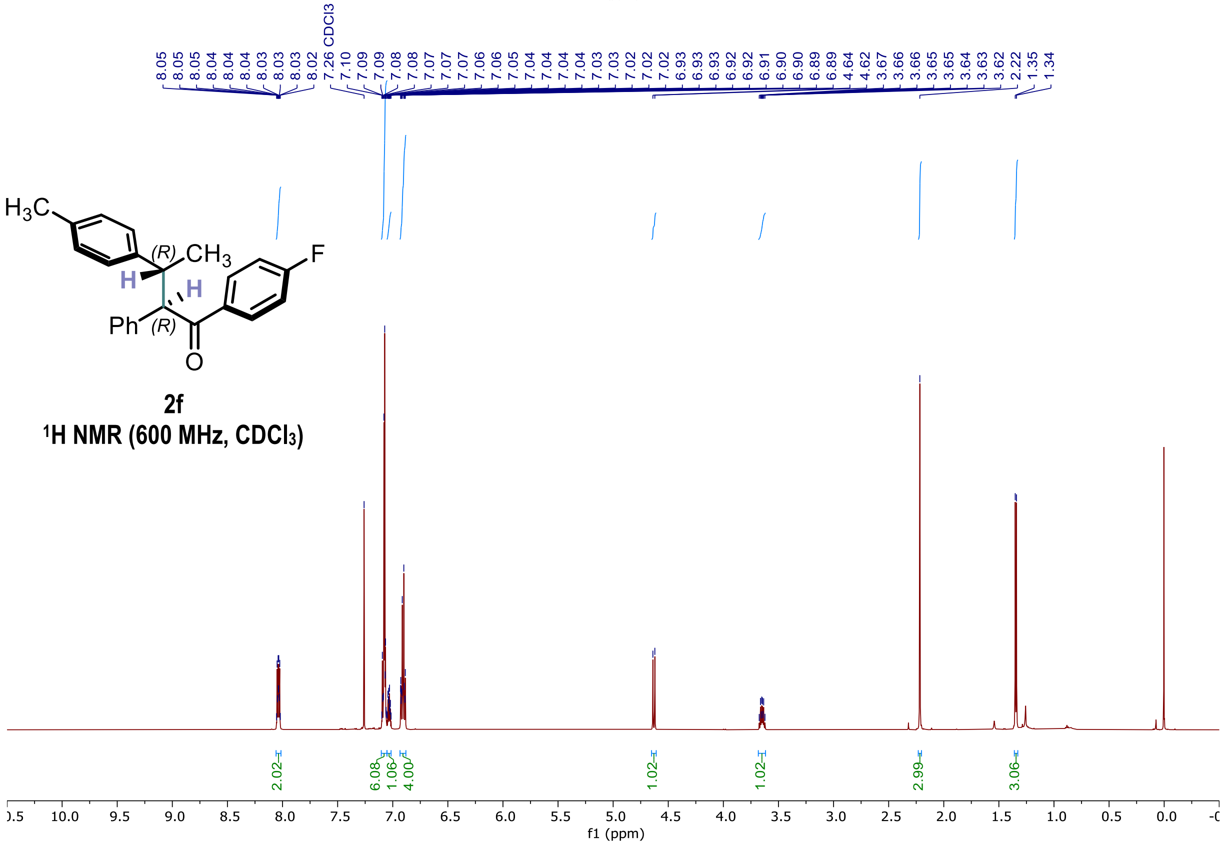

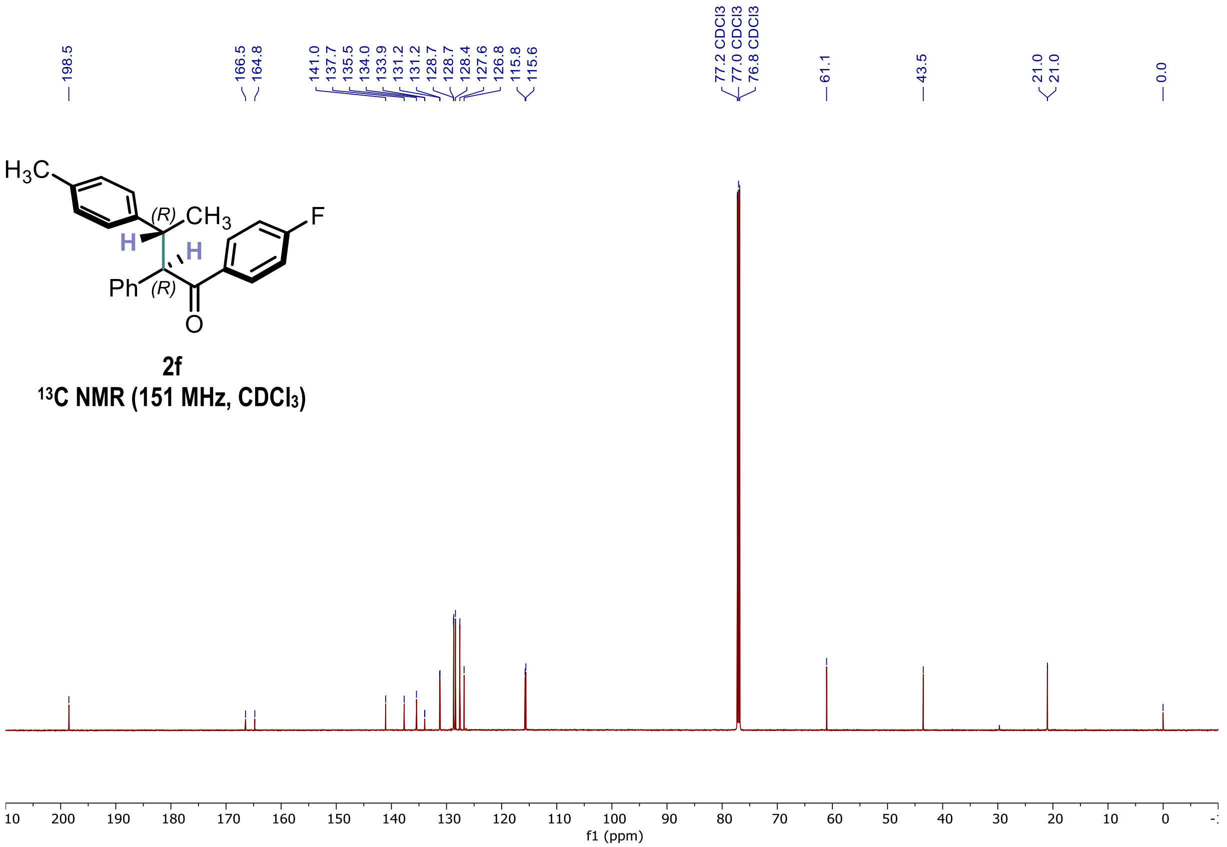

2f

^{13}C NMR (151 MHz, CDCl$_3$)

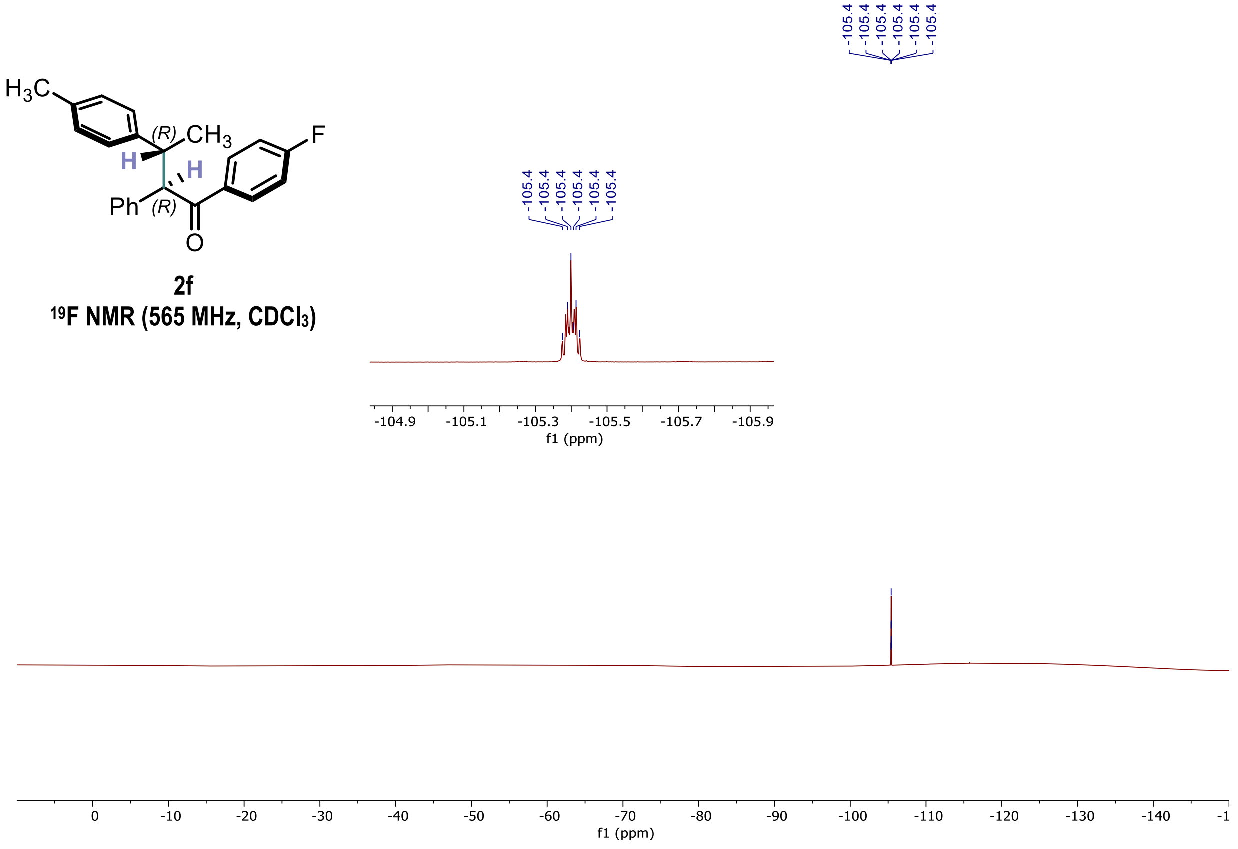

2f

^{19}F NMR (565 MHz, CDCl$_3$)

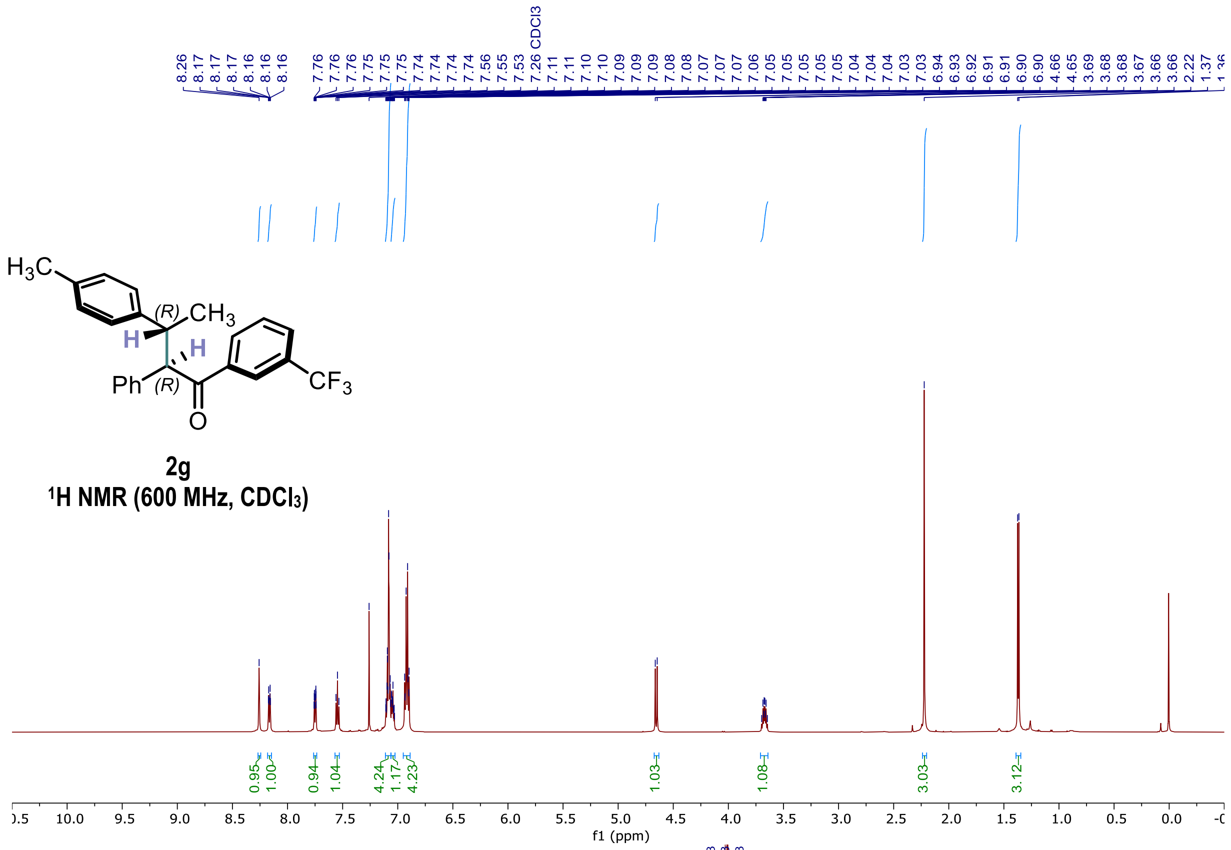

2g

^{1}H NMR (600 MHz, $CDCl_3$)

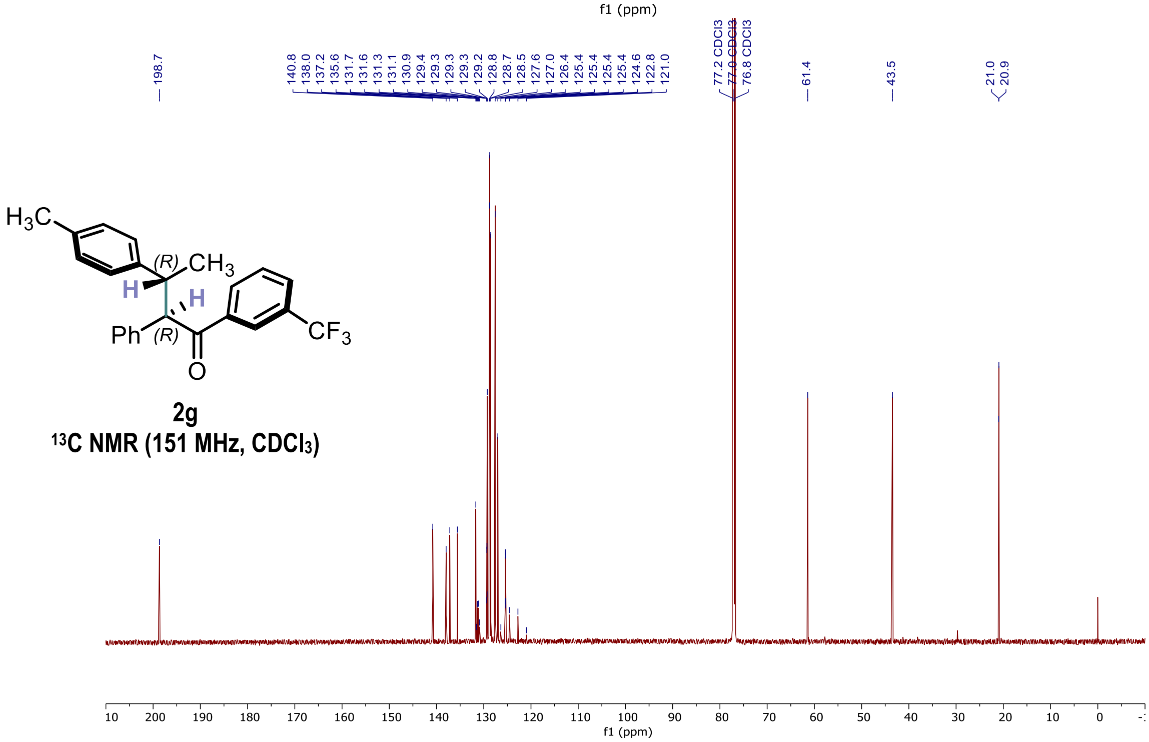

2g

^{13}C NMR (151 MHz, $CDCl_3$)

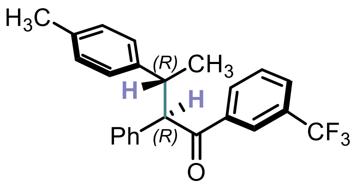

2g

^{19}F NMR (565 MHz, CDCl$_3$)

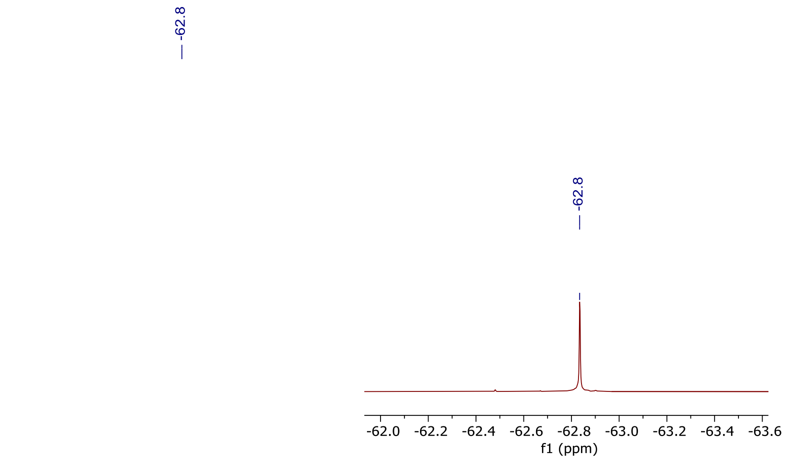

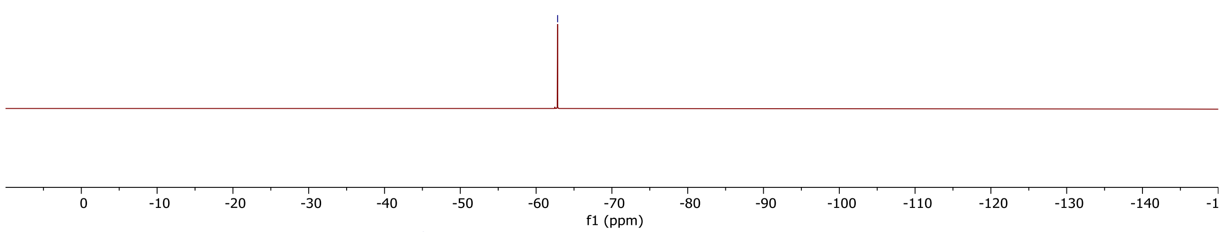

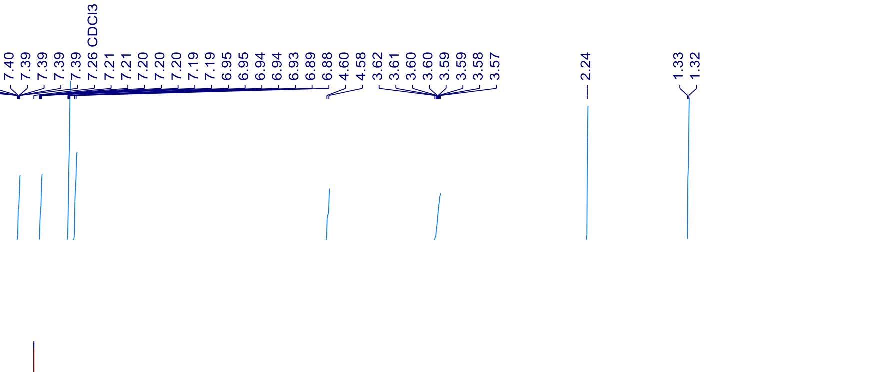

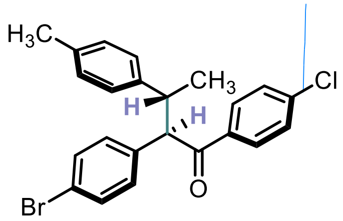

2h

^{1}H NMR (600 MHz, CDCl$_3$)

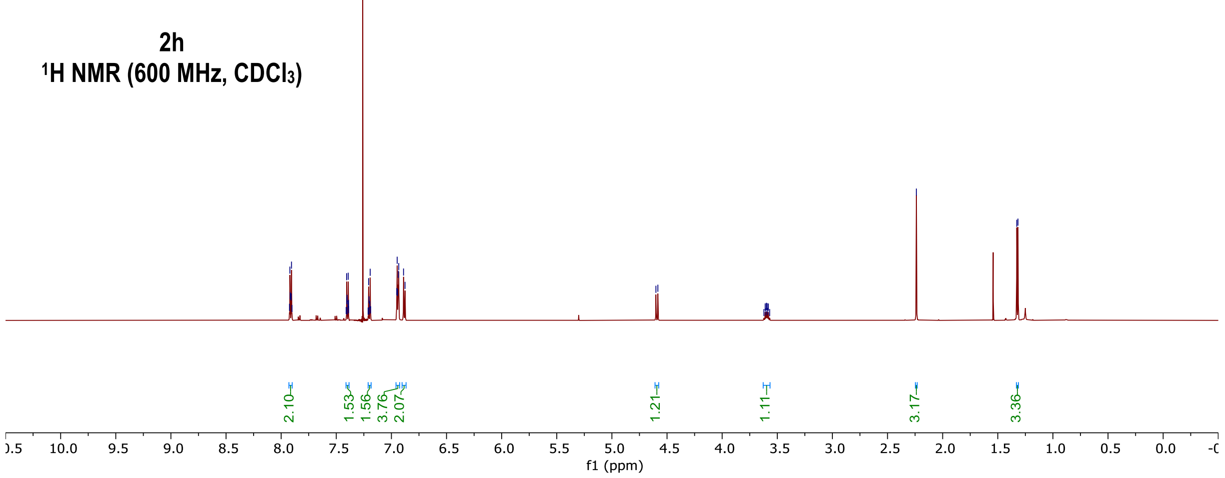

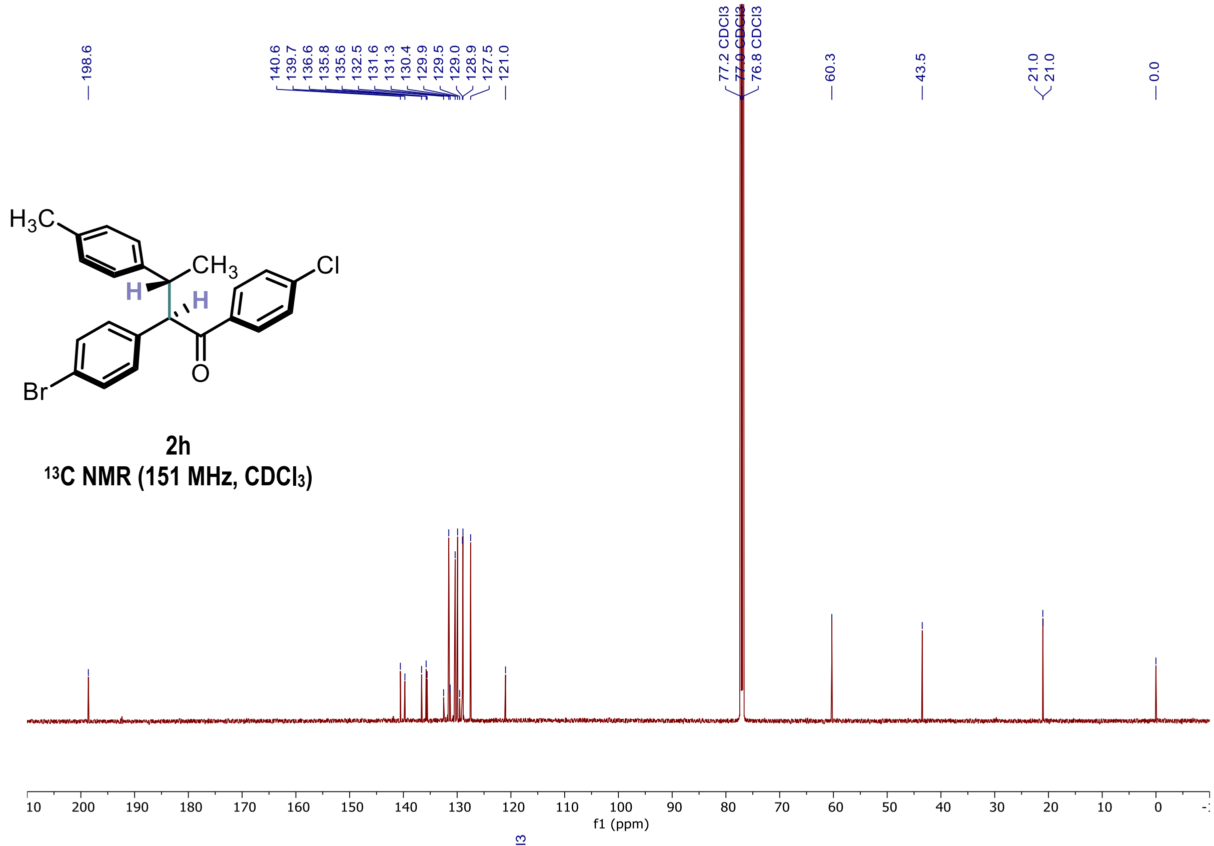

2h

^{13}C NMR (151 MHz, CDCl$_3$)

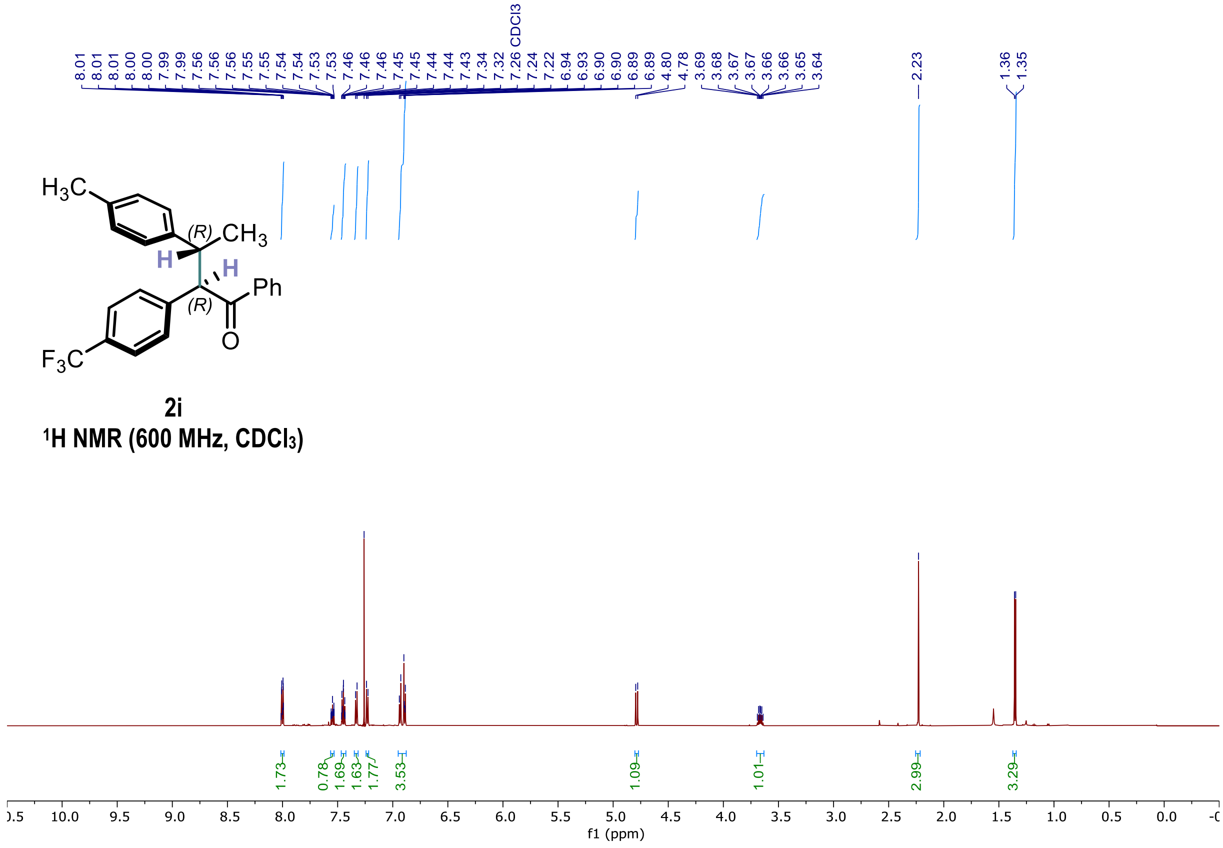

2i

^{1}H NMR (600 MHz, CDCl$_3$)

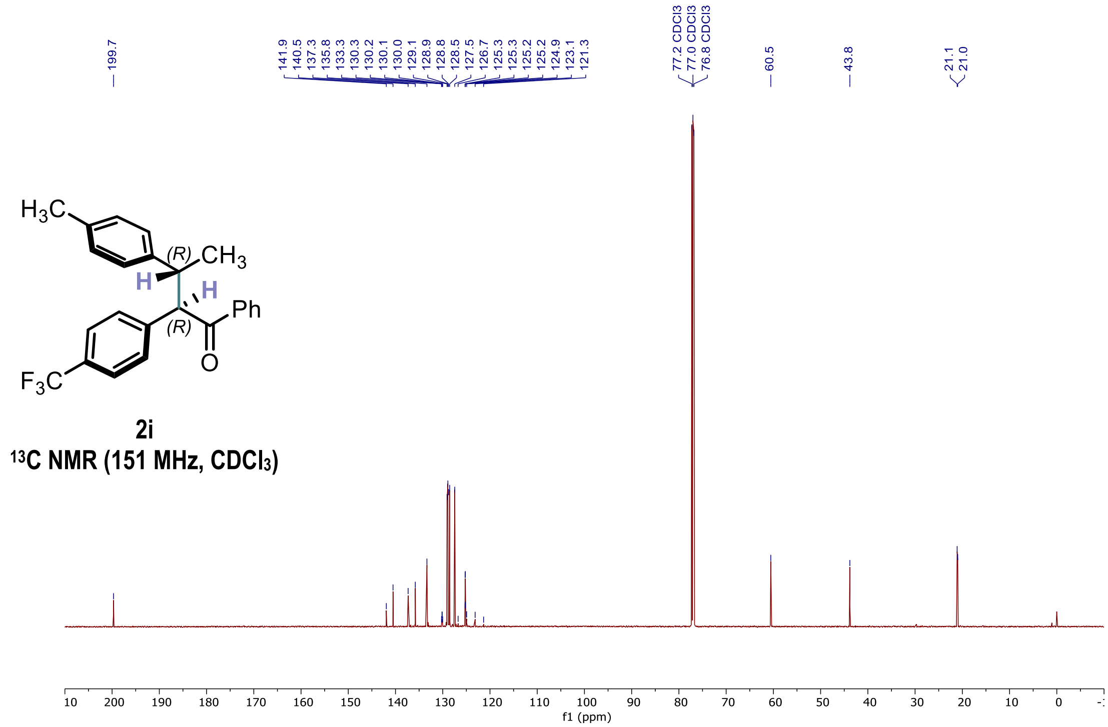

2i
^{13}C NMR (151 MHz, $CDCl_3$)

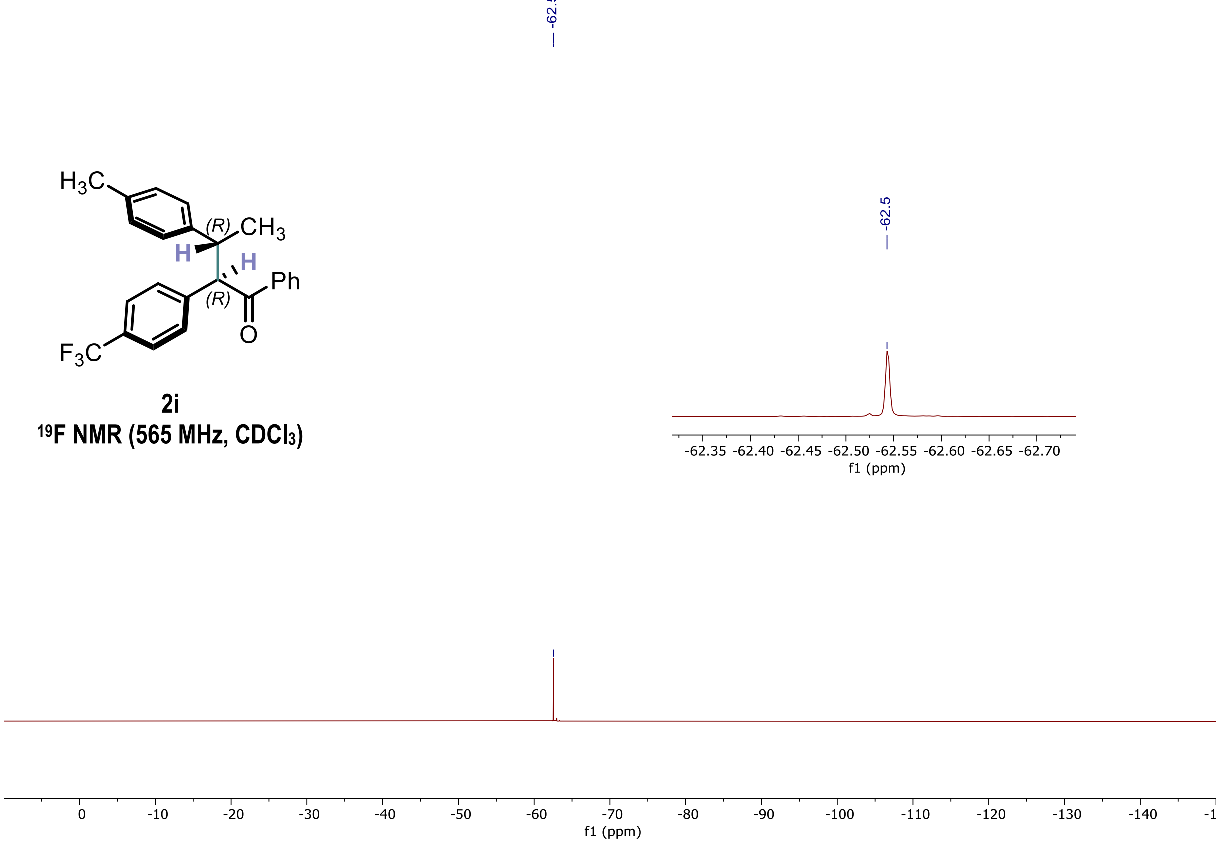

2i
^{19}F NMR (565 MHz, $CDCl_3$)

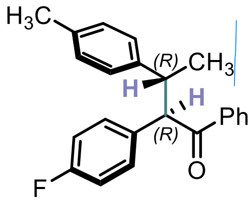

2j
^{1}HNMR (600 MHz, CDCl3)

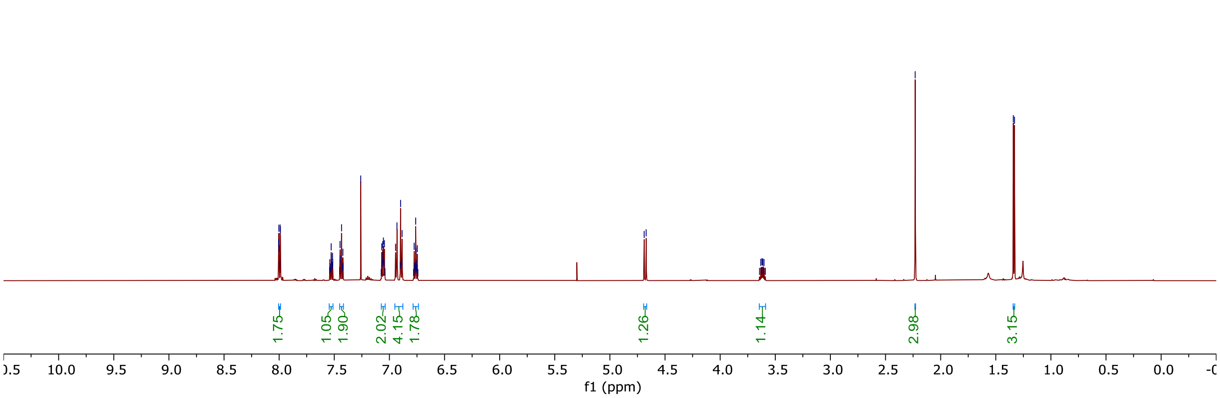

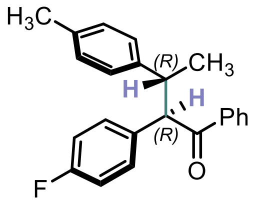

2j
^{13}C NMR (151 MHz, CDCl3)

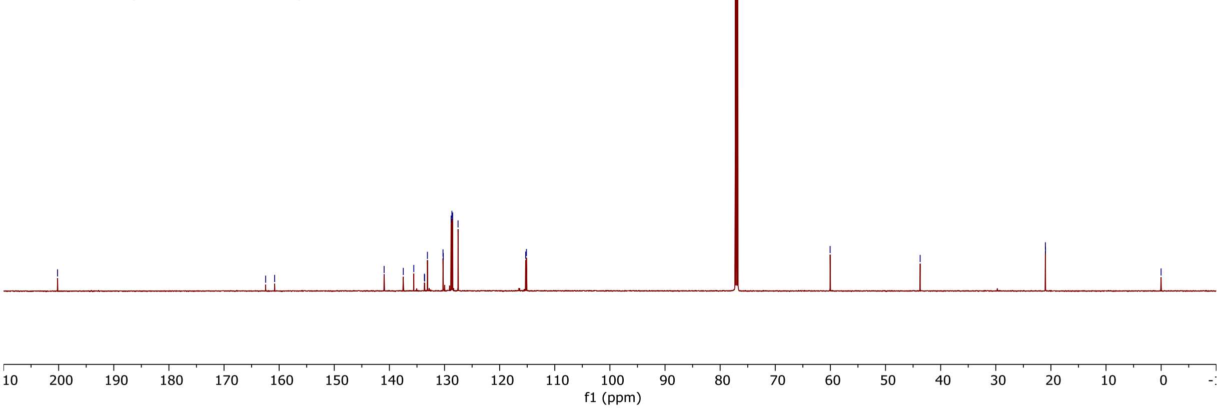

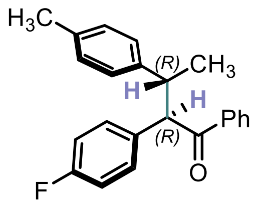

2j
^{19}F NMR (565 MHz, $CDCl_3$)

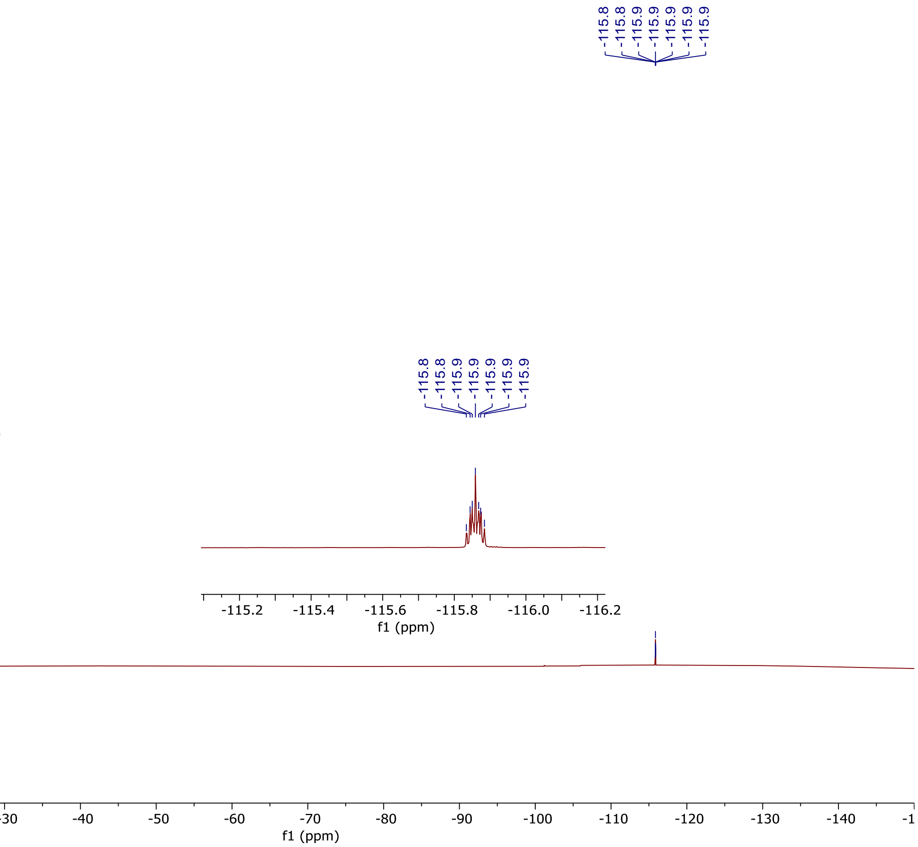

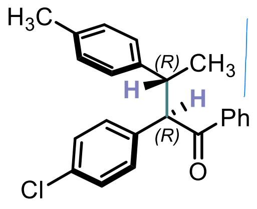

2k
^{1}H NMR (600 MHz, $CDCl_3$)

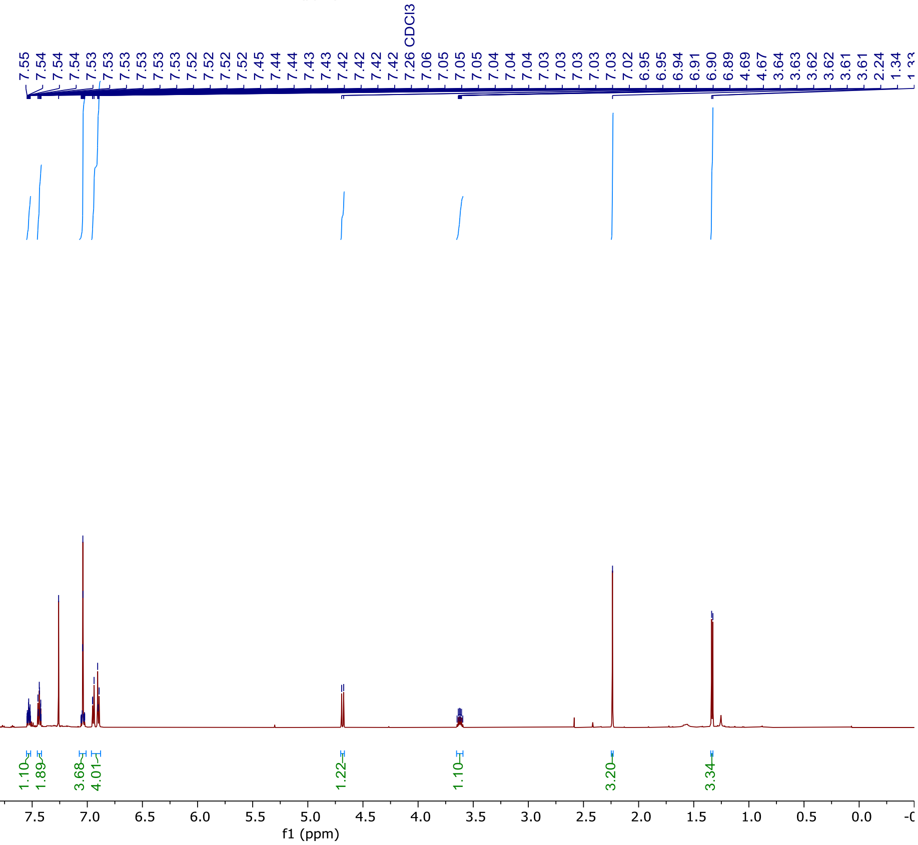

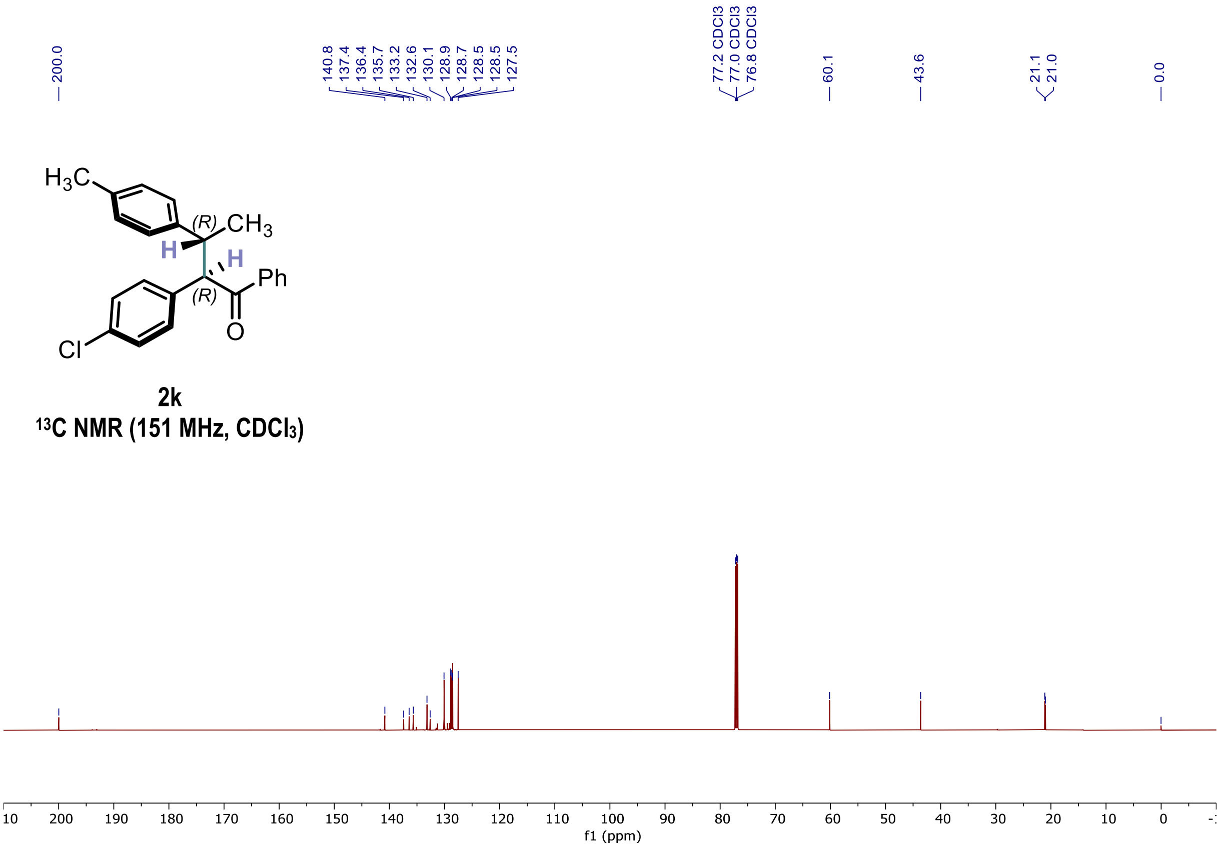

2k
^{13}C NMR (151 MHz, $CDCl_3$)

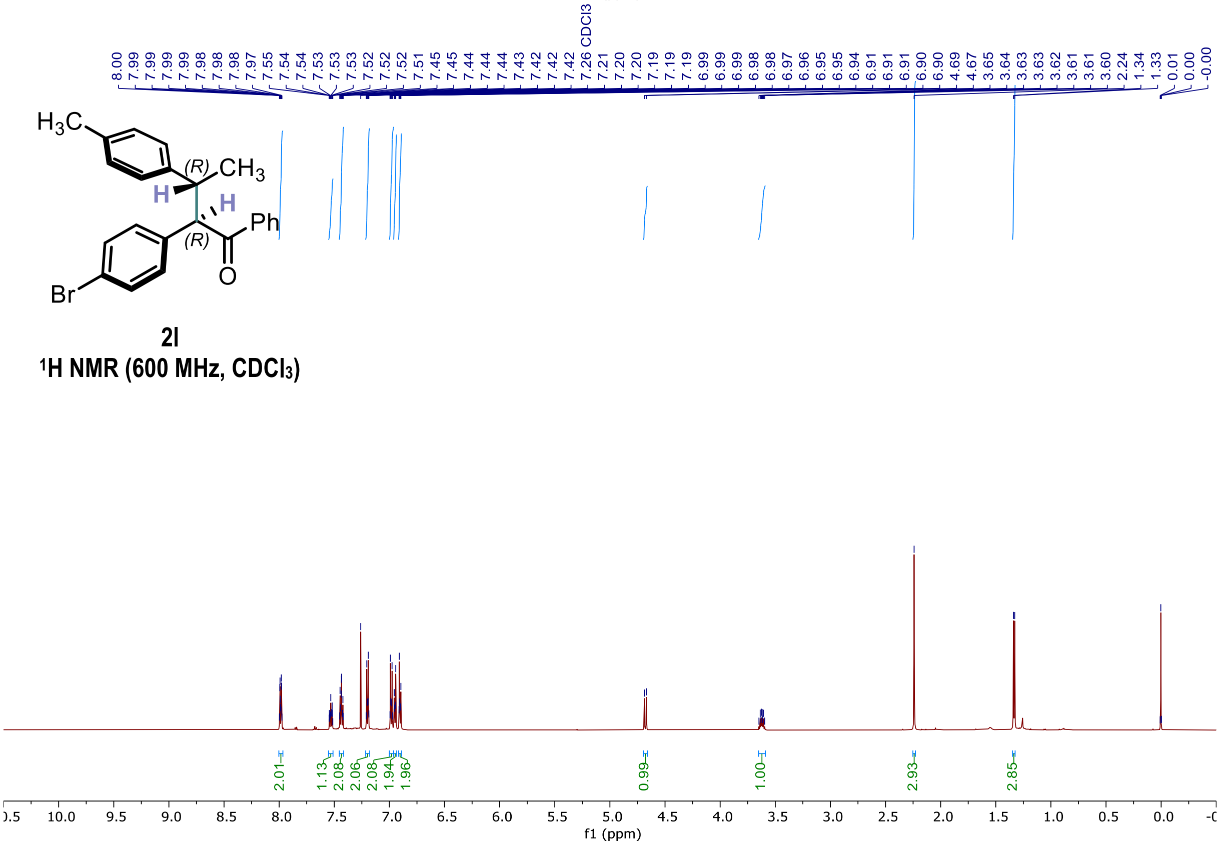

2l
^{1}H NMR (600 MHz, $CDCl_3$)

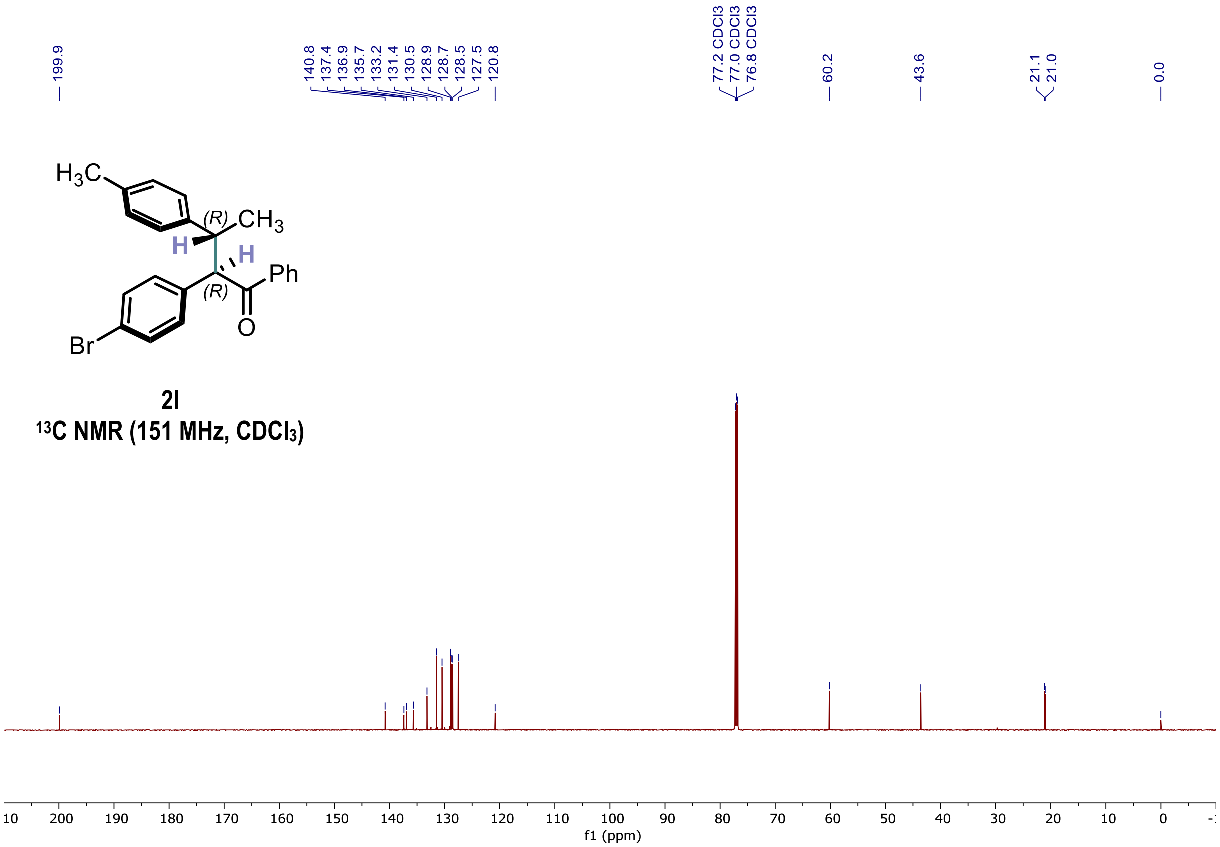

2l

^{13}C NMR (151 MHz, $CDCl_3$)

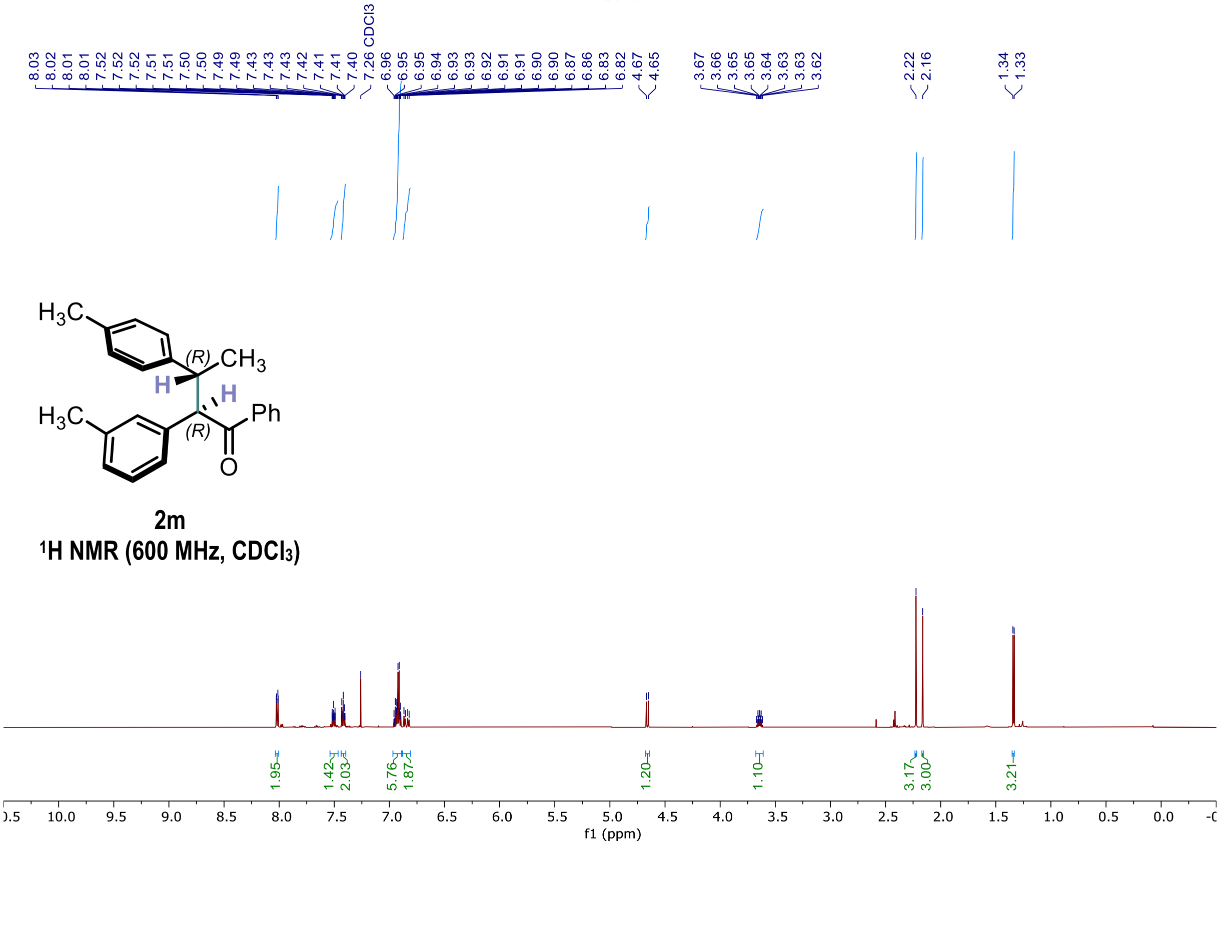

2m

^1H NMR (600 MHz, $CDCl_3$)

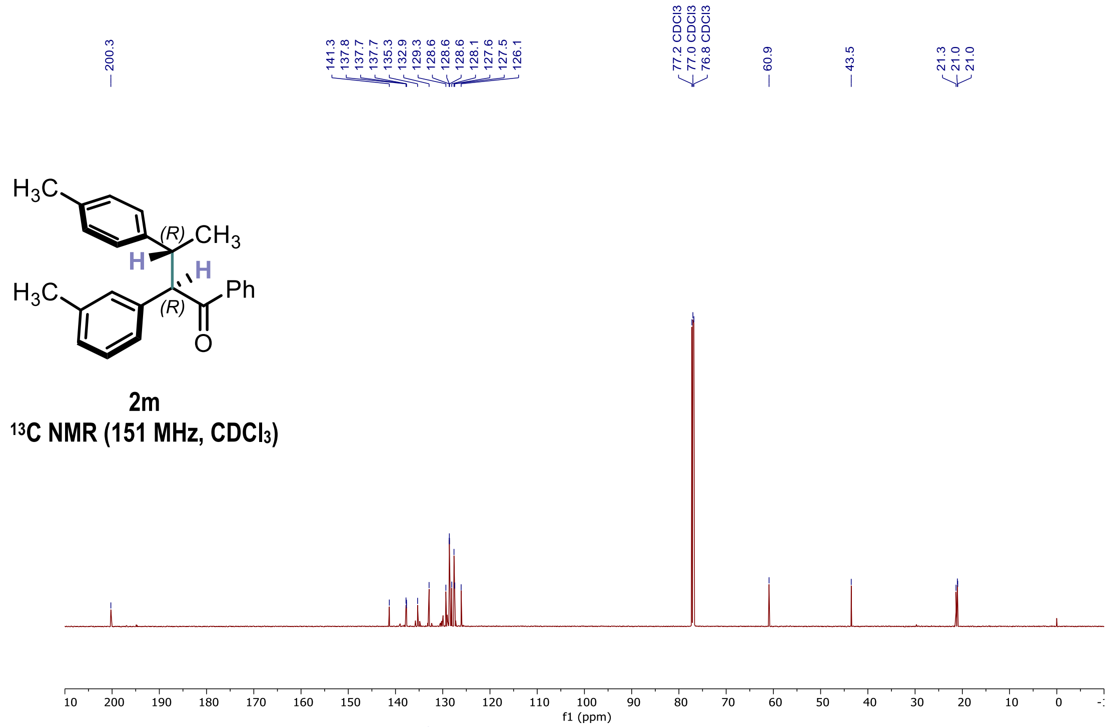

2m
^{13}C NMR (151 MHz, $CDCl_3$)

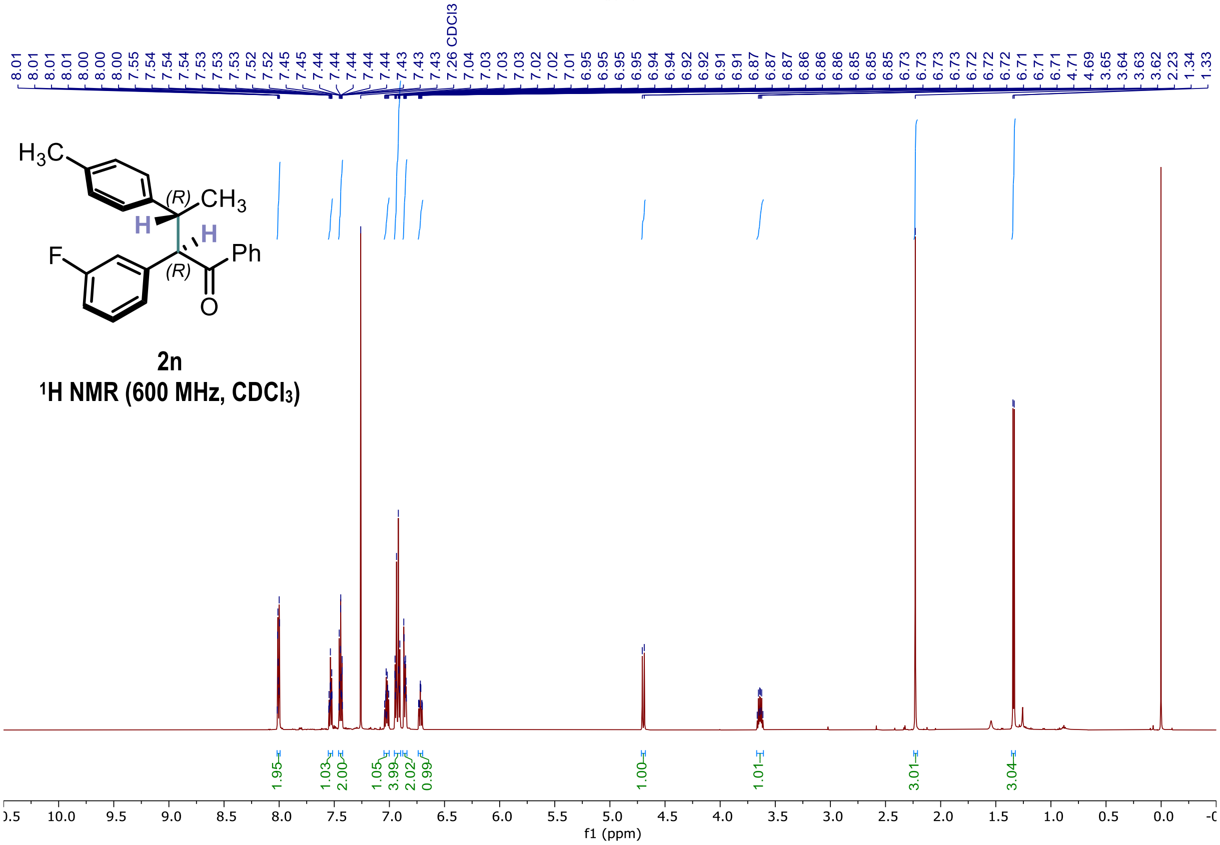

2n
^{1}H NMR (600 MHz, $CDCl_3$)

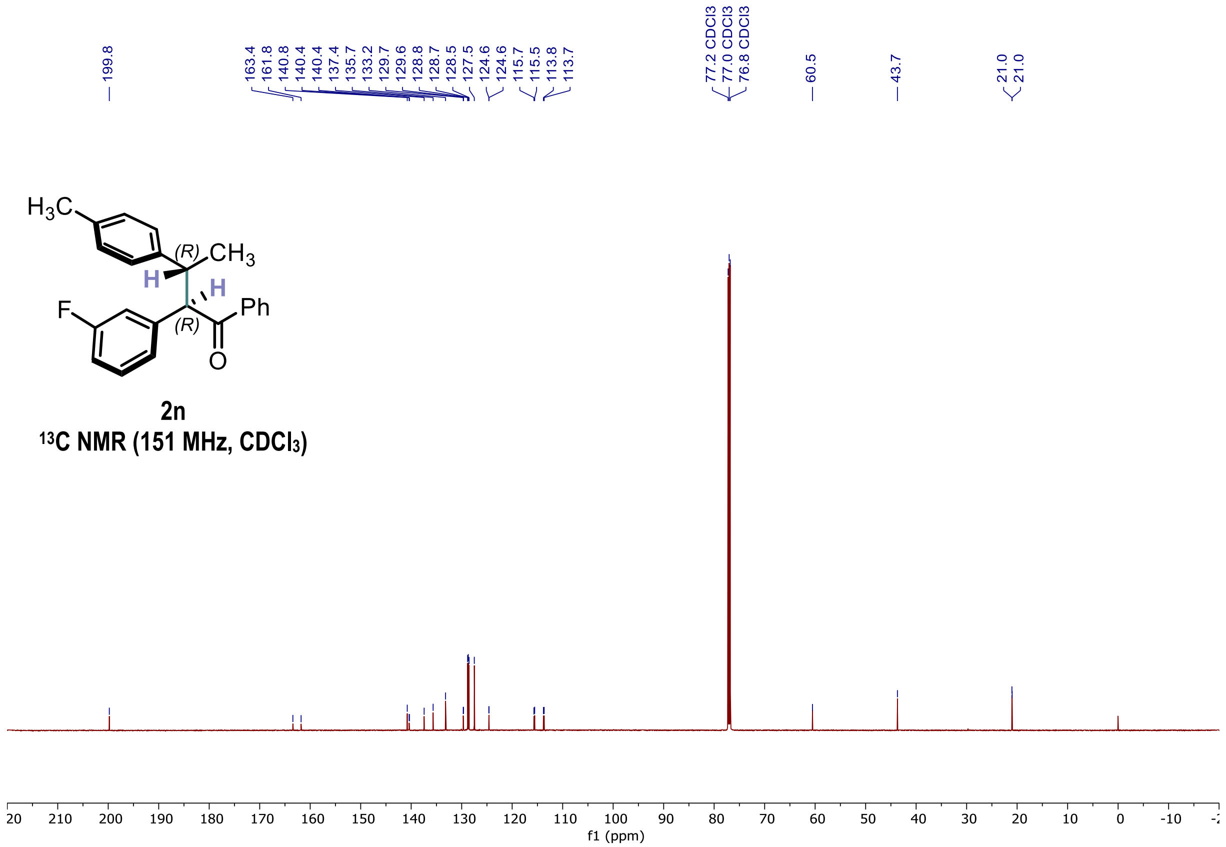
199.8
163.4
161.8
140.8
140.4
140.4
137.4
135.7
133.2
129.7
129.6
128.8
128.7
128.5
127.5
124.6
124.6
115.7
115.5
113.8
113.7
77.2 CDCl3
77.0 CDCl3
76.8 CDCl3
60.5
43.7
21.0
21.0
2n
^{13}C NMR (151 MHz, $CDCl_3$)
f1 (ppm)

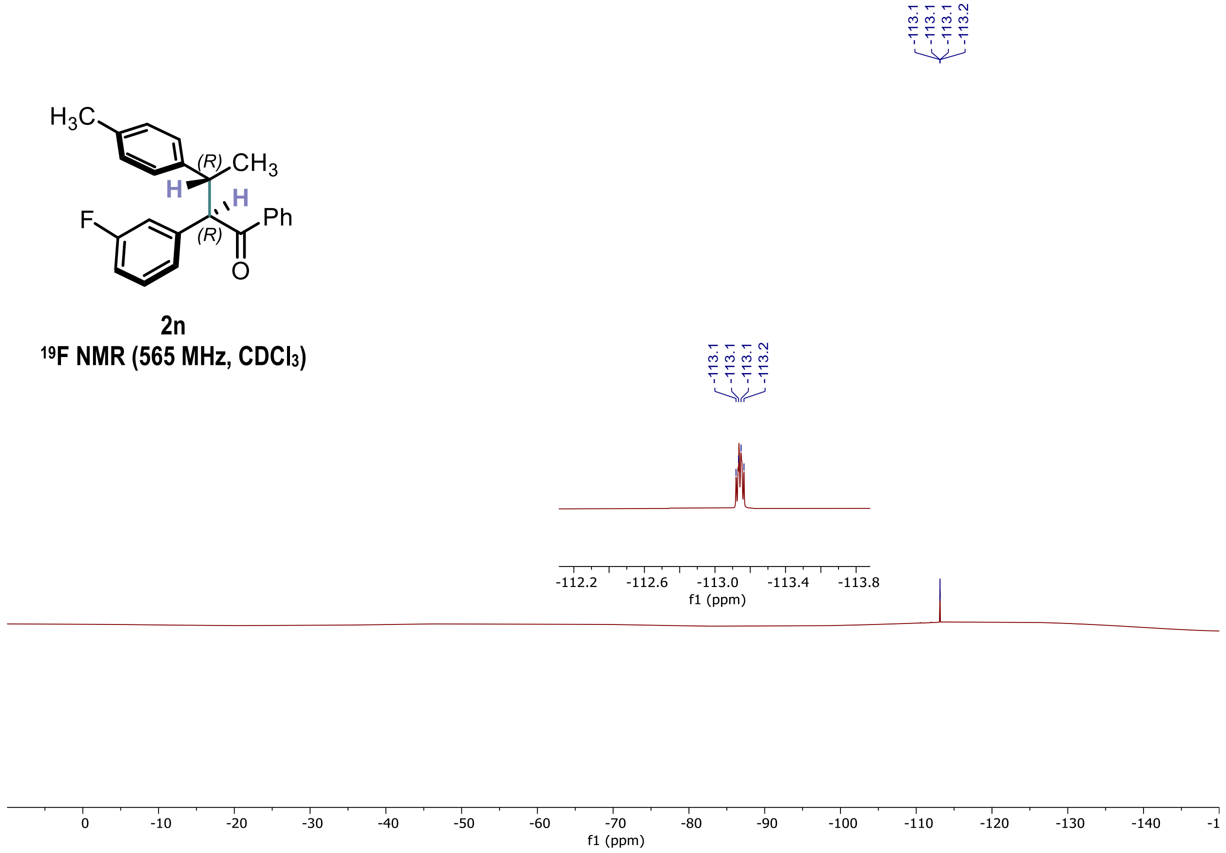
-113.1
-113.1
-113.1
-113.2
2n
^{19}F NMR (565 MHz, $CDCl_3$)
-112.2
-112.6
-113.0
-113.4
-113.8
f1 (ppm)
f1 (ppm)

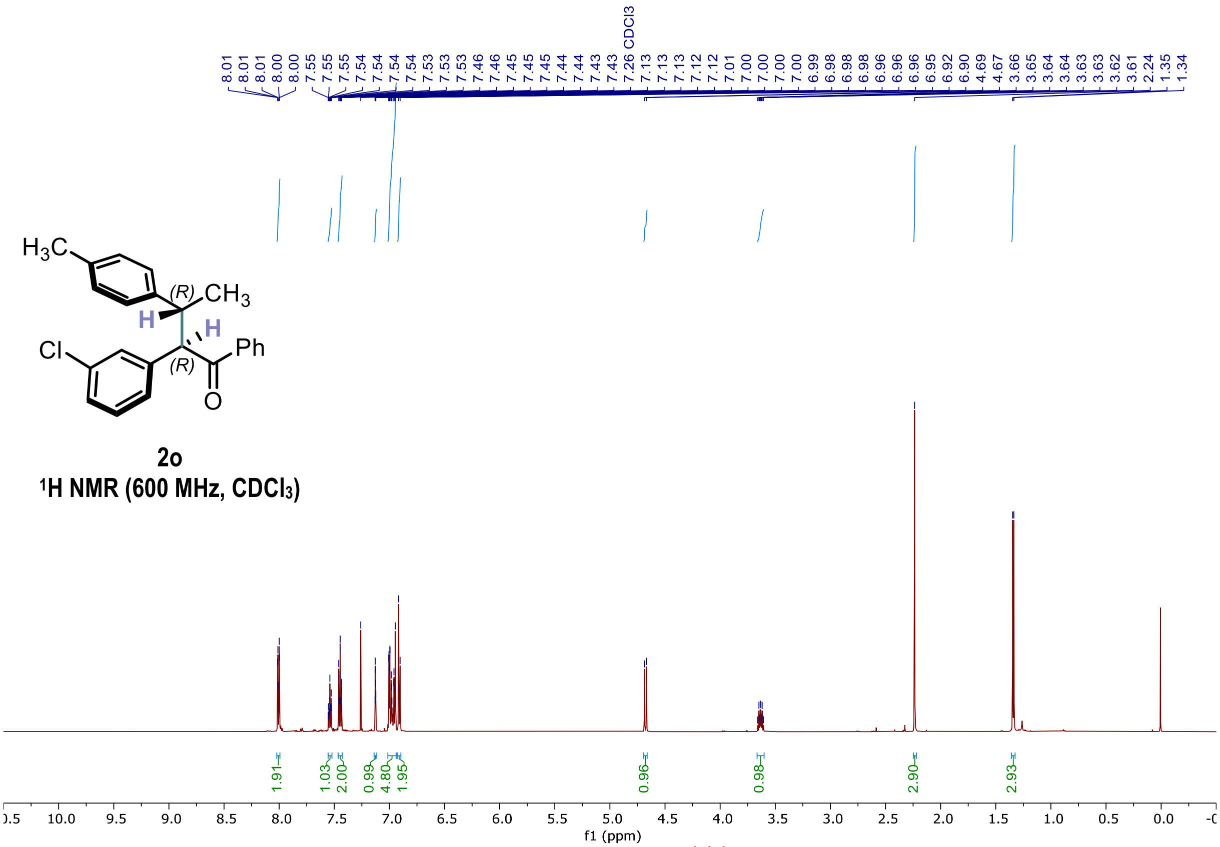

2o

^{1}H NMR (600 MHz, $CDCl_3$)

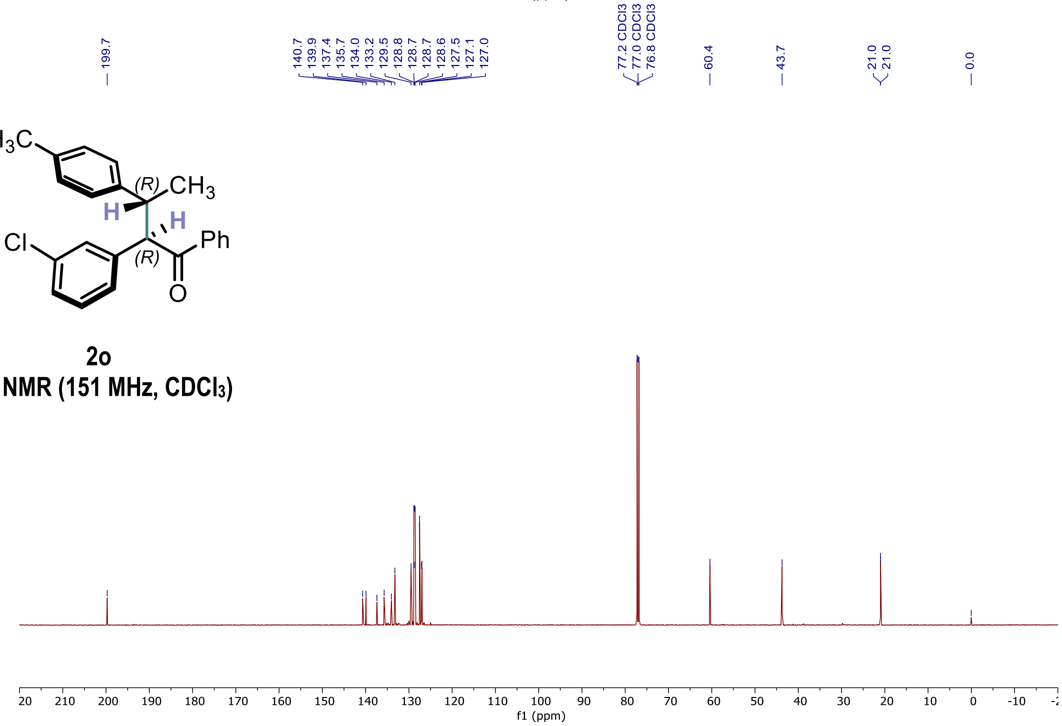

2o

^{13}C NMR (151 MHz, $CDCl_3$)

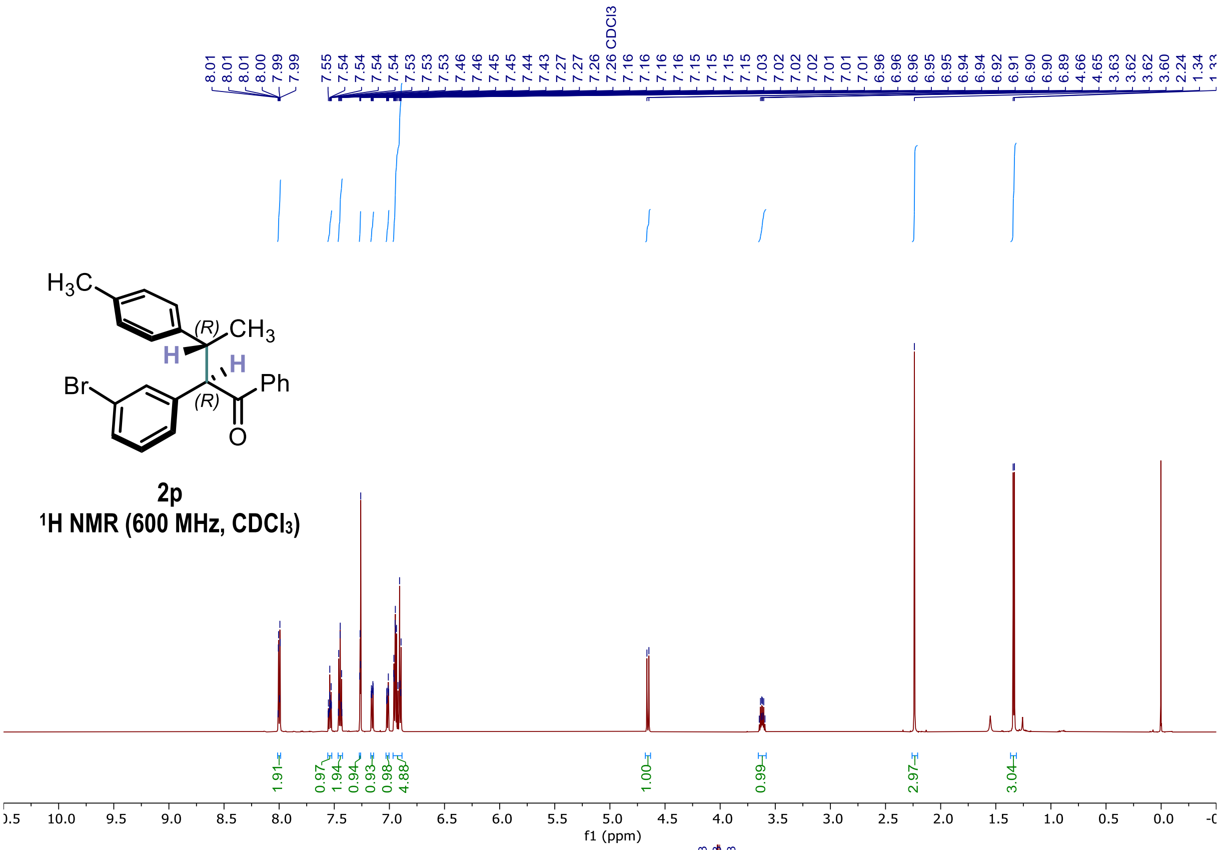

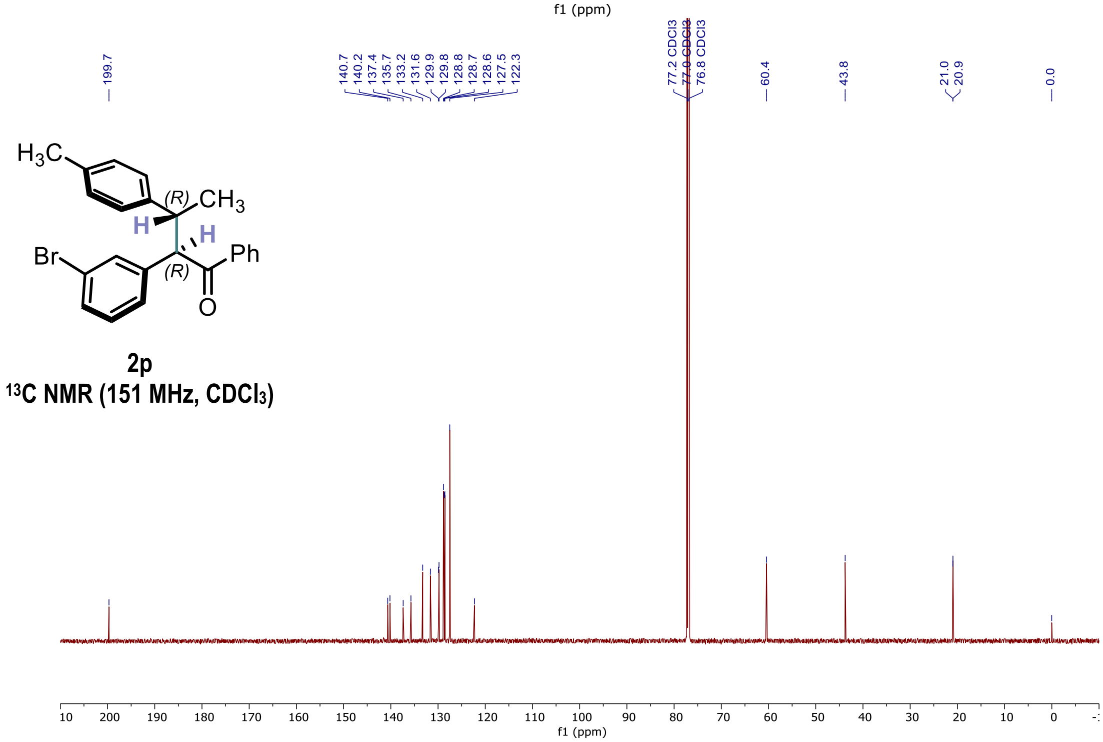

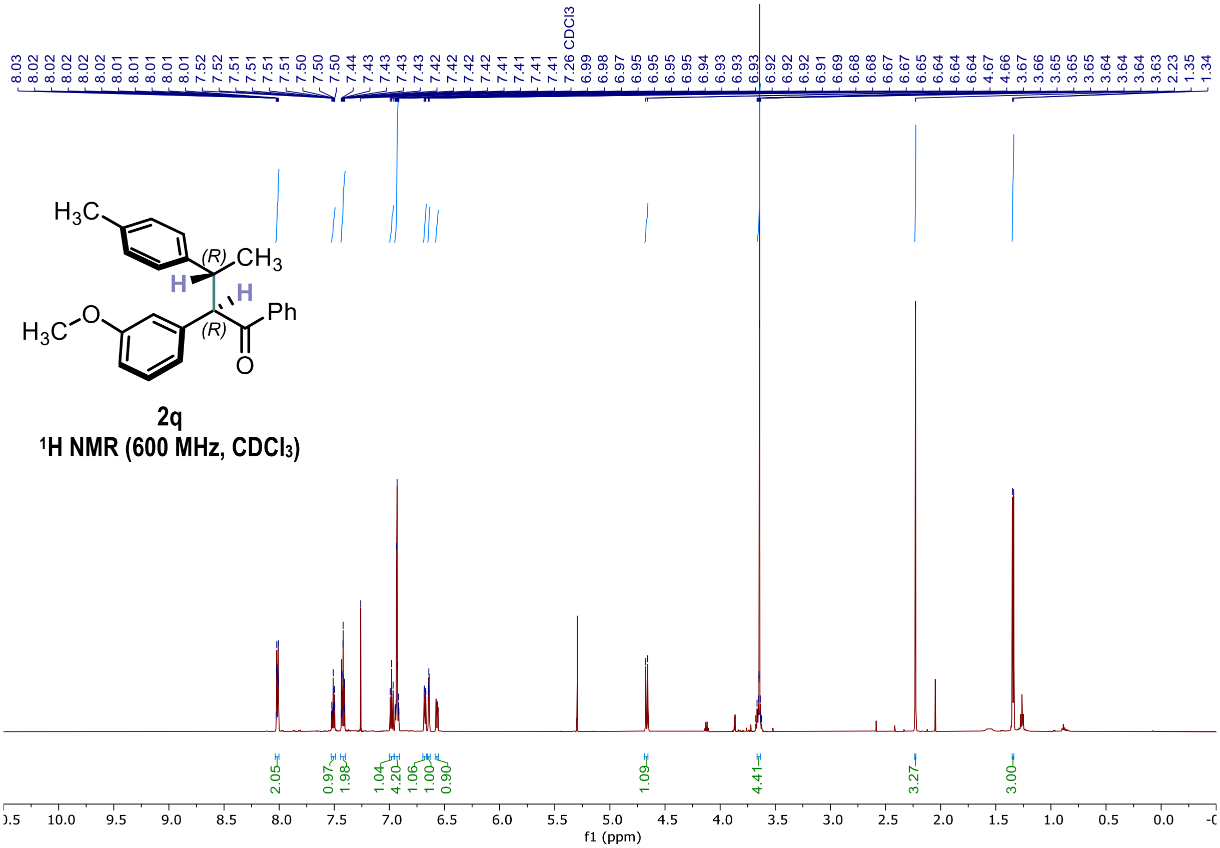

2q
^{1}H NMR (600 MHz, $CDCl_3$)

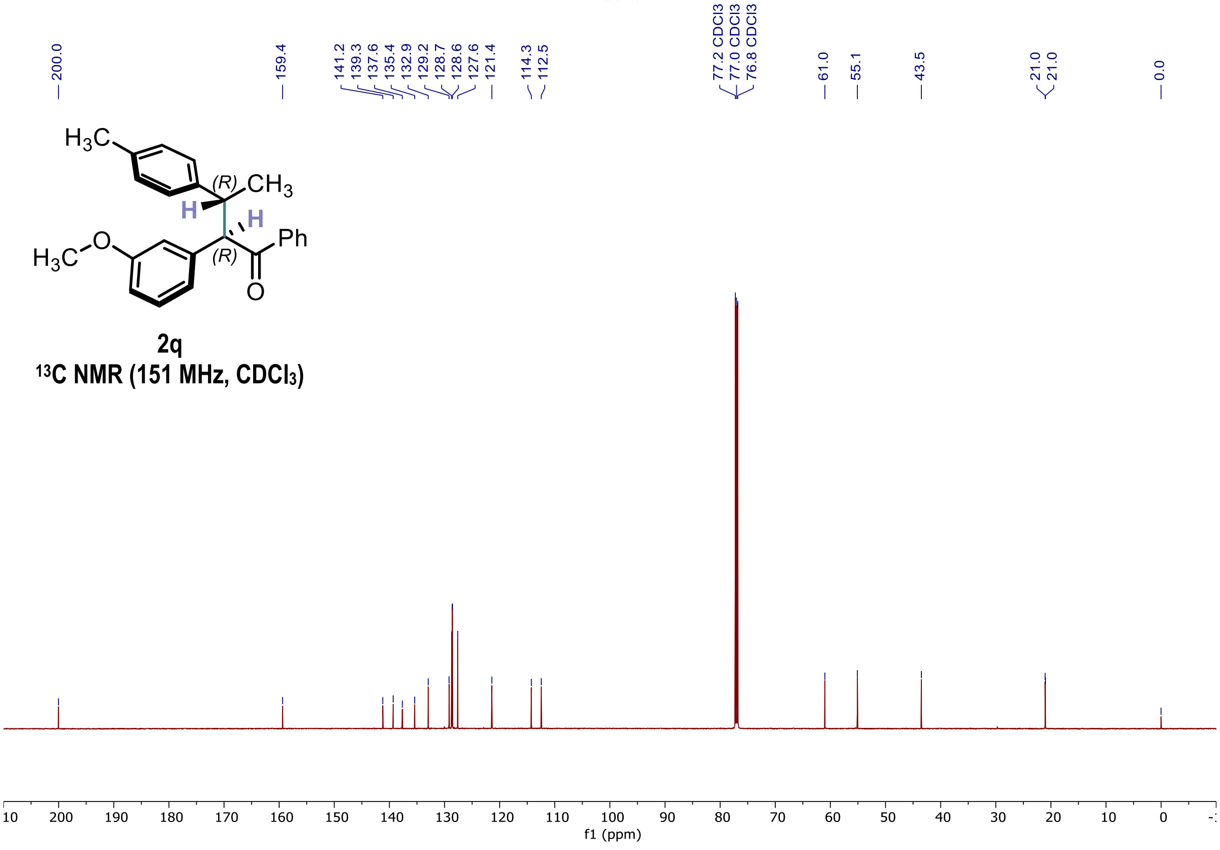

2q
^{13}C NMR (151 MHz, $CDCl_3$)

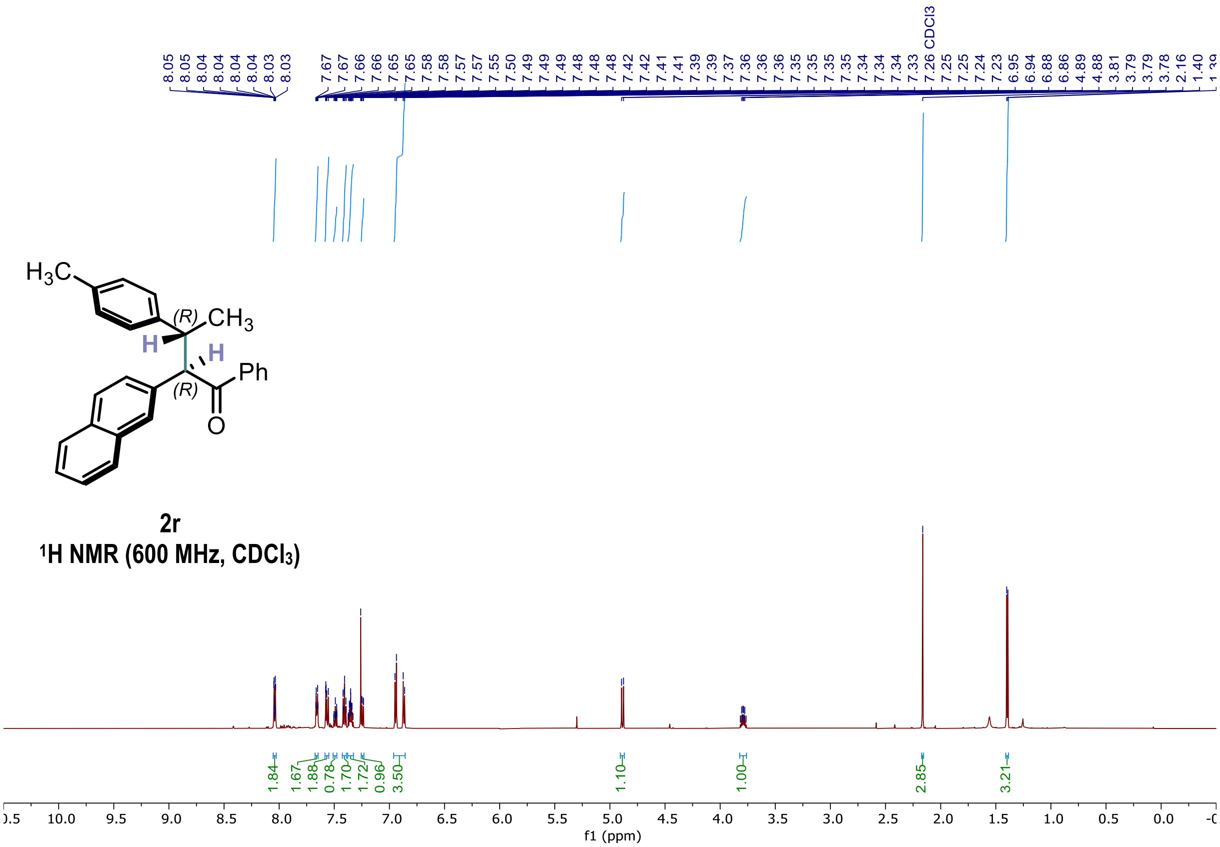

2r
^{1}H NMR (600 MHz, $CDCl_3$)
f1 (ppm)

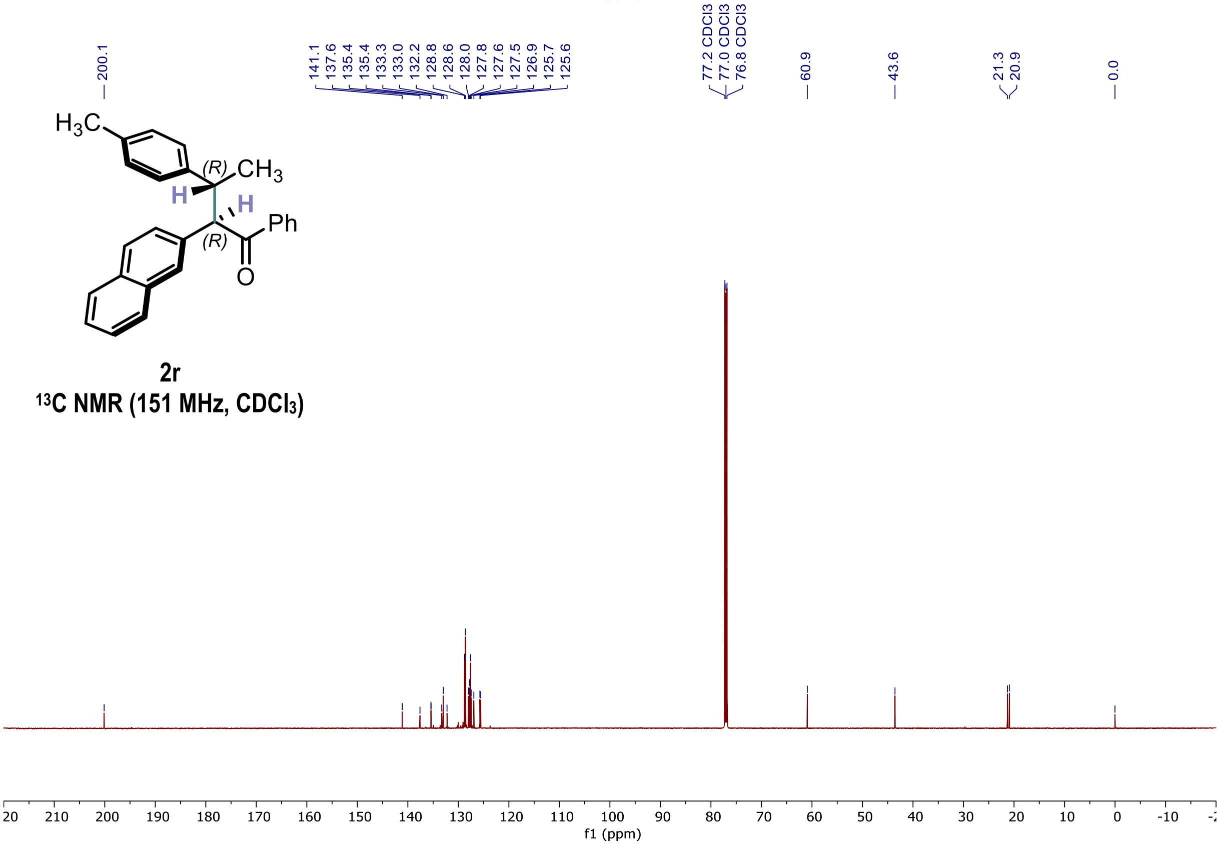

2r
^{13}C NMR (151 MHz, $CDCl_3$)
f1 (ppm)

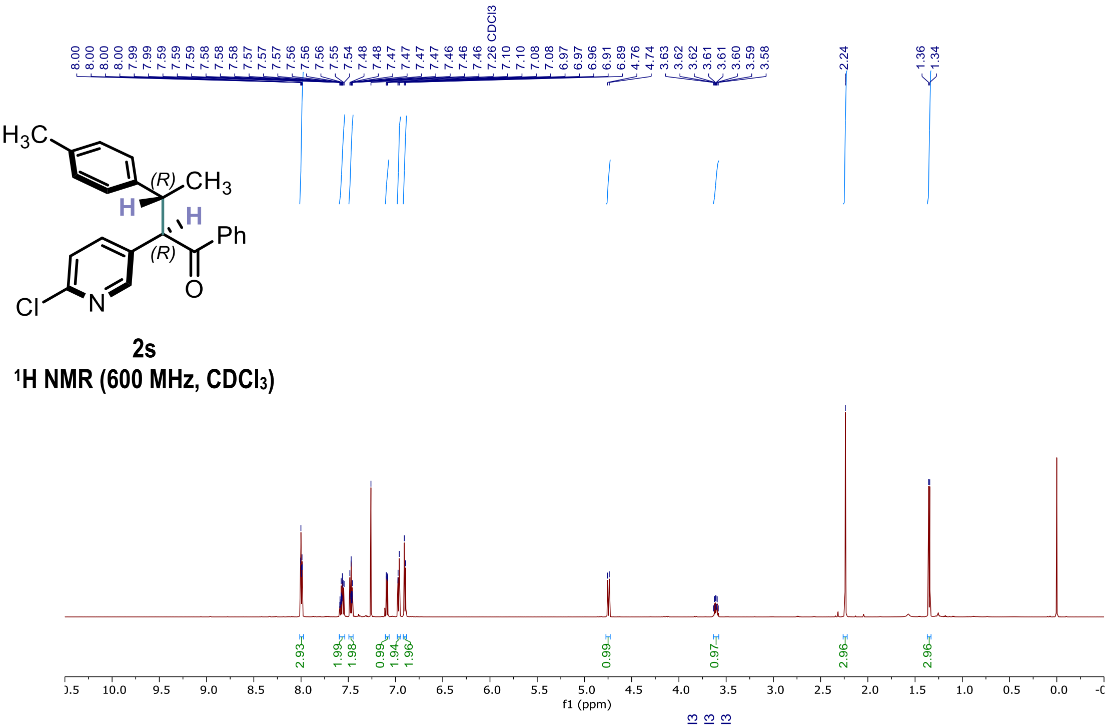

2s

^{1}H NMR (600 MHz, $CDCl_3$)

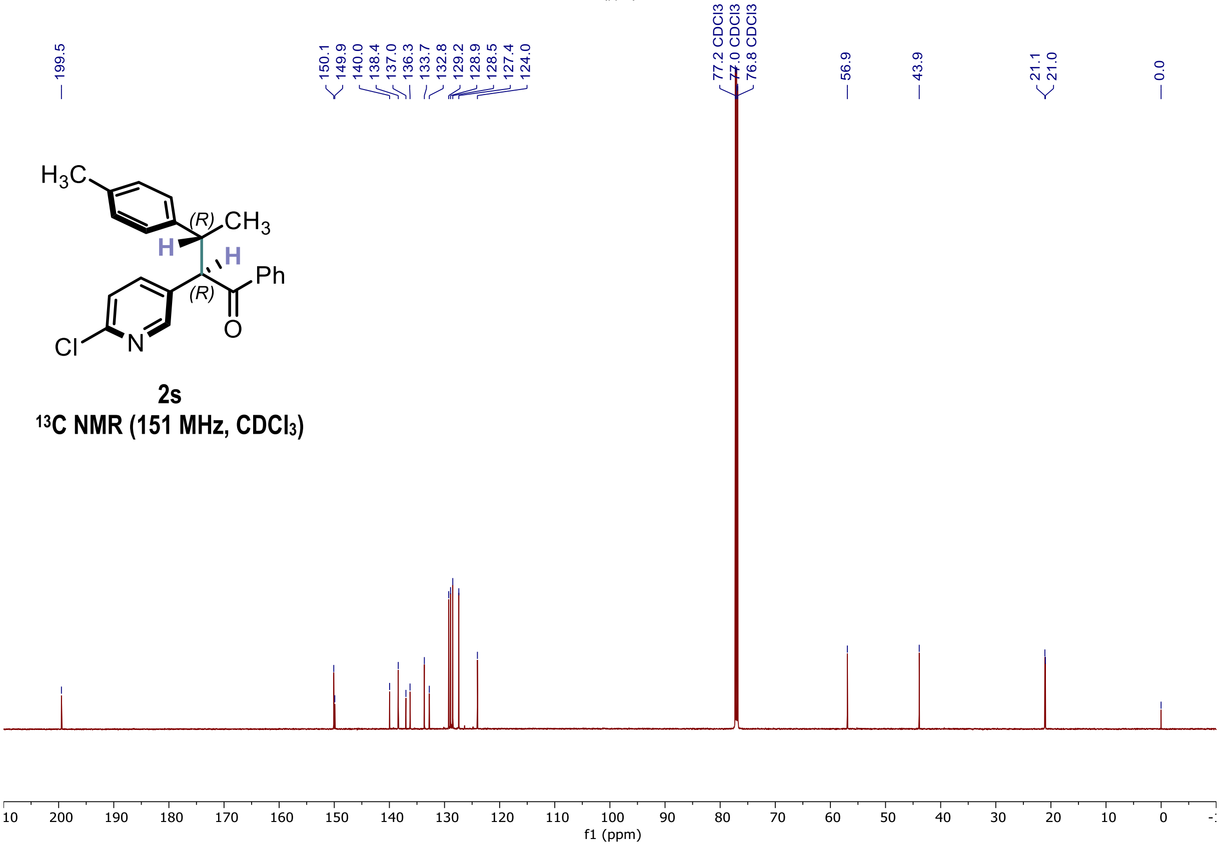

2s

^{13}C NMR (151 MHz, $CDCl_3$)

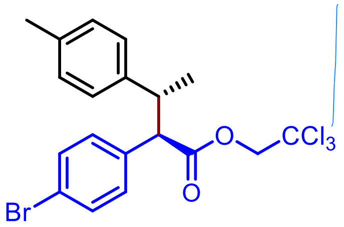

5

^{1}H NMR (400 MHz, $CDCl_3$)

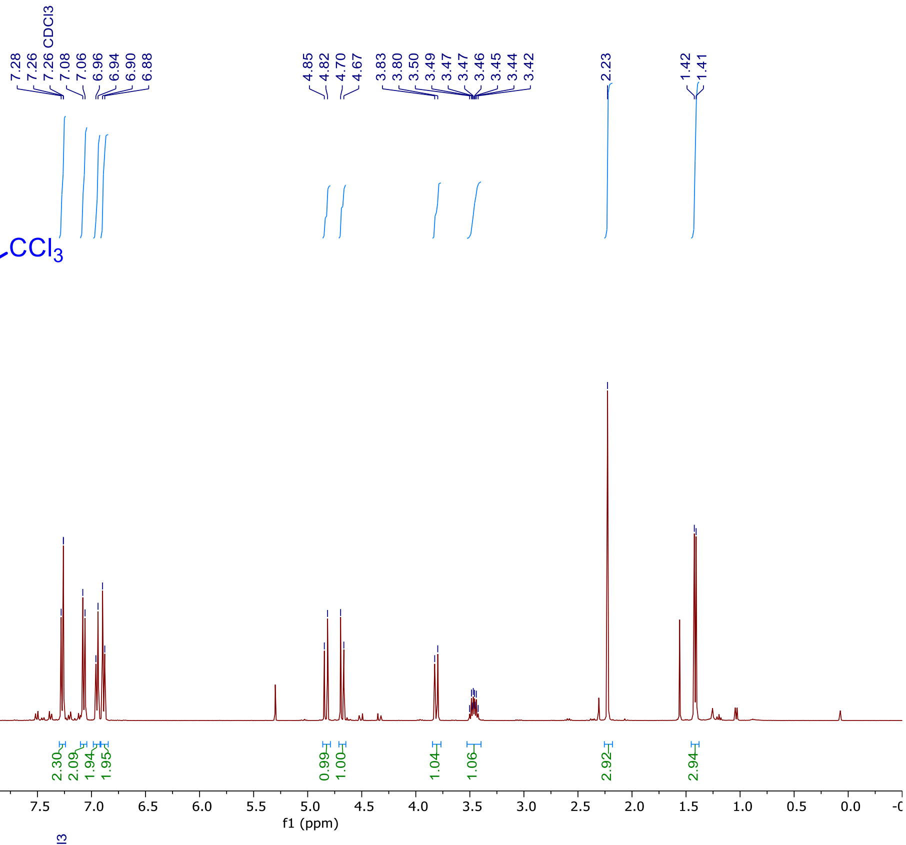

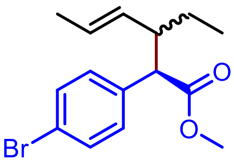

10

^{1}H NMR (400 MHz, $CDCl_3$)

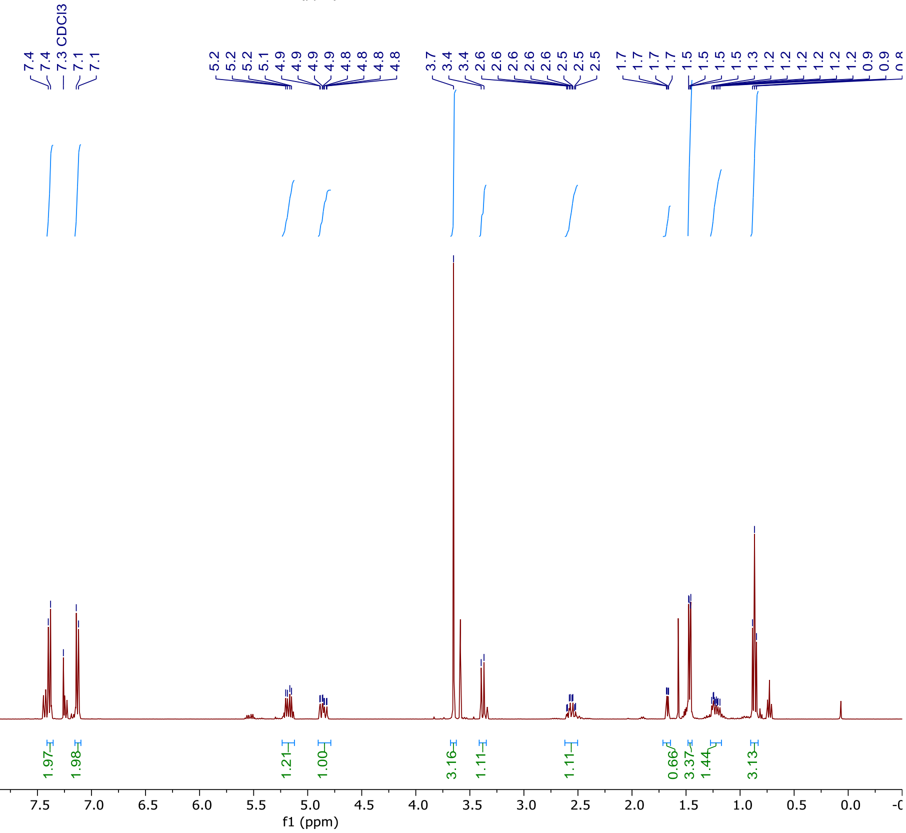

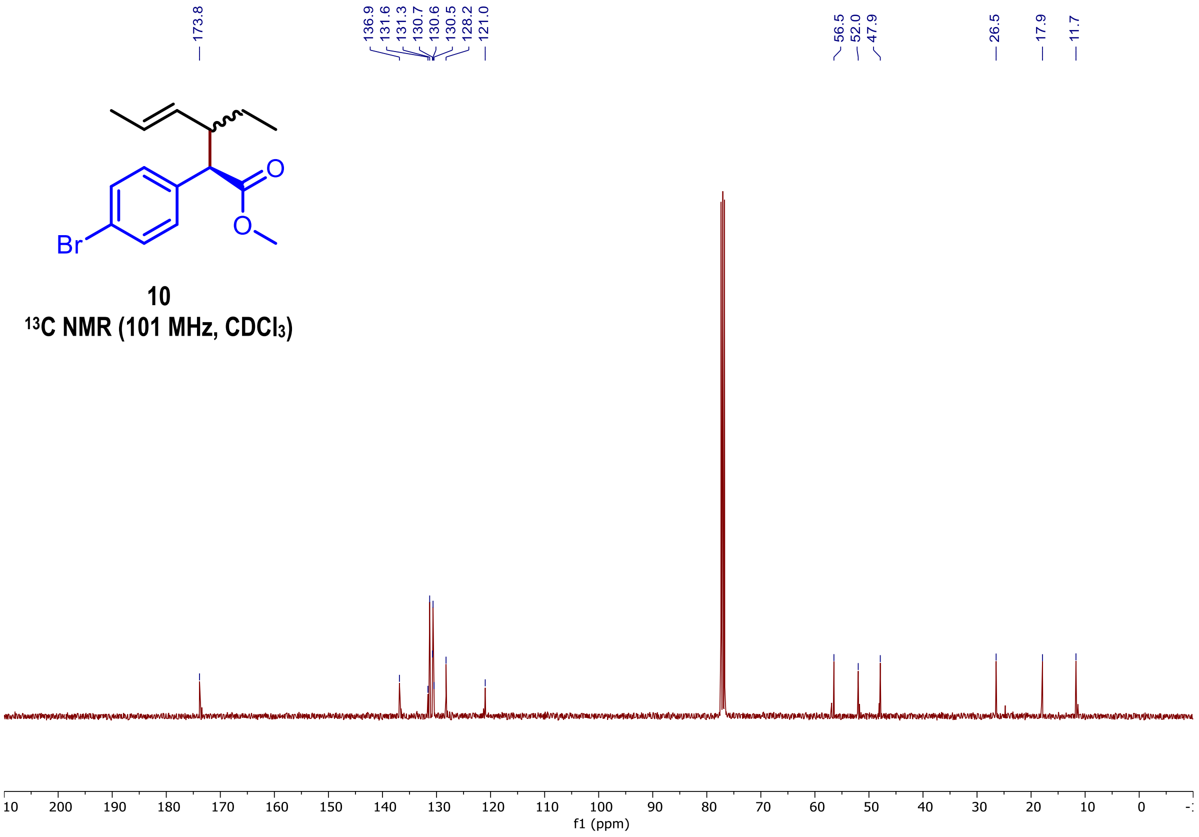

10
^{13}C NMR (101 MHz, $CDCl_3$)

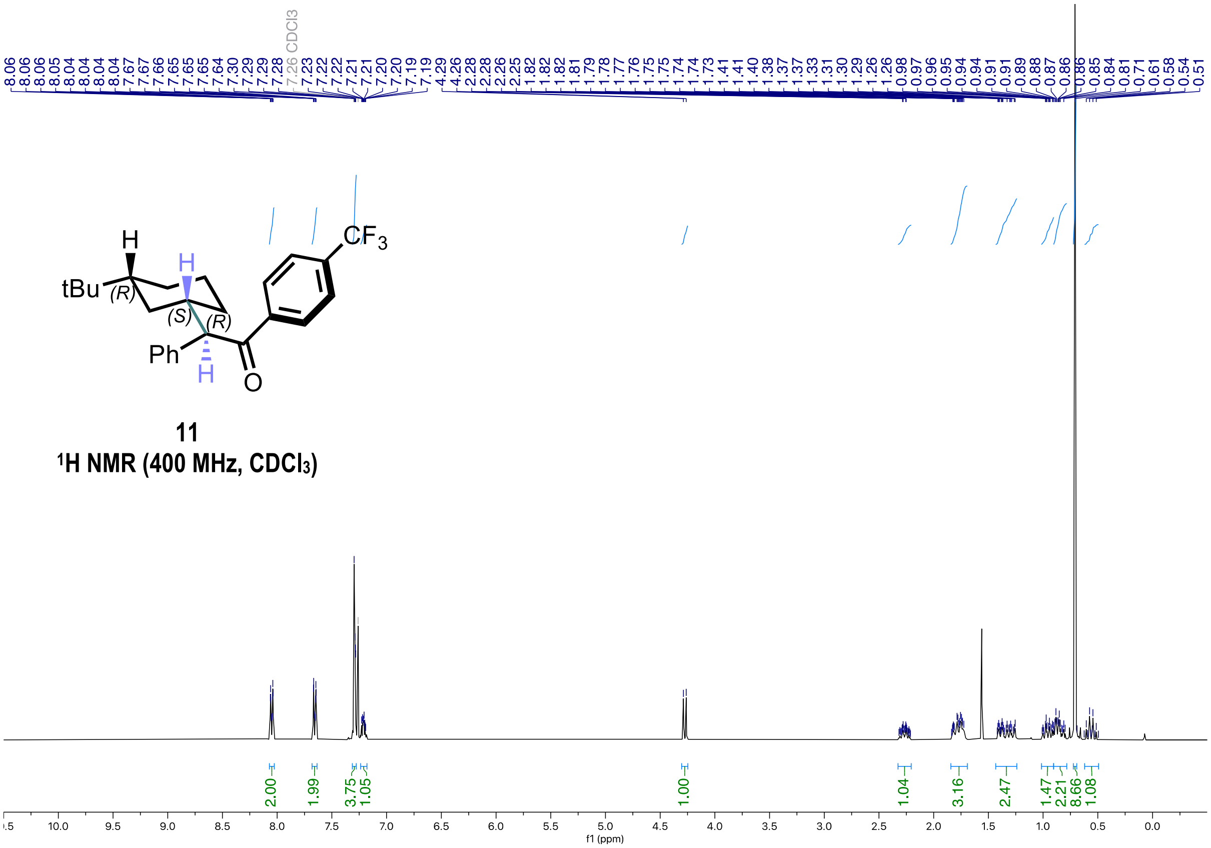

11
^{1}H NMR (400 MHz, $CDCl_3$)

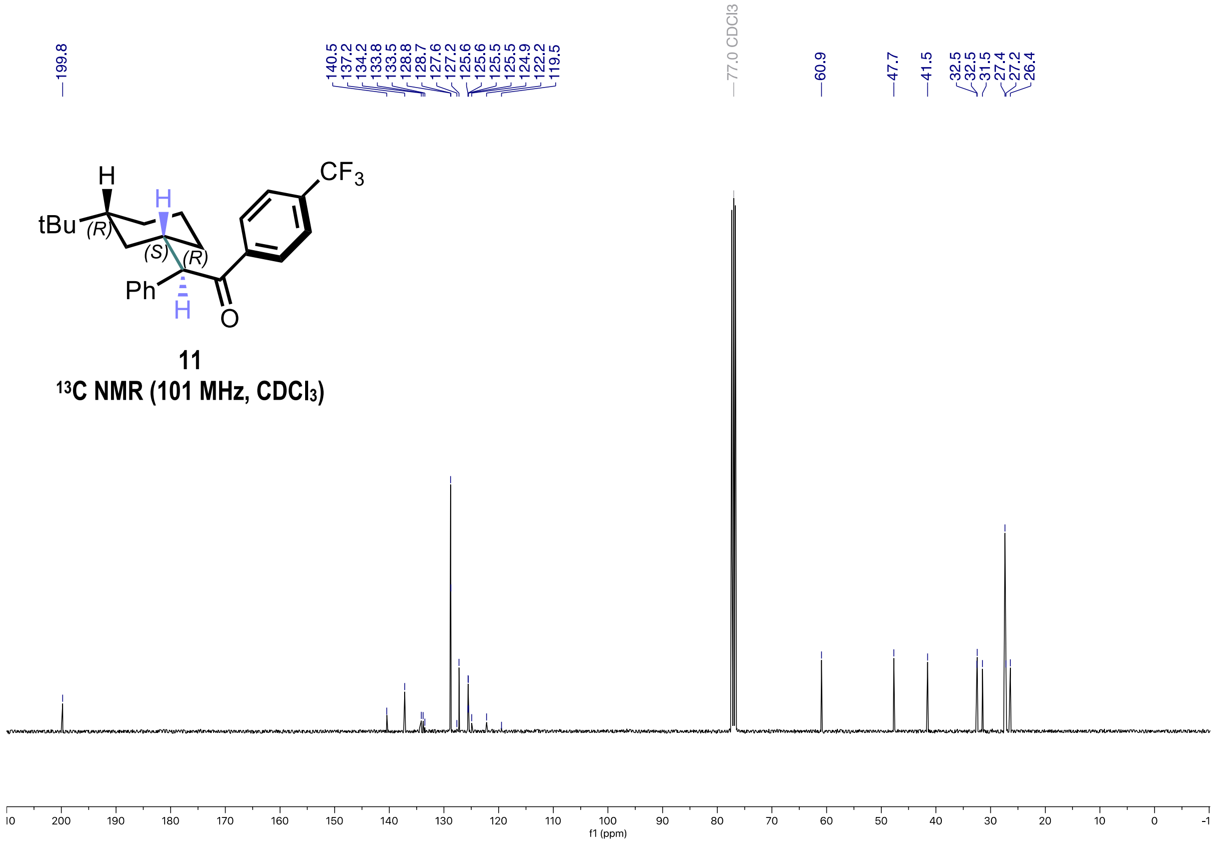

11

^{13}C NMR (101 MHz, $CDCl_3$)

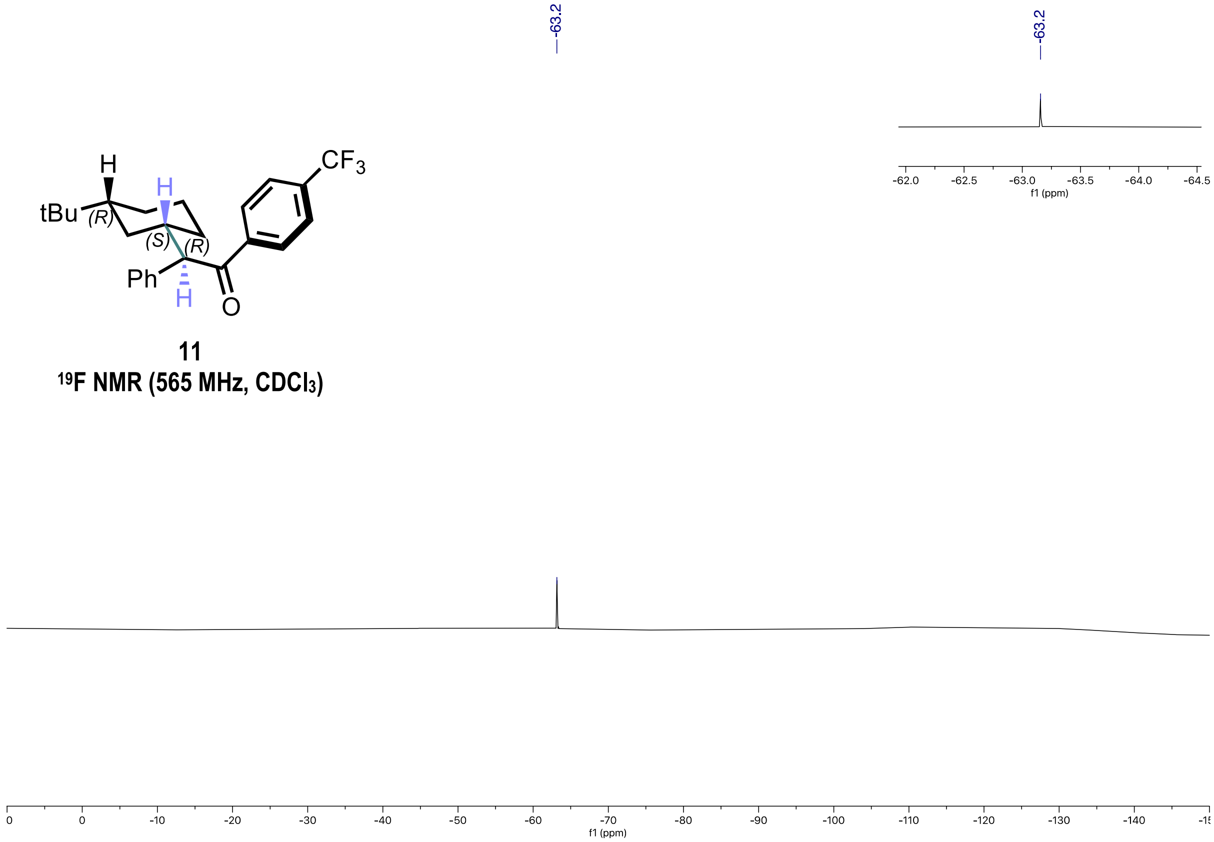

11

^{19}F NMR (565 MHz, $CDCl_3$)

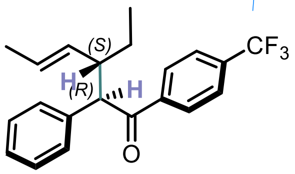

12

^{1}H NMR (600 MHz, $CDCl_3$)

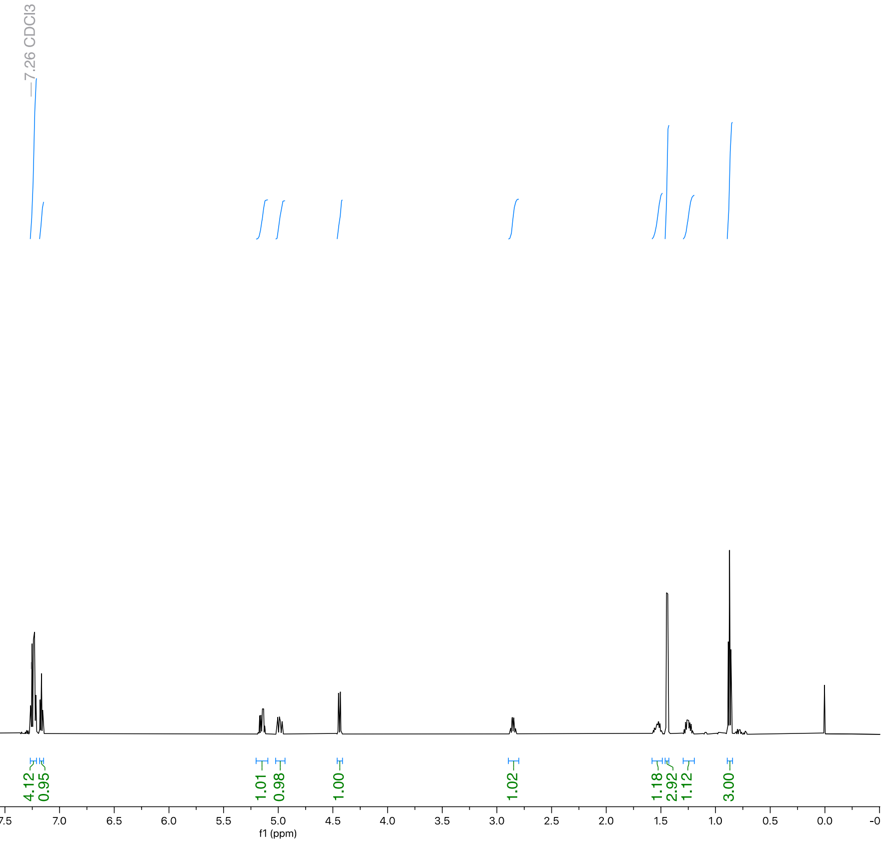

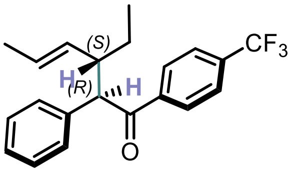

12

^{13}C NMR (151 MHz, $CDCl_3$)

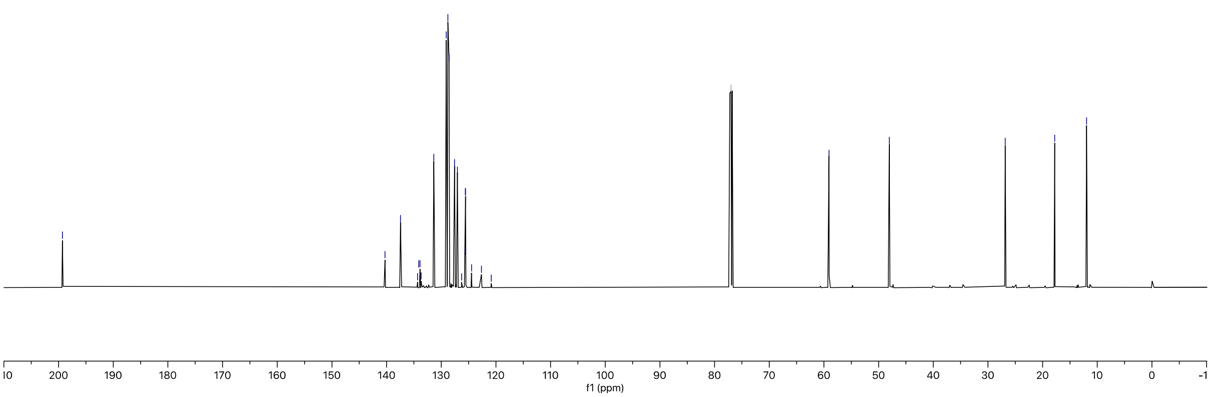

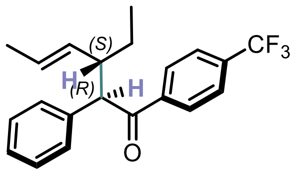

12
^{19}F NMR (565 MHz, CDCl$_3$)

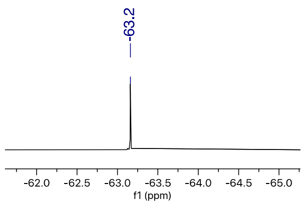

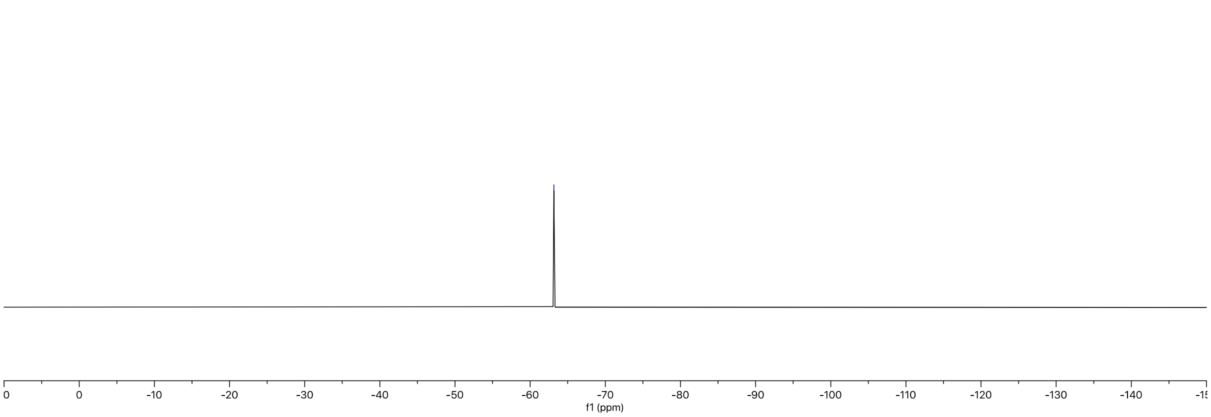

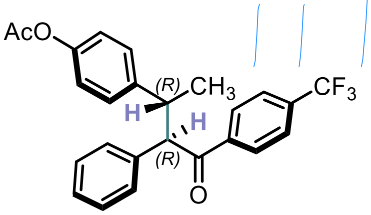

13
^{1}H NMR (400 MHz, CDCl$_3$)

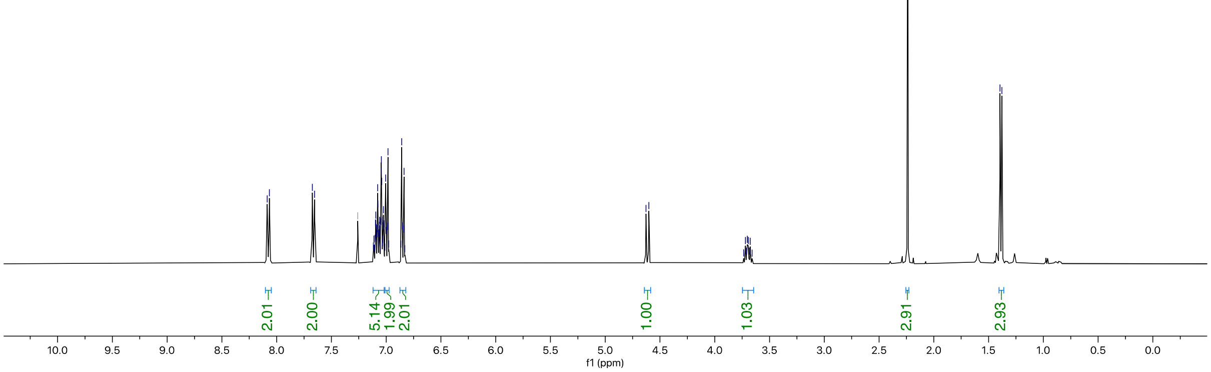

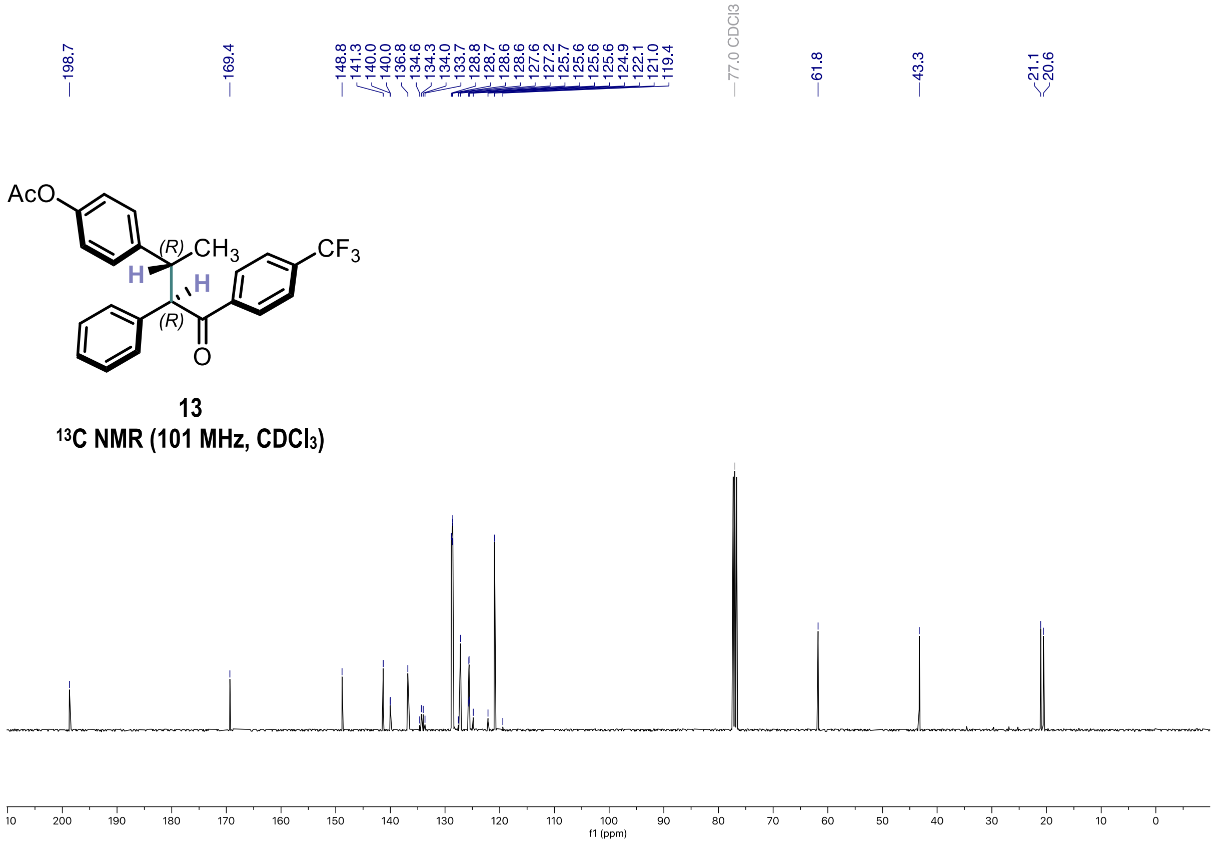
198.7
169.4
148.8
141.3
140.0
140.0
136.8
134.6
134.3
134.0
133.7
128.8
128.7
128.6
128.6
127.6
127.2
125.7
125.6
125.6
125.6
124.9
122.1
121.0
119.4
77.0 CDCl3
61.8
43.3
21.1
20.6
AcO
(R)
CH3
CF3
H
H
(R)
O
13
13C NMR (101 MHz, CDCl3)
f1 (ppm)

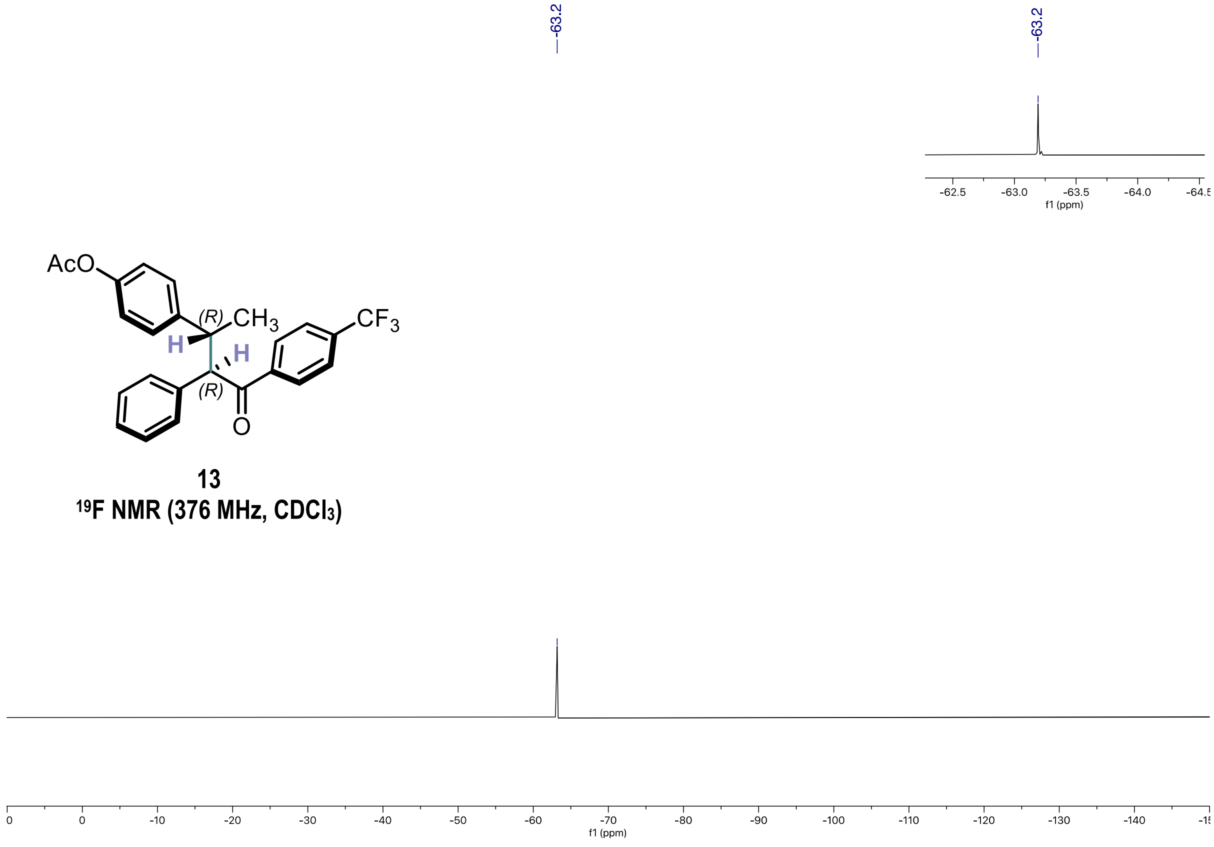
-63.2
-63.2
f1 (ppm)
AcO
(R)
CH3
CF3
H
H
(R)
O
13
19F NMR (376 MHz, CDCl3)
f1 (ppm)

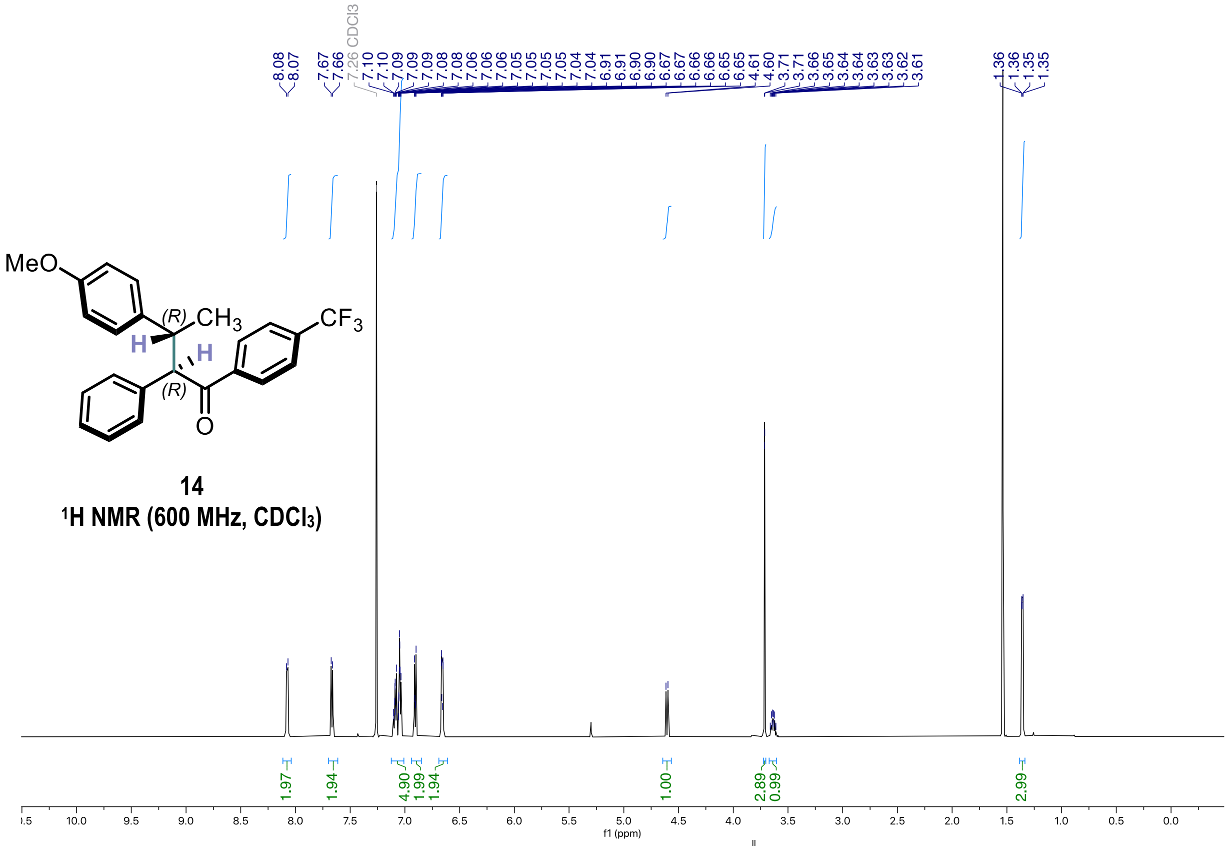

14
^{1}H NMR (600 MHz, $CDCl_3$)

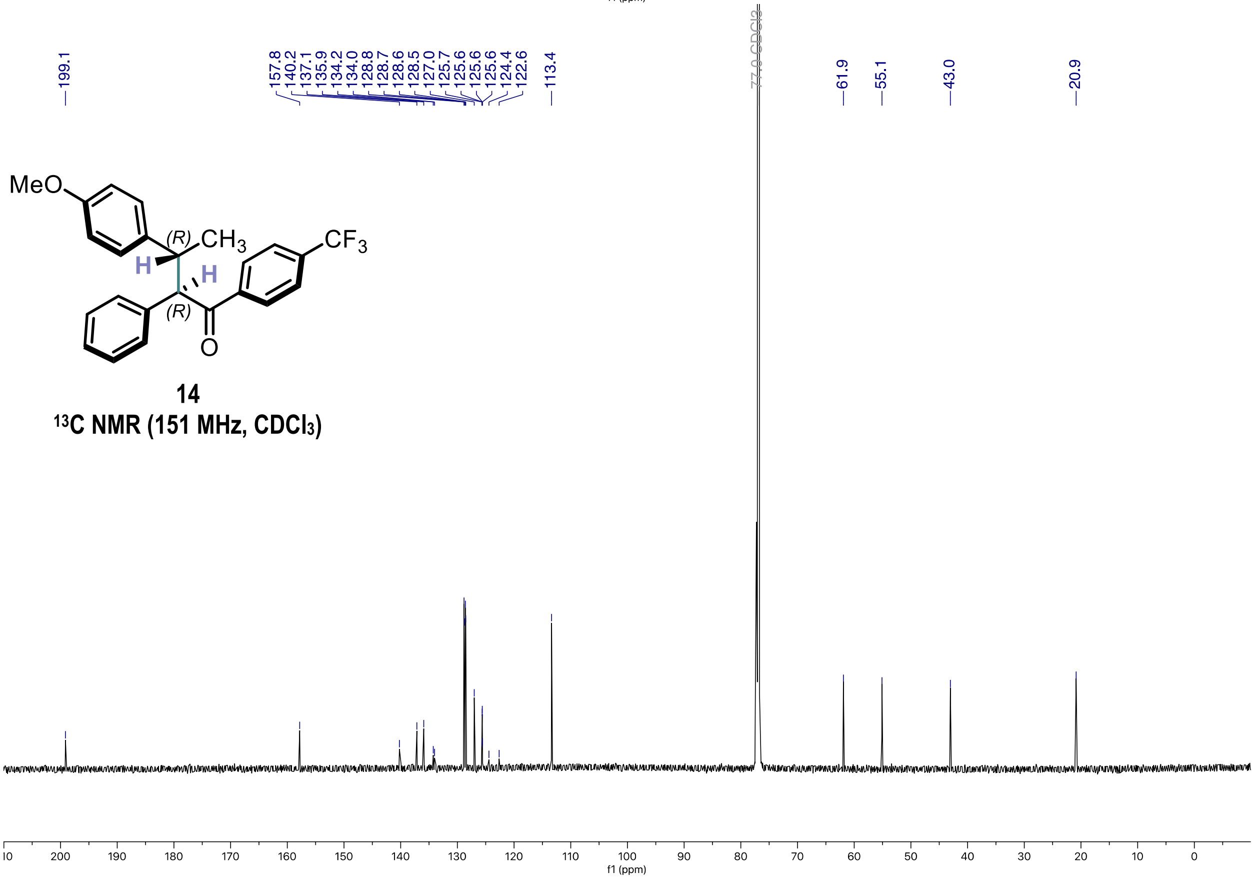

14
^{13}C NMR (151 MHz, $CDCl_3$)

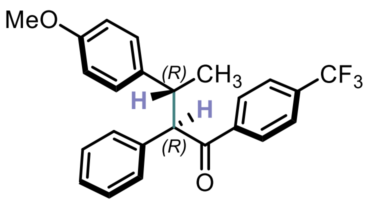

14

^{19}F NMR (565 MHz, $CDCl_3$)

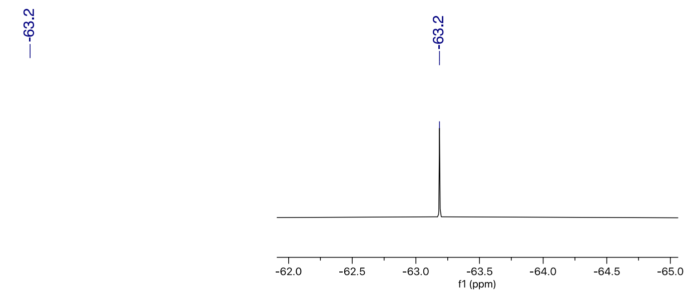

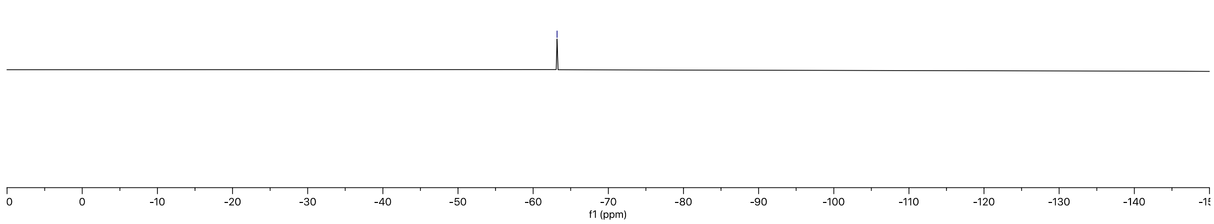

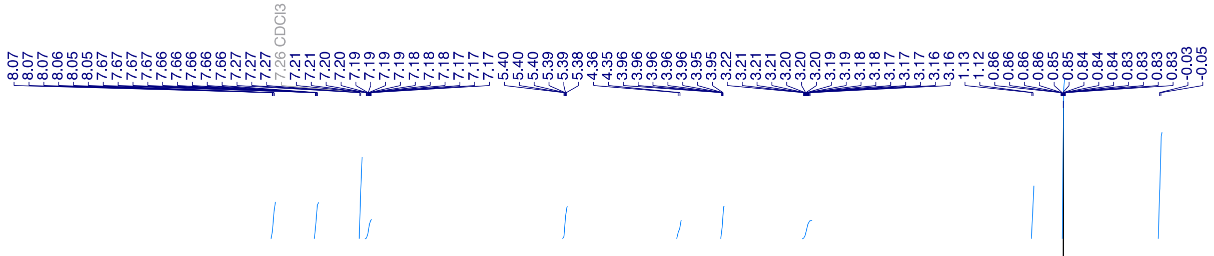

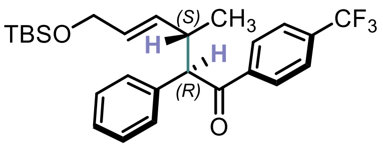

15

^{1}H NMR (600 MHz, $CDCl_3$)

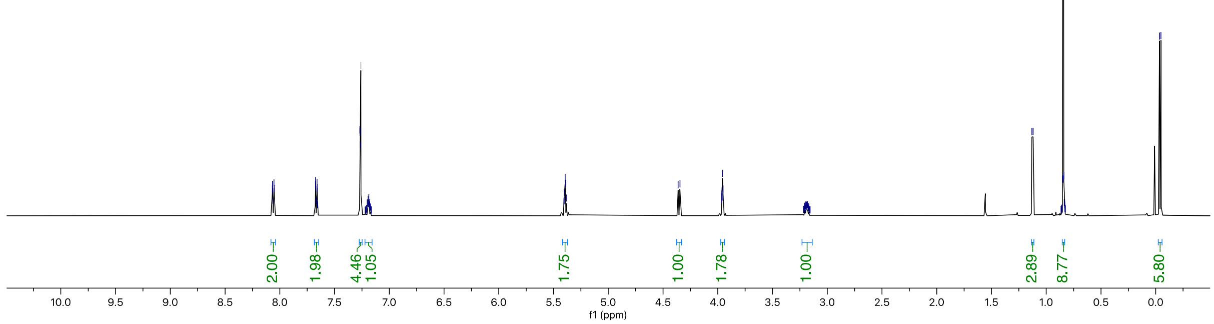

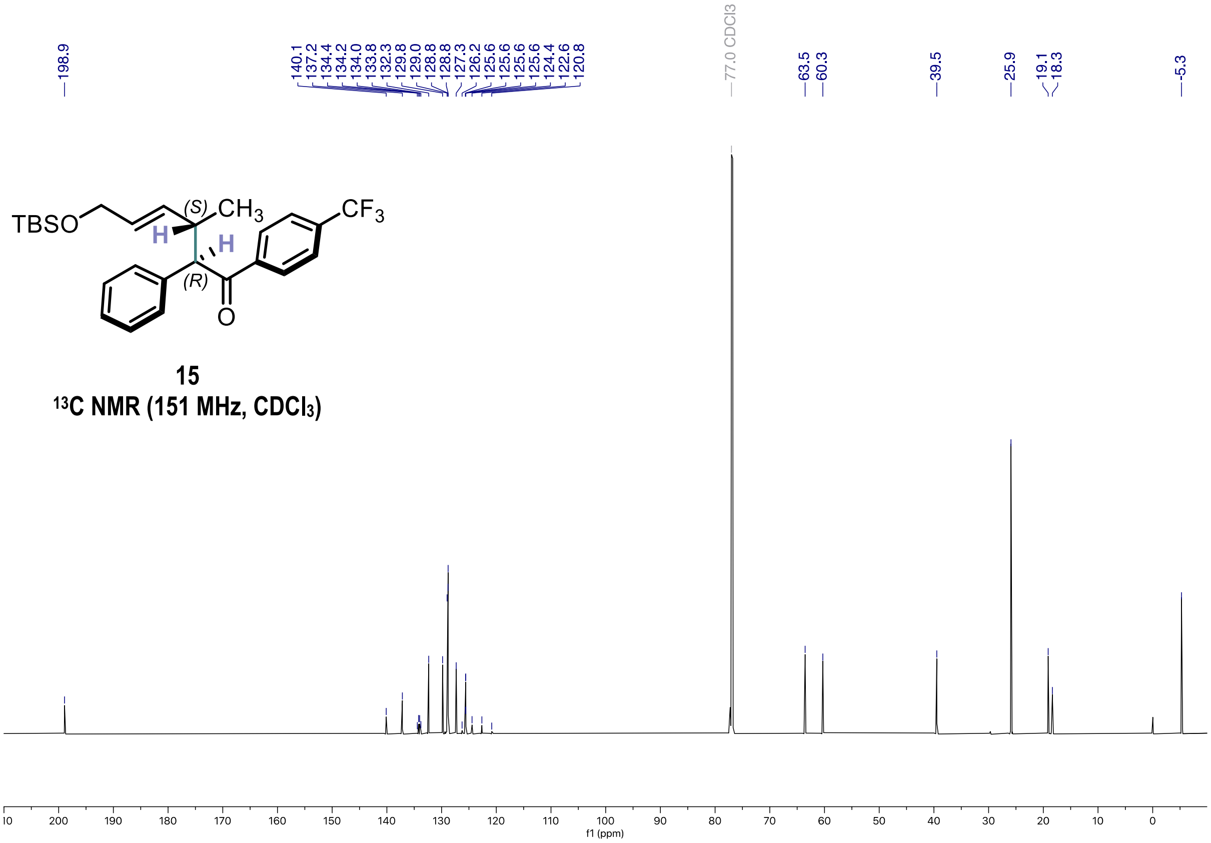

15
^{13}C NMR (151 MHz, $CDCl_3$)

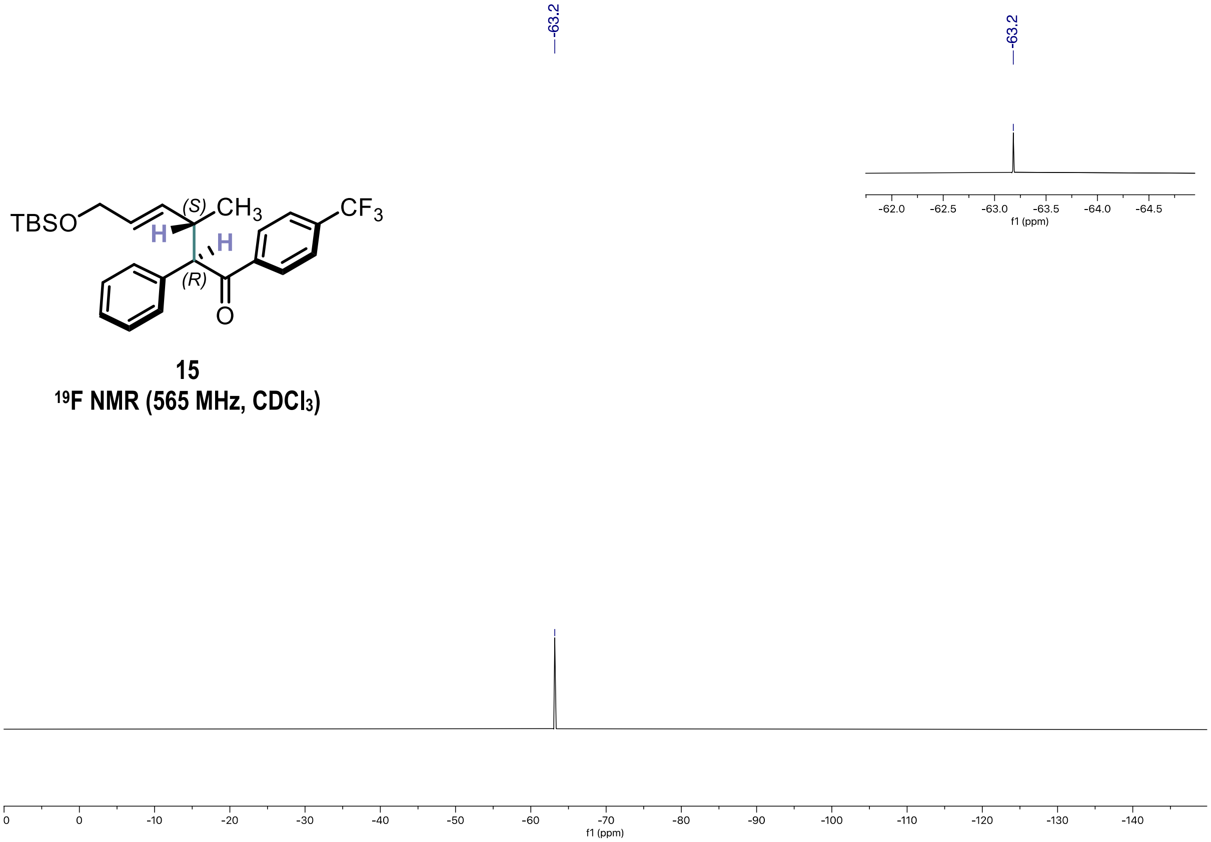

15
^{19}F NMR (565 MHz, $CDCl_3$)

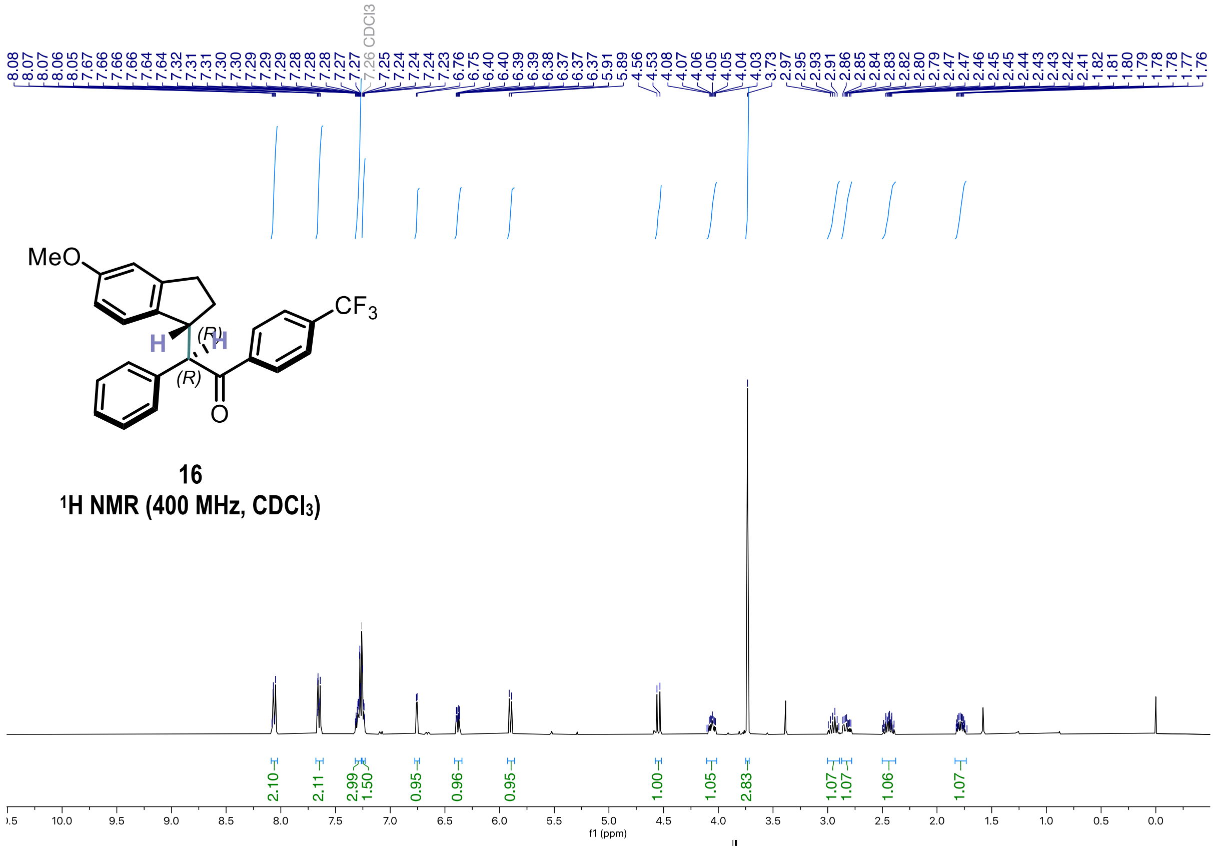

16

^{1}H NMR (400 MHz, $CDCl_3$)

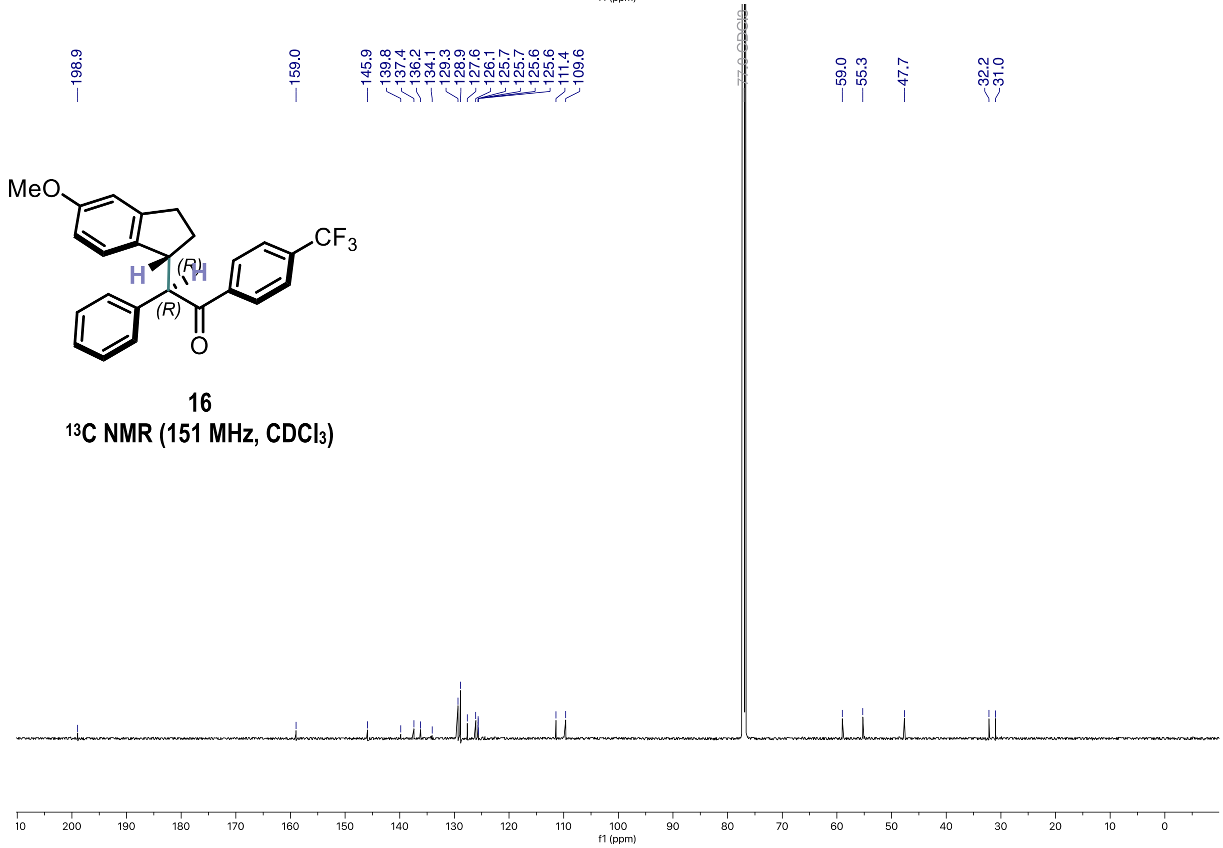

16

^{13}C NMR (151 MHz, $CDCl_3$)

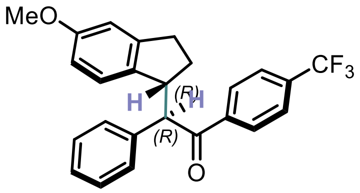

16

^{19}F NMR (376 MHz, CDCl$_3$)

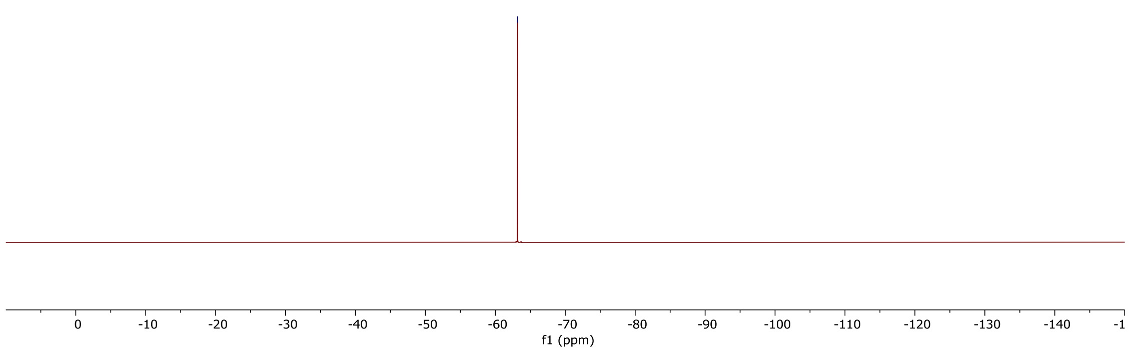

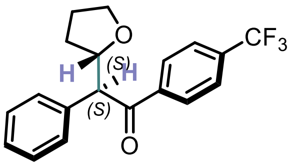

17

^{1}H NMR (600 MHz, CDCl$_3$)

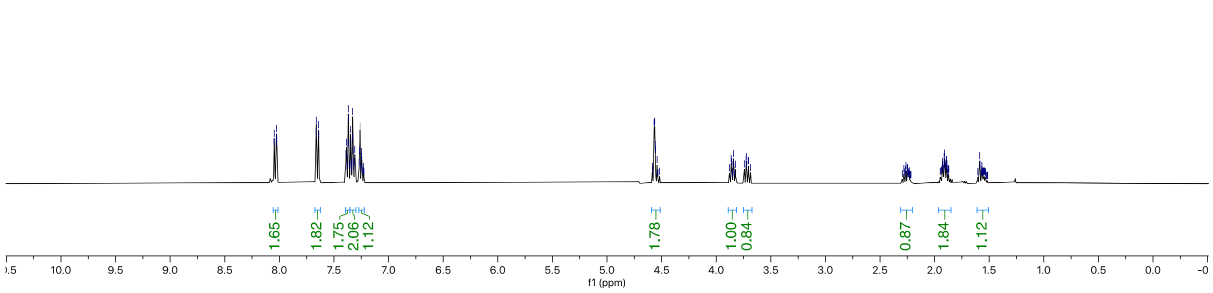

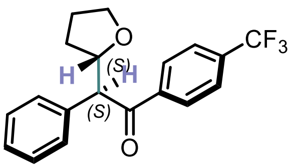

17

^{13}C NMR (151 MHz, CDCl$_3$)

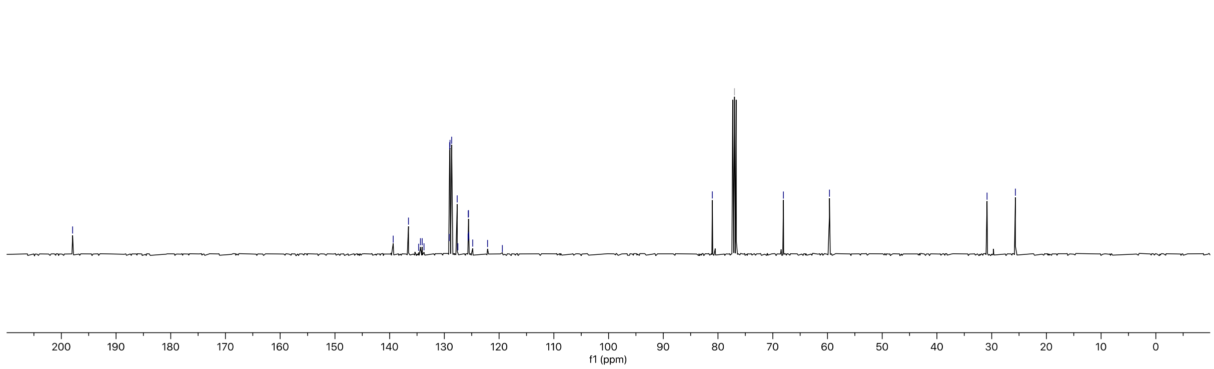

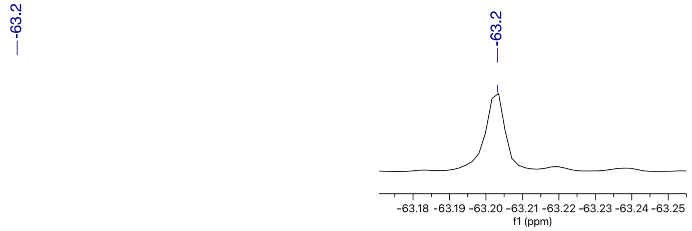

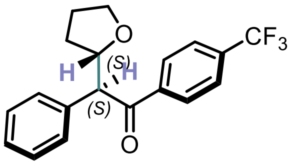

17

^{19}F NMR (565 MHz, CDCl$_3$)

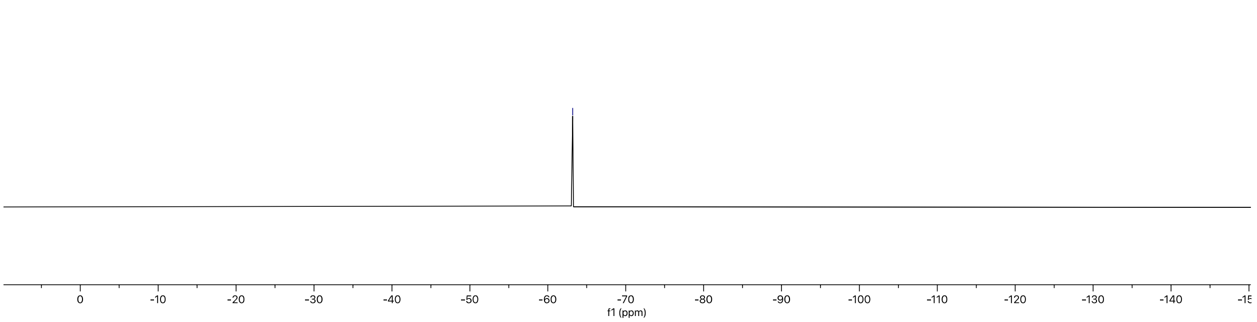

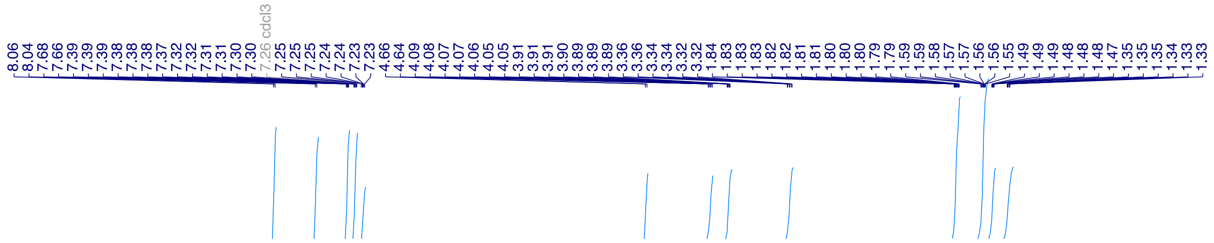

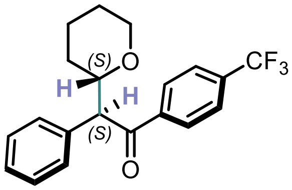

18

^{1}H NMR (600 MHz, $CDCl_3$)

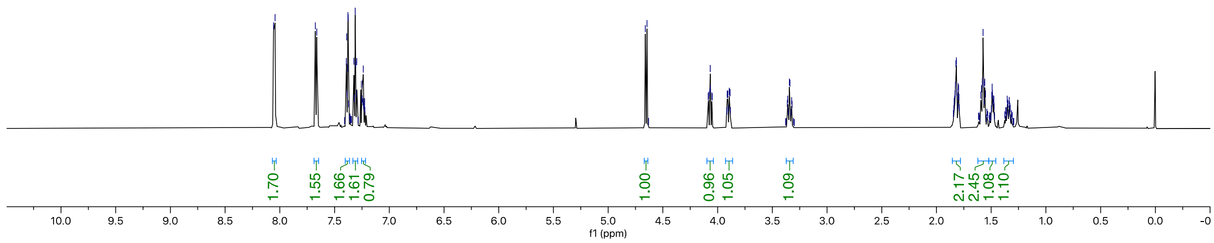

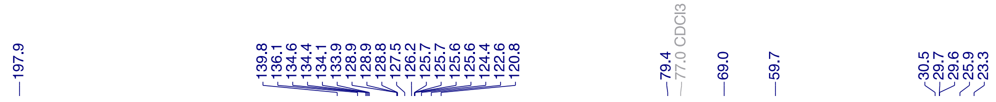

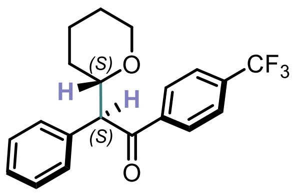

18

^{13}C NMR (151 MHz, $CDCl_3$)

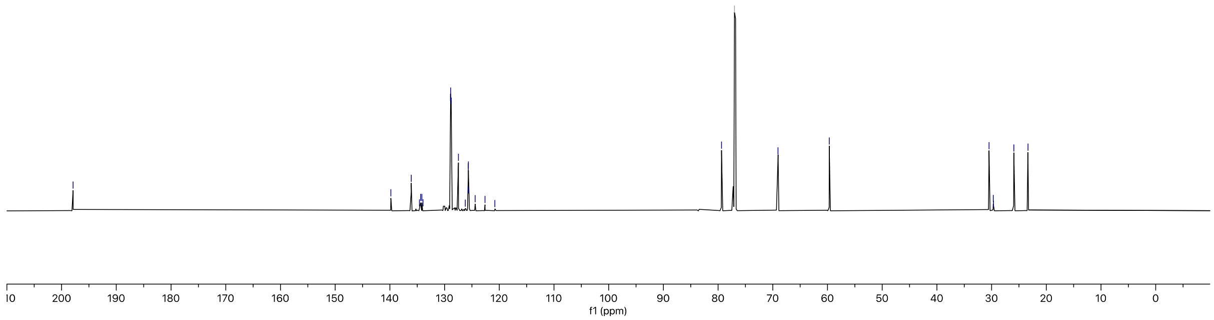

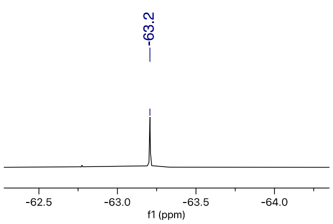

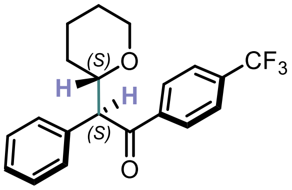

18

^{19}F NMR (565 MHz, $CDCl_3$)

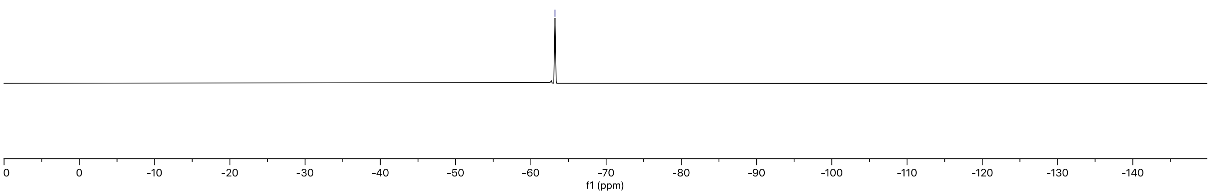

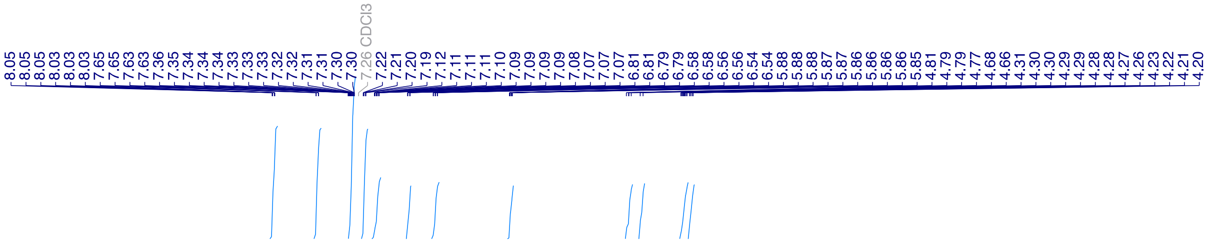

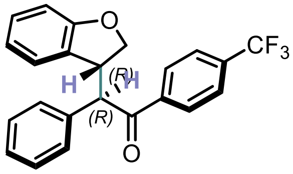

19

^{1}H NMR (400 MHz, $CDCl_3$)

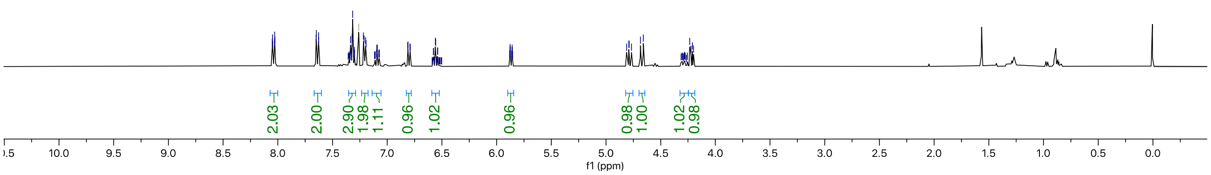

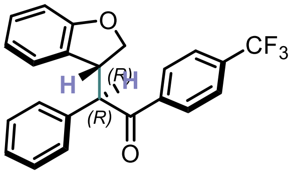

19

^{13}C NMR (101 MHz, CDCl$_3$)

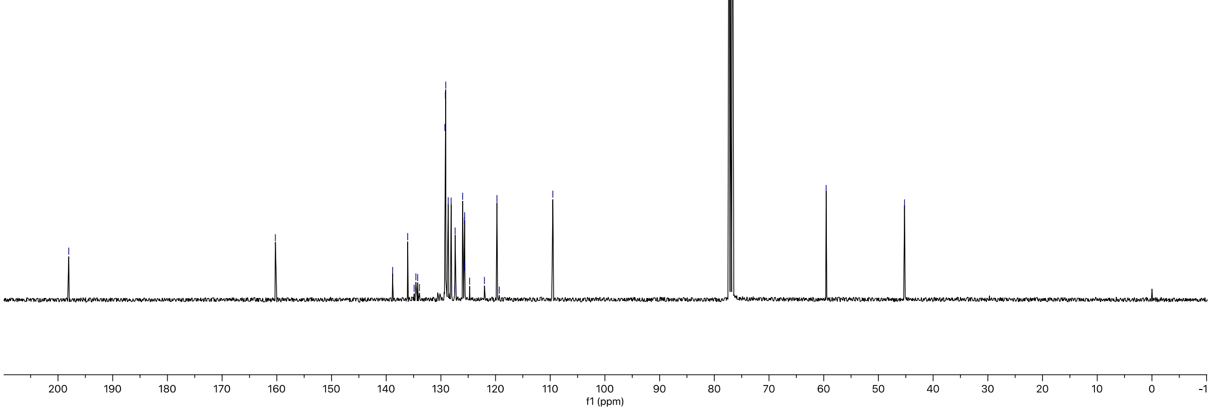

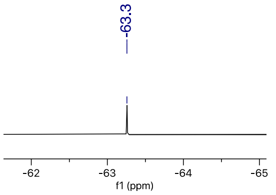

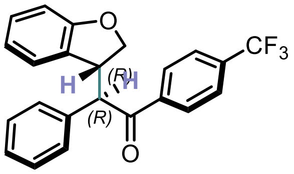

19

^{19}F NMR (376 MHz, CDCl$_3$)

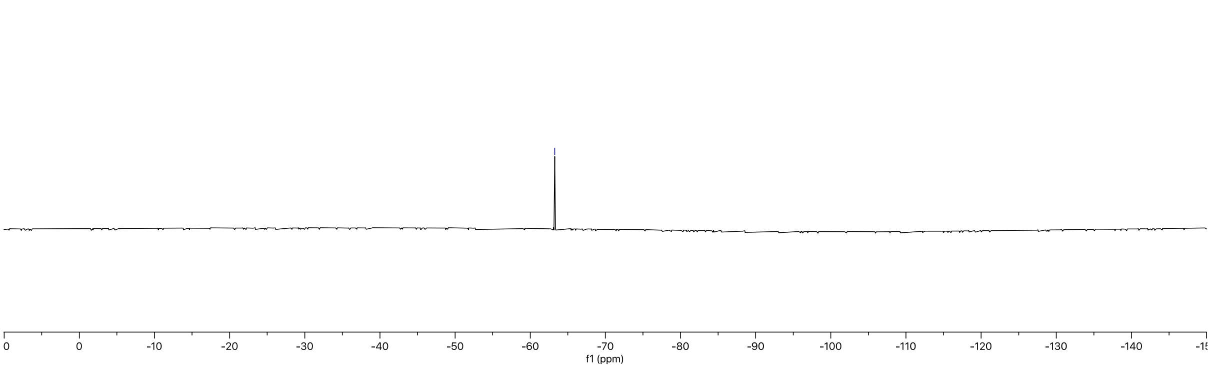

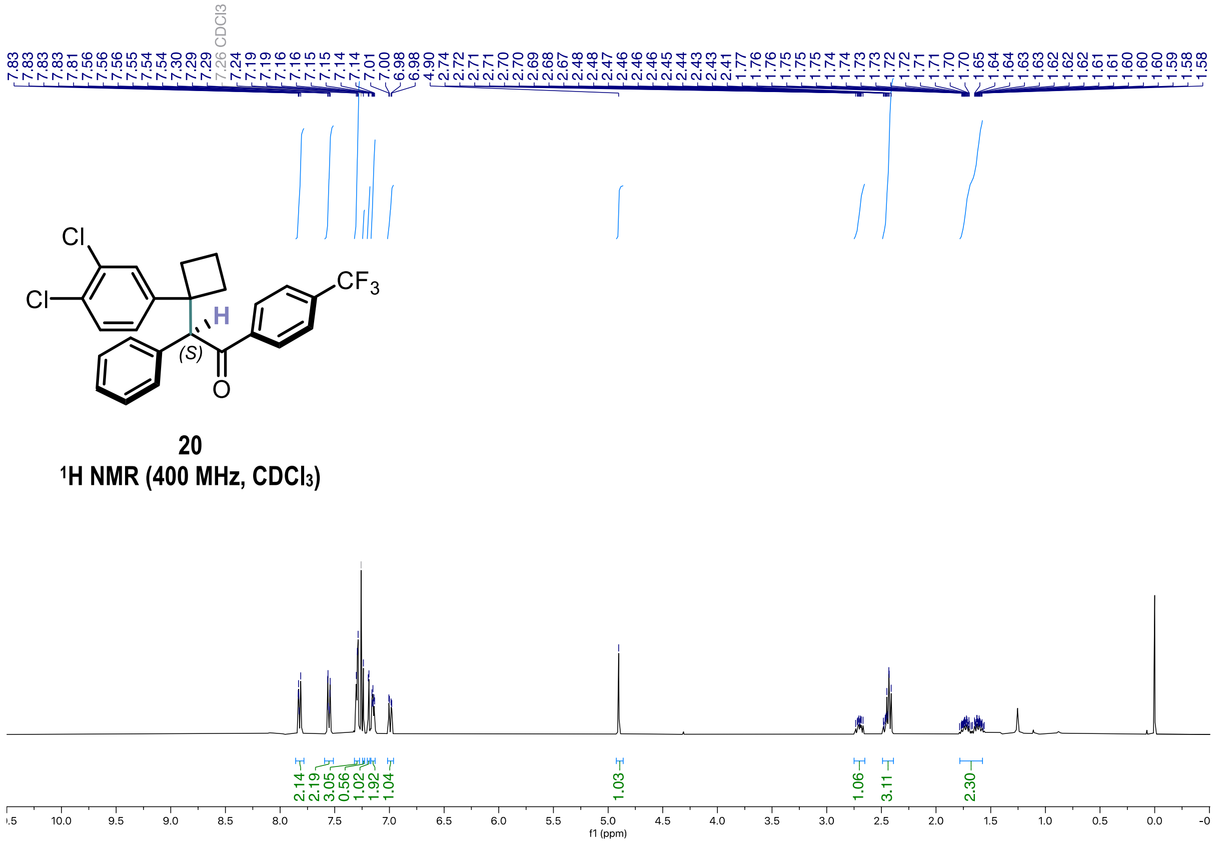
7.83 7.83 7.83 7.81 7.56 7.56 7.56 7.55 7.54 7.54 7.30 7.29 7.29 7.26 CDCl3 7.24 7.19 7.19 7.16 7.15 7.15 7.14 7.14 7.01 7.00 6.98 6.98 4.90 2.74 2.72 2.71 2.71 2.70 2.70 2.69 2.68 2.67 2.48 2.48 2.48 2.47 2.46 2.46 2.46 2.45 2.44 2.43 2.43 2.41 1.77 1.76 1.76 1.75 1.75 1.74 1.73 1.73 1.72 1.72 1.71 1.71 1.70 1.70 1.65 1.64 1.63 1.63 1.62 1.62 1.61 1.60 1.60 1.59 1.58
Cl
Cl
CF3
H
(S)
O
20
1H NMR (400 MHz, CDCl3)
2.14 2.19 3.05 0.56 1.02 1.92 1.04 1.03 1.06 3.11 2.30
f1 (ppm)

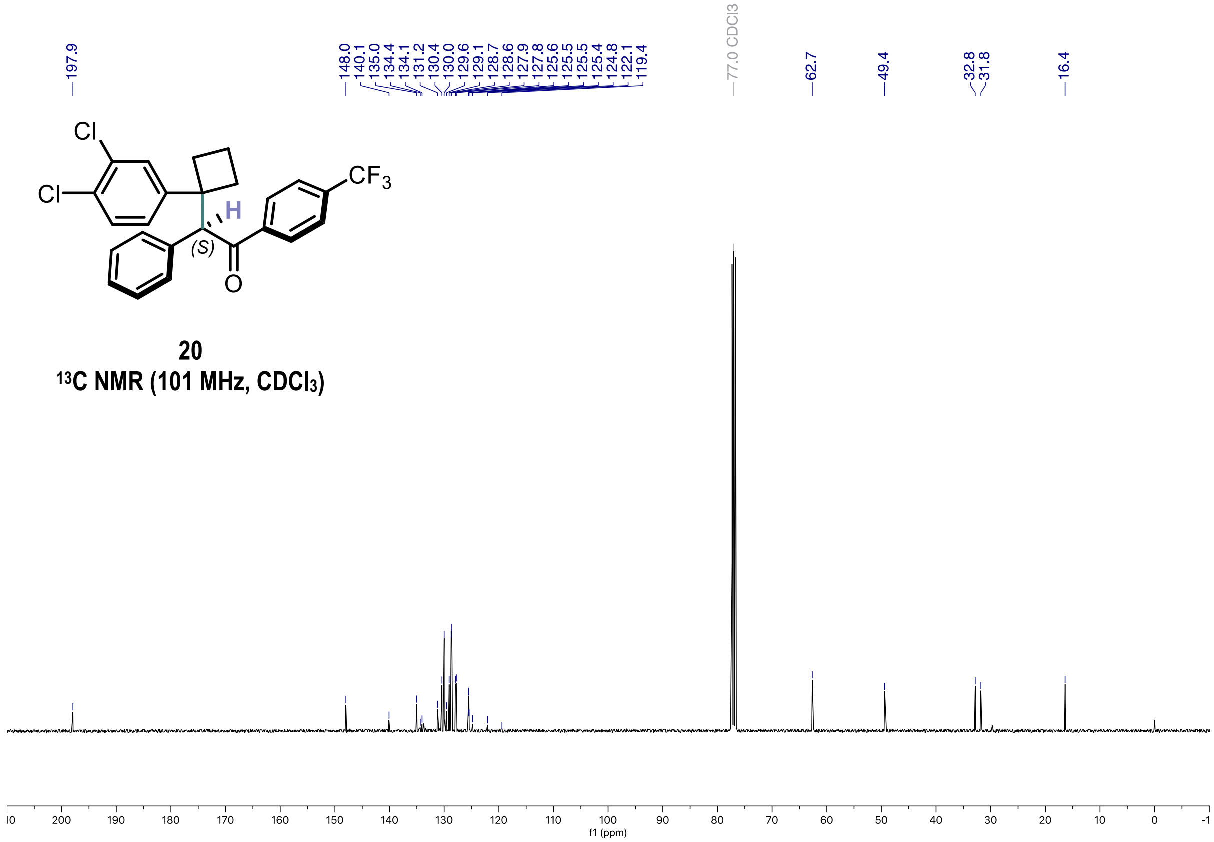
197.9 148.0 140.1 135.0 134.4 134.1 131.2 130.4 130.0 129.6 129.1 128.7 128.6 127.9 127.8 125.6 125.5 125.4 124.8 122.1 119.4 77.0 CDCl3 62.7 49.4 32.8 31.8 16.4
Cl
Cl
CF3
H
(S)
O
20
13C NMR (101 MHz, CDCl3)
f1 (ppm)

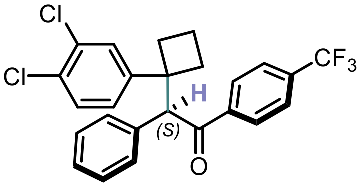

20

^{19}F NMR (376 MHz, CDCl$_3$)

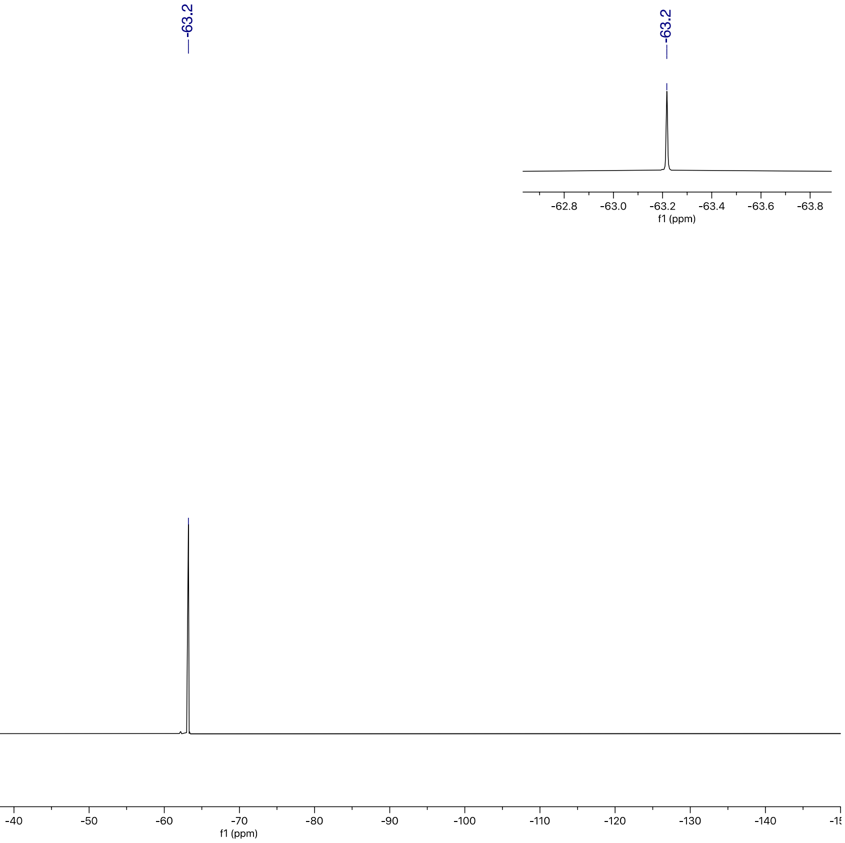

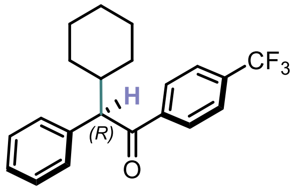

21

^{1}H NMR (600 MHz, CDCl$_3$)

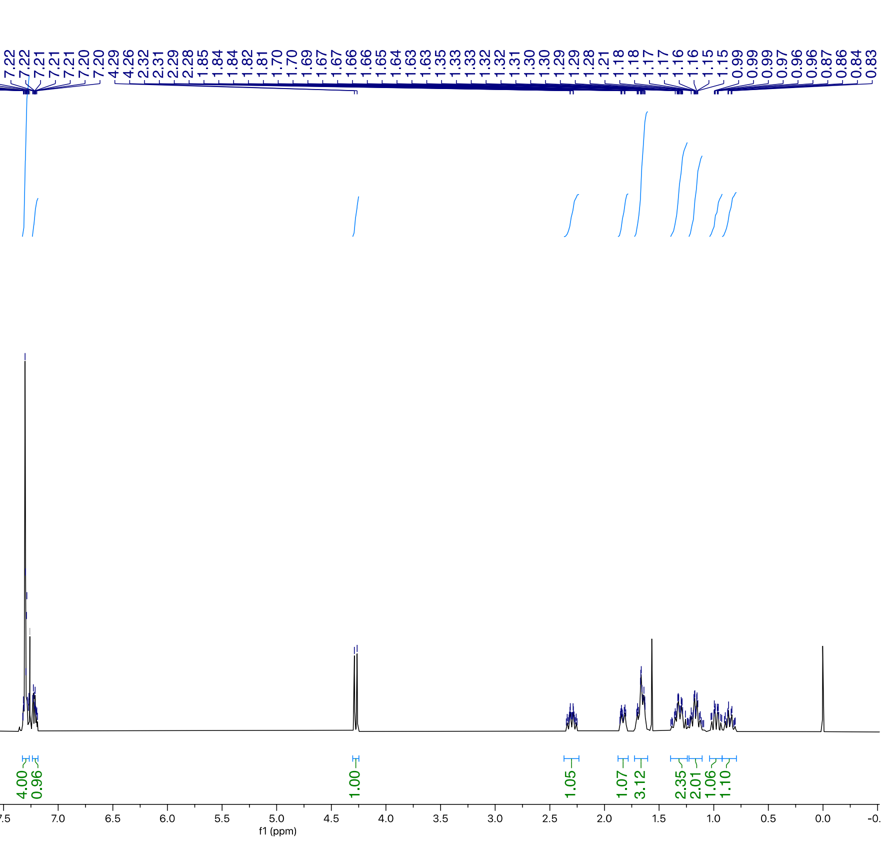

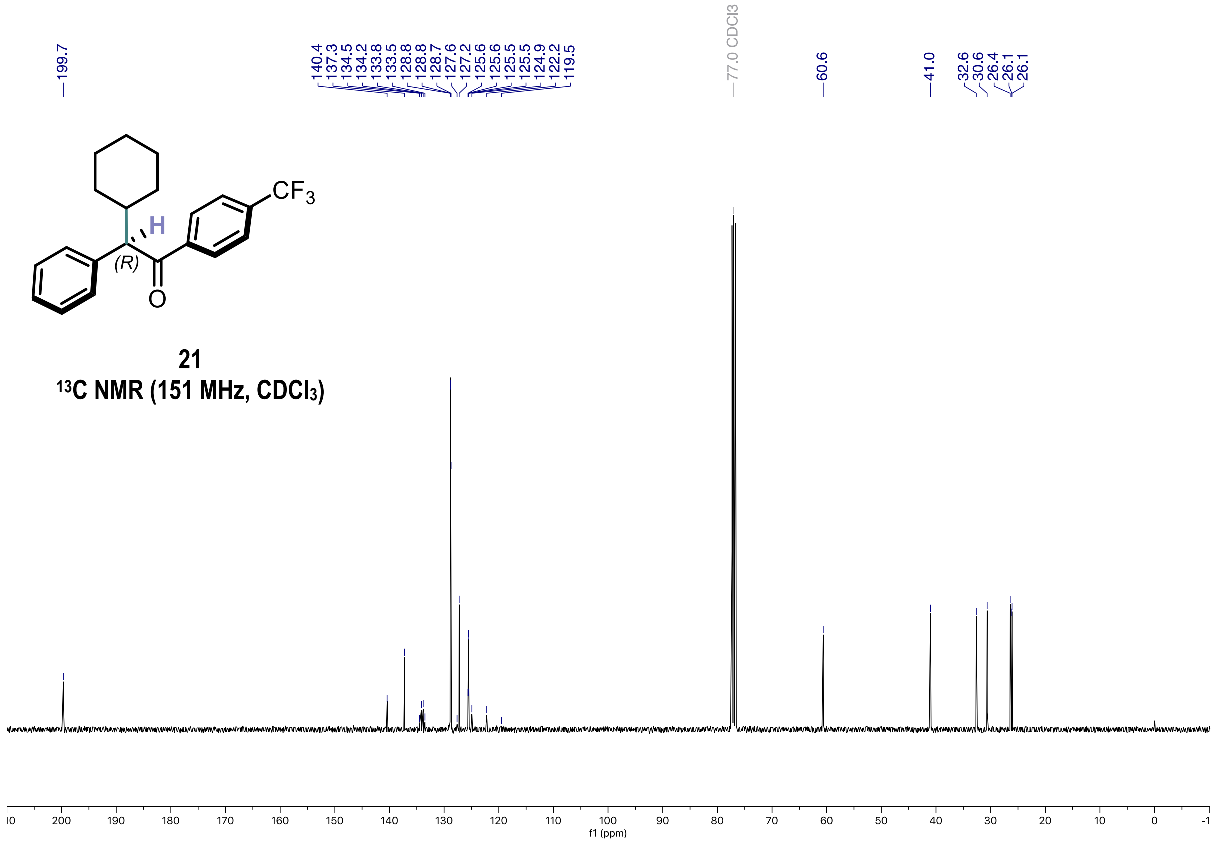

21
^{13}C NMR (151 MHz, $CDCl_3$)

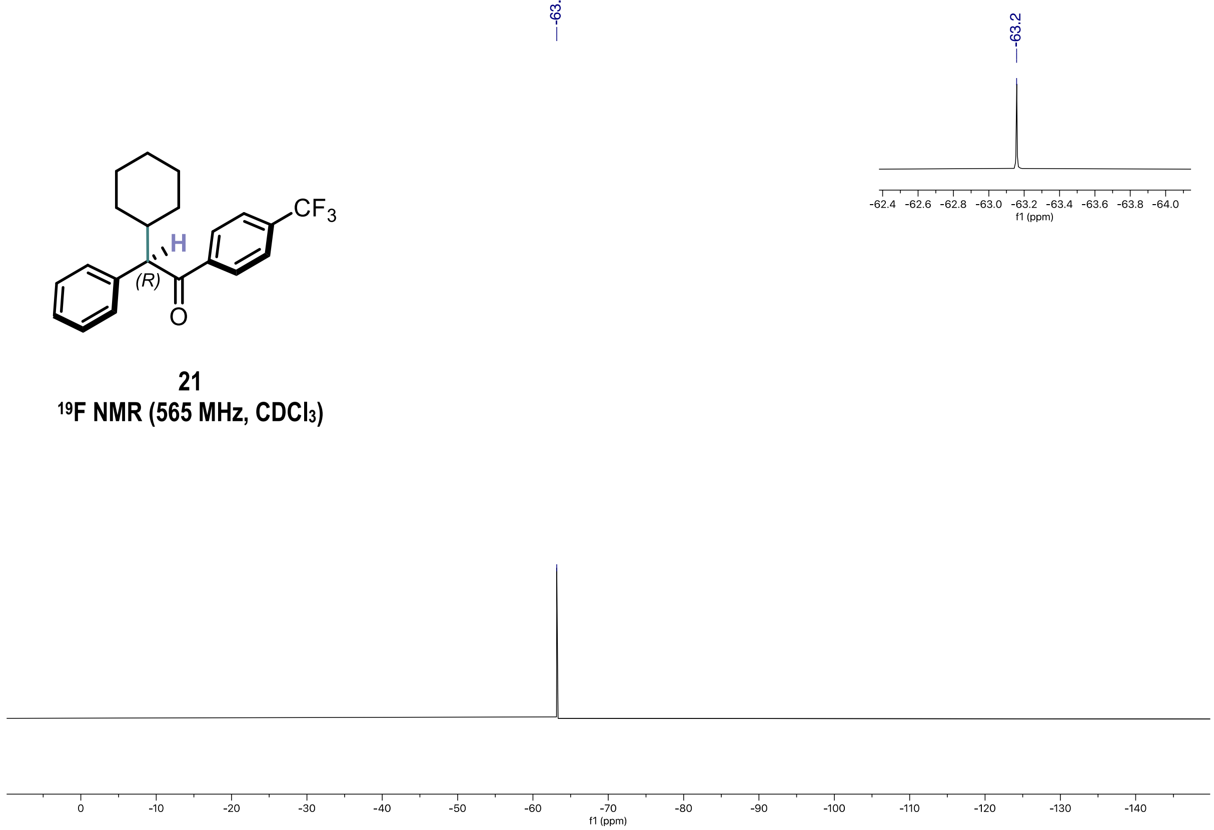

21
^{19}F NMR (565 MHz, $CDCl_3$)

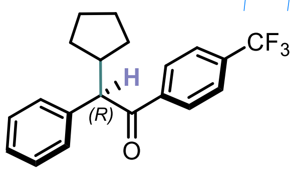

22

^{1}H NMR (400 MHz, CDCl$_3$)

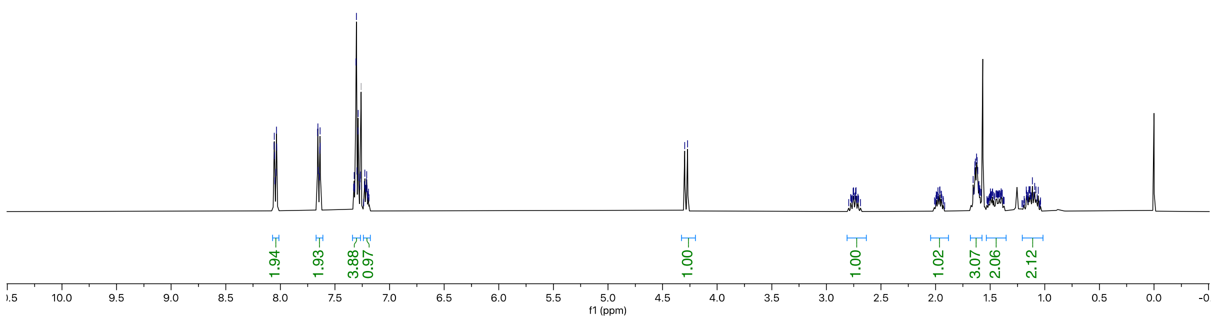

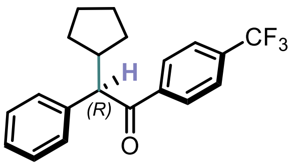

22

^{13}C NMR (151 MHz, CDCl$_3$)

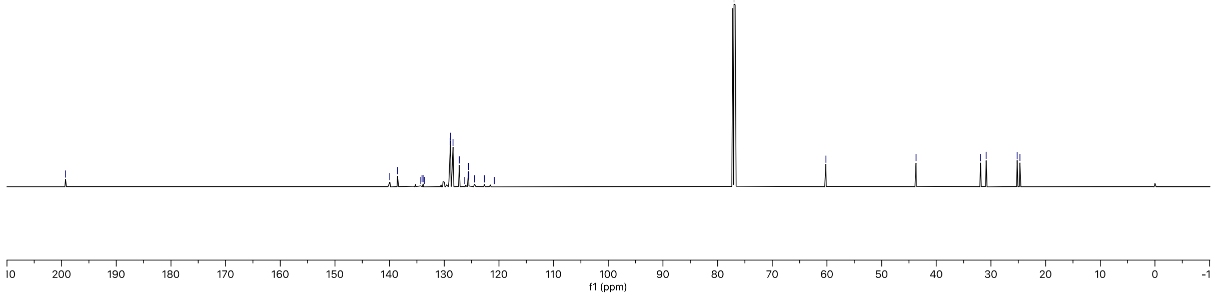

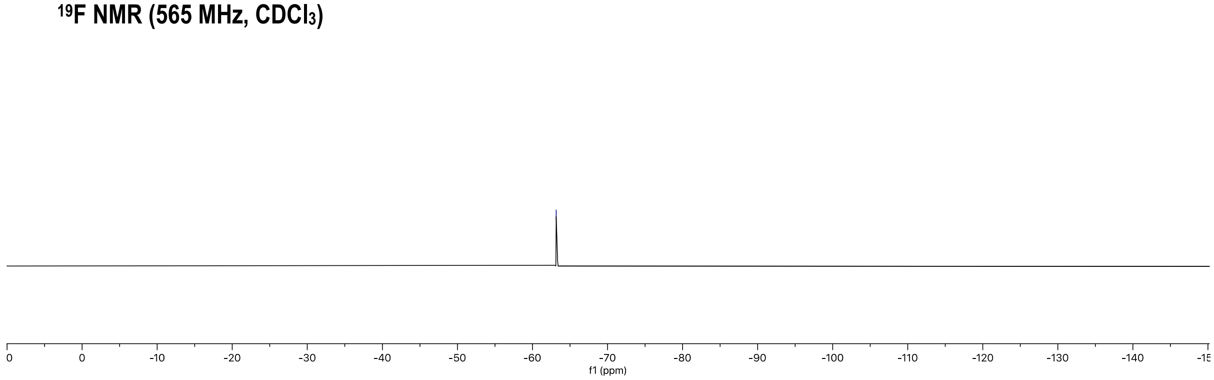

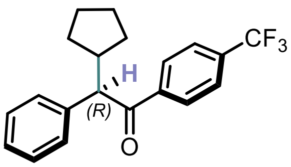

22

^{19}F NMR (565 MHz, $CDCl_3$)

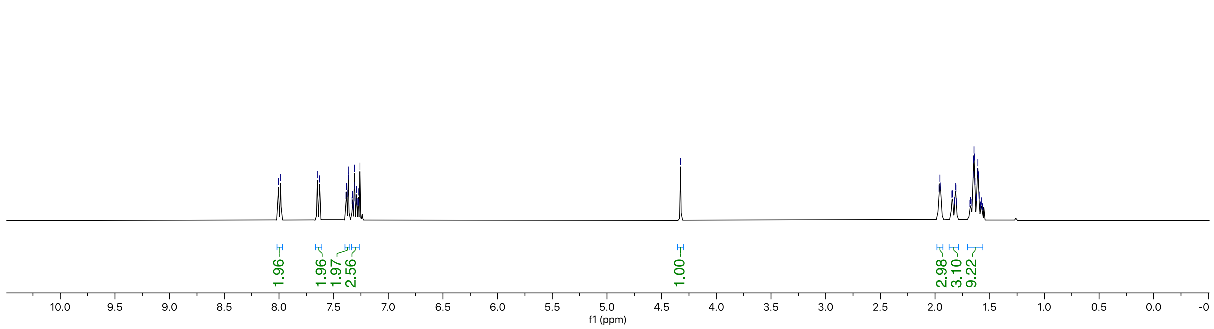

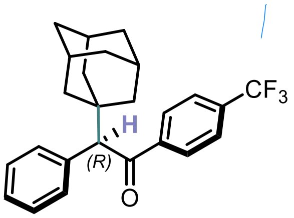

23

^{1}H NMR (400 MHz, $CDCl_3$)

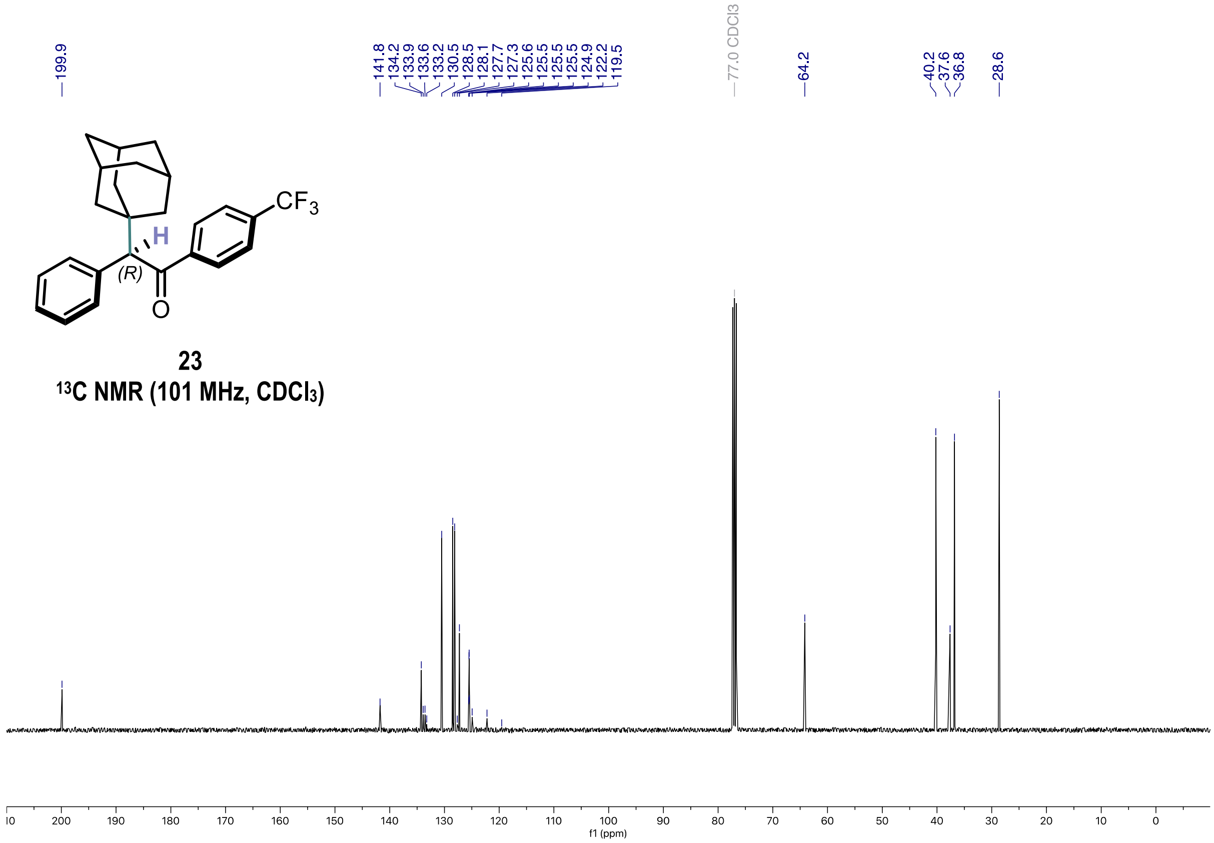

23

^{13}C NMR (101 MHz, CDCl$_3$)

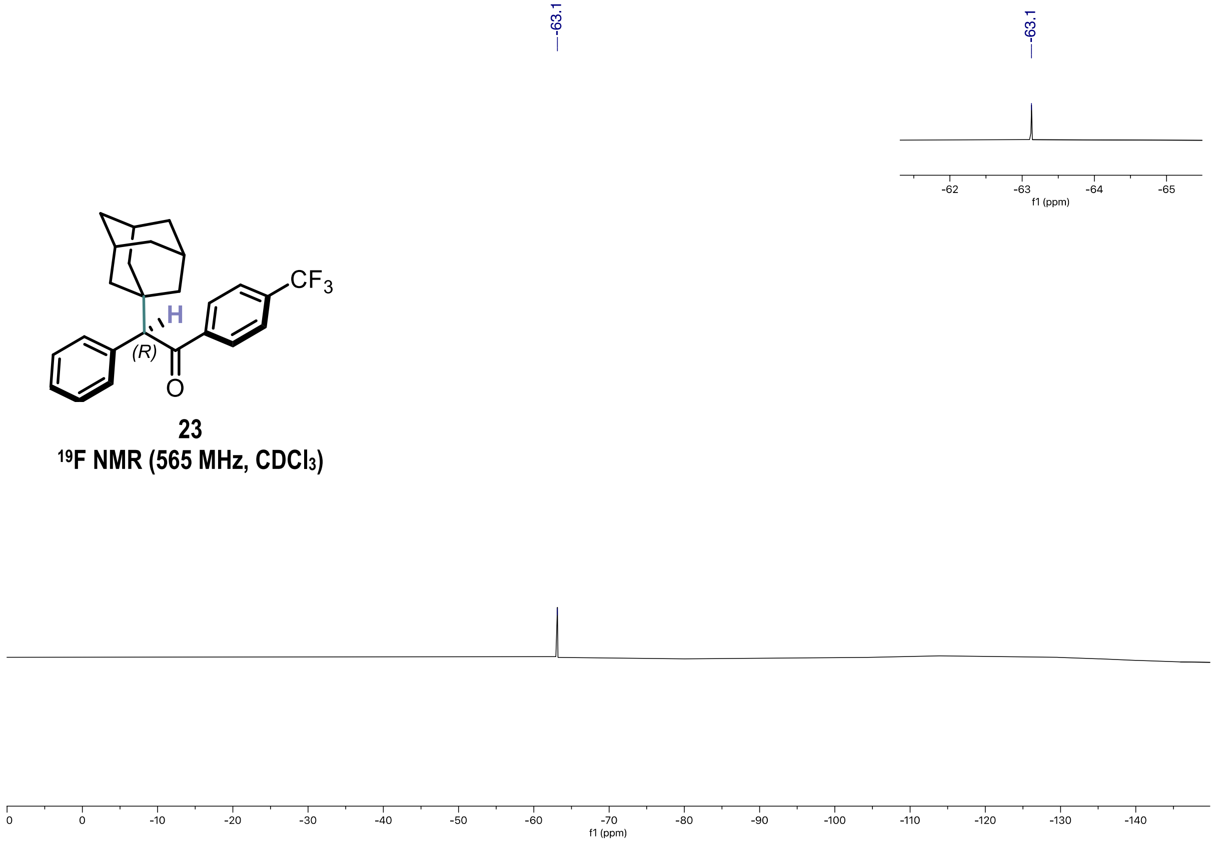

23

^{19}F NMR (565 MHz, CDCl$_3$)

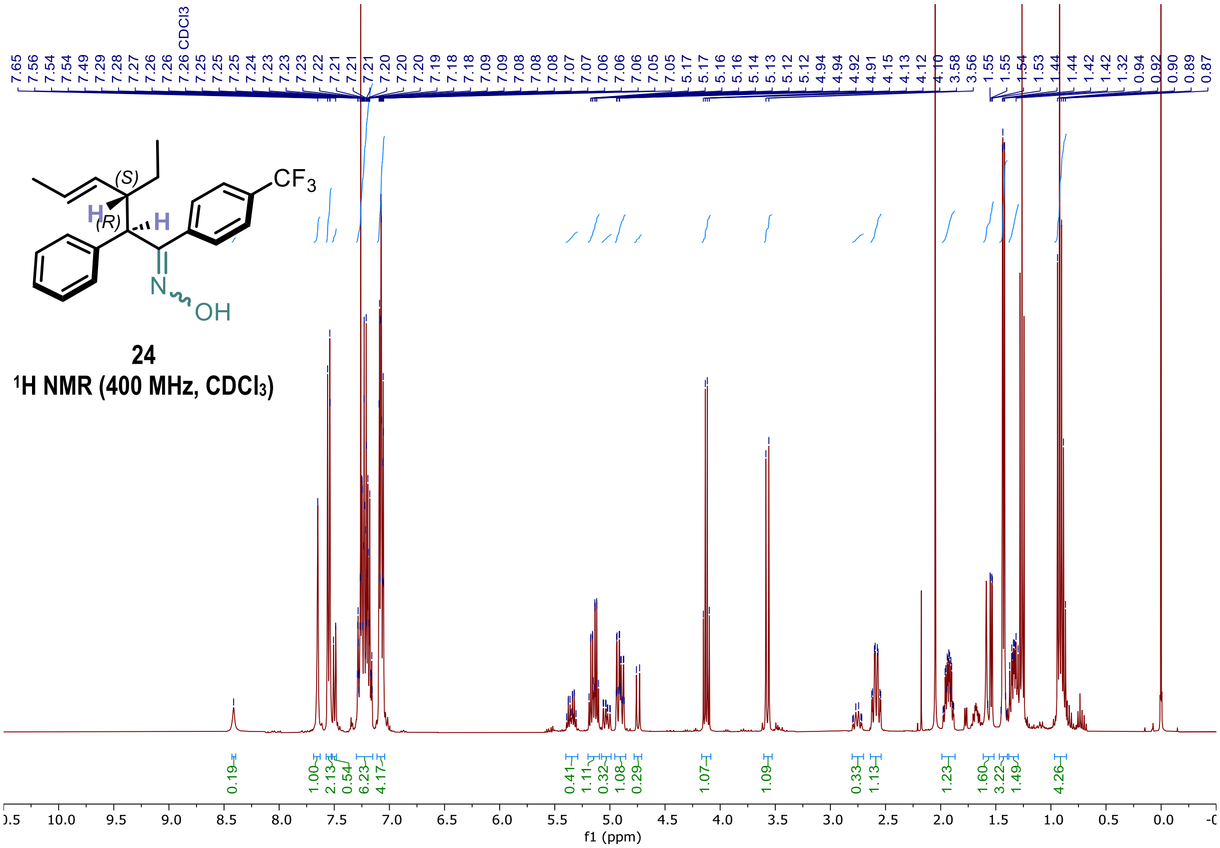
7.65 7.56 7.54 7.54 7.49 7.29 7.28 7.27 7.26 7.26 7.26 CDCl3 7.25 7.25 7.25 7.24 7.23 7.23 7.23 7.22 7.21 7.21 7.21 7.20 7.20 7.19 7.18 7.18 7.09 7.09 7.08 7.08 7.08 7.07 7.07 7.06 7.06 7.06 7.05 7.05 5.17 5.17 5.16 5.16 5.14 5.13 5.12 5.12 4.94 4.94 4.92 4.91 4.15 4.13 4.12 4.10 3.58 3.56 1.55 1.55 1.54 1.53 1.44 1.44 1.42 1.42 1.32 0.94 0.92 0.90 0.89 0.87
(S)
CF3
H (R)
H
N
OH
24
¹H NMR (400 MHz, CDCl3)
0.19 1.00 2.13 0.54 6.23 4.17 0.41 1.11 0.32 1.08 0.29 1.07 1.09 0.33 1.13 1.23 1.60 3.22 1.49 4.26
f1 (ppm)

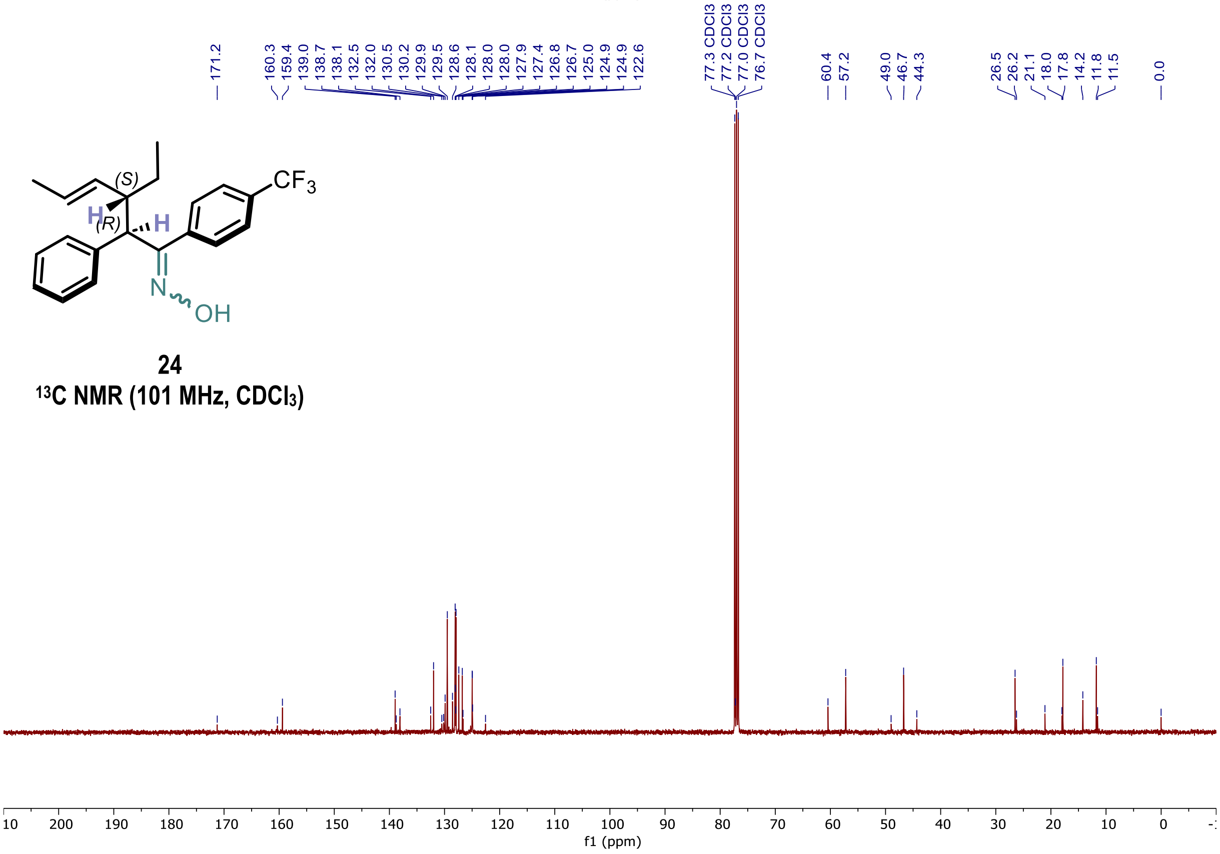
171.2 160.3 159.4 139.0 138.7 138.1 132.5 132.0 130.5 130.2 129.9 129.5 128.6 128.1 128.0 127.9 127.4 126.8 126.7 125.0 124.9 124.9 122.6
77.3 CDCl3 77.2 CDCl3 77.0 CDCl3 76.7 CDCl3
60.4 57.2 49.0 46.7 44.3 26.5 26.2 21.1 18.0 17.8 14.2 11.8 11.5 0.0
(S)
CF3
H (R)
H
N
OH
24
¹³C NMR (101 MHz, CDCl3)
f1 (ppm)

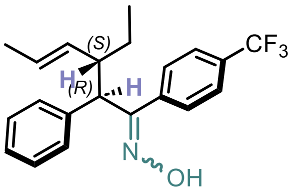

24

^{19}F NMR (376 MHz, CDCl$_3$)

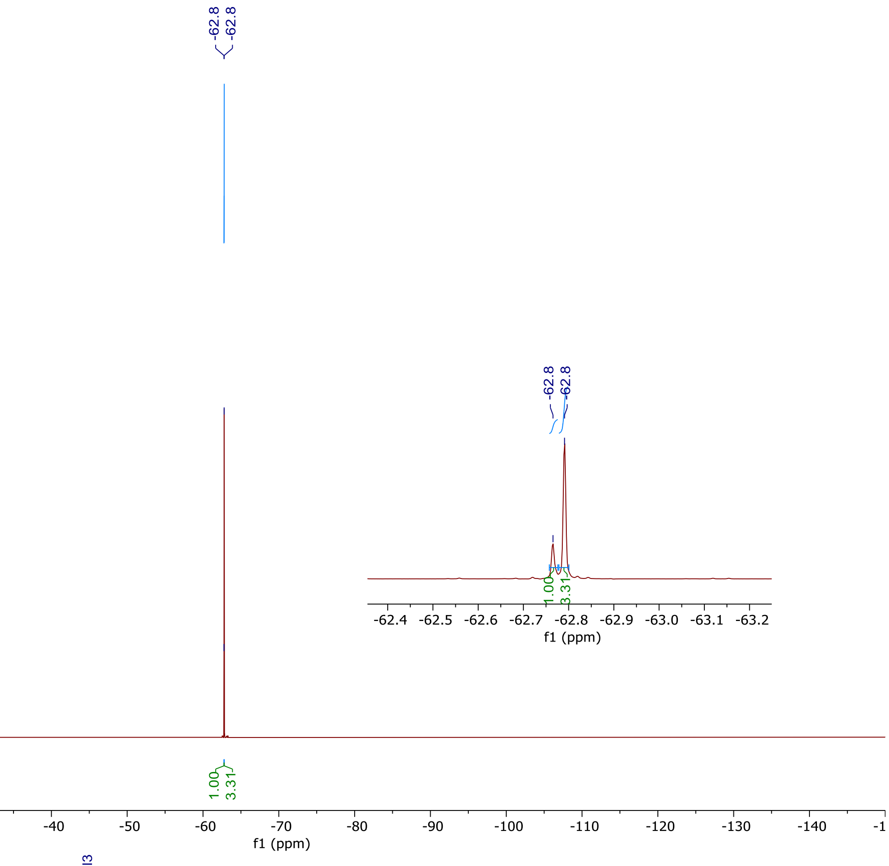

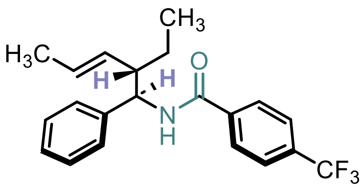

25

^{1}H NMR (600 MHz, CDCl$_3$)

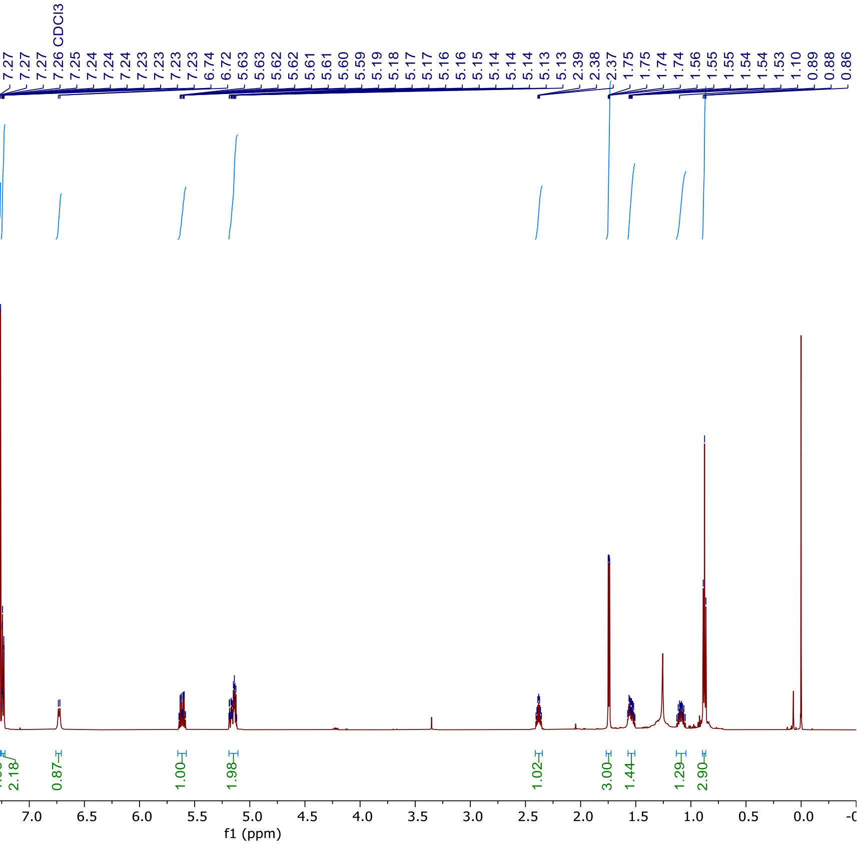

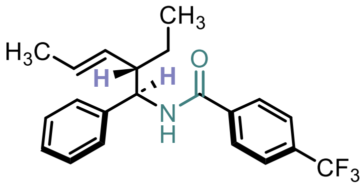

25

^{13}C NMR (151 MHz, CDCl$_3$)

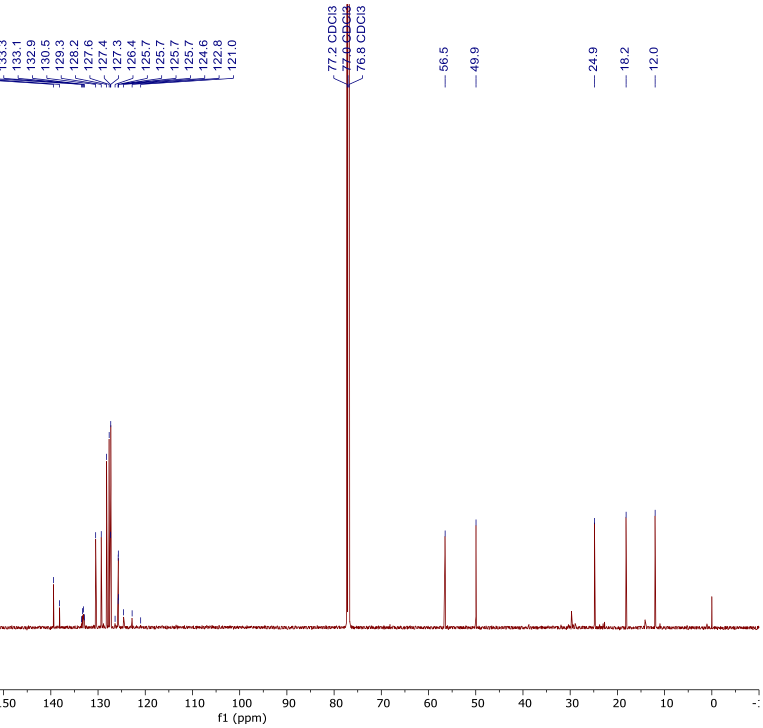

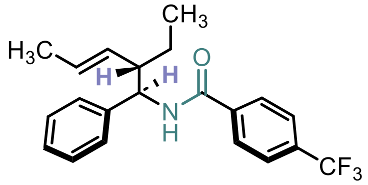

25

^{19}F NMR (565 MHz, CDCl$_3$)

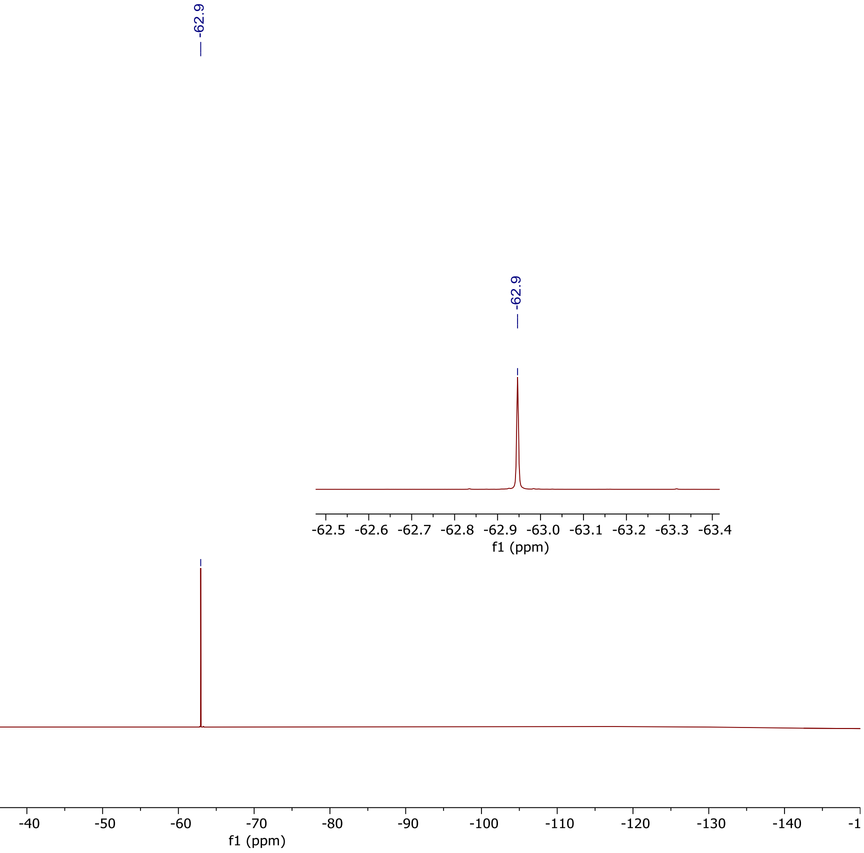

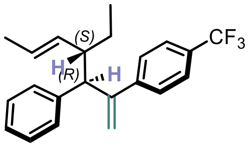

26

^{1}H NMR (400 MHz, $CDCl_3$)

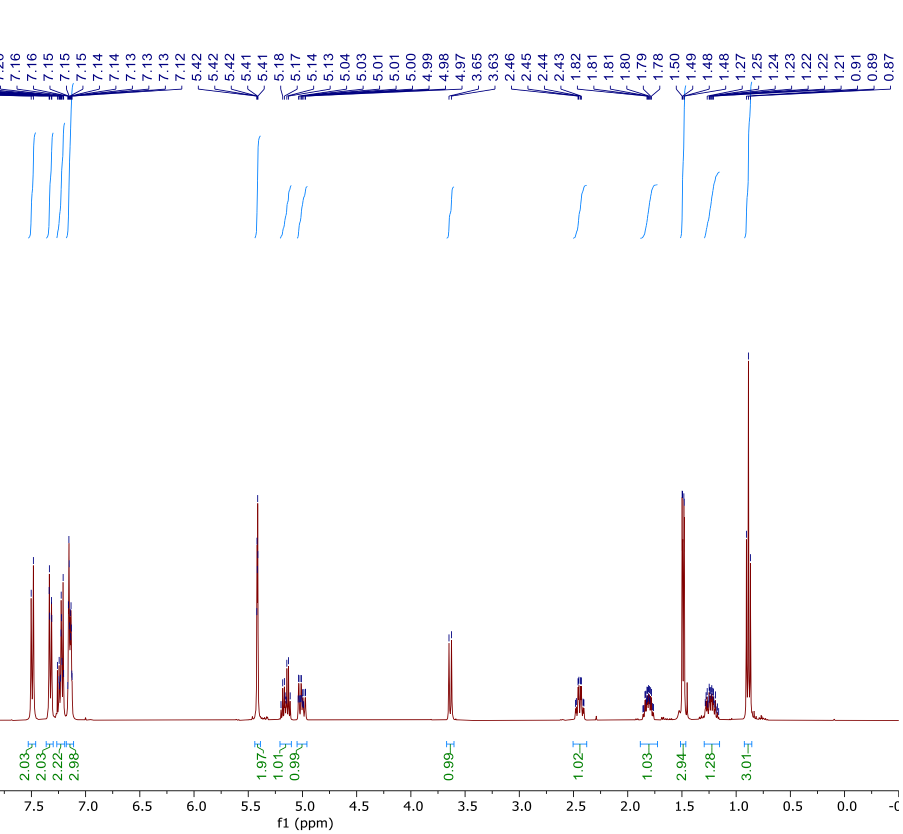

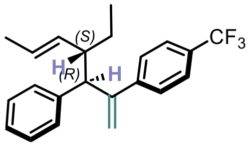

26

^{13}C NMR (101 MHz, $CDCl_3$)

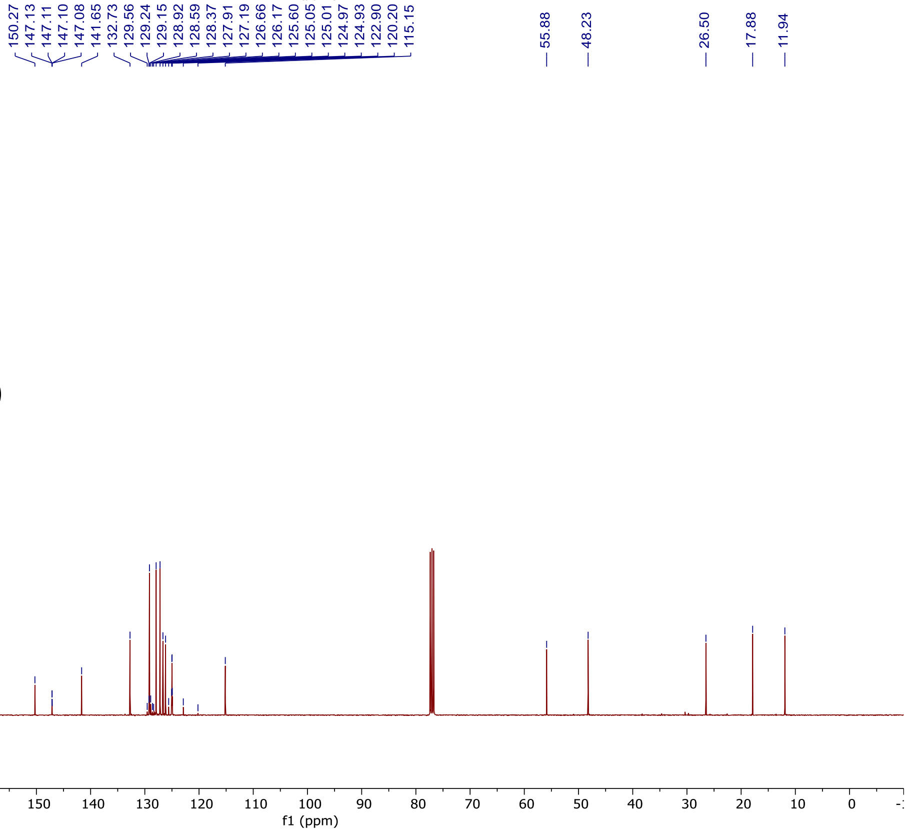

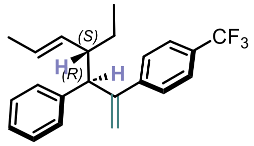

26

^{19}F NMR (376 MHz, CDCl$_3$)

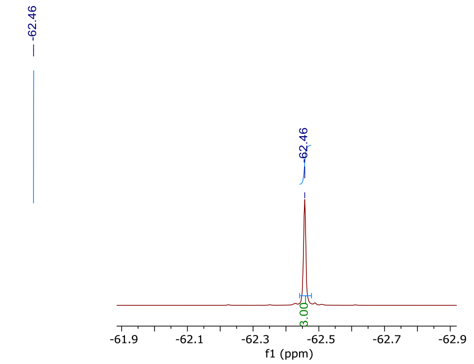

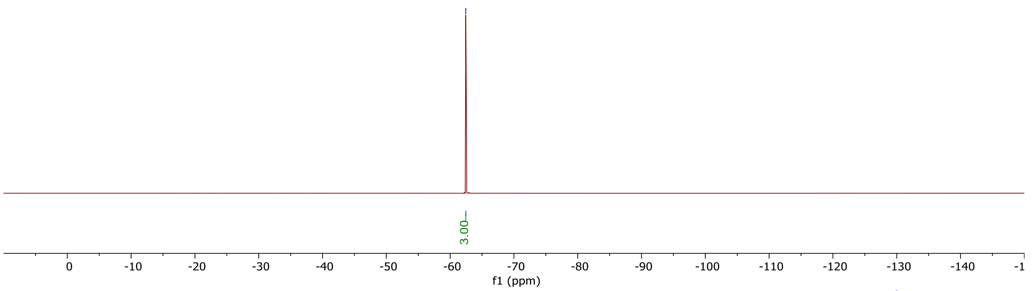

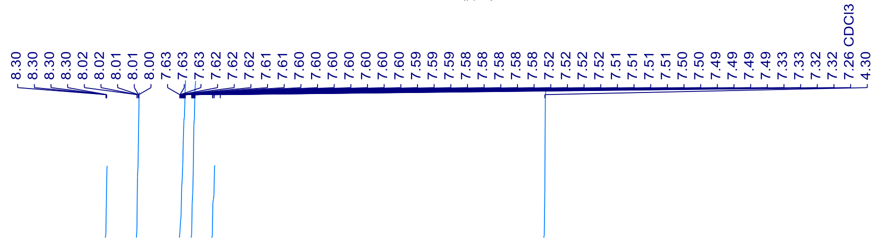

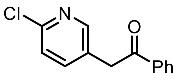

S27

^{1}H NMR (600 MHz, CDCl$_3$)

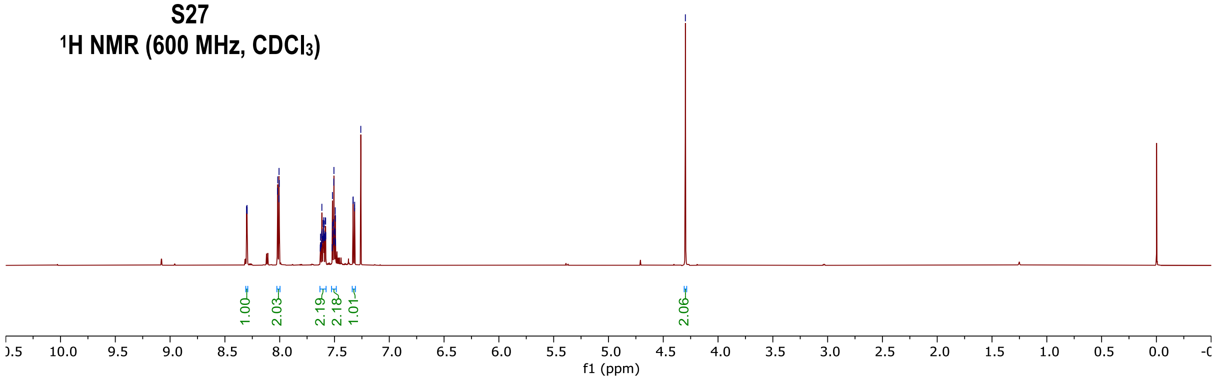

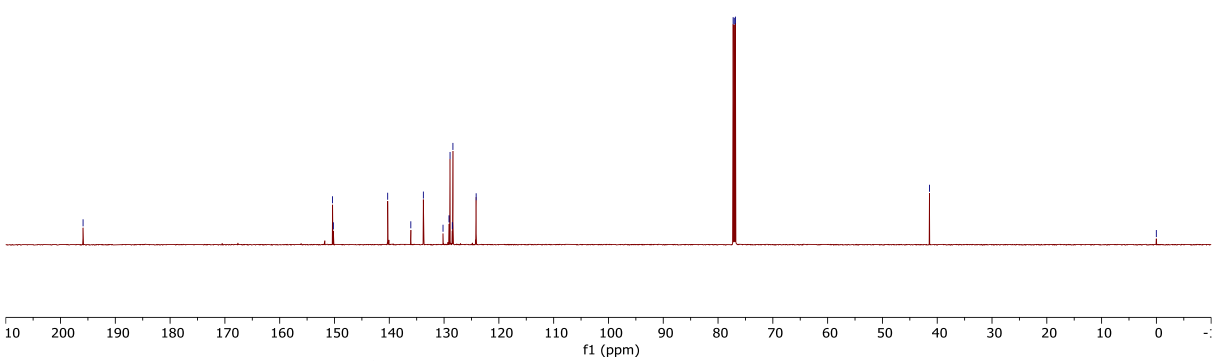

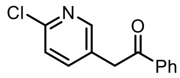

S27

^{13}C NMR (151 MHz, $CDCl_3$)

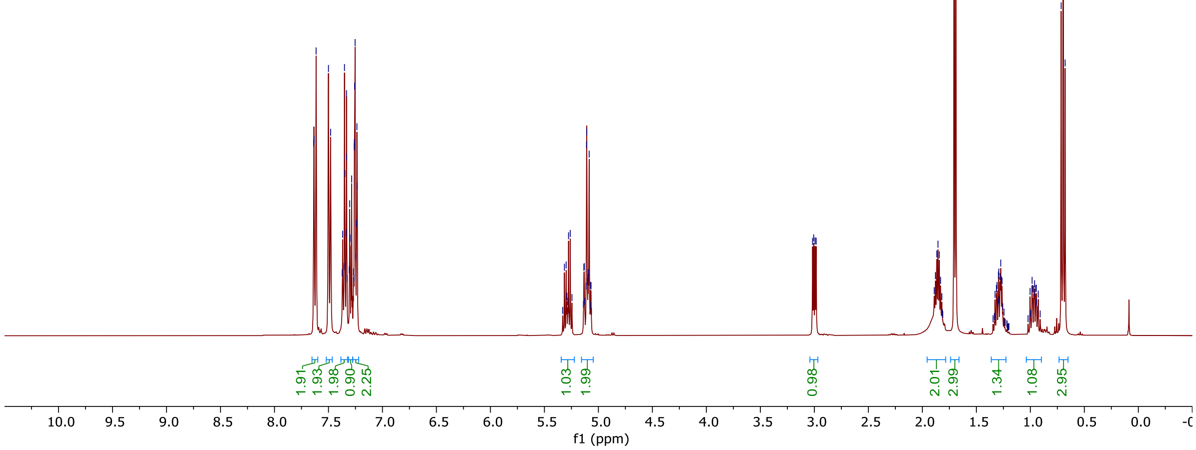

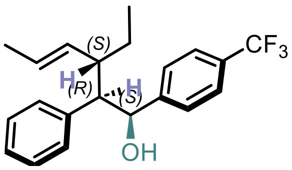

27

^{1}H NMR (400 MHz, $CDCl_3$)

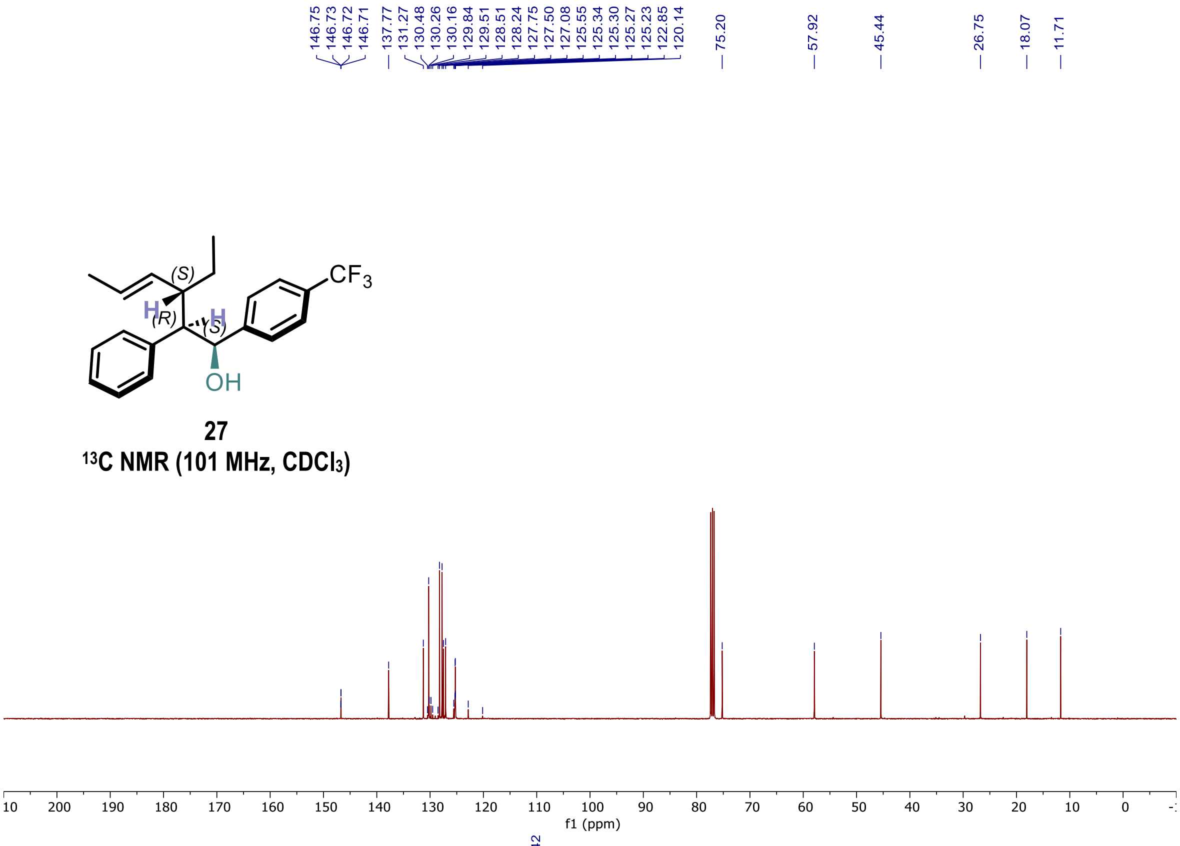

27

^{13}C NMR (101 MHz, CDCl$_3$)

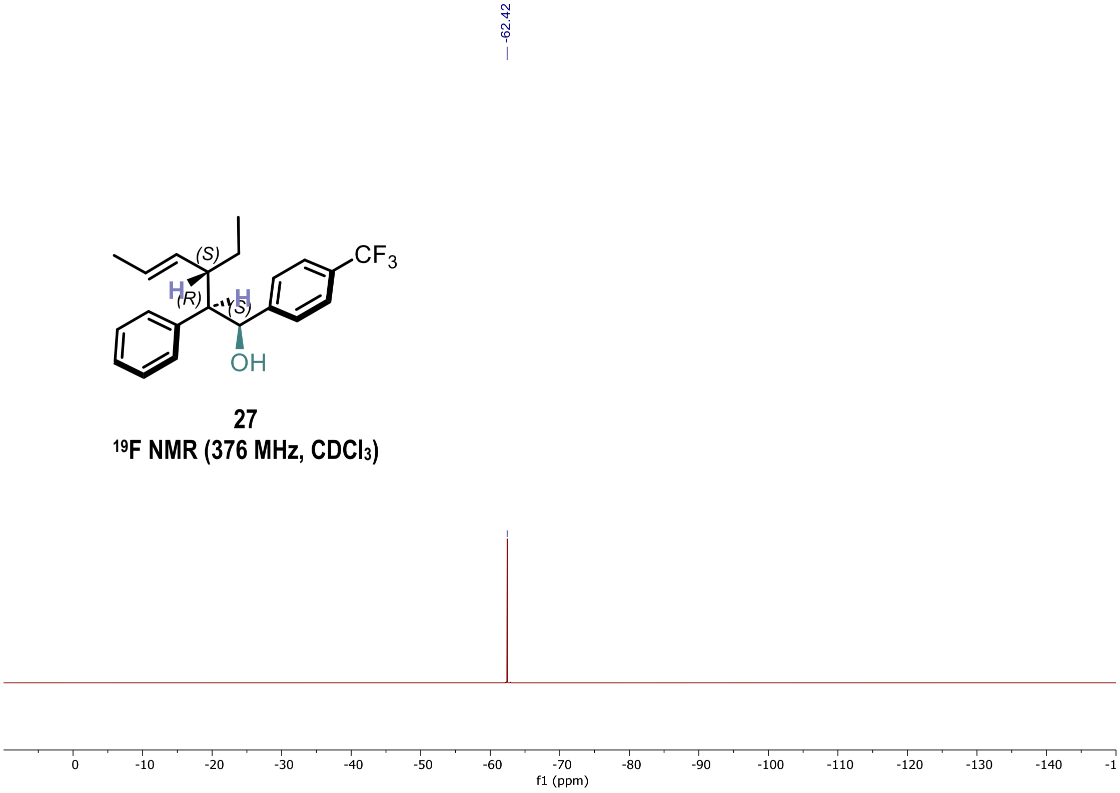

27

^{19}F NMR (376 MHz, CDCl$_3$)

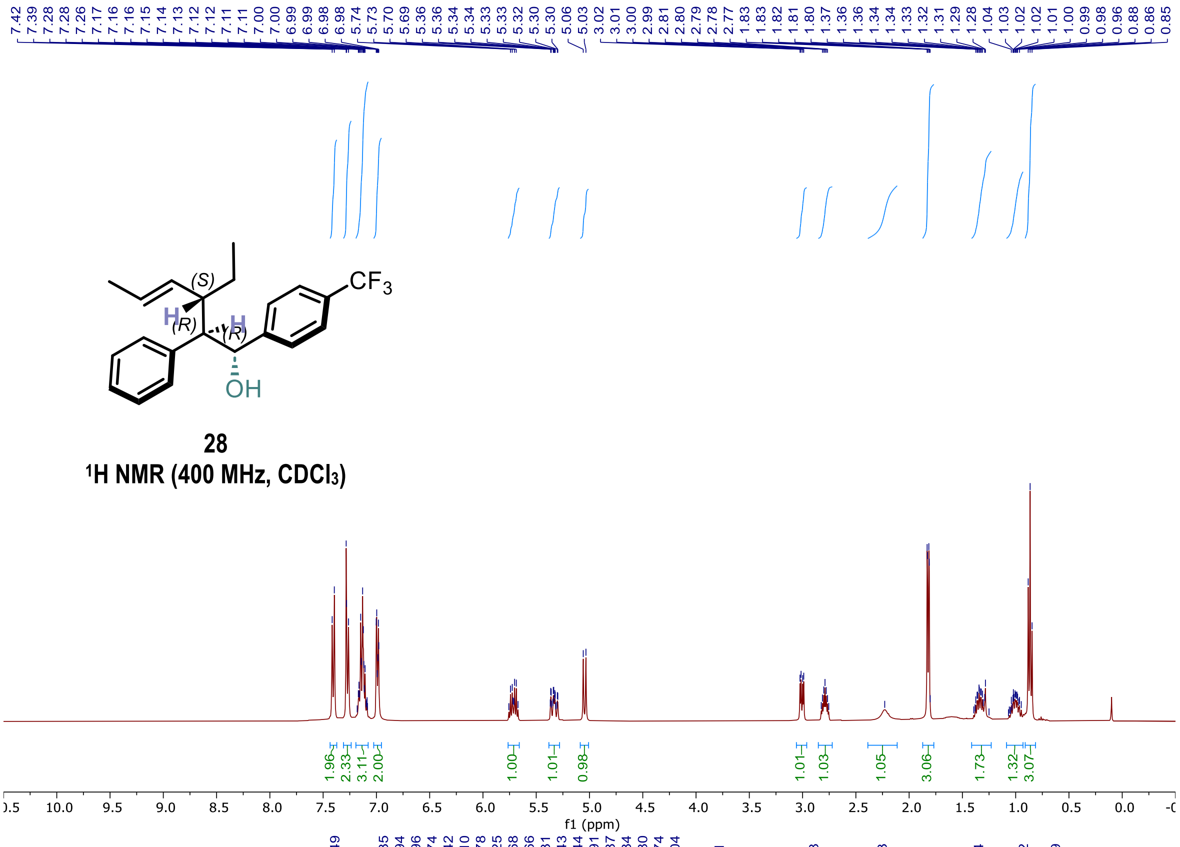

28

^{1}H NMR (400 MHz, $CDCl_3$)

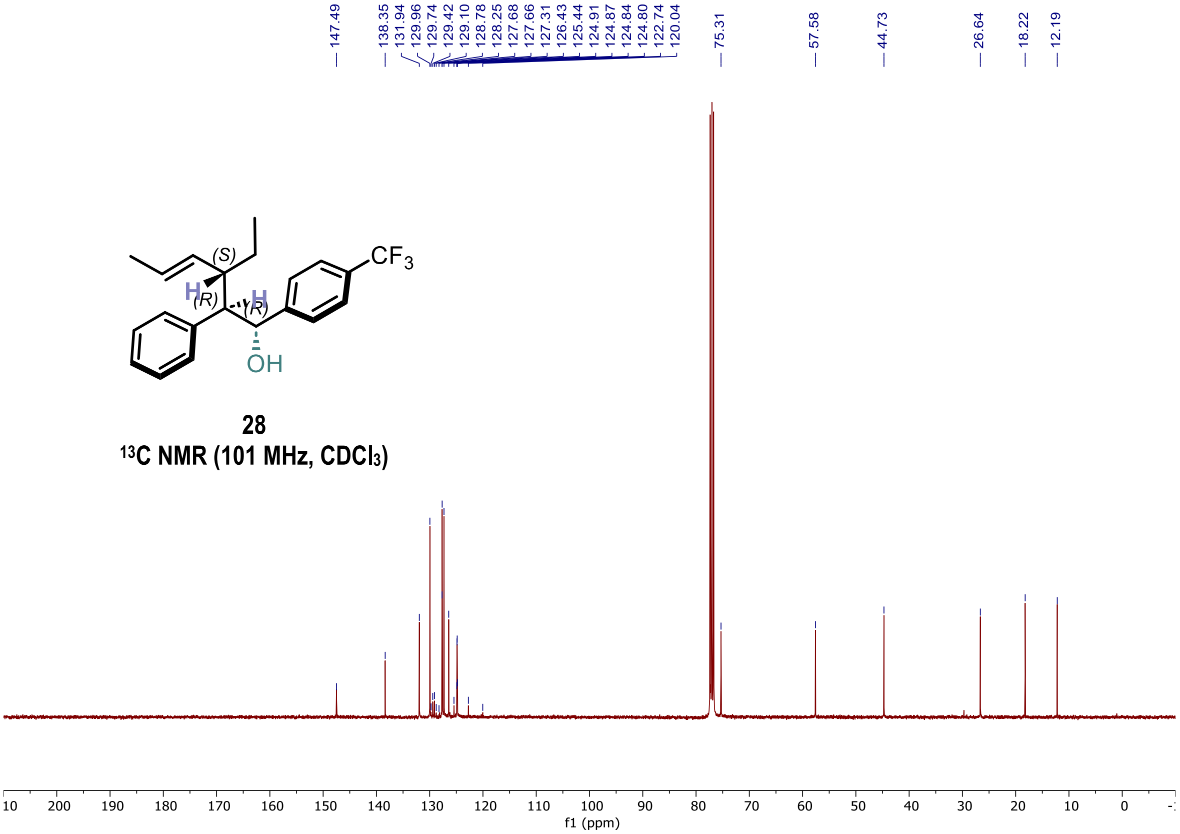

28

^{13}C NMR (101 MHz, $CDCl_3$)

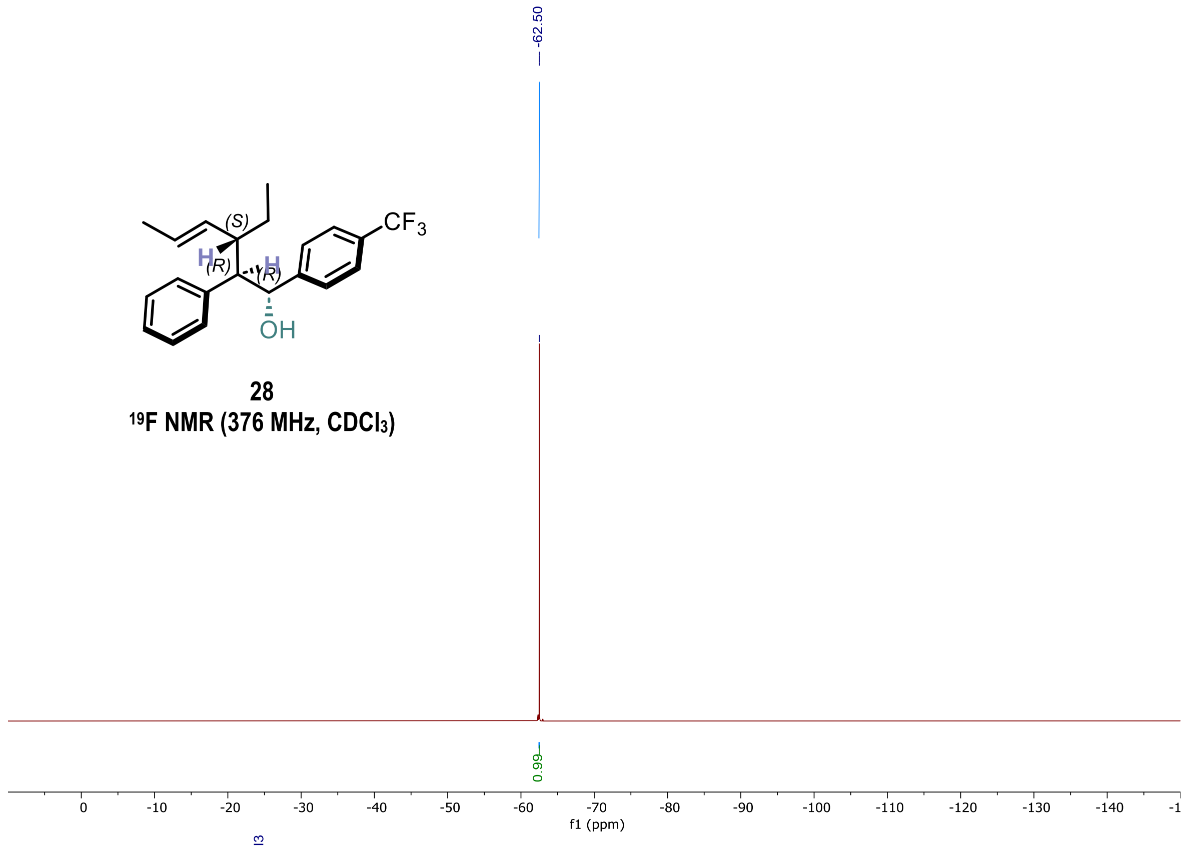

-62.50
CF3
OH
28
19F NMR (376 MHz, CDCl3)
0.99
0 -10 -20 -30 -40 -50 -60 -70 -80 -90 -100 -110 -120 -130 -140
f1 (ppm)

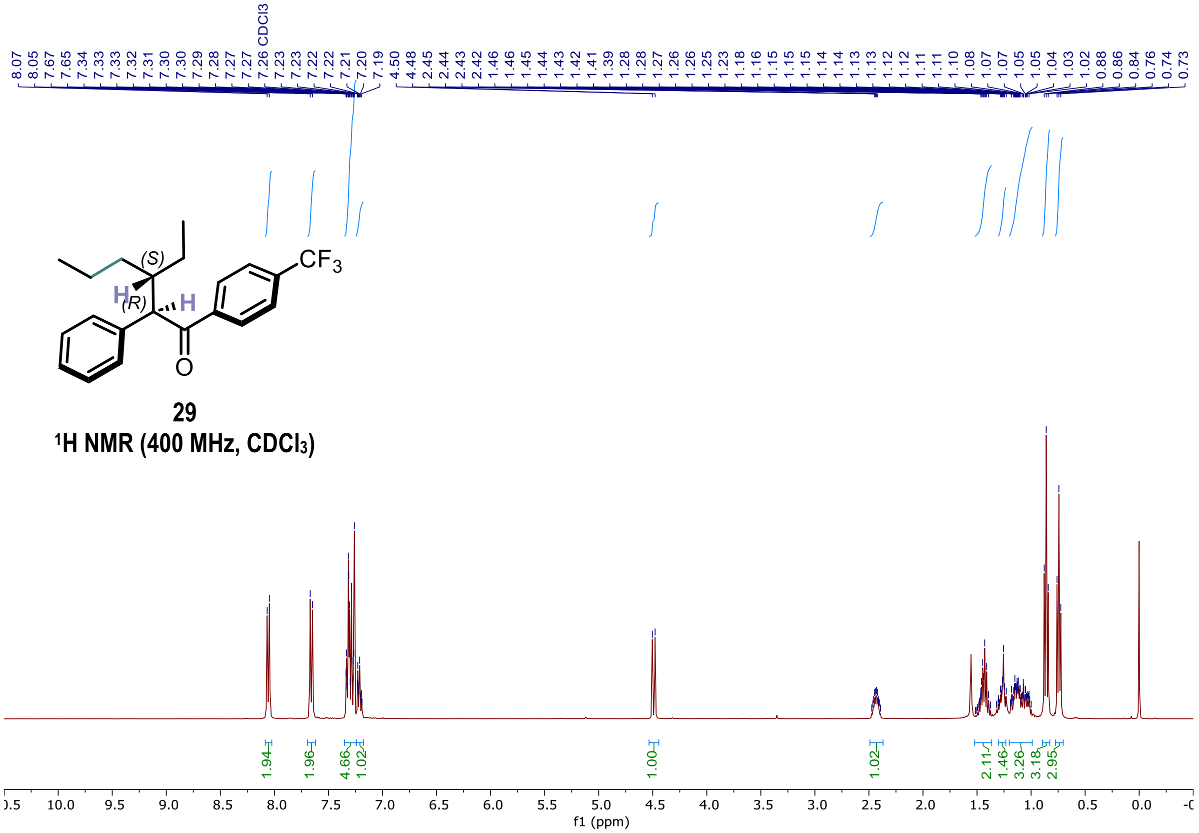

8.07 8.05 7.67 7.65 7.34 7.33 7.32 7.31 7.30 7.29 7.27 7.27 7.26 CDCl3 7.23 7.23 7.22 7.22 7.21 7.20 7.19 4.50 4.48 2.45 2.44 2.43 2.42 1.46 1.46 1.45 1.44 1.43 1.42 1.41 1.39 1.28 1.27 1.26 1.25 1.23 1.18 1.16 1.15 1.15 1.14 1.14 1.13 1.13 1.12 1.12 1.11 1.11 1.10 1.08 1.07 1.07 1.05 1.05 1.04 1.03 1.02 0.88 0.86 0.84 0.76 0.74 0.73
CF3
O
29
1H NMR (400 MHz, CDCl3)
1.94 1.96 4.66 1.02 1.00 1.02 2.11 1.46 3.26 3.18 2.95
10.0 9.5 9.0 8.5 8.0 7.5 7.0 6.5 6.0 5.5 5.0 4.5 4.0 3.5 3.0 2.5 2.0 1.5 1.0 0.5 0.0
f1 (ppm)

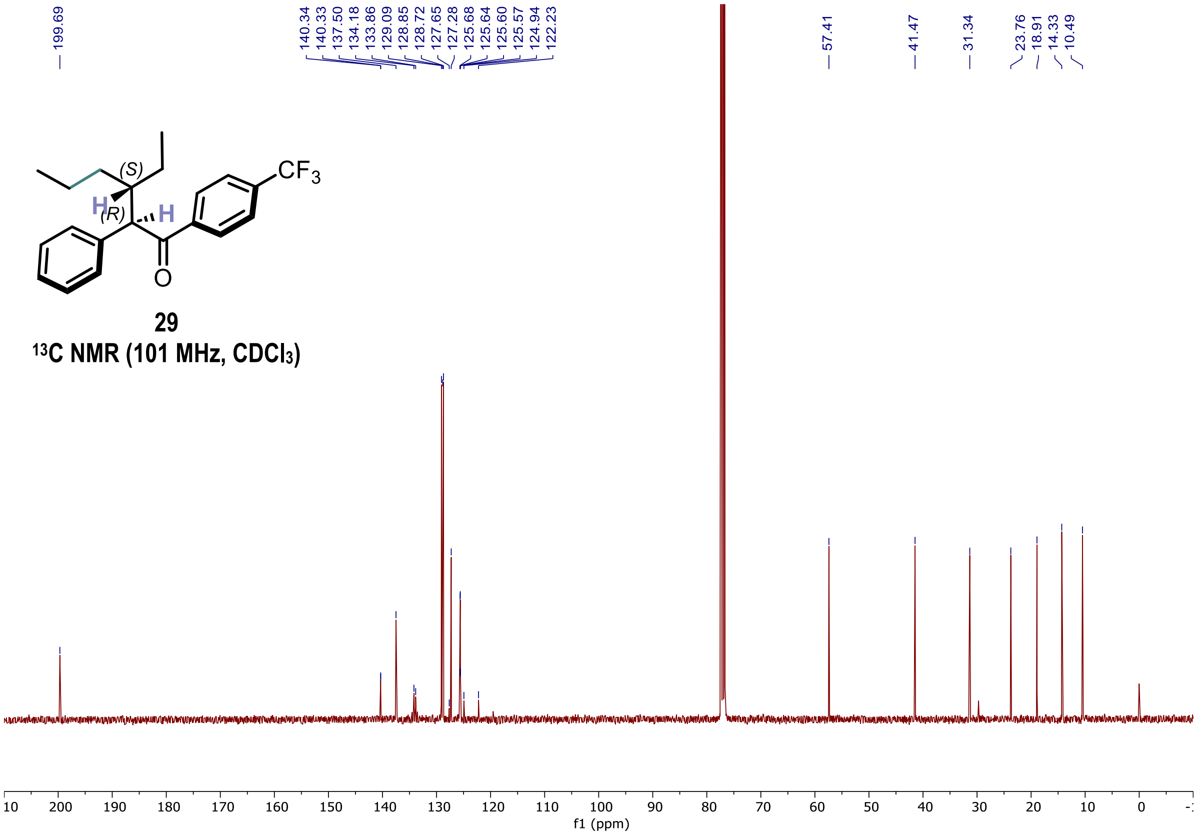
199.69
140.34
140.33
137.50
134.18
133.86
129.09
128.85
128.72
127.65
127.28
125.68
125.64
125.60
125.57
124.94
122.23
57.41
41.47
31.34
23.76
18.91
14.33
10.49
(S)
(R)
H
H
CF3
O
29
13C NMR (101 MHz, CDCl3)
f1 (ppm)

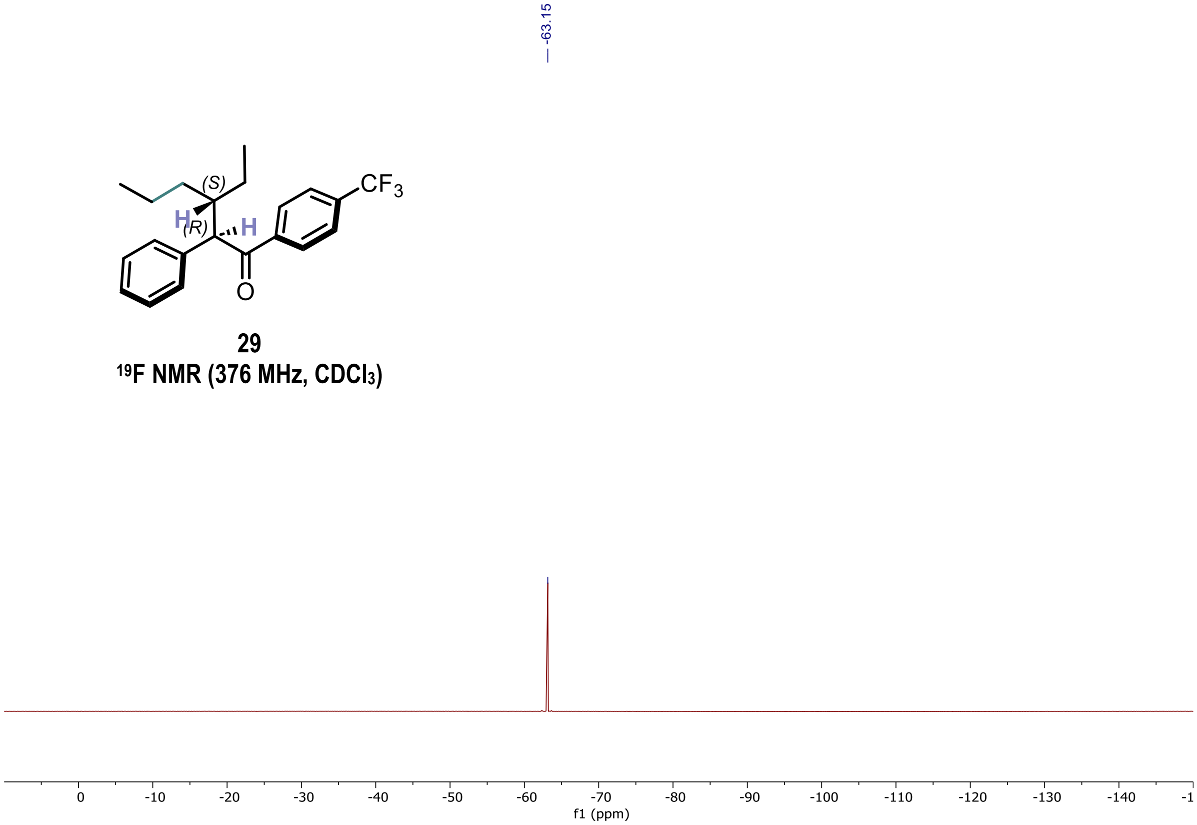
-63.15
(S)
(R)
H
H
CF3
O
29
19F NMR (376 MHz, CDCl3)
f1 (ppm)

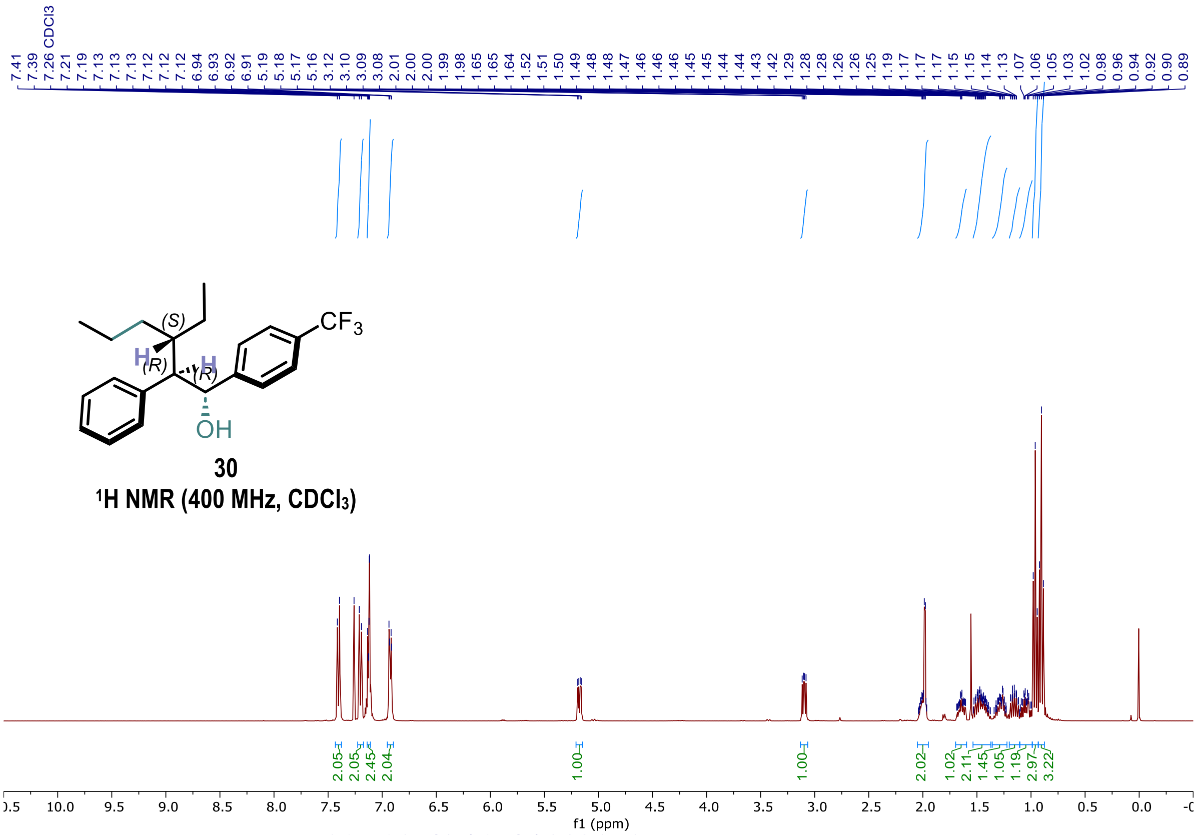

30

^{1}H NMR (400 MHz, $CDCl_3$)

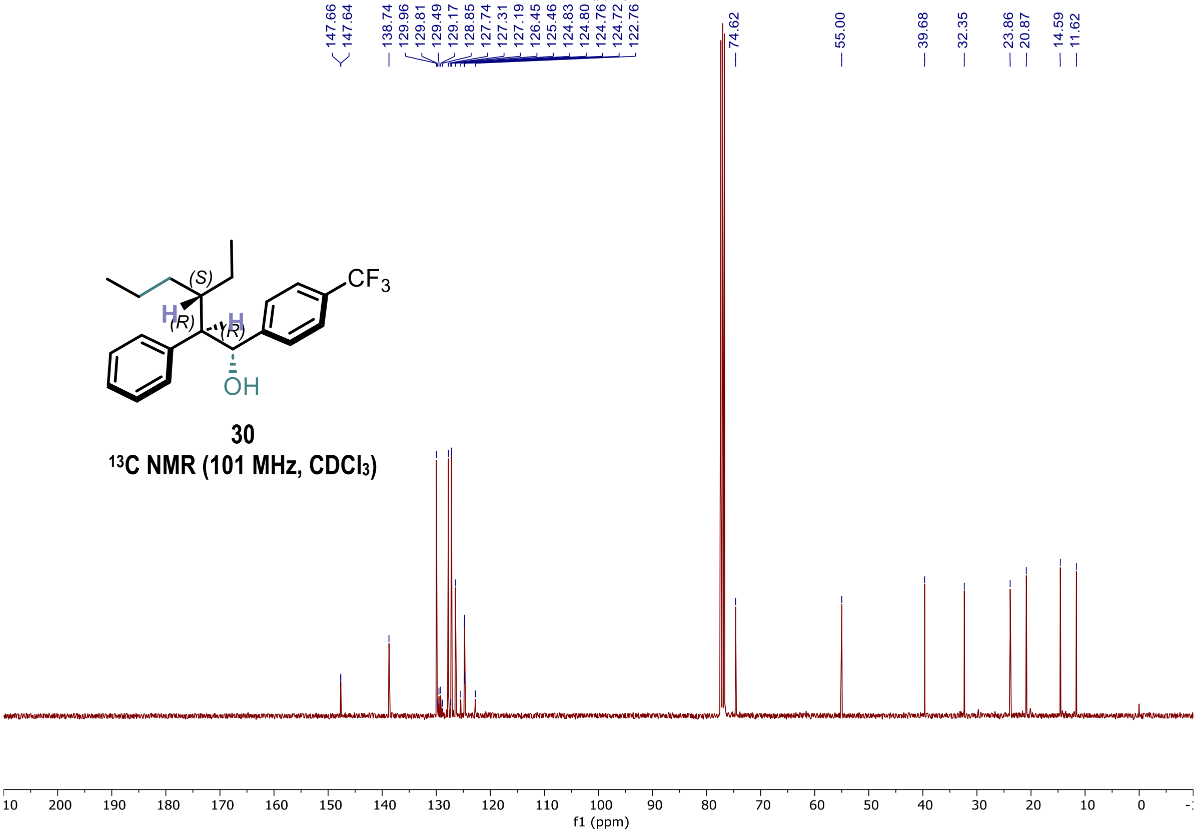

30

^{13}C NMR (101 MHz, $CDCl_3$)

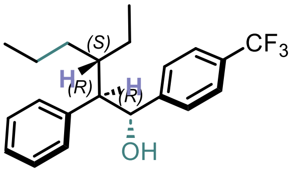

30

^{19}F NMR (376 MHz, $CDCl_3$)

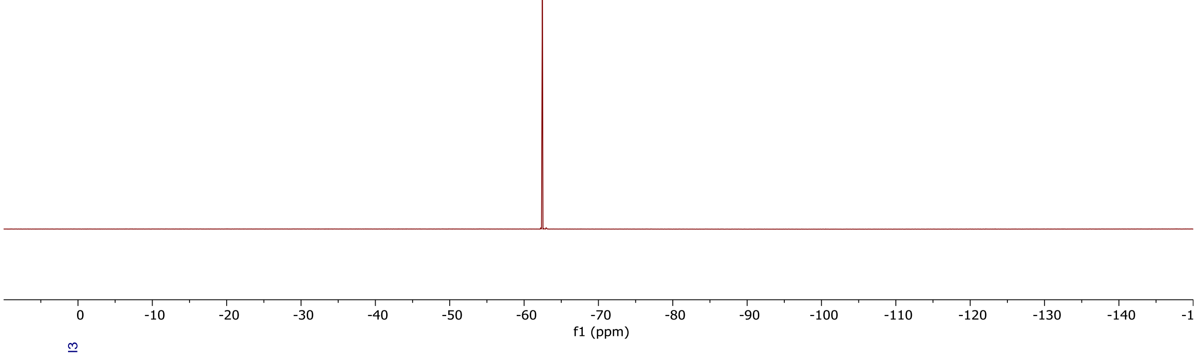

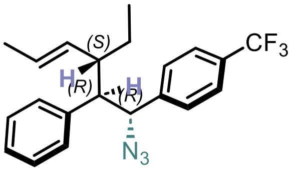

31

^{1}H NMR (400 MHz, $CDCl_3$)

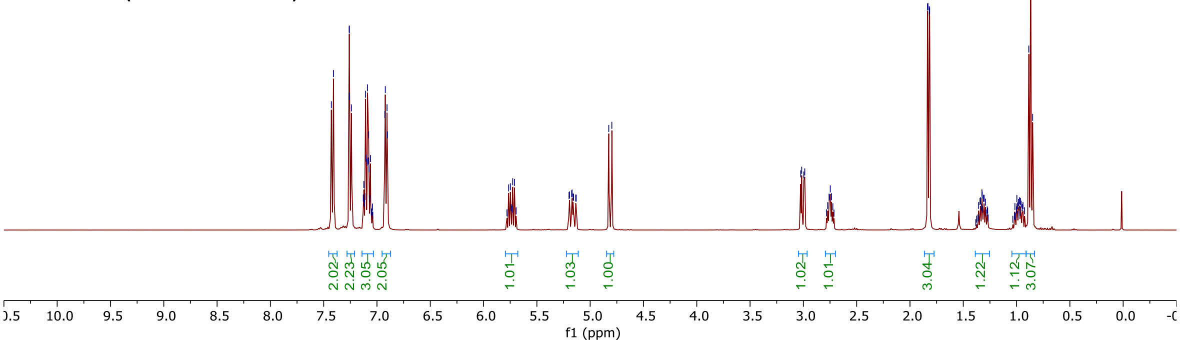

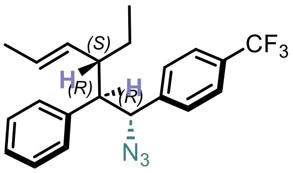

31

^{13}C NMR (101 MHz, CDCl$_3$)

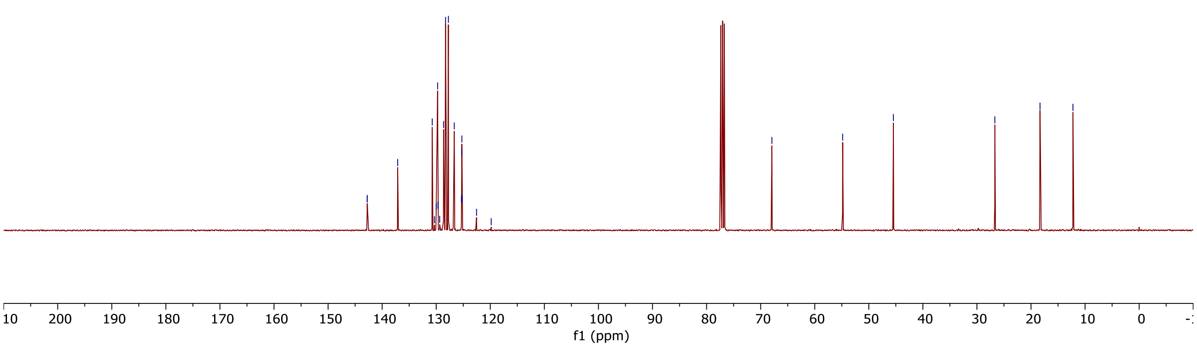

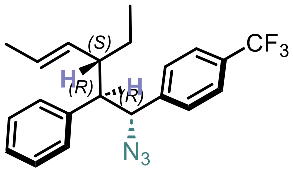

31

^{19}F NMR (376 MHz, CDCl$_3$)

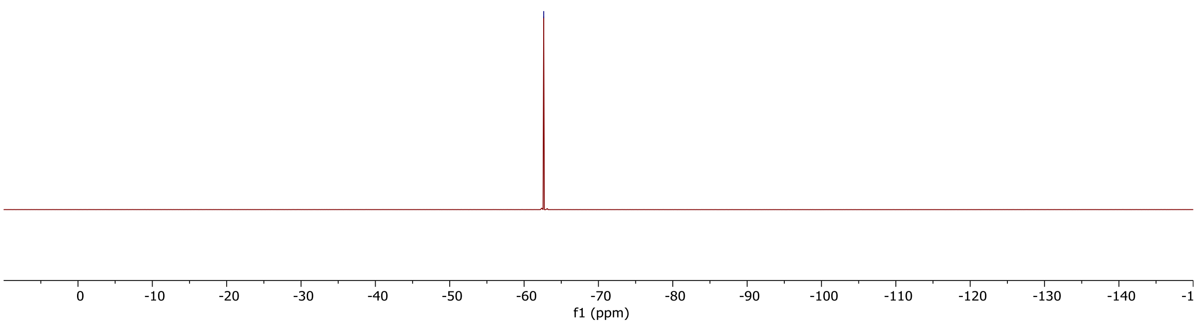

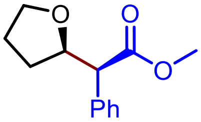

S35
^{1}H NMR (400 MHz, $CDCl_3$)

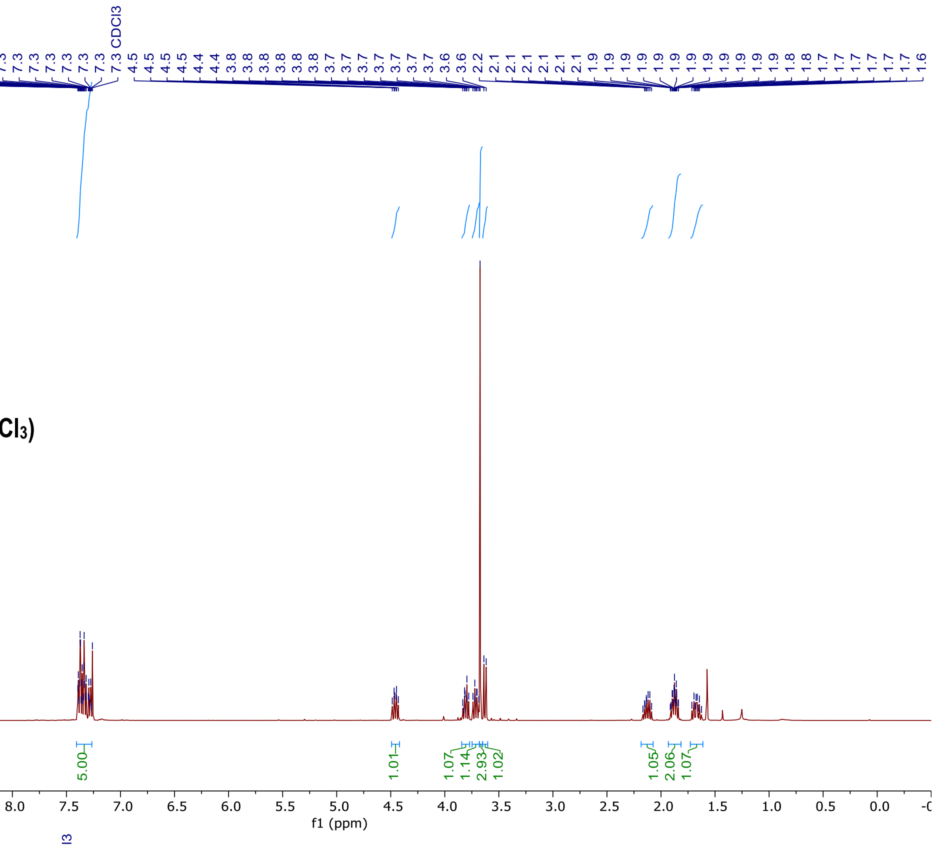

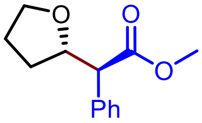

S36
^{1}H NMR (400 MHz, $CDCl_3$)

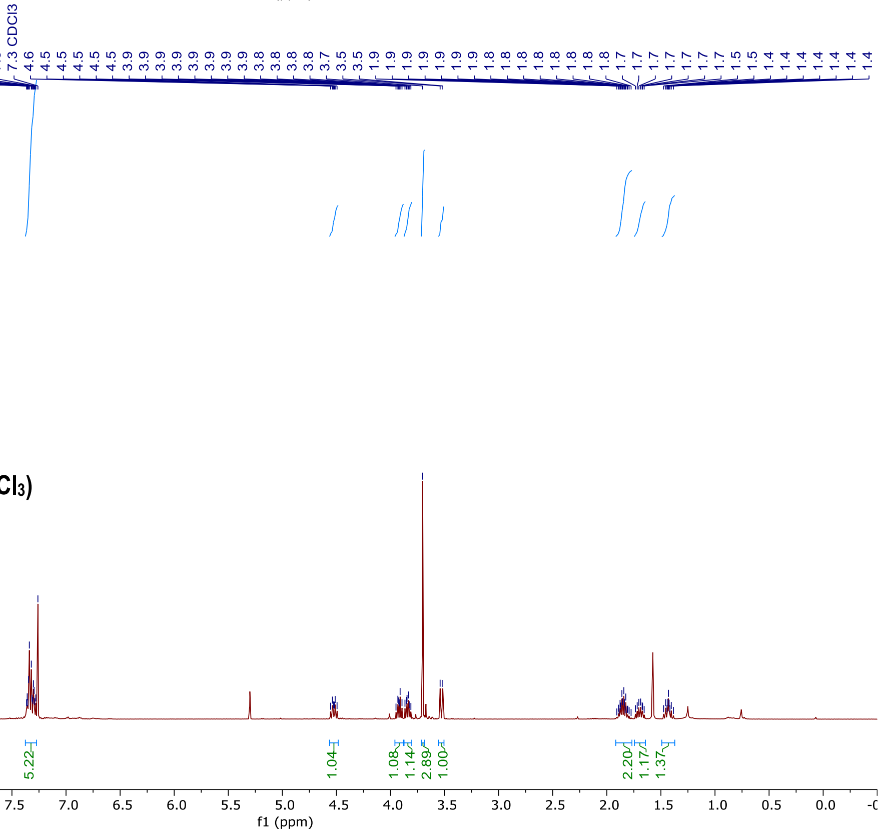

7. HPLC/SFC Data

Absolute configuration: the absolute configuration is tentatively assigned by analogy based on X-ray crystallography.

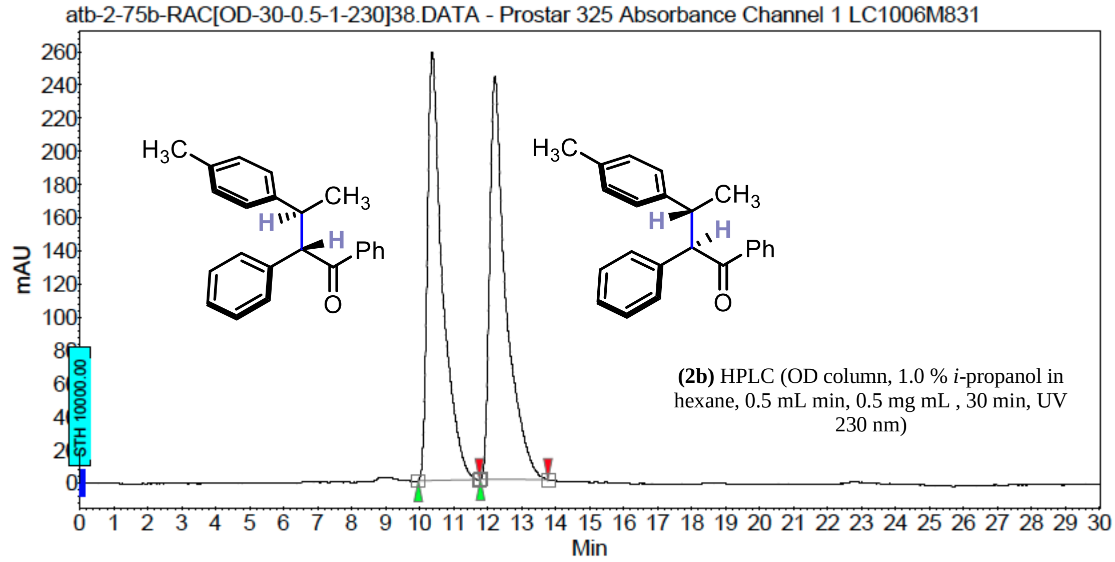

Peak results :

Index	Name	Time [Min]	Quantity [% Area]	Height [mAU]	Area [mAU.Min]	Area % [%]
1	UNKNOWN	10.38	49.51	257.7	133.4	49.515
2	UNKNOWN	12.22	50.49	243.1	136.0	50.485
Total			100.00	500.8	269.5	100.000

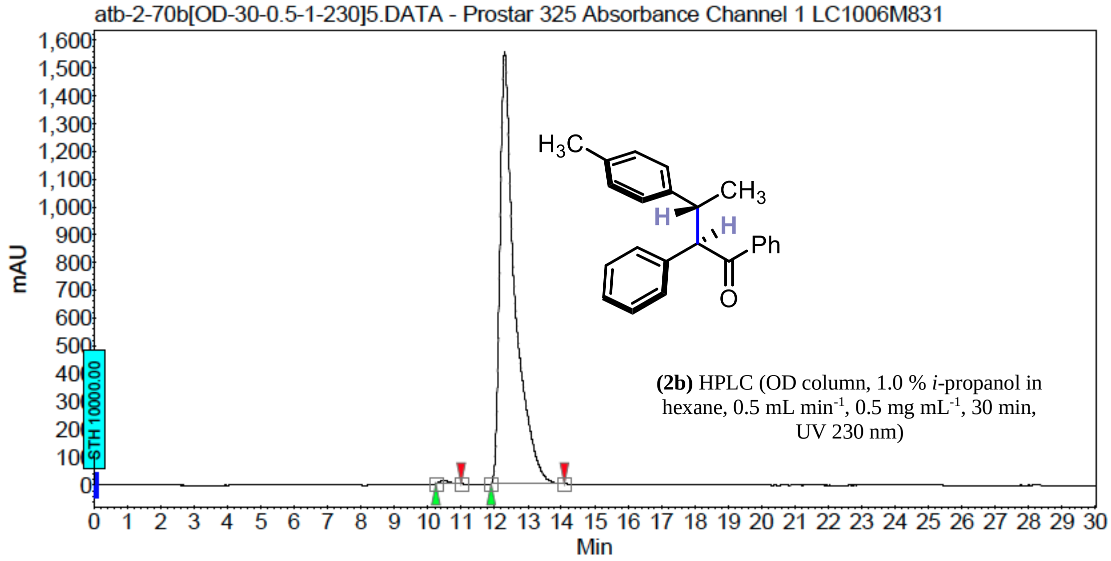

Peak results :

Index	Name	Time [Min]	Quantity [% Area]	Height [mAU]	Area [mAU.Min]	Area % [%]
1	UNKNOWN	10.45	0.55	12.2	4.5	0.549
2	UNKNOWN	12.30	99.45	1554.0	809.9	99.451
Total			100.00	1566.2	814.4	100.000

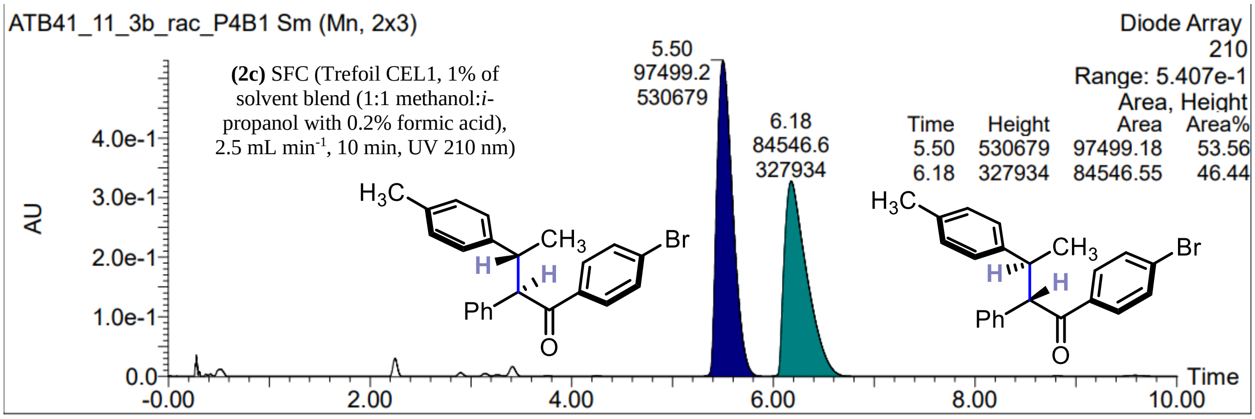

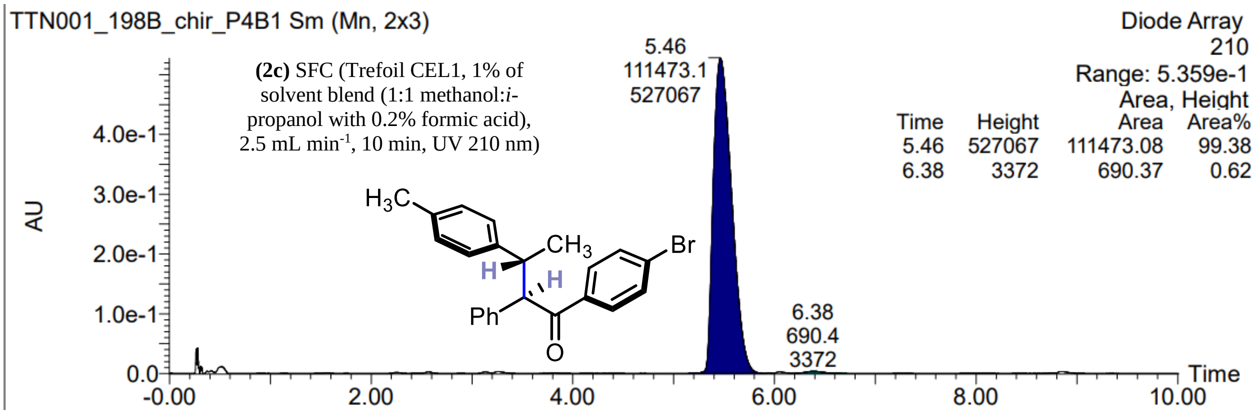

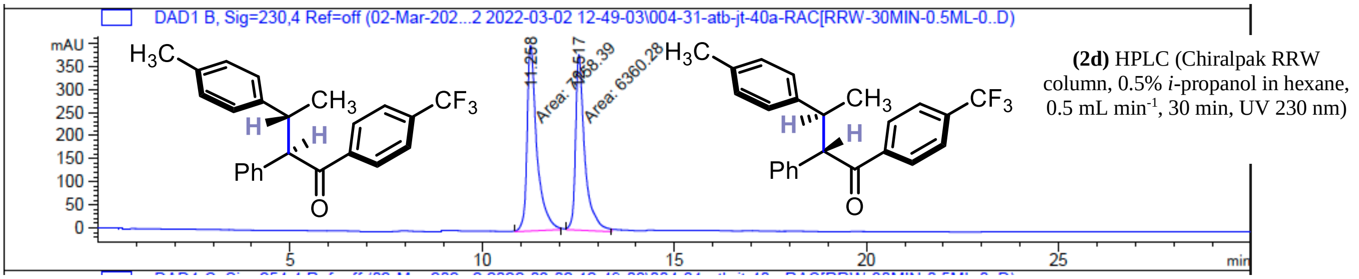

Signal 2: DAD1 B, Sig=230,4 Ref=off

Peak #	RetTime [min]	Type	Width [min]	Area [mAU*s]	Height [mAU]	Area %
1	11.258	MM	0.2971	7158.38770	401.52637	52.9519
2	12.517	MM	0.2790	6360.27539	379.92096	47.0481

Totals : 1.35187e4 781.44733

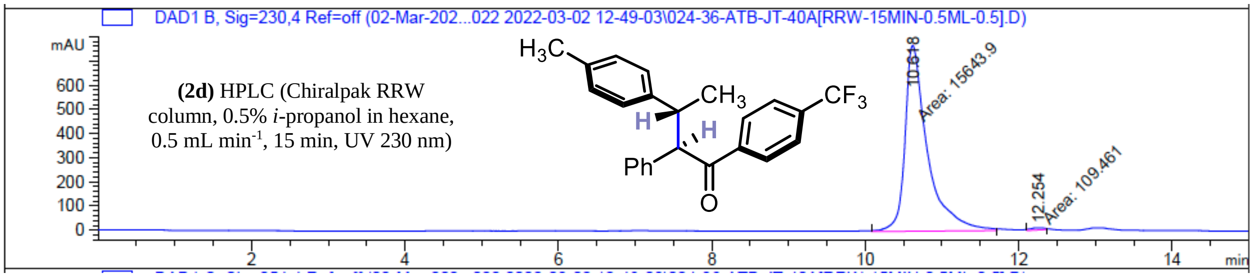

(2d) HPLC (Chiralpak RRW column, 0.5% *i*-propanol in hexane, 0.5 mL min^{-1}, 15 min, UV 230 nm)

Signal 2: DAD1 B, Sig=230,4 Ref=off

Peak #	RetTime [min]	Type	Width [min]	Area [mAU*s]	Height [mAU]	Area %
1	10.618	MM	0.3393	1.56439e4	768.34760	99.3052
2	12.254	MM	0.1433	109.46097	9.15441	0.6948
Totals :				1.57534e4	777.50201	

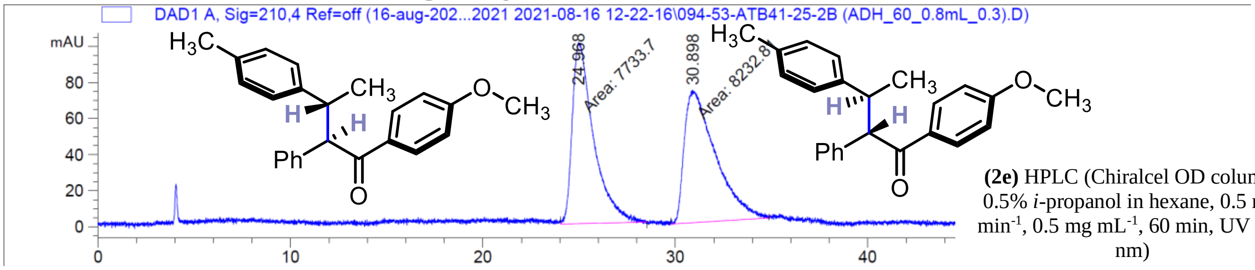

(2e) HPLC (Chiralcel OD column, 0.5% *i*-propanol in hexane, 0.5 mL min^{-1}, 0.5 mg mL^{-1}, 60 min, UV 210 nm)

Signal 1: DAD1 A, Sig=210,4 Ref=off

Peak #	RetTime [min]	Type	Width [min]	Area [mAU*s]	Height [mAU]	Area %
1	24.968	MM	1.2830	7733.70264	100.46152	48.4370
2	30.898	MM	1.8674	8232.81055	73.47857	51.5630
Totals :				1.59665e4	173.94009	

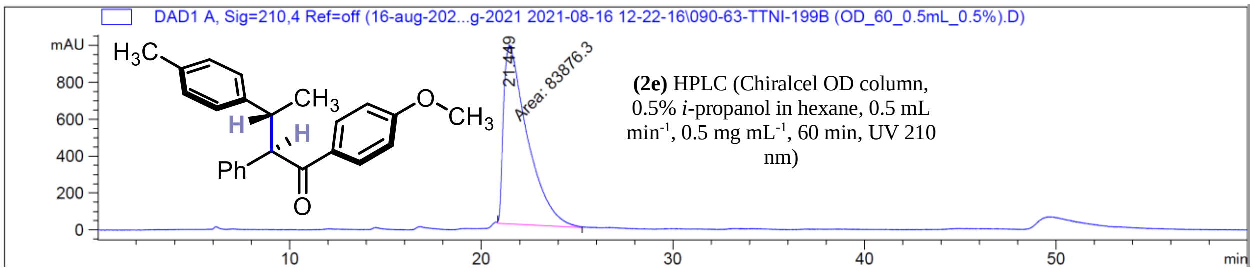

(2e) HPLC (Chiralcel OD column, 0.5% *i*-propanol in hexane, 0.5 mL min^{-1}, 0.5 mg mL^{-1}, 60 min, UV 210 nm)

Signal 1: DAD1 A, Sig=210,4 Ref=off

Peak #	RetTime [min]	Type	Width [min]	Area [mAU*s]	Height [mAU]	Area %
1	21.449	MM	1.4376	8.38763e4	972.39227	100.0000
Totals :				8.38763e4	972.39227	

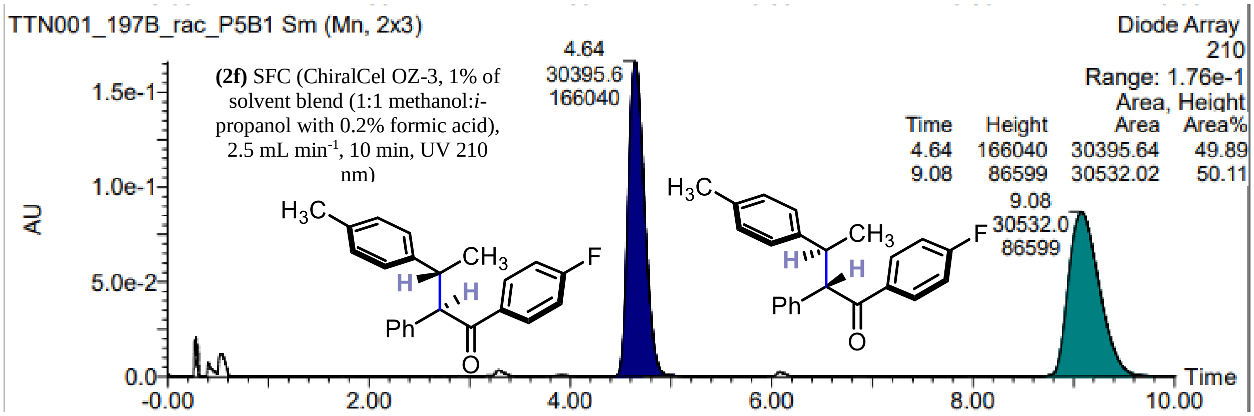

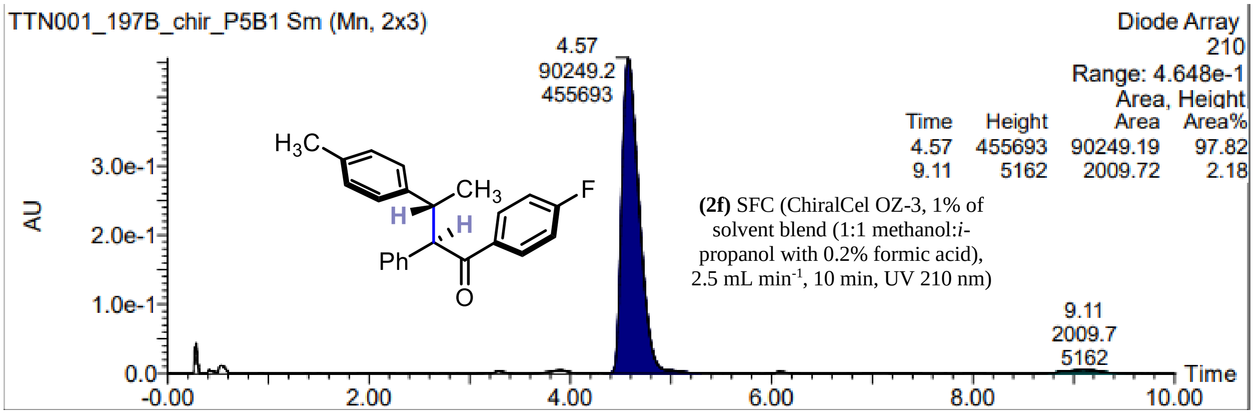

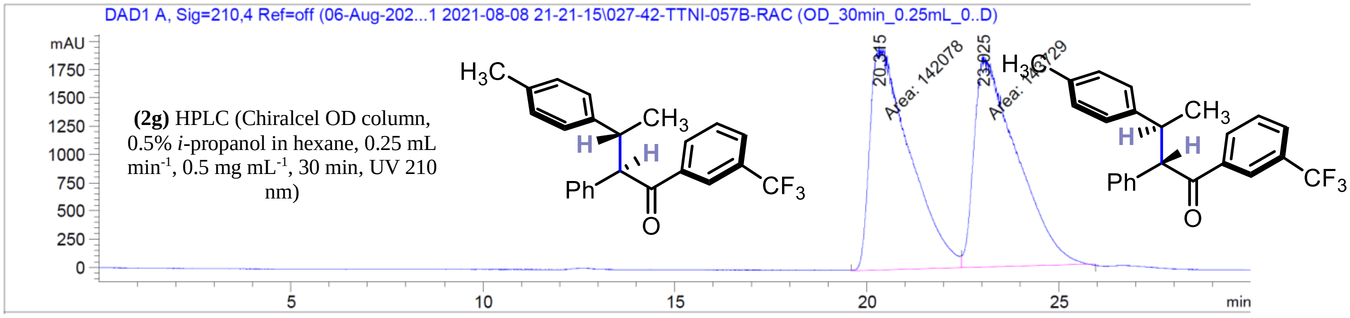

Signal 1: DAD1 A, Sig=210,4 Ref=off

Peak #	RetTime [min]	Type	Width [min]	Area [mAU*s]	Height [mAU]	Area %
1	20.315	MF	1.1911	1.42078e5	1988.09167	49.7111
2	23.025	FM	1.2768	1.43729e5	1876.10706	50.2889
Totals :				2.85807e5	3864.19873	

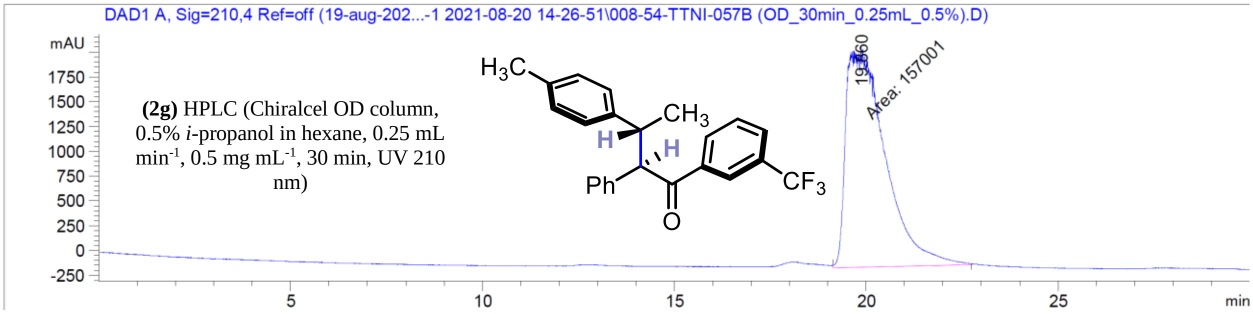

Signal 1: DAD1 A, Sig=210,4 Ref=off

Peak #	RetTime [min]	Type	Width [min]	Area [mAU*s]	Height [mAU]	Area %
1	19.860	MM	1.1682	1.57001e5	2239.87354	100.0000

Totals : 1.57001e5 2239.87354

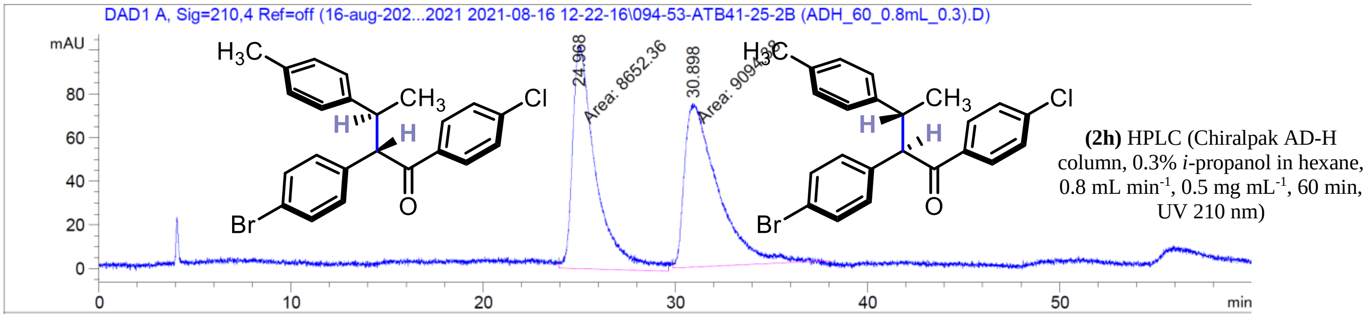

Signal 1: DAD1 A, Sig=210,4 Ref=off

Peak #	RetTime [min]	Type	Width [min]	Area [mAU*s]	Height [mAU]	Area %
1	24.968	MM	1.4103	8652.35742	102.25462	48.7547
2	30.898	MM	2.0190	9094.37500	75.07294	51.2453

Totals : 1.77467e4 177.32756

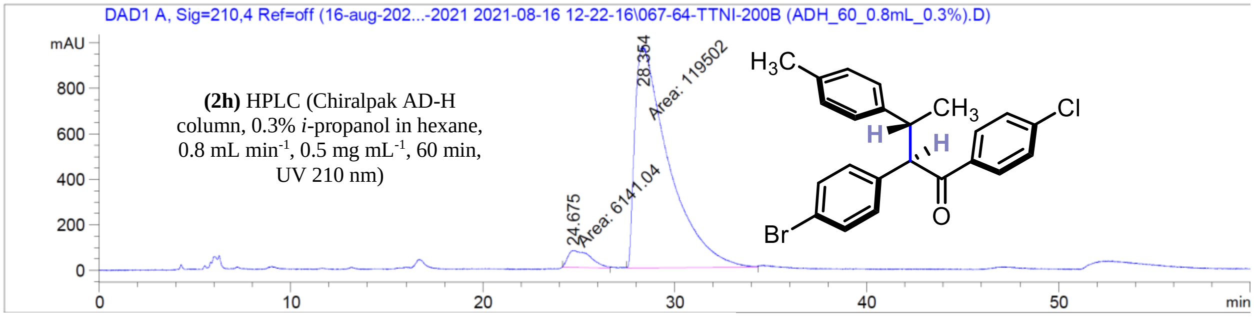

Signal 1: DAD1 A, Sig=210,4 Ref=off

Peak #	RetTime [min]	Type	Width [min]	Area [mAU*s]	Height [mAU]	Area %
1	24.675	MM	1.3670	6141.03760	74.87107	4.8877
2	28.354	MM	2.0366	1.19502e5	977.93500	95.1123

Totals : 1.25643e5 1052.80607

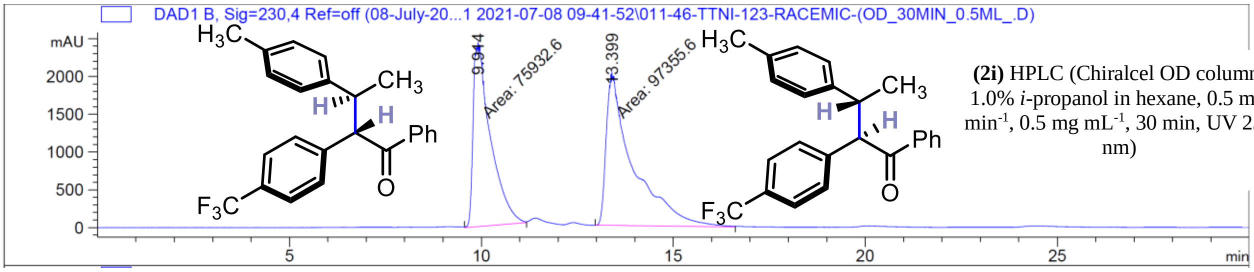

(2i) HPLC (Chiralcel OD column, 1.0% *i*-propanol in hexane, 0.5 mL min^{-1}, 0.5 mg mL^{-1}, 30 min, UV 230 nm)

Signal 2: DAD1 B, Sig=230,4 Ref=off

Peak #	RetTime [min]	Type	Width [min]	Area [mAU*s]	Height [mAU]	Area %
1	9.914	MM	0.5167	7.59326e4	2449.37964	43.8187
2	13.399	MM	0.7970	9.73556e4	2035.75317	56.1813

Totals : 1.73288e5 4485.13281

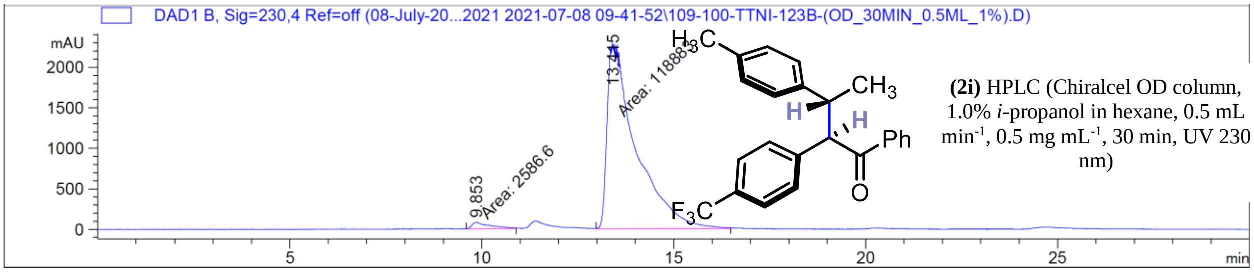

(2i) HPLC (Chiralcel OD column, 1.0% *i*-propanol in hexane, 0.5 mL min^{-1}, 0.5 mg mL^{-1}, 30 min, UV 230 nm)

Signal 2: DAD1 B, Sig=230,4 Ref=off

Peak #	RetTime [min]	Type	Width [min]	Area [mAU*s]	Height [mAU]	Area %
1	9.853	MM	0.5322	2586.60327	81.00648	2.1294
2	13.415	MM	0.8639	1.18883e5	2293.63428	97.8706

Totals : 1.21470e5 2374.64076

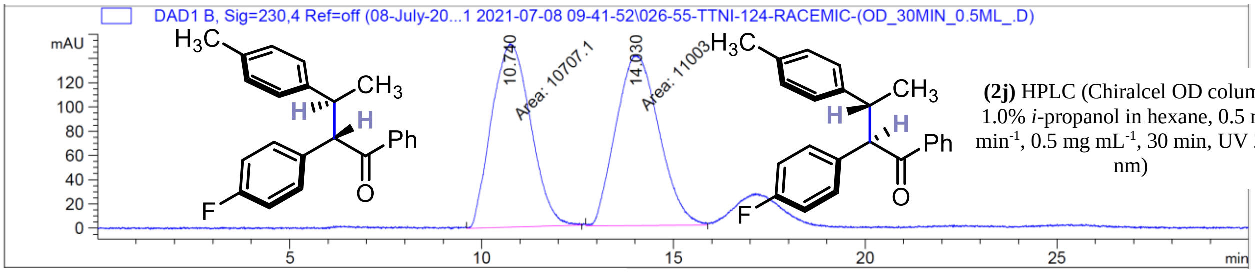

(2j) HPLC (Chiralcel OD column, 1.0% *i*-propanol in hexane, 0.5 mL min^{-1}, 0.5 mg mL^{-1}, 30 min, UV 230 nm)

Signal 2: DAD1 B, Sig=230,4 Ref=off

Peak #	RetTime [min]	Type	Width [min]	Area [mAU*s]	Height [mAU]	Area %
1	10.740	MM	1.1764	1.07071e4	151.69272	49.3186
2	14.030	MM	1.3039	1.10030e4	140.64478	50.6814

Totals : 2.17101e4 292.33749

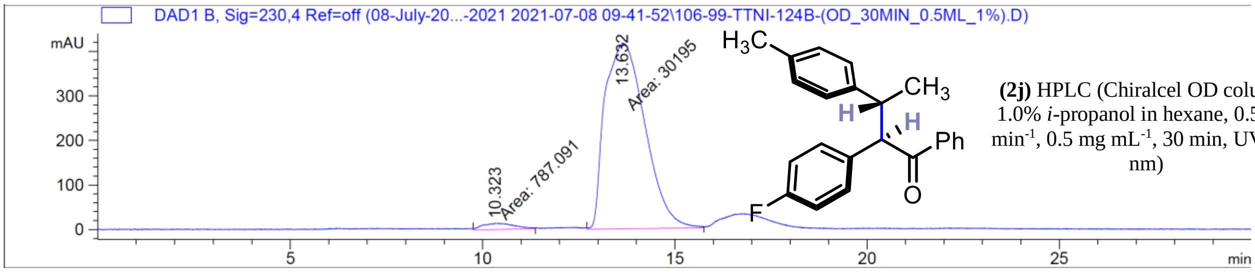

(2j) HPLC (Chiralcel OD column, 1.0% *i*-propanol in hexane, 0.5 mL min^{-1}, 0.5 mg mL^{-1}, 30 min, UV 230 nm)

Signal 2: DAD1 B, Sig=230,4 Ref=off

Peak #	RetTime [min]	Type	Width [min]	Area [mAU*s]	Height [mAU]	Area %
1	10.323	MM	0.9321	787.09052	14.07446	2.5405
2	13.632	MM	1.2065	3.01950e4	417.11780	97.4595

Totals : 3.09821e4 431.19226

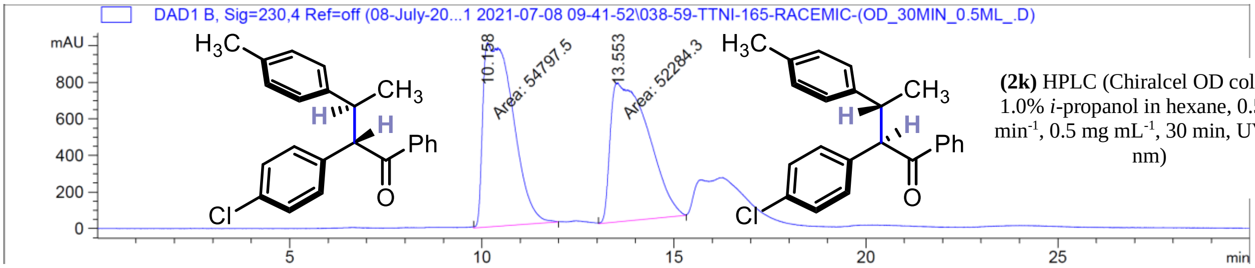

(2k) HPLC (Chiralcel OD column, 1.0% *i*-propanol in hexane, 0.5 mL min^{-1}, 0.5 mg mL^{-1}, 30 min, UV 230 nm)

Signal 2: DAD1 B, Sig=230,4 Ref=off

Peak #	RetTime [min]	Type	Width [min]	Area [mAU*s]	Height [mAU]	Area %
1	10.158	MM	0.9043	5.47975e4	1009.95337	51.1735
2	13.553	MM	1.1437	5.22843e4	761.91058	48.8265

Totals : 1.07082e5 1771.86395

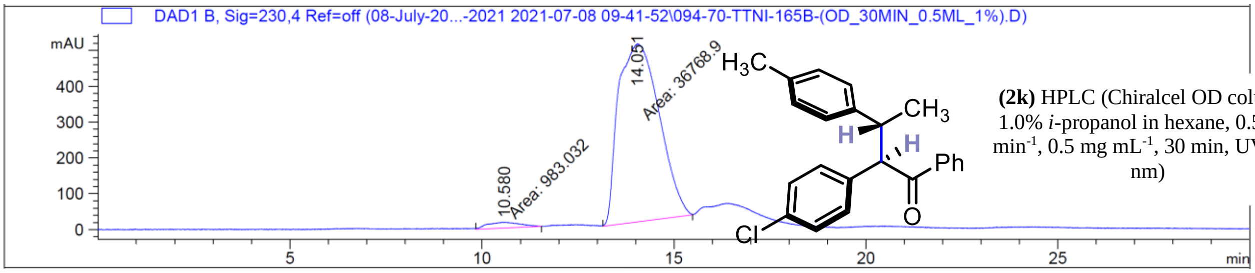

(2k) HPLC (Chiralcel OD column, 1.0% *i*-propanol in hexane, 0.5 mL min^{-1}, 0.5 mg mL^{-1}, 30 min, UV 230 nm)

Signal 2: DAD1 B, Sig=230,4 Ref=off

Peak #	RetTime [min]	Type	Width [min]	Area [mAU*s]	Height [mAU]	Area %
1	10.580	MM	0.9893	983.03162	16.56162	2.6039
2	14.051	MM	1.2305	3.67689e4	498.01541	97.3961

Totals : 3.77520e4 514.57703

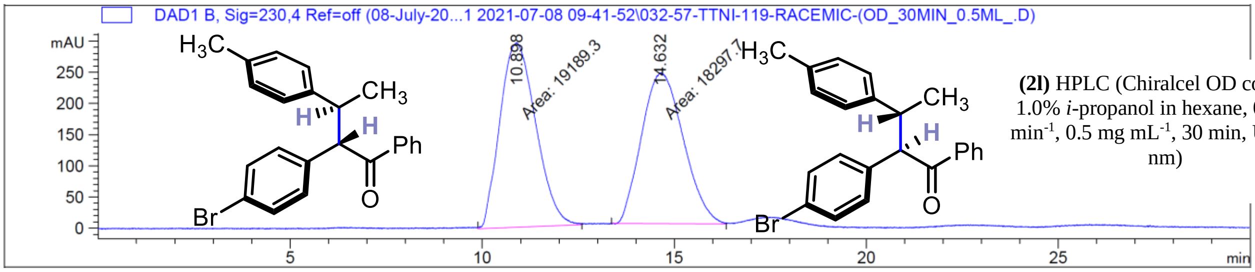

(2l) HPLC (Chiralcel OD column, 1.0% *i*-propanol in hexane, 0.5 mL min^{-1}, 0.5 mg mL^{-1}, 30 min, UV 230 nm)

Signal 2: DAD1 B, Sig=230,4 Ref=off

Peak #	RetTime [min]	Type	Width [min]	Area [mAU*s]	Height [mAU]	Area %
1	10.898	MM	1.0825	1.91893e4	295.44821	51.1892
2	14.632	MM	1.2624	1.82977e4	241.57219	48.8108

Totals : 3.74870e4 537.02040

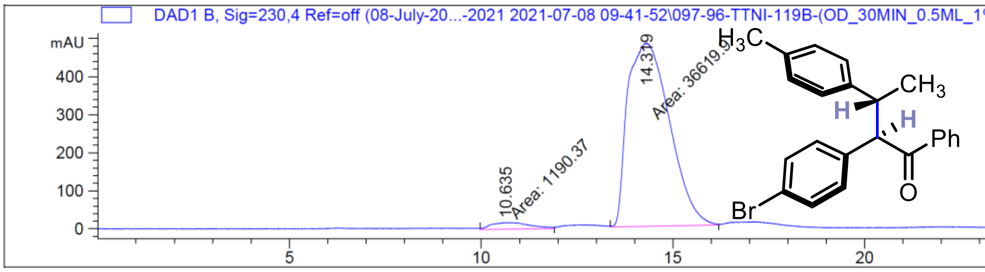

(2l) HPLC (Chiralcel OD column, 1.0% *i*-propanol in hexane, 0.5 mL min^{-1}, 0.5 mg mL^{-1}, 30 min, UV 230 nm)

Signal 2: DAD1 B, Sig=230,4 Ref=off

Peak #	RetTime [min]	Type	Width [min]	Area [mAU*s]	Height [mAU]	Area %
1	10.635	MM	1.1373	1190.36694	17.44499	3.1483
2	14.319	MM	1.2653	3.66199e4	482.36472	96.8517

Totals : 3.78102e4 499.80971

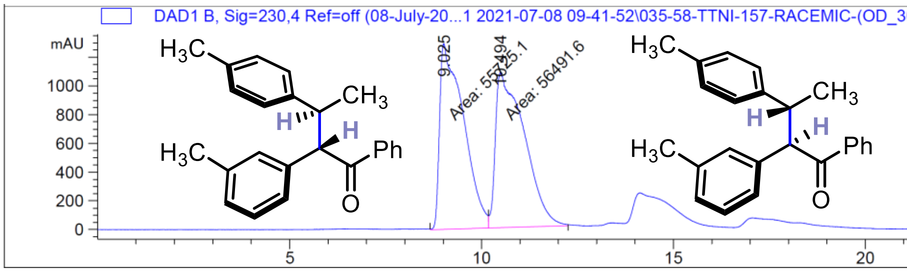

(2m) HPLC (Chiralcel OD column, 1.0% *i*-propanol in hexane, 0.5 mL min^{-1}, 0.5 mg mL^{-1}, 30 min, UV 230 nm)

Signal 2: DAD1 B, Sig=230,4 Ref=off

Peak #	RetTime [min]	Type	Width [min]	Area [mAU*s]	Height [mAU]	Area %
1	9.025	MF	0.7177	5.57251e4	1293.98608	49.6584
2	10.494	FM	0.8704	5.64916e4	1081.73840	50.3416

Totals : 1.12217e5 2375.72449

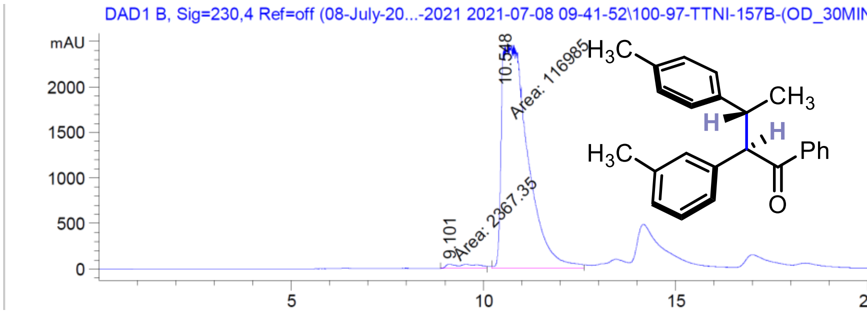

(2m) HPLC (Chiralcel OD column, 1.0% *i*-propanol in hexane, 0.5 mL min^{-1}, 0.5 mg mL^{-1}, 30 min, UV 230 nm)

Signal 2: DAD1 B, Sig=230,4 Ref=off

Peak #	RetTime [min]	Type	Width [min]	Area [mAU*s]	Height [mAU]	Area %
1	9.101	MM	0.7381	2356.49512	53.21304	2.0279
2	10.548	MM	0.7759	1.13846e5	2445.54614	97.9721

Totals : 1.16202e5 2498.75918

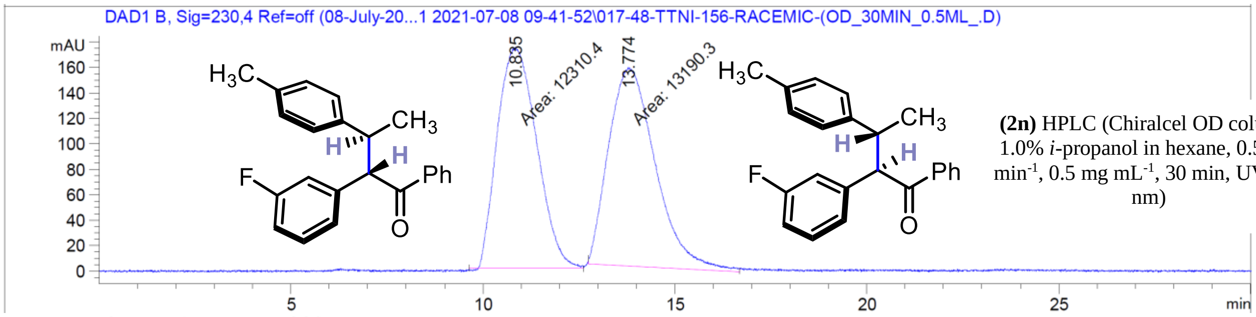

(2n) HPLC (Chiralcel OD column, 1.0% *i*-propanol in hexane, 0.5 mL min^{-1}, 0.5 mg mL^{-1}, 30 min, UV 230 nm)

Signal 2: DAD1 B, Sig=230,4 Ref=off

Peak #	RetTime [min]	Type	Width [min]	Area [mAU*s]	Height [mAU]	Area %
1	10.835	MM	1.1841	1.23104e4	173.27161	48.2747
2	13.774	MM	1.4095	1.31903e4	155.96814	51.7253
Totals :				2.55006e4	329.23975	

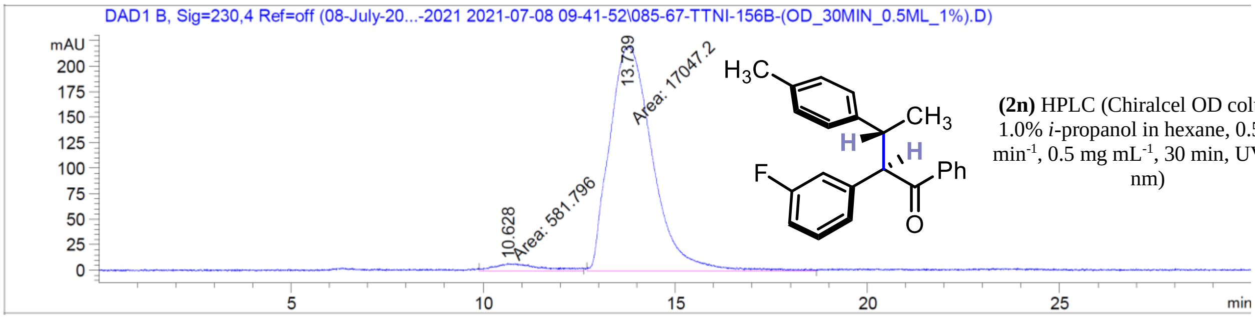

(2n) HPLC (Chiralcel OD column, 1.0% *i*-propanol in hexane, 0.5 mL min^{-1}, 0.5 mg mL^{-1}, 30 min, UV 230 nm)

Signal 2: DAD1 B, Sig=230,4 Ref=off

Peak #	RetTime [min]	Type	Width [min]	Area [mAU*s]	Height [mAU]	Area %
1	10.628	MM	1.3741	581.79608	7.05648	3.3002
2	13.739	MM	1.2918	1.70472e4	219.94418	96.6998
Totals :				1.76290e4	227.00066	

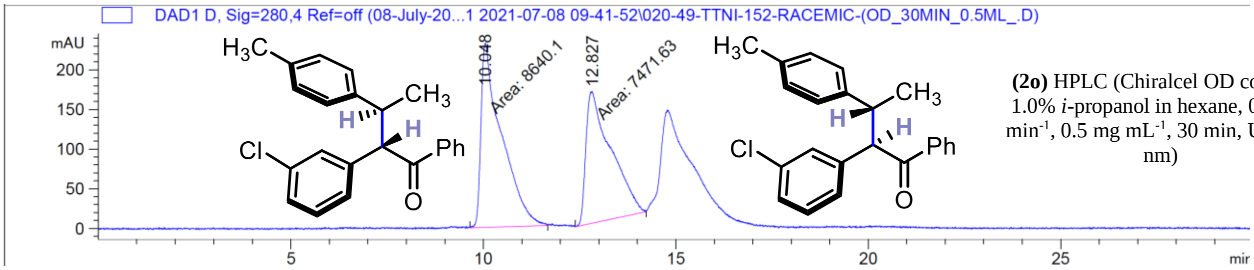

(2o) HPLC (Chiralcel OD column, 1.0% *i*-propanol in hexane, 0.5 mL min^{-1}, 0.5 mg mL^{-1}, 30 min, UV 280 nm)

Signal 4: DAD1 D, Sig=280,4 Ref=off

Peak #	RetTime [min]	Type	Width [min]	Area [mAU*s]	Height [mAU]	Area %
1	10.048	MM	0.6192	8640.10254	232.54771	53.6262
2	12.827	MM	0.7495	7471.62695	166.14180	46.3738
Totals :				1.61117e4	398.68951	

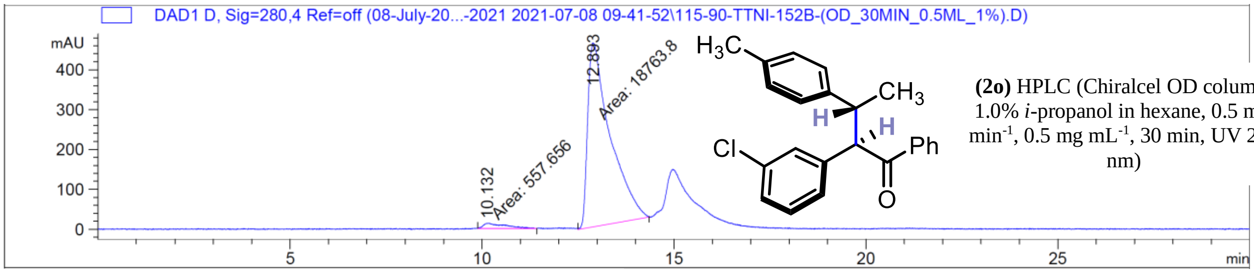

(2o) HPLC (Chiralcel OD column, 1.0% *i*-propanol in hexane, 0.5 mL min^{-1}, 0.5 mg mL^{-1}, 30 min, UV 280 nm)

Signal 4: DAD1 D, Sig=280,4 Ref=off

Peak #	RetTime [min]	Type	Width [min]	Area [mAU*s]	Height [mAU]	Area %
1	10.132	MM	0.6848	557.65552	13.57257	2.8862
2	12.893	MM	0.6779	1.87638e4	461.28848	97.1138

Totals : 1.93214e4 474.86105

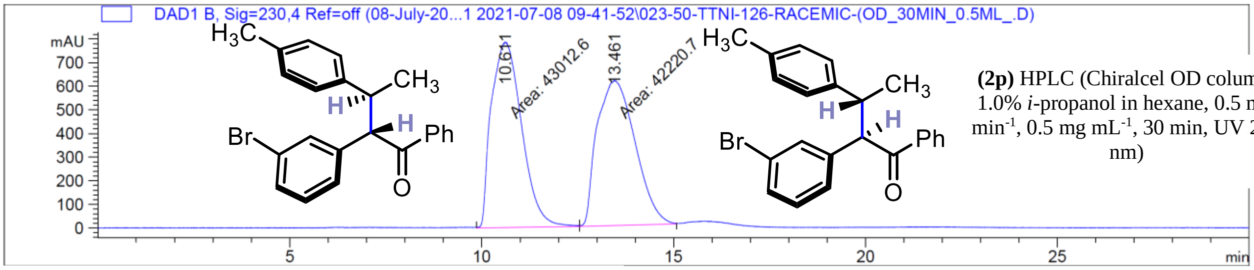

(2p) HPLC (Chiralcel OD column, 1.0% *i*-propanol in hexane, 0.5 mL min^{-1}, 0.5 mg mL^{-1}, 30 min, UV 230 nm)

Signal 2: DAD1 B, Sig=230,4 Ref=off

Peak #	RetTime [min]	Type	Width [min]	Area [mAU*s]	Height [mAU]	Area %
1	10.611	MM	0.9119	4.30126e4	786.17682	50.4645
2	13.461	MM	1.1441	4.22207e4	615.05444	49.5355

Totals : 8.52333e4 1401.23126

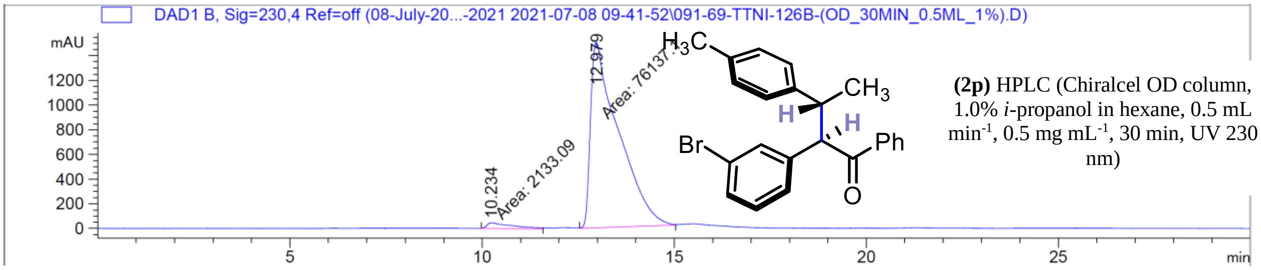

(2p) HPLC (Chiralcel OD column, 1.0% *i*-propanol in hexane, 0.5 mL min^{-1}, 0.5 mg mL^{-1}, 30 min, UV 230 nm)

Signal 2: DAD1 B, Sig=230,4 Ref=off

Peak #	RetTime [min]	Type	Width [min]	Area [mAU*s]	Height [mAU]	Area %
1	10.234	MM	0.7606	2133.09277	46.73950	2.7253
2	12.979	MM	0.8447	7.61371e4	1502.17407	97.2747

Totals : 7.82702e4 1548.91357

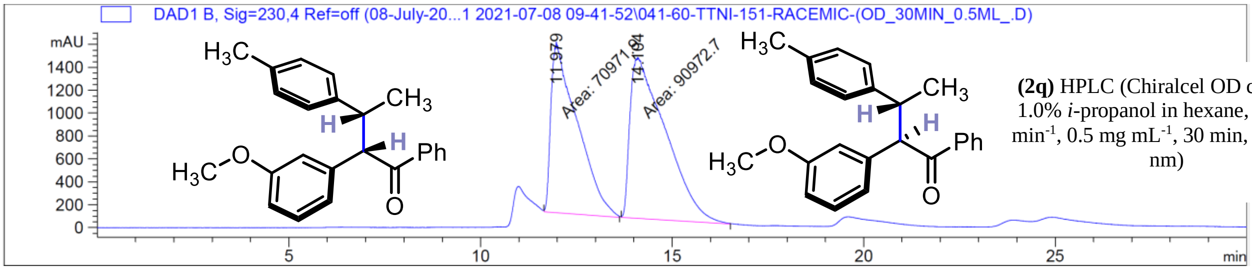

(2q) HPLC (Chiralcel OD column, 1.0% *i*-propanol in hexane, 0.5 mL min^{-1}, 0.5 mg mL^{-1}, 30 min, UV 230 nm)

Signal 2: DAD1 B, Sig=230,4 Ref=off

Peak #	RetTime [min]	Type	Width [min]	Area [mAU*s]	Height [mAU]	Area %
1	11.979	MM	0.7926	7.09719e4	1492.39807	43.8248
2	14.104	MM	1.0763	9.09727e4	1408.77393	56.1752

Totals : 1.61945e5 2901.17200

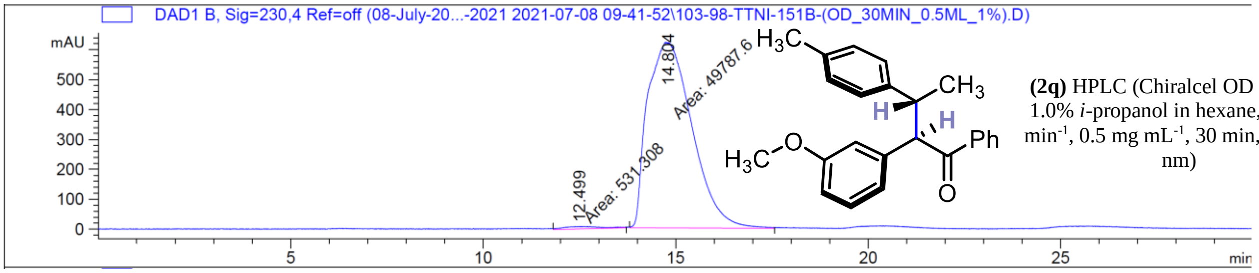

(2q) HPLC (Chiralcel OD column, 1.0% *i*-propanol in hexane, 0.5 mL min^{-1}, 0.5 mg mL^{-1}, 30 min, UV 230 nm)

Signal 2: DAD1 B, Sig=230,4 Ref=off

Peak #	RetTime [min]	Type	Width [min]	Area [mAU*s]	Height [mAU]	Area %
1	12.499	MM	1.0619	531.30823	8.33896	1.0559
2	14.804	MM	1.3376	4.97876e4	620.35730	98.9441

Totals : 5.03189e4 628.69626

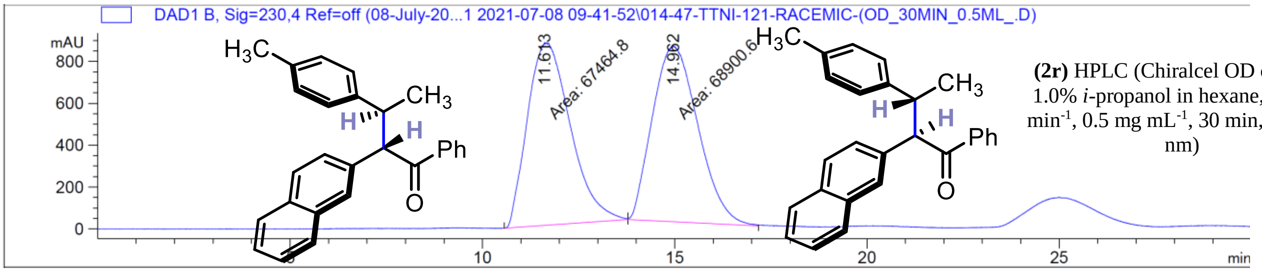

(2r) HPLC (Chiralcel OD column, 1.0% *i*-propanol in hexane, 0.5 mL min^{-1}, 0.5 mg mL^{-1}, 30 min, UV 230 nm)

Signal 2: DAD1 B, Sig=230,4 Ref=off

Peak #	RetTime [min]	Type	Width [min]	Area [mAU*s]	Height [mAU]	Area %
1	11.613	MM	1.2892	6.74648e4	872.16302	49.4735
2	14.962	MM	1.3651	6.89006e4	841.22498	50.5265

Totals : 1.36365e5 1713.38800

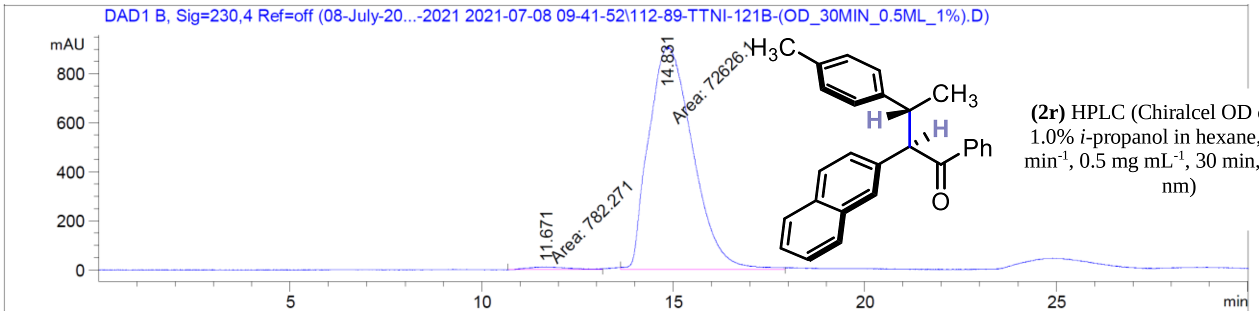

(2r) HPLC (Chiralcel OD column, 1.0% *i*-propanol in hexane, 0.5 mL min^{-1}, 0.5 mg mL^{-1}, 30 min, UV 230 nm)

Signal 2: DAD1 B, Sig=230,4 Ref=off

Peak #	RetTime [min]	Type	Width [min]	Area [mAU*s]	Height [mAU]	Area %
1	11.671	MM	1.1413	782.27069	11.42374	1.0656
2	14.831	MM	1.3287	7.26261e4	910.97052	98.9344

Totals : 7.34084e4 922.39426

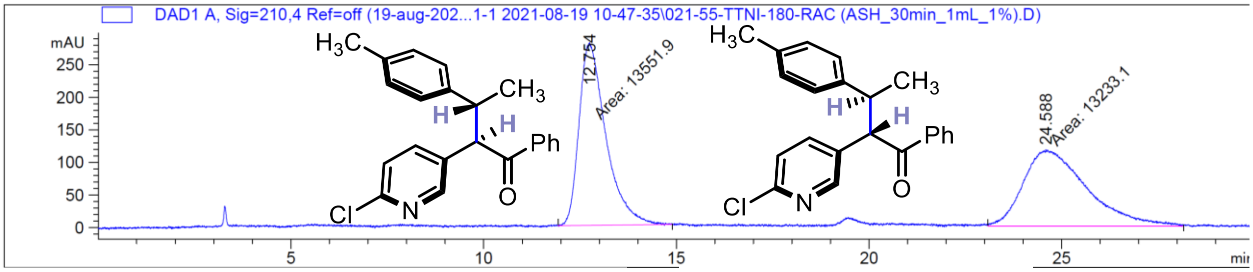

Signal 1: DAD1 A, Sig=210,4 Ref=off

Peak #	RetTime [min]	Type	Width [min]	Area [mAU*s]	Height [mAU]	Area %
1	12.754	MM	0.8048	1.35519e4	280.65030	50.5951
2	24.588	MM	1.8806	1.32331e4	117.27703	49.4049

Totals : 2.67850e4 397.92733

(2s) HPLC (Chiralpak AS-H column, 1.0% *i*-propanol in hexane, 1.0 mL min^{-1}, 0.5 mg mL^{-1}, 30 min, UV 210 nm)

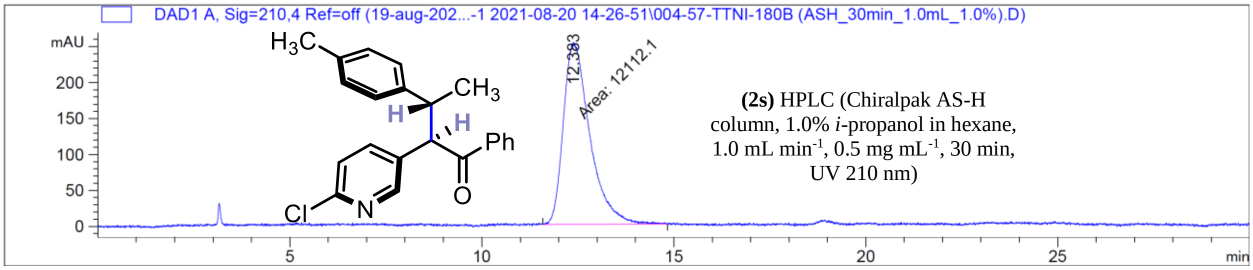

(2s) HPLC (Chiralpak AS-H column, 1.0% *i*-propanol in hexane, 1.0 mL min^{-1}, 0.5 mg mL^{-1}, 30 min, UV 210 nm)

Signal 1: DAD1 A, Sig=210,4 Ref=off

Peak #	RetTime [min]	Type	Width [min]	Area [mAU*s]	Height [mAU]	Area %
1	12.383	MM	0.7972	1.21121e4	253.22577	100.0000

Totals : 1.21121e4 253.22577

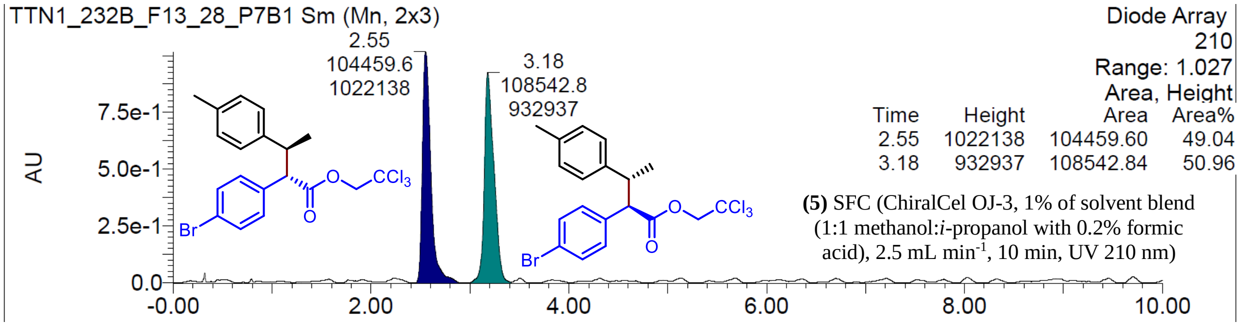

(5) SFC (ChiralCel OJ-3, 1% of solvent blend (1:1 methanol:*i*-propanol with 0.2% formic acid), 2.5 mL min^{-1}, 10 min, UV 210 nm)

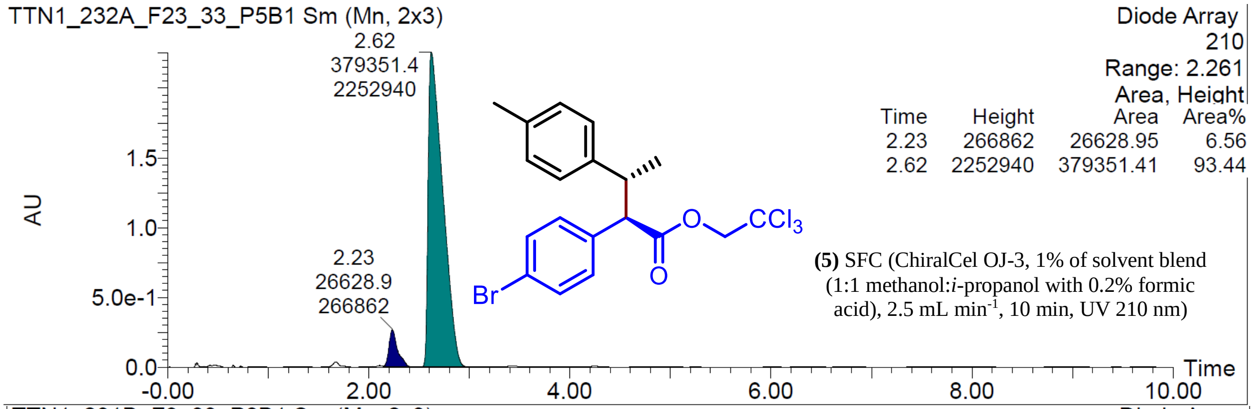

Time	Height	Area	Area%
2.23	266862	26628.95	6.56
2.62	2252940	379351.41	93.44

(5) SFC (ChiralCel OJ-3, 1% of solvent blend (1:1 methanol:*i*-propanol with 0.2% formic acid), 2.5 mL min^{-1}, 10 min, UV 210 nm)

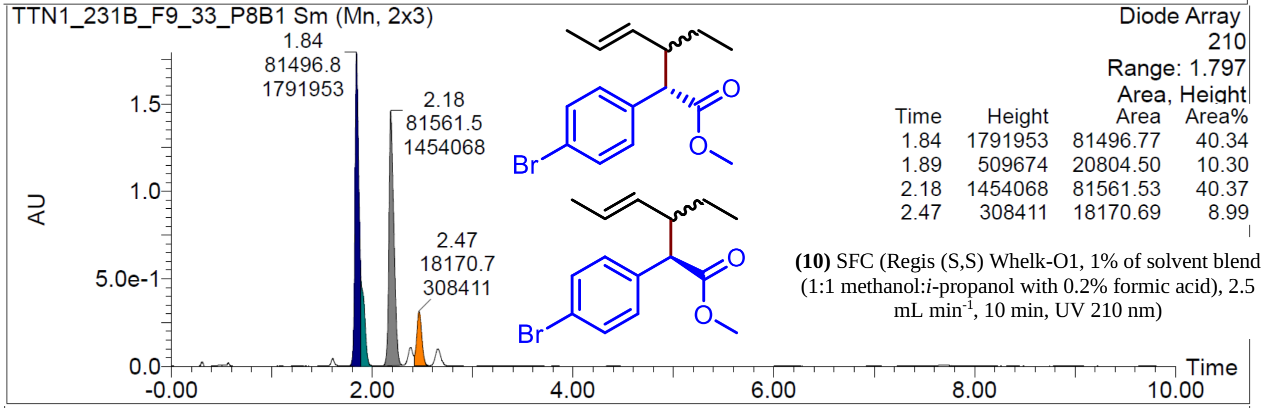

Time	Height	Area	Area%
1.84	1791953	81496.77	40.34
1.89	509674	20804.50	10.30
2.18	1454068	81561.53	40.37
2.47	308411	18170.69	8.99

(10) SFC (Regis (S,S) Whelk-O1, 1% of solvent blend (1:1 methanol:*i*-propanol with 0.2% formic acid), 2.5 mL min^{-1}, 10 min, UV 210 nm)

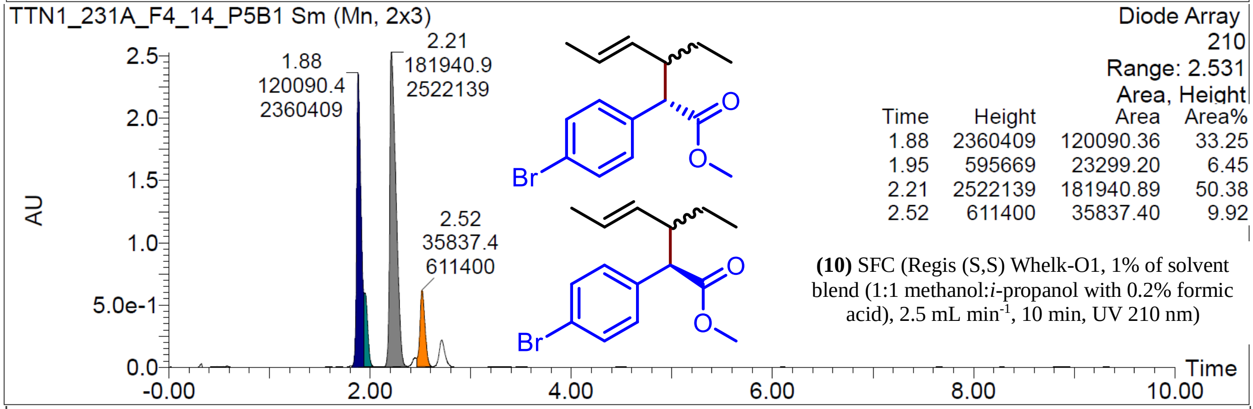

Time	Height	Area	Area%
1.88	2360409	120090.36	33.25
1.95	595669	23299.20	6.45
2.21	2522139	181940.89	50.38
2.52	611400	35837.40	9.92

(10) SFC (Regis (S,S) Whelk-O1, 1% of solvent blend (1:1 methanol:*i*-propanol with 0.2% formic acid), 2.5 mL min^{-1}, 10 min, UV 210 nm)

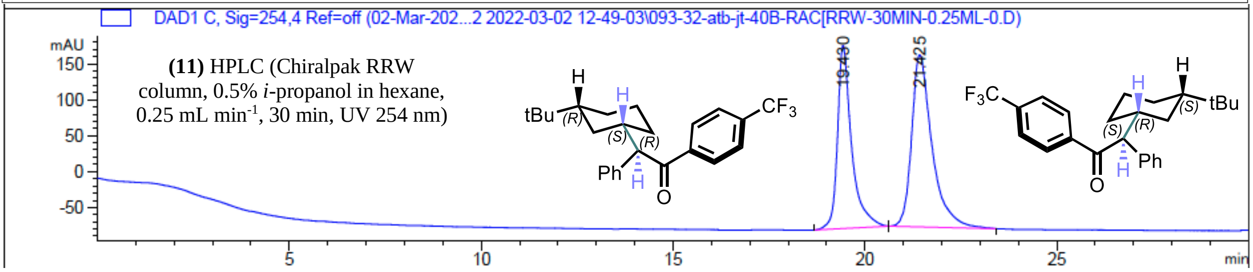

(11) HPLC (Chiralpak RRW column, 0.5% *i*-propanol in hexane, 0.25 mL min^{-1}, 30 min, UV 254 nm)

Signal 3: DAD1 C, Sig=254,4 Ref=off

Peak #	RetTime [min]	Type	Width [min]	Area [mAU*s]	Height [mAU]	Area %
1	19.430	BB	0.3554	6523.17822	255.00546	42.3810
2	21.425	BB	0.4518	8868.58594	239.63199	57.6190

Totals : 1.53918e4 494.63745

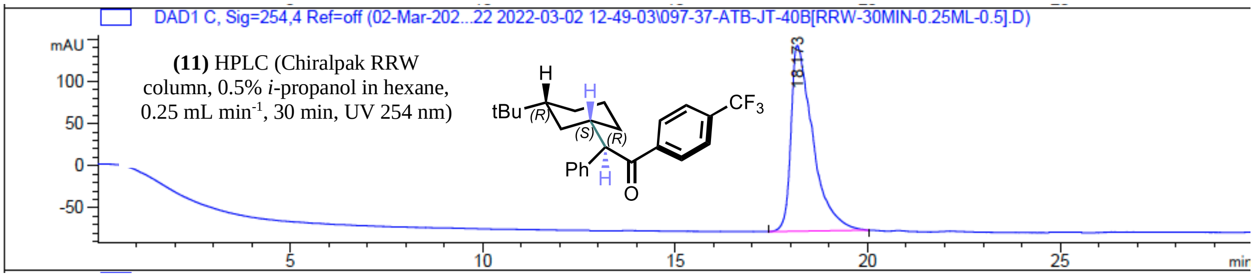

Signal 3: DAD1 C, Sig=254,4 Ref=off

Peak #	RetTime [min]	Type	Width [min]	Area [mAU*s]	Height [mAU]	Area %
1	18.173	BB	0.4717	8701.77051	219.88354	100.0000
Totals :				8701.77051	219.88354	

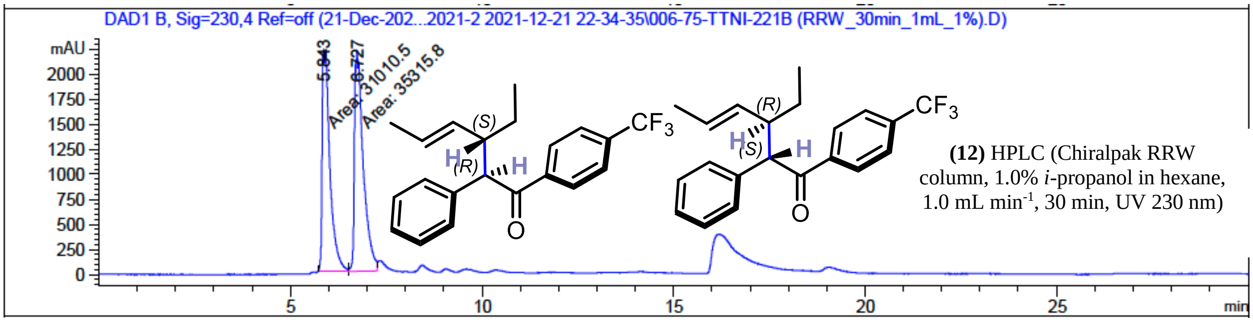

Signal 2: DAD1 B, Sig=230,4 Ref=off

Peak #	RetTime [min]	Type	Width [min]	Area [mAU*s]	Height [mAU]	Area %
1	5.843	MF	0.2332	3.10105e4	2216.41772	46.7544
2	6.727	FM	0.2666	3.53158e4	2207.50488	53.2456
Totals :				6.63263e4	4423.92261	

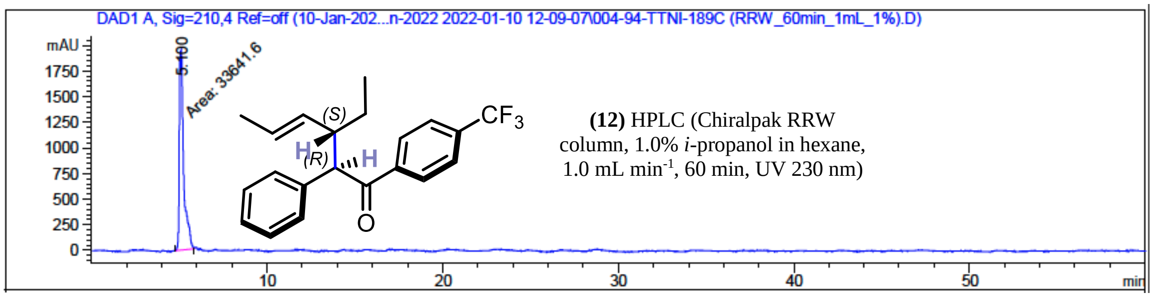

Signal 1: DAD1 A, Sig=210,4 Ref=off

Peak #	RetTime [min]	Type	Width [min]	Area [mAU*s]	Height [mAU]	Area %
1	5.100	MM	0.2826	3.36416e4	1983.71790	100.0000
Totals :				3.36416e4	1983.71790	

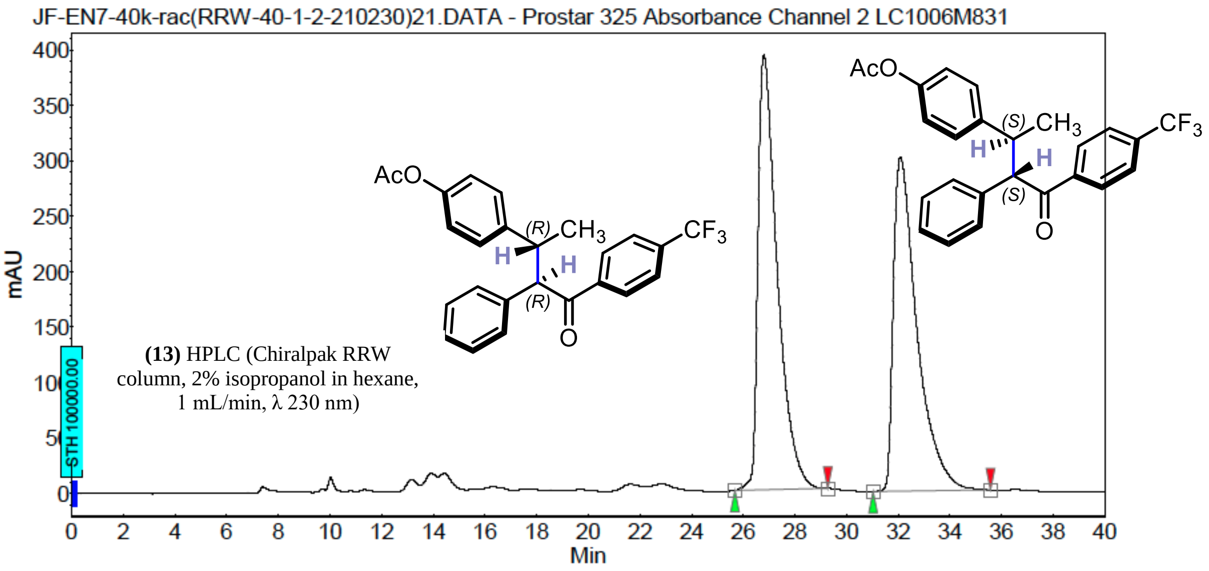

Peak results :

Index	Name	Time [Min]	Quantity [% Area]	Height [mAU]	Area [mAU.Min]	Area % [%]
1	UNKNOWN	26.81	51.59	392.4	340.2	51.591
2	UNKNOWN	32.09	48.41	301.2	319.2	48.409
Total			100.00	693.7	659.5	100.000

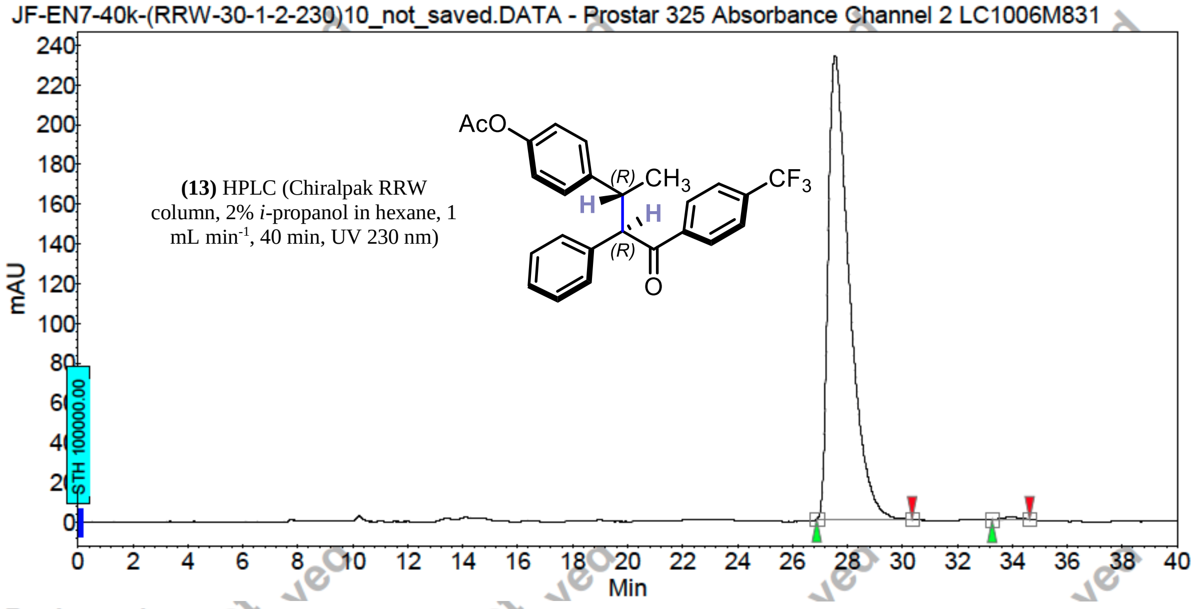

Peak results :

Index	Name	Time [Min]	Quantity [% Area]	Height [mAU]	Area [mAU.Min]	Area % [%]
1	UNKNOWN	27.55	99.50	233.8	209.8	99.498
2	UNKNOWN	33.89	0.50	1.5	1.1	0.502
Total			100.00	235.3	210.9	100.000

data not saved

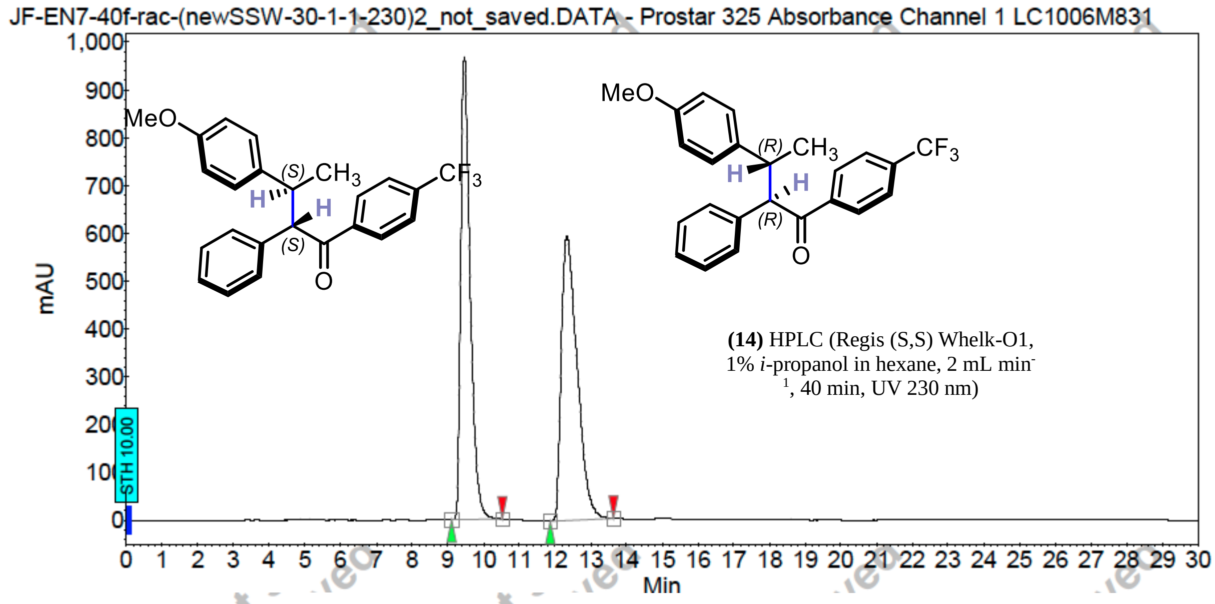

Peak results :

Index	Name	Time [Min]	Quantity [% Area]	Height [mAU]	Area [mAU.Min]	Area % [%]
1	UNKNOWN	9.46	49.28	968.3	297.0	49.282
2	UNKNOWN	12.33	50.72	595.3	305.6	50.718
Total			100.00	1563.6	602.6	100.000

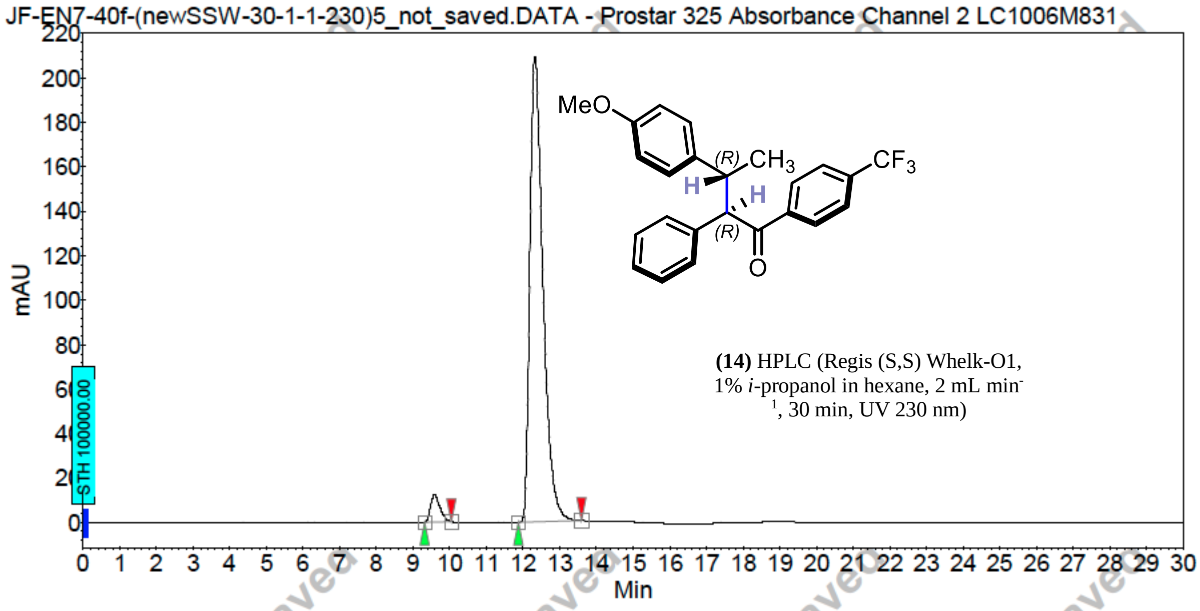

Peak results :

Index	Name	Time [Min]	Quantity [% Area]	Height [mAU]	Area [mAU.Min]	Area % [%]
1	UNKNOWN	9.58	4.03	12.3	3.6	4.034
2	UNKNOWN	12.32	95.97	209.3	86.1	95.966
Total			100.00	221.6	89.7	100.000

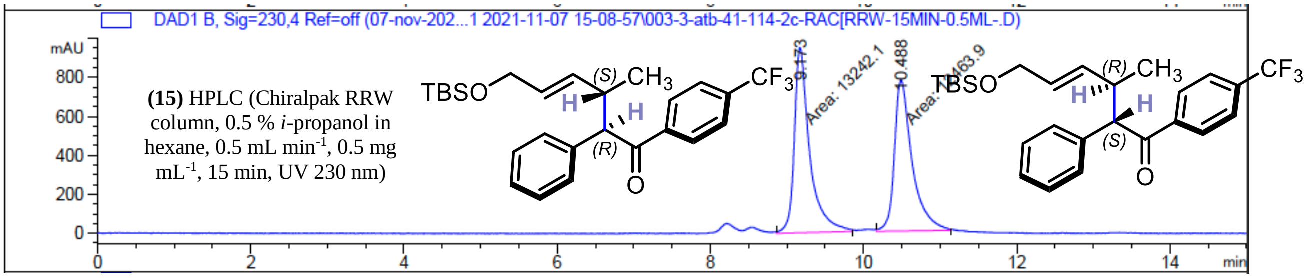

Signal 2: DAD1 B, Sig=230,4 Ref=off

Peak #	RetTime [min]	Type	Width [min]	Area [mAU*s]	Height [mAU]	Area %
1	9.173	MM	0.2322	1.32421e4	950.63538	51.5137
2	10.488	MM	0.2663	1.24639e4	780.21088	48.4863

Totals : 2.57060e4 1730.84625

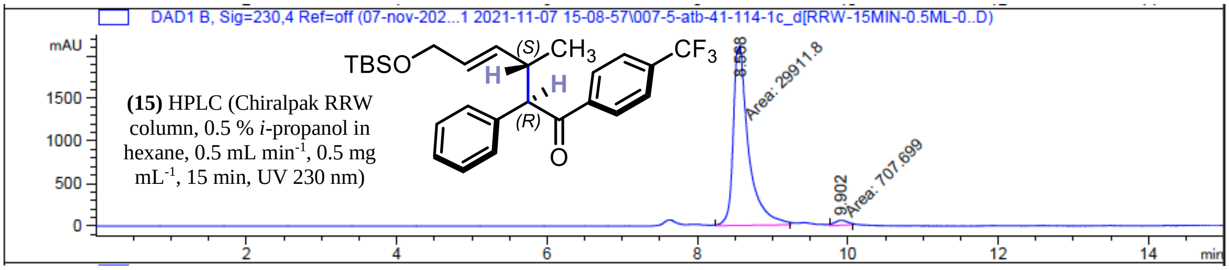

Signal 2: DAD1 B, Sig=230,4 Ref=off

Peak #	RetTime [min]	Type	Width [min]	Area [mAU*s]	Height [mAU]	Area %
1	8.568	MM	0.2344	2.99118e4	2126.39966	97.6887
2	9.902	MM	0.1947	707.69910	60.58871	2.3113

Totals : 3.06195e4 2186.98837

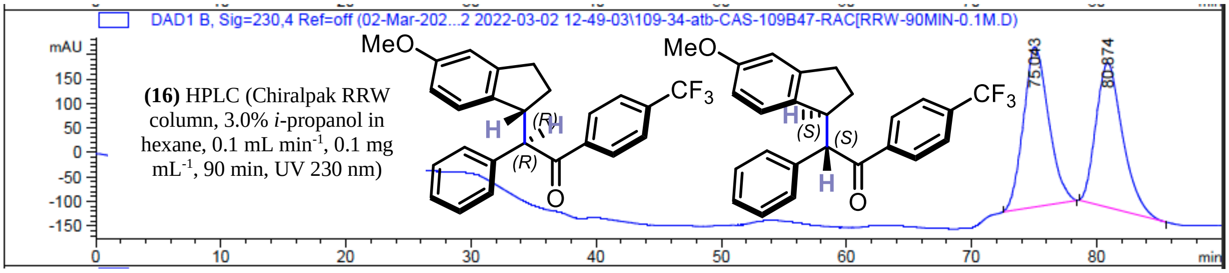

Signal 2: DAD1 B, Sig=230,4 Ref=off

Peak #	RetTime [min]	Type	Width [min]	Area [mAU*s]	Height [mAU]	Area %
1	75.043	BB	1.6340	4.56618e4	326.31503	51.1236
2	80.874	BB	1.7343	4.36547e4	294.26077	48.8764

Totals : 8.93165e4 620.57581

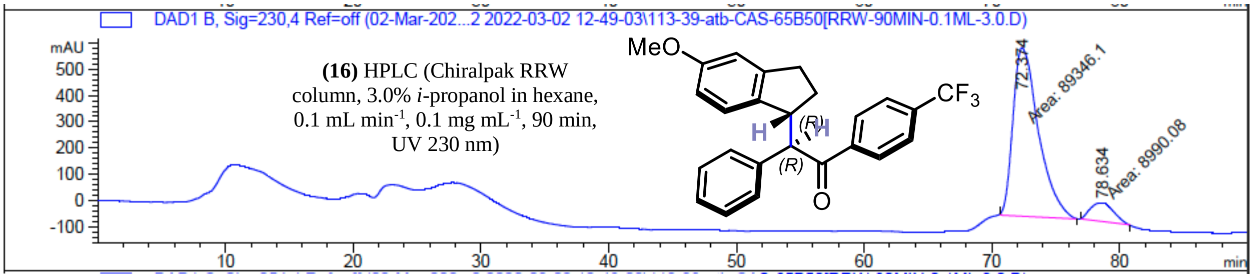

Signal 2: DAD1 B, Sig=230,4 Ref=off

Peak #	RetTime [min]	Type	Width [min]	Area [mAU*s]	Height [mAU]	Area %
1	72.374	MM	2.3145	8.93461e4	643.37170	90.8578
2	78.634	MM	2.0799	8990.08203	72.03977	9.1422

Totals : 9.83362e4 715.41148

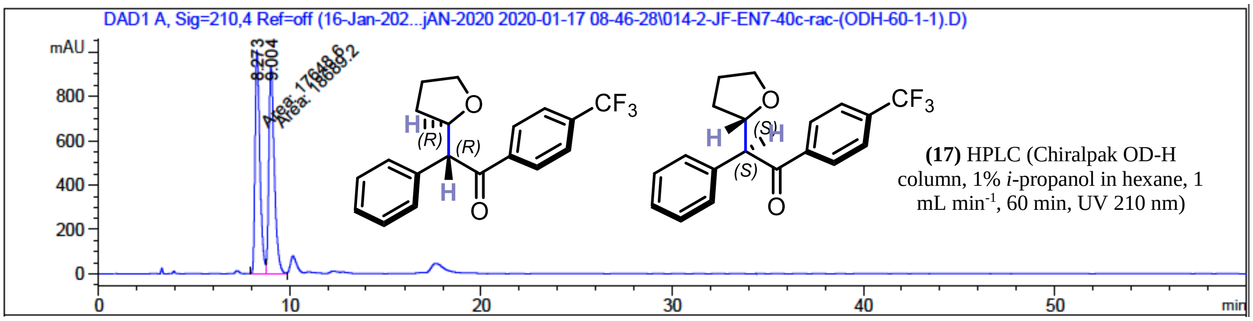

Signal 1: DAD1 A, Sig=210,4 Ref=off

Peak #	RetTime [min]	Type	Width [min]	Area [mAU*s]	Height [mAU]	Area %
1	8.273	MF	0.2915	1.76486e4	1009.18073	48.5682
2	9.004	FM	0.3341	1.86892e4	932.24548	51.4318

Totals : 3.63379e4 1941.42621

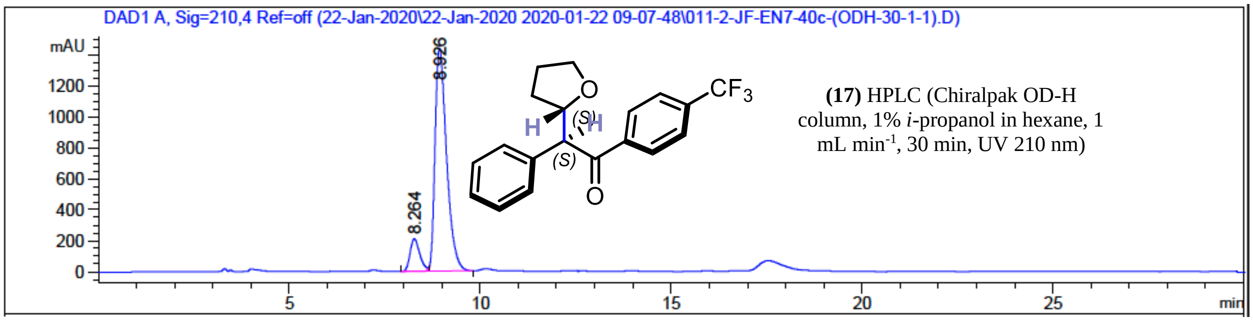

Signal 1: DAD1 A, Sig=210,4 Ref=off

Peak #	RetTime [min]	Type	Width [min]	Area [mAU*s]	Height [mAU]	Area %
1	8.264	BV E	0.2671	3717.57568	209.89127	10.9328
2	8.926	VB R	0.2980	3.02862e4	1439.06470	89.0672

Totals : 3.40038e4 1648.95596

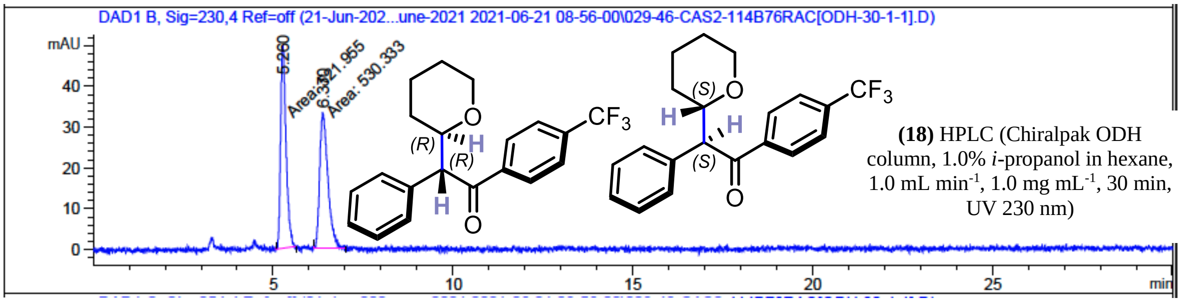

Signal 2: DAD1 B, Sig=230,4 Ref=off

Peak #	RetTime [min]	Type	Width [min]	Area [mAU*s]	Height [mAU]	Area %
1	5.260	MM	0.1745	521.95471	49.86419	49.6019
2	6.370	MM	0.2647	530.33270	33.39367	50.3981

Totals : 1052.28741 83.25787

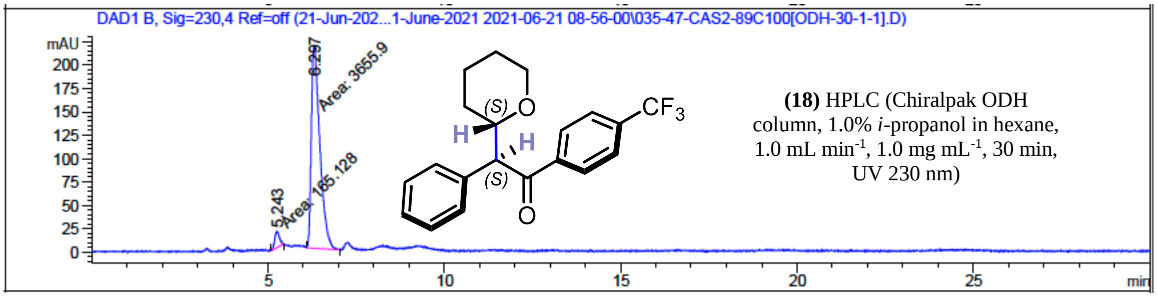

Signal 2: DAD1 B, Sig=230,4 Ref=off

Peak #	RetTime [min]	Type	Width [min]	Area [mAU*s]	Height [mAU]	Area %
1	5.243	MM	0.1578	165.12849	17.43653	4.3216
2	6.297	MM	0.2817	3655.89624	216.27599	95.6784

Totals : 3821.02473 233.71252

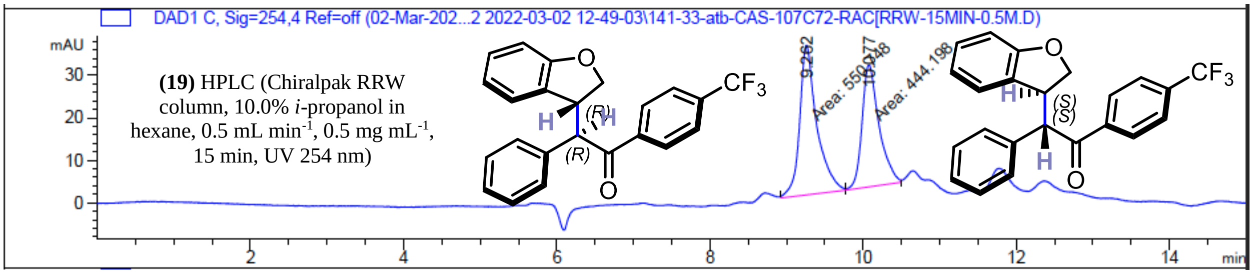

Signal 3: DAD1 C, Sig=254,4 Ref=off

Peak #	RetTime [min]	Type	Width [min]	Area [mAU*s]	Height [mAU]	Area %
1	9.262	MM	0.2603	550.74811	35.25984	55.3546
2	10.077	MM	0.2576	444.19769	28.73804	44.6454

Totals : 994.94580 63.99788

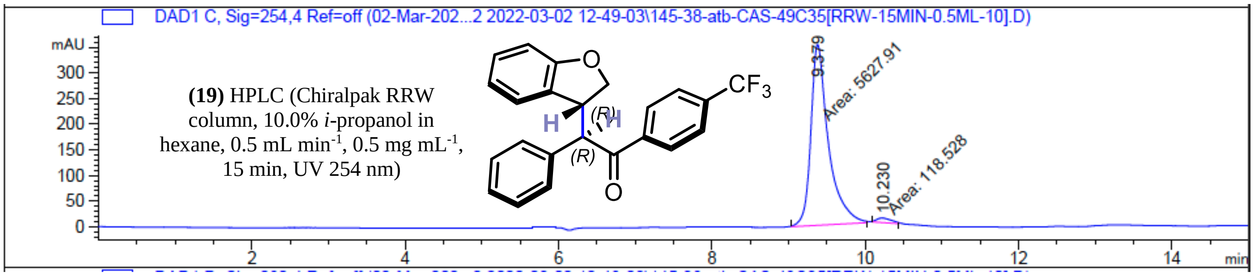

Signal 3: DAD1 C, Sig=254,4 Ref=off

Peak #	RetTime [min]	Type	Width [min]	Area [mAU*s]	Height [mAU]	Area %
1	9.379	MM	0.2666	5627.91016	351.82410	97.9374
2	10.230	MM	0.2215	118.52838	8.91872	2.0626

Totals : 5746.43854 360.74282

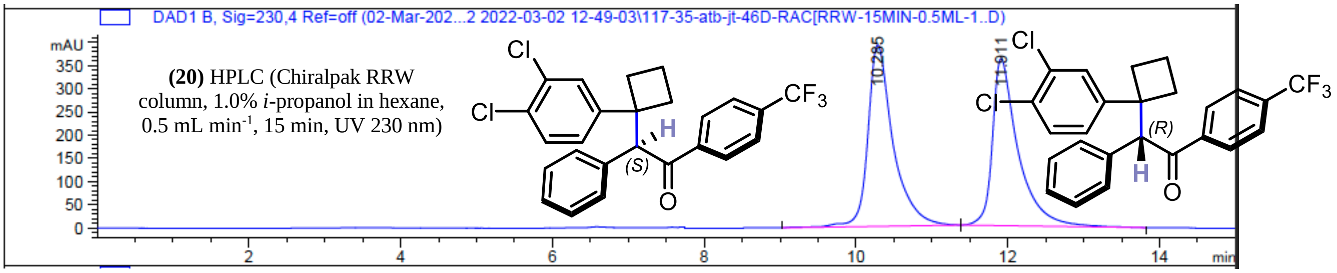

Signal 2: DAD1 B, Sig=230,4 Ref=off

Peak #	RetTime [min]	Type	Width [min]	Area [mAU*s]	Height [mAU]	Area %
1	10.285	BB	0.3330	9042.07227	390.73837	51.6616
2	11.911	BB	0.3379	8460.42480	359.68314	48.3384

Totals : 1.75025e4 750.42151

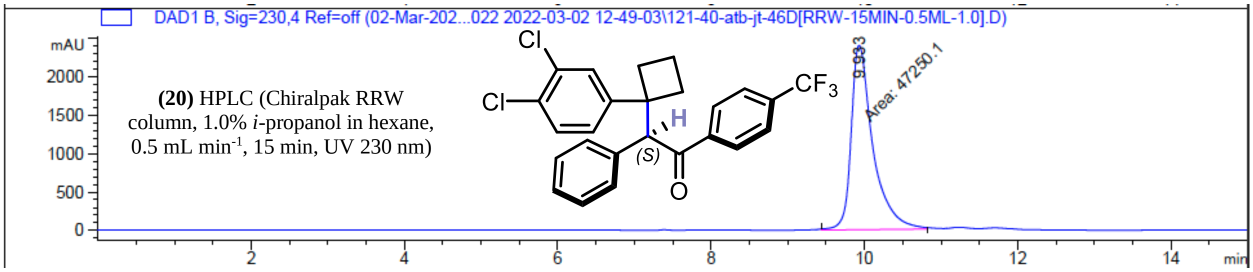

Signal 2: DAD1 B, Sig=230,4 Ref=off

Peak #	RetTime [min]	Type	Width [min]	Area [mAU*s]	Height [mAU]	Area %
1	9.933	MM	0.3282	4.72501e4	2399.23218	100.0000

Totals : 4.72501e4 2399.23218

JF-EN7-40e-rac(newSSW-40-0.5-0.5-210230)24.DATA - Prostar 325 Absorbance Channel 2 LC1006M831

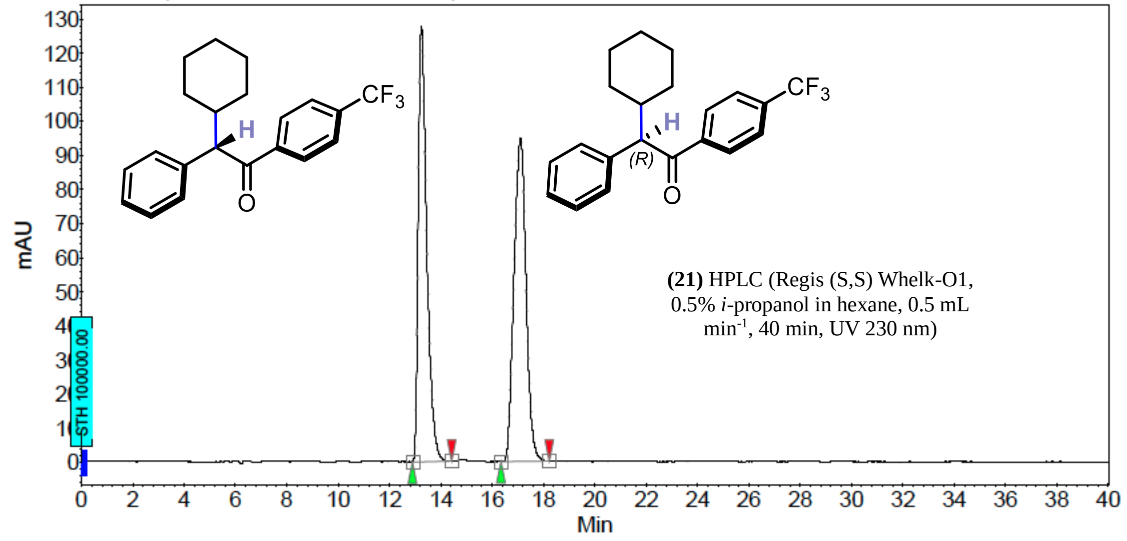

(21) HPLC (Regis (S,S) Whelk-O1, 0.5% *i*-propanol in hexane, 0.5 mL min^{-1}, 40 min, UV 230 nm)

Peak results :

Index	Name	Time [Min]	Quantity [% Area]	Height [mAU]	Area [mAU.Min]	Area % [%]
1	UNKNOWN	13.24	48.93	127.6	49.7	48.929
2	UNKNOWN	17.10	51.07	94.8	51.9	51.071
Total			100.00	222.5	101.6	100.000

JF-EN7-40e-(newSSW-30-0.5-0.5-210230)2.DATA - Prostar 325 Absorbance Channel 2 LC1006M831

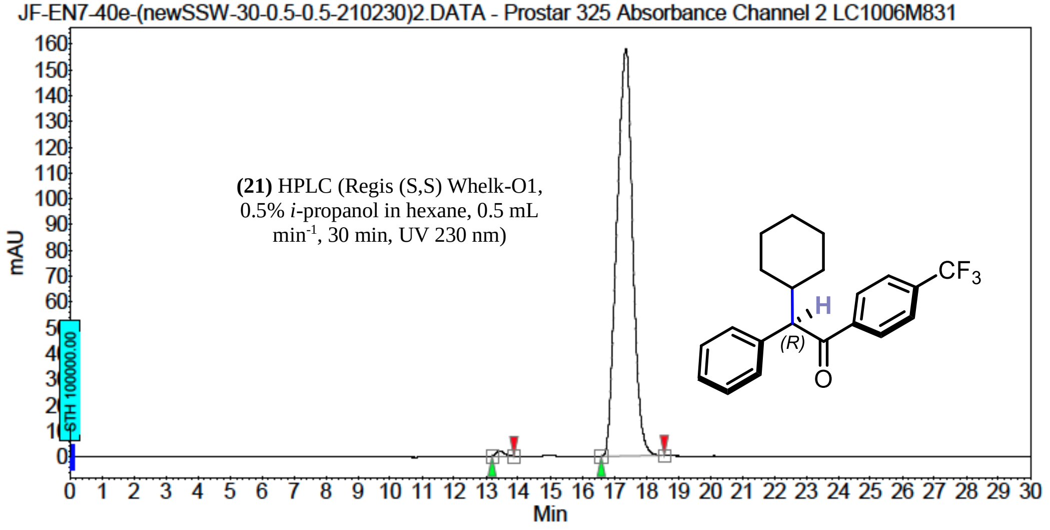

(21) HPLC (Regis (S,S) Whelk-O1, 0.5% *i*-propanol in hexane, 0.5 mL min^{-1}, 30 min, UV 230 nm)

Peak results :

Index	Name	Time [Min]	Quantity [% Area]	Height [mAU]	Area [mAU.Min]	Area % [%]
1	UNKNOWN	13.43	0.69	2.0	0.6	0.688
2	UNKNOWN	17.37	99.31	157.8	90.9	99.312
Total			100.00	159.9	91.5	100.000

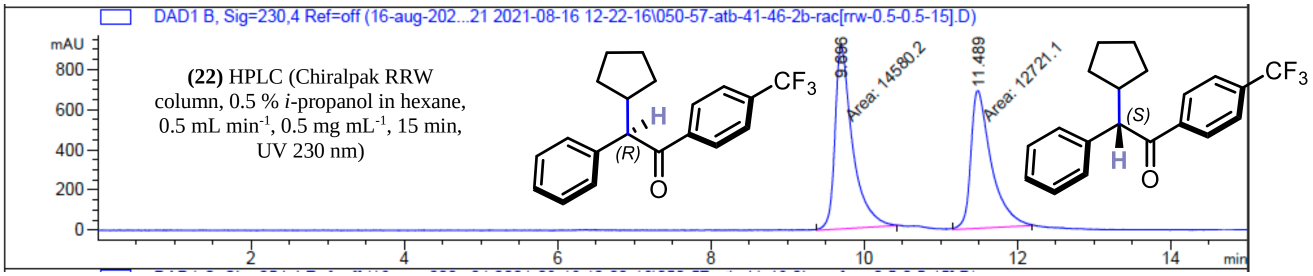

Signal 2: DAD1 B, Sig=230,4 Ref=off

Peak #	RetTime [min]	Type	Width [min]	Area [mAU*s]	Height [mAU]	Area %
1	9.696	MM	0.2651	1.45802e4	916.55933	53.4049
2	11.489	MM	0.3100	1.27211e4	683.90594	46.5951
Totals :				2.73013e4	1600.46527	

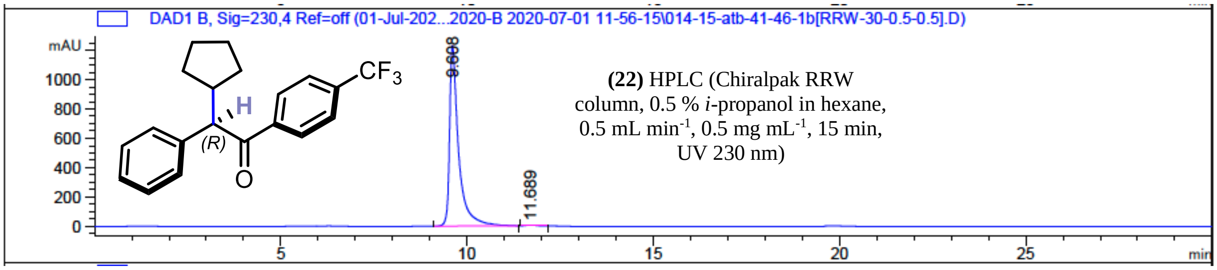

Signal 2: DAD1 B, Sig=230,4 Ref=off

Peak #	RetTime [min]	Type	Width [min]	Area [mAU*s]	Height [mAU]	Area %
1	9.608	VV R	0.2366	2.08042e4	1231.51782	99.5537
2	11.689	VB R	0.1912	93.26826	5.78229	0.4463
Totals :				2.08975e4	1237.30011	

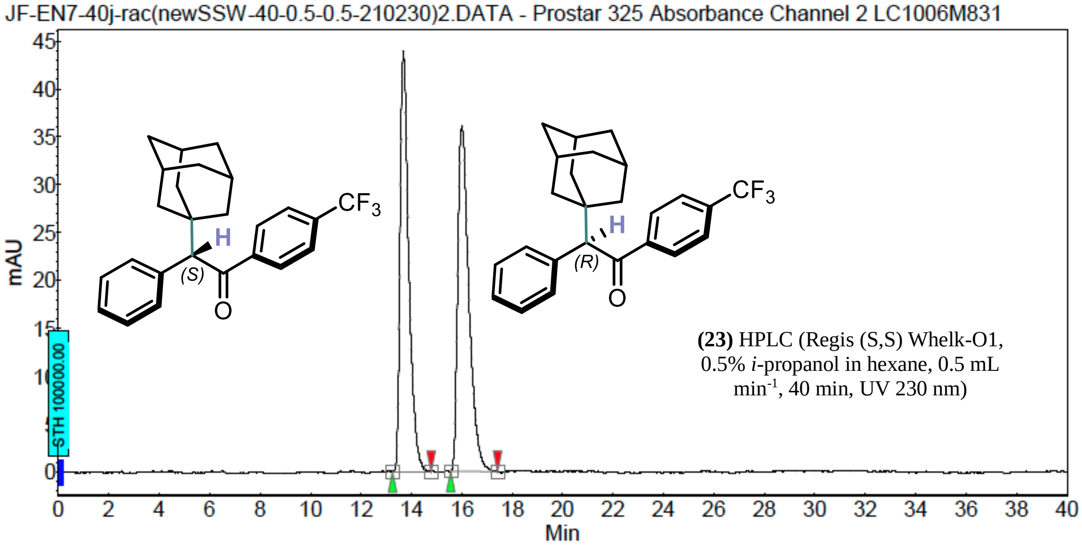

Peak results :

Index	Name	Time [Min]	Quantity [% Area]	Height [mAU]	Area [mAU.Min]	Area % [%]
1	UNKNOWN	13.68	48.76	44.0	17.6	48.761
2	UNKNOWN	16.00	51.24	36.1	18.5	51.239
Total			100.00	80.0	36.1	100.000

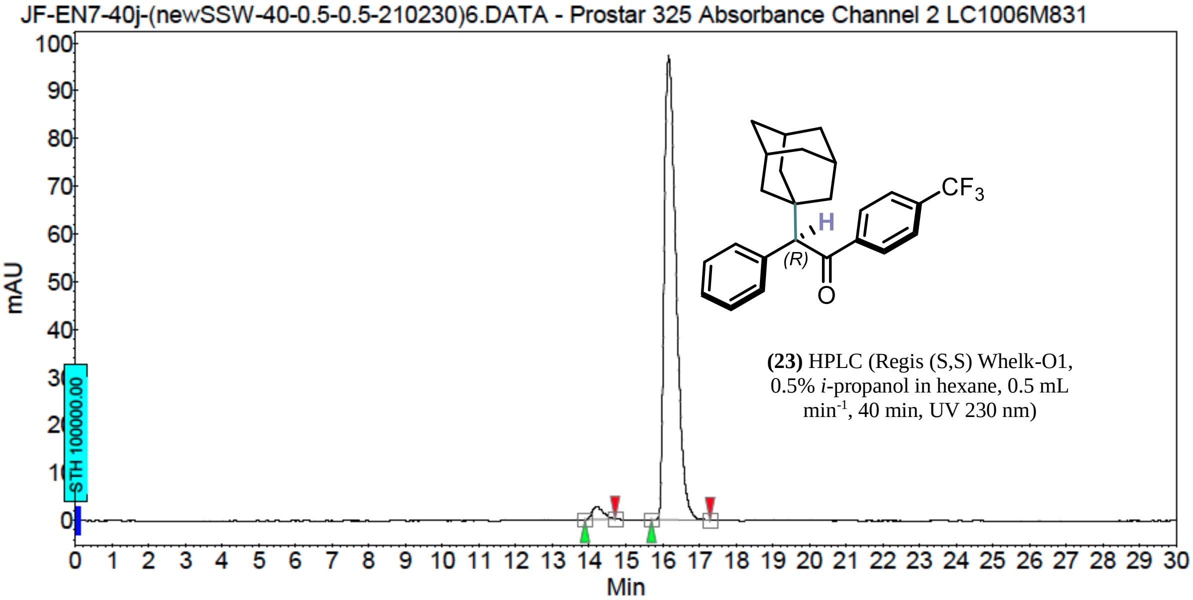

(23) HPLC (Regis (S,S) Whelk-O1, 0.5% *i*-propanol in hexane, 0.5 mL min^{-1}, 40 min, UV 230 nm)

Peak results :

Index	Name	Time [Min]	Quantity [% Area]	Height [mAU]	Area [mAU.Min]	Area % [%]
1	UNKNOWN	14.20	2.62	2.8	1.0	2.622
2	UNKNOWN	16.16	97.38	97.4	36.2	97.378
Total			100.00	100.2	37.2	100.000

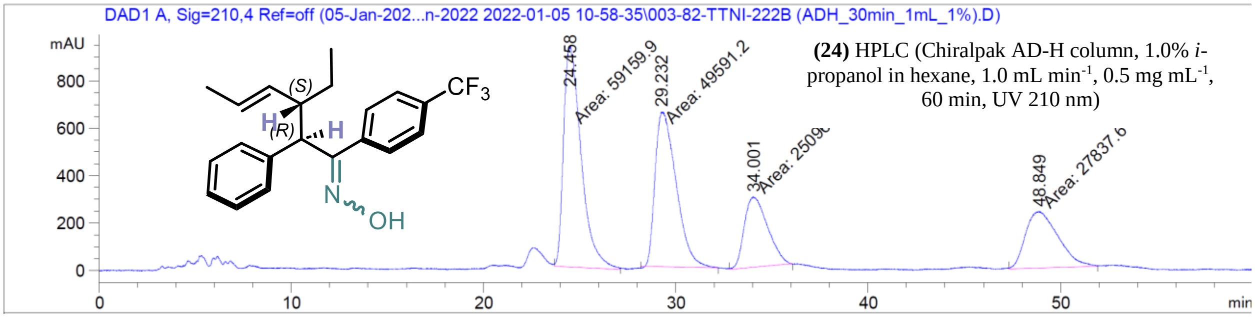

(24) HPLC (Chiralpak AD-H column, 1.0% *i*-propanol in hexane, 1.0 mL min^{-1}, 0.5 mg mL^{-1}, 60 min, UV 210 nm)

Signal 1: DAD1 A, Sig=210,4 Ref=off

Peak #	RetTime [min]	Type	Width [min]	Area [mAU*s]	Height [mAU]	Area %
1	24.458	MM	1.0565	5.91599e4	933.24683	36.5895
2	29.232	MM	1.2630	4.95912e4	654.43195	30.6714
3	34.001	MM	1.4017	2.50965e4	298.39767	15.5219
4	48.849	MM	1.9503	2.78376e4	237.89186	17.2172

Totals : 1.61685e5 2123.96831

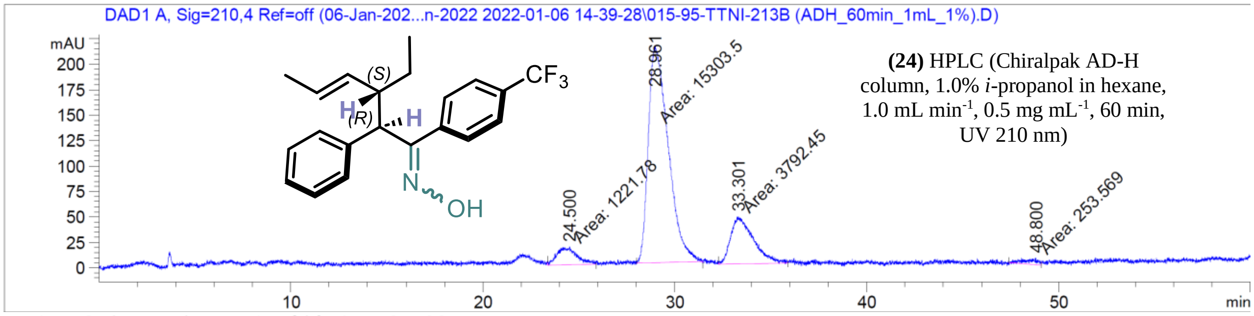

Signal 1: DAD1 A, Sig=210,4 Ref=off

Peak #	RetTime [min]	Type	Width [min]	Area [mAU*s]	Height [mAU]	Area %
1	24.500	MM	1.1439	1221.77625	17.80182	5.9392
2	28.961	MM	1.1963	1.53035e4	213.20369	74.3925
3	33.301	MM	1.3550	3792.44507	46.64852	18.4356
4	48.800	MM	0.6168	253.56923	6.85119	1.2326

Totals : 2.05713e4 284.50522

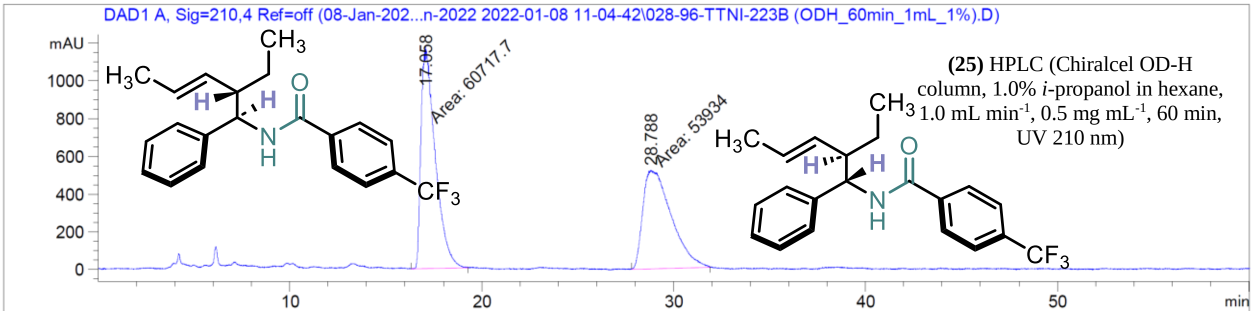

Signal 1: DAD1 A, Sig=210,4 Ref=off

Peak #	RetTime [min]	Type	Width [min]	Area [mAU*s]	Height [mAU]	Area %
1	17.058	MM	0.8550	6.07177e4	1183.63135	52.9584
2	28.788	MM	1.7119	5.39340e4	525.10101	47.0416

Totals : 1.14652e5 1708.73236

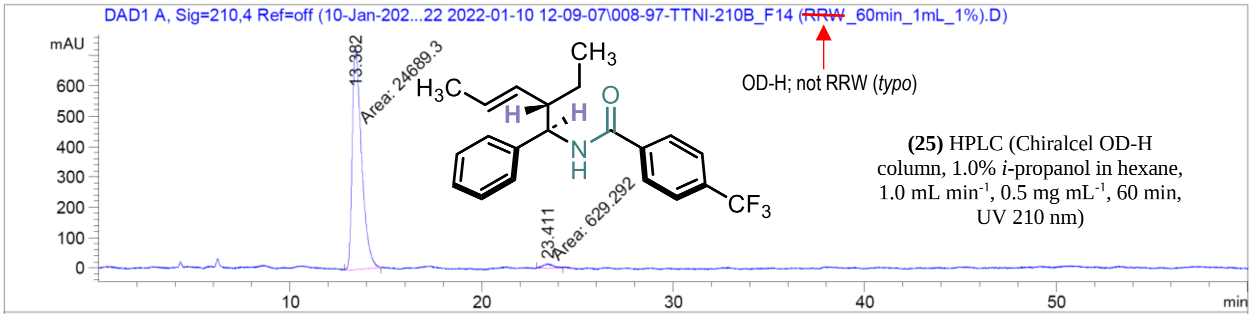

Signal 1: DAD1 A, Sig=210,4 Ref=off

Peak #	RetTime [min]	Type	Width [min]	Area [mAU*s]	Height [mAU]	Area %
1	13.382	MM	0.5579	2.46893e4	737.56012	97.5145
2	23.411	MM	0.6360	629.29224	16.49015	2.4855

Totals : 2.53186e4 754.05027

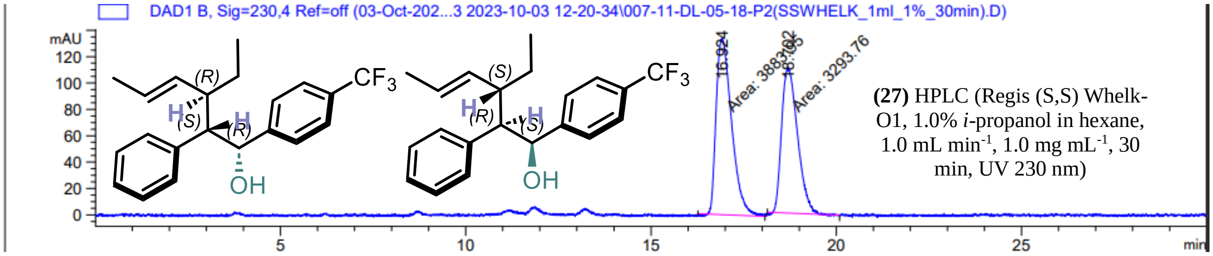

Signal 2: DAD1 B, Sig=230,4 Ref=off

Peak #	RetTime [min]	Type	Width [min]	Area [mAU*s]	Height [mAU]	Area %
1	16.924	MM	0.4840	3883.94604	133.75598	54.1112
2	18.702	MM	0.4978	3293.75977	110.27296	45.8888

Totals : 7177.70581 244.02894

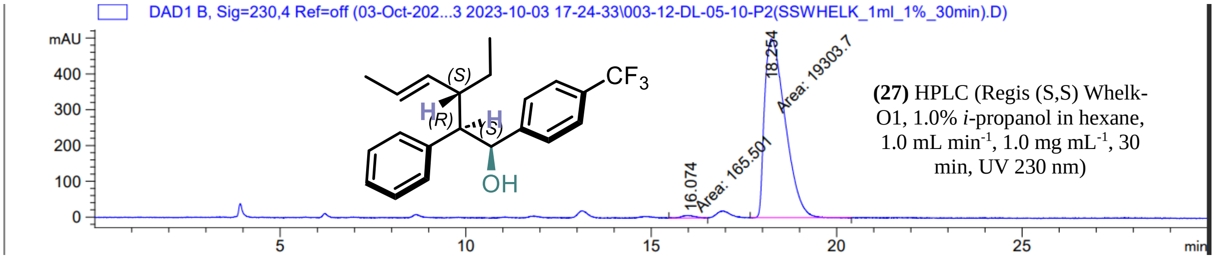

Signal 2: DAD1 B, Sig=230,4 Ref=off

Peak #	RetTime [min]	Type	Width [min]	Area [mAU*s]	Height [mAU]	Area %
1	16.074	MM	0.3838	165.50139	7.18701	0.8501
2	18.254	MM	0.6439	1.93037e4	499.69135	99.1499

Totals : 1.94692e4 506.87836

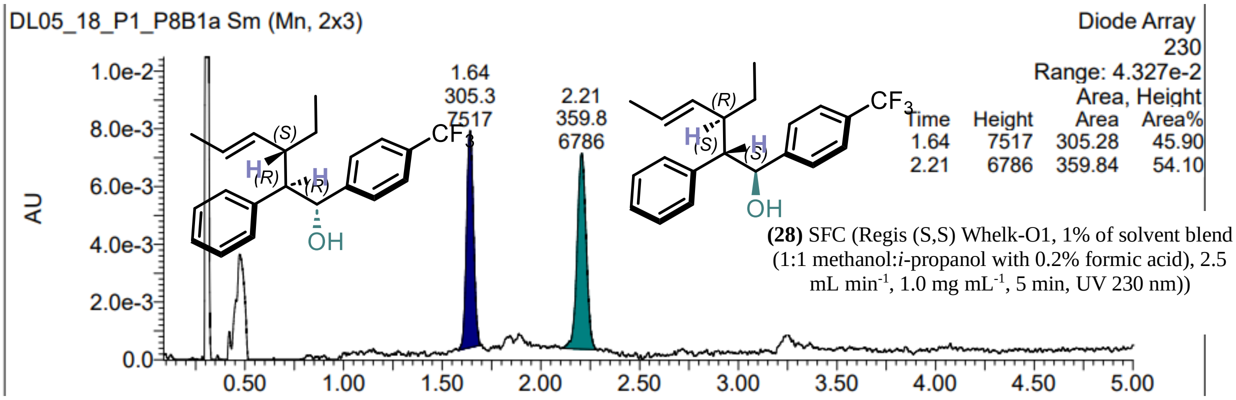

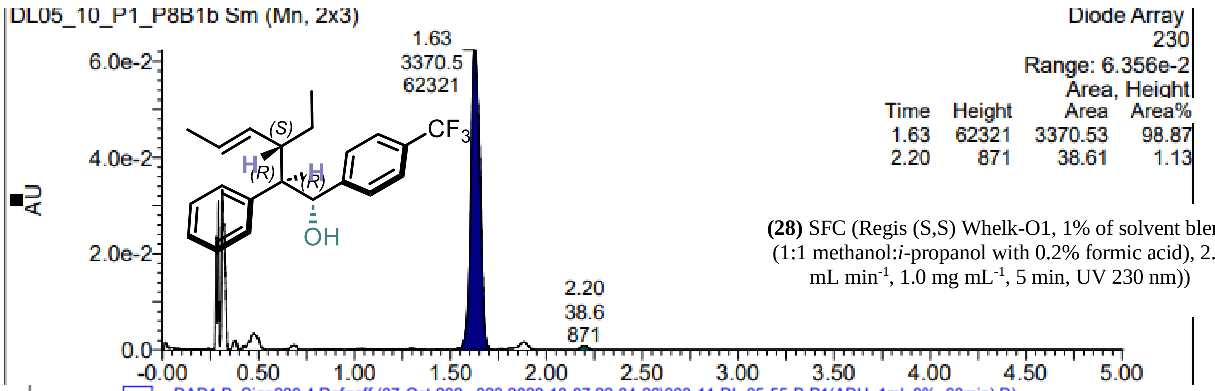

(28) SFC (Regis (S,S) Whelk-O1, 1% of solvent blend (1:1 methanol:*i*-propanol with 0.2% formic acid), 2.5 mL min^{-1}, 1.0 mg mL^{-1}, 5 min, UV 230 nm))

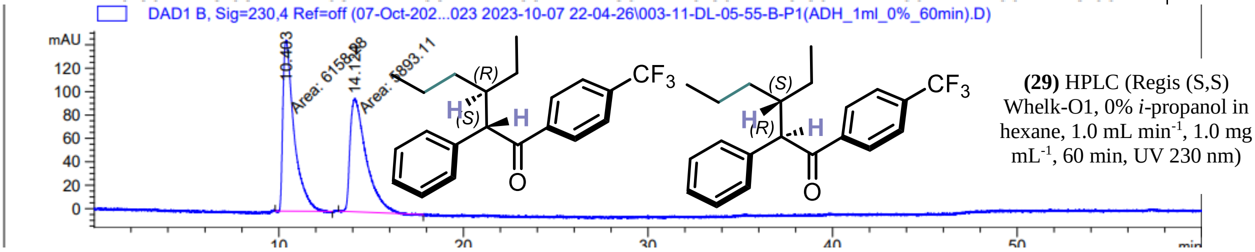

(29) HPLC (Regis (S,S) Whelk-O1, 0% *i*-propanol in hexane, 1.0 mL min^{-1}, 1.0 mg mL^{-1}, 60 min, UV 230 nm)

Signal 2: DAD1 B, Sig=230,4 Ref=off

Peak #	RetTime [min]	Type	Width [min]	Area [mAU*s]	Height [mAU]	Area %
1	10.403	MM	0.7022	6158.27783	146.17085	51.1001
2	14.124	MM	1.0135	5893.11426	96.90829	48.8999

Totals : 1.20514e4 243.07915

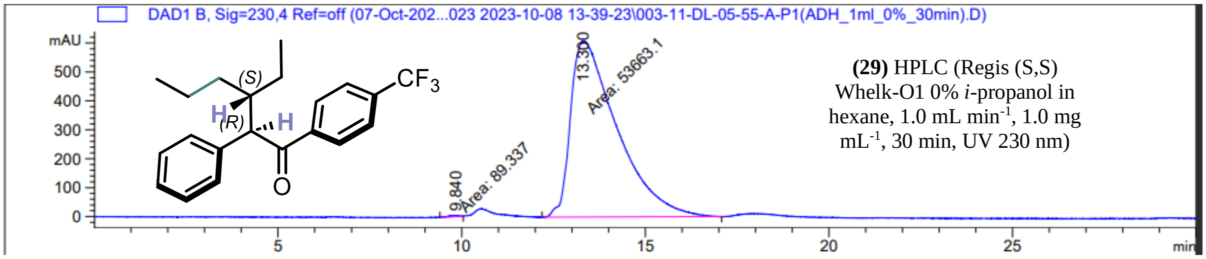

(29) HPLC (Regis (S,S) Whelk-O1 0% *i*-propanol in hexane, 1.0 mL min^{-1}, 1.0 mg mL^{-1}, 30 min, UV 230 nm)

Signal 2: DAD1 B, Sig=230,4 Ref=off

Peak #	RetTime [min]	Type	Width [min]	Area [mAU*s]	Height [mAU]	Area %
1	9.840	MM	0.3624	89.33697	4.10897	0.1662
2	13.300	MM	1.4677	5.36631e4	609.37885	99.8338

Totals : 5.37525e4 613.48781

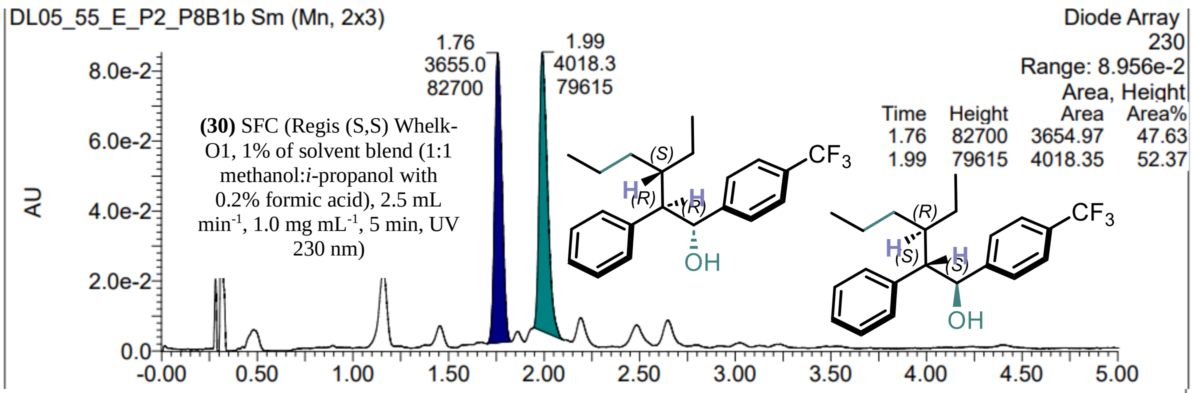

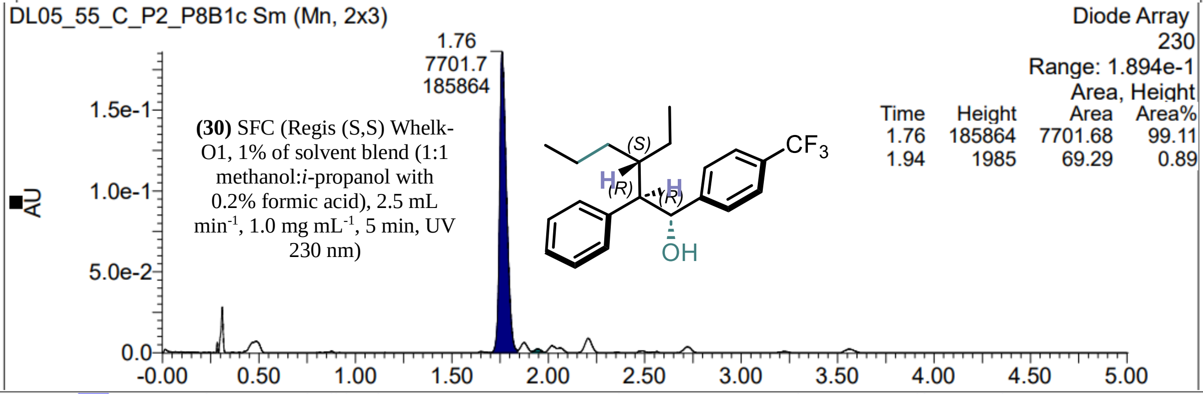

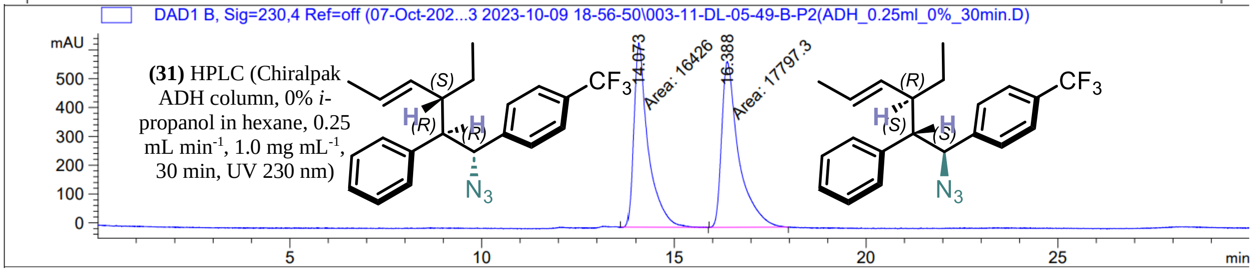

Signal 2: DAD1 B, Sig=230,4 Ref=off

Peak #	RetTime [min]	Type	Width [min]	Area [mAU*s]	Height [mAU]	Area %
1	14.073	MM	0.4276	1.64260e4	640.26764	47.9965
2	16.388	MM	0.5156	1.77973e4	575.31854	52.0035

Totals : 3.42232e4 1215.58618

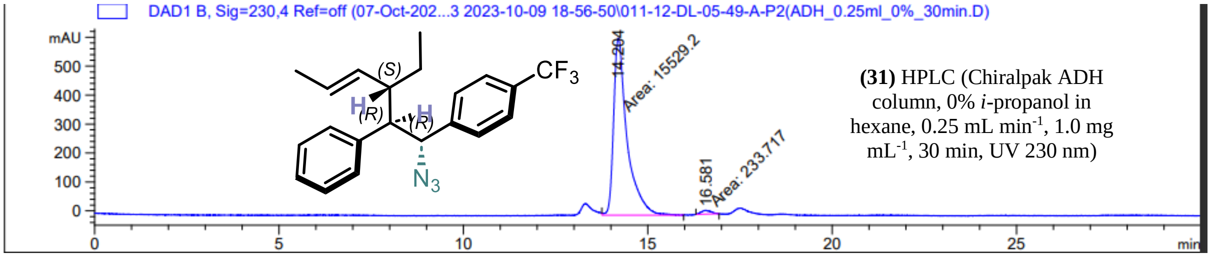

Signal 2: DAD1 B, Sig=230,4 Ref=off

Peak #	RetTime [min]	Type	Width [min]	Area [mAU*s]	Height [mAU]	Area %
1	14.204	MM	0.4224	1.55292e4	612.78015	98.5173
2	16.581	MM	0.3018	233.71716	12.90830	1.4827

Totals : 1.57629e4 625.68845

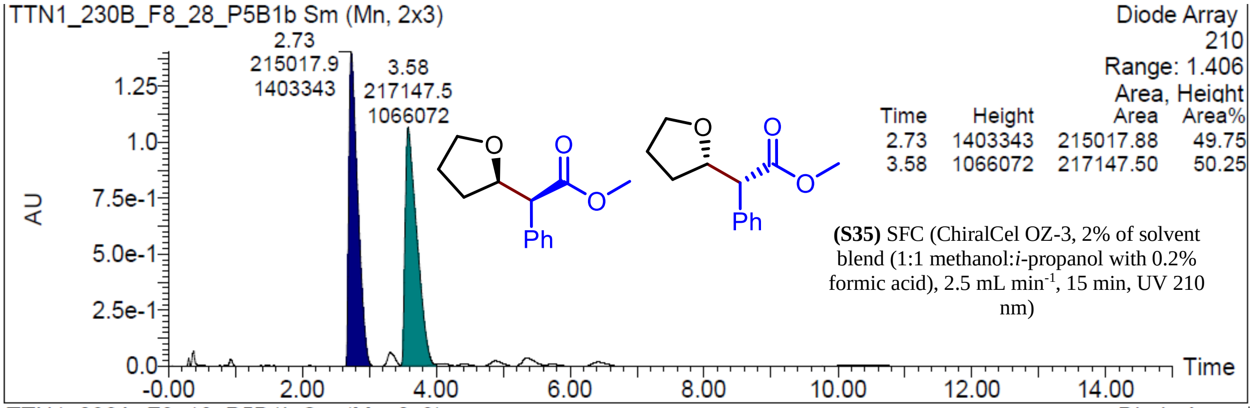

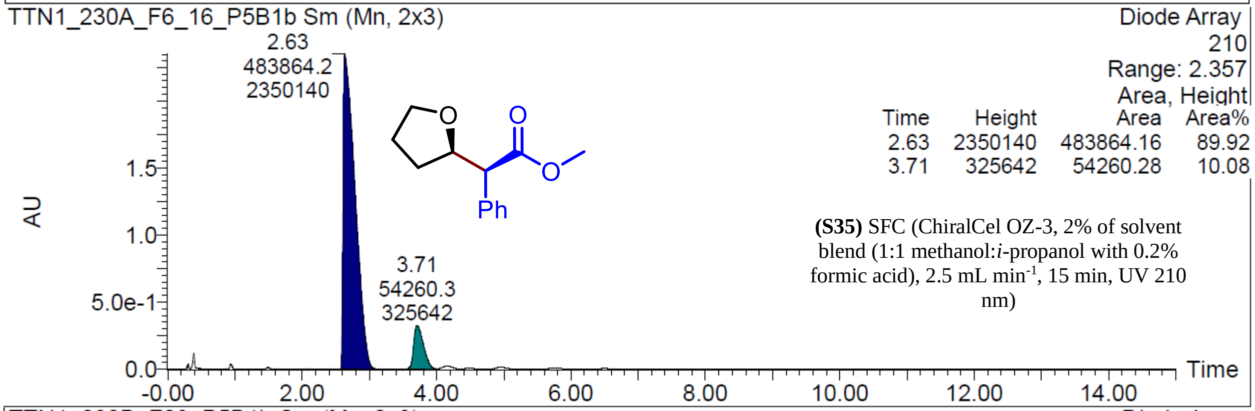

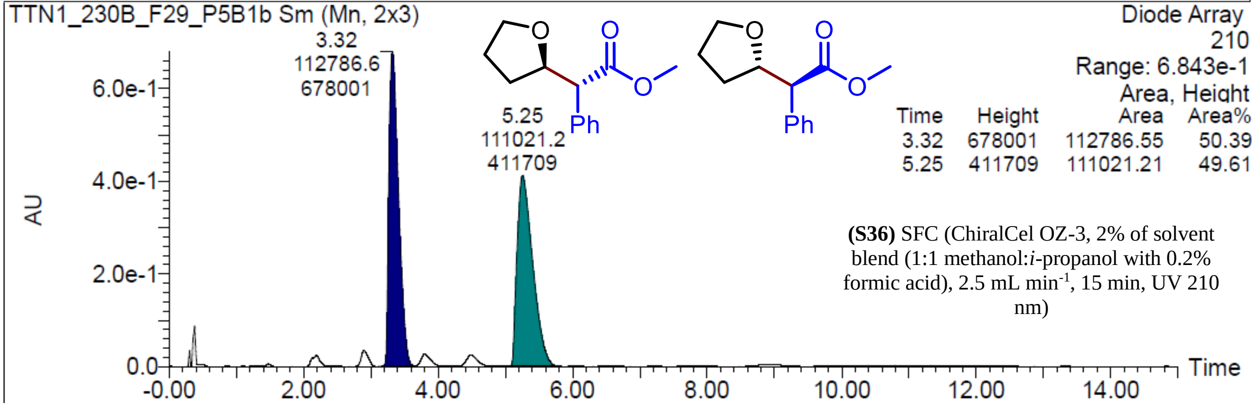

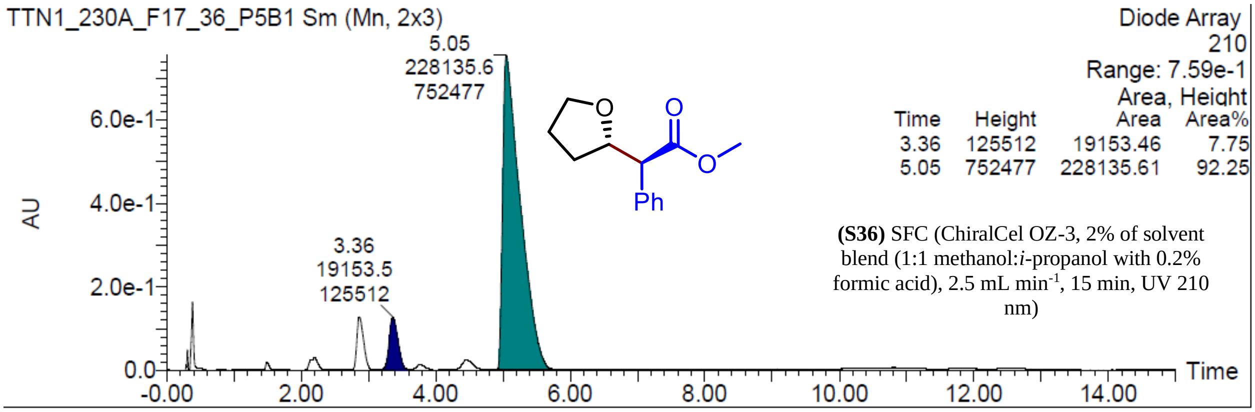

(S36) SFC (ChiralCel OZ-3, 2% of solvent blend (1:1 methanol:*i*-propanol with 0.2% formic acid), 2.5 mL min^{-1}, 15 min, UV 210 nm)

8. Determination of Absolute Configuration and X-ray data

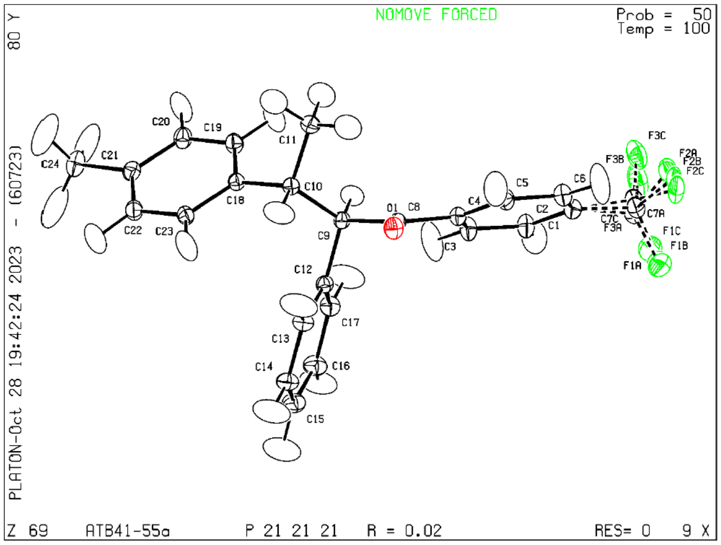

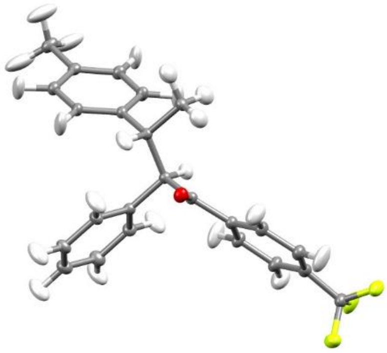

**Compound**	(2R,3R)-1-phenyl-3-(p-tolyl)-2-(4-(trifluoromethyl)phenyl)butan-1-one **(2d)**
Formula	$C_{24}H_{21}F_{3}O$
$D_{calc.}$/ g cm^{-3}	1.338
m/mm^{-1}	0.836
Formula Weight	382.41
Colour	colorless
Shape	needle-shaped
Size/mm^3	0.63×0.21×0.17
T/K	100(1)
Crystal System	orthorhombic
Flack Parameter	-0.05(6)
Hooft Parameter	-0.03(5)
Space Group	$P2_12_12_1$
a/Å	5.95673(5)
b/Å	15.28051(16)
c/Å	20.8537(2)
a/°	90
b/°	90
g/°	90
V/Å^3	1898.14(3)
Z	4
Z'	1
Wavelength/Å	1.54184
Radiation type	Cu K_a
Q_{min}/°	3.586
Q_{max}/°	77.254
Measured Refl's.	44177
Indep't Refl's	4000
Refl's I≥2 *s*(I)	3902
R_{int}	0.0675
Parameters	382
Restraints	428
Largest Peak	0.221
Deepest Hole	-0.148
GooF	1.038
wR_2 (all data)	0.0853
wR_2	0.0846
R_1 (all data)	0.0342
R_1	0.0333

**Compound**	(2R,3R)-2-(4-chlorophenyl)-1-phenyl-3-(p-tolyl)butan-1-one **(2k)**
Empirical formula	$C_{23}H_{21}ClO$
Formula weight	348.875
Temperature [K]	100.00(10)
Crystal system	orthorhombic
Space group (number)	$P2_12_12_1$ (19)
a [Å]	5.9233(5)
b [Å]	17.3606(12)
c [Å]	17.3654(14)
α [°]	90
β [°]	90
γ [°]	90
Volume [Å^3]	1785.7(2)
Z	4
ρ_{calc} [gcm^{-3}]	1.298
μ [mm^{-1}]	1.932
F(000)	739.506
Crystal size [mm^3]	0.031×0.05×0.484
Crystal colour	colourless
Crystal shape	needle
Radiation	Cu K_α (λ=1.54184 Å)
2θ range [°]	7.20 to 142.70 (0.81 Å)
Index ranges	$-7 \le h \le 7$<br>$-21 \le k \le 21$<br>$-21 \le l \le 21$
Reflections collected	20632
Independent reflections	3401<br>R_{int} = 0.0824<br>R_{sigma} = 0.0623
Completeness to θ = 67.6840°	99.7 %
Data / Restraints / Parameters	3401 / 393 / 415
Goodness-of-fit on F^2	1.0357
Final *R* indexes [$I \ge 2\sigma(I)$]	R_1 = 0.0512<br>wR_2 = 0.1074
Final *R* indexes [all data]	R_1 = 0.0671<br>wR_2 = 0.1162
Largest peak/hole [eÅ^{-3}]	0.28/−0.30
Flack X parameter	0.013(13)

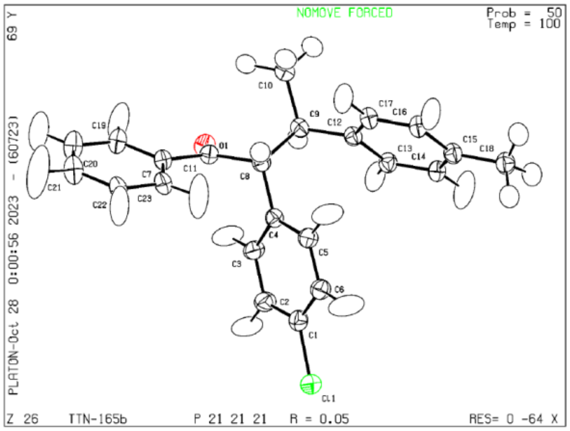

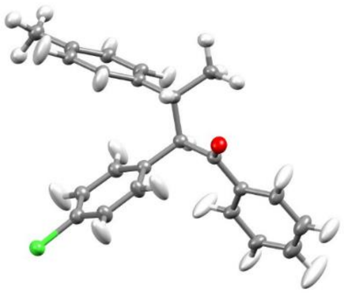

**Compound**	(2R,3R)-2-(6-chloropyridin-3-yl)-1-phenyl-3-(p-tolyl)butan-1-one **(2s)**	
Formula	$C_{22}H_{20}ClNO$	
$D_{calc.}$/ g cm^{-3}	1.291	
m/mm^{-1}	0.221	
Formula Weight	349.863	
Colour	colorless	
Shape	plate-shaped	
Size/mm^3	0.62×0.27×0.09	
T/K	100.0(5)	
Crystal System	orthorhombic	
Flack Parameter	0.021(19)	
Hooft Parameter	0.021(19)	
Space Group	$P2_12_12_1$	
a/Å	5.9620(2)	
b/Å	17.1460(6)	
c/Å	17.6127(8)	
a/°	90	
b/°	90	
g/°	90	
V/Å^3	1800.45(12)	
Z	4	
Z'	1	
Wavelength/Å	0.71073	
Radiation type	Mo K_a	
Q_{min}/°	2.38	
Q_{max}/°	33.13	
Measured Refl's.	34984	
Indep't Refl's	6746	
Refl's I≥2 *s*(I)	6049	
R_{int}	0.0580	
Parameters	406	
Restraints	378	
Largest Peak	0.2446	
Deepest Hole	-0.1799	
GooF	0.9902	
wR_2 (all data)	0.0552	
wR_2	0.0540	
R_1 (all data)	0.0328	
R_1	0.0277	

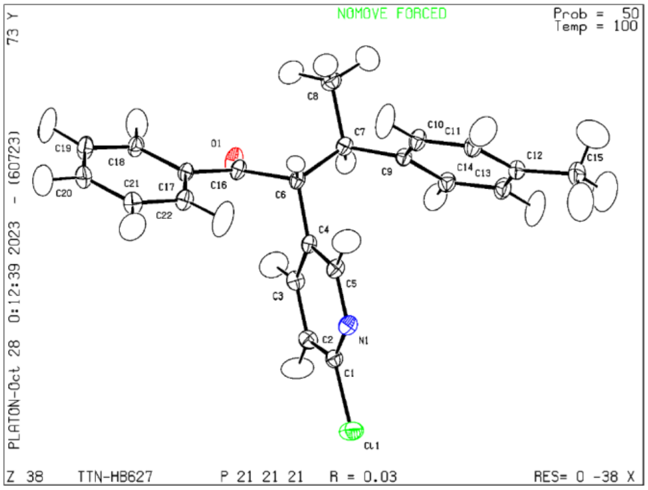

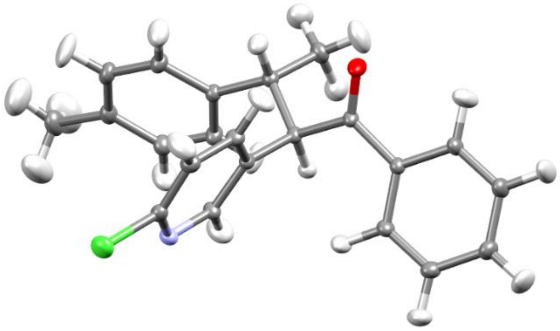

**Compound**	N-((1R,2S,E)-2-ethyl-1-phenylpent-3-en-1-yl)-4-(trifluoromethyl)benzamide **(25)**
Formula	$C_{22}H_{23}Cl_3F_3NO$
$D_{calc.}$/ g cm^{-3}	1.417
m/mm^{-1}	4.025
Formula Weight	480.76
Colour	colourless
Shape	needle-shaped
Size/mm^3	0.45×0.12×0.06
T/K	101(1)
Crystal System	monoclinic
Flack Parameter	0.040(15)
Hooft Parameter	0.040(15)
Space Group	$P2_1$
a/Å	20.4751(5)
b/Å	5.18050(10)
c/Å	21.5775(6)
a/°	90
b/°	99.971(2)
g/°	90
V/Å^3	2254.18(10)
Z	4
Z'	2
Wavelength/Å	1.54184
Radiation type	Cu K_a
Q_{min}/°	2.747
Q_{max}/°	68.244
Measured Refl's.	34235
Indep't Refl's	8121
Refl's I≥2 *s*(I)	7136
R_{int}	0.0526
Parameters	662
Restraints	601
Largest Peak	0.813
Deepest Hole	-0.786
GooF	1.060
wR_2 (all data)	0.2512
wR_2	0.2418
R_1 (all data)	0.0988
R_1	0.0907

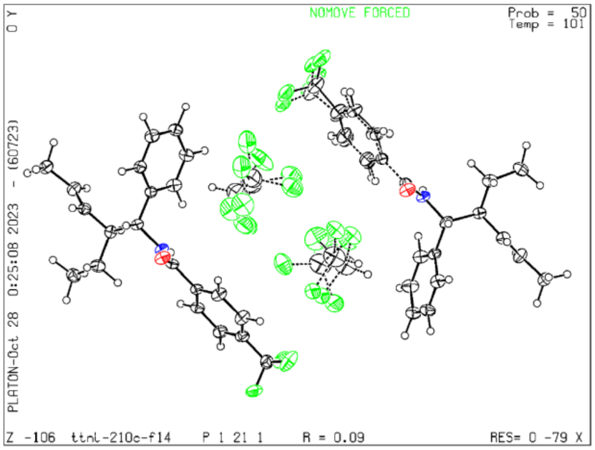

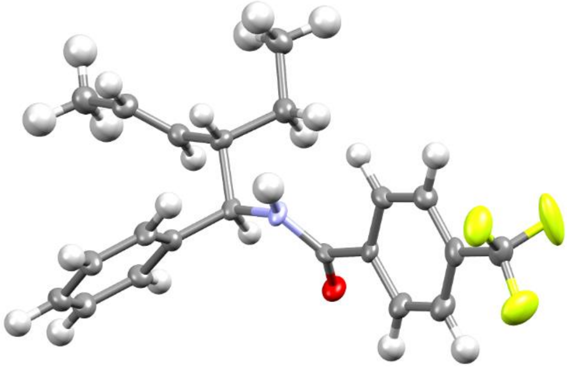

9. Computational Procedure

Geometry optimizations and frequency calculations for all reported structures were performed with the Gaussian 16 suite of programs[1] at the B3LYP-D3(BJ) level of theory with the corresponding Hay-Wadt effective core potential for Rh, and Grimme's empirical dispersion-correction (D3) with Becke-Johnson (BJ) damping for B3LYP.[2-5] In these calculations we used the [6-31G(d,p) + Lanl2dz (Rh)] basis sets. Bulk solvent

effects are incorporated for all calculations using the self-consistent reaction field polarizable continuum model (IEF-PCM).[6] As a solvent we chose dichloromethane. The Gibbs free energies are calculated at 298.15 K temperature and 1 atm. pressure. Previously, it was shown that the used [B3LYP-D3(BJ) + PCM]/BS1 approach describes appropriately the geometries of organic and organometallic species.[7]

Important pre-reaction complexes and transition states are given in Figure S1. In Figure S2 we have presented the free energy surface of the carbene insertion into the C–H bonds of the cyclohexane. The cartesian coordinates of all computed structures are included in the Table S1 of the Supporting Information.

1. Gaussian 16, Revision A.03, M. J. Frisch, G. W. Trucks, H. B. Schlegel, G. E. Scuseria, M. A. Robb, J. R. Cheeseman, G. Scalmani, V. Barone, G. A. Petersson, H. Nakatsuji, X. Li, M. Caricato, A. Marenich, J. Bloino, B. G. Janesko, R. Gomperts, B. Mennucci, H. P. Hratchian, J. V. Ortiz, A. F. Izmaylov, J. L. Sonnenberg, D. Williams-Young, F. Ding, F. Lipparini, F. Egidi, J. Goings, B. Peng, A. Petrone, T. Henderson, D. Ranasinghe, V. G. Zakrzewski, J. Gao, N. Rega, G. Zheng, W. Liang, M. Hada, M. Ehara, K. Toyota, R. Fukuda, J. Hasegawa, M. Ishida, T. Nakajima, Y. Honda, O. Kitao, H. Nakai, T. Vreven, K. Throssell, J. A. Montgomery, Jr., J. E. Peralta, F. Ogliaro, M. Bearpark, J. J. Heyd, E. Brothers, K. N. Kudin, V. N. Staroverov, T. Keith, R. Kobayashi, J. Normand, K. Raghavachari, A. Rendell, J. C. Burant, S. S. Iyengar, J. Tomasi, M. Cossi, J. M. Millam, M. Klene, C. Adamo, R. Cammi, J. W. Ochterski, R. L. Martin, K. Morokuma, O. Farkas, J. B. Foresman, and D. J. Fox, *Gaussian, Inc., Wallingford CT*, **2016**.
2. (a) Hay, P. J.; Wadt, W. R. Ab Initio Effective Core Potentials for Molecular Calculations. Potentials for the Transition Metal Atoms Sc to Hg. *J. Chem. Phys.* **1985**, *82*, 270-283. (b) Hay, P. J.; Wadt, W. R. Ab Initio Effective Core Potentials for Molecular Calculations. Potentials for K to Au Including the Outermost Core Orbitals. *J. Chem. Phys.* **1985**, *82*, 299-310. (c) Wadt, W. R.; Hay, P. J. Ab Initio Effective Core Potentials for Molecular Calculations. Potentials for Main Group Elements Na to Bi. *J. Chem. Phys*. **1985**, *82*, 284-298.
3. (a) Becke, A. D. Density-Functional Exchange-Energy Approximation with Correct Asymptotic Behavior. *Phys. Rev. A* **1988,** *38*, 3098-3100. (b) Lee, C.; Yang, W.; Parr, R. G. Development of The Colle-Salvetti Correlation-Energy Formula into a Functional of the Electron Density. *Phys. Rev. B* **1988,** *37*, 785-789. (c) Becke, A. D. A New Mixing of Hartree–Fock and Local Density-Functional Theories. *J. Chem. Phys.* **1993,** *98*, 1372-1377.
4. Grimme, S., Hansen, A., Brandenburg, J. G. & Bannwarth, C. Dispersion-Corrected Mean-Field Electronic Structure Methods. *Chem. Rev.* **116**, 5105-5154 (2016).
5. (a) Grimme, S.; Antony, J.; Ehrlich, S.; Krieg, H. A Consistent and Accurate Ab Initio Parametrization of Density Functional Dispersion Correction (DFT-D) for the 94 Elements H-Pu. *J. Chem. Phys.* **2010,** *132*, 154104-154122. (b) Becke, A. D.; Johnson, E. R. A Density-Functional Model of the Dispersion Interaction. *J. Chem. Phys.* **2005,** *123*, 154101-154106. (c) Becke, A. D.; Johnson, E. R. Exchange-Hole Dipole Moment and the Dispersion Interaction. *J. Chem. Phys.* **2005,** *122*, 154104-154109. (d) Johnson, E. R.; Becke, A. D. A Post-Hartree-Fock Model of Intermolecular Interactions: Inclusion of Higher-Order Corrections. *J. Chem. Phys.* **2006,** *124*, 174104-174112.
6. (a) Barone, V.; Cossi, M. Quantum Calculation of Molecular Energies and Energy Gradients in Solution by a Conductor Solvent Model. *J. Phys. Chem. A* **1998,** *102*, 1995-2001. (b) Cossi, M.; Rega, N.; Scalmani, G.; Barone, V. Energies, structures, and electronic properties of molecules in solution with the C-PCM solvation model. *J. Comput. Chem.* **2003,** *24*, 669-681.
7. Musaev, D. G.; Figg, T. M.; Kaledin, A. L. Versatile reactivity of Pd-catalysts: mechanistic features of the mono-N-protected amino acid ligand and cesium-halide base in Pd-catalyzed C–H bond functionalization. *Chem. Soc. Rev.* **2014**, *43,* 5009-5031.

Table S1. Cartesian Coordinates of all calculated structures

Cyclo-Hexane, chair

C	-4.28811700	-0.12057400	-0.00783700
C	-2.75272500	-0.12133700	-0.00841200
C	-2.19352500	1.30856400	-0.00679900
C	-2.75130800	2.12512500	1.16771900
C	-4.28669900	2.12590900	1.16828900
C	-4.84592300	0.69601500	1.16665300
H	-1.09781600	1.28886700	0.02949500
H	-2.39379700	-0.64804100	0.88725000
H	-2.37257800	-0.67793400	-0.87326400
H	-4.64702200	0.31590500	-0.95077900
H	-4.66945800	-1.14799900	0.02760700
H	-2.39241200	1.68860000	2.11064500
H	-2.36994800	3.15254400	1.13232200
H	-4.66684500	2.68249200	2.03315200
H	-4.64561700	2.65263500	0.27263600
H	-4.57120200	0.20218700	2.10958300
H	-5.94163100	0.71574200	1.13030100
H	-2.46830500	1.80239200	-0.94971300

N_2–[PhCOPh], diazoarylketone

C	0.08890100	2.23114400	0.16462300
C	-1.12575000	1.39380400	0.28515300
C	-2.15374500	1.74308900	1.17411800
C	-1.26933900	0.24403100	-0.50838600
C	-3.30009700	0.95514500	1.27105000
H	-2.05034500	2.62712700	1.79620100
C	-2.40750500	-0.55048600	-0.39307200
H	-0.48693700	-0.02515900	-1.20909700
C	-3.42790000	-0.19766400	0.49398600
H	-4.08759800	1.23700400	1.96299800
H	-2.50302200	-1.44041600	-1.00736400
C	1.49041300	1.82385200	-0.03014300
O	2.33365200	2.62711800	-0.43755400
H	-4.31645200	-0.81551800	0.57656400
N	-0.08276700	3.53811200	0.12617500
N	-0.19148800	4.66839200	0.10492200
C	1.87125000	0.41643600	0.29541400
C	2.90000300	-0.17293200	-0.45300800
C	1.29552800	-0.29319500	1.35934100
C	3.32671200	-1.46653000	-0.16286700
H	3.35073900	0.39507400	-1.25957700
C	1.73917500	-1.57935300	1.66132400
H	0.51435500	0.16288600	1.95618300

C	2.74659200	-2.17142700	0.89551900
H	4.11261300	-1.92354200	-0.75592600
H	1.29850700	-2.11967700	2.49312100
H	3.08193900	-3.17803300	1.12566700

$Rh_2(OAc)_4$

Rh	0.01306100	0.00304900	-1.18804600
O	-1.43504900	1.47366900	-1.13355400
O	1.48272300	1.45014900	-1.10704000
O	1.45997600	-1.46730100	-1.11362900
O	-1.45781300	-1.44517100	-1.14014300
Rh	-0.01270400	-0.00349700	1.20621500
C	-1.89076900	-1.85102700	-0.01302300
O	-1.48189200	-1.45108000	1.12521400
C	1.85057800	-1.89165500	0.02225200
O	1.43588500	-1.47364700	1.15173000
C	-1.85022100	1.89120600	-0.00407900
O	-1.46009600	1.46638100	1.13179200
O	1.45770400	1.44519400	1.15830900
C	1.89117800	1.85052100	0.03119700
C	3.00456800	2.86635300	0.04822300
H	2.91531700	3.51094600	0.92367400
H	3.96044400	2.33530700	0.10941900
H	2.99532500	3.45824000	-0.86747800
C	2.86565200	-3.00583600	0.02973900
H	3.46137000	-2.97171800	0.94252300
H	2.33354500	-3.96260900	-0.00081500
H	3.50655100	-2.94156800	-0.85042000
C	-2.86530200	3.00538000	-0.01152600
H	-2.33321400	3.96214700	0.01955900
H	-3.46070100	2.97158500	-0.92453100
H	-3.50650600	2.94078900	0.86838600
C	-3.00424700	-2.86676300	-0.03001400
H	-3.96010800	-2.33563800	-0.09076500
H	-2.91530600	-3.51110400	-0.90568300
H	-2.99476400	-3.45892000	0.88550900

Figure 1A: $(OAc)_4Rh_2$[Ph-COPh], Carbene

Rh	5.95799400	12.49912900	7.03463300
Rh	8.05033300	12.64439200	5.74063700
O	7.05639800	13.94579700	4.47774800
O	8.54386700	14.23973000	6.95509400
O	8.86822100	11.34472700	7.13435100
O	7.34509900	11.04628000	4.62304100
O	5.13239200	13.83036500	5.67872900
O	6.63680100	14.09429600	8.17465300
O	6.93128100	11.17679100	8.30473400

O	5.41819300	10.91041900	5.81398000
C	5.84314800	14.26338400	4.72254300
C	7.75467700	14.61663900	7.88461300
C	8.14616800	10.89413900	8.08938700
C	6.20552800	10.53359700	4.89700300
C	9.79583000	12.73615000	4.71959800
C	10.63987100	11.64663700	4.36469900
C	10.28411700	10.30578900	4.68314500
C	11.88365300	11.87882500	3.70570700
C	11.13519400	9.25925700	4.36196800
H	9.34335200	10.11915900	5.17756600
C	12.72424400	10.82654000	3.38816100
H	12.17003000	12.89251300	3.44943300
C	12.35066700	9.51603000	3.71770700
H	10.85816600	8.23997100	4.60880500
H	13.66816200	11.01367500	2.88792600
C	10.17932800	14.09982100	4.30100200
O	9.81115400	14.45169100	3.17643200
C	8.83691300	9.94981700	9.04312100
H	9.58284600	10.50640000	9.61880500
H	8.11716800	9.49723400	9.72465800
H	9.36335400	9.17632500	8.47897200
C	5.22402500	15.27355400	3.78701500
H	5.53980400	15.07935800	2.76044500
H	4.13696100	15.24951800	3.86421000
H	5.57783700	16.27192900	4.06464200
C	8.20347300	15.81863300	8.67984100
H	7.77918100	16.71844700	8.22178900
H	7.83770400	15.75217600	9.70552400
H	9.29084200	15.90127800	8.65983100
C	5.77615700	9.38264900	4.02001400
H	5.56952900	9.75903700	3.01342100
H	6.58821100	8.65595900	3.94164600
H	4.88070600	8.90773100	4.42001000
H	13.01226600	8.69190200	3.46931200
C	10.92581500	14.97550400	5.22916900
C	11.13618400	16.31639600	4.87878500
C	11.42726800	14.47948500	6.44108100
C	11.83481600	17.15759400	5.73911600
H	10.74131000	16.67659600	3.93485900
C	12.13204400	15.32124700	7.29758800
H	11.25253500	13.44484600	6.71706100
C	12.33207400	16.66022200	6.94883400
H	11.99342200	18.19776900	5.47305700
H	12.52000800	14.93874000	8.23607400
H	12.87680500	17.31732200	7.61981600

Figure 1B: TSeq_CO-site

Rh	6.25666800	12.69453100	6.13338500
Rh	8.51684400	12.73233300	5.20805300
O	7.78481500	13.90631900	3.67264900
O	8.81942200	14.44751200	6.31899600
O	9.09130900	11.58764400	6.83731500
O	8.00146000	11.04662700	4.10664900
O	5.69278600	13.91598100	4.55252600
O	6.75706300	14.35940700	7.25842200
O	6.97529200	11.47209100	7.64649300
O	5.89781200	11.02158600	4.94991300
C	6.55578600	14.24421400	3.68007600
C	7.91004400	14.87131400	7.10225800
C	8.21680700	11.20576900	7.68211000
C	6.82127200	10.56835200	4.20981900
C	10.63470000	12.60446000	4.31594700
C	11.47597600	11.47869000	4.76662000
C	10.91946500	10.19728200	4.95066100
C	12.86266100	11.63252400	4.96546300
C	11.71382000	9.12411900	5.34390300
H	9.85869200	10.05856400	4.78820800
C	13.65253800	10.56044600	5.37666400
H	13.32500600	12.59876300	4.80207600
C	13.08168500	9.30142200	5.57034800
H	11.26236700	8.14615800	5.48220600
H	14.71689500	10.70773000	5.53216300
C	11.11064100	13.99765700	4.29041100
O	10.93577700	14.73239000	3.30918400
C	8.72590300	10.37985800	8.83872800
H	9.18021900	11.04927000	9.57674700
H	7.90915800	9.83537500	9.31290700
H	9.49725500	9.68966100	8.49169000
C	6.10507500	15.14284400	2.55366700
H	6.60783500	14.86175100	1.62647500
H	5.02269900	15.09431400	2.43264800
H	6.38749600	16.17389500	2.79105900
C	8.24553800	16.11178200	7.89455100
H	8.13760300	16.98646500	7.24476700
H	7.57313500	16.21962100	8.74586000
H	9.28473800	16.06677400	8.22667600
C	6.52924300	9.34144600	3.38094700
H	5.46346300	9.11451200	3.38714000
H	6.87848300	9.49570200	2.35734600
H	7.08246600	8.49299900	3.79608400
H	13.69720700	8.46463400	5.88602000
C	11.75391000	14.55293800	5.52720800
C	12.47440300	15.74813000	5.39962700

C	11.63285700	13.95500500	6.79200700
C	13.07539300	16.33516400	6.51147900
H	12.55213700	16.20288300	4.41834100
C	12.22511200	14.54858900	7.90387400
H	11.05399400	13.04830600	6.91244000
C	12.94957300	15.73686600	7.76748800
H	13.63827100	17.25687500	6.40016200
H	12.11712900	14.08697400	8.88074700
H	13.41232900	16.19419600	8.63693600
C	9.56787900	12.14057400	-0.18460900
C	9.40960100	12.61993400	1.28455400
C	10.50555900	11.97386600	2.08287800
C	11.89839100	12.29649700	1.65126500
C	12.04767600	11.81686800	0.18026800
C	10.97288200	12.44067300	-0.71702500
H	9.97176600	12.39170300	3.39719100
H	9.52456100	13.70465300	1.33615700
H	8.42700200	12.35700500	1.67845200
H	9.37338100	11.06215100	-0.23817700
H	8.80059900	12.63490600	-0.78942600
H	12.05216800	13.37767300	1.69644800
H	12.63721600	11.80185500	2.28590700
H	13.05267000	12.07881900	-0.16571400
H	11.97071300	10.72306100	0.14482200
H	11.12094400	13.52748400	-0.75965200
H	11.07474700	12.06381100	-1.74062300
H	10.33739100	10.92821700	2.34637500

TSax_CO-site

Rh	6.02994000	12.78441300	6.47811700
Rh	8.18226600	12.77507400	5.30225300
O	7.27782000	13.88173500	3.80964100
O	8.58548700	14.54883500	6.28942100
O	8.92756500	11.69855300	6.91196700
O	7.55402600	11.01991400	4.38589900
O	5.28602400	13.90789900	4.89643500
O	6.63853800	14.50619100	7.45272800
O	6.92298600	11.65651400	7.97329700
O	5.54999500	11.04807000	5.44430200
C	6.04613100	14.19315700	3.92071800
C	7.76666000	15.00649500	7.14998300
C	8.15865700	11.38180900	7.87844100
C	6.39141300	10.55307600	4.63685700
C	10.16855100	12.68497000	4.23925600
C	11.07958700	11.56173800	4.54823600
C	10.57180400	10.28890700	4.87216100
C	12.47866000	11.71593600	4.45925600

C	11.43069800	9.22068800	5.11872300
H	9.50119500	10.15141700	4.92739400
C	13.33646700	10.64969200	4.71568300
H	12.89519000	12.67918700	4.18604700
C	12.81474100	9.39709900	5.04931100
H	11.01873000	8.24733500	5.36748200
H	14.41025300	10.79318700	4.64610000
C	10.67891600	14.07440400	4.26796000
O	10.46856500	14.87662700	3.34954600
C	8.80299100	10.63137500	9.01911500
H	9.35499900	11.34406300	9.64094800
H	8.04861700	10.13825100	9.63229600
H	9.51719700	9.90309800	8.63003100
C	5.44609100	14.94421700	2.75678100
H	5.27782000	14.23882400	1.93636400
H	4.49561900	15.39801200	3.03712300
H	6.14354600	15.70595100	2.40282400
C	8.19677900	16.26882800	7.85810200
H	8.18742700	17.09957700	7.14577000
H	7.52775100	16.49646400	8.68775100
H	9.22278700	16.15140800	8.21564400
C	5.99376800	9.31724500	3.86743300
H	5.76995400	9.60433400	2.83479700
H	6.82427700	8.60879300	3.84216100
H	5.11117000	8.85432800	4.30833800
H	13.48222900	8.56369000	5.24600900
C	11.42611700	14.53142300	5.48822300
C	12.13626400	15.73583300	5.39087500
C	11.42100000	13.83487700	6.70710000
C	12.83723800	16.23662300	6.48601700
H	12.12549900	16.26734100	4.44596200
C	12.11235800	14.34315900	7.80426000
H	10.85099100	12.92112700	6.81186700
C	12.82446400	15.54171200	7.69772700
H	13.38967600	17.16702900	6.39642900
H	12.09192100	13.80523500	8.74739300
H	13.36524500	15.93156600	8.55495600
C	7.63342000	12.06210600	1.15091700
C	8.81836500	13.03367700	1.19418700
C	10.05995800	12.46815300	1.82780400
C	10.39535000	11.03820000	1.55211100
C	9.17942500	10.10431200	1.53830600
C	8.05245000	10.66902300	0.66418000
H	10.89414000	13.16003200	1.87533300
H	9.14142200	13.27451800	0.16559900
H	8.55691800	13.97742600	1.67209300
H	7.21351700	11.98349600	2.15637800

H	6.85425100	12.48053300	0.50495800
H	10.86185700	11.06461400	0.55210100
H	11.18087700	10.68524900	2.22586200
H	9.48965400	9.11539800	1.18583700
H	8.81204000	9.98704900	2.56230500
H	8.38927000	10.72501300	-0.38035600
H	7.19117900	9.99203100	0.68078000
H	9.57911100	12.49068600	3.22393900

Figure 1C: TSeq_Ph-site

Rh	6.18517500	12.52283000	6.39846100
Rh	8.33563800	12.51144400	5.22477600
O	7.31594400	13.23640300	3.56942600
O	8.61788000	14.42859400	5.94884700
O	9.16754900	11.73863900	6.95462300
O	7.80396500	10.61080800	4.55625100
O	5.33655300	13.30993000	4.67093900
O	6.62749800	14.44139300	7.03507600
O	7.17260300	11.73786600	8.03938000
O	5.84221500	10.60936100	5.68990800
C	6.06719700	13.49471700	3.65257600
C	7.72379300	14.97229400	6.67481400
C	8.42219100	11.51559800	7.96375300
C	6.70547900	10.07968300	4.92605200
C	10.41726700	12.57460400	4.27008700
C	11.27567900	11.39487200	4.54871400
C	10.75313700	10.09515000	4.38539000
C	12.63555900	11.51156000	4.90581600
C	11.54599500	8.96788500	4.58245700
H	9.71050000	9.97601300	4.12343800
C	13.42272900	10.38029600	5.11419800
H	13.07239200	12.49140800	5.03519600
C	12.88554900	9.10216400	4.95426100
H	11.11267300	7.98073900	4.45083900
H	14.46481800	10.50167400	5.39453000
C	10.97155700	13.92309200	4.54304300
O	11.65071800	14.10026200	5.55921000
C	9.08579300	10.90294600	9.17360700
H	8.57683000	11.21711900	10.08645300
H	9.01375400	9.81335100	9.09621400
H	10.14026800	11.17931400	9.21293800
C	5.44018900	14.10151100	2.42140600
H	5.74677000	15.15118700	2.35606400
H	5.81135100	13.59735300	1.52662500
H	4.35329900	14.04797200	2.47029800
C	8.01626400	16.37165000	7.15903100
H	8.73227200	16.31660600	7.98472400

H	8.46933700	16.95450000	6.35477800
H	7.10530400	16.85721800	7.51101200
C	6.42573700	8.69433500	4.39493700
H	6.38430300	8.72726900	3.30252700
H	7.24656800	8.02774300	4.67366400
H	5.48504200	8.31039000	4.78848400
H	13.50266700	8.22326400	5.11418200
C	10.73440200	15.09153100	3.63775800
C	11.80474000	15.98572200	3.47364500
C	9.51880600	15.35249100	2.99350600
C	11.67441600	17.10135400	2.65028500
H	12.73701300	15.78593200	3.99072300
C	9.38436400	16.48443300	2.18781300
H	8.67434300	14.69363800	3.14011700
C	10.46080700	17.35417000	2.00449400
H	12.51491100	17.77510900	2.51531300
H	8.43354600	16.68533600	1.70296500
H	10.35458100	18.22686600	1.36733200
C	9.62751300	12.23631300	-0.42803200
C	9.30947400	12.69050100	1.02784400
C	10.43916400	12.20207000	1.88354600
C	11.79325800	12.73257400	1.54248100
C	12.10369800	12.28873000	0.08401400
C	11.00060600	12.74667400	-0.87650600
H	9.88089300	12.51531300	3.21759400
H	9.26059800	13.78157800	1.04915800
H	8.34978400	12.29296200	1.36210300
H	9.59950200	11.14139800	-0.48191400
H	8.83169000	12.61190200	-1.07924700
H	11.79614300	13.82509400	1.58528000
H	12.55514200	12.34799500	2.22503400
H	13.07531500	12.70401600	-0.20135000
H	12.19689400	11.19650200	0.05021700
H	10.98520500	13.84353500	-0.91681200
H	11.21729300	12.39298500	-1.89034900
H	10.41754600	11.13452900	2.10424300

TSax_Ph-site

Rh	6.03960200	12.92744400	6.54665200
Rh	8.09236200	12.86007500	5.18574300
O	7.01006800	13.85091700	3.71922300
O	8.62694300	14.65183600	6.08533600
O	8.95867800	11.87370300	6.79857600
O	7.40984600	11.05816300	4.42099700
O	5.12591100	13.93384300	4.98113000
O	6.72302700	14.72631100	7.32293600
O	7.05499000	11.89855500	8.03211200

O	5.51146600	11.11840900	5.66260100
C	5.78982700	14.15098800	3.92045200
C	7.84795900	15.17894800	6.94539900
C	8.27621200	11.60695200	7.83863000
C	6.28413700	10.59262800	4.80725000
C	9.95526900	12.77571500	4.09703300
C	10.84811800	11.78757500	4.71871300
C	10.56102600	10.41004400	4.64163300
C	11.87748900	12.19839700	5.59261700
C	11.31541600	9.47774200	5.34567900
H	9.71205800	10.08115300	4.05879000
C	12.60457200	11.26466700	6.32575300
H	12.08793500	13.25143500	5.72670500
C	12.34541600	9.89910400	6.19131500
H	11.08373500	8.42110200	5.25222500
H	13.38260600	11.60817300	7.00063200
C	10.48352500	14.13745900	3.64656200
O	9.68122000	15.01277600	3.34636500
C	9.00940200	10.90043700	8.95520600
H	9.36583100	11.64880400	9.67108200
H	8.33558100	10.22209400	9.48130000
H	9.87009400	10.35870600	8.56128100
C	5.06864800	14.80057800	2.76262600
H	4.83282100	14.02945300	2.02184700
H	4.14223900	15.26828400	3.09625100
H	5.71645400	15.53640100	2.28213200
C	8.33540700	16.45223000	7.59726800
H	7.49192700	17.04936300	7.94570500
H	8.95431600	16.18893900	8.46176800
H	8.94884800	17.02567600	6.90078200
C	5.84305800	9.30613700	4.15054900
H	5.59010700	9.51131700	3.10540700
H	6.66325700	8.58469900	4.15633900
H	4.97192700	8.89190600	4.65748000
H	12.92815500	9.17330600	6.74981200
C	11.94592700	14.43475000	3.50761500
C	12.37002000	15.74563800	3.77496300
C	12.87642400	13.49395700	3.03829000
C	13.70433200	16.10362800	3.60356400
H	11.64174000	16.46911900	4.12485700
C	14.20497100	13.86334800	2.83675600
H	12.56901600	12.47689800	2.82809400
C	14.62350900	15.16323200	3.12923800
H	14.02740100	17.11434300	3.83197300
H	14.91438700	13.13397500	2.45931300
H	15.66267100	15.44333900	2.98731100
C	7.26844000	11.94333600	1.15052000

C	8.50769300	12.84373500	1.12393900
C	9.74024400	12.20336500	1.71931000
C	9.97415200	10.74438300	1.42754400
C	8.70195300	9.89113700	1.46819900
C	7.57718500	10.52950000	0.64262300
H	10.63582300	12.80773500	1.59912400
H	8.78936000	13.06810000	0.08188000
H	8.31414500	13.80179500	1.61176100
H	6.89945100	11.88897400	2.17714800
H	6.48066900	12.40751800	0.54739200
H	10.39330400	10.73376900	0.40810600
H	10.76319200	10.34137400	2.07035900
H	8.93254200	8.88465000	1.10422800
H	8.36100400	9.79442200	2.50402900
H	7.87345400	10.56845100	-0.41487400
H	6.67713200	9.90682100	0.69457700
H	9.45308500	12.29710000	3.02395900

(c-Hex)$Rh_2(OAc)_4$[Ph-COPh], Ph-site

Rh	-2.55494500	0.53186600	1.08754300
Rh	-0.14700200	0.09017700	0.79534600
O	-0.33000700	1.02390600	-1.04487700
O	0.18339900	1.93236100	1.68201600
O	-0.19507800	-0.82450100	2.65235800
O	-0.70780000	-1.70666500	-0.08782900
O	-2.55716400	1.38321400	-0.80133900
O	-2.03359800	2.37303100	1.88647100
O	-2.39169900	-0.34464300	2.96216500
O	-2.91590300	-1.34379400	0.28834400
C	-1.46505900	1.45624200	-1.44006000
C	-0.80810000	2.67088500	2.00531700
C	-1.28160700	-0.83105000	3.32800200
C	-1.93879700	-2.05100200	-0.09895600
C	1.84592700	-0.23500600	0.61887000
C	2.51311300	-1.48858300	0.52162300
C	1.84754900	-2.70329700	0.83839700
C	3.85415000	-1.54973900	0.04519200
C	2.49143100	-3.91911500	0.66528000
H	0.84103400	-2.65866200	1.22607600
C	4.47647200	-2.77052300	-0.15379100
H	4.37728100	-0.63057000	-0.19474500
C	3.79540000	-3.95538700	0.15745800
H	1.98275800	-4.84318400	0.91811700
H	5.48871300	-2.81035100	-0.54107900
C	2.66161900	0.99430800	0.73298500
O	3.09434500	1.20598000	1.86961600
C	-1.20118800	-1.46587900	4.69493500

H	-0.64051200	-0.80275400	5.36132900
H	-2.19918800	-1.62506400	5.10275300
H	-0.65935400	-2.41218600	4.63422300
C	-1.49369000	2.08523100	-2.81180800
H	-1.69082700	1.30280600	-3.55184800
H	-2.29234400	2.82555900	-2.87166500
H	-0.52841400	2.53830500	-3.04089900
C	-0.45619700	4.01371000	2.59852600
H	-1.34619600	4.63390900	2.70203600
H	-0.00168200	3.86151000	3.58226200
H	0.28243500	4.51201800	1.96618300
C	-2.25021800	-3.42135100	-0.64962800
H	-2.40611300	-3.34134400	-1.72957700
H	-1.41690700	-4.10196400	-0.47209900
H	-3.16418600	-3.80728700	-0.19664800
H	4.28815000	-4.91124200	0.00912600
C	2.85749400	1.91328300	-0.40177500
C	3.42535500	3.17525900	-0.17106100
C	2.50418500	1.52789400	-1.70107500
C	3.62817500	4.04734900	-1.23519000
H	3.69381400	3.44963100	0.84341800
C	2.71901600	2.39838900	-2.76712300
H	2.05354400	0.55879800	-1.86993900
C	3.27625200	3.65807000	-2.53338200
H	4.05995100	5.02759500	-1.06055800
H	2.44756700	2.09886200	-3.77373100
H	3.43768100	4.33930800	-3.36318100
C	-0.75304500	-2.04781700	-3.69363600
C	-0.31277800	-1.15001900	-4.85826600
C	0.94322100	-0.34763600	-4.49136800
C	2.08356200	-1.26908800	-4.03461800
C	1.63490800	-2.17362200	-2.87920900
C	0.38390100	-2.97838100	-3.24971900
H	1.26437000	0.26840400	-5.33998000
H	-0.10015900	-1.77511800	-5.73688700
H	-1.12586800	-0.47276900	-5.14727200
H	-1.04104700	-1.41597400	-2.84343900
H	-1.63860500	-2.62927100	-3.97778300
H	2.40372700	-1.89346600	-4.88044200
H	2.95702600	-0.67609900	-3.73747100
H	2.44880200	-2.84176800	-2.57555800
H	1.39399900	-1.55926600	-2.00462900
H	0.62297300	-3.67323300	-4.06744900
H	0.06809700	-3.58736900	-2.39686900
H	0.69072700	0.34030400	-3.67527100

Prod, P2

Rh	-2.79416700	0.03890700	1.81687500
Rh	-0.76643000	0.51573400	0.60093200
O	-1.94592700	1.02789700	-1.01172500
O	-0.87870400	2.45832600	1.28961000
O	0.29225100	-0.02227900	2.29403200
O	-0.79992300	-1.46340100	-0.00491900
O	-3.85053100	0.51746200	0.10437100
O	-2.81224900	2.02336700	2.39146600
O	-1.61284400	-0.43426600	3.44971200
O	-2.66684400	-1.92764800	1.18499400
C	-3.20998200	0.89313600	-0.92834500
C	-1.86466100	2.79004600	2.02580500
C	-0.34797500	-0.37855500	3.33811100
C	-1.70914900	-2.25096900	0.41549900
C	1.77126000	-0.85190000	-1.64196900
C	2.52333300	-1.95362700	-0.88900600
C	2.31918500	-2.06413600	0.49596700
C	3.38901200	-2.86337300	-1.50877000
C	2.96525200	-3.05327200	1.23647800
H	1.64832300	-1.37774000	0.99944500
C	4.03421300	-3.85620100	-0.76722800
H	3.56848200	-2.81071200	-2.57651700
C	3.82670300	-3.95568800	0.60835600
H	2.79211300	-3.11941200	2.30683500
H	4.69863900	-4.55196800	-1.27086900
C	2.09322500	0.50442200	-1.02699100
O	1.22637900	1.10666400	-0.37543700
C	0.47941100	-0.73879200	4.54636400
H	0.85208600	0.18184000	5.00699800
H	-0.12027000	-1.28369600	5.27521500
H	1.34301500	-1.33314900	4.24111200
C	-4.00762100	1.17233500	-2.17724800
H	-4.11821100	0.23604300	-2.73476400
H	-5.00256700	1.53829700	-1.92063300
H	-3.48455700	1.88940400	-2.81072200
C	-1.89012600	4.20973800	2.53467900
H	-2.91541500	4.52497900	2.73135500
H	-1.32805500	4.25445700	3.47364700
H	-1.41316100	4.87947300	1.81795800
C	-1.62781100	-3.67644000	-0.06703500
H	-1.89172800	-3.70587300	-1.12899300
H	-0.59981800	-4.03339800	0.02858900
H	-2.30912800	-4.31682400	0.49250400
H	4.32844300	-4.72762800	1.18376400
C	3.41694200	1.15538200	-1.19336400
C	3.53695500	2.51518600	-0.85349200
C	4.54120800	0.46886300	-1.68076600

C	4.75020600	3.17572000	-1.00892100
H	2.66382300	3.03715700	-0.47950900
C	5.75771000	1.13189300	-1.82658200
H	4.47882600	-0.58409700	-1.91652100
C	5.86409200	2.48440800	-1.49629800
H	4.83104400	4.22709000	-0.75233300
H	6.62311700	0.59104000	-2.19543200
H	6.81254000	2.99891500	-1.61567700
C	-0.07300400	0.66983500	-3.76425900
C	1.43679700	0.42431700	-3.87176500
C	1.89158900	-0.88404700	-3.19044500
C	1.06463400	-2.06158300	-3.75591100
C	-0.45178600	-1.82986600	-3.65418100
C	-0.86842700	-0.51527400	-4.32604100
H	2.94213500	-1.03309800	-3.45950300
H	1.71143200	0.35218600	-4.93243200
H	1.99318000	1.27978600	-3.47381700
H	-0.36026800	0.83151100	-2.72164800
H	-0.33240900	1.58870100	-4.30393300
H	1.33501000	-2.18666300	-4.81266300
H	1.33283800	-2.99450900	-3.25069700
H	-0.97808000	-2.67570900	-4.11192700
H	-0.75785800	-1.80839100	-2.60149400
H	-0.69057900	-0.59135900	-5.40827000
H	-1.94340000	-0.34622000	-4.19336500
H	0.72201200	-0.99710000	-1.39394100

Structure 3A: [Rh_2(*R*-TPPTTL)$_4$•di-phenyl carbene]

Rh	34.8802680	1.1078800	-2.7762050
Rh	33.9601440	3.1543030	-3.8190040
O	35.5856440	3.1389940	-5.1183100
O	36.4393320	1.3163830	-4.0775870
O	39.0451120	-0.3023440	-5.7495770
O	38.6426950	3.4564130	-3.2112090
N	38.8447360	1.8280230	-4.8541520
C	39.3193440	0.5041810	-4.8812300
C	40.1892640	0.3533380	-3.6759880
C	40.2096560	1.5813440	-3.0072400
C	39.1777640	2.4519510	-3.6446850
C	40.9334450	-0.7395140	-3.2415930
C	41.7907890	-0.5269720	-2.1268910
C	41.8528150	0.7342020	-1.4872440
C	41.0394880	1.8186370	-1.9150330
C	37.7702050	2.2648140	-5.7454880
H	37.6433960	1.4220520	-6.4347390
C	36.4790980	2.2738820	-4.9186640
C	38.1028920	3.5085260	-6.6215920

C	38.0352210	4.8530540	-5.8769150
H	38.7799640	4.9136160	-5.0826560
H	38.2283350	5.6618100	-6.5903070
H	37.0514910	5.0128350	-5.4333170
C	37.1043770	3.5218830	-7.7960600
H	37.1512750	2.5910610	-8.3665820
H	36.0820710	3.6552430	-7.4381640
H	37.3455040	4.3475880	-8.4736230
C	39.5258810	3.3184730	-7.1770830
H	40.2774750	3.3894170	-6.3855560
H	39.6338760	2.3477050	-7.6662240
H	39.7393010	4.0962090	-7.9172100
C	40.7724250	-2.0740180	-3.8770970
C	41.8579270	-2.7859000	-4.4034030
H	42.8474150	-2.3420480	-4.3892100
C	41.6734170	-4.0654200	-4.9266740
H	42.5214750	-4.6079590	-5.3329260
C	40.4052030	-4.6523080	-4.9206910
H	40.2625920	-5.6479390	-5.3275250
C	39.3162300	-3.9398030	-4.4122480
H	38.3224140	-4.3748990	-4.4090770
C	39.4975040	-2.6570450	-3.9030840
H	38.6504200	-2.1071440	-3.5116210
C	42.6562890	-1.6422500	-1.6411380
C	44.0515230	-1.5066340	-1.6412330
H	44.4972150	-0.5828150	-1.9935600
C	44.8649900	-2.5447870	-1.1897560
H	45.9440130	-2.4251350	-1.1995090
C	44.2948540	-3.7324620	-0.7260900
H	44.9281670	-4.5390140	-0.3697000
C	42.9062860	-3.8791320	-0.7296770
H	42.4533220	-4.8010560	-0.3780300
C	42.0943960	-2.8447370	-1.1917190
H	41.0196530	-2.9728150	-1.2192190
C	42.7700660	0.9397530	-0.3271260
C	43.8178890	1.8660820	-0.4121610
H	43.9629310	2.4220030	-1.3327070
C	44.6635140	2.0804740	0.6750700
H	45.4741070	2.7979550	0.5928310
C	44.4659690	1.3792590	1.8668110
H	45.1211760	1.5499230	2.7153290
C	43.4214310	0.4573530	1.9609070
H	43.2604260	-0.0927760	2.8830310
C	42.5829900	0.2370370	0.8694160
H	41.7767260	-0.4808700	0.9482810
C	41.1037710	3.1600460	-1.2751280
C	41.3744300	4.2872790	-2.0650270

H	41.5243430	4.1657790	-3.1330020
C	41.4672880	5.5527590	-1.4892460
H	41.6837110	6.4141390	-2.1133910
C	41.2866780	5.7093040	-0.1131050
H	41.3574780	6.6943140	0.3376790
C	41.0093780	4.5934730	0.6791340
H	40.8563730	4.7067950	1.7478220
C	40.9216850	3.3275500	0.1039280
H	40.7067560	2.4727480	0.7311690
O	32.8966770	1.9195520	-5.1055330
O	33.8153210	0.0657950	-4.1866690
O	32.8401880	-3.1362120	-5.8952300
O	35.1118180	0.6144450	-7.1959440
N	33.6349860	-1.0822180	-6.6205960
C	33.7222760	-2.4670150	-6.4018210
C	35.0460470	-2.8860650	-6.9490290
C	35.6611150	-1.7573130	-7.5004820
C	34.8336920	-0.5702220	-7.1370080
C	35.6172760	-4.1507790	-7.0251990
C	36.8545570	-4.2572180	-7.7128290
C	37.4580920	-3.1209340	-8.2998130
C	36.8521350	-1.8378530	-8.2183060
C	32.5279830	-0.2781630	-6.1017300
H	31.9328290	-0.9930800	-5.5235910
C	33.1178730	0.6797600	-5.0560640
C	31.5900620	0.3035210	-7.1958950
C	32.2362330	1.3966070	-8.0633290
H	33.0942760	1.0153180	-8.6207190
H	31.4977110	1.7568720	-8.7879320
H	32.5699850	2.2395050	-7.4576070
C	30.3429460	0.8685240	-6.4883950
H	30.6002190	1.7209450	-5.8569700
H	29.6149740	1.2003830	-7.2358900
H	29.8625640	0.1091320	-5.8652160
C	31.1648610	-0.8642120	-8.1059740
H	32.0145380	-1.2547070	-8.6741410
H	30.7348710	-1.6838900	-7.5258340
H	30.4118150	-0.5194290	-8.8215640
C	34.9696370	-5.3312190	-6.3907610
C	34.4557930	-6.3791910	-7.1631780
H	34.5243970	-6.3298160	-8.2451900
C	33.8644530	-7.4814710	-6.5457510
H	33.4629620	-8.2873600	-7.1526200
C	33.7901190	-7.5497110	-5.1523540
H	33.3311340	-8.4092570	-4.6733120
C	34.3089930	-6.5093390	-4.3780500
H	34.2520980	-6.5522350	-3.2947240

C	34.8950800	-5.4044110	-4.9943410
H	35.2918870	-4.5850260	-4.4045310
C	37.4938810	-5.5987420	-7.8565570
C	37.7216980	-6.1431940	-9.1282400
H	37.4360600	-5.5781970	-10.0084490
C	38.3114370	-7.3986380	-9.2693390
H	38.4783670	-7.8050530	-10.2622080
C	38.6842630	-8.1303970	-8.1399450
H	39.1469700	-9.1063770	-8.2495190
C	38.4505570	-7.6023210	-6.8685900
H	38.7275740	-8.1660730	-5.9829220
C	37.8543500	-6.3497150	-6.7296750
H	37.6561950	-5.9559280	-5.7408550
C	38.7704320	-3.2663760	-8.9959140
C	38.8802540	-3.1138620	-10.3830460
H	37.9938110	-2.8899390	-10.9680740
C	40.1201640	-3.2410000	-11.0083340
H	40.1942270	-3.1256550	-12.0854560
C	41.2638920	-3.5106750	-10.2523010
H	42.2291980	-3.6046970	-10.7404320
C	41.1600790	-3.6548150	-8.8668970
H	42.0426960	-3.8573450	-8.2673280
C	39.9191730	-3.5358460	-8.2432660
H	39.8382380	-3.6398770	-7.1680580
C	37.3872760	-0.6790840	-8.9840500
C	36.5533240	-0.0556780	-9.9244610
H	35.5240170	-0.3853360	-10.0240690
C	37.0419970	0.9560660	-10.7496460
H	36.3844740	1.4227120	-11.4766460
C	38.3757740	1.3560400	-10.6485870
H	38.7626310	2.1363150	-11.2967510
C	39.2082210	0.7496810	-9.7057670
H	40.2453780	1.0580300	-9.6190350
C	38.7187050	-0.2554410	-8.8732870
H	39.3649960	-0.7142760	-8.1378540
O	35.1276160	4.2703280	-2.5169150
O	35.8141200	2.3929620	-1.4512760
O	38.0973220	2.6421050	1.0750970
O	33.9083310	4.3805160	0.6055560
N	36.1783410	3.8860200	0.7245160
C	36.9484200	2.9034200	1.3762700
C	36.0694770	2.3085310	2.4282760
C	34.8402570	2.9721410	2.3679030
C	34.8500610	3.8563750	1.1718640
C	36.2906780	1.2562840	3.3192510
C	35.2088490	0.9237140	4.1800920
C	34.0085960	1.6713350	4.1843370

C	33.8029500	2.7260040	3.2621080
C	36.5997050	4.4137680	-0.5758550
H	37.6111720	4.0152070	-0.7028210
C	35.7658550	3.6584580	-1.6216500
C	36.7283890	5.9590180	-0.6586290
C	35.3940970	6.7221750	-0.6324500
H	34.7434360	6.4098320	-1.4501640
H	35.5986640	7.7932310	-0.7392950
H	34.8581700	6.5703460	0.3055860
C	37.4839270	6.2921530	-1.9602270
H	38.4522370	5.7858650	-1.9990880
H	37.6574490	7.3718090	-2.0149320
H	36.9095890	5.9917610	-2.8387060
C	37.5861780	6.4061400	0.5398980
H	37.0717440	6.2240480	1.4881080
H	37.7918090	7.4788160	0.4668960
H	38.5439990	5.8788350	0.5624060
C	37.5799450	0.5132320	3.3566070
C	38.7885050	1.2005650	3.5368730
H	38.7784080	2.2795700	3.6382830
C	39.9984560	0.5097480	3.5852650
H	40.9263280	1.0570120	3.7170100
C	40.0155960	-0.8814990	3.4657910
H	40.9560980	-1.4225840	3.5123020
C	38.8166680	-1.5747650	3.2834640
H	38.8217200	-2.6550000	3.1773440
C	37.6089460	-0.8824590	3.2222230
H	36.6833660	-1.4260320	3.0723180
C	35.3401790	-0.2113300	5.1382530
C	36.2513740	-0.1602880	6.1993200
H	36.8721520	0.7207550	6.3265420
C	36.3657190	-1.2334100	7.0830380
H	37.0722390	-1.1803630	7.9056730
C	35.5781140	-2.3734220	6.9088050
H	35.6722740	-3.2106990	7.5933240
C	34.6712110	-2.4339880	5.8478870
H	34.0628560	-3.3212090	5.6998580
C	34.5489470	-1.3560010	4.9721840
H	33.8489330	-1.3997640	4.1441000
C	33.0036950	1.4373590	5.2655160
C	33.2445710	1.9850090	6.5327190
H	34.1605170	2.5416960	6.7055560
C	32.3179060	1.8221230	7.5620540
H	32.5155120	2.2533340	8.5388150
C	31.1392420	1.1084960	7.3336760
H	30.4142370	0.9832500	8.1322300
C	30.8986050	0.5547560	6.0754510

H	29.9913370	-0.0098750	5.8906850
C	31.8270190	0.7138350	5.0456490
H	31.6343240	0.2753600	4.0730700
C	32.6393730	3.6506670	3.3376030
C	32.8917920	5.0037110	3.6130110
H	33.9169600	5.3362810	3.7443260
C	31.8430420	5.9132060	3.7319690
H	32.0554840	6.9555860	3.9488420
C	30.5239230	5.4810270	3.5784250
H	29.7036420	6.1855850	3.6753720
C	30.2662990	4.1389110	3.2963770
H	29.2437410	3.7995770	3.1654740
C	31.3152020	3.2267060	3.1714150
H	31.1090400	2.1953040	2.9185460
O	32.4332250	3.0069360	-2.4212780
O	33.2461730	1.0970230	-1.5097360
O	30.8869330	0.2485900	1.6973730
O	30.6806510	0.0905850	-2.8775000
N	30.7704800	0.5279360	-0.6003710
C	30.6183770	-0.1908210	0.5922400
C	30.0817080	-1.5292220	0.2014280
C	29.9771000	-1.5535560	-1.1951590
C	30.4956600	-0.2608800	-1.7266120
C	29.6476070	-2.5929640	0.9875450
C	29.1412580	-3.7325620	0.3010370
C	29.0447300	-3.7547530	-1.1089240
C	29.4380630	-2.6314200	-1.8834200
C	31.2047410	1.9207110	-0.6480330
H	31.6416300	2.1101910	0.3389310
C	32.3818540	2.0258830	-1.6307740
C	30.0217070	2.9258270	-0.8237760
C	29.3984470	2.8969360	-2.2303380
H	28.9670000	1.9204080	-2.4615510
H	28.5926280	3.6373730	-2.2769410
H	30.1343120	3.1364640	-2.9982990
C	30.5355800	4.3433510	-0.5035790
H	31.2513240	4.6811280	-1.2531920
H	29.6919920	5.0411890	-0.4885640
H	31.0209380	4.3776680	0.4754900
C	28.9288140	2.5563540	0.1983930
H	28.4635750	1.5949990	-0.0386180
H	29.3275750	2.5018960	1.2127990
H	28.1433500	3.3177970	0.1820310
C	29.6638580	-2.5597850	2.4766280
C	28.4643220	-2.7160330	3.1855720
H	27.5314070	-2.8062840	2.6397920
C	28.4658010	-2.7774970	4.5778310

H	27.5287480	-2.8990070	5.1122840
C	29.6697290	-2.6943470	5.2800580
H	29.6759600	-2.7542150	6.3638970
C	30.8668510	-2.5197890	4.5829230
H	31.8004570	-2.4264600	5.1262350
C	30.8622820	-2.4404720	3.1914300
H	31.7902350	-2.2857600	2.6549540
C	28.7000760	-4.9226770	1.0863130
C	27.3801210	-5.3857080	0.9991840
H	26.6770980	-4.8714970	0.3528410
C	26.9687130	-6.4967970	1.7331200
H	25.9412500	-6.8396150	1.6591260
C	27.8737790	-7.1664160	2.5597820
H	27.5542570	-8.0340970	3.1286070
C	29.1905290	-6.7115740	2.6540590
H	29.9003820	-7.2232890	3.2966070
C	29.5982730	-5.5940460	1.9273790
H	30.6152110	-5.2302200	2.0262690
C	28.5466750	-4.9753590	-1.8088900
C	27.3832880	-4.9323670	-2.5887990
H	26.8311140	-4.0030020	-2.6729930
C	26.9367020	-6.0711130	-3.2575230
H	26.0311400	-6.0228810	-3.8544260
C	27.6516600	-7.2669960	-3.1631980
H	27.3067340	-8.1519000	-3.6890210
C	28.8110530	-7.3193840	-2.3868260
H	29.3724430	-8.2449740	-2.3048720
C	29.2511870	-6.1828920	-1.7101010
H	30.1401430	-6.2357770	-1.0918080
C	29.2059490	-2.5821490	-3.3540900
C	28.2091650	-1.7299230	-3.8504990
H	27.6721330	-1.0824250	-3.1641340
C	27.8903730	-1.7329530	-5.2068640
H	27.1075470	-1.0791490	-5.5786100
C	28.5772990	-2.5757440	-6.0844240
H	28.3300040	-2.5780050	-7.1412600
C	29.5930200	-3.4011590	-5.6004610
H	30.1549650	-4.0353310	-6.2773100
C	29.9012160	-3.4096790	-4.2406630
H	30.6800290	-4.0616290	-3.8684890
C	38.4419690	-2.5134530	-0.4113660
C	37.3239550	-2.2472500	-1.1762120
C	36.9200220	-0.9006830	-1.4390570
C	37.7133830	0.1552210	-0.9111120
C	38.8242650	-0.1246150	-0.1324250
C	39.1894700	-1.4503680	0.1122540
H	38.7376660	-3.5380900	-0.2139700

H	36.7477570	-3.0704510	-1.5801550
H	37.4209400	1.1767390	-1.1046340
H	39.3869820	0.6868950	0.3053710
H	40.0566590	-1.6671120	0.7222200
C	35.7172080	-0.6456130	-2.1540420
C	35.0484480	-1.8299410	-2.7403630
C	34.0082150	-2.5475000	-1.9769040
C	33.8850730	-2.3491120	-0.5924280
C	33.2705550	-3.5590040	-2.6104650
C	33.0885020	-3.2077750	0.1602300
H	34.4392800	-1.5555540	-0.1024000
C	32.4714430	-4.4070140	-1.8518040
H	33.3446290	-3.6757440	-3.6839370
C	32.3981100	-4.2470110	-0.4656650
H	33.0295020	-3.0873650	1.2350820
H	31.9184050	-5.2054310	-2.3348450
O	35.4503170	-2.1909300	-3.8500530
H	31.8004540	-4.9297790	0.1268040

Structure 3B: [Rh_2(*R*-TPPTTL)$_4$•di-phenyl carbene]-inverted

Rh	0.7914760	1.7275490	0.0000000
Rh	-0.0214340	3.6856760	-1.2711620
O	1.7738990	3.6002920	-2.3084910
O	2.5643000	1.9092230	-1.0231140
O	4.6645270	-0.1034620	-2.6864560
O	5.3078280	3.7801600	-0.3886450
N	4.9799080	2.0698440	-1.9288170
C	5.1931010	0.6789580	-1.9191840
C	6.1698470	0.4205510	-0.8220100
C	6.5207840	1.6515600	-0.2598750
C	5.5844380	2.6749690	-0.8210770
C	6.7048470	-0.7784130	-0.3643760
C	7.7064920	-0.6901570	0.6396570
C	8.1168730	0.5620810	1.1577590
C	7.4978580	1.7669550	0.7256130
C	3.9498310	2.6650210	-2.7751120
H	3.7276200	1.8719380	-3.4993140
C	2.6546450	2.7650940	-1.9659200
C	4.3880510	3.9162020	-3.6085660
C	4.2329480	5.2648150	-2.8826620
H	4.8435830	5.3063900	-1.9818240
H	4.5523990	6.0659040	-3.5582790
H	3.1950830	5.4496620	-2.6034510
C	3.5219490	3.9315360	-4.8850090
H	3.6680000	3.0230470	-5.4742360
H	2.4625970	4.0121430	-4.6339580
H	3.7973420	4.7899450	-5.5066270

C	5.8625990	3.7378870	-4.0103190
H	6.5318840	3.8534810	-3.1530000
H	6.0396550	2.7579960	-4.4577360
H	6.1326750	4.5000350	-4.7482650
C	6.1933850	-2.0920110	-0.8405480
C	7.0373350	-3.0426510	-1.4291410
H	8.0810480	-2.8010420	-1.5985700
C	6.5475650	-4.3029090	-1.7714800
H	7.2120490	-5.0327710	-2.2235830
C	5.2114520	-4.6276040	-1.5234750
H	4.8378570	-5.6163780	-1.7699680
C	4.3596550	-3.6779030	-0.9558700
H	3.3212170	-3.9210040	-0.7600160
C	4.8463720	-2.4157540	-0.6218750
H	4.1897990	-1.6834870	-0.1650820
C	8.3345900	-1.9462610	1.1444600
C	9.6985530	-2.1912590	0.9412420
H	10.3006400	-1.4532840	0.4212820
C	10.2817910	-3.3684790	1.4079590
H	11.3392810	-3.5483190	1.2403940
C	9.5094950	-4.3115120	2.0898180
H	9.9648030	-5.2262970	2.4565040
C	8.1487890	-4.0738010	2.2959200
H	7.5412560	-4.8022930	2.8240970
C	7.5639170	-2.9011460	1.8207960
H	6.5042360	-2.7243490	1.9663950
C	9.2006480	0.6308280	2.1814210
C	10.3441650	1.4053250	1.9382530
H	10.4310230	1.9466970	1.0022250
C	11.3612770	1.4883260	2.8875650
H	12.2412640	2.0896780	2.6813210
C	11.2484320	0.8019240	4.0987950
H	12.0390210	0.8680190	4.8397660
C	10.1149170	0.0260010	4.3489370
H	10.0193730	-0.5156050	5.2850580
C	9.1010430	-0.0596130	3.3966460
H	8.2351910	-0.6802050	3.5915580
C	7.8392630	3.0981740	1.2940990
C	8.2107100	4.1445760	0.4376720
H	8.2798280	3.9594240	-0.6294900
C	8.4938190	5.4103150	0.9456030
H	8.7835220	6.2095460	0.2703830
C	8.3997720	5.6492760	2.3189570
H	8.6124020	6.6370750	2.7162660
C	8.0349570	4.6115890	3.1781860
H	7.9528670	4.7863230	4.2450360
C	7.7656570	3.3414080	2.6727130

H	7.4921680	2.5452010	3.3551130
O	-0.8268270	2.3628910	-2.6216720
O	0.0589750	0.5687890	-1.5592070
O	-0.8742640	-2.6721970	-3.5342370
O	1.6885760	0.9999940	-4.4414760
N	0.1024660	-0.6410390	-4.0355640
C	0.0693770	-2.0400270	-3.9662880
C	1.3814920	-2.5147260	-4.4961410
C	2.1383720	-1.3853540	-4.8311320
C	1.3532980	-0.1713340	-4.4532280
C	1.8641070	-3.8125630	-4.6622620
C	3.1875540	-3.9374030	-5.1660690
C	3.9527550	-2.7956360	-5.4899520
C	3.4164650	-1.4858460	-5.3761440
C	-1.0362700	0.1730760	-3.6163800
H	-1.6950750	-0.5351660	-3.1029040
C	-0.5637880	1.1347990	-2.5191300
C	-1.8510190	0.7617330	-4.8092070
C	-1.1125270	1.8635970	-5.5881540
H	-0.1827000	1.4956160	-6.0270810
H	-1.7542950	2.2095970	-6.4058850
H	-0.8753250	2.7142340	-4.9492540
C	-3.1828130	1.3076880	-4.2587440
H	-3.0190680	2.1450870	-3.5792650
H	-3.8047600	1.6536530	-5.0909890
H	-3.7357190	0.5336950	-3.7196710
C	-2.1614160	-0.3977530	-5.7757560
H	-1.2534700	-0.7733450	-6.2576580
H	-2.6480010	-1.2279300	-5.2593300
H	-2.8335720	-0.0448680	-6.5640900
C	1.0512190	-5.0035910	-4.2910250
C	-0.2326890	-5.1934060	-4.8192070
H	-0.6282120	-4.4769270	-5.5301350
C	-1.0052410	-6.2864110	-4.4298150
H	-2.0006850	-6.4163670	-4.8430940
C	-0.5031720	-7.2083390	-3.5084210
H	-1.1068180	-8.0573130	-3.2028490
C	0.7814500	-7.0357800	-2.9870890
H	1.1823870	-7.7492220	-2.2737390
C	1.5535360	-5.9422850	-3.3776600
H	2.5480610	-5.8072350	-2.9674250
C	3.7241600	-5.2965620	-5.4731350
C	3.0661330	-6.0938480	-6.4204890
H	2.1772770	-5.7120650	-6.9121950
C	3.5424850	-7.3667650	-6.7304900
H	3.0240750	-7.9700080	-7.4695250
C	4.6812000	-7.8636860	-6.0923860

H	5.0505880	-8.8564640	-6.3305180
C	5.3422310	-7.0759230	-5.1479340
H	6.2279530	-7.4530640	-4.6455720
C	4.8697320	-5.7996670	-4.8446070
H	5.3849860	-5.1912890	-4.1141010
C	5.3349920	-2.9779810	-6.0252510
C	5.5519860	-3.2239420	-7.3843710
H	4.7038920	-3.2762340	-8.0599010
C	6.8490210	-3.4042320	-7.8670100
H	7.0089410	-3.5934040	-8.9241500
C	7.9376000	-3.3444550	-6.9941240
H	8.9456100	-3.4878840	-7.3710940
C	7.7248850	-3.0999880	-5.6354120
H	8.5650210	-3.0528040	-4.9490500
C	6.4296190	-2.9149940	-5.1543410
H	6.2611270	-2.7249130	-4.0994550
C	4.0908880	-0.3208640	-6.0111890
C	3.4138080	0.3348920	-7.0514050
H	2.4048490	0.0257050	-7.3060110
C	4.0324550	1.3527390	-7.7755650
H	3.4946620	1.8442870	-8.5804110
C	5.3430740	1.7266140	-7.4739800
H	5.8326490	2.5109700	-8.0428550
C	6.0178870	1.0900150	-6.4306180
H	7.0347320	1.3795990	-6.1845000
C	5.3953900	0.0813150	-5.6968720
H	5.9163170	-0.3899200	-4.8761830
O	0.9488990	4.9049030	0.1090330
O	1.5063130	3.1007920	1.3552150
O	4.5964310	3.9148690	3.5239730
O	0.1013610	4.7779770	3.4604730
N	2.4106570	4.6356100	3.2901120
C	3.4509830	3.9468800	3.9351080
C	2.8344180	3.2867780	5.1230690
C	1.4585660	3.5405230	5.0852390
C	1.1689860	4.3863710	3.8946980
C	3.4078750	2.4854840	6.1077260
C	2.5247820	1.9106300	7.0603320
C	1.1379460	2.1881800	7.0313730
C	0.5831230	3.0279320	6.0330860
C	2.5592130	5.1216230	1.9220240
H	3.5356150	4.7315550	1.6050610
C	1.5599210	4.3417220	1.0536170
C	2.6419520	6.6647110	1.7805220
C	1.3006280	7.3621020	2.0499840
H	0.5361120	7.0310980	1.3445330
H	1.4249150	8.4450180	1.9438360

H	0.9422430	7.1572220	3.0613070
C	3.1422570	6.9941620	0.3608790
H	4.0976780	6.4986030	0.1578160
H	3.2957690	8.0738380	0.2657360
H	2.4268570	6.6776700	-0.3987600
C	3.6925710	7.1630180	2.7920040
H	3.3652920	6.9978670	3.8224530
H	3.8525810	8.2369670	2.6563480
H	4.6519180	6.6533960	2.6545440
C	4.8764680	2.2515490	6.1560420
C	5.7495060	3.3356790	6.3119910
H	5.3442370	4.3390970	6.3901210
C	7.1272660	3.1309070	6.3719440
H	7.7923050	3.9777320	6.5098780
C	7.6511400	1.8416280	6.2570570
H	8.7238860	1.6834000	6.2829900
C	6.7859440	0.7559590	6.1046210
H	7.1849940	-0.2503110	6.0227260
C	5.4068970	0.9582010	6.0632370
H	4.7360010	0.1136270	5.9552160
C	3.0688550	0.9964850	8.1050900
C	3.9941210	1.4580590	9.0501730
H	4.3152350	2.4940220	9.0205120
C	4.5021970	0.5954150	10.0204180
H	5.2138910	0.9667730	10.7514190
C	4.0989510	-0.7413640	10.0507160
H	4.4977060	-1.4137490	10.8040960
C	3.1808780	-1.2099790	9.1081640
H	2.8646330	-2.2484970	9.1241480
C	2.6659180	-0.3450840	8.1436010
H	1.9480880	-0.7060910	7.4141540
C	0.2364780	1.6326650	8.0813140
C	0.4140700	1.9816580	9.4266720
H	1.2266220	2.6456740	9.7021360
C	-0.4429670	1.4816670	10.4062580
H	-0.2957420	1.7635410	11.4444110
C	-1.4876530	0.6243480	10.0533740
H	-2.1551360	0.2353130	10.8161790
C	-1.6711690	0.2716130	8.7148420
H	-2.4804590	-0.3916730	8.4278070
C	-0.8157080	0.7743260	7.7352130
H	-0.9649980	0.5012640	6.6967820
C	-0.8588170	3.4078360	6.0490940
C	-1.2850740	4.4087530	6.9306910
H	-0.5596530	4.8934090	7.5768830
C	-2.6292320	4.7788320	6.9826360
H	-2.9471760	5.5596180	7.6669530

C	-3.5608260	4.1394460	6.1630430
H	-4.6095930	4.4168460	6.2089610
C	-3.1407940	3.1330700	5.2905030
H	-3.8535780	2.6017370	4.6719560
C	-1.7943640	2.7741750	5.2243300
H	-1.4808130	1.9880640	4.5450430
O	-1.7804700	3.5571510	-0.1461260
O	-1.0396900	1.6884210	0.9010680
O	-3.4267600	0.4993390	3.5933200
O	-3.3180670	0.5600620	-0.9782870
N	-3.5168520	0.8855360	1.3105730
C	-3.5013740	0.0726430	2.4564590
C	-3.6331700	-1.3314940	1.9699410
C	-3.7558270	-1.2897930	0.5765000
C	-3.5211900	0.1178180	0.1383040
C	-3.7181260	-2.5223260	2.6856950
C	-3.9667770	-3.7054390	1.9344070
C	-4.1553000	-3.6511710	0.5348180
C	-4.0488170	-2.4238370	-0.1738570
C	-3.2347030	2.3181960	1.3760200
H	-2.9761730	2.4840320	2.4272710
C	-1.9204330	2.5629370	0.6170630
C	-4.4530100	3.2335690	1.0683950
C	-4.8539690	3.2390770	-0.4159620
H	-5.1584610	2.2457570	-0.7521110
H	-5.7007330	3.9192130	-0.5571320
H	-4.0300220	3.5743900	-1.0472760
C	-4.0986470	4.6617820	1.5276970
H	-3.2664340	5.0692830	0.9515680
H	-4.9648390	5.3176050	1.3948590
H	-3.8258330	4.6766960	2.5890250
C	-5.6438570	2.7321150	1.9068160
H	-5.9564110	1.7297320	1.6012100
H	-5.4010280	2.7020850	2.9735030
H	-6.4955740	3.4061350	1.7742000
C	-3.5346750	-2.5637750	4.1623320
C	-4.5271670	-3.1015100	4.9932280
H	-5.4550910	-3.4556670	4.5570610
C	-4.3222440	-3.1967860	6.3686540
H	-5.1002310	-3.6126690	7.0014100
C	-3.1147060	-2.7717200	6.9275650
H	-2.9450300	-2.8654800	7.9959680
C	-2.1249720	-2.2270870	6.1058640
H	-1.1863340	-1.8927250	6.5358450
C	-2.3371360	-2.1083660	4.7328260
H	-1.5771020	-1.6635810	4.0993260
C	-4.0341670	-5.0207950	2.6368170

C	-5.1967630	-5.8021990	2.5928790
H	-6.0587050	-5.4452920	2.0404200
C	-5.2523670	-7.0291570	3.2523990
H	-6.1627180	-7.6194090	3.2125070
C	-4.1435660	-7.4970000	3.9608960
H	-4.1858880	-8.4543050	4.4711220
C	-2.9821560	-6.7237870	4.0140980
H	-2.1165700	-7.0751530	4.5671930
C	-2.9314320	-5.4922550	3.3631690
H	-2.0367860	-4.8843250	3.4350990
C	-4.4918860	-4.8881580	-0.2351710
C	-5.7541520	-5.0167020	-0.8301320
H	-6.4847130	-4.2240410	-0.7047870
C	-6.0676920	-6.1425150	-1.5899090
H	-7.0501550	-6.2286430	-2.0439270
C	-5.1194760	-7.1518920	-1.7727970
H	-5.3603110	-8.0246420	-2.3719170
C	-3.8603470	-7.0315480	-1.1818120
H	-3.1144970	-7.8083080	-1.3203380
C	-3.5521510	-5.9097080	-0.4133990
H	-2.5730170	-5.8152950	0.0363440
C	-4.3319340	-2.3565950	-1.6318020
C	-5.3885100	-1.5574980	-2.0931930
H	-5.9448150	-0.9486910	-1.3874370
C	-5.7415170	-1.5634660	-3.4411830
H	-6.5676850	-0.9488140	-3.7850540
C	-5.0382060	-2.3649510	-4.3444410
H	-5.3146000	-2.3730410	-5.3943490
C	-3.9705850	-3.1430420	-3.8942960
H	-3.3988160	-3.7479610	-4.5902590
C	-3.6192980	-3.1395570	-2.5461610
H	-2.7870000	-3.7417350	-2.2064830
C	4.2857740	-1.3051690	3.0452490
C	3.0553110	-1.1732880	2.4286700
C	2.7041300	0.0355160	1.7530750
C	3.6456300	1.1001810	1.7464780
C	4.8423900	0.9826000	2.4322970
C	5.1752730	-0.2211830	3.0563240
H	4.5527710	-2.2335190	3.5385320
H	2.3585980	-2.0029550	2.4420760
H	3.3928580	2.0193260	1.2438330
H	5.5057620	1.8322850	2.4901780
H	6.1224020	-0.3080670	3.5722370
C	1.4231230	0.1855840	1.1491210
C	0.3846990	-0.7834680	1.5683650
C	0.2053890	-2.0438880	0.8259330
C	0.8139490	-2.2462240	-0.4220150

C	-0.4734920	-3.0976490	1.4568280
C	0.7308250	-3.4951500	-1.0311610
H	1.3251040	-1.4283210	-0.9173980
C	-0.5358350	-4.3451950	0.8492810
H	-0.9128210	-2.9271990	2.4311010
C	0.0615590	-4.5446650	-0.3988300
H	1.1900760	-3.6589060	-1.9948170
H	-1.0337450	-5.1647890	1.3549200
O	-0.2373800	-0.4943930	2.5941240
H	0.0113720	-5.5142100	-0.8827460

10. References

(1) Green, S. P.; Wheelhouse, K. M.; Payne, A. D.; Hallett, J. P.; Miller, P. W.; Bull, J. A. Thermal Stability and Explosive Hazard Assessment of Diazo Compounds and Diazo Transfer Reagents. *Org. Process Res. Dev.* **2020**, *24* (1), 67-84. DOI: 10.1021/acs.oprd.9b00422.
(2) Davies, H. M. L.; Bruzinski, P. R.; Lake, D. H.; Kong, N.; Fall, M. J. Asymmetric Cyclopropanations by Rhodium(II) N-(Arylsulfonyl)prolinate Catalyzed Decomposition of Vinyldiazomethanes in the Presence of Alkenes. Practical Enantioselective Synthesis of the Four Stereoisomers of 2-Phenylcyclopropan-1-amino Acid. *Journal of the American Chemical Society* **1996**, *118* (29), 6897-6907. DOI: 10.1021/ja9604931.
(3) Fu, J.; Ren, Z.; Bacsa, J.; Musaev, D. G.; Davies, H. M. L. Desymmetrization of cyclohexanes by site- and stereoselective C–H functionalization. *Nature* **2018**, *564* (7736), 395-399. DOI: 10.1038/s41586-018-0799-2.
(4) Reddy, R. P.; Davies, H. M. L. Dirhodium Tetracarboxylates Derived from Adamantylglycine as Chiral Catalysts for Enantioselective C−H Aminations. *Organic Letters* **2006**, *8* (22), 5013-5016. DOI: 10.1021/ol061742l.
(5) Müller, P.; Allenbach, Y.; Robert, E. Rhodium(II)-catalyzed olefin cyclopropanation with the phenyliodonium ylide derived from Meldrum's acid. *Tetrahedron: Asymmetry* **2003**, *14* (7), 779-785. DOI: https://doi.org/10.1016/S0957-4166(03)00029-6.
(6) Qin, C.; Davies, H. M. L. Role of Sterically Demanding Chiral Dirhodium Catalysts in Site-Selective C–H Functionalization of Activated Primary C–H Bonds. *Journal of the American Chemical Society* **2014**, *136* (27), 9792-9796. DOI: 10.1021/ja504797x.
(7) Liu, W.; Ren, Z.; Bosse, A. T.; Liao, K.; Goldstein, E. L.; Bacsa, J.; Musaev, D. G.; Stoltz, B. M.; Davies, H. M. L. Catalyst-Controlled Selective Functionalization of Unactivated C–H Bonds in the Presence of Electronically Activated C–H Bonds. *Journal of the American Chemical Society* **2018**, *140* (38), 12247-12255. DOI: 10.1021/jacs.8b07534.
(8) Vaitla, J.; Boni, Y. T.; Davies, H. M. L. Distal Allylic/Benzylic C−H Functionalization of Silyl Ethers Using Donor/Acceptor Rhodium(II) Carbenes. *Angewandte Chemie International Edition* **2020**, *59* (19), 7397-7402. DOI: https://doi.org/10.1002/anie.201916530.
(9) Tan, Y.; Yuan, W.; Gong, L.; Meggers, E. Aerobic Asymmetric Dehydrogenative Cross-Coupling between Two C-H Groups Catalyzed by a Chiral-at-Metal Rhodium Complex. *Angewandte Chemie International Edition* **2015**, *54* (44), 13045-13048. DOI: https://doi.org/10.1002/anie.201506273.
(10) Denton, J. R.; Davies, H. M. L. Enantioselective Reactions of Donor/Acceptor Carbenoids Derived from α-Aryl-α-Diazoketones. *Organic Letters* **2009**, *11* (4), 787-790. DOI: 10.1021/ol802614j.
(11) Kambe, T.; Maruyama, T.; Nakano, M.; Yamaura, Y.; Shono, T.; Seki, A.; Sakata, K.; Maruyama, T.; Nakai, H.; Toda, M. Discovery of Orally Available 8-Aza-5-thiaProstaglandin E1 Analogs as Highly

Selective EP4 Agonists. *Chemical and Pharmaceutical Bulletin* **2011**, *59* (12), 1523-1534. DOI: 10.1248/cpb.59.1523.
(12) Foster, R. W.; Lenz, E. N.; Simpkins, N. S.; Stead, D. Organocatalytic Stereoconvergent Synthesis of α-CF3 Amides: Triketopiperazines and Their Heterocyclic Metamorphosis. *Chemistry – A European Journal* **2017**, *23* (37), 8810-8813. DOI: https://doi.org/10.1002/chem.201701548.
(13) Wang, H.; Denton, J. R.; Davies, H. M. L. Sequential Rhodium-, Silver-, and Gold-Catalyzed Synthesis of Fused Dihydrofurans. *Organic Letters* **2011**, *13* (16), 4316-4319. DOI: 10.1021/ol2016548.
(14) Davies, J. R.; Kane, P. D.; Moody, C. J. The Diazo Route to Diazonamide A. Studies on the Indole Bis-oxazole Fragment. *The Journal of Organic Chemistry* **2005**, *70* (18), 7305-7316. DOI: 10.1021/jo0509760.
(15) Mocci, R.; Luca, L. D.; Delogu, F.; Porcheddu, A. An Environmentally Sustainable Mechanochemical Route to Hydroxamic Acid Derivatives. *Advanced Synthesis & Catalysis* **2016**, *358* (19), 3135-3144. DOI: https://doi.org/10.1002/adsc.201600350.
(16) Gupta, M. K.; Li, Z.; Snowden, T. S. Preparation of One-Carbon Homologated Amides from Aldehydes or Primary Alcohols. *Organic Letters* **2014**, *16* (6), 1602-1605. DOI: 10.1021/ol500200n.
(17) Kuwada, T.; Yoshinaga, M.; Ishizaka, T.; Wakasugi, D.; Shirokawa, S.-I.; Hattori, N.; Shimazaki, Y.; Miyakoshi, N. Preparation of 1,2,4-triazol-3-one derivatives as arginine vasopressin 1b (V1b) receptor antagonists. WO2012043791, 2012.
(18) Landers, B.; Berini, C.; Wang, C.; Navarro, O. (N-Heterocyclic Carbene)-Pd-Catalyzed Anaerobic Oxidation of Secondary Alcohols and Domino Oxidation−Arylation Reactions. *The Journal of Organic Chemistry* **2011**, *76* (5), 1390-1397. DOI: 10.1021/jo102385u.
(19) Gooßen, L. J.; Mamone, P.; Oppel, C. Catalytic Decarboxylative Cross-Ketonisation of Aryl- and Alkylcarboxylic Acids using Magnetite Nanoparticles. *Advanced Synthesis & Catalysis* **2011**, *353* (1), 57-63. DOI: https://doi.org/10.1002/adsc.201000429.
(20) Fu, W. C.; So, C. M.; Yuen, O. Y.; Lee, I. T. C.; Kwong, F. Y. Exploiting Aryl Mesylates and Tosylates in Catalytic Mono-α-arylation of Aryl- and Heteroarylketones. *Organic Letters* **2016**, *18* (8), 1872-1875. DOI: 10.1021/acs.orglett.6b00643.
(21) Gao, K.; Yorimitsu, H.; Osuka, A. α-Arylation of Ketimines with Aryl Sulfides at a Low Palladium Catalyst Loading. *Angewandte Chemie International Edition* **2016**, *55* (14), 4573-4576. DOI: https://doi.org/10.1002/anie.201600248.
(22) Terlizzi, L. D.; Cola, I.; Raviola, C.; Fagnoni, M.; Protti, S. Dyedauxiliary Group Strategy for the α-Functionalization of Ketones and Esters. *ACS Organic & Inorganic Au* **2021**, *1* (2), 68-71. DOI: 10.1021/acsorginorgau.1c00020.
(23) Mehta, V. P.; García-López, J.-A.; Greaney, M. F. Ruthenium-Catalyzed Cascade C[illegible]H Functionalization of Phenylacetophenones. *Angewandte Chemie International Edition* **2014**, *53* (6), 1529-1533. DOI: https://doi.org/10.1002/anie.201309114.
(24) Su, Y.; Sun, X.; Wu, G.; Jiao, N. Catalyst-Controlled Highly Selective Coupling and Oxygenation of Olefins: A Direct Approach to Alcohols, Ketones, and Diketones. *Angewandte Chemie International Edition* **2013**, *52* (37), 9808-9812. DOI: https://doi.org/10.1002/anie.201303917.
(25) Wommack, A. J.; Moebius, D. C.; Travis, A. L.; Kingsbury, J. S. Diverse Alkanones by Catalytic Carbon Insertion into the Formyl C−H Bond. Concise Access to the Natural Precursor of Achyrofuran. *Organic Letters* **2009**, *11* (15), 3202-3205. DOI: 10.1021/ol9010932.
(26) Harada, H.; Asano, O.; Ueda, M.; Miyazawa, S.; Kotake, Y.; Kabasawa, Y.; Yasuda, M.; Yasuda, N.; Iida, D.; Nagakawa, J.; et al. Preparation of pyrimidine derivatives as adenosine antagonists. WO2003035639, 2003.
(27) Wertz, B.; Ren, Z.; Bacsa, J.; Musaev, D. G.; Davies, H. M. L. Comparison of 1,2-Diarylcyclopropanecarboxylates with 1,2,2-Triarylcyclopropanecarboxylates as Chiral Ligands for

Dirhodium-Catalyzed Cyclopropanation and C–H Functionalization. *The Journal of Organic Chemistry* **2020**, *85* (19), 12199-12211. DOI: 10.1021/acs.joc.0c01276.
(28) Davies, H. M. L.; Jin, Q.; Ren, P.; Kovalevsky, A. Y. Catalytic Asymmetric Benzylic C−H Activation by Means of Carbenoid-Induced C−H Insertions. *The Journal of Organic Chemistry* **2002**, *67* (12), 4165-4169. DOI: 10.1021/jo016351t.
(29) Garlets, Z. J.; Wertz, B. D.; Liu, W.; Voight, E. A.; Davies, H. M. L. Regio- and Stereoselective Rhodium(II)-Catalyzed C–H Functionalization of Cyclobutanes. *Chem* **2020**, *6* (1), 304-313. DOI: https://doi.org/10.1016/j.chempr.2019.12.014.
(30) Davies, H. M. L.; Hansen, T.; Churchill, M. R. Catalytic Asymmetric C−H Activation of Alkanes and Tetrahydrofuran. *Journal of the American Chemical Society* **2000**, *122* (13), 3063-3070. DOI: 10.1021/ja994136c.
(31) Davies, H. M. L.; Hansen, T. Asymmetric Intermolecular Carbenoid C−H Insertions Catalyzed by Rhodium(II) (S)-N-(p-Dodecylphenyl)sulfonylprolinate. *Journal of the American Chemical Society* **1997**, *119* (38), 9075-9076. DOI: 10.1021/ja971915p.
(32) White, J. D.; Choi, Y. Catalyzed Asymmetric Diels−Alder Reaction of Benzoquinone. Total Synthesis of (−)-Ibogamine. *Organic Letters* **2000**, *2* (15), 2373-2376. DOI: 10.1021/ol0001463.
